# Combined inhibition of Chk1 and Wee1 as a new therapeutic strategy for mantle cell lymphoma

**DOI:** 10.18632/oncotarget.2583

**Published:** 2014-10-25

**Authors:** Rosaria Chilà, Alessandra Basana, Monica Lupi, Federica Guffanti, Eugenio Gaudio, Andrea Rinaldi, Luciano Cascione, Valentina Restelli, Chiara Tarantelli, Francesco Bertoni, Giovanna Damia, Laura Carrassa

**Affiliations:** ^1^ Laboratory of Molecular Pharmacology and Laboratory of Cancer Pharmacology, Department of Oncology, IRCCS-Istituto di Ricerche Farmacologiche “Mario Negri”, Milan, Italy; ^2^ Lymphoma and Genomics Research Program, IOR Institute of Oncology Research, Bellinzona, Switzerland; ^3^ Lymphoma Unit, IOSI Oncology Institute of Southern Switzerland, Bellinzona, Switzerland

**Keywords:** Chk1, Wee1, targeted therapy, mantle cell lymphoma, cell cycle regulation

## Abstract

Mantle cell lymphoma (MCL) is an aggressive, incurable disease, characterized by a deregulated cell cycle. Chk1 and Wee1 are main regulators of cell cycle progression and recent data on solid tumors suggest that simultaneous inhibition of these proteins has a strong synergistic cytotoxic effect. The effects of a Chk1 inhibitor (PF-00477736) and a Wee1 inhibitor (MK-1775) have been herein investigated in a large panel of mature B-cell lymphoma cell lines. We found that MCL cells were the most sensitive to the Chk1 inhibitor PF-00477736 and Wee1 inhibitor MK-1775 as single agents. Possible involvement of the translocation t(11;14) in Chk1 inhibitor sensitivity was hypothesized. The combined inhibition of Chk1 and Wee1 was strongly synergistic in MCL cells, leading to deregulation of the cell cycle, with increased activity of CDK2 and CDK1, and activation of apoptosis. *In vivo* treatment with the drug combination of mice bearing JeKo-1 xenografts (MCL) had a marked antitumor effect with tumor regressions observed at non-toxic doses (best T/C%=0.54%). Gene expression profiling suggested effect on genes involved in apoptosis. The strong synergism observed by combining Chk1 and Wee1 inhibitors in preclinical models of MCL provides the rationale for testing this combination in the clinical setting.

## INTRODUCTION

Mantle cell lymphoma (MCL) is one of the most common lymphoma subtypes, accounting for 5–10% of all non-Hodgkin's lymphomas [1], and its prognosis is the worst among B-cell lymphomas [2, 3]. MCL is considered non curable with current therapies due to often incomplete response to initial chemotherapy and early relapse and, thus, more effective therapeutic approaches are needed [4]. The strongest prognostic markers in MCL are related to proliferation including Ki-67 staining by immunohistochemistry and a specific gene expression proliferation signature [5, 6]. Indeed deregulation of cell cycle is the characteristic pathogenic hallmark of MCL. The presence of the t(11;14)(q13;q32) translocation, which leads to the constitutive expression of the *CCND1* gene, encoding cyclin D1, is virtually present in all the cases [1, 3]. The disease is also characterized by frequent additional genetic lesions deregulating genes, such as *CDKN2A, CDK4, TP53* and *ATM*, involved in cell cycle regulation and DNA damage response [7, 8]. In normal cells genomic stability and integrity is assured by the existence of surveillance pathways that control key processes such as DNA repair, cell cycle checkpoints, apoptosis and transcription [9, 10]. The checkpoint kinases Chk1 and Wee1 are key regulators of DNA damage surveillance pathways [11–13]. Chk1 regulates the S and G2 checkpoints, replication initiation and replication fork stability [12, 14, 15]. Wee1 has a major cell cycle function in control of the G2/M transition and in ensuring faithful DNA replication [11, 12, 16]. Chk1 and Wee1 are required during normal S phase to avoid deleterious DNA breakage, and prevent loss of genome integrity in the absence of exogenous DNA damage [11, 17–19]. Experimental evidence has identified Wee1 in synthetic lethality with Chk1 [13, 20] and combined treatment with Chk1 and Wee1 inhibitors showed a strong synergistic cytotoxic effect in various human solid tumors cell lines [13, 20–22]. Little is known about the putative role and effects of Chk1 and Wee1 inhibitors in lymphomas.

We herein performed a cytotoxic screening in 35 B-cell lymphoma cell lines with Chk1 and Wee1 inhibitors. MCL were the lymphoma cell lines most sensitive to Chk1 and to a lesser extent to Wee1 inhibition. Chk1 and Wee1 inhibitors were then combined leading to a strong synergistic cytotoxic effect, affecting cell cycle and apoptosis. The combined treatment also resulted in strong antitumor activity in MCL xenografts *in vivo*. We provide the first preclinical evidence of Chk1 and Wee1 inhibitors as new therapeutic approach in MCL, which warrants investigation in a clinical setting.

## RESULTS

### MCL cell lines display a high sensitivity to Chk1 and Wee1 inhibitors as single agents

**Figure 1:**
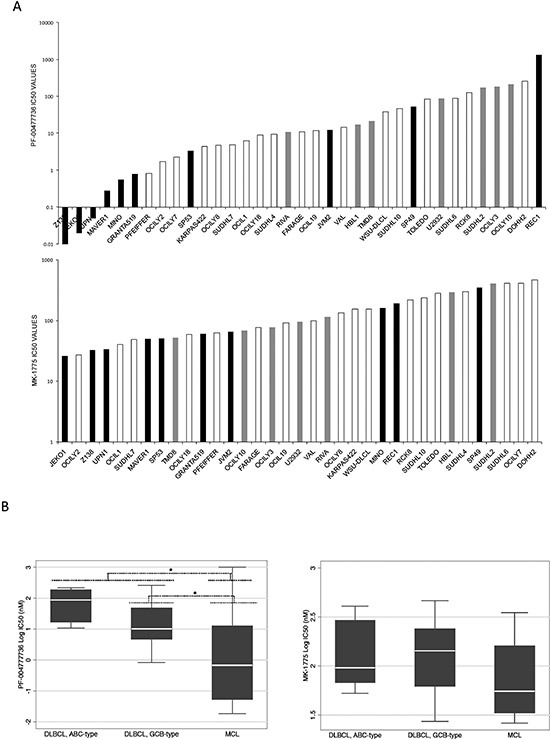
PF-00477736 and MK-1775 activity in B-cell lymphoma cell lines **(A)** PF-00477736 IC50 (upper part) and MK-1775 IC50 (lower part) in the 35 B-cell lymphoma cell lines screened. MCL: black bars; ABC-DLBCL: grey bars; GCB-DLBCL: white bars. **(B)** IC50 values distribution (left panel, PF-00477736; right panel, MK-1775) in different lymphoma pre-clinical models. In each box-plot, the line in the middle of the box represents the median and the box extends from the 25th to the 75th percentile (interquartile range, IQ); the whiskers extend to the upper and lower adjacent values (i.e., ±1.5 IQ). DLBCL, diffuse large B-cell lymphoma; ABC, activated B-cell like; GCB, germinal center B-cell; MCL, mantle cell lymphoma. *P < 0.05. Y axis, Log10 of the IC50 values in nM.

We investigated the effects of Chk1 and Wee1 inhibition in a large panel of lymphoma cell lines: 35 mature B-cell lymphoma cell lines comprising ten MCL and 25 DLBCL cell lines (seven ABC-DLBCL and 18 GCB-DLBCL), by treating them with specific Chk1 and Wee1 inhibitors, respectively PF-00477736 and MK-1775. MCL cell lines were significantly more sensitive to PF-0047736 and slightly more sensitive to MK-1775 as compared to DLBCL cell lines (Figure 1A). The median IC50 value for PF-00477736 was 0.68 nM in MCL cell lines, significantly lower than in DLBCL cell lines (p = 0.0117). PF-00477736 IC50 values were significantly lower in GCB-DLBCL (10.2 nM) than in ABC-DLBCL (87.3 nM) (p = 0.029). The MK-1775 median IC50 in MCL cells was 55.5 nM, a value lower that the one observed in DLBCL cell lines (p = 0.053) (Figure 1B). We validated the results by treating these cell lines in a 96 well plate setting. Supplementary Figure 1A summarizes IC50 values obtained in the panel of cell lines. Although the absolute IC50 values were slightly higher than the ones obtained in 384 well plates (especially for PF-00477736), the MCL cell lines (black bars) were again more sensitive to both drugs as compared to the DLBCL cells included in the validation step (Supplementary Figure 1A and C). Similar results were obtained by treating this panel of cell lines with another Chk1 inhibitor, AZD-7762 (Supplementary Figure 1B and C). Since MCL appeared the most sensitive to both compounds, we then focused on this lymphoma type.

### Sensitivity to Chk1 inhibition is associated with a high cell proliferation signature and with the presence of t(11;14)

To identify the biologic features determining the highest sensitivity to Chk1 inhibition in lymphomas, we compared the baseline gene expression profiling of 21 of the most sensitive cell lines to PF-00477736 (IC50 < 25 nM) versus the five most resistant cell lines (IC50 > 150 nM). The gene expression profiles of sensitive cell lines appeared significantly enriched of gene-sets involved in cell proliferation (Supplementary Table 1 and Supplementary Figure 2). This was true also when we limited the analysis to DLBCL cell lines only (data not shown). In accordance with a higher sensitivity of GCB-DLBCL than ABC-DLBCL, germinal center-associated gene-sets were also enriched in the transcripts higher in the sensitive cell lines, while NFKB and JAK/STAT-related gene-sets were enriched in the gene expression profiles of the resistant cell lines (Supplementary Table 1).

Since cell proliferation signatures were associated with a higher sensitivity to Chk1-inhibition and since MCL is the only lymphoma bearing the t(11;14)(q13;q32) [1] that leads to an increased activation of the CDK/cyclins involved in G1-S transition [4], we next asked if the deregulation of the cyclin D1 might be correlated with the high sensitivity to Chk1 inhibitor. As expected, cyclin D1 was constitutively expressed in the ten MCL cell lines, while not detectable in other hematological cancer cell lines (Supplementary Figure 3). To further investigate the possible role of the t(11;14) in Chk1 inhibitor sensitivity, we selected MM cell lines, with or without the t(11;14), and treated them with Chk1 inhibitor. KMS12BM and U266 cell lines displaying the t(11;14) and overexpressing cyclin D1 (Supplementary Figure 4B) were much more sensitive to the Chk1 inhibitor compared to the KMS11, RPMI8226 and OPM2 cell lines not harboring the t(11;14) translocation (Supplementary Figure 4A). In fact, the PF-00477736 mean and median IC50 were at least 40 times lower in cells with the translocation than in cells without (Supplementary Figure 4C). These data suggest that the t(11;14) may be positively correlated with the strong sensitivity of MCL cell lines to Chk1 inhibitors.

**Figure 2:**
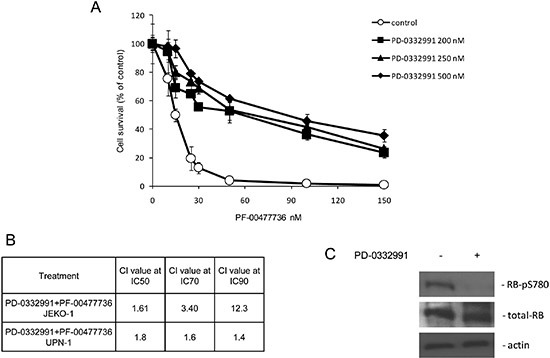
Treatment with PD-0332991 antagonizes the cytotoxic activity of PF-00477736 **(A)** Cytotoxic effects of PF-00477736 in JeKo-1 cells as single agent (control) or with different concentrations of PD-0332991. **(B)** Combination index (CI) at different concentrations of PD-0332991 combined with PF-00477736 in JeKo-1 and UPN-1 cells. **(C)** Western blot analysis showing RB-PS780, RB and actin protein levels in JeKo-1 cells either untreated or treated with PD-0332991 200 nM.

In order to better elucidate if the high activity of the CDK4/6-cyclin D1 complex is involved in the sensitivity to such inhibitor, we performed a combined treatment of PF-00477736 with a selective inhibitor of the CDK4/6-cyclin D1 complex (PD-0332991) [23] in JeKo-1 cell line. Figure 2A shows the effect of Chk1 inhibitor treatment in the presence of different non toxic concentrations of PD-0332991, being importantly antagonized. Figure 2B reports the CI values, having PD-0332991 a substantial antagonistic effect (CI > 1) when combined with PF-00477736. The results were confirmed, although at a lesser extent, in another MCL cell line, UPN-1 (Figure 2B). A slight antagonism between PF-00477736 and PD-0332991 was confirmed in the MM cell line KMS12BM with the translocation, but not in OPM2 cell line without the translocation, thus corroborating the hypothesis that this effect is limited to the t(11;14) positive cell lines (Supplementary Figure 5). The concentrations of PD-0332991 used inhibited the target of interest (Figure 2C) and were not toxic, although a slight G1 block was observed after such treatment (data not shown). These data partly suggest that the high activity of CDK4/6-cyclin D1 complex of these cells can explain sensitivity to Chk1 inhibitors; we can not however completely rule out that the little but consistent cell cycle delay observed by treating with non toxic concentrations of PD-00332991 could account for the antagonism observed.

### Combination of PF-00477736 and MK-1775 is strongly synergistic in MCL cell lines

**Figure 3:**
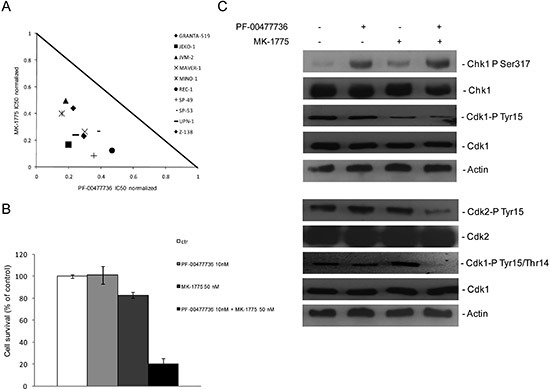
Synergistic effects of Chk1 and Wee1 inhibitors in MCL cell lines **(A)** Normalized IC50 isobologram showing the synergistic effects of the combination in 10 MCL cell lines (see legend). **(B)** JeKo-1 cell survival after 72 hrs of treatment with PF-00477736 10 nM, MK-1775 50 nM or both drugs. Data are percentages of untreated cells and represent the mean ±SD of three independent experiments. **(C)** (upper panel) Western blot analysis showing pS317-Chk1, Chk1, pY15-CDK1, CDK1 and actin protein levels in JeKo-1 cells, 24 hrs after treatment with the single drugs or the combination. (lower panel) Western blot analysis showing pY15-CDK2, CDK2, pY15/T14-CDK1, CDK1 and actin protein levels in JeKo-1 cells, 48 hrs after treatment with the single drugs or the combination.

The effect of the combined treatment with Chk1 and Wee1 inhibitors was then evaluated in all the ten MCL cell lines. The isobologram in Figure 3A summarizes the CI value of each cell line at an IC50 dose when the two drugs were combined and clearly shows a synergistic effect (CI < 1) in all the cell lines. Figure 3B schematically presents the strong synergistic effect in JeKo-1 cells with low drug concentrations. Decreased phosphorylation in Y15 CDK1, pharmacodynamic parameter of Wee1 inhibition, [24] and increased phosphorylation in S317 of Chk1, marker of Chk1 inhibition, [25] were observed 24 hrs after such treatments, suggesting that the concentrations of the two inhibitors used as single agents, although not toxic, are effective in inhibiting their targets (Figure 3C, upper part). A complete activation of CDK1 and CDK2 (involved in control of replication initiation and control of mitotic entry, respectively), was only observed after 48 hrs of exposure to the combined treatment (decrease in phosphorylation in Y15 of CDK2 and in Y15/T14 of CDK1) (Figure 3C, lower panel). These data suggest that the combined treatment leads to a general deregulation of the cell cycle.

**Figure 4:**
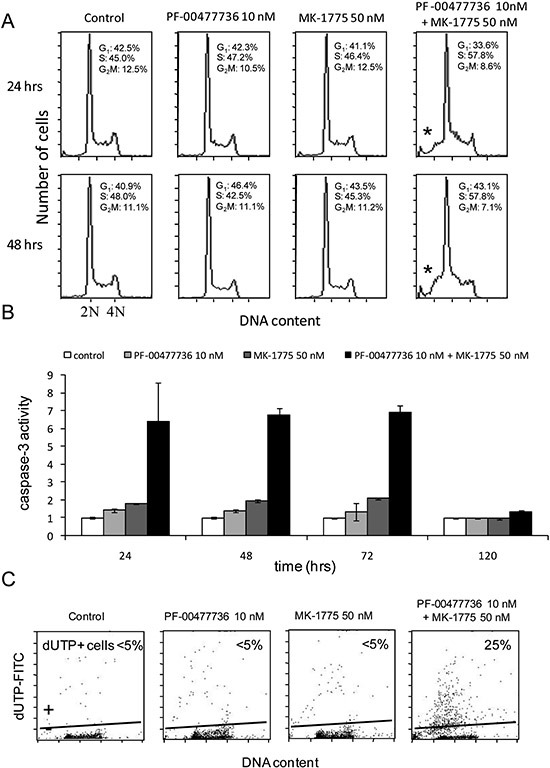
Combined treatment induces apoptosis **(A)** Analysis of DNA content by FACS after 24 and 48 hrs of treatment with the two drugs either singly or combined. Percentages of cell cycle phases (G1-S-G2/M) are included in the figure. The asterisk (*) points the sub-G1 population in the combined samples. **(B)** Activation of caspase-3 by enzymatic assay in JeKo-1 cells 24, 48, 72 and 120 hrs after treatment with PF-00477736 and MK-1775 either singly or combined. Data are represented as fold change over untreated cells and are the mean ±SD of two independent experiments. **(C)** TUNEL assay performed in JeKo-1 cells 72 hrs after treatment with the drugs singly or combined.

We performed FACS analysis in JeKo-1 cells treated for 24 and 48 hrs with the two drugs either individually or combined at concentrations not toxic as single agents. No cell cycle perturbation was observed after the single drugs, while combined treatment caused slight accumulation of cells in S phase with a DNA content between 2N and 4N starting from 24 hrs (57.8% vs 45% in control, 47,2% in PF-00477736 and 46,4% in MK-1755 samples) and persisting up to 48 hrs after treatment (57,8% vs 48% in control, 42, 5% in PF-0047736 and 45,3% in MK-1775 samples) (Figure 4A). In samples treated with the combination at both time points, there was an increase in the sub G1 population suggesting the presence of an apoptotic population. These data were corroborated by the detection of caspase-3 activity in cells treated with the two drugs, with values six times higher from 24 to 72 hrs as compared to cells untreated or treated with the single agents, and a return to basal levels by 120 hrs. (Figure 4B). Similar results were obtained also in MAVER-1 and Z-138 cells (Supplementary Figure 6). These data were corroborated by the TUNEL assay performed 72 hrs after treatment (Figure 4C).

### Combined treatment has a strong *in vivo* antitumor activity

**Figure 5:**
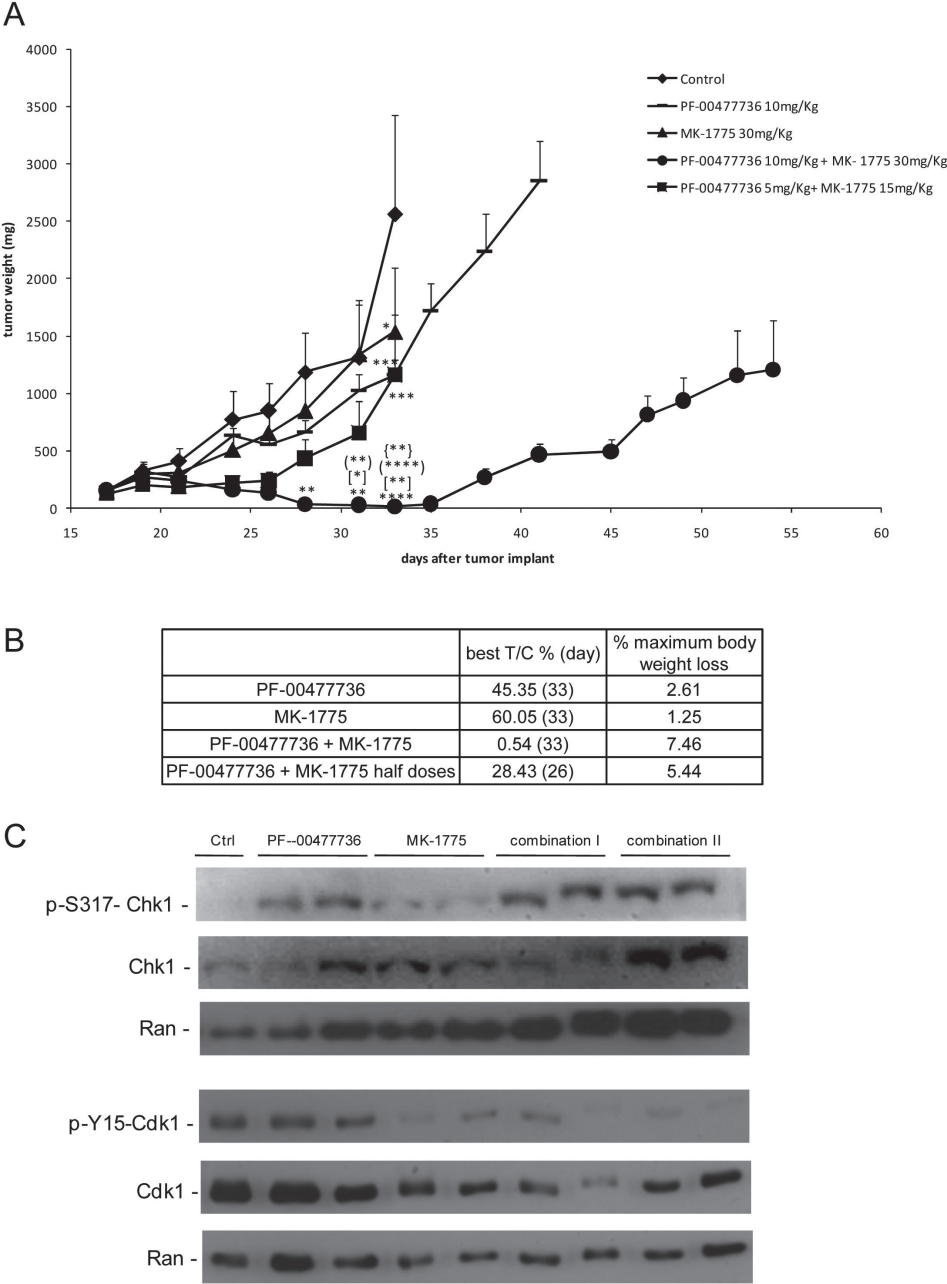
*In vivo* antitumor effect and target modulation of single and combined treatment in MCL xenografts **(A)** Tumor growth curves in JeKo-1 xenografts transplanted mice untreated and treated with PF-00477736 (daily) at 10 mg/kg, MK-1775 (twice a day) at 30 mg/kg, with both drugs at half doses for 16 days (from day 18 to day 33 from transplant); (•) group of mice treated with combination at full doses for 16 days (first cycle) and then after 5 days of treatment interruption, treated again for 11 additional days; *, *P* < 0.05; **P < 0.01, ***P < 0.001, ****P < 0.0001 compared with untreated animals; [*]*P* < 0.05 and [**]*P* < 0.01 compared with PF-00477736 treated animals; (**)*P* < 0.01 and (****)*P* < 0.0001 compared with MK-1775 treated animals; {**}*P* < 0.01 compared with half doses combined treated animals. Data are represented as the mean±SE. Anova with GraphPad Prism Software was used for statistical analysis. Tumor growth was measured three times weekly with a caliper, and tumor weights (mg = mm^3^) were calculated as follows: (length [mm] × width [mm]^2^)/2. **(B)** Antitumor activity parameters in the different experimental groups: T/C% treated/control mean tumor weight × 100 (day); % maximum mice body weight loss during treatment. **(C)** Western Blot Analysis showing pS317-Chk1, Chk1, pY15CDK1, CDK1 and Ran protein levels in protein extracts from tumor samples of the different experimental groups (Control, PF-00477736, MK-1775, Combo I and Combo II).

**Figure 6:**
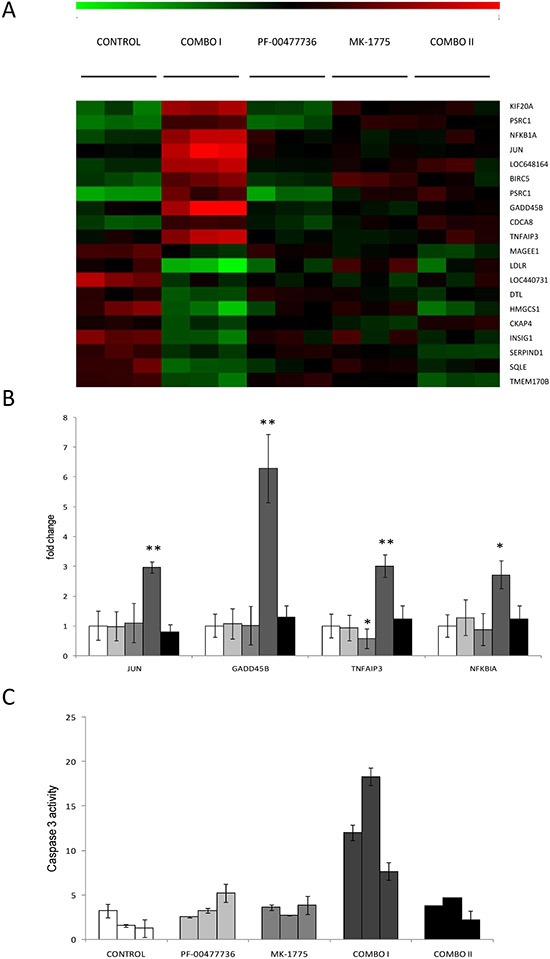
Analysis of gene expression profile and apoptosis evaluation in JeKo-1 Xenografts samples treated or not with the single or combined Chk1 and Wee1 inhibitor **(A)** Heat map showing, in each xenograft sample, the relative expression levels of the top ten up- (red) and top ten down-regulated (green) transcripts in Combo I versus the control xenografts. Expression values are log2-transformed and median-centered by transcript. (COMBO I: three days of drugs combination; COMBO II: at the end of the second cycle of drugs combination). **(B)** Up-regulation of JUN (p-value: 0.009), GADD45B (p-value: 0.002), TNFAIP3 (p-value: 0.0098), NFKBIA (p-value: 0.018) by Real Time PCR in tumor samples obtained from mice untreated (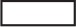), treated with single drugs (PF-00477736 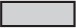 and MK-1775 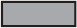), treated for three days with combined treatment (combo I, 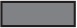) and after the end of the second cycle of treatment (combo II, 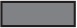). In samples treated with MK-1775 TNFAIP3 is significantly down-regulated as compared to control sample, with p-value = 0.048. **(C)** Activation of caspase-3 by enzymatic assay in tumor tissue extracts from mice treated for 3 days with the two drugs singly or combined. Data are represented as fold change of untreated cells and are the mean ±SD of two independent tissue protein extracts. COMBO I: tumor samples treated for three days with the higher drugs combination and processed three hrs after the end of the last treatment as described in Methods and Supplemental Methods. COMBO II: tumor samples treated with the higher combination for two cycles as described in Methods and taken at the end of the second cycle, three hrs after the last dose (as specified in Supplemental Methods).

The combination was tested *in vivo* in nude mice bearing JeKo-1-MCL cell line. Oral MK-1775 (30 mg/kg twice a day) and i.p. PF-00477736 (10 mg/kg once daily) were given for 16 consecutive days alone or combined. In addition, considering the striking cytotoxic activity *in vitro*, we decided to treat a group of mice with the combination at half the doses used as single agents (15 and 5 mg/kg respectively). All these treatments caused no significant body weight loss (Figure 5B). Even though the tumor weight of the single treatments differed significantly from control only on day 33, the antitumor activity was negligible as suggested by the T/C% > 45% and 60% respectively for the higher PF-00477736 and MK-1775 dose schedule (Figure 5A). However, there was a striking significant antitumor activity in mice treated with the drug combination, with T/C% of 0.54% at higher concentrations and of 28.43% at the lower concentrations (Figure 5B). At the higher dose schedule of combination, tumor regressions were observed and maintained for all the duration of the treatment. Treatment withdrawal was followed by tumor re-growth in all the mice. When a second cycle of combination treatment was given to the high dose combination group a less tumor growth inhibition as compared to the first cycle could be observed; in fact mainly tumor stabilization was observed (Figure 5). Analysis on xenografts after three days of treatment, showed that the targets were inhibited *in vivo* by one or both drugs (at the higher doses) (Figure 5C), and the treatments induced changes of transcripts that were significantly enriched of genes coding for proteins involved in cell cycle regulation and DNA damage response (Figure 6A, Supplementary Tables 2–3). Based on their known function, and on *in vitro* data of apoptosis induced by the combination, we focused on four among the ten most up-regulated genes in the first combination cycle, involved in the mechanisms of apoptosis activation: c-JUN, GADD45B, TNFAIP3 and NFKBI (Figure 6A).

These genes were validated by real time PCR, confirming their increased expression compared to all the other groups (Figure 6B). Additionally, there was a six-fold increase in caspase-3 activity in samples from the same experimental group as compared to the other ones (Figure 6C), further corroborating the *in vitro* data of induction of apoptosis by the combination.

## DISCUSSION

In recent years the development of Chk1 and Wee1 inhibitors has emerged as an effective strategy to potentiate the cytotoxic effect of chemotherapeutic drugs [11, 16, 26, 27]. The role of both Chk1 and Wee1 in regulating cell cycle progression in the absence of exogenous DNA damage, by phosphorylating CDK1 and CDK2 and by controlling DNA replication, is also an active area of investigation [12, 27]. For both roles it is widely accepted that the functions of Chk1 and Wee1 are distinct, since co-depletion of the two proteins leads to more complete hyper-activation of the two CDKs and to a more extensive replication fork slowing than with inhibition of either protein alone [13, 19]. The non redundant roles are consistent with the *in vitro* and *in vivo* data collected on the synergistic activity of combining Chk1 and Wee1 inhibitors in solid tumors [13, 20–22] which was shown to be specific for tumor cells, thus enhancing the therapeutic potential of this combination [13, 20]. Little has been reported about the activity of Chk1 and Wee1 inhibitors in hematologic malignancies. We previously reported a role of Chk1 in hematopoietic differentiation, with a peculiar kinetic of Chk1 expression during this process, showing a shift toward higher lymphoid differentiation upon Chk1 inhibition [28]. Chk1 inhibitors have been shown to be effective against mouse models Myc-driven malignancies, such as B-cell lymphoma [29], and Wee1 inhibitors enhance the efficacy of the SRC inhibitors in Burkitt lymphomas [30]. Moreover, both Chk1 and Wee1 inhibitors sensitize AML cell lines to antimetabolites chemotherapeutics, such as cytarabine, independently from p53 [31, 32]. Recently a synergism between Chk1 and Wee1 inhibitors has been described in AML [33].

Here, we have explored the effects of Chk1 and Wee1 inhibitors as single agents in a wide panel of B-cell lymphomas. Our data clearly showed that, among all the lymphoma cell lines, MCL cell lines are significantly more sensitive to the Chk1 inhibitor PF-00477736 and, even though to a lesser extent, more sensitive to the Wee1 inhibitor MK-1775. In addition, MCL cell lines were 10 and 6 times more sensitive to Chk1 and Wee1 inhibitors, respectively, than different epithelial carcinoma cell lines [13]. Due to the high difference in range of sensitivity to Chk1 inhibitor, we compared the baseline gene expression profiles of the cell lines most sensitive to Chk1 inhibitor with the most resistant ones among the entire panel of B-cell lymphoma cell lines. While cell cycle-related gene-sets were associated with a higher sensitivity to the Chk1 inhibitor, the gene expression profiles of the most resistant cell lines presented an enrichment in NFKB and JAK-STAT anti apoptotic and pro survival pathways. It is to note that among the DLBCL cell lines, those derived from the ABC subtype, characterized by activation of these pathways [34], were less sensitive than the GCB-DLBCL cell lines to PF-00477736. Studies are ongoing to better investigate this different sensitivity.

Among MCL models, only one cell line (REC-1) showed a clear resistance to PF-00477736. Although the precise biological mechanisms of this resistance have still to be elucidated, when compared to other MCL cell lines REC-1 appears more resistant to several drugs (i.e. cisplatin, doxorubicin, cytarabine, gemcitabine) [35]. Here, mutational status of p53 and ATM did not seem to be correlated to resistance, since REC-1 cell line displays both p53 and ATM wt, similarly to one of the most sensitive cell line, Z138.

**Figure 7:**
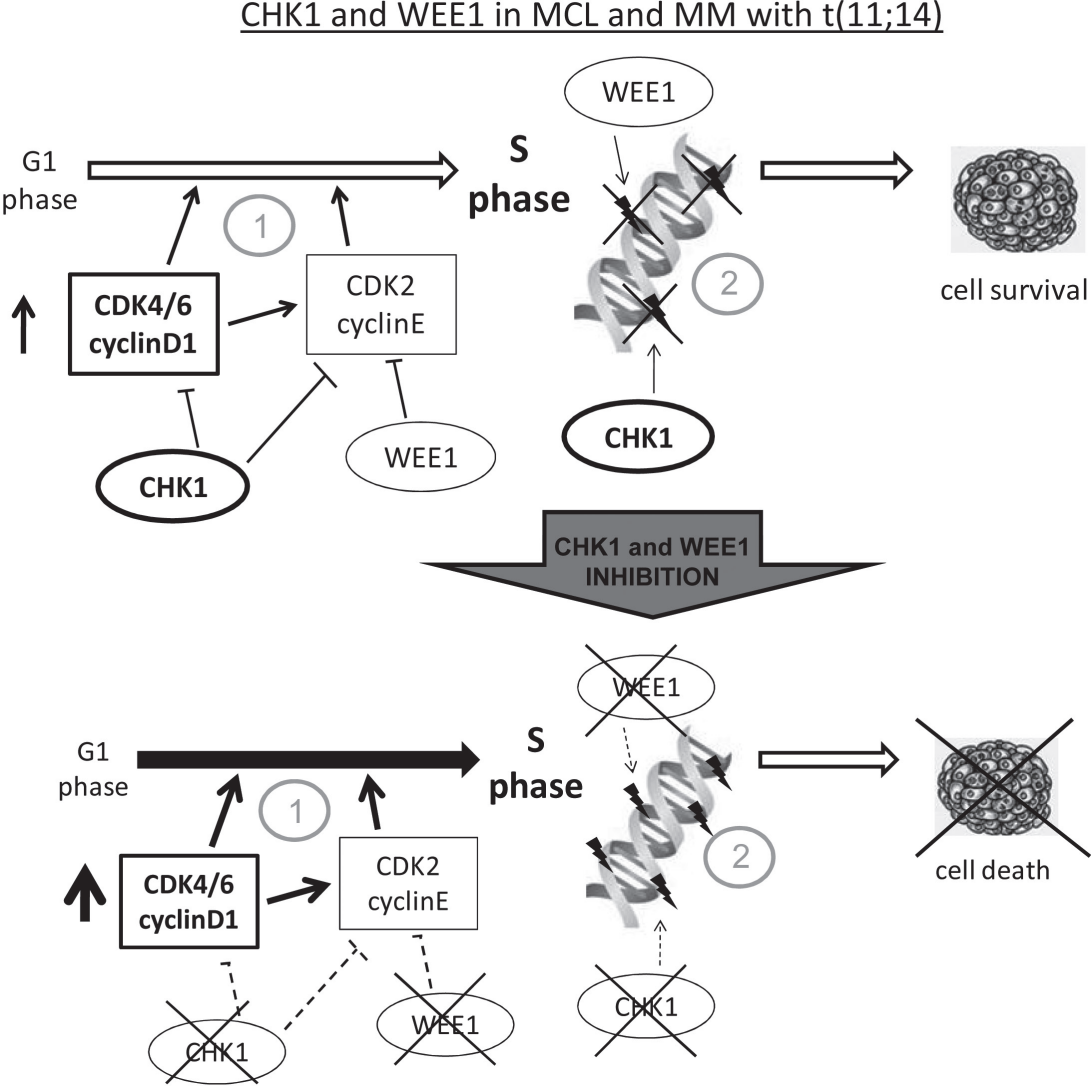
Model of Chk1 and Wee1 role in MCL and MM with t(11;14) **(A)** Cell lines with the chromosomal translocation t(11;14), have an enhanced G1-S transition due to cyclin D1 constitutive expression and are more dependent on Chk1 and Wee1 function since they are both crucial in control of initiation of DNA replication (1) and in the regulation of correct progression into S phase, minimizing endogenous DNA damage (2). Our data suggest that MCL and MM with t(11;14) rely more on Chk1 than on Wee1 activity (see text). **(B)** When both the kinases are inhibited cells undergo massive cell death because they lost the crucial protein kinases involved in control of G1 –S transition (1) and in monitoring the correct progression through S phase (2). Our data indeed show that these experimental systems are extremely susceptible to combined inhibition of Chk1 and Wee1 inhibitors at much lower concentrations than the ones used in other experimental systems. The non redundant roles played by Chk1 and Wee1 in the functions above mentioned justify the higher activity of the combination than the inhibitors used as single agents.

We did not observe any correlation between sensitivity to Chk1 inhibitor and the presence of a Myc-gene expression signature, suggesting that the high sensitivity to this class of compounds described in mouse models of Myc-driven lymphomas [29] might depend more on the deregulated cell cycle and DNA damage repair mechanisms rather than on the deregulation of Myc itself. The observed enrichment in cell proliferation and cell cycle related genes signature in the most sensitive cell lines matched with the association observed between Chk1 inhibitor sensitivity and the derivation from MCL, characterized by the presence of the t(11;14). A higher cytotoxicity to Chk1 inhibitors was indeed also observed in a panel of MM cell lines displaying the t(11;14) as compared to those without the translocation. Moreover, the inhibition of the CDK4/6-cyclin D1 complex activity, which in MCL cell lines is over-activated, with PD-0332991 [23], partly neutralized the cytotoxic effect of the Chk1 inhibitor. Modulation of cyclin D1 expression could help in elucidating its role in sensitivity to such treatments; we strenuously tried to downregulate cyclin D1 by siRNA, but we were unsuccessful. The t(11;14) deregulates the expression of *CCND1* gene, leading to increased activity of the CDK4/6-cyclin D1 complex, which by phosphorylating Rb induces the release of E2F1 transcription factor and the consequent progression of cells into S phase. In MCL and MM with the translocation the control of G1-S transition is further impaired by the increased activity of the CDK2-cyclin E complex related to the CDK4-cyclin D1 dependent sequestration of its inhibitor p27kip and by the frequent presence of CDK4 amplification and p16INK4 inactivation [4, 8, 36]. Taken together, these molecular features suggest that MCL and MM with the translocation may be even more dependent on Chk1, being crucial in regulating entry in S phase and in ensuring a correct DNA replication [12]. The sensitivity to Wee1 inhibitor does not seem to be correlated with the translocation in MM cell lines and is only slightly antagonized by the treatment with PD-0332991 (data not shown). The stronger dependence of MCL to Chk1 inhibitors than to Wee1 inhibitors may be linked to the fact that, differently from Wee1, Chk1 not only controls CDK1 and CDK2 activity, but also indirectly controls CDK4 activity through the Cdc25A [37]. In addition, it has been suggested that Chk1 has a major role in DNA repair by regulating components of homologous recombination repair pathways, involved in repair of double strand break occurring during DNA replication defects [12, 38]. The strong synergism observed with the drugs combination in MCL cell lines with very low concentrations of the two inhibitors, suggested that also in this experimental system the two protein kinases have non redundant roles. The simultaneous lack of the two main S phase progression regulators would further increase the activity of the CDKs involved in S phase entry and render MCL unable to tolerate the endogenous DNA damage developed during DNA replication (Figure 7). These events may explain the observed higher accumulation of cells in S phase after the combined treatment, which may ultimately led to cell death by apoptosis. The strong *in vitro* synergism also translated to the *in vivo* setting. Tumor regressions were observed only with the combination of Chk1 and Wee1 inhibitors and not with the single agents. The outstanding tumor growth inhibition (best T/C%: 0.54%) occurred at half of the doses used in our previous *in vivo* experiment in ovarian cancer xenografts [13]. The antitumor activity observed in MCL xenografts with the combination was stronger than the one observed in neuroblastoma xenografts treated with MK-1775 and the Chk1 inhibitor MK-8776, and comparable to what observed in colon cancer xenografts, where MK-1775 was used at much higher dose (50 mg/kg) [21, 22]. These observations strongly suggest that MCL cells respond better than other tumor models to the combination of Chk1 and Wee1 inhibitors. Activation of apoptosis was observed after three days of combination treatment *in vivo*, but it was not observed at the end of the second cycle of combination treatment after which a lower effect of the combination was observed. Gene expression profiling also showed an effect on genes involved in apoptosis: four genes involved in apoptosis were found to be up-regulated only during the first cycle of combined treatment. C-JUN transcription factor is implicated in several functions including apoptosis induction in various experimental systems such as multiple myeloma [39]; the positive mediator of FAS inducing apoptosis GADD45B, is crucial for activation of pro-apoptotic genes [40]; NFKBIA and TNFAIP3 are both negative regulators of NFKB, anti apoptotic transcription factor inducing pro-survival pathways [41, 42].

In conclusion, MCL cell lines are considerably more sensitive to Chk1 and Wee1 inhibitors as single agents than other lymphoma cell lines and epithelial tumor cell lines. Our experimental data suggested a correlation of the t(11;14) chromosomal translocation with Chk1 inhibitor sensitivity. The drugs combination presented a strong synergism at very low concentrations both in an *in vitro* and in an *in vivo* setting. The crucial non redundant role played by both proteins in control of DNA replication initiation and progression may explain why the drugs combination was highly effective in MCL cells, which are characterized by cell cycle deregulation. As a whole, our data provide strong preclinical evidence for the translation of Chk1 and Wee1 inhibitor combination in the clinical setting, hopefully providing a new therapeutic approach to treat MCL patients.

## METHODS

### Cell cultures

A total of 40 human established cell lines were used: ten cell lines derived from MCL (GRANTA-519, JeKo-1, JVM-2, MAVER-1, MINO, REC-1, SP-49, SP-53, UPN-1 and Z-138), seven (RIVA, HBL-1, TMD8, U2932, SU-DHL-2, OCILY3, OCILY10) from activated B-cell like diffuse large B-cell lymphoma (ABC-DLBCL), 18 (Pfeiffer, OCI-Ly1, OCI-Ly2, OCI-Ly7, OCI-Ly8, OCI-Ly18, OCI-Ly19, KARPAS422, SU-DHL-4, SU-DHL-6, SU-DHL-7, SU-DHL10, FARAGE, VAL, WSU-DLCL, TOLEDO, RCK8, DOHH2) from germinal center B (GCB) cell DLBCL, and five (KMS12BM, U266 KMS11, RPMI8226 and OPM2) from multiple myeloma (MM). They were maintained in RPMI supplemented with 1% glutamine, 1% penicillin/streptomycin and 10% fetal bovine serum (FBS). Details on cell lines and drugs are specified in Supplementary Methods.

### Quantification of the effect of the treatments

Cell lines were seeded in the experimental setting of 384 well plate and treated with ten growing concentrations of each drug (ranging between 0.05 nM and 100 μM) and seven replicates each concentration, using an automated liquid handling system (JANUS™, PerkinElmer), connected to a WinPREP for Janus software, with which it is possible to set up ad hoc programs for the seeding and treatments. MTS assay, performed 72 hrs after treatment, was used to measure cell proliferation using a plate reader (Infinite M200, TECAN). For validation step the experimental setting of 96 well plates was used. The IC50s of the two compounds for each cell line were calculated by Calcusyn Software. A non parametric Mann Whitney t-test statistical analysis was performed to compare median values. To obtain the response of cell lines to the combination of PF-00477736 with MK-1775, cells were treated simultaneously with growing concentrations of the two drugs. Results were examined by isobologram analysis with Calcusyn Software (Biosoft), to calculate the efficacy (combination index, CI) of the experimental points (details in Supplementary Methods).

### Western blotting analysis

Proteins were extracted and visualized using standard techniques, and as already described [43]. (Antibodies details in Supplementary Methods)

### Immunofluorescence analysis

To stain with phosphorylated H2AX (γH2AX), JeKo-1 and REC-1 cells were treated as already described [43].

### Caspase-3 activity assay

Caspase-3 activity was measured by enzymatic assay using a fluorogenic substrate for caspase-3, Ac-DEVD-AMC (acetyl Asp-Glu-Val-Asp 7-amido-4-methylcoumarin) as already described [13].

### FACS-Analysis

To detect DNA by FACS, cells were fixed 24 and 48 hrs after PF-00477736 and MK-1775 treatments either singly or combined and processed as already described [43]. Percentages of cell cycle phases (G1-S-G2/M) of Jeko-1, Maver-1 and Z-138 cell lines after treatment for 24 and 48 hrs with PF-00477736, MK-1775 and the combination were obtained analyzing DNA histograms with the previously described software [44]

### Two-parameter flow cytometry analysis: DNA content and FITC-conjugated dUTP

DNA fragmentation in JeKo-1 cells, either untreated or treated with PF-00477736, MK-1775 or the combination was detected by the TdT-mediated dUTP nick-end labeling technique (TUNEL), following a procedure already described [13].

### Analysis of gene expression and real time PCR

Total RNA was extracted from cell lines using TRIzol reagent (Invitrogen) and purified using RNeasy total RNA Isolation kit (Quiagen), and with the SV-total RNA kit according to manufacturer's instructions (Promega, Milan, Italy), for xenografts tumor fragments (homogenised in RNA lysis buffer in ice with an Ultra-Turrax), quantified by NanoDrop spectrophotometer. Gene expression profiling was done using the HumanHT-12 v4 Expression BeadChip (Illumina, San Diego, CA, USA), and data were first extracted with the Illumina GenomeStudio software and then imported in R and quantile normalized, as previously performed [45]. Functional annotation was performed using the Gene Set Enrichment Analysis (GSEA) tool using the GSEA C2 and C3.tft collections [46] and the SignatureDB gene-sets collection [47]. Differential expression analysis was performed using LIMMA [48]. Statistical significance was multiple test corrected using the Benjamini & Hochberg method. Transcripts with FDR < 0.10 were considered statically deregulated. Raw data will be available at the National Center for Biotechnology Information Gene Expression Omnibus (http://www.ncbi.nlm.nih.gov/geo) database. Real time PCR was performed as described in Supplemental Methods.

### Xenografts models

Five-week-old female NCr-nu/nu mice were obtained from Harlan S.p.a Italy and maintained under specific pathogen-free conditions. Procedures involving animals and their care were conducted in conformity with institutional guidelines, in compliance with national and international laws and policies and in line with Guidelines for the welfare and use of animals in cancer research [49]. Exponentially growing JeKo-1 cells were injected subcutaneously in three mice (approximately 10^7^ cells per mouse). When tumors grew, animals were sacrificed and tumor fragments were implanted subcutaneously in sixty mice. When tumors reached approximately 150 mg, animals were randomized (seven or eight per group) to receive PF-00477736 i.p., 10 mg/kg, MK-1775 orally twice a day, 30 mg/kg or the combination of both at these dosages or at half doses, for 16 days (COMBO I). A second cycle of treatment was given to the high dose combination group for 11 days, after 5 days treatment withdraw, when tumors started to re-grow (COMBO II). Animals were sacrificed when tumor weight reached the 10% of their body weight. Pharmacodynamic studies are described in Supplementary Methods.

## SUPPLEMENTARY METHODS, FIGURES AND TABLES







## References

[1] Swerdlow SH, Campo E, Seto M, Muller-Hermelink HK, Swerdlow S, Campo E, Harris NL, Jaffe ES, Pileri SA, Stein H, Thiele J, Vardiman JW (2008). Mantle cell lymphoma. WHO Classification of Tumours of Haematopoietic and Lymphoid Tissues.

[2] Ghielmini M, Zucca E (2009). How I treat mantle cell lymphoma. Blood.

[3] Dreyling M, Thieblemont C, Gallamini A, Arcaini L, Campo E, Hermine O, Kluin-Nelemans JC, Ladetto M, Le Gouill S, Iannitto E, Pileri S, Rodriguez J, Schmitz N, Wotherspoon A, Zinzani P, Zucca E (2013). ESMO Consensus conferences: guidelines on malignant lymphoma. part 2: marginal zone lymphoma, mantle cell lymphoma, peripheral T-cell lymphoma. Ann Oncol.

[4] Perez-Galan P, Dreyling M, Wiestner A (2011). Mantle cell lymphoma: biology, pathogenesis, and the molecular basis of treatment in the genomic era. Blood.

[5] Rosenwald A, Wright G, Wiestner A, Chan WC, Connors JM, Campo E, Gascoyne RD, Grogan TM, Muller-Hermelink HK, Smeland EB, Chiorazzi M, Giltnane JM, Hurt EM, Zhao H, Averett L, Henrickson S (2003). The proliferation gene expression signature is a quantitative integrator of oncogenic events that predicts survival in mantle cell lymphoma. Cancer Cell.

[6] Raty R, Franssila K, Joensuu H, Teerenhovi L, Elonen E (2002). Ki-67 expression level, histological subtype, and the International Prognostic Index as outcome predictors in mantle cell lymphoma. Eur J Haematol.

[7] Greiner TC, Moynihan MJ, Chan WC, Lytle DM, Pedersen A, Anderson JR, Weisenburger DD (1996). p53 mutations in mantle cell lymphoma are associated with variant cytology and predict a poor prognosis. Blood.

[8] Pinyol M, Hernandez L, Cazorla M, Balbin M, Jares P, Fernandez PL, Montserrat E, Cardesa A, Lopez-Otin C, Campo E (1997). Deletions and loss of expression of p16INK4a and p21Waf1 genes are associated with aggressive variants of mantle cell lymphomas. Blood.

[9] Poehlmann A, Roessner A (2010). Importance of DNA damage checkpoints in the pathogenesis of human cancers. Pathol Res Pract.

[10] Bartek J, Lukas J (2007). DNA damage checkpoints: from initiation to recovery or adaptation. Curr Opin Cell Biol.

[11] Stathis A, Oza A (2010). Targeting Wee1-like protein kinase to treat cancer. Drug News Perspect.

[12] Sorensen CS, Syljuasen RG (2012). Safeguarding genome integrity: the checkpoint kinases ATR, CHK1 and WEE1 restrain CDK activity during normal DNA replication. Nucleic Acids Res.

[13] Carrassa L, Chila R, Lupi M, Ricci F, Celenza C, Mazzoletti M, Broggini M, Damia G (2012). Combined inhibition of Chk1 and Wee1: *in vitro* synergistic effect translates to tumor growth inhibition *in vivo*. Cell Cycle.

[14] Zhao H, Watkins JL, Piwnica-Worms H (2002). Disruption of the checkpoint kinase 1/cell division cycle 25A pathway abrogates ionizing radiation-induced S and G2 checkpoints. Proc Natl Acad Sci U S A.

[15] Petermann E, Woodcock M, Helleday T (2010). Chk1 promotes replication fork progression by controlling replication initiation. Proc Natl Acad Sci U S A.

[16] Vriend LE, De Witt Hamer PC, Van Noorden CJ, Wurdinger T (2013). WEE1 inhibition and genomic instability in cancer. Biochim Biophys Acta.

[17] Beck H, Nahse V, Larsen MS, Groth P, Clancy T, Lees M, Jorgensen M, Helleday T, Syljuasen RG, Sorensen CS (2010). Regulators of cyclin-dependent kinases are crucial for maintaining genome integrity in S phase. J Cell Biol.

[18] Beck H, Nahse-Kumpf V, Larsen MS, O'Hanlon KA, Patzke S, Holmberg C, Mejlvang J, Groth A, Nielsen O, Syljuasen RG, Sorensen CS (2012). Cyclin-dependent kinase suppression by WEE1 kinase protects the genome through control of replication initiation and nucleotide consumption. Mol Cell Biol.

[19] Dominguez-Kelly R, Martin Y, Koundrioukoff S, Tanenbaum ME, Smits VA, Medema RH, Debatisse M, Freire R (2011). Wee1 controls genomic stability during replication by regulating the Mus81-Eme1 endonuclease. J Cell Biol.

[20] Davies KD, Cable PL, Garrus JE, Sullivan FX, von Carlowitz I, Huerou YL, Wallace E, Woessner RD, Gross S (2011). Chk1 inhibition and Wee1 inhibition combine synergistically to impede cellular proliferation. Cancer Biol Ther.

[21] Russell MR, Levin K, Rader J, Belcastro L, Li Y, Martinez D, Pawel B, Shumway SD, Maris JM, Cole KA (2013). Combination therapy targeting the Chk1 and Wee1 kinases shows therapeutic efficacy in neuroblastoma. Cancer Res.

[22] Guertin AD, Martin MM, Roberts B, Hurd M, Qu X, Miselis NR, Liu Y, Li J, Feldman I, Benita Y, Bloecher A, Toniatti C, Shumway SD (2012). Unique functions of CHK1 and WEE1 underlie synergistic anti-tumor activity upon pharmacologic inhibition. Cancer Cell Int.

[23] Leonard JP, LaCasce AS, Smith MR, Noy A, Chirieac LR, Rodig SJ, Yu JQ, Vallabhajosula S, Schoder H, English P, Neuberg DS, Martin P, Millenson MM, Ely SA, Courtney R, Shaik N (2012). Selective CDK4/6 inhibition with tumor responses by PD0332991 in patients with mantle cell lymphoma. Blood.

[24] Rajeshkumar NV, De Oliveira E, Ottenhof N, Watters J, Brooks D, Demuth T, Shumway SD, Mizuarai S, Hirai H, Maitra A, Hidalgo M (2011). MK-1775, a potent Wee1 inhibitor, synergizes with gemcitabine to achieve tumor regressions, selectively in p53-deficient pancreatic cancer xenografts. Clin Cancer Res.

[25] Leung-Pineda V, Ryan CE, Piwnica-Worms H (2006). Phosphorylation of Chk1 by ATR is antagonized by a Chk1-regulated protein phosphatase 2A circuit. Mol Cell Biol.

[26] Carrassa L, Damia G (2011). Unleashing Chk1 in cancer therapy. Cell Cycle.

[27] McNeely S, Beckmann R, Bence Lin AK (2014). CHEK again: revisiting the development of CHK1 inhibitors for cancer therapy. Pharmacol Ther.

[28] Carrassa L, Montelatici E, Lazzari L, Zangrossi S, Simone M, Broggini M, Damia G (2010). Role of Chk1 in the differentiation program of hematopoietic stem cells. Cell Mol Life Sci.

[29] Ferrao PT, Bukczynska EP, Johnstone RW, McArthur GA (2012). Efficacy of CHK inhibitors as single agents in MYC-driven lymphoma cells. Oncogene.

[30] Cozzi M, Giorgi F, Marcelli E, Pentimalli F, Forte IM, Schenone S, D'Urso V, De Falco G, Botta M, Giordano A, Indovina P (2012). Antitumor activity of new pyrazolo [3, 4-d] pyrimidine SRC kinase inhibitors in Burkitt lymphoma cell lines and its enhancement by WEE1 inhibition. Cell Cycle.

[31] Schenk EL, Koh BD, Flatten KS, Peterson KL, Parry D, Hess AD, Smith BD, Karp JE, Karnitz LM, Kaufmann SH (2012). Effects of selective checkpoint kinase 1 inhibition on cytarabine cytotoxicity in acute myelogenous leukemia cells in vitro. Clin Cancer Res.

[32] Van Linden AA, Baturin D, Ford JB, Fosmire SP, Gardner L, Korch C, Reigan P, Porter CC (2013). Inhibition of Wee1 Sensitizes Cancer Cells to Antimetabolite Chemotherapeutics In Vitro and In Vivo, Independent of p53 Functionality. Mol Cancer Ther.

[33] Chaudhuri L, Vincelette ND, Koh BD, Naylor RM, Flatten KS, Peterson KL, McNally A, Gojo I, Karp JE, Mesa RA, Sproat LO, Bogenberger JM, Kaufmann SH, Tibes R (2014). CHK1 and WEE1 inhibition combine synergistically to enhance therapeutic efficacy in acute myeloid leukemia ex vivo. Haematologica.

[34] Shaffer AL, Young RM, Staudt LM (2012). Pathogenesis of human B cell lymphomas. Annu Rev Immunol.

[35] Rolland D, Raharijaona M, Barbarat A, Houlgatte R, Thieblemont C (2010). Inhibition of GST-pi nuclear transfer increases mantle cell lymphoma sensitivity to cisplatin, cytarabine, gemcitabine, bortezomib and doxorubicin. Anticancer Res.

[36] Quintanilla-Martinez L, Davies-Hill T, Fend F, Calzada-Wack J, Sorbara L, Campo E, Jaffe ES, Raffeld M (2003). Sequestration of p27Kip1 protein by cyclin D1 in typical and blastic variants of mantle cell lymphoma (MCL): implications for pathogenesis. Blood.

[37] Bertero T, Gastaldi C, Bourget-Ponzio I, Mari B, Meneguzzi G, Barbry P, Ponzio G, Rezzonico R (2013). CDC25A targeting by miR-483-3p decreases CCND-CDK4/6 assembly and contributes to cell cycle arrest. Cell Death Differ.

[38] Sorensen CS, Hansen LT, Dziegielewski J, Syljuasen RG, Lundin C, Bartek J, Helleday T (2005). The cell-cycle checkpoint kinase Chk1 is required for mammalian homologous recombination repair. Nat Cell Biol.

[39] Podar K, Raab MS, Tonon G, Sattler M, Barila D, Zhang J, Tai YT, Yasui H, Raje N, DePinho RA, Hideshima T, Chauhan D, Anderson KC (2007). Up-regulation of c-Jun inhibits proliferation and induces apoptosis via caspase-triggered c-Abl cleavage in human multiple myeloma. Cancer Res.

[40] Cho HJ, Park SM, Hwang EM, Baek KE, Kim IK, Nam IK, Im MJ, Park SH, Bae S, Park JY, Yoo J (2010). Gadd45b mediates Fas-induced apoptosis by enhancing the interaction between p38 and retinoblastoma tumor suppressor. J Biol Chem.

[41] Honma K, Tsuzuki S, Nakagawa M, Tagawa H, Nakamura S, Morishima Y, Seto M (2009). TNFAIP3/A20 functions as a novel tumor suppressor gene in several subtypes of non-Hodgkin lymphomas. Blood.

[42] Cabannes E, Khan G, Aillet F, Jarrett RF, Hay RT (1999). Mutations in the IkBa gene in Hodgkin's disease suggest a tumour suppressor role for IkappaBalpha. Oncogene.

[43] Carrassa L, Sanchez Y, Erba E, Damia G (2009). U2OS cells lacking Chk1 undergo aberrant mitosis and fail to activate the spindle checkpoint. J Cell Mol Med.

[44] Ubezio P (1985). Microcomputer experience in analysis of flow cytometric DNA distributions. Comput Programs Biomed.

[45] Bonetti P, Testoni M, Scandurra M, Ponzoni M, Piva R, Mensah AA, Rinaldi A, Kwee I, Tibiletti MG, Iqbal J, Greiner TC, Chan WC, Gaidano G, Piris MA, Cavalli F, Zucca E (2013). Deregulation of ETS1 and FLI1 contributes to the pathogenesis of diffuse large B-cell lymphoma. Blood.

[46] Subramanian A, Tamayo P, Mootha VK, Mukherjee S, Ebert BL, Gillette MA, Paulovich A, Pomeroy SL, Golub TR, Lander ES, Mesirov JP (2005). Gene set enrichment analysis: a knowledge-based approach for interpreting genome-wide expression profiles. Proc Natl Acad Sci U S A.

[47] Shaffer AL, Wright G, Yang L, Powell J, Ngo V, Lamy L, Lam LT, Davis RE, Staudt LM (2006). A library of gene expression signatures to illuminate normal and pathological lymphoid biology. Immunol Rev.

[48] Smyth GK, Gentleman R, Carey V, Dudoit S, Irizarry R, Huber W (2005). Limma: linear models for microarray data. Bioinformatics and Computational Biology Solutions using R and Bioconductor.

[49] Workman P, Aboagye EO, Balkwill F, Balmain A, Bruder G, Chaplin DJ, Double JA, Everitt J, Farningham DA, Glennie MJ, Kelland LR, Robinson V, Stratford IJ, Tozer GM, Watson S, Wedge SR (2010). Guidelines for the welfare and use of animals in cancer research. Br J Cancer.

