# Supplementary material for: Combined inhibition of Chk1 and Wee1 as a new therapeutic strategy for mantle cell lymphoma
Source: Oncotarget. 2014 Oct 25;6(5):3394–408. doi: 10.18632/oncotarget.2583 (PMC4413661; doi:10.18632/oncotarget.2583)
Supplement: Supplementary file 1 [file oncotarget-06-3394-s001.pdf]

SUPPLEMENTARY METHODS, FIGURES AND TABLES

Cell lines and drugs

REC1 and DOHH2 were kindly provided by Finbarr Cotter (London, UK), MAVER-1 by Alberto Zamò (Verona, IT), JEKO-1 by Eisaku Kondo, (Okayama, JP), JVM-2 by Elias Campo (Barcelona, SP), SP-49, SP-53, MINO, Z-138 and UPN-1 by Robert Kridel (Vancouver, Canada), OCI-Ly1, OCI-Ly2, OCI-Ly3, OCI-Ly7, OCI-Ly8, OCI-Ly10, OCI-Ly18, RCK8, SU-DHL-2, SU-DHL-4, SU-DHL-7, SU-DHL-10, RIVA by Laura Pasqualucci (New York, NY, USA), Toledo by Miguel A. Piris (Santander, SP), HBL1, TMD8, by Fabio Martinon (Lausanne, CH), VAL by José Ángel Martinez-Climent (Pamplona, SP), SU-DHL-6 and OCI-Ly19, by Louis M. Staudt (Bethesda, MD, USA), U2932 by Bettina Borisch (Geneva, CH), WSU-DLCL2 by Stefano Casola (Milan, IT), KMS-11 and KMS12BM by Antonino Neri (Milan, IT), while the other cell lines were obtained either from the American Type Culture Collection (ATCC: Pfeiffer, FARAGE), or from the Deutsche Sammlung von Mikro-organismen und Zellkulturen (DSMZ; GRANTA-519, KARPAS422). Cell line authentication was carried out by the authors within the last 6 months. The Wee1 inhibitor MK-1775 (Chemietek) was dissolved in DMSO in a stock solution of 20 mM and stored at -20°C. The Chk1 inhibitor PF-00477736 (Axon Medchem) was dissolved in DMSO in a stock solution of 10 mM and stored at -20°C. AZD-7762 (Selleckchem) was dissolved in DMSO in a stock solution of 20 mM. CDK4/6 inhibitor PD-0332991 (Axon Medchem) was dissolved in DMSO in a stock solution of 20 mM and stored at -20°C. For in vivo treatments PF-00477736 was dissolved in 4% dextrose in 50 nM of sodium acetate pH 4 and made fresh every 5 days, while MK-1775 was dissolved in Methylcellulose 0.5%, kept in the dark continuously stirring, and made fresh every 4 days.

Quantification of the effect of the treatments

The concentrations of the two drugs exerting a 50% antiproliferative effect when combined were normalized to the IC50 of the single drugs. The effect of the combination was evaluated independently in each cell line and all the resulting IC50s CI were plotted on the same isobologram graph for direct comparison. Additive combinations fall along the diagonal line connecting the IC50 normalized for the single drugs, synergistic combinations below the line and antagonistic above it. Each point represents the CI for a specific cell line when the two drugs were combined to reduce viability of 50%. The treatments of Jeko-1 cells with growing concentrations of PD-0332991 in combination with PF-00477736 or MK-1775 were conducted as just described.

Antibodies

Primary anti Chk1 (G4), pT14/Y15-Cdk1, Cdk1, Cdk2, cyclin D1, Rb, actin and ß-tubulin were purchased from Santa Cruz Biotechnology. Primary anti pS317-Chk1, pY15-Cdc2 and pS780 Rb were purchased from Cell Signaling Technology. Primary anti pY15-Cdk2 was purchased from Abcam. The mouse monoclonal anti-Ran (clone 20) is from BD Transduction Laboratories.

Real Time PCR

RNA was retro-transcribed to cDNA using High Capacity cDNA Reverse Transcription Kit (Applied Biosystem). Optimal primer pairs were chosen for each gene of interest using PRIMER-3 software (Supplementary Table 4). Differences in gene expression were determined by real time RT-PCR performed with Sybr Green PCR master mix (Applied Biosystem) and the curve of dissociation was evaluated for each gene. Samples were then normalized using the expression of the housekeeping gene (actin) and their levels were compared to control samples. Real-time PCR was done using the 7900HT Sequence Detection System (Applied Biosystems). An unpaired parametric t-test Analysis statistical analysis was performed on validated samples.

Pharmacodynamic

For pharmacodynamic studies, tumor-bearing mice were given PF-00477736, MK-1775 or both for three days. Animals were sacrified three hours after the last treatment and tumors were dissected and snap-frozen. The same time schedule was applied for the samples that were snap frozen after the end of the second cycle of drugs combination (COMBO II). The frozen specimens were homogenized in protein lysis buffer, loaded on SDS-PAGE, and immunoblotted with specific antibodies, and caspase-3 activity measured as previously described. In parallel some fragments of the tumors were lysed in RNA lysis buffer and RNA was extracted for the subsequent analysis of gene expression profile (as described in dedicated section).

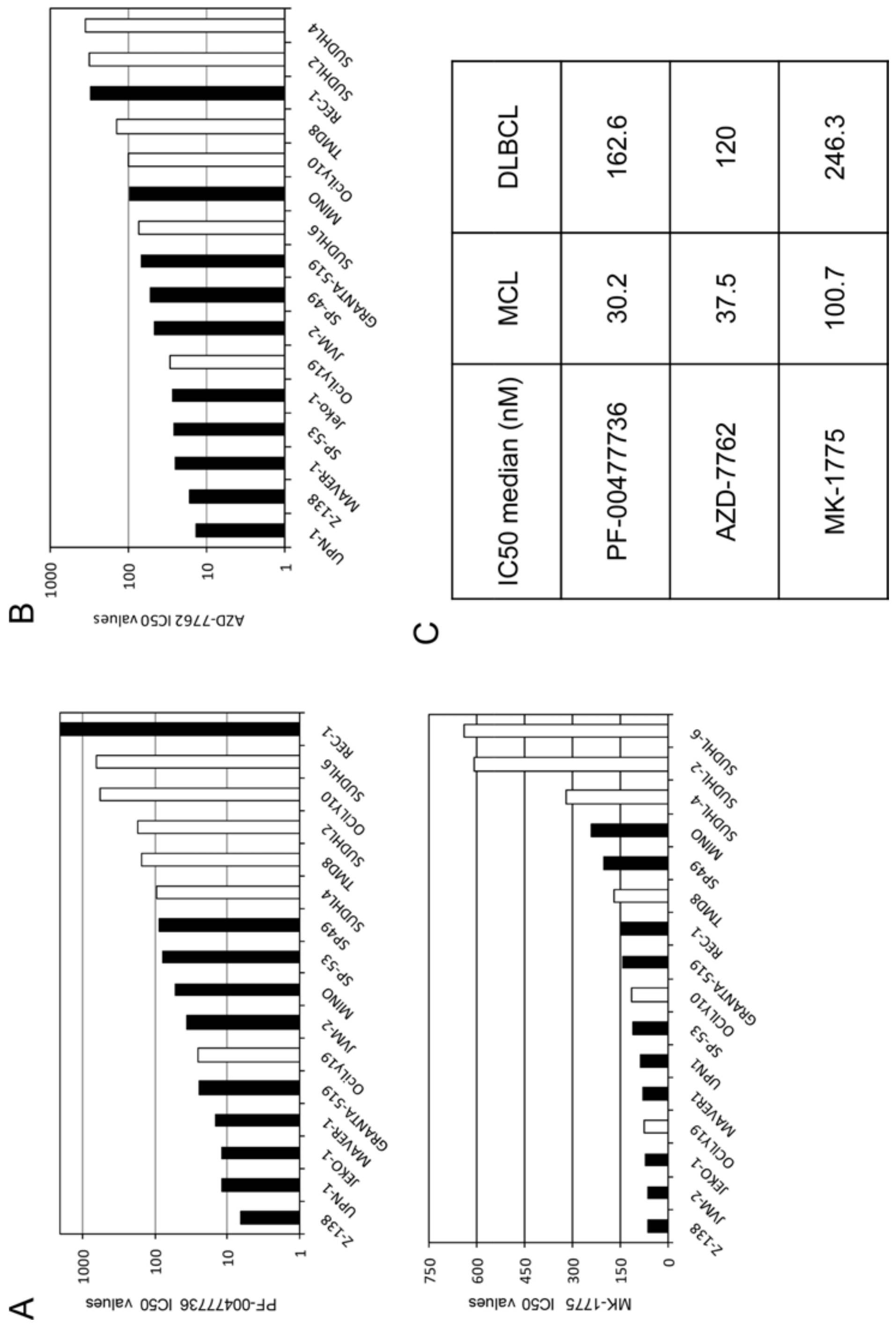

IC50 median (nM)	MCL	DLBCL
PF-00477736	30.2	162.6
AZD-7762	37.5	120
MK-1775	100.7	246.3

supplementary Figure S1: (A) PF-00477736 IC50 (upper panel) and MK-1775 IC50 (lower panel) in the ten MCL and six DLBCL included in the validation step. **(B)** AZD-7762 IC50 in the same panel of MCL and DLBCL cell lines. **(C)** PF-00477736, AZD-7762 and MK-1775 median values in MCL and DLBCL. The PF-00477736 median IC50 value in MCL cell lines was 30.2 nM, significantly lower than in the DLBCL lymphoma cell lines (p = 0.0312); the AZD-7762 median IC50 value is also significantly lower than in the DLBCL lymphoma cell lines (p = 0.0242), while the median MK-1775 IC50 value in MCL was not significantly different, but lower than in the DLBCL.

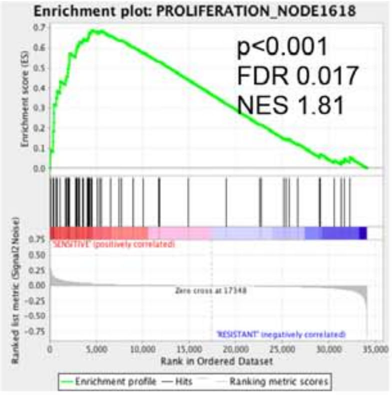

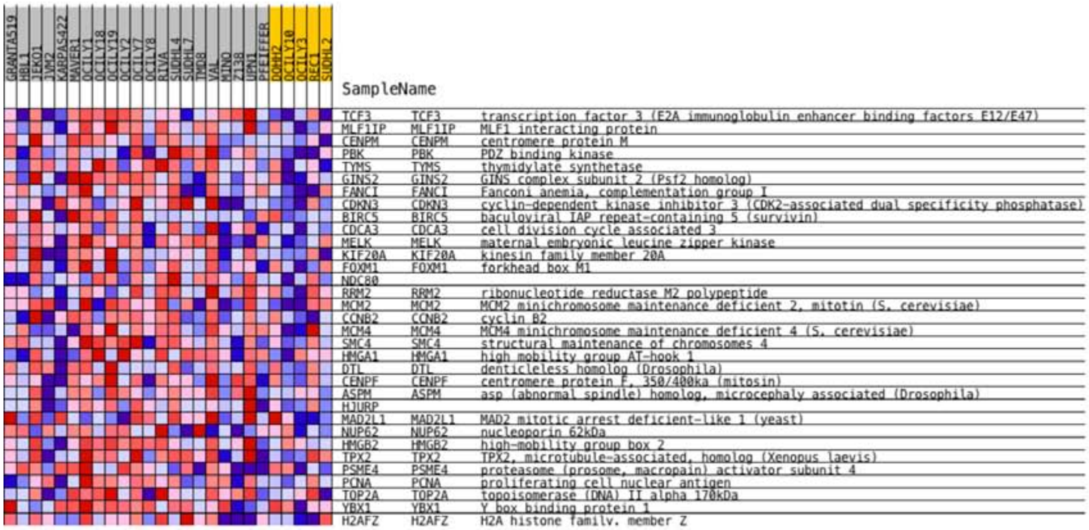

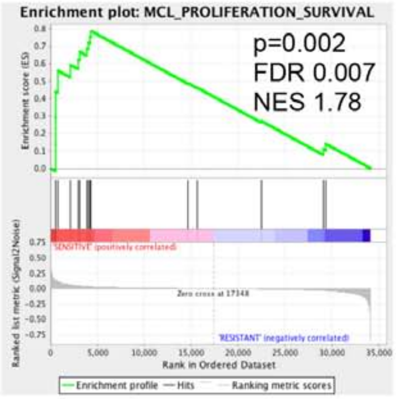

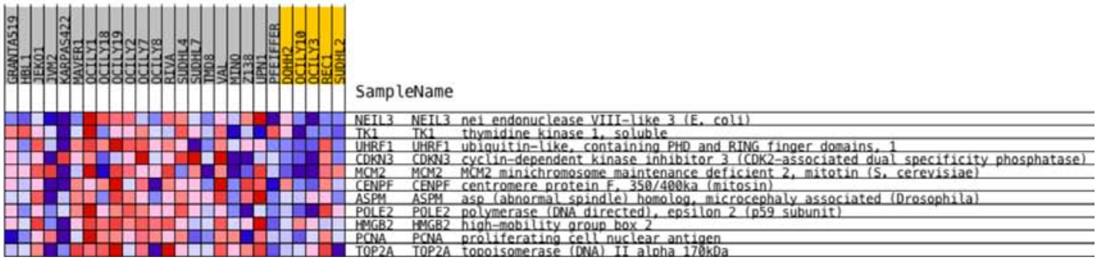

Supplementary Figure S2: GSEA plots and the corresponding heatmap of the leading edge genes of two representative cell proliferation-related gene-sets significantly enriched among the gene expression profiles of the most sensitive cell lines. FDR, false discovery rate; NES, normalized enrichment score. The heatmaps show the relative expression values for the genes part of the core enrichment.

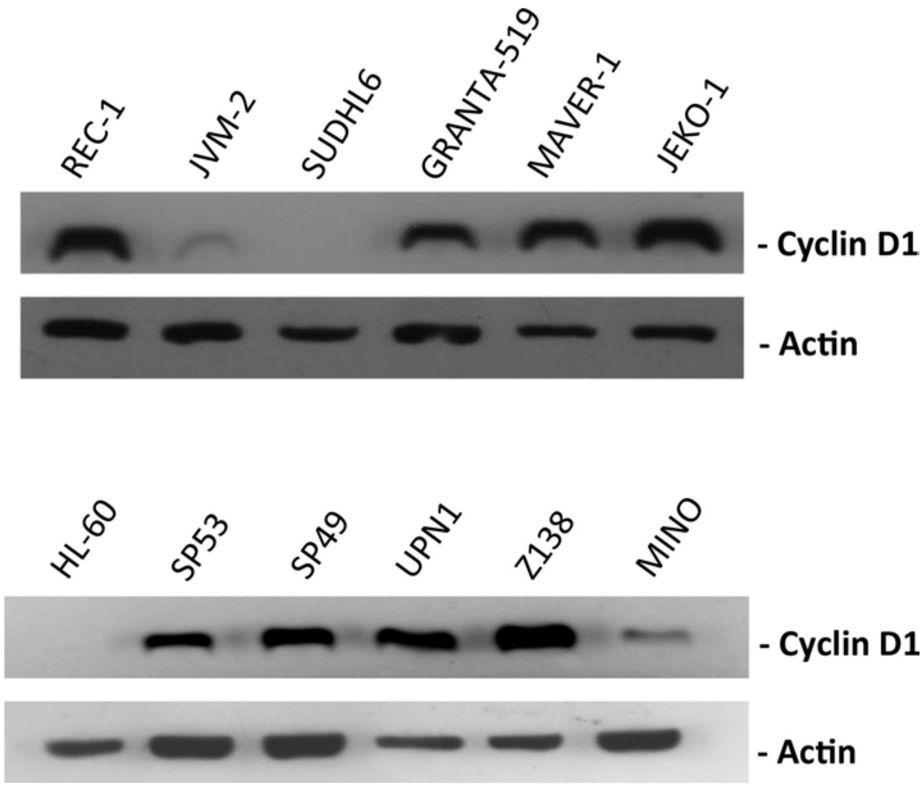

Supplementary Figure S3: Cyclin D1 and actin protein levels in ten MCL cell lines, in SUDHL6 (DLBCL) and in HL-60 (Promyelocytic Leukemia cell line).

A

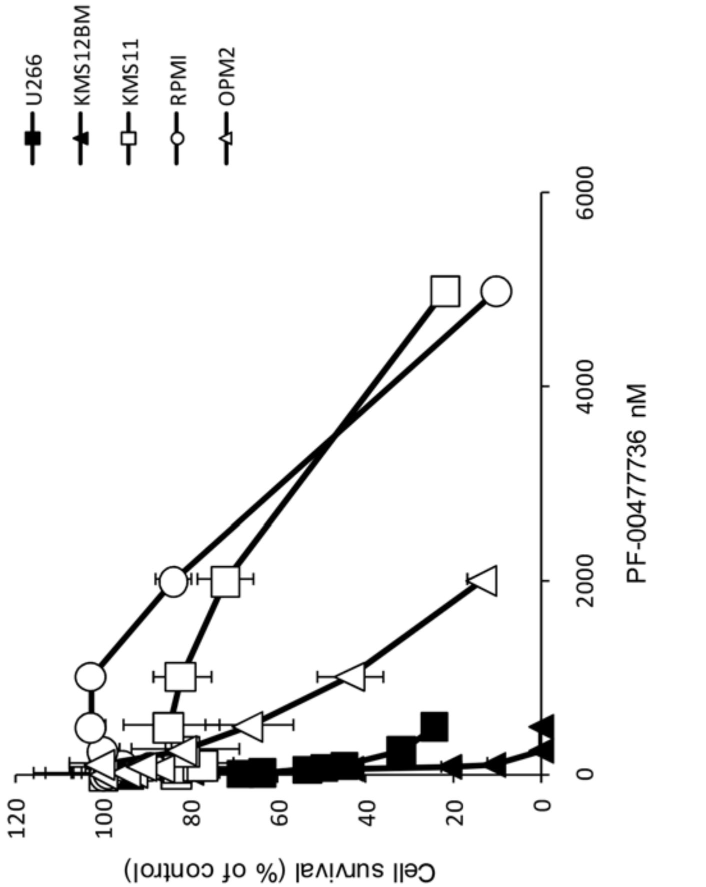

B

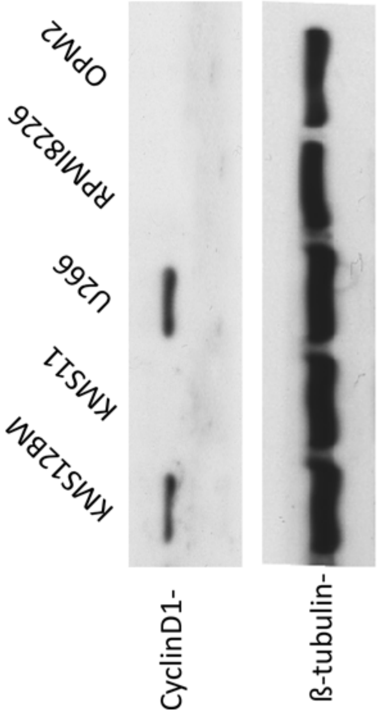

C

PF-00477736			
t(11;14)	CELL LINE	IC50 MEAN (nM)	IC50 MEDIAN (nM)
NO	OPM2	680.6±155	2605.1
	RPMI8226	4322.6±135	
	KMS11	2605.1±330	
YES	KMS12BM	33.9±7.6	57.25
	U266	80.6±25	

Supplementary Figure S4: (A) Cytotoxic effects of PF-00477736 in MM cell lines with the translocation t(11;14) (black indicators) and without the translocation (white indicators) **(B)** Western blot analysis showing Cyclin D1 and ß-tubulin protein levels in the MM cell lines **(C)** PF-00477736 IC50 (mean ±SD of three independent experiments) and median in 5 MM cell lines.

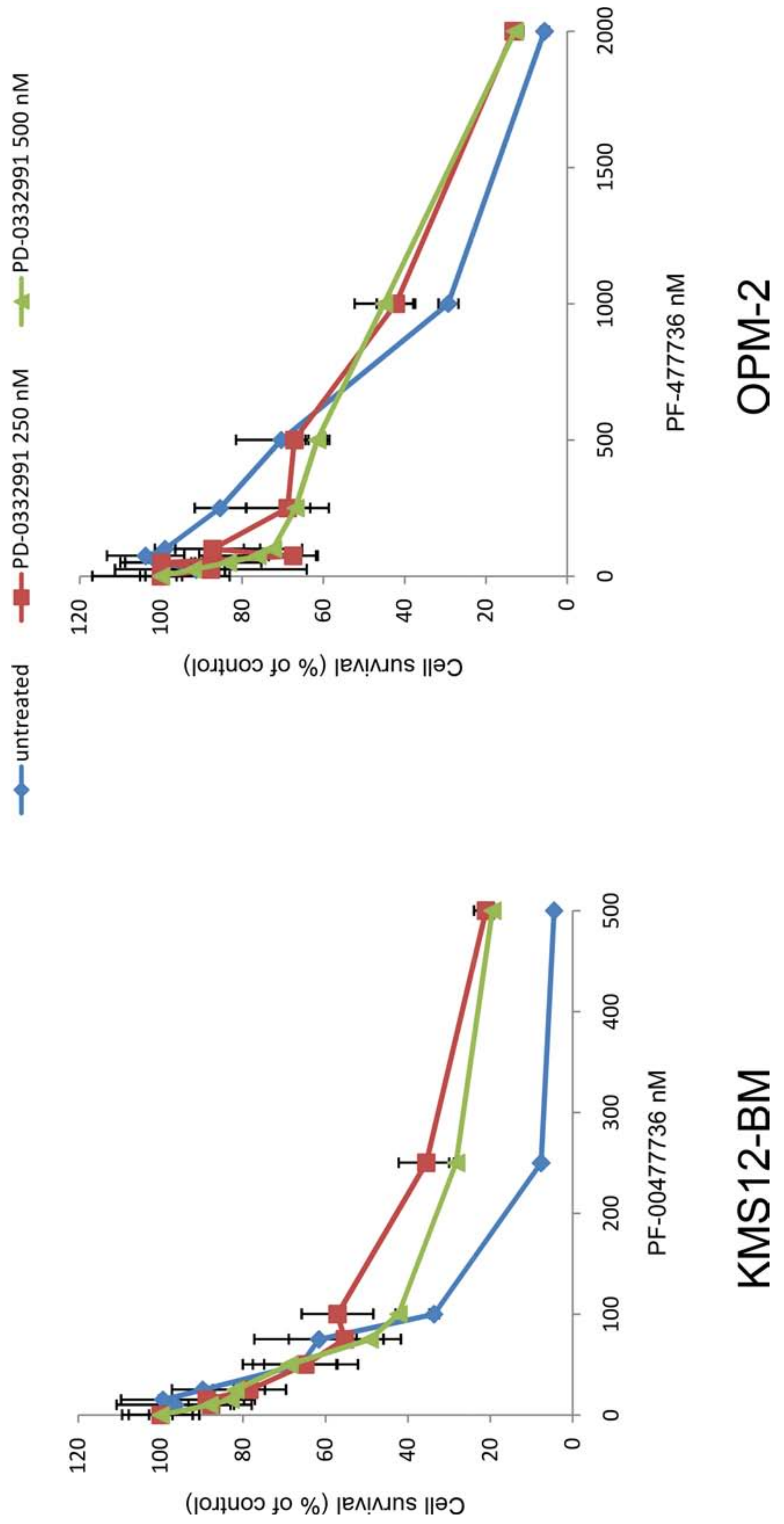

Supplementary Figure S5: Cytotoxic effect of PF-00477736 in KMS12-BM cell line with the t(11;14) (left panel) and in OPM2 cell line with no translocation (right panel) as single agent or with two concentrations of PD-0332991 (see legend in the figure).

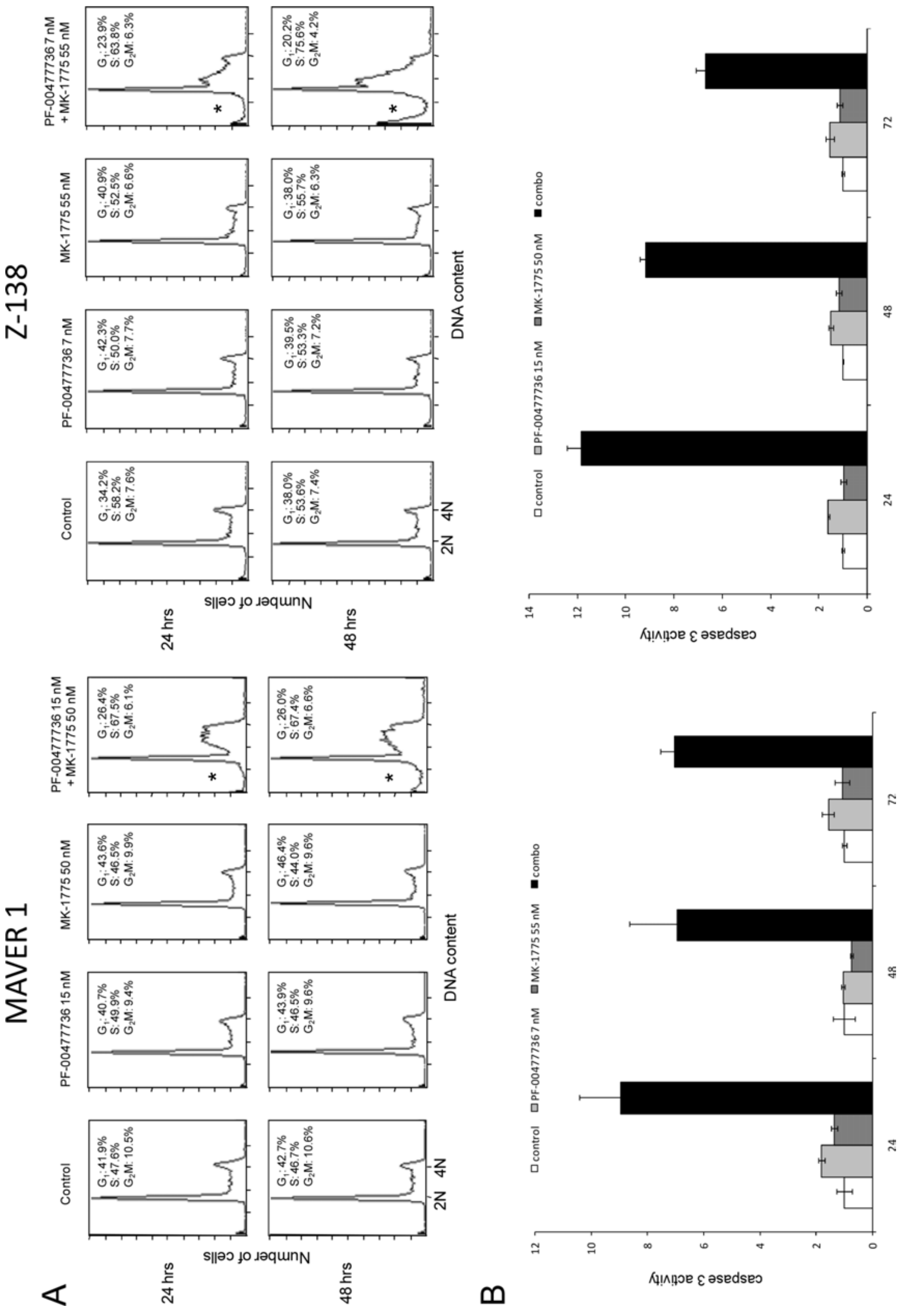

Supplementary Figure S6: (A) Analysis of DNA content by FACS after 24 and 48 hrs of treatment with the two drugs either singly or combined in MAVER-1 (left panel) and in Z-138 cell lines (right panel). Percentages of cell cycle phases (G1-S-G2/M) are included in the figure. The asterisk (*) points the sub-G1 population in the combined samples. **(B)** Activation of caspase-3 by enzymatic assay in these cell lines 24, 48, and 72 hrs after treatment with PF-00477736 and MK-1775 either singly or combined. Data are represented as fold change over untreated cells and are the mean ±SD of two independent experiments.

Supplementary Table S1. Gene-sets belonging to the C2.CGP (Chemical and Genetic Perturbations) Gene Set Enrichment Analysis (GSEA) collection most differentially expressed between sensitive versus resistant PF-00477736 cells. The table shows the 15 gene-sets most significantly enriched among the genes up-regulated and the 15 most significantly enriched among the genes down-regulated in sensitive versus resistant PF-00477736 cells. NAME, name of significant gene set; SIZE, number of members in gene set; ES, enrichment score; NES, normalized enrichment score; NOM p-val, nominal p-value; FDR q-val, false discovery rate q-value. The genes contained in the enrichment core of the top five gene-sets of each comparison are shown.

Comparison	NAME	SIZE	ES	NES	NOM p-val	FDR q-val	Genes in the Enrichment Core
up in sensitive	HUMMEL_ BURKITTS_ LYMPHOMA_UP	39	0.869	2.365	0.000	0.000	D4S234E, ALDH5A1, BACH2, VPREB1, BMP7, LEF1, TCF3, SSBP2, MME, TUBB2B, SOX11, ID3, SMARCA4, FHOD3, NASP, PTBP2, LZTS1, TFDP2, TCL6, UCHL1, TERT, LOC282997, HADH, PRDM10, SLC35E3
up in sensitive	ZHOU_CELL_ CYCLE_GENES_IN_ IR_RESPONSE_24HR	120	0.698	2.282	0.000	0.000	NASP, KNTC1, FBLN1, KIFC1, NCAPH2, NEIL3, UHRF1, HMGB3, DCN, LXN, FBXO5, BUB1, CDKN3, PTTG3P, UBE2T, CDK2, BIRC5, DNAJC9, RBBP8, GINS3, KIF22, KIF11, TRIP13, MAGOHB, CENPN, RFC5, MELK, KIF20A, CENPK, PTTG1, WIPI1, UBE2C, RACGAP1, CCNA2, NEK2, KIF23, EIF2S1, SPC25, MCM2, CCNB2, FAM64A, SMC2, HAUS8, HN1, H2AFV, NUP107, TIMELESS, CENPF, RAD54L, ASPM, COLEC12, ANLN, TOPBP1, MAD2L1, TDP1, PTTG2, MDC1, HMGB2, SMPD4, PCNA, TOP2A, MCM3, MCM8
up in sensitive	PYEON_HPV_ POSITIVE_TUMORS_ UP	82	0.733	2.266	0.000	0.000	CDKN2A, MYB, NASP, BTNL9, SMARCA2, RBBP4, KNTC1, ZSCAN16, TYMS, BARD1, STAG3, CDC7, UHRF1, E2F7, CDCA7, USP34, SLFN13, CDKN2C, ZNF789, PSIP1, TMEM194A, FUBP1, ATAD2, WDR76, LIG1, TM7SF3, MEIS1, DHFR, IL17RB, TIA1, FANCL, RTKN2, CENPK, NEURL1B, KLHL35, TARDBP, PDIK1L, KIF15, DTL, CENPF, NR1D2, OVOS2, CCHCR1, ANKRD32, MAP7D2, MCM3, MCM8, DONSON
up in sensitive	BASSO_CD40_ SIGNALING_DN	65	0.754	2.255	0.000	0.000	ALDH5A1, SLC2A5, CD27, IGJ, NCF4, BMP7, TCF3, TRIB2, GPER, UBE2J1, BIK, ID3, RIMS3, GCNT1, CNR1, CAMP, TUBB3, RAPGEF5, METTL7A, AIF1, SH2B2, PTP4A2, BCL6, KCNN3, RERE, BICD2, VNN2, TTC9, HSD17B8, CD38, ID1, DFNA5, ST6GAL1

(*continued*)

Comparison	NAME	SIZE	ES	NES	NOM p-val	FDR q-val	Genes in the Enrichment Core
up in sensitive	NIKOLSKY_BREAST_CANCER_16Q24_AMPLICON	46	0.778	2.198	0.000	0.000	TUBB3, GINS2, RPL13, KIAA0182, ACSF3, DBNDD1, SPG7, ZCCHC14, GAS8, CENPBD1, CDK10, APRT, BANP, MVD, KLHDC4, TCF25, DEF8, CDT1, MAP1LC3B, SPIRE2, SLC7A5, PRDM7, ZFPM1, ANKRD11, CHMP1A, DPEP1, JPH3, GALNS
up in sensitive	DUTERTRE_ESTRADIOL_RESPONSE_24HR_UP	309	0.589	2.146	0.000	0.000	
up in sensitive	SOTIRIOU_BREAST_CANCER_GRADE_1_VS_3_UP	143	0.623	2.125	0.000	0.000	
up in sensitive	PUJANA_BRCA2_PCC_NETWORK	407	0.563	2.119	0.000	0.000	
up in sensitive	WU_APOPTOSIS_BY_CDKN1A_VIA_TP53	52	0.731	2.110	0.000	0.000	
up in sensitive	KONG_E2F3_TARGETS	96	0.665	2.109	0.000	0.000	
up in sensitive	ROSTY_CERVICAL_CANCER_PROLIFERATION_CLUSTER	137	0.629	2.080	0.000	0.000	
up in sensitive	ZHOU_CELL_CYCLE_GENES_IN_IR_RESPONSE_6HR	80	0.672	2.074	0.000	0.000	
up in sensitive	ISHIDA_E2F_TARGETS	52	0.715	2.041	0.000	0.000	
up in sensitive	RICKMAN_HEAD_AND_NECK_CANCER_D	29	0.785	2.040	0.000	0.000	
up in sensitive	PUJANA_XPRSS_INT_NETWORK	160	0.598	2.036	0.000	0.000	
up in resistant	KUROZUMI_RESPONSE_TO_ONCOCYTIC_VIRUS	42	-0.886	-2.355	0.000	0.000	ITGAM, IL15, IL1B, LTB, IL16, IL1R2, CCL5, IL1A, LTA, CASP1, CXCL9, IL2RB, SPP1, CCL3, IL10RA, CCL22, CXCR5, TNF, CCR7, TNFRSF1B, CXCL10
up in resistant	ALTEMEIER_RESPONSE_TO_LPS_WITH_MECHANICAL_VENTILATION	126	-0.738	-2.301	0.000	0.000	CCRL2, RCAN1, ADAM19, ITGAM, CEBPD, TNFSF9, GEM, IL15, GPR65, JUNB, OAS2, IL1B, ST3GAL1, GDA, TNFAIP3, SOCS3, MT1A, GK, OAS3, IL1R2, IFIT1, CYBB, LITAF, IL4I1, BCL3, CASP4, IRF7, NFKBIA, TIMP1, MT2A, MAP3K8, IL1A, BCL2A1, SLA, SOD2, UPP1, CCL3, FGL2, GBP1, IL6, SLC15A3, SELL, ATF3, PLEK, NFKBIZ, EBI3, CXCL10

Comparison	NAME	SIZE	ES	NES	NOM p-val	FDR q-val	Genes in the Enrichment Core
up in resistant	SEKI_ INFLAMMATORY_ RESPONSE_LPS_UP	74	-0.764	-2.246	0.000	0.000	TNFAIP2, JDP2, GBP4, STX11, CCRL2, RND1, ICAM1, TNIP1, TMEM2, RIPK2, SLC11A2, BIRC3, TNFAIP3, PION, PLSCR1, CASP4, NFKBIA, SOD2, ZC3H12A, NFKBIE, GBP2, CCL3, GBP1, CD40, SLC15A3, NFKBIZ, CD44, CXCL10
up in resistant	LINDSTEDT_ DENDRITIC_CELL_ MATURATION_A	66	-0.769	-2.206	0.000	0.000	SLC3A2, ISG15, TXNRD1, CXCL11, CCL20, IL12B, ICAM1, STAT4, TNFSF9, CD58, PLA2G4A, IL1B, RAB5A, PSMA3, CCL5, GPR137B, IL7, IL8, SOD2, NFKBIE, KYNU, PTGER4, CD48, CCL3, GBP1, IL6, CD40, TNF, ENTPD1, PLEK, CD44, CXCL10
up in resistant	DIRMEIER_LMP1_ RESPONSE_LATE_UP	55	-0.788	-2.205	0.000	0.000	TNIP1, CD58, NFKB1, SWAP70, VOPP1, TRIP10, RUNX3, LYN, LITAF, SPIB, SQSTM1, STAT5A, IRF5, MDFIC, RHOG, CFLAR, HMOX1, CD40, CCL22, MARCKS, FEZ1, PLEK, TNFRSF1B, CD44, BATF
up in resistant	GHANDHI_ BYSTANDER_ IRRADIATION_UP	82	-0.712	-2.094	0.000	0.000	
up in resistant	DIRMEIER_LMP1_ RESPONSE_EARLY	66	-0.732	-2.079	0.000	0.000	
up in resistant	DEBOSSCHER_ NFKB_TARGETS_ REPRESSED_BY_ GLUCOCORTICOIDS	24	-0.854	-2.054	0.000	0.000	
up in resistant	EINAV_ INTERFERON_ SIGNATURE_IN_ CANCER	27	-0.846	-2.052	0.000	0.000	
up in resistant	TAKEDA_TARGETS_ OF_NUP98_HOXA9_ FUSION_3D_UP	171	-0.624	-2.029	0.000	0.000	
up in resistant	BOWIE_RESPONSE_ TO_TAMOXIFEN	18	-0.905	-2.004	0.000	0.001	
up in resistant	GALINDO_IMMUNE_ RESPONSE_TO_ ENTEROTOXIN	83	-0.664	-2.000	0.000	0.001	
up in resistant	MAHADEVAN_ RESPONSE_TO_ MP470_UP	20	-0.849	-1.996	0.000	0.001	

(*continued*)

Comparison	NAME	SIZE	ES	NES	NOM p-val	FDR q-val	Genes in the Enrichment Core
up in resistant	HUMMEL_ BURKITTS_ LYMPHOMA_DN	15	-0.917	-1.985	0.000	0.001	
up in resistant	VALK_AML_ CLUSTER_1	25	-0.836	-1.979	0.000	0.001	

Supplementary Table S2. List of transcripts differentially expressed between control xenografts and at least one of the treatment groups (PF-00477736, MK-1775, Combo I, Combo II). Transcripts are sorted based the comparison between control xenograft versus Combo I. t-stat, t-statistics value; mean, average expression value.

Supplementary Table S3. Gene-sets belonging to the C2. CGP GSEA collection most differentially expressed between control xenografts and xenografts treated with MK-1775 and PF-00477736 alone or combined (Combo I and Combo II). The table shows the 15 gene-sets most significantly enriched among the genes up-regulated and the 15 most significantly enriched among the genes down-regulated for each comparison. NAME, name of significant gene set; SIZE, number of members in gene set; ES, enrichment score; NES, normalized enrichment score; NOM p-val, nominal p-value; FDR q-val, false discovery rate q-value. The genes contained in the enrichment core of the top five gene-sets of each comparison are shown.

Supplementary Table S4. List of primers used for real-time PCR on cDNA

	Primer FOR	Primer REV
ß-actin	TACAATGAGCTGCGTGTGG	GGGGTGTTGAAGGTCTCAAA
Gadd45b	AGTCGGCCAAGTTGATGAAT	GGATGAGCGTGAAGTGGATT
c-Jun	CAGGTGGCACAGCTTAAACA	GTTTGCAACTGCTGCGTTAG
Tnfaip3	TGGGACTCCAGAAAACAAGG	GTGGGACTGACTTTCCCTGA
Nfkbia	GCTGATGTCAATGCTCAGGA	ACACCAGGTCAGGATTTTGC
