# Supplementary material for: Combined inhibition of Chk1 and Wee1 as a new therapeutic strategy for mantle cell lymphoma
Source: Oncotarget. 2014 Oct 25;6(5):3394–408. doi: 10.18632/oncotarget.2583 (PMC4413661; doi:10.18632/oncotarget.2583)
Supplement: Supplementary file 2 [file oncotarget-06-3394-s002.doc]

| Probe_ID | Symbol | t-stat, Ctrl vs Combo1 | t-stat,Ctrl vs PF | t-stat, Ctrl vs MK | t-stat, Ctrl vs Combo2 | Ctrl mean | Combo1 mean | PF mean | MK mean | Combo2 mean |
| --- | --- | --- | --- | --- | --- | --- | --- | --- | --- | --- |
| ILMN_1695658 | KIF20A | -12.54 | -1.25 | -5.67 | -5.16 | 9.47 | 10.93 | 9.61 | 10.13 | 10.07 |
| ILMN_2315964 | PSRC1 | -11.74 | -1.43 | -9.22 | -8.02 | 7.25 | 8.29 | 7.38 | 8.07 | 7.96 |
| ILMN_1773154 | NFKBIA | -11.31 | -3.00 | -1.97 | -2.97 | 10.25 | 11.61 | 10.61 | 10.49 | 10.61 |
| ILMN_1806023 | JUN | -10.84 | -0.68 | -0.49 | -0.32 | 6.96 | 8.44 | 7.05 | 7.03 | 7.00 |
| ILMN_1704284 | LOC648164 | -10.66 | -1.54 | -2.86 | -3.68 | 7.03 | 8.32 | 7.22 | 7.38 | 7.48 |
| ILMN_2349459 | BIRC5 | -10.62 | -1.85 | -8.25 | -3.77 | 9.96 | 10.99 | 10.14 | 10.76 | 10.33 |
| ILMN_1671843 | PSRC1 | -10.12 | -1.45 | -7.13 | -8.05 | 7.61 | 8.96 | 7.80 | 8.56 | 8.69 |
| ILMN_1718977 | GADD45B | -9.53 | 0.66 | 0.36 | -0.60 | 7.46 | 8.95 | 7.36 | 7.40 | 7.55 |
| ILMN_1709294 | CDCA8 | -9.38 | -2.36 | -5.28 | -8.12 | 9.77 | 10.54 | 9.96 | 10.20 | 10.43 |
| ILMN_1702691 | TNFAIP3 | -9.32 | 0.68 | 1.26 | -1.69 | 7.31 | 8.32 | 7.24 | 7.18 | 7.50 |
| ILMN_2357438 | AURKA | -9.26 | 0.51 | -4.01 | -5.57 | 10.26 | 11.53 | 10.19 | 10.81 | 11.03 |
| ILMN_2301083 | UBE2C | -9.19 | -2.67 | -8.11 | -7.31 | 11.68 | 12.68 | 11.97 | 12.56 | 12.48 |
| ILMN_1685916 | KIF2C | -9.14 | -3.09 | -7.62 | -7.12 | 9.05 | 9.79 | 9.30 | 9.67 | 9.63 |
| ILMN_1658121 | CFP | -9.08 | -1.09 | -1.04 | -3.01 | 7.10 | 8.31 | 7.24 | 7.24 | 7.50 |
| ILMN_1703906 | HJURP | -9.05 | 0.00 | 0.58 | -6.99 | 9.48 | 10.26 | 9.48 | 9.43 | 10.08 |
| ILMN_1680955 | AURKA | -8.96 | -0.02 | -4.29 | -6.14 | 10.40 | 11.63 | 10.41 | 10.99 | 11.24 |
| ILMN_1735579 | CGB8 | -8.79 | -1.94 | -1.62 | -2.38 | 6.81 | 7.82 | 7.03 | 7.00 | 7.08 |
| ILMN_2354547 | TUSC3 | -8.72 | -1.40 | -1.95 | -0.97 | 6.79 | 7.47 | 6.90 | 6.94 | 6.86 |
| ILMN_1727753 | CR2 | -8.62 | -1.12 | -0.49 | -0.67 | 6.65 | 7.30 | 6.74 | 6.69 | 6.70 |
| ILMN_1751776 | CKAP2L | -8.54 | -1.99 | -2.91 | -4.82 | 9.14 | 10.15 | 9.37 | 9.48 | 9.71 |
| ILMN_1691647 | CGB5 | -8.51 | -1.46 | -1.27 | -1.75 | 6.78 | 7.67 | 6.93 | 6.91 | 6.96 |
| ILMN_1714730 | UBE2C | -8.41 | -1.25 | -7.48 | -5.19 | 11.69 | 12.66 | 11.83 | 12.55 | 12.29 |
| ILMN_1801257 | CENPA | -8.39 | -1.78 | -6.14 | -4.27 | 9.35 | 10.91 | 9.68 | 10.49 | 10.14 |
| ILMN_1663390 | CDC20 | -8.35 | -1.27 | -7.11 | -4.25 | 10.94 | 12.03 | 11.11 | 11.87 | 11.50 |
| ILMN_2077550 | RACGAP1 | -8.32 | -1.21 | -5.92 | -6.11 | 9.52 | 10.55 | 9.67 | 10.25 | 10.28 |
| ILMN_1682717 | IER3 | -8.23 | -0.99 | -2.80 | -4.33 | 7.25 | 8.33 | 7.38 | 7.62 | 7.82 |
| ILMN_1671933 | CLCC1 | -7.88 | -0.64 | -6.90 | -6.90 | 8.48 | 9.50 | 8.57 | 9.38 | 9.38 |
| ILMN_1660654 | CDCA2 | -7.85 | -0.62 | -3.21 | -5.95 | 7.69 | 8.57 | 7.76 | 8.05 | 8.36 |
| ILMN_2392472 | CENPA | -7.77 | -2.03 | -5.58 | -4.35 | 7.76 | 9.17 | 8.13 | 8.77 | 8.55 |
| ILMN_1811472 | KIF23 | -7.69 | -0.72 | -3.95 | -3.03 | 8.55 | 9.69 | 8.66 | 9.14 | 9.00 |
| ILMN_2222008 | KIFC1 | -7.61 | -1.19 | -7.08 | -5.41 | 9.51 | 10.66 | 9.69 | 10.58 | 10.33 |
| ILMN_1773119 | CCNF | -7.59 | -0.62 | -4.72 | -3.32 | 10.42 | 11.31 | 10.50 | 10.98 | 10.81 |
| ILMN_1761486 | C13orf34 | -7.58 | -0.09 | -3.76 | -4.11 | 9.72 | 10.59 | 9.73 | 10.15 | 10.19 |
| ILMN_1717403 | C9orf100 | -7.55 | -0.21 | -3.88 | -0.21 | 7.02 | 7.61 | 7.03 | 7.32 | 7.03 |
| ILMN_1674662 | C15orf42 | -7.54 | -1.21 | -3.94 | -1.78 | 7.95 | 8.61 | 8.05 | 8.29 | 8.10 |
| ILMN_2355738 | INCENP | -7.53 | -0.71 | -5.23 | -4.07 | 8.72 | 9.50 | 8.79 | 9.26 | 9.14 |
| ILMN_1749078 | TIMP2 | -7.50 | -1.13 | -1.65 | -1.65 | 7.02 | 7.84 | 7.14 | 7.20 | 7.20 |
| ILMN_1721127 | HIST1H3D | -7.47 | -0.55 | -1.06 | -3.01 | 7.34 | 8.47 | 7.42 | 7.50 | 7.79 |
| ILMN_1740429 | FTL | -7.39 | -1.19 | -1.08 | -2.74 | 11.87 | 12.60 | 11.99 | 11.98 | 12.14 |
| ILMN_2115340 | HIST2H4A | -7.34 | -2.25 | -3.06 | -3.72 | 7.80 | 9.70 | 8.38 | 8.59 | 8.76 |
| ILMN_1814823 | FTL | -7.34 | -1.71 | -1.71 | -3.23 | 11.78 | 12.39 | 11.92 | 11.92 | 12.05 |
| ILMN_1681221 | C9orf100 | -7.34 | 0.00 | -5.88 | -3.89 | 7.38 | 7.98 | 7.38 | 7.86 | 7.70 |
| ILMN_1808591 | LOC731049 | -7.19 | -2.04 | -7.36 | -4.86 | 11.03 | 11.76 | 11.24 | 11.78 | 11.53 |
| ILMN_1744830 | ARHGAP11A | -7.14 | -1.94 | -2.49 | -3.93 | 7.20 | 7.77 | 7.36 | 7.40 | 7.51 |
| ILMN_1811767 | INHBE | -7.13 | -2.58 | -7.38 | -4.46 | 6.87 | 8.59 | 7.49 | 8.65 | 7.95 |
| ILMN_3292244 | LOC100132804 | -7.11 | -0.35 | -0.53 | -0.79 | 8.17 | 8.98 | 8.21 | 8.23 | 8.26 |
| ILMN_1808071 | KIF14 | -7.05 | 0.19 | -5.72 | -3.44 | 9.85 | 10.58 | 9.83 | 10.44 | 10.21 |
| ILMN_1728106 | TNF | -7.01 | -0.28 | 0.07 | -1.74 | 6.64 | 7.30 | 6.67 | 6.64 | 6.81 |
| ILMN_1662198 | RANGAP1 | -7.01 | -1.14 | -6.00 | -2.11 | 10.52 | 11.24 | 10.63 | 11.13 | 10.73 |
| ILMN_2181060 | CKAP2 | -6.95 | -0.92 | -3.56 | -1.75 | 10.20 | 11.03 | 10.31 | 10.62 | 10.41 |
| ILMN_3238233 | HIST2H4B | -6.93 | -2.21 | -2.68 | -2.63 | 7.60 | 9.01 | 8.05 | 8.15 | 8.14 |
| ILMN_1791728 | SLC25A25 | -6.89 | -0.54 | -1.89 | -2.54 | 8.24 | 8.88 | 8.29 | 8.42 | 8.48 |
| ILMN_1654118 | BCL2L1 | -6.87 | -5.17 | -4.42 | -6.56 | 8.93 | 9.67 | 9.48 | 9.40 | 9.63 |
| ILMN_1797307 | BUB1B | -6.84 | -0.87 | -5.82 | -3.07 | 8.84 | 9.73 | 8.95 | 9.59 | 9.24 |
| ILMN_1792473 | AIF1 | -6.83 | -1.70 | -1.55 | -4.28 | 8.87 | 10.08 | 9.17 | 9.15 | 9.63 |
| ILMN_1695949 | SIGLEC12 | -6.82 | -0.57 | -2.25 | -0.39 | 7.07 | 7.71 | 7.13 | 7.28 | 7.11 |
| ILMN_2100209 | CCL4L1 | -6.79 | -0.74 | -1.55 | -6.93 | 6.91 | 8.01 | 7.03 | 7.16 | 8.03 |
| ILMN_1775498 | FAM100B | -6.67 | -1.50 | -3.07 | -2.22 | 7.94 | 8.65 | 8.10 | 8.27 | 8.18 |
| ILMN_1739641 | MTMR3 | -6.64 | -3.46 | -3.18 | 0.00 | 8.41 | 9.13 | 8.78 | 8.75 | 8.41 |
| ILMN_2202948 | BUB1 | -6.64 | -0.14 | -4.80 | -5.97 | 9.74 | 10.54 | 9.76 | 10.32 | 10.46 |
| ILMN_1651316 | CD69 | -6.60 | -1.46 | -2.69 | -3.42 | 7.48 | 8.91 | 7.80 | 8.06 | 8.22 |
| ILMN_1656057 | PLAU | -6.56 | -0.70 | -1.99 | -0.95 | 7.33 | 8.23 | 7.43 | 7.60 | 7.46 |
| ILMN_2374865 | ATF3 | -6.52 | -1.88 | -4.06 | -2.95 | 6.80 | 8.00 | 7.14 | 7.54 | 7.34 |
| ILMN_2188333 | CD69 | -6.50 | -1.55 | -2.54 | -4.01 | 8.47 | 10.44 | 8.94 | 9.24 | 9.69 |
| ILMN_2132161 | KIF18A | -6.46 | 0.69 | -2.75 | -2.00 | 8.49 | 9.18 | 8.42 | 8.79 | 8.71 |
| ILMN_1674411 | CKAP2 | -6.45 | -1.28 | -3.80 | -2.73 | 9.07 | 10.01 | 9.25 | 9.62 | 9.47 |
| ILMN_2323944 | FAM110A | -6.39 | -2.53 | -4.41 | -5.98 | 7.65 | 8.54 | 8.00 | 8.26 | 8.48 |
| ILMN_1739241 | CHAC1 | -6.36 | -1.71 | -5.30 | -1.40 | 6.60 | 7.34 | 6.80 | 7.22 | 6.76 |
| ILMN_1810891 | ZNF629 | -6.36 | -4.45 | -5.10 | -4.92 | 7.20 | 7.79 | 7.61 | 7.67 | 7.65 |
| ILMN_3251341 | TUBA1C | -6.31 | -1.09 | -5.19 | -5.03 | 12.09 | 12.88 | 12.23 | 12.74 | 12.72 |
| ILMN_1747016 | CEP55 | -6.31 | 1.19 | -4.76 | -0.40 | 10.18 | 10.77 | 10.07 | 10.62 | 10.22 |
| ILMN_1739645 | ANLN | -6.30 | -1.44 | -5.52 | -3.10 | 8.67 | 9.59 | 8.88 | 9.47 | 9.12 |
| ILMN_1728934 | PRC1 | -6.29 | 0.72 | -4.25 | -2.09 | 10.90 | 11.74 | 10.80 | 11.47 | 11.18 |
| ILMN_1702140 | RACGAP1 | -6.29 | -2.12 | -4.44 | -4.63 | 7.56 | 8.33 | 7.82 | 8.10 | 8.12 |
| ILMN_1762787 | RNF26 | -6.27 | -2.05 | -3.59 | -6.97 | 7.58 | 8.25 | 7.80 | 7.96 | 8.32 |
| ILMN_1651496 | HIST1H2BD | -6.27 | -1.54 | -3.44 | -4.04 | 10.02 | 11.00 | 10.26 | 10.56 | 10.65 |
| ILMN_2148847 | AKIRIN2 | -6.27 | -1.51 | -3.78 | -2.56 | 10.16 | 10.74 | 10.30 | 10.51 | 10.39 |
| ILMN_1781943 | FAM83D | -6.23 | -0.89 | -2.20 | -3.80 | 9.03 | 10.03 | 9.17 | 9.38 | 9.64 |
| ILMN_1742145 | ESPL1 | -6.23 | -3.43 | -4.69 | -7.49 | 7.77 | 8.43 | 8.13 | 8.27 | 8.56 |
| ILMN_1724407 | TACC3 | -6.23 | -1.45 | -5.61 | -0.32 | 9.24 | 9.89 | 9.39 | 9.82 | 9.28 |
| ILMN_3267670 | LOC100130550 | -6.21 | 0.49 | -3.52 | -2.89 | 7.93 | 8.69 | 7.87 | 8.36 | 8.28 |
| ILMN_1796949 | TPX2 | -6.20 | -1.45 | -5.19 | -4.00 | 9.62 | 10.69 | 9.87 | 10.52 | 10.31 |
| ILMN_1664516 | CENPF | -6.17 | -1.83 | -5.29 | -8.07 | 8.90 | 9.47 | 9.07 | 9.39 | 9.64 |
| ILMN_1720373 | SLC7A5 | -6.17 | -3.31 | -7.93 | 2.28 | 10.75 | 11.43 | 11.12 | 11.62 | 10.50 |
| ILMN_1702487 | SGK | -6.13 | -1.98 | -1.21 | -0.69 | 7.89 | 8.93 | 8.22 | 8.09 | 8.00 |
| ILMN_2413650 | STIL | -6.01 | -0.49 | -2.36 | -1.83 | 9.30 | 9.79 | 9.34 | 9.49 | 9.45 |
| ILMN_1748770 | CKAP5 | -5.99 | -2.54 | -5.21 | -2.96 | 11.11 | 11.67 | 11.35 | 11.60 | 11.39 |
| ILMN_3309514 | MIR574 | -5.96 | -0.41 | -0.94 | -1.55 | 7.38 | 9.51 | 7.52 | 7.71 | 7.93 |
| ILMN_1676984 | DDIT3 | -5.95 | -0.76 | -1.95 | -4.32 | 8.16 | 9.66 | 8.35 | 8.65 | 9.25 |
| ILMN_1747078 | HYLS1 | -5.93 | -0.44 | -1.90 | -2.27 | 7.81 | 8.66 | 7.87 | 8.08 | 8.13 |
| ILMN_1782095 | C20orf55 | -5.90 | -1.22 | -3.23 | -4.60 | 7.87 | 8.53 | 8.00 | 8.23 | 8.38 |
| ILMN_1740938 | APOE | -5.89 | 0.64 | -1.41 | -3.63 | 9.09 | 10.82 | 8.90 | 9.50 | 10.15 |
| ILMN_1760563 | BAT2 | -5.89 | -4.91 | -6.82 | -4.10 | 8.45 | 8.89 | 8.81 | 8.96 | 8.75 |
| ILMN_1669718 | PSENEN | -5.87 | -0.47 | -1.15 | -1.15 | 7.73 | 8.47 | 7.79 | 7.87 | 7.87 |
| ILMN_3239771 | DLGAP5 | -5.82 | 0.99 | -3.73 | -3.19 | 8.91 | 9.95 | 8.74 | 9.58 | 9.48 |
| ILMN_1736176 | PLK1 | -5.81 | 0.47 | -4.54 | -2.10 | 8.50 | 9.49 | 8.42 | 9.28 | 8.86 |
| ILMN_3248164 | LOC100132096 | -5.80 | -2.63 | -1.95 | -3.81 | 6.80 | 7.23 | 6.99 | 6.94 | 7.08 |
| ILMN_2139816 | GPSM2 | -5.78 | -0.33 | -3.97 | -4.36 | 8.06 | 8.99 | 8.12 | 8.70 | 8.76 |
| ILMN_2086077 | JUNB | -5.75 | -1.46 | -2.18 | -2.66 | 6.99 | 7.55 | 7.13 | 7.20 | 7.25 |
| ILMN_1677768 | POR | -5.75 | -1.80 | -1.49 | -1.71 | 7.57 | 8.17 | 7.76 | 7.72 | 7.75 |
| ILMN_2051373 | NEK2 | -5.73 | 1.14 | -3.73 | 0.17 | 11.09 | 11.65 | 10.98 | 11.45 | 11.08 |
| ILMN_2215881 | ARHGAP11B | -5.73 | -2.00 | -3.33 | -1.85 | 7.21 | 7.72 | 7.39 | 7.51 | 7.37 |
| ILMN_1788166 | TTK | -5.72 | -0.52 | -3.03 | -2.85 | 9.09 | 9.94 | 9.16 | 9.54 | 9.51 |
| ILMN_1757467 | H1F0 | -5.72 | -4.20 | -4.78 | -4.89 | 9.76 | 11.06 | 10.71 | 10.84 | 10.87 |
| ILMN_2407619 | CDC25C | -5.70 | 0.48 | -2.01 | -1.83 | 7.55 | 8.18 | 7.50 | 7.77 | 7.75 |
| ILMN_1720270 | CDR2 | -5.68 | 1.47 | -0.85 | 2.40 | 9.29 | 9.78 | 9.16 | 9.36 | 9.08 |
| ILMN_1718972 | MFSD3 | -5.67 | -3.84 | -3.62 | -4.39 | 8.81 | 9.40 | 9.21 | 9.19 | 9.27 |
| ILMN_1658607 | DLEU2 | -5.65 | -1.28 | -2.01 | -3.49 | 9.57 | 10.53 | 9.79 | 9.91 | 10.16 |
| ILMN_1750961 | TM6SF1 | -5.65 | -3.22 | -6.11 | -0.30 | 7.82 | 8.52 | 8.22 | 8.58 | 7.86 |
| ILMN_1659936 | PPP1R15A | -5.65 | 0.06 | -1.40 | 0.45 | 7.94 | 9.15 | 7.93 | 8.24 | 7.84 |
| ILMN_1749829 | DLGAP5 | -5.64 | 0.71 | -4.07 | -3.48 | 9.39 | 10.42 | 9.26 | 10.13 | 10.02 |
| ILMN_1673282 | LAMP2 | -5.62 | -0.52 | -1.21 | 0.52 | 7.92 | 8.61 | 7.99 | 8.07 | 7.86 |
| ILMN_1737406 | KLF6 | -5.55 | -0.79 | -2.73 | 0.29 | 8.12 | 9.06 | 8.25 | 8.58 | 8.07 |
| ILMN_2368530 | IL32 | -5.55 | -1.55 | -2.37 | -8.59 | 6.89 | 7.64 | 7.10 | 7.21 | 8.05 |
| ILMN_1802706 | IDH3G | -5.51 | -1.91 | -3.21 | -3.81 | 8.30 | 8.92 | 8.51 | 8.66 | 8.73 |
| ILMN_1723843 | CSNK2A2 | -5.47 | -1.66 | -2.87 | 0.94 | 8.68 | 9.16 | 8.82 | 8.93 | 8.59 |
| ILMN_1787815 | TRIB3 | -5.41 | -3.43 | -4.70 | -6.04 | 8.52 | 9.43 | 9.10 | 9.31 | 9.54 |
| ILMN_2347798 | IFI6 | -5.41 | -2.47 | -3.72 | -2.49 | 7.28 | 8.50 | 7.84 | 8.12 | 7.84 |
| ILMN_1719695 | NFKBIZ | -5.37 | -1.13 | 3.11 | 1.46 | 8.68 | 9.23 | 8.80 | 8.36 | 8.53 |
| ILMN_1782611 | LOC643870 | -5.37 | -0.92 | -0.89 | 0.13 | 9.18 | 9.87 | 9.30 | 9.30 | 9.17 |
| ILMN_2099301 | UNC84B | -5.35 | -1.31 | -6.93 | -4.46 | 10.41 | 11.09 | 10.57 | 11.29 | 10.97 |
| ILMN_1789733 | CLIP3 | -5.35 | 0.05 | -0.92 | 1.11 | 7.16 | 7.53 | 7.16 | 7.22 | 7.08 |
| ILMN_3236704 | LOC100133420 | -5.35 | -2.83 | -1.77 | -5.86 | 6.82 | 7.17 | 7.00 | 6.93 | 7.20 |
| ILMN_1781942 | HMMR | -5.35 | 1.25 | -1.49 | -0.47 | 9.96 | 10.76 | 9.78 | 10.19 | 10.03 |
| ILMN_1749368 | HIST1H3H | -5.27 | -1.30 | -1.90 | -2.66 | 7.15 | 7.87 | 7.33 | 7.41 | 7.51 |
| ILMN_1725260 | CDC25C | -5.26 | 0.86 | -3.52 | -1.60 | 7.93 | 8.53 | 7.84 | 8.33 | 8.11 |
| ILMN_3221807 | LOC729313 | -5.23 | 0.99 | 0.30 | 1.51 | 7.42 | 7.82 | 7.34 | 7.40 | 7.30 |
| ILMN_1697448 | TXNIP | -5.23 | -0.86 | -2.41 | -4.07 | 10.30 | 11.11 | 10.44 | 10.68 | 10.93 |
| ILMN_1737728 | CDCA3 | -5.21 | 1.00 | -4.08 | -2.50 | 9.94 | 10.74 | 9.78 | 10.56 | 10.32 |
| ILMN_1747911 | CDC2 | -5.20 | 0.86 | -3.00 | -1.81 | 10.39 | 11.29 | 10.24 | 10.91 | 10.70 |
| ILMN_3179891 | LOC100128007 | -5.18 | 0.86 | -3.66 | -1.40 | 7.43 | 7.95 | 7.35 | 7.80 | 7.57 |
| ILMN_1709882 | ICK | -5.18 | -2.06 | -4.43 | -2.91 | 7.93 | 8.57 | 8.18 | 8.48 | 8.29 |
| ILMN_1664761 | TMEM138 | -5.17 | -0.38 | -0.83 | -4.38 | 8.22 | 8.68 | 8.25 | 8.29 | 8.61 |
| ILMN_1684217 | AURKB | -5.17 | 0.99 | -0.93 | -0.82 | 8.76 | 9.39 | 8.64 | 8.87 | 8.86 |
| ILMN_1708934 | ADM | -5.15 | -1.25 | -1.18 | -2.68 | 7.09 | 7.58 | 7.21 | 7.20 | 7.35 |
| ILMN_1753524 | HIST1H2AB | -5.15 | -1.78 | -1.23 | -0.55 | 7.00 | 7.53 | 7.18 | 7.12 | 7.05 |
| ILMN_1687384 | IFI6 | -5.14 | -2.08 | -3.76 | -2.63 | 10.23 | 11.35 | 10.68 | 11.05 | 10.80 |
| ILMN_3235832 | LOC728835 | -5.12 | -1.28 | -1.05 | -5.92 | 6.84 | 7.84 | 7.09 | 7.04 | 8.00 |
| ILMN_1678300 | MGC40489 | -5.10 | 0.60 | -2.21 | -2.98 | 9.10 | 10.03 | 8.99 | 9.51 | 9.65 |
| ILMN_1726815 | HIST1H3G | -5.10 | -2.36 | -2.52 | -1.32 | 7.72 | 8.51 | 8.09 | 8.11 | 7.93 |
| ILMN_1788489 | HIST1H3F | -5.09 | -1.85 | -2.31 | -1.75 | 8.28 | 8.97 | 8.53 | 8.59 | 8.52 |
| ILMN_1710428 | CDC2 | -5.05 | 0.35 | -3.03 | -0.99 | 8.61 | 9.48 | 8.55 | 9.13 | 8.78 |
| ILMN_1786125 | CCNA2 | -5.04 | 1.17 | -2.65 | 3.66 | 10.23 | 10.87 | 10.09 | 10.57 | 9.77 |
| ILMN_1771224 | SKA1 | -5.01 | -1.04 | -3.16 | -4.89 | 8.18 | 8.74 | 8.29 | 8.53 | 8.72 |
| ILMN_1716766 | CEBPG | -5.00 | -1.63 | -3.90 | -1.50 | 8.91 | 9.41 | 9.08 | 9.30 | 9.06 |
| ILMN_1794539 | KIF11 | -4.98 | -0.52 | -3.20 | -0.08 | 9.92 | 10.52 | 9.98 | 10.31 | 9.93 |
| ILMN_1693014 | CEBPB | -4.97 | -2.33 | -3.54 | -1.27 | 9.94 | 10.79 | 10.34 | 10.55 | 10.16 |
| ILMN_1780769 | TUBB2C | -4.92 | -1.53 | -4.27 | -2.32 | 10.05 | 10.84 | 10.30 | 10.73 | 10.42 |
| ILMN_1686097 | TOP2A | -4.92 | 0.84 | -2.58 | -1.89 | 11.28 | 11.97 | 11.16 | 11.64 | 11.54 |
| ILMN_1778561 | WEE1 | -4.87 | -3.09 | -2.84 | -2.82 | 9.82 | 10.66 | 10.35 | 10.31 | 10.31 |
| ILMN_1798459 | PPAN | -4.85 | -3.71 | -2.98 | -8.16 | 7.74 | 8.14 | 8.04 | 7.98 | 8.41 |
| ILMN_1791702 | SMARCA2 | -4.84 | -2.39 | -3.63 | -0.69 | 9.10 | 9.68 | 9.39 | 9.54 | 9.18 |
| ILMN_1693340 | RAC3 | -4.84 | -0.86 | -0.82 | -4.97 | 6.93 | 7.40 | 7.01 | 7.01 | 7.41 |
| ILMN_1751120 | HIST1H4H | -4.83 | -1.53 | -2.13 | -3.07 | 8.29 | 9.35 | 8.62 | 8.75 | 8.96 |
| ILMN_1696316 | CPT1A | -4.83 | -0.14 | -0.19 | -4.40 | 7.26 | 7.60 | 7.27 | 7.27 | 7.57 |
| ILMN_2225718 | CENPE | -4.82 | -0.16 | -3.34 | -1.03 | 8.79 | 9.72 | 8.82 | 9.43 | 8.99 |
| ILMN_1779373 | HIST1H2BF | -4.82 | -1.57 | -4.60 | -2.34 | 7.18 | 7.62 | 7.33 | 7.60 | 7.40 |
| ILMN_1755910 | LOC648366 | -4.79 | -0.48 | -3.54 | -2.44 | 7.02 | 7.35 | 7.05 | 7.26 | 7.19 |
| ILMN_1709334 | TM9SF1 | -4.78 | -2.51 | -2.87 | -5.82 | 7.44 | 7.84 | 7.65 | 7.68 | 7.92 |
| ILMN_1756326 | CKS2 | -4.78 | -0.10 | -3.64 | -3.14 | 11.16 | 11.96 | 11.18 | 11.77 | 11.68 |
| ILMN_1716279 | CENPE | -4.76 | 1.42 | -3.38 | -1.07 | 9.62 | 10.43 | 9.38 | 10.19 | 9.80 |
| ILMN_1786015 | CTCF | -4.76 | -3.14 | -3.50 | -1.32 | 8.33 | 8.81 | 8.65 | 8.69 | 8.47 |
| ILMN_1792689 | HIST1H2AC | -4.76 | -1.25 | -2.64 | -3.79 | 8.40 | 9.73 | 8.75 | 9.14 | 9.46 |
| ILMN_1806692 | HEXB | -4.73 | 2.04 | 1.18 | -1.86 | 9.58 | 10.11 | 9.35 | 9.44 | 9.79 |
| ILMN_1880052 |  | -4.72 | -0.38 | -3.30 | -3.62 | 7.42 | 8.01 | 7.47 | 7.83 | 7.87 |
| ILMN_1678781 | SNX26 | -4.70 | -3.29 | -4.91 | -3.53 | 7.52 | 7.91 | 7.79 | 7.92 | 7.81 |
| ILMN_1807042 | MARCKS | -4.70 | -0.09 | -2.41 | 5.37 | 11.15 | 11.69 | 11.16 | 11.43 | 10.54 |
| ILMN_1673363 | CD97 | -4.70 | -1.28 | -4.76 | -1.27 | 8.56 | 9.58 | 8.84 | 9.60 | 8.84 |
| ILMN_2155272 | PIF1 | -4.69 | -0.29 | -4.11 | -2.35 | 8.40 | 9.32 | 8.45 | 9.20 | 8.86 |
| ILMN_1797172 | ERCC1 | -4.68 | -1.54 | -3.68 | -2.15 | 7.52 | 8.16 | 7.73 | 8.02 | 7.81 |
| ILMN_3238797 | FAM72A | -4.68 | 0.21 | -2.20 | -2.00 | 8.01 | 8.92 | 7.97 | 8.44 | 8.40 |
| ILMN_1662364 | AARS | -4.67 | -4.20 | -4.35 | 0.60 | 11.16 | 11.66 | 11.61 | 11.63 | 11.10 |
| ILMN_1734827 | MKI67 | -4.65 | -1.75 | -3.59 | -2.81 | 7.52 | 8.01 | 7.70 | 7.90 | 7.82 |
| ILMN_1745397 | OAS3 | -4.65 | -1.05 | -3.64 | -1.76 | 7.36 | 8.24 | 7.56 | 8.05 | 7.69 |
| ILMN_2108735 | EEF1A2 | -4.63 | -3.29 | -5.39 | -4.40 | 9.29 | 10.54 | 10.18 | 10.74 | 10.47 |
| ILMN_1775224 | NOS3 | -4.63 | -4.47 | -6.32 | -10.39 | 7.04 | 7.50 | 7.49 | 7.67 | 8.08 |
| ILMN_2371055 | EFNA1 | -4.61 | -0.90 | -0.66 | -1.35 | 6.55 | 7.06 | 6.65 | 6.62 | 6.70 |
| ILMN_1760649 | PCK2 | -4.60 | -2.32 | -3.96 | -2.09 | 6.62 | 6.96 | 6.79 | 6.91 | 6.78 |
| ILMN_1726456 | SLC3A2 | -4.59 | -2.65 | -3.74 | -2.26 | 8.75 | 9.37 | 9.11 | 9.25 | 9.05 |
| ILMN_2413508 | CD97 | -4.59 | -1.38 | -5.11 | -2.27 | 6.83 | 7.24 | 6.95 | 7.28 | 7.03 |
| ILMN_1728972 | FAM64A | -4.58 | 0.11 | -4.79 | -2.78 | 8.39 | 8.97 | 8.38 | 8.99 | 8.74 |
| ILMN_1701882 | LOC653820 | -4.58 | -0.98 | -2.02 | -2.63 | 8.43 | 9.61 | 8.68 | 8.95 | 9.11 |
| ILMN_1680643 | KIAA1333 | -4.57 | -0.93 | -3.07 | -3.40 | 8.02 | 8.66 | 8.15 | 8.45 | 8.49 |
| ILMN_3236713 | SNHG1 | -4.57 | 0.28 | 1.48 | -2.42 | 11.24 | 11.95 | 11.19 | 11.01 | 11.61 |
| ILMN_1832656 |  | -4.57 | -2.24 | -0.91 | -2.74 | 7.47 | 8.58 | 8.02 | 7.69 | 8.14 |
| ILMN_1693242 | ZNF296 | -4.55 | -0.10 | -2.67 | -2.19 | 10.83 | 11.26 | 10.84 | 11.08 | 11.04 |
| ILMN_1801939 | CCNB2 | -4.53 | 0.90 | -3.24 | -0.55 | 11.09 | 11.83 | 10.95 | 11.62 | 11.18 |
| ILMN_1758146 | SIRPA | -4.52 | -0.73 | -2.51 | -4.13 | 7.21 | 8.18 | 7.36 | 7.75 | 8.10 |
| ILMN_1700337 | TROAP | -4.52 | -0.90 | -2.98 | -3.44 | 8.95 | 9.80 | 9.12 | 9.51 | 9.60 |
| ILMN_1708098 | LIX1L | -4.52 | -2.01 | -4.77 | -4.91 | 7.76 | 8.42 | 8.05 | 8.45 | 8.47 |
| ILMN_1746148 | LRRC33 | -4.51 | -1.36 | -3.24 | -1.56 | 9.06 | 9.52 | 9.20 | 9.39 | 9.22 |
| ILMN_1785756 | LOC731314 | -4.50 | -2.29 | -4.77 | -4.37 | 9.30 | 9.78 | 9.55 | 9.81 | 9.77 |
| ILMN_1758778 | CEP110 | -4.49 | -1.35 | -1.47 | -3.39 | 8.37 | 8.74 | 8.48 | 8.49 | 8.65 |
| ILMN_1723971 | SLC29A1 | -4.48 | -3.59 | -3.86 | -5.33 | 9.01 | 9.56 | 9.45 | 9.48 | 9.66 |
| ILMN_3208881 | LOC647597 | -4.48 | -2.86 | -6.64 | -4.48 | 8.16 | 8.49 | 8.37 | 8.64 | 8.49 |
| ILMN_2042771 | PTTG1 | -4.47 | 0.48 | -1.86 | -1.33 | 11.64 | 12.26 | 11.58 | 11.90 | 11.83 |
| ILMN_2212690 | ZC3H7A | -4.47 | -1.47 | -0.53 | 4.59 | 7.82 | 8.07 | 7.90 | 7.85 | 7.56 |
| ILMN_1746699 | SGOL2 | -4.47 | 2.07 | -2.01 | -1.81 | 8.78 | 9.69 | 8.36 | 9.19 | 9.15 |
| ILMN_1737314 | BCL6 | -4.46 | -0.46 | -0.50 | -0.88 | 7.08 | 7.47 | 7.12 | 7.13 | 7.16 |
| ILMN_1708747 | OR6C4 | -4.44 | 0.30 | -0.77 | -1.13 | 6.55 | 6.80 | 6.54 | 6.60 | 6.62 |
| ILMN_1698478 | SNAPC2 | -4.43 | -2.09 | -3.37 | -4.76 | 7.91 | 8.32 | 8.10 | 8.22 | 8.35 |
| ILMN_1654421 | MPHOSPH9 | -4.42 | -0.80 | -2.43 | -1.90 | 7.69 | 8.17 | 7.78 | 7.95 | 7.90 |
| ILMN_1713990 | TRIP6 | -4.42 | -0.88 | -2.58 | -6.08 | 7.00 | 7.40 | 7.08 | 7.23 | 7.55 |
| ILMN_1774256 | PRSS21 | -4.42 | -2.82 | -4.27 | -10.59 | 8.83 | 9.22 | 9.08 | 9.21 | 9.78 |
| ILMN_1781285 | DUSP1 | -4.42 | 1.21 | 1.37 | 3.38 | 8.12 | 8.56 | 8.00 | 7.98 | 7.78 |
| ILMN_1662184 | C5orf34 | -4.42 | 0.53 | -1.40 | -3.54 | 7.64 | 8.06 | 7.59 | 7.78 | 7.98 |
| ILMN_1684549 | RNPC2 | -4.42 | -2.28 | -0.84 | -2.48 | 6.82 | 7.11 | 6.97 | 6.87 | 6.98 |
| ILMN_1729115 | LOC651816 | -4.40 | -0.38 | -3.54 | -2.48 | 9.17 | 10.16 | 9.26 | 9.97 | 9.73 |
| ILMN_1786564 | LOC497256 | -4.38 | 0.44 | 0.09 | 1.23 | 7.49 | 7.83 | 7.46 | 7.49 | 7.40 |
| ILMN_2200331 | H2AFX | -4.38 | -2.58 | -3.91 | -3.85 | 7.72 | 8.22 | 8.01 | 8.16 | 8.16 |
| ILMN_2067852 | SLC30A1 | -4.38 | -0.44 | -1.03 | -1.09 | 7.17 | 7.67 | 7.22 | 7.29 | 7.29 |
| ILMN_2265654 | UBE2C | -4.38 | -1.96 | -3.15 | -3.15 | 6.96 | 7.40 | 7.15 | 7.27 | 7.27 |
| ILMN_1762439 | C19orf6 | -4.37 | -2.00 | -2.05 | -2.65 | 7.29 | 7.87 | 7.55 | 7.56 | 7.64 |
| ILMN_3292320 | LOC642513 | -4.37 | -0.78 | -2.41 | -1.43 | 7.46 | 7.81 | 7.52 | 7.65 | 7.57 |
| ILMN_2225577 | C5orf37 | -4.36 | 1.25 | -0.08 | -0.03 | 9.18 | 9.69 | 9.03 | 9.19 | 9.18 |
| ILMN_1668228 | LOC136143 | -4.36 | -0.34 | 0.20 | -0.03 | 8.31 | 8.73 | 8.34 | 8.29 | 8.31 |
| ILMN_1780057 | RENBP | -4.35 | -2.52 | -2.97 | -7.74 | 8.34 | 9.36 | 8.93 | 9.04 | 10.15 |
| ILMN_1684571 | RSPH1 | -4.34 | -0.70 | -0.22 | -1.49 | 6.62 | 6.95 | 6.67 | 6.63 | 6.73 |
| ILMN_1701711 | BRD8 | -4.34 | 1.58 | 0.53 | -0.58 | 8.68 | 9.27 | 8.46 | 8.60 | 8.76 |
| ILMN_1712803 | CCNB1 | -4.34 | 0.74 | -2.02 | -1.78 | 10.37 | 11.02 | 10.26 | 10.67 | 10.64 |
| ILMN_1738407 | ULBP1 | -4.34 | -0.78 | -4.29 | -2.10 | 6.68 | 7.22 | 6.78 | 7.21 | 6.94 |
| ILMN_1683932 | ZNF425 | -4.34 | -1.49 | -2.85 | -2.70 | 7.04 | 7.51 | 7.20 | 7.35 | 7.33 |
| ILMN_2183389 | TTC9C | -4.33 | 0.07 | -2.01 | -5.15 | 7.85 | 8.27 | 7.84 | 8.04 | 8.35 |
| ILMN_1715437 | CASC5 | -4.33 | -0.95 | -2.15 | 1.30 | 7.41 | 7.82 | 7.50 | 7.61 | 7.29 |
| ILMN_1730906 | FILIP1L | -4.32 | 0.30 | -1.64 | -1.94 | 6.93 | 7.26 | 6.91 | 7.06 | 7.08 |
| ILMN_2063586 | CLIC4 | -4.31 | -1.01 | -4.47 | -1.11 | 10.06 | 10.50 | 10.17 | 10.52 | 10.18 |
| ILMN_1736234 | C1orf77 | -4.30 | -2.69 | -3.08 | -3.97 | 9.38 | 9.78 | 9.63 | 9.67 | 9.75 |
| ILMN_1745329 | PRR14 | -4.29 | -2.87 | -3.68 | -3.32 | 10.48 | 10.92 | 10.77 | 10.86 | 10.82 |
| ILMN_2253065 | H2AFJ | -4.29 | -0.35 | -1.66 | -1.59 | 7.47 | 8.20 | 7.53 | 7.76 | 7.74 |
| ILMN_1664511 | NDC80 | -4.28 | -0.45 | -2.73 | -3.04 | 9.39 | 10.36 | 9.49 | 10.01 | 10.08 |
| ILMN_2058782 | IFI27 | -4.27 | -2.52 | -3.46 | -1.91 | 8.98 | 11.44 | 10.43 | 10.97 | 10.08 |
| ILMN_1735014 | KLF6 | -4.26 | -0.17 | -1.91 | 1.84 | 9.45 | 10.27 | 9.48 | 9.82 | 9.10 |
| ILMN_1813338 | LAG3 | -4.25 | -2.95 | -2.48 | -2.54 | 6.77 | 7.22 | 7.08 | 7.03 | 7.04 |
| ILMN_2328666 | CD83 | -4.24 | -1.29 | 0.29 | -1.62 | 8.02 | 8.90 | 8.29 | 7.96 | 8.36 |
| ILMN_1698653 | ODF2 | -4.23 | -1.84 | -4.00 | -4.28 | 7.47 | 7.78 | 7.61 | 7.76 | 7.78 |
| ILMN_1684982 | PDK4 | -4.23 | -3.43 | -1.11 | -6.27 | 6.69 | 7.14 | 7.05 | 6.81 | 7.35 |
| ILMN_1812226 | ICAM1 | -4.22 | 1.26 | 0.65 | -0.70 | 6.80 | 7.08 | 6.71 | 6.75 | 6.84 |
| ILMN_1737918 | C1QA | -4.22 | 0.05 | -0.50 | -2.98 | 6.93 | 7.24 | 6.93 | 6.97 | 7.15 |
| ILMN_1726720 | NUSAP1 | -4.21 | 0.00 | -3.02 | -0.59 | 11.09 | 11.68 | 11.09 | 11.51 | 11.17 |
| ILMN_2049021 | PTTG3P | -4.21 | 2.14 | -1.29 | -0.28 | 10.95 | 11.60 | 10.62 | 11.15 | 10.99 |
| ILMN_3243274 | NCRNA00200 | -4.20 | -0.35 | -0.88 | -0.95 | 6.96 | 7.36 | 7.00 | 7.05 | 7.05 |
| ILMN_1766054 | ABCA1 | -4.20 | -3.00 | -0.95 | -2.74 | 10.29 | 10.83 | 10.68 | 10.41 | 10.64 |
| ILMN_1688953 | ARHGAP19 | -4.20 | -2.95 | -5.93 | -2.21 | 9.84 | 10.22 | 10.10 | 10.37 | 10.04 |
| ILMN_1671288 | ODF2 | -4.18 | -1.42 | -3.46 | -4.33 | 7.76 | 8.14 | 7.89 | 8.07 | 8.15 |
| ILMN_1880406 |  | -4.18 | -1.33 | -1.39 | -2.14 | 10.21 | 10.64 | 10.34 | 10.35 | 10.43 |
| ILMN_1716004 | NSUN4 | -4.18 | -0.95 | -2.61 | -1.29 | 8.30 | 8.75 | 8.40 | 8.58 | 8.44 |
| ILMN_1702933 | ADM2 | -4.18 | -1.92 | -6.90 | -3.94 | 6.83 | 7.24 | 7.02 | 7.51 | 7.22 |
| ILMN_1652749 | ERF | -4.17 | -2.30 | -4.37 | -3.73 | 7.47 | 7.82 | 7.66 | 7.84 | 7.78 |
| ILMN_2143155 | KIF11 | -4.16 | 0.60 | -2.29 | 1.31 | 10.51 | 11.02 | 10.43 | 10.79 | 10.35 |
| ILMN_1710514 | BCL3 | -4.14 | 0.00 | -1.86 | -2.21 | 8.17 | 8.74 | 8.17 | 8.43 | 8.47 |
| ILMN_1742779 | CENPL | -4.14 | 0.27 | -1.33 | -1.68 | 7.73 | 8.59 | 7.67 | 8.00 | 8.08 |
| ILMN_2286870 | CSNK1D | -4.14 | -2.32 | -1.77 | -1.39 | 7.66 | 7.98 | 7.84 | 7.80 | 7.77 |
| ILMN_1746704 | TRIM8 | -4.13 | -2.29 | -4.53 | -3.81 | 8.54 | 8.84 | 8.71 | 8.87 | 8.82 |
| ILMN_1758623 | HIST1H2BD | -4.12 | -0.43 | -2.37 | -2.93 | 8.57 | 9.46 | 8.66 | 9.08 | 9.20 |
| ILMN_1723522 | APOLD1 | -4.11 | 0.19 | -4.33 | -2.20 | 7.15 | 7.57 | 7.13 | 7.60 | 7.38 |
| ILMN_2342240 | MGAT2 | -4.10 | -1.50 | -3.38 | -5.18 | 9.89 | 10.25 | 10.02 | 10.18 | 10.34 |
| ILMN_3231026 | LOC654780 | -4.10 | -1.09 | -0.68 | -0.12 | 6.94 | 7.50 | 7.09 | 7.03 | 6.95 |
| ILMN_2196984 | OIP5 | -4.10 | 1.24 | -3.88 | 1.35 | 10.99 | 11.35 | 10.88 | 11.33 | 10.87 |
| ILMN_1717173 | ECT2 | -4.10 | -0.34 | -2.84 | 0.45 | 10.12 | 10.76 | 10.17 | 10.56 | 10.05 |
| ILMN_1753183 | CDCA4 | -4.09 | -0.88 | -0.80 | -0.76 | 9.11 | 9.44 | 9.18 | 9.18 | 9.17 |
| ILMN_2382964 | PRSS21 | -4.06 | -2.61 | -4.73 | -10.43 | 6.97 | 7.28 | 7.17 | 7.33 | 7.76 |
| ILMN_2141807 | C15orf23 | -4.06 | 0.85 | -1.75 | -0.96 | 8.36 | 9.23 | 8.17 | 8.73 | 8.56 |
| ILMN_1806408 | ACADVL | -4.06 | -1.45 | -2.43 | -3.82 | 9.84 | 10.29 | 10.00 | 10.11 | 10.27 |
| ILMN_1716400 | FOXM1 | -4.04 | -1.19 | -2.11 | -2.82 | 8.09 | 8.53 | 8.22 | 8.32 | 8.39 |
| ILMN_1753196 | PTTG1 | -4.03 | 1.85 | -1.29 | -1.43 | 10.41 | 11.09 | 10.10 | 10.63 | 10.65 |
| ILMN_1741475 | C7orf47 | -4.03 | 0.27 | -3.01 | -1.94 | 9.97 | 10.42 | 9.94 | 10.31 | 10.19 |
| ILMN_1728512 | YWHAH | -4.02 | -1.35 | -4.62 | -0.63 | 11.13 | 11.56 | 11.28 | 11.62 | 11.20 |
| ILMN_2404906 | SGOL1 | -4.02 | -0.07 | -0.35 | -0.98 | 7.33 | 7.72 | 7.34 | 7.37 | 7.43 |
| ILMN_2360415 | PRNP | -3.99 | -1.76 | -2.29 | -4.95 | 7.11 | 7.51 | 7.28 | 7.34 | 7.60 |
| ILMN_1652008 | C15orf23 | -3.98 | 1.14 | -1.53 | -0.58 | 8.04 | 8.87 | 7.81 | 8.36 | 8.16 |
| ILMN_2179837 | BANF1 | -3.97 | -1.37 | -1.82 | -2.00 | 11.20 | 11.64 | 11.35 | 11.40 | 11.42 |
| ILMN_1751598 | SESN2 | -3.95 | -1.60 | -4.75 | -0.96 | 6.63 | 6.96 | 6.76 | 7.03 | 6.71 |
| ILMN_1810214 | JUND | -3.95 | -1.43 | -2.19 | -0.25 | 11.69 | 12.21 | 11.88 | 11.98 | 11.72 |
| ILMN_1775762 | GNAI2 | -3.94 | -1.43 | -2.20 | -0.77 | 9.83 | 10.34 | 10.01 | 10.11 | 9.93 |
| ILMN_1693227 | ZC3H7A | -3.94 | 0.14 | -1.32 | 1.03 | 9.51 | 10.00 | 9.49 | 9.67 | 9.38 |
| ILMN_1779257 | CD40 | -3.93 | -0.22 | 0.71 | -5.05 | 7.71 | 8.19 | 7.73 | 7.62 | 8.32 |
| ILMN_1734317 | DPF2 | -3.93 | -2.00 | -1.78 | -3.18 | 9.83 | 10.26 | 10.05 | 10.03 | 10.18 |
| ILMN_1658847 | MGC61598 | -3.93 | 0.07 | -1.47 | -3.86 | 7.98 | 8.34 | 7.97 | 8.11 | 8.34 |
| ILMN_1680626 | PDIA6 | -3.93 | -1.42 | -0.97 | -1.91 | 7.77 | 8.47 | 8.03 | 7.95 | 8.11 |
| ILMN_1807277 | IFI30 | -3.92 | -2.08 | -1.39 | -9.38 | 7.31 | 7.68 | 7.51 | 7.44 | 8.21 |
| ILMN_1806275 | ADAT3 | -3.92 | -2.07 | -3.80 | -1.11 | 7.53 | 7.87 | 7.71 | 7.86 | 7.63 |
| ILMN_2072296 | CKS2 | -3.92 | -0.17 | -2.17 | -1.96 | 12.24 | 12.94 | 12.27 | 12.63 | 12.59 |
| ILMN_2409220 | HMMR | -3.92 | 1.48 | -1.11 | -0.86 | 10.47 | 11.06 | 10.25 | 10.64 | 10.60 |
| ILMN_1666179 | HIST2H3C | -3.90 | -2.04 | -1.75 | -2.49 | 6.85 | 7.20 | 7.04 | 7.01 | 7.08 |
| ILMN_1739805 | NDE1 | -3.90 | 0.09 | -2.20 | -1.51 | 9.27 | 9.83 | 9.25 | 9.59 | 9.49 |
| ILMN_1708160 | KPNA2 | -3.90 | 0.92 | -2.41 | -0.75 | 9.05 | 9.77 | 8.88 | 9.50 | 9.19 |
| ILMN_1654065 | ATOH8 | -3.90 | -1.32 | -5.55 | -2.01 | 7.08 | 7.51 | 7.22 | 7.69 | 7.30 |
| ILMN_1653822 | NEK2 | -3.90 | 0.17 | -2.85 | -2.05 | 7.68 | 8.20 | 7.65 | 8.06 | 7.95 |
| ILMN_2168347 | EPOR | -3.90 | -1.47 | -4.69 | -7.42 | 7.23 | 7.66 | 7.39 | 7.74 | 8.04 |
| ILMN_2408400 | NSUN5 | -3.89 | -1.60 | -2.05 | -3.06 | 7.91 | 8.28 | 8.06 | 8.11 | 8.20 |
| ILMN_1654942 | NFIC | -3.89 | -0.27 | -0.35 | 0.53 | 6.78 | 7.07 | 6.80 | 6.80 | 6.74 |
| ILMN_2223911 | ADAM3A | -3.88 | -0.46 | -0.41 | -1.63 | 6.52 | 6.78 | 6.55 | 6.55 | 6.63 |
| ILMN_2408179 | BAT2 | -3.87 | -2.87 | -4.33 | -3.66 | 7.28 | 7.71 | 7.60 | 7.76 | 7.68 |
| ILMN_1703564 | DYNLRB1 | -3.86 | -0.99 | -1.58 | -2.50 | 8.94 | 9.29 | 9.03 | 9.08 | 9.16 |
| ILMN_1729374 | ETFB | -3.86 | 1.11 | -0.35 | -0.35 | 8.52 | 9.23 | 8.32 | 8.58 | 8.58 |
| ILMN_1666305 | CDKN3 | -3.83 | 2.40 | -2.10 | -0.34 | 9.86 | 10.46 | 9.49 | 10.19 | 9.91 |
| ILMN_1855286 |  | -3.82 | 0.42 | -0.47 | -2.97 | 6.77 | 7.07 | 6.74 | 6.81 | 7.01 |
| ILMN_2098446 | PMAIP1 | -3.82 | -1.24 | -2.68 | -0.06 | 9.93 | 10.84 | 10.23 | 10.57 | 9.94 |
| ILMN_3266197 | LOC100130171 | -3.82 | 0.05 | -3.68 | -1.13 | 6.85 | 7.12 | 6.85 | 7.11 | 6.93 |
| ILMN_1739450 | NFE2L1 | -3.81 | -1.91 | -4.18 | -2.98 | 8.32 | 8.84 | 8.58 | 8.89 | 8.73 |
| ILMN_2065773 | SCG5 | -3.80 | 0.44 | -2.42 | 0.40 | 7.46 | 8.10 | 7.39 | 7.87 | 7.40 |
| ILMN_2325234 | PQBP1 | -3.79 | -1.79 | -3.00 | -7.16 | 8.71 | 9.01 | 8.85 | 8.95 | 9.28 |
| ILMN_1795448 | OR2B6 | -3.79 | -0.97 | -1.29 | -3.72 | 6.77 | 7.13 | 6.86 | 6.89 | 7.12 |
| ILMN_3235517 | ZNF777 | -3.78 | -3.72 | -3.78 | -2.11 | 8.20 | 8.57 | 8.57 | 8.57 | 8.41 |
| ILMN_1784602 | CDKN1A | -3.78 | 0.64 | -3.21 | -3.93 | 7.68 | 8.70 | 7.51 | 8.55 | 8.74 |
| ILMN_2307025 | CPNE1 | -3.78 | -4.12 | -2.69 | -8.60 | 8.58 | 9.03 | 9.07 | 8.90 | 9.61 |
| ILMN_1829989 |  | -3.78 | -0.16 | -0.97 | 6.05 | 8.00 | 8.31 | 8.01 | 8.08 | 7.50 |
| ILMN_3244669 | LOC100134329 | -3.75 | -0.46 | -1.18 | -3.23 | 6.84 | 7.09 | 6.87 | 6.92 | 7.05 |
| ILMN_2299612 | TMEM150A | -3.73 | -2.69 | -4.61 | -5.32 | 7.55 | 7.99 | 7.87 | 8.09 | 8.18 |
| ILMN_2346649 | ZNF473 | -3.72 | -2.06 | -2.33 | -5.78 | 7.49 | 7.71 | 7.61 | 7.63 | 7.84 |
| ILMN_1795930 | PTGER4 | -3.72 | -0.66 | -0.34 | -4.34 | 8.33 | 8.95 | 8.44 | 8.38 | 9.05 |
| ILMN_1721022 | SHC1 | -3.70 | -1.42 | -1.39 | -4.93 | 9.63 | 10.00 | 9.77 | 9.77 | 10.12 |
| ILMN_1821517 |  | -3.70 | -1.37 | -3.10 | -3.01 | 12.93 | 13.48 | 13.13 | 13.39 | 13.37 |
| ILMN_1771039 | GTSE1 | -3.70 | 0.80 | -3.98 | -1.19 | 8.13 | 8.75 | 7.99 | 8.79 | 8.33 |
| ILMN_2086095 | ID2 | -3.70 | -1.94 | -4.94 | -6.30 | 9.15 | 9.89 | 9.54 | 10.14 | 10.42 |
| ILMN_1791726 | TUBB3 | -3.69 | -2.67 | -4.99 | 3.26 | 9.19 | 9.84 | 9.66 | 10.07 | 8.61 |
| ILMN_1672128 | ATF4 | -3.68 | -1.81 | -3.86 | -1.79 | 12.33 | 12.98 | 12.65 | 13.01 | 12.65 |
| ILMN_2389013 | ADRM1 | -3.66 | -3.54 | -4.63 | -6.83 | 8.97 | 9.29 | 9.28 | 9.38 | 9.58 |
| ILMN_2354515 | NDUFAF3 | -3.64 | -3.37 | -4.68 | -2.30 | 11.24 | 11.64 | 11.61 | 11.76 | 11.49 |
| ILMN_1720053 | ZFAND3 | -3.62 | -2.56 | -4.09 | -2.88 | 7.57 | 7.83 | 7.76 | 7.87 | 7.78 |
| ILMN_1785646 | PMP22 | -3.62 | 0.62 | -0.20 | 2.86 | 8.16 | 8.90 | 8.04 | 8.20 | 7.58 |
| ILMN_1809818 | PRCC | -3.61 | -2.13 | -2.17 | -5.97 | 7.38 | 7.70 | 7.57 | 7.57 | 7.90 |
| ILMN_1701455 | FBXO6 | -3.61 | -1.33 | -3.46 | -5.01 | 7.59 | 8.14 | 7.79 | 8.12 | 8.36 |
| ILMN_1766514 | LOC650274 | -3.61 | -1.44 | -2.89 | -3.21 | 6.72 | 6.98 | 6.82 | 6.93 | 6.95 |
| ILMN_1772487 | SFRS14 | -3.60 | -2.44 | -2.78 | -4.28 | 8.27 | 8.69 | 8.55 | 8.59 | 8.77 |
| ILMN_1689525 | PMAIP1 | -3.59 | -0.95 | -3.97 | 0.23 | 7.87 | 8.43 | 8.02 | 8.49 | 7.83 |
| ILMN_1732071 | HIST2H2BE | -3.58 | -1.62 | -1.45 | -3.29 | 8.60 | 9.38 | 8.95 | 8.92 | 9.32 |
| ILMN_1755303 | ZNF217 | -3.58 | -2.80 | -3.21 | -3.65 | 9.37 | 9.70 | 9.63 | 9.66 | 9.70 |
| ILMN_1652906 | GBGT1 | -3.56 | -0.98 | -2.31 | -3.21 | 6.81 | 7.12 | 6.90 | 7.01 | 7.09 |
| ILMN_1670096 | NRBP1 | -3.56 | -2.78 | -2.61 | -2.85 | 7.30 | 7.65 | 7.57 | 7.56 | 7.58 |
| ILMN_1670841 | CPNE1 | -3.56 | -3.61 | -2.62 | -5.82 | 9.26 | 9.70 | 9.70 | 9.58 | 9.98 |
| ILMN_1684346 | TNFAIP8L1 | -3.55 | -1.50 | -3.84 | -2.90 | 7.53 | 8.10 | 7.77 | 8.15 | 8.00 |
| ILMN_1781479 | SUV39H1 | -3.55 | -1.80 | -4.07 | -2.23 | 9.59 | 9.86 | 9.73 | 9.90 | 9.76 |
| ILMN_1736751 | VPS26B | -3.54 | -2.05 | -3.00 | -3.73 | 7.25 | 7.51 | 7.40 | 7.47 | 7.52 |
| ILMN_1694731 | CLCN7 | -3.54 | -2.95 | -2.21 | -4.22 | 9.55 | 9.97 | 9.90 | 9.81 | 10.05 |
| ILMN_1662340 | ZNF358 | -3.54 | -4.79 | -5.24 | -3.34 | 8.69 | 9.12 | 9.27 | 9.32 | 9.09 |
| ILMN_2358457 | ATF4 | -3.53 | -2.22 | -4.43 | -1.57 | 8.84 | 9.55 | 9.29 | 9.73 | 9.16 |
| ILMN_2367469 | CARS | -3.52 | -2.65 | -4.25 | -2.61 | 8.31 | 8.81 | 8.69 | 8.91 | 8.68 |
| ILMN_1766637 | GLA | -3.51 | 1.03 | -3.90 | 1.24 | 9.74 | 10.07 | 9.64 | 10.11 | 9.62 |
| ILMN_1797786 | MAPK12 | -3.51 | -2.84 | -3.92 | -5.15 | 7.13 | 7.50 | 7.43 | 7.55 | 7.68 |
| ILMN_1683450 | CDCA5 | -3.50 | -1.69 | -1.49 | -3.82 | 11.31 | 11.60 | 11.45 | 11.43 | 11.63 |
| ILMN_1672843 | FBXO8 | -3.50 | -0.71 | -1.96 | 3.45 | 8.28 | 8.56 | 8.34 | 8.44 | 8.01 |
| ILMN_1812403 | BCAP31 | -3.49 | -2.73 | -3.19 | -5.65 | 9.10 | 9.48 | 9.40 | 9.45 | 9.72 |
| ILMN_2223350 | C13orf1 | -3.48 | -1.46 | -4.06 | -3.18 | 7.59 | 8.09 | 7.80 | 8.17 | 8.05 |
| ILMN_1747598 | PPP1R11 | -3.47 | -2.97 | -4.27 | -3.77 | 9.76 | 10.10 | 10.05 | 10.18 | 10.13 |
| ILMN_1792733 | FOXA3 | -3.47 | -1.93 | -3.74 | -7.09 | 6.82 | 7.21 | 7.03 | 7.24 | 7.61 |
| ILMN_1652762 | HIC2 | -3.47 | -4.08 | -3.93 | -2.06 | 7.40 | 7.70 | 7.75 | 7.74 | 7.58 |
| ILMN_1733107 | NOC4L | -3.47 | -3.80 | -4.80 | -6.74 | 7.45 | 7.69 | 7.72 | 7.79 | 7.92 |
| ILMN_1794017 | SERTAD1 | -3.47 | -0.66 | -2.41 | -3.35 | 7.67 | 8.05 | 7.74 | 7.94 | 8.04 |
| ILMN_1772645 | AGK | -3.45 | -2.80 | -2.60 | -5.54 | 7.56 | 7.98 | 7.90 | 7.88 | 8.23 |
| ILMN_1793712 | SCAMP3 | -3.45 | -1.69 | -2.05 | -6.57 | 9.53 | 9.89 | 9.71 | 9.74 | 10.21 |
| ILMN_1674698 | AARS2 | -3.43 | -2.96 | -3.21 | -4.26 | 8.20 | 8.57 | 8.52 | 8.54 | 8.66 |
| ILMN_1803673 | LOC113230 | -3.42 | -3.19 | -2.32 | -5.22 | 6.59 | 6.79 | 6.77 | 6.72 | 6.89 |
| ILMN_1737164 | TM9SF1 | -3.42 | -2.39 | -2.11 | -4.77 | 6.91 | 7.12 | 7.06 | 7.04 | 7.20 |
| ILMN_1737988 | PRNP | -3.40 | -1.54 | -2.49 | -6.18 | 8.78 | 9.28 | 9.01 | 9.15 | 9.69 |
| ILMN_3261811 | C14orf167 | -3.39 | -2.43 | -3.15 | -3.26 | 7.51 | 7.93 | 7.81 | 7.90 | 7.92 |
| ILMN_2345016 | PTGES2 | -3.39 | -2.74 | -2.97 | -6.90 | 7.96 | 8.25 | 8.20 | 8.22 | 8.56 |
| ILMN_1675472 | LOC644799 | -3.38 | -1.86 | -2.91 | -3.18 | 9.36 | 9.81 | 9.61 | 9.75 | 9.78 |
| ILMN_1806867 | PPM1G | -3.37 | -4.19 | -3.44 | -2.74 | 11.33 | 11.68 | 11.77 | 11.69 | 11.62 |
| ILMN_2064694 | STIM1 | -3.36 | -2.23 | -4.29 | -6.01 | 7.88 | 8.14 | 8.05 | 8.22 | 8.35 |
| ILMN_2333440 | TM9SF1 | -3.35 | -0.82 | -0.86 | -4.01 | 6.73 | 7.00 | 6.80 | 6.80 | 7.06 |
| ILMN_1731783 | ATP1A1 | -3.35 | -1.71 | -2.12 | -5.17 | 8.37 | 8.67 | 8.53 | 8.56 | 8.84 |
| ILMN_1784717 | RPS19 | -3.35 | -1.66 | -2.76 | -2.92 | 13.95 | 14.23 | 14.09 | 14.18 | 14.20 |
| ILMN_1654893 | SCAMP2 | -3.34 | -1.15 | -1.46 | -3.27 | 7.15 | 7.47 | 7.26 | 7.29 | 7.47 |
| ILMN_2374352 | DBNDD1 | -3.33 | -4.24 | -6.80 | 0.21 | 7.29 | 7.60 | 7.69 | 7.93 | 7.27 |
| ILMN_1796490 | GRINA | -3.33 | -1.86 | -2.12 | -3.10 | 7.41 | 7.79 | 7.62 | 7.65 | 7.76 |
| ILMN_1811921 | CSRP1 | -3.33 | -0.68 | -2.92 | -8.18 | 7.19 | 7.60 | 7.27 | 7.55 | 8.19 |
| ILMN_1761247 | PIR | -3.33 | -1.04 | -4.15 | -2.89 | 6.73 | 6.99 | 6.81 | 7.05 | 6.96 |
| ILMN_1773780 | FAM173A | -3.30 | -3.02 | -3.18 | -5.01 | 8.06 | 8.42 | 8.39 | 8.40 | 8.60 |
| ILMN_2367710 | PKN1 | -3.29 | -2.15 | -3.11 | -4.65 | 7.23 | 7.48 | 7.39 | 7.47 | 7.58 |
| ILMN_2078389 | SLC4A2 | -3.26 | -3.58 | -3.42 | -7.20 | 7.45 | 7.79 | 7.82 | 7.80 | 8.20 |
| ILMN_1783753 | TXNDC12 | -3.26 | -0.93 | -3.06 | -4.86 | 9.53 | 9.91 | 9.64 | 9.88 | 10.09 |
| ILMN_1731275 | DPEP3 | -3.26 | -2.41 | -3.47 | -3.41 | 11.05 | 11.61 | 11.47 | 11.65 | 11.64 |
| ILMN_1716276 | CCL4L2 | -3.24 | 0.15 | -0.48 | -4.19 | 6.91 | 7.47 | 6.88 | 6.99 | 7.63 |
| ILMN_1793990 | ID2 | -3.24 | -1.49 | -5.45 | -7.24 | 7.93 | 8.61 | 8.25 | 9.08 | 9.45 |
| ILMN_1746090 | STT3A | -3.23 | -0.33 | -2.60 | -4.67 | 8.96 | 9.35 | 9.00 | 9.27 | 9.52 |
| ILMN_1655549 | SIGLEC10 | -3.22 | -2.14 | -4.46 | -5.79 | 7.89 | 8.24 | 8.12 | 8.37 | 8.51 |
| ILMN_1815346 | TMEM136 | -3.22 | -1.38 | -2.01 | -5.54 | 7.05 | 7.54 | 7.26 | 7.35 | 7.89 |
| ILMN_2351309 | TIAL1 | -3.22 | -2.87 | -3.07 | -2.72 | 8.57 | 8.94 | 8.90 | 8.93 | 8.89 |
| ILMN_1666208 | C14orf106 | -3.20 | -1.33 | -2.72 | -4.11 | 10.96 | 11.29 | 11.10 | 11.24 | 11.39 |
| ILMN_2361163 | SSBP3 | -3.20 | -1.92 | -3.84 | -1.80 | 7.19 | 7.46 | 7.35 | 7.51 | 7.34 |
| ILMN_3245983 | NEURL1B | -3.20 | -0.41 | -3.58 | -3.55 | 7.93 | 8.30 | 7.98 | 8.34 | 8.34 |
| ILMN_1800308 | GTF2H4 | -3.18 | -2.97 | -2.50 | -3.65 | 8.38 | 8.72 | 8.70 | 8.65 | 8.77 |
| ILMN_1789642 | DNAJC5 | -3.18 | -2.14 | -2.11 | -3.44 | 7.10 | 7.38 | 7.29 | 7.29 | 7.41 |
| ILMN_1690653 | CDK2AP2 | -3.17 | -2.13 | -4.56 | -6.03 | 8.02 | 8.29 | 8.20 | 8.41 | 8.54 |
| ILMN_1776998 | DNAJA4 | -3.17 | -2.23 | -2.37 | -3.20 | 7.13 | 7.44 | 7.35 | 7.36 | 7.45 |
| ILMN_1685005 | TNFRSF1A | -3.16 | -1.89 | -2.40 | -3.42 | 6.79 | 7.12 | 6.99 | 7.04 | 7.15 |
| ILMN_3247261 | RAPGEF2 | -3.16 | -1.94 | -0.42 | 4.00 | 7.98 | 8.23 | 8.14 | 8.02 | 7.67 |
| ILMN_1813374 | C19orf28 | -3.15 | -3.78 | -5.56 | -4.52 | 7.71 | 7.96 | 8.01 | 8.16 | 8.07 |
| ILMN_1692962 | CTDSP2 | -3.14 | -4.04 | -3.38 | -4.04 | 10.34 | 10.69 | 10.79 | 10.71 | 10.79 |
| ILMN_1799667 | KIF4A | -3.12 | 0.34 | -4.11 | 2.22 | 8.64 | 9.01 | 8.60 | 9.12 | 8.37 |
| ILMN_2319996 | RPL3 | -3.12 | -0.44 | -2.65 | -3.06 | 13.11 | 13.44 | 13.16 | 13.39 | 13.44 |
| ILMN_3238123 | ZNF844 | -3.12 | -2.33 | -1.88 | -3.26 | 6.68 | 6.98 | 6.90 | 6.86 | 6.99 |
| ILMN_2402499 | SC4MOL | -3.10 | -2.96 | -2.46 | -3.01 | 7.02 | 7.25 | 7.24 | 7.20 | 7.24 |
| ILMN_1695759 | AMDHD2 | -3.09 | -2.79 | -1.80 | -4.37 | 7.76 | 8.14 | 8.10 | 7.98 | 8.30 |
| ILMN_1788095 | SPRYD3 | -3.08 | -1.91 | -1.91 | -5.12 | 7.03 | 7.27 | 7.18 | 7.18 | 7.43 |
| ILMN_1803317 | C9orf114 | -3.07 | -2.12 | -2.67 | -3.81 | 8.90 | 9.18 | 9.10 | 9.15 | 9.25 |
| ILMN_1759097 | MLLT11 | -3.05 | -1.81 | -4.98 | 2.29 | 10.32 | 10.66 | 10.52 | 10.87 | 10.07 |
| ILMN_1692865 | VPS37D | -3.05 | -1.45 | -2.53 | -3.61 | 7.49 | 7.90 | 7.69 | 7.83 | 7.98 |
| ILMN_1703471 | ATF6 | -3.04 | -1.54 | -3.04 | -3.01 | 8.95 | 9.24 | 9.10 | 9.24 | 9.23 |
| ILMN_1762529 | SLC12A8 | -3.04 | -1.00 | -4.00 | 0.42 | 6.89 | 7.13 | 6.97 | 7.21 | 6.85 |
| ILMN_3290019 | LOC646753 | -3.03 | -0.69 | -1.08 | -4.92 | 9.18 | 9.59 | 9.27 | 9.32 | 9.85 |
| ILMN_2082209 | C20orf100 | -3.03 | -2.48 | -1.13 | -6.67 | 8.62 | 9.17 | 9.07 | 8.82 | 9.84 |
| ILMN_2234970 | SLC39A3 | -3.03 | -2.13 | -4.25 | -5.72 | 9.66 | 10.00 | 9.90 | 10.13 | 10.30 |
| ILMN_1731720 | PDRG1 | -3.03 | -1.32 | 0.19 | -2.93 | 8.45 | 8.77 | 8.59 | 8.43 | 8.76 |
| ILMN_1701558 | MAP1A | -3.01 | -1.31 | -1.12 | 2.76 | 7.18 | 7.51 | 7.32 | 7.30 | 6.88 |
| ILMN_1665865 | IGFBP4 | -2.99 | -0.88 | -1.87 | -4.14 | 6.67 | 6.96 | 6.76 | 6.85 | 7.08 |
| ILMN_2367818 | CD40 | -2.99 | -0.24 | 0.37 | -2.85 | 7.21 | 7.58 | 7.24 | 7.16 | 7.57 |
| ILMN_1776723 | PHF11 | -2.97 | -0.16 | -1.39 | 2.74 | 10.28 | 10.71 | 10.31 | 10.48 | 9.89 |
| ILMN_1716979 | GSTM4 | -2.97 | -1.36 | -2.37 | -3.02 | 6.71 | 6.90 | 6.80 | 6.86 | 6.91 |
| ILMN_3187771 | C14orf167 | -2.97 | -2.06 | -2.60 | -2.78 | 7.99 | 8.47 | 8.32 | 8.41 | 8.44 |
| ILMN_2214197 | TP53INP1 | -2.96 | -2.35 | -1.73 | -4.27 | 7.11 | 7.42 | 7.35 | 7.29 | 7.55 |
| ILMN_2374159 | HERPUD1 | -2.96 | -1.14 | 0.06 | 2.96 | 10.06 | 10.41 | 10.19 | 10.05 | 9.70 |
| ILMN_1748124 | TSC22D3 | -2.95 | -1.66 | -3.75 | -2.88 | 9.98 | 11.00 | 10.55 | 11.27 | 10.97 |
| ILMN_1736575 | TRIM28 | -2.95 | -2.63 | -4.38 | -4.80 | 8.92 | 9.23 | 9.19 | 9.37 | 9.42 |
| ILMN_1702197 | C9orf140 | -2.95 | 0.94 | -4.23 | -0.88 | 8.48 | 8.94 | 8.34 | 9.14 | 8.62 |
| ILMN_1671005 | IRF2BP2 | -2.95 | -1.96 | -3.67 | -3.06 | 7.45 | 7.80 | 7.68 | 7.89 | 7.82 |
| ILMN_1745655 | PEX16 | -2.94 | -1.68 | -2.12 | -5.58 | 8.81 | 9.09 | 8.97 | 9.01 | 9.35 |
| ILMN_1716907 | FLJ20254 | -2.94 | -3.94 | -3.19 | -5.63 | 7.11 | 7.31 | 7.38 | 7.33 | 7.49 |
| ILMN_1797828 | DDRGK1 | -2.92 | 0.04 | -0.40 | -3.03 | 8.76 | 9.03 | 8.76 | 8.80 | 9.04 |
| ILMN_1711596 | PQBP1 | -2.92 | -1.31 | -1.57 | -5.77 | 7.87 | 8.13 | 7.99 | 8.01 | 8.38 |
| ILMN_1796013 | PYCR1 | -2.91 | -4.05 | -3.29 | -3.13 | 7.91 | 8.21 | 8.33 | 8.25 | 8.24 |
| ILMN_2365465 | XBP1 | -2.91 | 1.08 | -0.73 | 4.14 | 8.97 | 9.36 | 8.83 | 9.07 | 8.42 |
| ILMN_3240354 | CYCSP52 | -2.91 | 0.90 | 0.23 | 3.32 | 8.60 | 9.18 | 8.42 | 8.55 | 7.93 |
| ILMN_2403994 | RALY | -2.90 | -1.81 | -1.92 | -3.70 | 9.10 | 9.47 | 9.33 | 9.34 | 9.57 |
| ILMN_1780141 | TMEM66 | -2.90 | -0.79 | -0.79 | -4.08 | 10.88 | 11.15 | 10.95 | 10.95 | 11.26 |
| ILMN_1778010 | IL32 | -2.90 | -0.71 | -0.71 | -3.83 | 6.77 | 7.03 | 6.83 | 6.83 | 7.11 |
| ILMN_2231021 | TMEM185B | -2.90 | -1.26 | -2.40 | -5.03 | 7.13 | 7.38 | 7.24 | 7.34 | 7.57 |
| ILMN_1692938 | PSAT1 | -2.90 | -1.50 | -4.14 | -0.65 | 8.80 | 9.41 | 9.11 | 9.67 | 8.93 |
| ILMN_1704571 | FAM53B | -2.90 | -6.74 | -7.39 | -2.51 | 8.35 | 8.60 | 8.94 | 9.00 | 8.57 |
| ILMN_2069593 | SFRS2IP | -2.89 | 0.33 | -2.81 | -5.29 | 8.50 | 8.74 | 8.48 | 8.73 | 8.94 |
| ILMN_1748034 | KLHDC4 | -2.88 | -4.05 | -5.14 | 6.33 | 9.03 | 9.30 | 9.41 | 9.51 | 8.43 |
| ILMN_1669674 | CNPY3 | -2.88 | -1.11 | -2.19 | -4.51 | 8.61 | 9.05 | 8.78 | 8.95 | 9.30 |
| ILMN_1687941 | RNF4 | -2.88 | -3.01 | -2.58 | -4.02 | 7.80 | 8.02 | 8.03 | 8.00 | 8.11 |
| ILMN_1815086 | NINJ1 | -2.88 | -0.90 | -3.35 | -4.74 | 8.60 | 9.07 | 8.74 | 9.14 | 9.37 |
| ILMN_1774596 | BSCL2 | -2.88 | -2.06 | -3.53 | -4.74 | 9.21 | 9.59 | 9.48 | 9.68 | 9.84 |
| ILMN_2126239 | SMG5 | -2.88 | -1.91 | -2.25 | -3.10 | 9.04 | 9.35 | 9.25 | 9.28 | 9.37 |
| ILMN_1797531 | PRKAG2 | -2.88 | -1.13 | -2.02 | -5.45 | 7.23 | 7.55 | 7.36 | 7.46 | 7.84 |
| ILMN_1722774 | VPS72 | -2.87 | -3.17 | -2.73 | -6.07 | 8.05 | 8.34 | 8.37 | 8.33 | 8.66 |
| ILMN_1799819 | MARS | -2.87 | -2.70 | -3.95 | -2.89 | 10.41 | 10.86 | 10.84 | 11.03 | 10.87 |
| ILMN_1674874 | MFSD10 | -2.86 | -2.90 | -3.98 | -3.77 | 10.30 | 10.57 | 10.57 | 10.68 | 10.66 |
| ILMN_2346479 | HOMER2 | -2.86 | 1.36 | 1.43 | 4.51 | 7.13 | 7.40 | 7.00 | 7.00 | 6.71 |
| ILMN_2340877 | MEN1 | -2.84 | -4.64 | -4.29 | -7.70 | 7.46 | 7.68 | 7.81 | 7.79 | 8.05 |
| ILMN_1791388 | ZNF787 | -2.84 | -1.75 | -3.71 | -3.77 | 8.42 | 8.72 | 8.60 | 8.81 | 8.82 |
| ILMN_1765446 | EMP3 | -2.84 | -1.32 | -3.82 | -4.42 | 8.11 | 8.62 | 8.35 | 8.80 | 8.90 |
| ILMN_1673649 | HYOU1 | -2.84 | -2.07 | -1.27 | -3.41 | 7.30 | 7.55 | 7.48 | 7.41 | 7.60 |
| ILMN_1682567 | CCDC106 | -2.84 | -4.34 | -4.54 | -3.69 | 8.16 | 8.49 | 8.67 | 8.69 | 8.59 |
| ILMN_1692760 | DPAGT1 | -2.83 | -1.82 | -4.07 | -8.36 | 9.39 | 9.65 | 9.55 | 9.76 | 10.15 |
| ILMN_1665831 | CLPTM1 | -2.83 | -2.27 | -3.56 | -6.09 | 8.56 | 8.85 | 8.79 | 8.92 | 9.18 |
| ILMN_1666050 | TMUB1 | -2.83 | -3.02 | -3.45 | -4.31 | 9.37 | 9.72 | 9.74 | 9.79 | 9.90 |
| ILMN_3239177 | MEX3A | -2.83 | -2.95 | -4.68 | -2.78 | 7.41 | 7.64 | 7.65 | 7.78 | 7.63 |
| ILMN_1778374 | BSG | -2.82 | -1.67 | -4.37 | -4.59 | 9.59 | 9.96 | 9.81 | 10.17 | 10.20 |
| ILMN_1711361 | ZNF319 | -2.82 | -3.22 | -4.23 | 1.41 | 7.86 | 8.05 | 8.08 | 8.14 | 7.77 |
| ILMN_1663954 | TH1L | -2.81 | -2.24 | -3.02 | -3.93 | 10.63 | 10.90 | 10.85 | 10.92 | 11.01 |
| ILMN_1678490 | RILPL2 | -2.81 | -1.23 | -1.83 | -3.22 | 7.97 | 8.32 | 8.12 | 8.20 | 8.37 |
| ILMN_1707720 | SLC1A5 | -2.81 | -2.33 | -4.99 | -1.30 | 9.34 | 9.77 | 9.70 | 10.11 | 9.54 |
| ILMN_1746712 | TSHR | -2.80 | -2.65 | -2.69 | -5.18 | 6.94 | 7.18 | 7.16 | 7.17 | 7.38 |
| ILMN_1738173 | METTL4 | -2.79 | -0.70 | -2.38 | -2.92 | 7.33 | 7.68 | 7.42 | 7.63 | 7.70 |
| ILMN_1810875 | SYNGR1 | -2.79 | -0.55 | -2.94 | -4.52 | 6.89 | 7.14 | 6.94 | 7.15 | 7.30 |
| ILMN_1682938 | ARF3 | -2.78 | -2.70 | -1.69 | -2.74 | 8.14 | 8.59 | 8.57 | 8.41 | 8.58 |
| ILMN_1674038 | CTSD | -2.78 | -2.02 | -2.45 | -4.57 | 7.64 | 7.84 | 7.78 | 7.81 | 7.96 |
| ILMN_1802905 | PIAS4 | -2.77 | -2.06 | -4.05 | -3.07 | 9.55 | 9.82 | 9.75 | 9.95 | 9.85 |
| ILMN_1773117 | BCOR | -2.76 | -5.11 | -4.93 | -4.38 | 10.26 | 10.46 | 10.64 | 10.62 | 10.58 |
| ILMN_1679809 | GSTP1 | -2.75 | -1.37 | -2.75 | -3.66 | 11.97 | 12.41 | 12.19 | 12.41 | 12.56 |
| ILMN_3304022 | LOC729102 | -2.74 | -2.62 | -0.54 | -3.26 | 11.49 | 11.79 | 11.78 | 11.55 | 11.85 |
| ILMN_1759766 | CTXN1 | -2.74 | -2.39 | -3.29 | -3.24 | 8.04 | 8.56 | 8.49 | 8.66 | 8.65 |
| ILMN_2045994 | SEPW1 | -2.74 | 0.21 | -0.27 | 4.11 | 8.87 | 9.14 | 8.85 | 8.90 | 8.47 |
| ILMN_3213176 | LOC728263 | -2.73 | -1.56 | 0.59 | -3.48 | 7.20 | 7.43 | 7.33 | 7.15 | 7.49 |
| ILMN_1730734 | TMEM205 | -2.72 | -3.29 | -2.27 | -5.95 | 9.84 | 10.16 | 10.23 | 10.11 | 10.54 |
| ILMN_3249110 | CSRNP2 | -2.72 | -2.89 | -2.51 | -4.35 | 7.28 | 7.54 | 7.56 | 7.52 | 7.70 |
| ILMN_1737394 | LMNA | -2.71 | -1.66 | -4.70 | -3.42 | 7.19 | 7.60 | 7.44 | 7.90 | 7.70 |
| ILMN_1796900 | NUDCD3 | -2.71 | -2.40 | -2.71 | -2.74 | 8.03 | 8.29 | 8.26 | 8.29 | 8.30 |
| ILMN_1670193 | ACY3 | -2.71 | -3.33 | -5.49 | -5.10 | 7.45 | 7.95 | 8.06 | 8.45 | 8.38 |
| ILMN_1687864 | POP5 | -2.71 | -0.94 | -1.27 | -6.52 | 8.21 | 8.59 | 8.34 | 8.39 | 9.13 |
| ILMN_1724376 | C2orf30 | -2.69 | -2.50 | -2.66 | -4.78 | 8.68 | 8.96 | 8.94 | 8.96 | 9.18 |
| ILMN_1741440 | SLC35A1 | -2.69 | -0.58 | -2.75 | -2.81 | 8.66 | 8.97 | 8.73 | 8.98 | 8.99 |
| ILMN_1880521 |  | -2.69 | -0.79 | -3.74 | 0.93 | 7.01 | 7.25 | 7.08 | 7.34 | 6.93 |
| ILMN_1770035 | NCOA5 | -2.69 | -2.78 | -3.18 | -4.88 | 8.90 | 9.17 | 9.18 | 9.22 | 9.39 |
| ILMN_1672004 | TOB1 | -2.67 | 0.24 | -3.25 | -4.97 | 7.47 | 7.73 | 7.45 | 7.79 | 7.95 |
| ILMN_1667966 | C1orf24 | -2.67 | -2.15 | -2.15 | -3.50 | 6.64 | 6.84 | 6.80 | 6.80 | 6.90 |
| ILMN_1727809 | STK35 | -2.66 | -2.57 | -2.49 | -3.27 | 7.33 | 7.69 | 7.68 | 7.67 | 7.78 |
| ILMN_1662618 | SQSTM1 | -2.66 | -1.53 | -0.89 | -3.74 | 9.42 | 9.78 | 9.63 | 9.54 | 9.93 |
| ILMN_1765520 | MTIF2 | -2.66 | -0.73 | -1.58 | -2.77 | 8.84 | 9.30 | 8.96 | 9.11 | 9.32 |
| ILMN_1711886 | ALG3 | -2.65 | -1.01 | -1.81 | -4.64 | 8.78 | 9.04 | 8.88 | 8.96 | 9.23 |
| ILMN_1746012 | MBD6 | -2.65 | -4.58 | -3.99 | -3.50 | 9.44 | 9.74 | 9.96 | 9.90 | 9.84 |
| ILMN_1651315 | HMG20B | -2.65 | -1.89 | -4.38 | -2.51 | 9.13 | 9.46 | 9.37 | 9.67 | 9.44 |
| ILMN_2368597 | SMG7 | -2.65 | -0.82 | -0.84 | -2.78 | 7.51 | 7.89 | 7.63 | 7.63 | 7.91 |
| ILMN_3245236 | FBRS | -2.63 | -1.08 | -1.82 | -2.90 | 8.41 | 8.73 | 8.54 | 8.63 | 8.76 |
| ILMN_1793770 | DNAJB6 | -2.63 | -2.11 | -3.56 | -7.19 | 8.93 | 9.27 | 9.20 | 9.39 | 9.86 |
| ILMN_1728645 | LOC649095 | -2.62 | -1.90 | -2.40 | -3.78 | 7.08 | 7.64 | 7.48 | 7.59 | 7.88 |
| ILMN_1756920 | ADAM15 | -2.62 | -3.30 | -2.90 | -7.88 | 9.10 | 9.38 | 9.45 | 9.41 | 9.94 |
| ILMN_2332250 | ACOT7 | -2.61 | -1.64 | -0.83 | -3.25 | 8.26 | 8.80 | 8.60 | 8.43 | 8.93 |
| ILMN_1811624 | THADA | -2.61 | -2.09 | -2.91 | -2.78 | 7.56 | 7.83 | 7.78 | 7.86 | 7.85 |
| ILMN_1730612 | DBNDD2 | -2.61 | -1.71 | -3.88 | -2.27 | 8.87 | 9.91 | 9.55 | 10.41 | 9.77 |
| ILMN_1661484 | ZBTB45 | -2.61 | -2.13 | -4.74 | -0.77 | 7.27 | 7.41 | 7.39 | 7.53 | 7.31 |
| ILMN_1747412 | DPP3 | -2.60 | -1.18 | -1.06 | -2.99 | 7.08 | 7.36 | 7.21 | 7.19 | 7.41 |
| ILMN_2269136 | AGAP3 | -2.60 | -1.87 | -2.35 | -4.32 | 7.45 | 7.79 | 7.70 | 7.76 | 8.02 |
| ILMN_1810559 | RHOQ | -2.59 | -3.61 | -5.01 | -4.27 | 10.05 | 10.40 | 10.54 | 10.73 | 10.63 |
| ILMN_1790518 | PHF16 | -2.59 | -1.24 | -1.35 | -3.88 | 9.23 | 9.56 | 9.39 | 9.40 | 9.73 |
| ILMN_1668743 | LOC728069 | -2.59 | -1.09 | -0.63 | -3.80 | 8.36 | 8.66 | 8.49 | 8.43 | 8.80 |
| ILMN_2371700 | UCHL5IP | -2.59 | -2.95 | -3.03 | -4.94 | 9.06 | 9.28 | 9.31 | 9.31 | 9.47 |
| ILMN_2192620 | DCAF15 | -2.58 | -3.98 | -4.34 | -3.15 | 8.59 | 8.75 | 8.84 | 8.87 | 8.79 |
| ILMN_1770733 | RIC8A | -2.58 | -2.38 | -3.19 | -6.74 | 9.62 | 9.88 | 9.86 | 9.94 | 10.29 |
| ILMN_2231020 | TMEM185B | -2.58 | -1.36 | -2.50 | -5.71 | 7.70 | 8.01 | 7.86 | 8.00 | 8.40 |
| ILMN_1673352 | IFITM2 | -2.58 | -1.37 | -3.99 | -3.49 | 10.33 | 11.04 | 10.71 | 11.43 | 11.29 |
| ILMN_2357015 | GRK6 | -2.57 | -1.49 | -2.89 | -4.06 | 6.90 | 7.09 | 7.01 | 7.11 | 7.20 |
| ILMN_2328029 | EXT2 | -2.57 | -1.27 | -1.90 | -4.54 | 7.85 | 8.08 | 7.96 | 8.02 | 8.25 |
| ILMN_1792092 | ZCCHC8 | -2.57 | -0.67 | -1.71 | -3.11 | 8.15 | 8.48 | 8.24 | 8.37 | 8.55 |
| ILMN_1771026 | GARS | -2.57 | -2.01 | -4.43 | -0.17 | 11.49 | 11.85 | 11.77 | 12.10 | 11.52 |
| ILMN_2246956 | BCL2 | -2.57 | -1.89 | -2.50 | -3.47 | 9.29 | 9.77 | 9.64 | 9.75 | 9.93 |
| ILMN_1726025 | ASXL1 | -2.54 | -1.31 | -0.70 | -3.30 | 8.21 | 8.65 | 8.44 | 8.33 | 8.79 |
| ILMN_3242211 | TMEM187 | -2.54 | -1.23 | -0.77 | -5.01 | 7.31 | 7.67 | 7.48 | 7.42 | 8.02 |
| ILMN_2069446 | SAFB2 | -2.53 | -0.62 | -1.29 | -3.12 | 9.94 | 10.17 | 10.00 | 10.06 | 10.23 |
| ILMN_1774196 | URM1 | -2.53 | -2.80 | -2.10 | -3.39 | 10.20 | 10.36 | 10.38 | 10.33 | 10.41 |
| ILMN_2308849 | MYADM | -2.53 | -1.14 | -4.46 | -1.39 | 9.92 | 10.63 | 10.24 | 11.18 | 10.31 |
| ILMN_1851547 |  | -2.53 | -2.37 | -3.58 | -4.28 | 6.92 | 7.19 | 7.18 | 7.31 | 7.38 |
| ILMN_1718207 | SETDB1 | -2.51 | -1.79 | -0.07 | -4.62 | 8.75 | 8.99 | 8.92 | 8.76 | 9.18 |
| ILMN_1795561 | CAMK1D | -2.50 | -1.82 | -4.28 | -0.52 | 7.22 | 7.43 | 7.38 | 7.58 | 7.27 |
| ILMN_2216582 | LYL1 | -2.48 | -2.50 | -4.06 | -3.06 | 10.55 | 11.08 | 11.08 | 11.41 | 11.20 |
| ILMN_1760143 | ADRM1 | -2.48 | -1.80 | -2.83 | -4.92 | 10.71 | 10.90 | 10.85 | 10.93 | 11.09 |
| ILMN_1752303 | YY1AP1 | -2.48 | -2.07 | -1.43 | -3.53 | 10.11 | 10.33 | 10.29 | 10.24 | 10.42 |
| ILMN_1759870 | LOC653066 | -2.48 | -3.27 | -0.47 | -6.03 | 7.58 | 7.75 | 7.81 | 7.61 | 8.01 |
| ILMN_2214098 | BIVM | -2.47 | -3.94 | -4.22 | -2.08 | 7.97 | 8.26 | 8.44 | 8.47 | 8.22 |
| ILMN_1791366 | RCOR2 | -2.47 | -2.55 | -3.93 | -1.26 | 8.25 | 8.55 | 8.56 | 8.72 | 8.40 |
| ILMN_1804929 | OXTR | -2.47 | -0.98 | -0.66 | -2.82 | 7.57 | 7.88 | 7.70 | 7.66 | 7.93 |
| ILMN_1705310 | VEZF1 | -2.47 | -1.26 | -1.51 | -2.84 | 9.49 | 9.76 | 9.63 | 9.65 | 9.80 |
| ILMN_1733859 | DCAF15 | -2.47 | -2.04 | -1.19 | -2.99 | 7.10 | 7.27 | 7.24 | 7.18 | 7.31 |
| ILMN_1654493 | LOC649169 | -2.47 | -1.26 | -1.17 | -3.24 | 7.95 | 8.21 | 8.08 | 8.07 | 8.30 |
| ILMN_1791097 | RSBN1 | -2.46 | -1.22 | -0.30 | -4.00 | 9.49 | 9.77 | 9.63 | 9.52 | 9.94 |
| ILMN_1698259 | TMEM100 | -2.46 | -0.75 | -5.37 | 0.48 | 9.39 | 9.77 | 9.51 | 10.21 | 9.32 |
| ILMN_1778144 | FLJ20489 | -2.45 | -2.29 | -2.47 | -5.61 | 7.34 | 7.64 | 7.62 | 7.65 | 8.04 |
| ILMN_1747556 | CDK9 | -2.45 | -2.29 | -1.32 | -5.85 | 8.92 | 9.12 | 9.11 | 9.03 | 9.40 |
| ILMN_1794643 | ZGPAT | -2.44 | -2.63 | -2.13 | -4.93 | 8.86 | 9.16 | 9.18 | 9.12 | 9.46 |
| ILMN_2352042 | MRPL4 | -2.43 | -2.03 | -1.77 | -5.58 | 8.07 | 8.38 | 8.33 | 8.30 | 8.78 |
| ILMN_1684183 | RAD9A | -2.42 | -3.22 | -1.54 | -4.36 | 7.28 | 7.46 | 7.52 | 7.39 | 7.61 |
| ILMN_1720858 | C6orf115 | -2.42 | -1.20 | -3.20 | 3.41 | 8.53 | 8.84 | 8.69 | 8.94 | 8.10 |
| ILMN_1651800 | GSTM4 | -2.42 | -0.39 | -1.36 | -3.00 | 6.80 | 7.01 | 6.84 | 6.92 | 7.06 |
| ILMN_2087646 | HLX | -2.41 | -4.07 | -5.78 | -5.46 | 7.75 | 8.02 | 8.21 | 8.41 | 8.37 |
| ILMN_1668822 | BATF | -2.40 | -0.72 | 1.32 | -3.65 | 7.55 | 7.75 | 7.61 | 7.44 | 7.86 |
| ILMN_1696749 | LMNA | -2.40 | -0.97 | -3.93 | -2.15 | 8.12 | 8.77 | 8.38 | 9.18 | 8.70 |
| ILMN_1728305 | PUM2 | -2.40 | -2.33 | -3.17 | -2.95 | 9.36 | 9.73 | 9.72 | 9.84 | 9.81 |
| ILMN_3297644 | TMEM214 | -2.40 | -3.78 | -3.65 | -5.36 | 7.35 | 7.55 | 7.66 | 7.65 | 7.79 |
| ILMN_1701477 | CCDC101 | -2.39 | -2.15 | -3.35 | -4.88 | 7.21 | 7.38 | 7.36 | 7.44 | 7.55 |
| ILMN_1745513 | MCAT | -2.39 | -1.82 | -4.05 | -2.88 | 7.77 | 7.98 | 7.93 | 8.13 | 8.02 |
| ILMN_1798172 | IPO4 | -2.39 | -0.70 | -1.91 | -3.34 | 8.34 | 8.55 | 8.40 | 8.51 | 8.64 |
| ILMN_1670272 | LRP10 | -2.38 | -1.54 | -4.02 | -2.85 | 9.04 | 9.25 | 9.18 | 9.40 | 9.30 |
| ILMN_1785618 | SMTN | -2.38 | -0.97 | -4.79 | 1.44 | 7.22 | 7.44 | 7.31 | 7.66 | 7.08 |
| ILMN_3236945 | PTPMT1 | -2.38 | -1.90 | -2.05 | -4.90 | 9.93 | 10.14 | 10.10 | 10.11 | 10.37 |
| ILMN_1772677 | CNOT4 | -2.37 | -0.29 | -0.90 | -2.92 | 8.11 | 8.41 | 8.14 | 8.22 | 8.48 |
| ILMN_1693836 | LOC653344 | -2.37 | -1.79 | -2.30 | 5.08 | 9.15 | 9.38 | 9.33 | 9.38 | 8.65 |
| ILMN_1692664 | PRR5 | -2.36 | -1.45 | -1.88 | -3.10 | 6.92 | 7.19 | 7.09 | 7.14 | 7.28 |
| ILMN_1772731 | HAGH | -2.36 | -2.94 | -5.06 | -3.22 | 7.80 | 8.03 | 8.09 | 8.29 | 8.11 |
| ILMN_2325978 | HDGF2 | -2.35 | -2.35 | -2.41 | -2.79 | 7.68 | 7.96 | 7.96 | 7.96 | 8.01 |
| ILMN_1717052 | STARD10 | -2.35 | -1.58 | -3.74 | -4.13 | 7.27 | 7.48 | 7.41 | 7.60 | 7.63 |
| ILMN_1652196 | CTAG1A | -2.35 | -1.31 | -2.55 | -5.24 | 7.69 | 7.93 | 7.82 | 7.95 | 8.21 |
| ILMN_2356838 | CEPT1 | -2.35 | -1.24 | -1.55 | -3.62 | 10.04 | 10.32 | 10.19 | 10.23 | 10.47 |
| ILMN_1673073 | WDR62 | -2.34 | -2.74 | -1.54 | -2.74 | 6.92 | 7.06 | 7.08 | 7.01 | 7.08 |
| ILMN_1712914 | SEMA6B | -2.34 | -1.29 | -3.35 | -2.92 | 6.90 | 7.06 | 6.99 | 7.13 | 7.10 |
| ILMN_2128770 | CDR2L | -2.34 | -2.31 | -3.05 | -4.65 | 7.31 | 7.57 | 7.57 | 7.65 | 7.83 |
| ILMN_2408102 | TFIP11 | -2.33 | -0.61 | -1.26 | -4.17 | 8.61 | 8.81 | 8.66 | 8.72 | 8.97 |
| ILMN_1707070 | PCOLCE | -2.33 | -0.92 | -2.91 | -3.68 | 7.13 | 7.39 | 7.23 | 7.45 | 7.53 |
| ILMN_1764165 | TRIM65 | -2.33 | -1.23 | -2.74 | -2.86 | 6.79 | 6.98 | 6.89 | 7.01 | 7.02 |
| ILMN_1693650 | FES | -2.33 | -2.07 | -3.72 | -3.13 | 10.06 | 10.39 | 10.35 | 10.58 | 10.50 |
| ILMN_1667561 | IFRD1 | -2.33 | -0.15 | -0.53 | -3.20 | 8.12 | 8.44 | 8.14 | 8.19 | 8.56 |
| ILMN_1810992 | CAD | -2.32 | -4.85 | -4.75 | -7.81 | 8.34 | 8.57 | 8.82 | 8.81 | 9.11 |
| ILMN_1697998 | FAM54A | -2.32 | -0.23 | -0.13 | -3.03 | 7.37 | 7.61 | 7.39 | 7.38 | 7.68 |
| ILMN_1660498 | RPS27 | -2.32 | -2.58 | -2.24 | -3.31 | 13.90 | 14.11 | 14.13 | 14.10 | 14.19 |
| ILMN_1655748 | ZNF323 | -2.31 | 2.36 | 1.48 | 2.88 | 7.78 | 7.95 | 7.60 | 7.66 | 7.56 |
| ILMN_1693766 | CEP135 | -2.31 | -0.89 | -0.59 | 2.74 | 8.84 | 9.09 | 8.94 | 8.90 | 8.54 |
| ILMN_2347592 | NMB | -2.31 | -0.31 | -3.08 | -4.89 | 8.11 | 8.31 | 8.13 | 8.37 | 8.53 |
| ILMN_1719286 | CTSA | -2.31 | -1.15 | -1.95 | -7.16 | 7.11 | 7.31 | 7.21 | 7.28 | 7.71 |
| ILMN_1799860 | PIGM | -2.30 | -0.90 | -0.95 | -2.94 | 8.67 | 8.97 | 8.78 | 8.79 | 9.05 |
| ILMN_1810334 | COMMD7 | -2.30 | -1.96 | -1.59 | -2.73 | 10.32 | 10.68 | 10.62 | 10.57 | 10.74 |
| ILMN_2157435 | DYNLRB1 | -2.30 | -1.44 | -1.30 | -3.59 | 9.28 | 9.49 | 9.41 | 9.40 | 9.60 |
| ILMN_2115218 | ANKRD10 | -2.30 | -2.38 | -3.23 | -3.10 | 8.98 | 9.16 | 9.17 | 9.24 | 9.23 |
| ILMN_1761259 | EXT2 | -2.29 | -1.62 | -3.23 | -4.62 | 7.88 | 8.12 | 8.05 | 8.22 | 8.36 |
| ILMN_2387090 | CGGBP1 | -2.29 | 3.69 | 0.40 | 3.33 | 8.68 | 8.85 | 8.41 | 8.65 | 8.44 |
| ILMN_1681154 | ZBTB39 | -2.29 | -0.59 | -2.57 | -2.81 | 7.62 | 7.84 | 7.67 | 7.86 | 7.89 |
| ILMN_2235283 | MAPK1 | -2.29 | -0.84 | -4.05 | 0.15 | 8.43 | 8.67 | 8.52 | 8.86 | 8.41 |
| ILMN_1695962 | SLC12A9 | -2.29 | -1.80 | -1.63 | -5.21 | 8.43 | 8.70 | 8.64 | 8.62 | 9.04 |
| ILMN_2320964 | ADAR | -2.29 | -1.99 | -1.38 | -2.84 | 9.50 | 9.80 | 9.76 | 9.68 | 9.88 |
| ILMN_3181296 | LOC100130623 | -2.29 | -3.35 | -2.63 | -4.85 | 7.39 | 7.61 | 7.71 | 7.64 | 7.86 |
| ILMN_1787879 | ARL2 | -2.28 | -2.67 | -3.10 | -4.32 | 10.55 | 10.89 | 10.94 | 11.01 | 11.19 |
| ILMN_1690708 | SPTBN1 | -2.28 | -1.12 | -3.00 | -2.84 | 7.00 | 7.34 | 7.17 | 7.45 | 7.42 |
| ILMN_1706413 | C1orf66 | -2.28 | -2.28 | -2.89 | -4.35 | 7.41 | 7.63 | 7.63 | 7.69 | 7.83 |
| ILMN_1756992 | MUC1 | -2.28 | -1.37 | -1.49 | -6.20 | 6.98 | 7.18 | 7.10 | 7.11 | 7.51 |
| ILMN_1695354 | BMF | -2.27 | -3.27 | -3.91 | -2.61 | 7.13 | 7.51 | 7.67 | 7.78 | 7.56 |
| ILMN_1682459 | TUBB4 | -2.27 | -1.32 | -4.06 | -3.61 | 8.21 | 8.50 | 8.38 | 8.72 | 8.67 |
| ILMN_3247906 | RNF114 | -2.26 | -0.58 | -1.16 | -5.63 | 8.82 | 8.96 | 8.86 | 8.89 | 9.18 |
| ILMN_1691117 | DNTTIP1 | -2.26 | -1.31 | -1.59 | -3.04 | 9.44 | 9.71 | 9.60 | 9.63 | 9.81 |
| ILMN_1655952 | FAM39E | -2.26 | -2.72 | -2.57 | -3.49 | 9.65 | 9.94 | 10.00 | 9.98 | 10.10 |
| ILMN_1815656 | SERINC3 | -2.25 | -1.74 | -2.69 | -4.51 | 8.52 | 8.79 | 8.73 | 8.85 | 9.07 |
| ILMN_1679428 | CHIC2 | -2.25 | 0.07 | -0.79 | 4.10 | 9.93 | 10.15 | 9.93 | 10.01 | 9.53 |
| ILMN_1800425 | SLC9A1 | -2.24 | -1.59 | -1.56 | -2.92 | 7.60 | 7.83 | 7.77 | 7.76 | 7.90 |
| ILMN_1715968 | MLL4 | -2.24 | -1.67 | -1.49 | -2.99 | 7.38 | 7.59 | 7.54 | 7.52 | 7.66 |
| ILMN_2392635 | ABCF1 | -2.24 | -2.86 | -0.10 | -4.41 | 9.29 | 9.50 | 9.56 | 9.30 | 9.71 |
| ILMN_1806037 | TK1 | -2.24 | -1.33 | -3.62 | -3.95 | 8.91 | 9.18 | 9.07 | 9.34 | 9.38 |
| ILMN_3210491 | LOC389049 | -2.23 | -1.70 | -2.60 | -3.80 | 7.76 | 8.11 | 8.03 | 8.17 | 8.35 |
| ILMN_1808636 | LARP1B | -2.23 | -0.46 | -1.41 | 3.05 | 7.20 | 7.36 | 7.23 | 7.30 | 6.97 |
| ILMN_2061310 | ZNF280C | -2.22 | -0.98 | -1.04 | 3.05 | 8.95 | 9.20 | 9.06 | 9.07 | 8.61 |
| ILMN_1671583 | MKRN1 | -2.22 | -2.89 | -0.63 | -2.89 | 10.17 | 10.35 | 10.40 | 10.22 | 10.40 |
| ILMN_2324672 | USF2 | -2.22 | -2.25 | -1.82 | -3.65 | 7.31 | 7.55 | 7.56 | 7.51 | 7.71 |
| ILMN_3209973 | LOC646044 | -2.22 | -3.46 | -4.11 | -5.05 | 7.57 | 7.80 | 7.92 | 7.99 | 8.09 |
| ILMN_2350574 | MYADM | -2.21 | -1.35 | -4.62 | -0.78 | 7.61 | 8.14 | 7.93 | 8.73 | 7.80 |
| ILMN_1680937 | HIST1H2BC | -2.21 | -0.31 | -1.55 | -2.80 | 7.43 | 7.72 | 7.47 | 7.63 | 7.80 |
| ILMN_3247139 | C17orf96 | -2.20 | -2.62 | -2.55 | -4.37 | 8.04 | 8.25 | 8.29 | 8.28 | 8.45 |
| ILMN_2108823 | FZD7 | -2.20 | -0.44 | -2.69 | -3.97 | 7.28 | 7.43 | 7.31 | 7.46 | 7.55 |
| ILMN_2139100 | SHISA5 | -2.20 | -3.05 | -2.28 | -2.91 | 9.37 | 9.63 | 9.73 | 9.64 | 9.71 |
| ILMN_3237209 | LOC642661 | -2.20 | -1.47 | -1.91 | -3.54 | 7.15 | 7.33 | 7.27 | 7.31 | 7.44 |
| ILMN_3250201 | CNBP | -2.20 | -0.46 | 0.46 | -3.30 | 7.49 | 7.65 | 7.52 | 7.45 | 7.73 |
| ILMN_1801119 | BCL2 | -2.20 | -2.52 | -3.15 | -4.74 | 8.88 | 9.17 | 9.21 | 9.29 | 9.50 |
| ILMN_1664012 | CANT1 | -2.19 | -0.34 | -1.35 | -3.51 | 8.43 | 8.64 | 8.46 | 8.56 | 8.77 |
| ILMN_1702763 | ZMYM1 | -2.19 | 1.27 | -0.24 | -4.42 | 7.58 | 7.79 | 7.45 | 7.60 | 8.01 |
| ILMN_1770356 | POLRMT | -2.18 | -4.36 | -2.99 | -8.00 | 8.73 | 8.92 | 9.11 | 8.99 | 9.43 |
| ILMN_1703866 | SUPT5H | -2.17 | -3.09 | -2.54 | -5.82 | 8.50 | 8.69 | 8.78 | 8.73 | 9.02 |
| ILMN_1715715 | CEBPA | -2.17 | -2.48 | -3.20 | -5.13 | 7.07 | 7.28 | 7.31 | 7.38 | 7.56 |
| ILMN_2387636 | ITGB4BP | -2.17 | -2.08 | -2.34 | -4.77 | 10.57 | 10.96 | 10.94 | 10.99 | 11.42 |
| ILMN_1684789 | CCDC101 | -2.17 | -0.77 | -2.31 | -3.97 | 8.55 | 8.75 | 8.62 | 8.76 | 8.91 |
| ILMN_3244117 | STMN3 | -2.16 | -2.37 | -2.31 | -3.84 | 10.94 | 11.43 | 11.48 | 11.47 | 11.81 |
| ILMN_2130411 | KDELR1 | -2.16 | -2.72 | -3.39 | -5.07 | 8.18 | 8.37 | 8.42 | 8.48 | 8.63 |
| ILMN_2400500 | LASS2 | -2.15 | -2.72 | -2.30 | -5.35 | 8.76 | 9.00 | 9.07 | 9.02 | 9.36 |
| ILMN_1804207 | MRPL4 | -2.15 | -1.09 | -1.33 | -2.76 | 7.66 | 7.87 | 7.77 | 7.79 | 7.93 |
| ILMN_1668134 | GSTM1 | -2.15 | -1.06 | -1.93 | -3.75 | 6.86 | 7.08 | 6.97 | 7.06 | 7.25 |
| ILMN_2330112 | CNIH | -2.15 | -2.18 | -1.77 | -3.92 | 9.65 | 9.91 | 9.92 | 9.87 | 10.13 |
| ILMN_1768870 | CAPZA2 | -2.15 | -2.11 | -0.44 | -5.81 | 9.81 | 9.99 | 9.98 | 9.84 | 10.29 |
| ILMN_2387784 | DEAF1 | -2.15 | -1.86 | -0.89 | -2.91 | 6.69 | 6.87 | 6.85 | 6.77 | 6.93 |
| ILMN_1671766 | F12 | -2.14 | -0.85 | -4.09 | -7.29 | 7.34 | 7.74 | 7.50 | 8.10 | 8.69 |
| ILMN_1711450 | TH1L | -2.14 | -1.07 | -1.96 | -4.31 | 10.36 | 10.60 | 10.48 | 10.58 | 10.84 |
| ILMN_1726108 | LASS2 | -2.14 | -3.58 | -2.70 | -6.08 | 8.60 | 8.82 | 8.96 | 8.87 | 9.22 |
| ILMN_1678052 | C19orf24 | -2.14 | -2.42 | -3.36 | -4.49 | 8.34 | 8.57 | 8.60 | 8.70 | 8.82 |
| ILMN_1790228 | FURIN | -2.14 | -2.28 | -2.98 | -4.47 | 6.74 | 6.89 | 6.90 | 6.94 | 7.04 |
| ILMN_1768595 | DLG4 | -2.13 | -2.29 | -2.17 | -2.90 | 6.98 | 7.15 | 7.17 | 7.16 | 7.22 |
| ILMN_3209117 | LOC644214 | -2.13 | -0.77 | -0.80 | -3.65 | 9.18 | 9.45 | 9.28 | 9.28 | 9.64 |
| ILMN_1754865 | LOC400455 | -2.12 | -3.48 | -2.12 | -4.93 | 9.10 | 9.26 | 9.36 | 9.26 | 9.46 |
| ILMN_1758543 | CNIH | -2.12 | -0.22 | -1.01 | -3.15 | 9.47 | 9.73 | 9.50 | 9.60 | 9.86 |
| ILMN_1780756 | RBM23 | -2.12 | -0.88 | -0.52 | 3.29 | 10.39 | 10.61 | 10.48 | 10.45 | 10.06 |
| ILMN_1740395 | RAVER1 | -2.12 | -2.75 | -3.94 | -2.96 | 8.64 | 8.81 | 8.86 | 8.95 | 8.88 |
| ILMN_1695827 | PPP1CA | -2.12 | 0.08 | -1.57 | -2.97 | 9.75 | 10.02 | 9.74 | 9.95 | 10.13 |
| ILMN_1802951 | CDCA1 | -2.11 | -0.68 | -1.55 | -2.96 | 6.90 | 7.20 | 6.99 | 7.12 | 7.32 |
| ILMN_1687410 | OSBPL11 | -2.11 | 0.39 | -0.46 | 4.29 | 8.93 | 9.13 | 8.89 | 8.97 | 8.52 |
| ILMN_1811328 | DPP7 | -2.11 | -1.14 | -0.45 | -3.00 | 7.80 | 8.11 | 7.97 | 7.87 | 8.25 |
| ILMN_2206413 | CTAG1A | -2.10 | -1.39 | -2.40 | -4.58 | 7.54 | 7.83 | 7.73 | 7.87 | 8.17 |
| ILMN_3229467 | LOC729217 | -2.10 | -0.33 | -1.31 | -3.93 | 8.64 | 8.96 | 8.69 | 8.84 | 9.24 |
| ILMN_2390162 | PHF11 | -2.10 | -0.51 | -1.61 | 3.28 | 10.29 | 10.64 | 10.38 | 10.56 | 9.76 |
| ILMN_1670398 | BCR | -2.08 | -4.21 | -2.36 | -3.84 | 7.02 | 7.17 | 7.32 | 7.19 | 7.30 |
| ILMN_2120022 | ARL5B | -2.08 | 0.48 | -1.76 | 2.84 | 8.19 | 8.36 | 8.15 | 8.34 | 7.95 |
| ILMN_1700822 | DPP3 | -2.07 | -1.18 | -2.29 | -4.40 | 8.04 | 8.20 | 8.13 | 8.22 | 8.39 |
| ILMN_2209163 | CHD6 | -2.07 | -1.46 | -2.81 | -2.72 | 8.50 | 8.67 | 8.62 | 8.73 | 8.72 |
| ILMN_1659524 | C6orf66 | -2.07 | 1.34 | -0.50 | -3.59 | 7.88 | 8.12 | 7.73 | 7.94 | 8.29 |
| ILMN_1672443 | QDPR | -2.07 | -1.97 | -2.27 | 2.77 | 9.57 | 9.78 | 9.77 | 9.80 | 9.29 |
| ILMN_2367707 | PKN1 | -2.07 | -2.37 | -4.44 | -3.28 | 7.75 | 7.89 | 7.91 | 8.04 | 7.97 |
| ILMN_1699226 | UBR4 | -2.07 | -0.76 | -0.59 | -3.91 | 8.87 | 9.15 | 8.98 | 8.95 | 9.40 |
| ILMN_2061043 | CD48 | -2.06 | 0.16 | -1.72 | -7.52 | 9.77 | 10.03 | 9.75 | 9.99 | 10.73 |
| ILMN_1658702 | HIST1H2BJ | -2.06 | -0.92 | -2.02 | -3.03 | 8.46 | 9.00 | 8.70 | 8.99 | 9.25 |
| ILMN_1703477 | ARHGEF2 | -2.06 | -0.55 | -1.79 | -3.13 | 11.53 | 11.89 | 11.62 | 11.84 | 12.07 |
| ILMN_1771462 | RPL36 | -2.06 | -0.71 | -1.91 | -2.92 | 7.18 | 7.40 | 7.25 | 7.38 | 7.49 |
| ILMN_1701169 | HP1BP3 | -2.06 | 0.50 | -0.73 | -3.15 | 7.27 | 7.47 | 7.22 | 7.34 | 7.58 |
| ILMN_1808226 | RGS16 | -2.06 | -1.55 | -2.65 | -4.37 | 7.34 | 7.64 | 7.56 | 7.72 | 7.97 |
| ILMN_1747241 | IWS1 | -2.05 | -0.41 | -0.24 | -2.80 | 8.99 | 9.27 | 9.05 | 9.02 | 9.38 |
| ILMN_1746565 | CD6 | -2.05 | -0.41 | -1.14 | -7.73 | 6.73 | 6.88 | 6.76 | 6.82 | 7.30 |
| ILMN_1750130 | GSPT1 | -2.04 | 1.50 | -1.08 | 2.92 | 12.44 | 12.66 | 12.27 | 12.56 | 12.11 |
| ILMN_1672496 | DNAJA1 | -2.04 | 2.40 | 1.65 | 3.85 | 12.48 | 12.73 | 12.20 | 12.29 | 12.02 |
| ILMN_2385178 | MIB2 | -2.04 | -1.07 | -1.54 | -4.62 | 8.87 | 9.31 | 9.10 | 9.20 | 9.86 |
| ILMN_1800447 | PHKB | -2.03 | -0.07 | -0.42 | 4.65 | 9.33 | 9.62 | 9.34 | 9.39 | 8.66 |
| ILMN_1733991 | UBL7 | -2.03 | 1.27 | 0.23 | 2.91 | 8.55 | 8.73 | 8.44 | 8.53 | 8.30 |
| ILMN_1766125 | LONP1 | -2.03 | -2.66 | -2.10 | -4.09 | 10.12 | 10.32 | 10.39 | 10.33 | 10.53 |
| ILMN_1681802 | GRK6 | -2.03 | -1.58 | -3.35 | -3.16 | 6.93 | 7.14 | 7.09 | 7.28 | 7.26 |
| ILMN_1676980 | MTSS1 | -2.02 | -2.25 | -1.64 | -3.01 | 7.35 | 7.76 | 7.81 | 7.68 | 7.97 |
| ILMN_1711270 | SFRS14 | -2.02 | -2.27 | -0.98 | -4.11 | 9.61 | 9.83 | 9.85 | 9.71 | 10.05 |
| ILMN_1722218 | MBOAT7 | -2.02 | -0.37 | -1.60 | -3.30 | 7.12 | 7.25 | 7.15 | 7.22 | 7.33 |
| ILMN_1794085 | SAPS1 | -2.01 | -1.90 | -3.28 | -5.78 | 7.38 | 7.56 | 7.55 | 7.68 | 7.90 |
| ILMN_1654735 | SLCO3A1 | -2.01 | -2.61 | -3.95 | -5.20 | 6.92 | 7.20 | 7.29 | 7.47 | 7.65 |
| ILMN_1772605 | FRS3 | -2.01 | -2.77 | -1.73 | -3.09 | 7.74 | 7.91 | 7.97 | 7.89 | 8.00 |
| ILMN_1752728 | FUCA1 | -2.01 | -0.03 | -0.20 | -4.44 | 9.01 | 9.28 | 9.01 | 9.04 | 9.60 |
| ILMN_2248725 | TYSND1 | -2.00 | -1.36 | -1.85 | -3.52 | 6.80 | 6.98 | 6.92 | 6.96 | 7.11 |
| ILMN_1654001 | SMC6 | -2.00 | -1.63 | -2.49 | -2.84 | 8.30 | 8.57 | 8.52 | 8.64 | 8.69 |
| ILMN_1746408 | MIDN | -1.99 | -3.99 | -5.52 | -4.59 | 8.79 | 8.99 | 9.19 | 9.34 | 9.25 |
| ILMN_1791770 | SMARCC2 | -1.99 | -2.05 | -2.11 | -3.29 | 7.06 | 7.28 | 7.29 | 7.30 | 7.43 |
| ILMN_2073289 | MTSS1 | -1.99 | -3.38 | -2.69 | -3.38 | 9.35 | 9.83 | 10.17 | 10.00 | 10.17 |
| ILMN_1741942 | STX16 | -1.99 | -3.21 | -2.75 | -4.88 | 9.27 | 9.55 | 9.72 | 9.65 | 9.95 |
| ILMN_1711828 | ANKRD10 | -1.98 | -3.01 | -4.12 | -3.71 | 8.30 | 8.48 | 8.58 | 8.68 | 8.64 |
| ILMN_1729161 | NOTCH1 | -1.98 | -0.13 | -1.50 | -2.73 | 7.17 | 7.38 | 7.19 | 7.33 | 7.45 |
| ILMN_1664750 | TMBIM4 | -1.98 | -1.00 | -1.60 | -4.58 | 10.57 | 10.82 | 10.70 | 10.77 | 11.16 |
| ILMN_1665235 | CRTAP | -1.98 | -1.28 | -1.45 | -2.90 | 6.69 | 6.93 | 6.85 | 6.87 | 7.04 |
| ILMN_2135756 | HSF2BP | -1.98 | -2.09 | -2.59 | -9.71 | 6.84 | 7.01 | 7.02 | 7.06 | 7.69 |
| ILMN_1739876 | RAB3GAP1 | -1.98 | -2.36 | -1.98 | -3.91 | 9.56 | 9.70 | 9.73 | 9.70 | 9.84 |
| ILMN_2383484 | C19orf48 | -1.98 | -3.58 | -1.70 | -5.10 | 7.80 | 8.01 | 8.19 | 7.98 | 8.35 |
| ILMN_1745395 | CTCFL | -1.97 | -1.68 | -3.14 | -3.97 | 10.70 | 11.01 | 10.97 | 11.20 | 11.33 |
| ILMN_1898394 |  | -1.97 | -1.26 | -1.21 | -2.78 | 6.70 | 6.83 | 6.79 | 6.78 | 6.89 |
| ILMN_3208014 | LOC100131866 | -1.97 | 1.05 | 0.25 | 2.82 | 10.68 | 10.94 | 10.54 | 10.64 | 10.30 |
| ILMN_3251728 | MTMR10 | -1.96 | -1.44 | -0.07 | 3.80 | 9.09 | 9.29 | 9.24 | 9.10 | 8.71 |
| ILMN_3287244 | LOC728138 | -1.96 | -1.65 | -3.08 | -4.07 | 9.50 | 9.75 | 9.71 | 9.89 | 10.02 |
| ILMN_1722309 | ENDOG | -1.96 | -1.62 | -3.24 | -5.64 | 8.31 | 8.54 | 8.50 | 8.69 | 8.98 |
| ILMN_1739792 | RHOG | -1.95 | -1.04 | -2.15 | -4.00 | 9.59 | 9.92 | 9.76 | 9.95 | 10.27 |
| ILMN_3242428 | LOC100134624 | -1.95 | -1.21 | -0.92 | -3.33 | 6.74 | 6.85 | 6.81 | 6.79 | 6.93 |
| ILMN_2364357 | RPS6KB2 | -1.95 | -2.45 | -2.37 | -4.21 | 10.73 | 10.97 | 11.04 | 11.03 | 11.26 |
| ILMN_3249366 | JMJD8 | -1.95 | -2.89 | -3.97 | -2.65 | 9.63 | 9.81 | 9.90 | 10.01 | 9.88 |
| ILMN_1704286 | FXYD5 | -1.94 | -1.16 | -2.38 | -4.83 | 8.45 | 8.69 | 8.59 | 8.74 | 9.05 |
| ILMN_1737344 | DDX41 | -1.94 | -3.17 | -1.15 | -3.70 | 8.97 | 9.11 | 9.21 | 9.05 | 9.25 |
| ILMN_1738099 | C2orf34 | -1.94 | -3.11 | -4.04 | 0.05 | 7.95 | 8.19 | 8.33 | 8.44 | 7.94 |
| ILMN_1739345 | C11orf48 | -1.94 | -0.59 | -0.44 | -3.55 | 9.93 | 10.15 | 9.99 | 9.98 | 10.33 |
| ILMN_1665554 | BRF2 | -1.93 | -0.62 | -1.89 | -3.59 | 7.58 | 7.89 | 7.68 | 7.88 | 8.15 |
| ILMN_1669424 | LOC646531 | -1.93 | -1.83 | -1.97 | -3.55 | 11.51 | 11.69 | 11.68 | 11.69 | 11.84 |
| ILMN_1759495 | XPO5 | -1.93 | -0.83 | -1.98 | -2.86 | 9.57 | 9.82 | 9.68 | 9.82 | 9.94 |
| ILMN_2089329 | SPRY2 | -1.93 | -1.80 | -3.77 | 0.34 | 8.18 | 8.57 | 8.54 | 8.95 | 8.11 |
| ILMN_3240365 | MNX1 | -1.92 | -3.63 | -4.96 | -3.51 | 7.63 | 7.78 | 7.92 | 8.02 | 7.91 |
| ILMN_1758311 | NET1 | -1.92 | 0.51 | -0.80 | 3.23 | 9.99 | 10.17 | 9.95 | 10.07 | 9.70 |
| ILMN_1769135 | DPP7 | -1.92 | -0.96 | -1.10 | -4.39 | 7.55 | 7.83 | 7.69 | 7.71 | 8.19 |
| ILMN_2320336 | CLK3 | -1.92 | -2.26 | -1.95 | -2.88 | 8.55 | 8.74 | 8.77 | 8.74 | 8.83 |
| ILMN_1728168 | C20orf45 | -1.91 | 0.18 | -0.31 | -3.34 | 8.60 | 8.85 | 8.58 | 8.64 | 9.03 |
| ILMN_1654571 | FCHO1 | -1.91 | -2.36 | -2.93 | -5.24 | 7.17 | 7.33 | 7.36 | 7.41 | 7.60 |
| ILMN_1673753 | LOC642033 | -1.91 | -0.60 | -1.52 | -2.86 | 7.21 | 7.39 | 7.27 | 7.36 | 7.48 |
| ILMN_1702020 | PLEKHG4B | -1.90 | -1.13 | -1.33 | -4.97 | 6.49 | 6.61 | 6.56 | 6.58 | 6.81 |
| ILMN_2414014 | RBM10 | -1.89 | -2.39 | -2.39 | -2.88 | 11.34 | 11.52 | 11.57 | 11.57 | 11.61 |
| ILMN_2156982 | IMP4 | -1.89 | -2.44 | -2.50 | -7.29 | 11.55 | 11.77 | 11.83 | 11.84 | 12.39 |
| ILMN_1774375 | LOC284422 | -1.89 | -2.22 | -2.36 | -3.45 | 6.62 | 6.75 | 6.77 | 6.78 | 6.86 |
| ILMN_1714165 | ST3GAL2 | -1.89 | -0.59 | 0.56 | 3.15 | 7.45 | 7.62 | 7.50 | 7.40 | 7.16 |
| ILMN_1781999 | ABCF2 | -1.89 | -1.56 | -1.30 | -2.86 | 8.15 | 8.54 | 8.47 | 8.42 | 8.73 |
| ILMN_1674009 | TKTL1 | -1.88 | -0.17 | -0.71 | -4.72 | 7.13 | 7.42 | 7.16 | 7.24 | 7.86 |
| ILMN_1670638 | PITPNC1 | -1.88 | -1.60 | -3.28 | -2.95 | 7.28 | 7.55 | 7.51 | 7.75 | 7.71 |
| ILMN_2212354 | WDR46 | -1.88 | -2.20 | -1.68 | -2.88 | 9.02 | 9.18 | 9.21 | 9.16 | 9.26 |
| ILMN_1724148 | ORAI1 | -1.88 | -1.62 | -2.22 | -3.39 | 7.18 | 7.35 | 7.32 | 7.38 | 7.48 |
| ILMN_1675797 | EPDR1 | -1.88 | -1.94 | -4.15 | -4.06 | 9.23 | 9.45 | 9.46 | 9.73 | 9.72 |
| ILMN_1797684 | PDCD2 | -1.88 | -1.29 | -1.76 | -3.20 | 9.30 | 9.51 | 9.45 | 9.50 | 9.66 |
| ILMN_1795089 | RASAL3 | -1.88 | -2.18 | -1.83 | -4.19 | 8.13 | 8.27 | 8.29 | 8.27 | 8.45 |
| ILMN_1683859 | SLC7A1 | -1.87 | -3.23 | -3.35 | -3.09 | 9.68 | 9.99 | 10.22 | 10.24 | 10.19 |
| ILMN_2308338 | BMF | -1.87 | -2.22 | -3.49 | -3.22 | 7.07 | 7.32 | 7.36 | 7.53 | 7.50 |
| ILMN_1801421 | EMD | -1.87 | -2.91 | -3.52 | -3.30 | 9.22 | 9.45 | 9.58 | 9.65 | 9.62 |
| ILMN_2411963 | RBM39 | -1.87 | -0.85 | -3.12 | -3.26 | 8.80 | 8.98 | 8.88 | 9.11 | 9.12 |
| ILMN_1661337 | SRM | -1.86 | -1.24 | -0.53 | -3.24 | 9.61 | 9.94 | 9.83 | 9.70 | 10.18 |
| ILMN_1690268 | HNRPUL1 | -1.86 | -3.10 | -2.35 | -2.95 | 10.52 | 10.74 | 10.88 | 10.80 | 10.87 |
| ILMN_1741054 | SLC5A6 | -1.86 | -1.71 | -1.76 | -3.39 | 8.97 | 9.32 | 9.29 | 9.30 | 9.61 |
| ILMN_1728002 | LOC653387 | -1.86 | -1.57 | -2.42 | -3.72 | 7.39 | 7.58 | 7.55 | 7.63 | 7.77 |
| ILMN_1727617 | XRN2 | -1.86 | -1.80 | -0.83 | -3.58 | 8.53 | 8.75 | 8.74 | 8.63 | 8.95 |
| ILMN_1795893 | TMEM167B | -1.86 | 0.00 | 0.11 | -3.19 | 8.15 | 8.33 | 8.15 | 8.14 | 8.45 |
| ILMN_2166506 | XRCC6 | -1.86 | -2.13 | -0.50 | -2.97 | 11.24 | 11.46 | 11.49 | 11.30 | 11.59 |
| ILMN_1784737 | S1PR4 | -1.85 | -1.70 | -2.57 | -5.02 | 8.59 | 8.92 | 8.89 | 9.05 | 9.49 |
| ILMN_3268564 | C19orf60 | -1.85 | -3.92 | -3.66 | -3.76 | 7.83 | 8.02 | 8.23 | 8.21 | 8.22 |
| ILMN_1668345 | OAF | -1.84 | -2.09 | -3.59 | -5.22 | 7.56 | 7.92 | 7.97 | 8.26 | 8.57 |
| ILMN_1792305 | ZNF318 | -1.84 | -2.29 | -2.72 | -2.95 | 9.23 | 9.53 | 9.60 | 9.67 | 9.71 |
| ILMN_1748883 | CDKN2D | -1.84 | -0.39 | -1.97 | -3.45 | 8.35 | 8.65 | 8.41 | 8.67 | 8.91 |
| ILMN_1737163 | SH3BGRL3 | -1.84 | -3.12 | -4.14 | -4.35 | 8.93 | 9.14 | 9.29 | 9.41 | 9.43 |
| ILMN_1772189 | ABCD1 | -1.83 | -2.18 | -1.59 | -4.93 | 6.89 | 7.06 | 7.10 | 7.04 | 7.36 |
| ILMN_1659227 | CD79A | -1.83 | -1.55 | -2.39 | -5.57 | 8.41 | 8.81 | 8.75 | 8.94 | 9.63 |
| ILMN_1719165 | BRMS1 | -1.82 | -1.63 | -1.94 | -4.73 | 7.72 | 7.94 | 7.91 | 7.95 | 8.29 |
| ILMN_1721704 | FNTA | -1.82 | -1.48 | -1.22 | -3.45 | 9.21 | 9.42 | 9.38 | 9.35 | 9.61 |
| ILMN_1652525 | FAM125B | -1.82 | -3.35 | -4.08 | -2.11 | 7.74 | 7.95 | 8.13 | 8.21 | 7.99 |
| ILMN_1690839 | PPAPDC3 | -1.82 | -2.18 | -4.35 | -5.37 | 7.08 | 7.33 | 7.38 | 7.68 | 7.82 |
| ILMN_1703327 | PSTPIP1 | -1.82 | -1.33 | -1.68 | -3.05 | 7.01 | 7.15 | 7.11 | 7.14 | 7.24 |
| ILMN_1789112 | TMEM145 | -1.81 | -3.92 | -3.33 | -4.70 | 8.19 | 8.47 | 8.79 | 8.70 | 8.90 |
| ILMN_1699334 | AATK | -1.80 | -1.28 | -1.80 | -3.09 | 6.94 | 7.09 | 7.04 | 7.09 | 7.19 |
| ILMN_1749006 | RCSD1 | -1.80 | -1.82 | -1.13 | -2.91 | 9.47 | 9.78 | 9.78 | 9.66 | 9.97 |
| ILMN_1780170 | APOD | -1.80 | -0.20 | -0.78 | -4.46 | 6.52 | 6.70 | 6.54 | 6.60 | 6.96 |
| ILMN_1814971 | TCF25 | -1.80 | -1.10 | -1.65 | 3.83 | 10.11 | 10.42 | 10.30 | 10.40 | 9.43 |
| ILMN_1734895 | SFT2D1 | -1.80 | 0.34 | -2.98 | -2.81 | 10.21 | 10.35 | 10.18 | 10.45 | 10.43 |
| ILMN_1772370 | ARHGEF1 | -1.80 | -2.05 | -1.80 | -2.95 | 7.67 | 7.86 | 7.89 | 7.86 | 7.98 |
| ILMN_1802397 | GNA11 | -1.80 | -3.81 | -4.47 | -3.01 | 7.15 | 7.33 | 7.52 | 7.58 | 7.44 |
| ILMN_1710543 | SLC39A3 | -1.80 | -0.62 | -2.24 | -3.89 | 7.43 | 7.75 | 7.54 | 7.83 | 8.13 |
| ILMN_1653429 | SLC35A3 | -1.80 | -2.46 | -2.53 | -3.59 | 7.01 | 7.17 | 7.23 | 7.24 | 7.33 |
| ILMN_1701681 | SEC11C | -1.79 | -0.95 | -1.23 | -3.66 | 10.42 | 10.66 | 10.55 | 10.58 | 10.90 |
| ILMN_1821280 |  | -1.79 | -3.01 | -6.08 | -6.52 | 8.57 | 8.77 | 8.91 | 9.25 | 9.30 |
| ILMN_2210482 | MRPS34 | -1.79 | -1.48 | -1.82 | -3.30 | 7.34 | 7.50 | 7.47 | 7.50 | 7.63 |
| ILMN_1800897 | NRD1 | -1.78 | -0.55 | -2.65 | -3.01 | 9.76 | 9.95 | 9.82 | 10.04 | 10.08 |
| ILMN_2328433 | NOP2 | -1.78 | -0.16 | 0.54 | -2.73 | 8.81 | 9.11 | 8.84 | 8.72 | 9.27 |
| ILMN_1681972 | TMEM69 | -1.78 | -0.14 | -0.47 | -3.03 | 7.72 | 8.01 | 7.75 | 7.80 | 8.22 |
| ILMN_1747943 | ZNF695 | -1.78 | -1.97 | -2.02 | -3.56 | 6.85 | 6.98 | 6.99 | 6.99 | 7.10 |
| ILMN_2352009 | ACADVL | -1.77 | -0.28 | -2.61 | -6.49 | 7.67 | 7.80 | 7.69 | 7.86 | 8.14 |
| ILMN_1779343 | SNCB | -1.77 | 0.64 | -0.38 | -3.71 | 6.62 | 6.73 | 6.58 | 6.65 | 6.85 |
| ILMN_1768754 | PILRB | -1.77 | -0.97 | -1.79 | -3.18 | 8.80 | 9.11 | 8.97 | 9.11 | 9.36 |
| ILMN_1723632 | PIGC | -1.77 | 0.80 | 0.04 | -3.50 | 8.95 | 9.08 | 8.89 | 8.94 | 9.21 |
| ILMN_1683811 | TNPO3 | -1.77 | -1.23 | -0.69 | -2.92 | 8.13 | 8.36 | 8.29 | 8.22 | 8.51 |
| ILMN_1656196 | E2F6 | -1.77 | -1.39 | -2.10 | -3.04 | 8.45 | 8.70 | 8.65 | 8.75 | 8.88 |
| ILMN_2157421 | STUB1 | -1.77 | -1.60 | -1.29 | -3.06 | 7.87 | 8.04 | 8.03 | 8.00 | 8.17 |
| ILMN_1656066 | TNPO2 | -1.77 | -1.94 | -0.81 | -3.07 | 10.41 | 10.57 | 10.59 | 10.48 | 10.70 |
| ILMN_1707858 | H2AFZ | -1.76 | -0.11 | -0.21 | 2.86 | 13.01 | 13.17 | 13.02 | 13.03 | 12.74 |
| ILMN_1728062 | TLX2 | -1.76 | -1.20 | -3.43 | -3.68 | 6.63 | 6.82 | 6.76 | 7.00 | 7.02 |
| ILMN_1673944 | MANBAL | -1.76 | -1.84 | -1.95 | -3.07 | 9.50 | 9.78 | 9.79 | 9.81 | 9.99 |
| ILMN_3239217 | LOC729057 | -1.76 | -1.07 | -1.87 | -3.37 | 7.26 | 7.47 | 7.39 | 7.48 | 7.67 |
| ILMN_2393254 | CAPNS1 | -1.76 | -0.64 | -2.18 | -2.82 | 7.59 | 7.81 | 7.67 | 7.86 | 7.94 |
| ILMN_1666364 | COQ10A | -1.75 | -2.54 | -2.20 | -3.35 | 8.56 | 8.86 | 9.00 | 8.94 | 9.14 |
| ILMN_2212590 | TMEM170A | -1.75 | 0.50 | -0.32 | 4.89 | 7.82 | 7.98 | 7.77 | 7.85 | 7.36 |
| ILMN_1669572 | RNF126 | -1.75 | -0.92 | -3.15 | -3.18 | 8.04 | 8.22 | 8.14 | 8.37 | 8.37 |
| ILMN_1751561 | CAMK1D | -1.75 | -0.28 | -3.81 | 3.07 | 7.87 | 8.04 | 7.90 | 8.24 | 7.58 |
| ILMN_2067656 | CCND2 | -1.74 | -0.83 | -0.30 | -5.10 | 7.34 | 7.63 | 7.48 | 7.39 | 8.20 |
| ILMN_1793201 | HAGHL | -1.74 | -0.87 | -1.08 | -6.38 | 6.69 | 6.82 | 6.75 | 6.77 | 7.18 |
| ILMN_3310351 | RNU6-15 | -1.73 | -1.68 | -1.74 | -3.07 | 11.15 | 11.70 | 11.68 | 11.70 | 12.12 |
| ILMN_1761175 | RPS6KB2 | -1.73 | -1.45 | -2.09 | -3.80 | 7.53 | 7.83 | 7.78 | 7.90 | 8.20 |
| ILMN_1783333 | C16orf61 | -1.73 | 2.14 | 1.05 | 6.51 | 11.33 | 11.50 | 11.12 | 11.22 | 10.69 |
| ILMN_1691702 | ZNF775 | -1.73 | -1.26 | -2.44 | -3.79 | 7.92 | 8.06 | 8.02 | 8.12 | 8.22 |
| ILMN_3251312 | TMEM69 | -1.73 | -1.03 | -1.33 | -3.97 | 8.90 | 9.09 | 9.01 | 9.04 | 9.33 |
| ILMN_2321064 | BAX | -1.71 | -2.16 | -1.45 | -3.01 | 7.57 | 7.87 | 7.95 | 7.82 | 8.09 |
| ILMN_1711909 | EDEM2 | -1.71 | -2.87 | -2.35 | -6.80 | 8.06 | 8.20 | 8.29 | 8.25 | 8.59 |
| ILMN_1726138 | EI24 | -1.71 | -2.42 | -1.50 | -3.11 | 8.33 | 8.49 | 8.56 | 8.47 | 8.63 |
| ILMN_1782939 | ALB | -1.71 | -1.35 | -1.42 | -3.88 | 7.38 | 7.64 | 7.59 | 7.60 | 7.97 |
| ILMN_1691480 | LONP2 | -1.71 | 0.90 | -1.47 | 4.65 | 7.98 | 8.10 | 7.91 | 8.08 | 7.65 |
| ILMN_2085862 | SLC15A3 | -1.71 | -1.23 | -2.19 | -3.11 | 8.54 | 8.86 | 8.77 | 8.95 | 9.12 |
| ILMN_1657983 | TERF2IP | -1.70 | -0.35 | -1.05 | 4.05 | 9.36 | 9.57 | 9.40 | 9.49 | 8.86 |
| ILMN_1765082 | RBM10 | -1.70 | -4.62 | -4.20 | -3.40 | 8.37 | 8.48 | 8.66 | 8.64 | 8.59 |
| ILMN_2349129 | DPP3 | -1.70 | -1.14 | -1.35 | -4.23 | 8.32 | 8.49 | 8.43 | 8.45 | 8.73 |
| ILMN_1726181 | C19orf52 | -1.70 | -1.70 | -2.37 | -4.26 | 8.00 | 8.17 | 8.17 | 8.24 | 8.44 |
| ILMN_1721029 | RHEBL1 | -1.70 | -1.70 | -1.12 | -3.85 | 7.02 | 7.21 | 7.21 | 7.15 | 7.45 |
| ILMN_1780283 | C20orf201 | -1.70 | -0.64 | -2.83 | -4.69 | 7.10 | 7.34 | 7.19 | 7.50 | 7.77 |
| ILMN_3297510 | LOC729495 | -1.69 | -2.04 | -3.36 | -4.15 | 7.83 | 8.05 | 8.09 | 8.26 | 8.36 |
| ILMN_1728984 | PA2G4 | -1.69 | -0.71 | -0.62 | -2.99 | 10.22 | 10.52 | 10.35 | 10.33 | 10.75 |
| ILMN_1676588 | CEPT1 | -1.69 | -0.66 | -1.21 | -3.14 | 10.35 | 10.63 | 10.46 | 10.55 | 10.87 |
| ILMN_1764871 | PIGP | -1.69 | -0.62 | -2.21 | -3.32 | 9.57 | 9.74 | 9.63 | 9.80 | 9.91 |
| ILMN_2157075 | LRCH4 | -1.69 | -1.18 | -2.41 | -2.99 | 9.35 | 9.54 | 9.48 | 9.62 | 9.68 |
| ILMN_1733421 | PRKCQ | -1.69 | -2.17 | -2.71 | -4.35 | 6.81 | 6.93 | 6.97 | 7.01 | 7.13 |
| ILMN_3298423 | TOX2 | -1.69 | -1.42 | -1.06 | -5.61 | 7.06 | 7.31 | 7.27 | 7.22 | 7.90 |
| ILMN_1729455 | EML1 | -1.68 | -2.86 | -1.65 | -6.26 | 6.68 | 6.84 | 6.94 | 6.83 | 7.25 |
| ILMN_1799598 | SIRT5 | -1.68 | -0.27 | -0.94 | -2.78 | 7.23 | 7.37 | 7.25 | 7.31 | 7.46 |
| ILMN_1662243 | ING1 | -1.68 | 0.16 | -1.91 | 4.17 | 8.71 | 8.86 | 8.70 | 8.88 | 8.36 |
| ILMN_1706434 | LOC440359 | -1.67 | -1.86 | -2.28 | -2.96 | 8.83 | 9.17 | 9.20 | 9.29 | 9.42 |
| ILMN_1763694 | RSPRY1 | -1.67 | -0.59 | -0.96 | 3.63 | 9.82 | 10.00 | 9.89 | 9.93 | 9.43 |
| ILMN_1781987 | CDK5 | -1.67 | -2.78 | -2.29 | -5.99 | 8.42 | 8.63 | 8.76 | 8.70 | 9.16 |
| ILMN_1682864 | SPSB3 | -1.67 | -2.09 | -0.95 | -3.23 | 8.41 | 8.56 | 8.59 | 8.49 | 8.69 |
| ILMN_2389528 | MTL5 | -1.67 | -1.62 | -2.35 | -2.92 | 6.63 | 6.74 | 6.74 | 6.78 | 6.82 |
| ILMN_1658624 | UBXN6 | -1.67 | -2.87 | -2.93 | -3.86 | 7.98 | 8.16 | 8.29 | 8.30 | 8.40 |
| ILMN_1812940 | TRMT1 | -1.66 | -2.21 | -1.43 | -3.07 | 9.27 | 9.48 | 9.55 | 9.45 | 9.66 |
| ILMN_1789775 | WDR74 | -1.66 | -0.87 | -1.14 | -5.69 | 9.47 | 9.66 | 9.57 | 9.60 | 10.12 |
| ILMN_3230215 | TOX2 | -1.66 | -1.32 | -0.13 | -3.14 | 6.77 | 6.90 | 6.87 | 6.78 | 7.02 |
| ILMN_1705849 | SPR | -1.65 | 0.74 | -1.11 | -4.96 | 7.46 | 7.67 | 7.37 | 7.60 | 8.08 |
| ILMN_1658902 | DEAF1 | -1.65 | -3.33 | -2.07 | -8.35 | 7.27 | 7.40 | 7.54 | 7.44 | 7.93 |
| ILMN_1658821 | SAMD1 | -1.64 | -3.16 | -2.77 | -3.14 | 7.51 | 7.76 | 7.99 | 7.93 | 7.99 |
| ILMN_1794823 | ZNF626 | -1.63 | -1.73 | -1.83 | -2.72 | 6.62 | 6.79 | 6.80 | 6.81 | 6.90 |
| ILMN_1774584 | C2orf28 | -1.63 | -1.09 | -1.93 | -3.08 | 10.62 | 10.89 | 10.80 | 10.94 | 11.14 |
| ILMN_1661197 | CLCF1 | -1.63 | -0.09 | -0.75 | -5.83 | 7.05 | 7.23 | 7.06 | 7.13 | 7.67 |
| ILMN_1700831 | SLC27A2 | -1.62 | -1.29 | -3.03 | -8.03 | 7.02 | 7.22 | 7.18 | 7.39 | 7.99 |
| ILMN_1770803 | BNIP2 | -1.61 | 0.64 | 0.28 | 3.87 | 10.05 | 10.18 | 10.00 | 10.03 | 9.73 |
| ILMN_1670172 | WDR33 | -1.61 | -0.08 | -0.70 | -2.93 | 10.31 | 10.52 | 10.32 | 10.40 | 10.69 |
| ILMN_2231298 | SERHL2 | -1.61 | 0.61 | -0.11 | 2.93 | 6.96 | 7.11 | 6.90 | 6.97 | 6.68 |
| ILMN_2328972 | DNMT3B | -1.61 | 0.08 | 0.72 | -4.06 | 8.24 | 8.50 | 8.22 | 8.12 | 8.91 |
| ILMN_1736692 | SRL | -1.61 | -3.47 | -4.44 | -1.79 | 7.36 | 7.64 | 7.98 | 8.15 | 7.68 |
| ILMN_1662417 | LRPPRC | -1.60 | -3.04 | -1.80 | -4.37 | 9.16 | 9.39 | 9.61 | 9.42 | 9.80 |
| ILMN_1801109 | NARG1L | -1.60 | -0.08 | -0.27 | 3.09 | 8.82 | 9.02 | 8.83 | 8.85 | 8.43 |
| ILMN_2203891 | SMAD7 | -1.60 | -0.75 | -3.29 | -2.96 | 7.05 | 7.17 | 7.11 | 7.29 | 7.26 |
| ILMN_1721170 | GREB1 | -1.60 | 1.40 | 0.00 | 3.46 | 7.03 | 7.16 | 6.91 | 7.03 | 6.73 |
| ILMN_1718013 | CPSF3L | -1.59 | -1.51 | -0.62 | -3.26 | 8.27 | 8.41 | 8.40 | 8.33 | 8.55 |
| ILMN_1663054 | LOC653199 | -1.59 | -0.13 | 0.54 | 4.61 | 7.66 | 7.79 | 7.67 | 7.62 | 7.29 |
| ILMN_2041161 | DENND4A | -1.59 | 0.82 | -0.82 | 3.06 | 8.00 | 8.22 | 7.89 | 8.11 | 7.58 |
| ILMN_1661653 | DET1 | -1.58 | -2.54 | -1.73 | 2.78 | 7.62 | 7.73 | 7.80 | 7.74 | 7.43 |
| ILMN_3306440 | TMEM194A | -1.58 | -0.43 | -1.03 | -2.93 | 9.95 | 10.16 | 10.00 | 10.08 | 10.34 |
| ILMN_1723689 | RANBP3 | -1.58 | -2.34 | -1.53 | -2.87 | 7.41 | 7.52 | 7.57 | 7.51 | 7.61 |
| ILMN_2381121 | UQCC | -1.58 | 0.02 | 0.19 | -3.49 | 8.01 | 8.23 | 8.01 | 7.98 | 8.49 |
| ILMN_1766359 | GATAD2B | -1.58 | -1.34 | -3.33 | -3.57 | 8.22 | 8.38 | 8.35 | 8.55 | 8.57 |
| ILMN_1653042 | HSD3B7 | -1.58 | -0.66 | -0.48 | -3.85 | 6.83 | 6.95 | 6.88 | 6.87 | 7.13 |
| ILMN_1763723 | PTDSS2 | -1.58 | -2.28 | -2.36 | -5.18 | 7.77 | 7.96 | 8.04 | 8.05 | 8.39 |
| ILMN_1814194 | TCF4 | -1.57 | -1.47 | -4.33 | -1.60 | 9.93 | 10.09 | 10.08 | 10.38 | 10.10 |
| ILMN_1698019 | LGMN | -1.57 | 0.06 | -0.67 | -3.41 | 8.69 | 8.94 | 8.68 | 8.80 | 9.24 |
| ILMN_1676197 | LRP11 | -1.57 | -1.37 | -0.78 | -8.72 | 7.41 | 7.51 | 7.50 | 7.46 | 8.00 |
| ILMN_1703244 | MAP1LC3B | -1.57 | 0.78 | 0.44 | 4.73 | 9.46 | 9.64 | 9.37 | 9.41 | 8.92 |
| ILMN_1727300 | ZNF444 | -1.56 | -3.44 | -2.94 | -2.90 | 7.61 | 7.75 | 7.91 | 7.87 | 7.87 |
| ILMN_2380839 | SNRNP70 | -1.56 | -1.60 | -2.08 | -2.92 | 7.97 | 8.28 | 8.28 | 8.38 | 8.54 |
| ILMN_3223798 | ZNF84 | -1.56 | -4.05 | -2.61 | -4.68 | 7.19 | 7.31 | 7.51 | 7.39 | 7.56 |
| ILMN_1725518 | ANGPTL6 | -1.56 | -0.74 | -0.67 | -4.81 | 6.97 | 7.11 | 7.04 | 7.03 | 7.42 |
| ILMN_1690125 | PDLIM7 | -1.55 | -0.39 | -2.25 | -6.89 | 9.41 | 9.61 | 9.46 | 9.70 | 10.30 |
| ILMN_2368773 | FAM3C | -1.55 | -1.18 | -0.87 | -5.29 | 7.08 | 7.28 | 7.23 | 7.19 | 7.75 |
| ILMN_1700044 | SAP130 | -1.55 | -1.19 | -0.61 | -3.99 | 8.34 | 8.53 | 8.48 | 8.41 | 8.82 |
| ILMN_2312296 | PCBP2 | -1.55 | -2.38 | -1.72 | -4.22 | 12.30 | 12.46 | 12.54 | 12.48 | 12.73 |
| ILMN_1678928 | SLCO3A1 | -1.55 | -1.75 | -2.50 | -4.50 | 6.68 | 6.79 | 6.80 | 6.85 | 6.98 |
| ILMN_1782730 | ZNF473 | -1.55 | -1.15 | -0.13 | -3.85 | 6.87 | 6.99 | 6.96 | 6.88 | 7.16 |
| ILMN_1749009 | REXO2 | -1.54 | -0.36 | -1.13 | -3.20 | 9.95 | 10.12 | 9.99 | 10.07 | 10.31 |
| ILMN_1705783 | NXF1 | -1.54 | -4.11 | -3.64 | -5.74 | 8.91 | 9.03 | 9.23 | 9.20 | 9.36 |
| ILMN_3228595 | LOC729768 | -1.54 | 0.65 | 1.44 | -3.22 | 9.03 | 9.18 | 8.96 | 8.89 | 9.34 |
| ILMN_1813490 | FSD1 | -1.54 | -2.19 | -2.04 | -3.22 | 7.45 | 7.66 | 7.75 | 7.73 | 7.89 |
| ILMN_1775566 | ATP1A1 | -1.54 | -0.79 | -1.10 | -3.06 | 7.59 | 7.88 | 7.74 | 7.80 | 8.17 |
| ILMN_1793033 | RBM28 | -1.54 | -0.11 | 1.13 | -3.15 | 7.64 | 7.78 | 7.65 | 7.54 | 7.92 |
| ILMN_2185845 | BRSK1 | -1.54 | -2.38 | -1.87 | -5.24 | 7.29 | 7.43 | 7.51 | 7.46 | 7.77 |
| ILMN_2206411 | CTAG1A | -1.54 | -1.07 | -2.06 | -5.01 | 7.89 | 8.07 | 8.01 | 8.13 | 8.47 |
| ILMN_3201485 | LOC644988 | -1.53 | -1.87 | -3.79 | -3.88 | 7.56 | 7.73 | 7.77 | 7.98 | 7.99 |
| ILMN_1677402 | LOC387763 | -1.53 | -0.56 | -3.47 | -3.89 | 7.36 | 7.47 | 7.40 | 7.61 | 7.64 |
| ILMN_3295494 | LOC389386 | -1.53 | 1.49 | -0.32 | 2.99 | 8.20 | 8.36 | 8.05 | 8.24 | 7.89 |
| ILMN_3178529 | FAM108A2 | -1.52 | -1.44 | -3.52 | -3.78 | 7.55 | 7.73 | 7.72 | 7.95 | 7.98 |
| ILMN_1700268 | QPRT | -1.52 | -1.72 | -2.69 | -3.89 | 7.80 | 8.08 | 8.11 | 8.29 | 8.51 |
| ILMN_2198185 | CXorf12 | -1.52 | -0.94 | -1.22 | -4.63 | 7.27 | 7.49 | 7.41 | 7.45 | 7.94 |
| ILMN_1663919 | TFF2 | -1.51 | -0.42 | -1.67 | -3.13 | 6.81 | 7.00 | 6.86 | 7.02 | 7.21 |
| ILMN_2347748 | FLJ12949 | -1.51 | -1.25 | -1.02 | -2.87 | 8.25 | 8.44 | 8.41 | 8.38 | 8.62 |
| ILMN_1676848 | LOC728844 | -1.51 | -0.69 | 0.51 | -3.32 | 7.05 | 7.25 | 7.14 | 6.98 | 7.48 |
| ILMN_1736982 | PHACTR1 | -1.51 | -2.03 | -2.51 | -3.20 | 8.30 | 8.62 | 8.73 | 8.83 | 8.98 |
| ILMN_3203059 | LOC100131176 | -1.50 | -0.75 | -0.67 | -2.73 | 6.74 | 6.87 | 6.80 | 6.80 | 6.97 |
| ILMN_2334350 | BTBD3 | -1.50 | -3.56 | -4.57 | 1.85 | 8.62 | 9.18 | 9.95 | 10.33 | 7.93 |
| ILMN_1752579 | ATP6V0A1 | -1.50 | -1.47 | -0.77 | -3.09 | 8.95 | 9.12 | 9.12 | 9.04 | 9.30 |
| ILMN_2061446 | AADACL1 | -1.50 | 0.55 | -0.55 | 3.41 | 7.90 | 8.00 | 7.86 | 7.93 | 7.67 |
| ILMN_2319913 | DGKA | -1.50 | -0.23 | -0.32 | -4.24 | 7.46 | 7.64 | 7.49 | 7.50 | 7.95 |
| ILMN_1653466 | HES4 | -1.50 | -1.08 | 0.14 | -3.18 | 6.81 | 6.92 | 6.89 | 6.80 | 7.04 |
| ILMN_1713964 | BTBD3 | -1.50 | -3.70 | -4.87 | 1.66 | 7.92 | 8.42 | 9.15 | 9.54 | 7.36 |
| ILMN_1674941 | ANO6 | -1.49 | -0.41 | -1.86 | -3.95 | 7.11 | 7.24 | 7.14 | 7.27 | 7.46 |
| ILMN_1792168 | GALE | -1.49 | -0.16 | -1.02 | -5.34 | 7.78 | 7.91 | 7.80 | 7.87 | 8.24 |
| ILMN_2117323 | PIK3C2B | -1.49 | -4.23 | -3.40 | -6.38 | 8.40 | 8.59 | 8.93 | 8.83 | 9.20 |
| ILMN_2370772 | EIF4G1 | -1.49 | -2.44 | -1.75 | -3.31 | 9.11 | 9.33 | 9.47 | 9.37 | 9.60 |
| ILMN_1786108 | TUT1 | -1.48 | -1.57 | -0.93 | -3.08 | 7.86 | 8.04 | 8.05 | 7.98 | 8.24 |
| ILMN_1688011 | UPF1 | -1.48 | -2.41 | -1.02 | -3.89 | 7.29 | 7.45 | 7.55 | 7.40 | 7.71 |
| ILMN_2273595 | TEX11 | -1.48 | -0.76 | -1.02 | -2.79 | 6.70 | 6.82 | 6.76 | 6.78 | 6.92 |
| ILMN_2330307 | SLC43A3 | -1.48 | 0.07 | 0.00 | -3.59 | 7.67 | 7.80 | 7.66 | 7.67 | 7.99 |
| ILMN_2223720 | ATMIN | -1.48 | -0.52 | -2.27 | 4.26 | 8.30 | 8.44 | 8.35 | 8.52 | 7.88 |
| ILMN_1717934 | SYT11 | -1.48 | -1.80 | -0.93 | -3.59 | 8.26 | 8.60 | 8.67 | 8.47 | 9.07 |
| ILMN_2228453 | KIAA0562 | -1.48 | -0.29 | -0.12 | -4.31 | 7.50 | 7.62 | 7.52 | 7.51 | 7.85 |
| ILMN_2344907 | FBXO43 | -1.47 | 0.68 | -1.31 | -3.18 | 7.09 | 7.21 | 7.03 | 7.20 | 7.35 |
| ILMN_1738759 | PIGT | -1.47 | -2.89 | -3.76 | -4.85 | 9.33 | 9.64 | 9.94 | 10.12 | 10.35 |
| ILMN_1680347 | ZNF317 | -1.47 | -1.85 | -0.95 | -3.32 | 8.73 | 8.94 | 9.00 | 8.87 | 9.22 |
| ILMN_1682233 | ESCO1 | -1.47 | -1.02 | -1.47 | -2.96 | 8.39 | 8.57 | 8.51 | 8.57 | 8.74 |
| ILMN_1669788 | NUDT14 | -1.47 | 1.00 | 0.06 | -3.14 | 8.41 | 8.65 | 8.25 | 8.40 | 8.91 |
| ILMN_1785570 | SUSD3 | -1.46 | -3.54 | -1.04 | -7.45 | 7.56 | 7.75 | 8.03 | 7.70 | 8.54 |
| ILMN_3246065 | CCDC151 | -1.46 | -1.49 | -1.26 | -5.97 | 6.74 | 6.91 | 6.91 | 6.89 | 7.42 |
| ILMN_1687592 | WWC3 | -1.46 | -4.56 | -4.59 | -0.49 | 8.15 | 8.41 | 8.95 | 8.96 | 8.24 |
| ILMN_1765704 | CDC2L1 | -1.46 | -1.55 | -1.34 | -4.44 | 7.24 | 7.40 | 7.41 | 7.38 | 7.72 |
| ILMN_2415179 | CLSTN1 | -1.46 | -2.71 | -2.28 | -3.27 | 9.83 | 10.01 | 10.17 | 10.11 | 10.24 |
| ILMN_1664177 | ATXN7L2 | -1.46 | -1.20 | -1.34 | -3.94 | 7.96 | 8.12 | 8.09 | 8.11 | 8.41 |
| ILMN_1651872 | UBIAD1 | -1.46 | 0.56 | 0.56 | -3.28 | 9.28 | 9.44 | 9.22 | 9.22 | 9.64 |
| ILMN_3256674 | LOC100128485 | -1.45 | -1.65 | -1.65 | -3.14 | 6.95 | 7.08 | 7.10 | 7.10 | 7.23 |
| ILMN_2404539 | C20orf30 | -1.45 | -0.94 | -0.51 | -2.73 | 10.87 | 11.03 | 10.98 | 10.93 | 11.17 |
| ILMN_1695420 | CLTA | -1.45 | 1.18 | 1.22 | 3.62 | 9.75 | 9.90 | 9.64 | 9.63 | 9.40 |
| ILMN_1714108 | TP53INP1 | -1.45 | -1.88 | -0.03 | -3.81 | 7.45 | 7.61 | 7.65 | 7.45 | 7.87 |
| ILMN_3191922 | KRT8P9 | -1.45 | -0.37 | -0.99 | 3.40 | 6.96 | 7.08 | 6.99 | 7.04 | 6.69 |
| ILMN_1768534 | BHLHB2 | -1.45 | -2.37 | -4.54 | -2.68 | 9.61 | 9.81 | 9.94 | 10.24 | 9.98 |
| ILMN_2103761 | TLE4 | -1.45 | -0.78 | -1.98 | 3.09 | 9.27 | 9.44 | 9.36 | 9.50 | 8.90 |
| ILMN_1675482 | LOC732425 | -1.44 | -2.60 | -3.55 | -4.42 | 7.18 | 7.29 | 7.39 | 7.46 | 7.53 |
| ILMN_1805519 | CD24 | -1.44 | 0.04 | -0.83 | -2.77 | 7.35 | 7.47 | 7.35 | 7.42 | 7.58 |
| ILMN_2214678 | MXD1 | -1.44 | 1.62 | 2.07 | 5.01 | 7.22 | 7.32 | 7.10 | 7.06 | 6.85 |
| ILMN_1657797 | FIBP | -1.44 | 0.15 | -0.60 | -2.91 | 10.24 | 10.43 | 10.22 | 10.32 | 10.63 |
| ILMN_1676520 | KGFLP1 | -1.44 | 0.19 | 0.04 | -3.42 | 7.09 | 7.22 | 7.08 | 7.09 | 7.39 |
| ILMN_1706706 | WDR68 | -1.44 | -0.10 | -1.93 | -3.03 | 9.53 | 9.71 | 9.54 | 9.78 | 9.92 |
| ILMN_1754199 | BCL7B | -1.44 | -1.68 | -1.06 | -3.12 | 7.35 | 7.45 | 7.47 | 7.43 | 7.57 |
| ILMN_1765621 | HDGF | -1.43 | -2.14 | -1.71 | -3.49 | 9.44 | 9.66 | 9.77 | 9.71 | 9.98 |
| ILMN_1658802 | KRTCAP2 | -1.43 | -1.56 | -2.24 | -6.24 | 10.31 | 10.50 | 10.52 | 10.61 | 11.14 |
| ILMN_1723846 | FAM119B | -1.43 | -1.40 | -2.07 | -4.45 | 8.54 | 8.79 | 8.78 | 8.90 | 9.32 |
| ILMN_1723743 | ROM1 | -1.43 | -0.15 | -0.10 | -3.74 | 7.18 | 7.28 | 7.19 | 7.19 | 7.43 |
| ILMN_2375599 | RNH1 | -1.43 | -2.35 | -3.19 | -8.11 | 7.52 | 7.64 | 7.71 | 7.78 | 8.17 |
| ILMN_1798360 | CXCR7 | -1.43 | 0.27 | 0.31 | -3.43 | 7.15 | 7.38 | 7.10 | 7.10 | 7.71 |
| ILMN_1680738 | C5orf13 | -1.43 | -1.57 | -1.57 | -3.05 | 10.80 | 11.00 | 11.02 | 11.02 | 11.22 |
| ILMN_1732577 | TMEM216 | -1.42 | -2.42 | -2.13 | -3.37 | 8.74 | 9.02 | 9.22 | 9.16 | 9.40 |
| ILMN_3293049 | LOC284167 | -1.42 | -3.24 | -3.51 | -7.46 | 8.39 | 8.51 | 8.66 | 8.69 | 9.02 |
| ILMN_1778013 | PDPR | -1.42 | -0.83 | -0.22 | 3.27 | 7.59 | 7.74 | 7.68 | 7.61 | 7.23 |
| ILMN_2228710 | PDCD5 | -1.41 | -1.00 | -0.55 | -3.33 | 9.31 | 9.48 | 9.43 | 9.37 | 9.71 |
| ILMN_2241953 | PILRA | -1.41 | -0.60 | -1.91 | -4.22 | 6.76 | 6.94 | 6.83 | 7.00 | 7.29 |
| ILMN_1699049 | PMS2L3 | -1.41 | -0.70 | -1.55 | -3.35 | 7.69 | 7.85 | 7.77 | 7.87 | 8.08 |
| ILMN_1746720 | TTC39C | -1.41 | 0.95 | -1.59 | -3.59 | 7.75 | 7.86 | 7.68 | 7.87 | 8.02 |
| ILMN_1703246 | SBF1 | -1.41 | -4.38 | -6.56 | -3.36 | 8.86 | 9.01 | 9.32 | 9.55 | 9.21 |
| ILMN_1708151 | LAGE3 | -1.41 | -2.81 | -1.27 | -3.09 | 9.61 | 9.71 | 9.81 | 9.70 | 9.83 |
| ILMN_1723768 | NLRX1 | -1.40 | -1.30 | 0.94 | -3.02 | 7.08 | 7.17 | 7.16 | 7.02 | 7.27 |
| ILMN_1741997 | SNRPC | -1.40 | -0.88 | -0.79 | -3.81 | 8.77 | 9.00 | 8.91 | 8.90 | 9.38 |
| ILMN_1713406 | FAM39DP | -1.40 | -2.95 | -2.63 | -2.92 | 8.94 | 9.10 | 9.28 | 9.24 | 9.28 |
| ILMN_2344455 | G3BP1 | -1.40 | 0.68 | 0.56 | 2.98 | 10.17 | 10.32 | 10.10 | 10.11 | 9.85 |
| ILMN_1801504 | RUNX1 | -1.39 | -0.87 | -1.36 | -3.56 | 8.15 | 8.31 | 8.25 | 8.30 | 8.56 |
| ILMN_1654939 | TMED2 | -1.39 | -2.50 | -1.67 | -5.43 | 9.85 | 10.00 | 10.12 | 10.03 | 10.44 |
| ILMN_1705686 | NRGN | -1.38 | -1.95 | -2.24 | -4.98 | 7.08 | 7.20 | 7.25 | 7.28 | 7.52 |
| ILMN_1777190 | CFD | -1.38 | -0.51 | -2.67 | -6.58 | 6.81 | 6.94 | 6.86 | 7.07 | 7.46 |
| ILMN_2186597 | RPP21 | -1.38 | -1.41 | -1.63 | -3.61 | 10.50 | 10.65 | 10.65 | 10.67 | 10.88 |
| ILMN_1783675 | ASB8 | -1.38 | -0.24 | 0.52 | -2.93 | 7.94 | 8.07 | 7.96 | 7.89 | 8.22 |
| ILMN_1746598 | SCNM1 | -1.38 | 0.02 | -0.39 | -3.75 | 8.46 | 8.66 | 8.46 | 8.52 | 9.00 |
| ILMN_1676555 | TTC26 | -1.38 | -1.45 | -0.92 | -3.18 | 6.98 | 7.10 | 7.11 | 7.06 | 7.26 |
| ILMN_2378952 | GPX4 | -1.37 | -0.28 | -0.59 | -4.71 | 10.60 | 10.78 | 10.64 | 10.68 | 11.22 |
| ILMN_1809433 | XBP1 | -1.37 | 1.26 | -0.18 | 3.59 | 9.35 | 9.58 | 9.14 | 9.38 | 8.74 |
| ILMN_2145116 | TMEM173 | -1.37 | -0.32 | 1.02 | -3.98 | 7.06 | 7.19 | 7.09 | 6.97 | 7.44 |
| ILMN_1751195 | LOC653438 | -1.37 | -1.81 | -3.16 | -3.54 | 7.55 | 7.73 | 7.79 | 7.96 | 8.01 |
| ILMN_2229170 | CRSP9 | -1.37 | 1.60 | 1.71 | 2.86 | 7.52 | 7.64 | 7.38 | 7.37 | 7.27 |
| ILMN_1716195 | HIST1H2BG | -1.37 | 2.57 | 1.08 | 4.19 | 8.08 | 8.24 | 7.78 | 7.95 | 7.59 |
| ILMN_2060105 | PPAN-P2RY11 | -1.37 | -1.07 | -2.27 | -3.33 | 7.10 | 7.24 | 7.21 | 7.33 | 7.44 |
| ILMN_1678962 | DFFB | -1.37 | -2.18 | -2.18 | -6.88 | 7.45 | 7.62 | 7.73 | 7.73 | 8.32 |
| ILMN_2319825 | ACYP1 | -1.36 | 0.26 | -0.77 | -3.20 | 8.43 | 8.65 | 8.39 | 8.55 | 8.93 |
| ILMN_1665871 | ANGEL1 | -1.36 | -2.85 | -2.97 | -7.06 | 8.23 | 8.34 | 8.46 | 8.47 | 8.80 |
| ILMN_1761101 | CCDC112 | -1.36 | 2.21 | 2.19 | 4.96 | 10.04 | 10.25 | 9.69 | 9.69 | 9.26 |
| ILMN_2058468 | BACH2 | -1.36 | -0.50 | -2.27 | -3.41 | 9.60 | 9.77 | 9.66 | 9.88 | 10.01 |
| ILMN_1779825 | PRDM2 | -1.36 | -2.48 | -1.87 | -3.93 | 6.59 | 6.68 | 6.76 | 6.72 | 6.87 |
| ILMN_1693311 | TMBIM6 | -1.35 | -2.79 | -1.86 | -6.62 | 10.81 | 10.93 | 11.05 | 10.97 | 11.38 |
| ILMN_1797425 | DDX55 | -1.35 | -2.96 | -2.35 | -3.92 | 9.78 | 9.98 | 10.23 | 10.14 | 10.38 |
| ILMN_1698934 | CMTM7 | -1.35 | -2.50 | -3.88 | -1.58 | 10.45 | 10.73 | 10.97 | 11.25 | 10.78 |
| ILMN_1794711 | LOC642477 | -1.35 | -0.88 | -2.04 | -3.39 | 7.66 | 7.85 | 7.79 | 7.95 | 8.15 |
| ILMN_1657760 | SYT17 | -1.34 | -1.46 | -0.85 | -2.88 | 7.89 | 8.12 | 8.14 | 8.04 | 8.38 |
| ILMN_1662328 | CNNM3 | -1.34 | -2.56 | -2.89 | -3.86 | 8.29 | 8.40 | 8.50 | 8.52 | 8.60 |
| ILMN_2391861 | GSTM1 | -1.34 | -1.39 | -2.17 | -2.95 | 8.77 | 9.05 | 9.06 | 9.22 | 9.39 |
| ILMN_1730523 | FAM195A | -1.34 | -0.29 | -1.40 | -5.19 | 7.27 | 7.40 | 7.30 | 7.41 | 7.80 |
| ILMN_2046896 | ESRRAP2 | -1.33 | 0.03 | -0.88 | -4.02 | 9.14 | 9.29 | 9.13 | 9.24 | 9.61 |
| ILMN_2064725 | METTL7B | -1.33 | -0.80 | -2.94 | -6.11 | 7.01 | 7.17 | 7.11 | 7.36 | 7.74 |
| ILMN_3226045 | LOC728533 | -1.33 | -0.44 | -0.55 | 5.35 | 11.29 | 11.42 | 11.34 | 11.35 | 10.77 |
| ILMN_2319000 | MATK | -1.33 | -0.76 | -2.38 | -3.42 | 7.02 | 7.22 | 7.13 | 7.37 | 7.52 |
| ILMN_1787762 | HEATR1 | -1.33 | 0.03 | -0.32 | -3.10 | 9.91 | 10.05 | 9.90 | 9.94 | 10.23 |
| ILMN_1698243 | C1orf85 | -1.32 | -1.48 | -0.62 | -5.73 | 8.97 | 9.14 | 9.16 | 9.05 | 9.71 |
| ILMN_3237665 | COX7A2L | -1.32 | -2.70 | -2.53 | -4.09 | 10.10 | 10.30 | 10.51 | 10.48 | 10.71 |
| ILMN_1712776 | KCNK7 | -1.32 | -0.08 | -1.39 | -5.03 | 7.02 | 7.14 | 7.03 | 7.14 | 7.46 |
| ILMN_1785037 | SSR2 | -1.32 | -1.95 | -2.87 | -3.23 | 7.12 | 7.23 | 7.29 | 7.36 | 7.39 |
| ILMN_2214144 | TWSG1 | -1.31 | -2.57 | -3.35 | -4.55 | 8.76 | 8.99 | 9.20 | 9.33 | 9.54 |
| ILMN_1784031 | TIMM44 | -1.31 | -0.96 | -2.31 | -4.43 | 8.17 | 8.29 | 8.26 | 8.39 | 8.59 |
| ILMN_3226904 | NOP2 | -1.31 | 0.38 | 0.46 | -3.24 | 8.50 | 8.72 | 8.44 | 8.42 | 9.04 |
| ILMN_1803686 | ADA | -1.31 | -3.74 | -3.21 | -4.55 | 8.10 | 8.29 | 8.63 | 8.55 | 8.74 |
| ILMN_3241979 | TMEM179B | -1.31 | -0.10 | -0.67 | -2.94 | 8.64 | 8.82 | 8.66 | 8.73 | 9.04 |
| ILMN_2152581 | STK38 | -1.31 | -0.86 | -2.07 | -4.82 | 8.19 | 8.38 | 8.31 | 8.49 | 8.90 |
| ILMN_1677404 | RAP2A | -1.30 | -1.61 | -3.07 | 3.41 | 8.80 | 8.94 | 8.97 | 9.13 | 8.43 |
| ILMN_3235168 | MUL1 | -1.30 | -0.89 | 0.34 | -4.31 | 9.16 | 9.29 | 9.25 | 9.13 | 9.58 |
| ILMN_1787410 | EIF6 | -1.30 | -1.97 | -2.26 | -5.40 | 10.33 | 10.55 | 10.67 | 10.72 | 11.26 |
| ILMN_1723235 | DUS3L | -1.29 | -2.82 | -1.95 | -4.11 | 8.56 | 8.70 | 8.87 | 8.78 | 9.02 |
| ILMN_2388975 | CERK | -1.29 | -1.99 | -4.41 | -4.11 | 8.83 | 9.04 | 9.16 | 9.57 | 9.52 |
| ILMN_1766916 | RPAP3 | -1.29 | 0.21 | -0.07 | -3.98 | 8.99 | 9.18 | 8.96 | 9.00 | 9.57 |
| ILMN_1790471 | CICE | -1.29 | 0.95 | 0.56 | -2.86 | 7.62 | 7.77 | 7.51 | 7.55 | 7.96 |
| ILMN_1697561 | FBXL16 | -1.29 | -0.78 | -0.32 | -2.81 | 6.69 | 6.78 | 6.74 | 6.71 | 6.89 |
| ILMN_1700306 | OCIAD2 | -1.29 | 0.88 | -0.66 | 2.80 | 10.25 | 10.37 | 10.17 | 10.31 | 10.00 |
| ILMN_1800935 | ARV1 | -1.29 | 0.85 | -0.50 | -7.31 | 8.85 | 8.99 | 8.75 | 8.90 | 9.68 |
| ILMN_3227485 | LOC729198 | -1.29 | -0.60 | -1.67 | 3.47 | 7.03 | 7.13 | 7.08 | 7.16 | 6.76 |
| ILMN_1703013 | NOC4L | -1.28 | -1.72 | -0.71 | -3.44 | 6.92 | 7.05 | 7.09 | 6.99 | 7.26 |
| ILMN_1805216 | GPC6 | -1.28 | -1.45 | -1.36 | -3.22 | 7.10 | 7.20 | 7.22 | 7.21 | 7.36 |
| ILMN_1667295 | VASN | -1.28 | -1.54 | -1.94 | -5.61 | 6.66 | 6.75 | 6.77 | 6.80 | 7.08 |
| ILMN_1715693 | LOC440160 | -1.27 | -1.55 | -2.82 | -3.64 | 8.75 | 8.94 | 8.98 | 9.16 | 9.28 |
| ILMN_1797875 | ALOX5AP | -1.27 | -1.24 | -2.22 | -3.38 | 11.93 | 12.15 | 12.15 | 12.32 | 12.52 |
| ILMN_1689378 | CCRN4L | -1.27 | 0.31 | 0.71 | 4.15 | 8.47 | 8.66 | 8.42 | 8.36 | 7.85 |
| ILMN_2108709 | ANKRD11 | -1.27 | -2.35 | -2.13 | 4.21 | 9.76 | 9.92 | 10.05 | 10.03 | 9.24 |
| ILMN_2401779 | FAM102A | -1.27 | -1.63 | -2.81 | -3.46 | 7.79 | 7.97 | 8.03 | 8.20 | 8.30 |
| ILMN_1792972 | ZNF439 | -1.26 | -1.56 | -1.72 | -3.60 | 8.25 | 8.58 | 8.66 | 8.70 | 9.18 |
| ILMN_1727200 | SLCO4A1 | -1.26 | -1.87 | -1.92 | -5.82 | 7.01 | 7.08 | 7.12 | 7.12 | 7.36 |
| ILMN_2313158 | MBNL1 | -1.26 | -0.81 | -2.58 | -3.45 | 9.16 | 9.31 | 9.25 | 9.46 | 9.57 |
| ILMN_1747506 | DHX34 | -1.26 | 0.52 | -1.52 | -3.74 | 7.84 | 7.94 | 7.80 | 7.96 | 8.13 |
| ILMN_1678678 | SLC37A4 | -1.26 | -0.96 | -1.59 | -4.29 | 9.48 | 9.60 | 9.57 | 9.63 | 9.87 |
| ILMN_2337931 | CXCR5 | -1.26 | -2.08 | -1.41 | -8.17 | 7.07 | 7.18 | 7.25 | 7.19 | 7.76 |
| ILMN_2371911 | MUC1 | -1.25 | -0.87 | -1.15 | -2.98 | 6.66 | 6.74 | 6.72 | 6.74 | 6.86 |
| ILMN_1734608 | ZNF77 | -1.25 | -1.83 | -1.51 | -2.84 | 6.89 | 7.15 | 7.27 | 7.20 | 7.48 |
| ILMN_1783448 | DYNC1LI2 | -1.25 | -0.75 | 2.14 | 6.14 | 10.01 | 10.13 | 10.08 | 9.81 | 9.44 |
| ILMN_2304495 | PPP1R1B | -1.25 | -1.12 | -1.76 | -8.02 | 7.02 | 7.18 | 7.16 | 7.25 | 8.05 |
| ILMN_2372639 | TRAPPC5 | -1.24 | -1.05 | -1.64 | -3.31 | 9.38 | 9.60 | 9.56 | 9.67 | 9.96 |
| ILMN_3245066 | DENND4B | -1.24 | -3.51 | -2.86 | -3.88 | 9.39 | 9.55 | 9.83 | 9.75 | 9.87 |
| ILMN_1778255 | FARSA | -1.24 | -1.31 | -1.13 | -3.14 | 9.16 | 9.27 | 9.28 | 9.26 | 9.44 |
| ILMN_1772644 | EML3 | -1.24 | -2.08 | -2.38 | -5.18 | 8.86 | 9.03 | 9.14 | 9.18 | 9.56 |
| ILMN_1758523 | ABCA3 | -1.24 | -2.70 | -4.88 | -7.24 | 7.60 | 7.75 | 7.93 | 8.19 | 8.48 |
| ILMN_2311761 | AP3S1 | -1.24 | 0.66 | 0.91 | 3.10 | 8.44 | 8.60 | 8.35 | 8.32 | 8.03 |
| ILMN_1716086 | WDR33 | -1.24 | -0.61 | -0.49 | -2.74 | 7.75 | 7.93 | 7.84 | 7.82 | 8.15 |
| ILMN_3308335 | RNU6-1 | -1.23 | -1.11 | -1.17 | -2.97 | 11.24 | 11.61 | 11.58 | 11.59 | 12.14 |
| ILMN_1773751 | HRAS | -1.23 | -1.62 | -1.14 | -3.27 | 8.72 | 8.86 | 8.90 | 8.85 | 9.08 |
| ILMN_1731914 | SCN4A | -1.23 | -2.97 | -1.98 | -6.02 | 6.88 | 6.98 | 7.13 | 7.04 | 7.38 |
| ILMN_1805922 | EBPL | -1.23 | -0.20 | -0.94 | -3.26 | 8.45 | 8.67 | 8.48 | 8.62 | 9.05 |
| ILMN_1736548 | PHACTR4 | -1.22 | 0.26 | -0.71 | 4.29 | 9.78 | 9.91 | 9.75 | 9.85 | 9.34 |
| ILMN_1795778 | P4HA2 | -1.22 | 0.98 | -0.20 | 2.81 | 7.17 | 7.27 | 7.09 | 7.19 | 6.94 |
| ILMN_1685012 | EAF1 | -1.22 | 1.22 | -0.21 | 3.18 | 8.69 | 8.81 | 8.57 | 8.71 | 8.39 |
| ILMN_1679476 | GART | -1.22 | -0.34 | 0.00 | -4.01 | 10.66 | 10.78 | 10.69 | 10.66 | 11.05 |
| ILMN_3238326 | RNF144A | -1.22 | -1.53 | -2.34 | -4.73 | 6.67 | 6.75 | 6.77 | 6.82 | 6.98 |
| ILMN_1701306 | MRRF | -1.22 | 0.18 | 0.62 | -2.75 | 7.23 | 7.38 | 7.20 | 7.15 | 7.58 |
| ILMN_2340721 | TMEM134 | -1.22 | -2.24 | -1.74 | -5.76 | 8.28 | 8.43 | 8.55 | 8.49 | 8.98 |
| ILMN_2331062 | CBFA2T2 | -1.21 | -1.75 | 0.30 | -4.48 | 7.15 | 7.26 | 7.31 | 7.13 | 7.55 |
| ILMN_1798083 | CHERP | -1.21 | -2.26 | -0.61 | -3.30 | 7.52 | 7.61 | 7.69 | 7.56 | 7.77 |
| ILMN_1683305 | COMMD2 | -1.21 | -0.10 | 0.03 | 4.52 | 9.06 | 9.19 | 9.07 | 9.06 | 8.60 |
| ILMN_1677440 | ATP6AP2 | -1.21 | -0.64 | -0.34 | -2.93 | 10.72 | 10.84 | 10.78 | 10.75 | 11.01 |
| ILMN_1706261 | SLCO3A1 | -1.21 | -2.25 | -3.09 | -5.15 | 6.74 | 6.86 | 6.96 | 7.04 | 7.25 |
| ILMN_1773407 | C16orf72 | -1.21 | 0.21 | -0.17 | 3.08 | 9.61 | 9.78 | 9.58 | 9.63 | 9.18 |
| ILMN_1749210 | BUD13 | -1.21 | -1.91 | -0.62 | -2.84 | 7.83 | 7.93 | 7.99 | 7.88 | 8.07 |
| ILMN_1807491 | LAIR2 | -1.21 | -1.09 | -0.94 | -3.36 | 6.70 | 6.81 | 6.80 | 6.78 | 7.00 |
| ILMN_2330584 | DDX19B | -1.21 | 0.64 | 0.45 | 3.93 | 7.63 | 7.80 | 7.54 | 7.57 | 7.08 |
| ILMN_1711699 | LOC728014 | -1.21 | -3.00 | -3.56 | -7.38 | 8.60 | 8.73 | 8.94 | 9.00 | 9.43 |
| ILMN_1757697 | NEIL3 | -1.20 | 0.40 | 0.25 | 3.97 | 7.80 | 7.93 | 7.76 | 7.78 | 7.37 |
| ILMN_1725271 | GPR3 | -1.20 | -1.11 | -0.90 | -2.73 | 6.69 | 6.79 | 6.78 | 6.76 | 6.91 |
| ILMN_1786789 | FAM102B | -1.20 | -1.74 | -4.73 | -6.19 | 8.90 | 8.97 | 9.00 | 9.19 | 9.28 |
| ILMN_1702712 | TRIM55 | -1.19 | -2.24 | -3.76 | -4.45 | 6.53 | 6.64 | 6.74 | 6.88 | 6.94 |
| ILMN_2143795 | MGC4677 | -1.19 | 0.70 | 0.83 | -3.24 | 7.74 | 7.92 | 7.64 | 7.62 | 8.22 |
| ILMN_2387919 | PRKAG2 | -1.19 | -1.04 | -2.46 | -4.80 | 7.06 | 7.17 | 7.15 | 7.28 | 7.49 |
| ILMN_1691178 | LOC650003 | -1.19 | -2.04 | -4.04 | -3.78 | 6.75 | 6.84 | 6.91 | 7.06 | 7.04 |
| ILMN_2354381 | PON2 | -1.19 | -1.21 | -2.67 | -4.43 | 8.56 | 8.73 | 8.74 | 8.95 | 9.21 |
| ILMN_1783843 | MIIP | -1.19 | -0.80 | -2.03 | -3.95 | 7.59 | 7.71 | 7.67 | 7.79 | 7.97 |
| ILMN_1795388 | INSM2 | -1.19 | -1.55 | -0.50 | -4.66 | 6.86 | 7.04 | 7.10 | 6.93 | 7.58 |
| ILMN_3240187 | TMEM111 | -1.19 | 0.03 | 0.28 | -4.96 | 9.39 | 9.53 | 9.38 | 9.35 | 9.99 |
| ILMN_1913678 |  | -1.18 | -2.49 | -2.84 | -5.68 | 8.16 | 8.55 | 8.99 | 9.10 | 10.04 |
| ILMN_2066124 | AFG3L2 | -1.18 | -1.23 | -0.35 | -3.78 | 10.36 | 10.50 | 10.51 | 10.40 | 10.83 |
| ILMN_2396148 | HIP1R | -1.18 | -2.22 | -3.47 | -7.80 | 7.36 | 7.47 | 7.57 | 7.69 | 8.09 |
| ILMN_3290380 | LOC387703 | -1.18 | -2.40 | -1.04 | -3.99 | 9.95 | 10.03 | 10.12 | 10.02 | 10.24 |
| ILMN_2359456 | ERGIC3 | -1.18 | -0.59 | -0.18 | -3.77 | 8.33 | 8.47 | 8.40 | 8.35 | 8.76 |
| ILMN_1814789 | UBAP2L | -1.18 | -1.95 | -1.07 | -4.51 | 8.96 | 9.14 | 9.25 | 9.12 | 9.64 |
| ILMN_1772527 | C12orf44 | -1.18 | -1.30 | -2.07 | -6.58 | 8.99 | 9.11 | 9.13 | 9.21 | 9.68 |
| ILMN_1798485 | ATP6V1E1 | -1.18 | 0.38 | 0.50 | -4.83 | 10.19 | 10.34 | 10.14 | 10.12 | 10.83 |
| ILMN_3248113 | SNRNP70 | -1.17 | -3.22 | -2.43 | -3.03 | 9.90 | 10.05 | 10.30 | 10.20 | 10.27 |
| ILMN_2369580 | C16orf35 | -1.17 | -1.28 | -0.11 | -3.12 | 9.41 | 9.52 | 9.53 | 9.42 | 9.71 |
| ILMN_3240370 | C11orf83 | -1.17 | -2.44 | -1.86 | -4.81 | 8.13 | 8.28 | 8.45 | 8.37 | 8.76 |
| ILMN_1667081 | CCND2 | -1.17 | -1.09 | -0.25 | -5.18 | 6.83 | 6.93 | 6.92 | 6.85 | 7.25 |
| ILMN_2377900 | MAP1B | -1.17 | -1.09 | -2.39 | 2.86 | 7.40 | 7.56 | 7.55 | 7.72 | 7.02 |
| ILMN_1761721 | VPS35 | -1.17 | 0.00 | -0.95 | 2.78 | 10.55 | 10.68 | 10.55 | 10.65 | 10.26 |
| ILMN_2201580 | GSTM2 | -1.17 | -1.40 | -2.26 | -3.23 | 8.32 | 8.53 | 8.58 | 8.74 | 8.92 |
| ILMN_2415189 | ATP1A1 | -1.16 | -0.72 | -1.97 | -5.22 | 10.34 | 10.44 | 10.40 | 10.50 | 10.77 |
| ILMN_3227250 | LOC729941 | -1.16 | 1.72 | 0.19 | 2.75 | 7.00 | 7.09 | 6.88 | 6.99 | 6.81 |
| ILMN_1682957 | PACSIN3 | -1.16 | -0.66 | -0.15 | -3.06 | 6.89 | 6.99 | 6.95 | 6.91 | 7.16 |
| ILMN_2384536 | PECI | -1.16 | 1.61 | 1.88 | 3.57 | 7.69 | 7.84 | 7.49 | 7.46 | 7.25 |
| ILMN_1751425 | ERMP1 | -1.15 | -1.11 | -2.51 | -3.54 | 7.17 | 7.26 | 7.26 | 7.38 | 7.46 |
| ILMN_1808584 | FAM36A | -1.15 | 0.64 | 0.06 | 4.77 | 8.60 | 8.72 | 8.53 | 8.59 | 8.07 |
| ILMN_1830462 | XYLT1 | -1.15 | -1.39 | -2.11 | -3.76 | 8.16 | 8.34 | 8.37 | 8.49 | 8.74 |
| ILMN_1761797 | CSTB | -1.15 | 0.93 | 0.83 | -3.10 | 9.27 | 9.50 | 9.09 | 9.11 | 9.88 |
| ILMN_1776173 | PSMD7 | -1.15 | 0.45 | -0.81 | 4.16 | 10.74 | 10.91 | 10.67 | 10.86 | 10.13 |
| ILMN_1797107 | SCLT1 | -1.15 | 0.08 | -0.32 | 4.29 | 7.30 | 7.40 | 7.30 | 7.33 | 6.94 |
| ILMN_2403247 | CMTM7 | -1.15 | -2.43 | -3.80 | -2.08 | 8.88 | 9.12 | 9.39 | 9.68 | 9.32 |
| ILMN_1656951 | APCDD1 | -1.15 | -1.90 | -5.35 | -1.15 | 7.21 | 7.36 | 7.46 | 7.92 | 7.36 |
| ILMN_3262031 | BRD7P2 | -1.15 | -1.59 | 0.18 | 7.41 | 9.33 | 9.41 | 9.45 | 9.31 | 8.77 |
| ILMN_1676759 | DDX27 | -1.15 | -1.29 | -1.34 | -2.80 | 8.42 | 8.58 | 8.60 | 8.61 | 8.81 |
| ILMN_1775170 | MT1X | -1.15 | 0.23 | 3.84 | 3.71 | 9.25 | 9.50 | 9.20 | 8.40 | 8.43 |
| ILMN_1767848 | PCMTD2 | -1.14 | -2.68 | -0.75 | -4.70 | 9.28 | 9.43 | 9.64 | 9.38 | 9.91 |
| ILMN_1739618 | ZNF408 | -1.14 | -0.87 | -0.54 | -2.95 | 7.71 | 7.84 | 7.81 | 7.77 | 8.04 |
| ILMN_1758055 | YIF1B | -1.14 | -1.11 | -2.42 | -3.87 | 7.56 | 7.69 | 7.69 | 7.85 | 8.02 |
| ILMN_1726967 | TWSG1 | -1.14 | -0.92 | -1.27 | -3.29 | 6.81 | 6.90 | 6.88 | 6.91 | 7.06 |
| ILMN_1809866 | WDR74 | -1.14 | -1.06 | -0.64 | -2.94 | 9.93 | 10.08 | 10.07 | 10.02 | 10.33 |
| ILMN_1655915 | MMP11 | -1.14 | -2.57 | -3.29 | -3.56 | 6.76 | 6.86 | 6.99 | 7.05 | 7.08 |
| ILMN_1740418 | CYP27B1 | -1.13 | -0.96 | -1.20 | -3.72 | 7.44 | 7.55 | 7.53 | 7.56 | 7.79 |
| ILMN_1782560 | CD86 | -1.13 | 0.71 | 1.13 | -4.86 | 6.94 | 7.02 | 6.89 | 6.86 | 7.29 |
| ILMN_1808707 | FSCN1 | -1.13 | -0.13 | -0.91 | -3.67 | 7.67 | 7.82 | 7.68 | 7.79 | 8.15 |
| ILMN_1658619 | WWC1 | -1.13 | -0.38 | 0.19 | -3.02 | 6.95 | 7.03 | 6.98 | 6.94 | 7.16 |
| ILMN_1661554 | DIAPH1 | -1.13 | -1.16 | -0.29 | -2.87 | 7.78 | 7.91 | 7.91 | 7.81 | 8.11 |
| ILMN_1754864 | SLC25A18 | -1.13 | -1.74 | -1.87 | -4.24 | 6.84 | 7.01 | 7.10 | 7.12 | 7.49 |
| ILMN_1810392 | ZNHIT2 | -1.13 | -0.74 | -0.98 | -3.51 | 8.51 | 8.64 | 8.59 | 8.62 | 8.90 |
| ILMN_1772207 | LOC653377 | -1.12 | 3.29 | 1.16 | 4.13 | 9.13 | 9.23 | 8.86 | 9.04 | 8.79 |
| ILMN_2060115 | SORL1 | -1.12 | -1.53 | -1.46 | -4.39 | 6.82 | 6.94 | 6.98 | 6.98 | 7.29 |
| ILMN_1757847 | C11orf68 | -1.12 | -0.79 | -0.82 | -3.03 | 7.94 | 8.05 | 8.02 | 8.02 | 8.25 |
| ILMN_1724897 | C14orf93 | -1.12 | -1.15 | -0.03 | 3.89 | 8.28 | 8.40 | 8.41 | 8.29 | 7.87 |
| ILMN_1809040 | LDLRAP1 | -1.12 | -2.59 | -1.12 | -3.82 | 6.99 | 7.06 | 7.16 | 7.06 | 7.24 |
| ILMN_1660880 | RNH1 | -1.12 | -0.43 | -2.30 | -5.00 | 7.53 | 7.64 | 7.57 | 7.76 | 8.04 |
| ILMN_2282077 | MIB2 | -1.12 | -1.16 | -0.94 | -4.35 | 9.05 | 9.23 | 9.24 | 9.20 | 9.77 |
| ILMN_1814985 | PDLIM7 | -1.11 | 0.24 | -1.88 | -5.18 | 7.04 | 7.15 | 7.02 | 7.22 | 7.54 |
| ILMN_1665428 | GSDMD | -1.11 | -0.96 | -1.38 | -3.34 | 8.11 | 8.24 | 8.22 | 8.27 | 8.48 |
| ILMN_1701875 | ZYX | -1.11 | 0.51 | -0.53 | -3.06 | 8.35 | 8.55 | 8.26 | 8.45 | 8.90 |
| ILMN_2327346 | SSBP4 | -1.11 | -2.33 | -1.80 | -4.13 | 7.79 | 7.92 | 8.06 | 8.00 | 8.27 |
| ILMN_1680403 | SSR4 | -1.10 | -0.72 | -0.97 | -3.62 | 10.74 | 10.87 | 10.83 | 10.86 | 11.19 |
| ILMN_1781623 | TEX264 | -1.10 | 0.00 | 0.43 | -3.60 | 7.75 | 7.84 | 7.75 | 7.71 | 8.05 |
| ILMN_1772627 | D4S234E | -1.10 | 1.04 | 1.20 | -7.68 | 6.76 | 6.83 | 6.69 | 6.68 | 7.25 |
| ILMN_1838863 |  | -1.09 | -0.85 | -2.33 | -3.32 | 8.81 | 8.96 | 8.92 | 9.13 | 9.26 |
| ILMN_2408796 | C19orf28 | -1.09 | -0.52 | -2.12 | -3.93 | 6.98 | 7.05 | 7.01 | 7.11 | 7.23 |
| ILMN_1659926 | ST7L | -1.09 | -0.16 | 0.52 | -3.47 | 6.82 | 6.89 | 6.83 | 6.79 | 7.05 |
| ILMN_1808148 | SMCHD1 | -1.09 | -0.33 | -0.86 | -2.73 | 7.20 | 7.34 | 7.24 | 7.31 | 7.56 |
| ILMN_1711023 | CDK5RAP1 | -1.09 | 0.04 | 0.49 | -2.85 | 10.17 | 10.27 | 10.17 | 10.13 | 10.42 |
| ILMN_1737738 | NDUFA12 | -1.08 | -0.54 | -0.10 | -4.47 | 11.03 | 11.14 | 11.09 | 11.04 | 11.47 |
| ILMN_1657771 | CRTC2 | -1.08 | -1.78 | -1.59 | -4.59 | 7.60 | 7.72 | 7.79 | 7.77 | 8.08 |
| ILMN_1672571 | TFIP11 | -1.08 | -0.04 | -0.29 | -2.99 | 8.63 | 8.73 | 8.63 | 8.65 | 8.90 |
| ILMN_1752802 | CLPTM1L | -1.08 | -1.42 | -2.10 | -3.52 | 9.41 | 9.53 | 9.57 | 9.65 | 9.82 |
| ILMN_1772521 | MTHFD1L | -1.08 | -2.16 | -1.87 | -3.46 | 8.70 | 8.80 | 8.90 | 8.87 | 9.02 |
| ILMN_1658677 | DTX3 | -1.08 | -4.41 | -2.57 | -5.00 | 6.89 | 7.03 | 7.46 | 7.22 | 7.54 |
| ILMN_2410771 | KEAP1 | -1.07 | -1.31 | -1.63 | -3.10 | 8.47 | 8.56 | 8.58 | 8.60 | 8.73 |
| ILMN_1882047 |  | -1.07 | -2.27 | -2.22 | -2.95 | 6.68 | 6.76 | 6.85 | 6.85 | 6.91 |
| ILMN_1739083 | SIRT1 | -1.07 | -0.72 | 0.09 | 3.50 | 9.54 | 9.63 | 9.60 | 9.54 | 9.27 |
| ILMN_1764321 | ACOT4 | -1.06 | -1.45 | -1.23 | -3.26 | 6.68 | 6.79 | 6.83 | 6.81 | 7.02 |
| ILMN_1792176 | TADA3 | -1.06 | -0.66 | -0.11 | -3.63 | 7.53 | 7.62 | 7.59 | 7.54 | 7.86 |
| ILMN_2044645 | CGB1 | -1.06 | 0.11 | -2.24 | -3.97 | 6.73 | 6.79 | 6.72 | 6.86 | 6.97 |
| ILMN_1910948 |  | -1.06 | 0.93 | -0.88 | -5.92 | 6.98 | 7.06 | 6.91 | 7.05 | 7.43 |
| ILMN_3236061 | ZNF783 | -1.06 | -2.36 | -1.09 | -7.18 | 6.72 | 6.83 | 6.98 | 6.84 | 7.51 |
| ILMN_1792672 | POLR2D | -1.06 | 0.24 | -0.53 | -2.89 | 9.26 | 9.43 | 9.22 | 9.35 | 9.73 |
| ILMN_2328224 | MADD | -1.06 | -1.27 | -1.20 | -3.88 | 7.30 | 7.40 | 7.42 | 7.42 | 7.67 |
| ILMN_1762316 | CPSF3L | -1.06 | -2.12 | -0.78 | -3.49 | 8.43 | 8.54 | 8.65 | 8.51 | 8.80 |
| ILMN_1795218 | DHX30 | -1.06 | -3.41 | -2.15 | -4.56 | 9.06 | 9.18 | 9.45 | 9.31 | 9.58 |
| ILMN_1756590 | SYS1 | -1.06 | -1.72 | -2.51 | -4.25 | 7.71 | 7.91 | 8.04 | 8.19 | 8.53 |
| ILMN_2150802 | FLJ22795 | -1.05 | -0.66 | -1.11 | 5.24 | 8.37 | 8.48 | 8.44 | 8.49 | 7.79 |
| ILMN_1802519 | VPS36 | -1.05 | -1.41 | -1.88 | -2.78 | 10.34 | 10.50 | 10.55 | 10.62 | 10.75 |
| ILMN_1756793 | POLS | -1.05 | 2.03 | 1.93 | 3.45 | 8.46 | 8.60 | 8.18 | 8.19 | 7.99 |
| ILMN_1796663 | B4GALNT4 | -1.05 | -0.55 | -0.38 | -2.98 | 6.76 | 6.85 | 6.81 | 6.79 | 7.00 |
| ILMN_1794692 | DNMT3B | -1.05 | -0.02 | 0.94 | -4.02 | 7.70 | 7.86 | 7.70 | 7.56 | 8.30 |
| ILMN_2231299 | SERHL2 | -1.05 | 1.17 | 0.22 | 3.09 | 7.45 | 7.65 | 7.22 | 7.41 | 6.85 |
| ILMN_1726107 | UBE2V1 | -1.04 | 0.30 | -1.64 | -3.61 | 7.30 | 7.39 | 7.27 | 7.45 | 7.62 |
| ILMN_3215381 | LOC645175 | -1.04 | -2.00 | -1.37 | -2.83 | 7.76 | 7.84 | 7.92 | 7.87 | 7.98 |
| ILMN_1700001 | TCTA | -1.04 | -1.29 | -1.04 | -3.12 | 7.50 | 7.61 | 7.64 | 7.61 | 7.84 |
| ILMN_1814247 | TCFL5 | -1.04 | -0.70 | -1.82 | -7.18 | 8.75 | 8.87 | 8.83 | 8.97 | 9.60 |
| ILMN_2198893 | LOC407835 | -1.04 | -0.82 | -1.86 | -2.84 | 8.69 | 8.82 | 8.79 | 8.92 | 9.05 |
| ILMN_1744604 | CYBA | -1.04 | 0.95 | -0.92 | 3.05 | 11.84 | 11.96 | 11.74 | 11.95 | 11.50 |
| ILMN_1761820 | EDARADD | -1.03 | -0.59 | -1.14 | -2.80 | 6.85 | 6.94 | 6.90 | 6.95 | 7.09 |
| ILMN_1759341 | MAN2B1 | -1.02 | -1.44 | -1.57 | -8.37 | 7.58 | 7.66 | 7.69 | 7.70 | 8.23 |
| ILMN_1768284 | P2RY8 | -1.01 | -3.26 | -4.43 | -4.12 | 8.23 | 8.34 | 8.59 | 8.71 | 8.68 |
| ILMN_1735180 | NCSTN | -1.01 | -1.01 | -0.51 | -3.34 | 9.73 | 9.88 | 9.88 | 9.80 | 10.21 |
| ILMN_1676631 | CCNO | -1.01 | -1.52 | -1.63 | -5.62 | 6.80 | 6.92 | 6.99 | 7.00 | 7.50 |
| ILMN_3247587 | SLC48A1 | -1.01 | -2.03 | -2.38 | -5.35 | 7.59 | 7.76 | 7.92 | 7.98 | 8.46 |
| ILMN_1718177 | CYP20A1 | -1.01 | 0.12 | 0.81 | -3.28 | 8.09 | 8.17 | 8.08 | 8.02 | 8.36 |
| ILMN_2191929 | C9orf6 | -1.01 | 1.37 | 0.29 | -2.77 | 8.44 | 8.57 | 8.26 | 8.40 | 8.79 |
| ILMN_1750800 | ACO1 | -1.01 | 1.12 | 1.48 | 3.57 | 10.18 | 10.28 | 10.08 | 10.05 | 9.85 |
| ILMN_1887177 |  | -1.01 | -1.93 | -1.72 | -2.81 | 6.88 | 6.96 | 7.03 | 7.02 | 7.10 |
| ILMN_1683678 | SPATS2L | -1.00 | 0.87 | -0.58 | 2.82 | 8.20 | 8.41 | 8.02 | 8.32 | 7.62 |
| ILMN_3305055 | TP63 | -1.00 | -1.49 | -0.72 | -5.56 | 6.80 | 6.93 | 6.99 | 6.89 | 7.52 |
| ILMN_1715131 | CCR7 | -1.00 | -0.35 | 1.83 | -2.73 | 7.59 | 7.76 | 7.65 | 7.28 | 8.05 |
| ILMN_1800341 | WDR66 | -1.00 | 0.26 | 0.78 | -3.22 | 6.83 | 6.91 | 6.81 | 6.77 | 7.08 |
| ILMN_2358474 | TMPRSS3 | -1.00 | -1.51 | -2.29 | -4.82 | 7.01 | 7.16 | 7.24 | 7.36 | 7.75 |
| ILMN_1785405 | SLC17A9 | -0.99 | -1.10 | -2.91 | -5.41 | 7.45 | 7.54 | 7.55 | 7.72 | 7.94 |
| ILMN_2377019 | CORO1B | -0.99 | 0.00 | -2.16 | -3.24 | 7.06 | 7.18 | 7.06 | 7.31 | 7.43 |
| ILMN_2092756 | TMEM109 | -0.99 | -3.89 | -2.15 | -8.39 | 9.17 | 9.25 | 9.49 | 9.35 | 9.85 |
| ILMN_1775939 | SF3B2 | -0.99 | -2.17 | -1.15 | -3.44 | 10.19 | 10.29 | 10.42 | 10.31 | 10.56 |
| ILMN_2298818 | RPS29 | -0.99 | 0.19 | 0.48 | -3.65 | 8.13 | 8.27 | 8.11 | 8.07 | 8.64 |
| ILMN_3300313 | P4HTM | -0.99 | -2.19 | -2.43 | -3.17 | 6.98 | 7.07 | 7.19 | 7.21 | 7.28 |
| ILMN_1712887 | SLC10A3 | -0.99 | -2.56 | -1.61 | -3.54 | 7.05 | 7.12 | 7.24 | 7.17 | 7.31 |
| ILMN_2128128 | SHFM1 | -0.99 | 1.15 | 1.43 | -2.94 | 10.84 | 11.01 | 10.63 | 10.58 | 11.36 |
| ILMN_2304577 | KCNK7 | -0.98 | -1.18 | -2.11 | -6.68 | 6.91 | 6.97 | 6.99 | 7.05 | 7.36 |
| ILMN_3239113 | LOC144438 | -0.98 | -1.15 | -1.18 | -3.26 | 8.78 | 8.89 | 8.91 | 8.92 | 9.16 |
| ILMN_1669966 | NDUFS7 | -0.98 | -1.30 | -2.30 | -3.03 | 10.64 | 10.78 | 10.82 | 10.96 | 11.06 |
| ILMN_2381064 | TPD52 | -0.98 | -2.46 | -2.04 | -5.67 | 8.85 | 8.97 | 9.14 | 9.09 | 9.53 |
| ILMN_1738263 | PIGU | -0.98 | -2.09 | -1.79 | -4.24 | 9.37 | 9.46 | 9.57 | 9.54 | 9.79 |
| ILMN_1849186 |  | -0.98 | -4.31 | -3.73 | -1.82 | 7.89 | 7.99 | 8.32 | 8.26 | 8.07 |
| ILMN_1702837 | PSMD1 | -0.97 | 0.18 | -0.56 | -3.38 | 9.58 | 9.71 | 9.56 | 9.65 | 10.02 |
| ILMN_1654392 | KHNYN | -0.97 | -2.43 | -2.31 | -3.04 | 7.49 | 7.57 | 7.69 | 7.68 | 7.74 |
| ILMN_1694233 | ACYP1 | -0.97 | 0.18 | -0.72 | -3.20 | 9.68 | 9.81 | 9.66 | 9.78 | 10.10 |
| ILMN_1774949 | PIGP | -0.97 | -0.10 | -1.22 | -3.75 | 7.48 | 7.58 | 7.49 | 7.60 | 7.84 |
| ILMN_1779184 | CTU2 | -0.97 | -1.14 | -1.01 | 3.99 | 7.29 | 7.36 | 7.37 | 7.36 | 6.98 |
| ILMN_1690454 | C3orf54 | -0.97 | -1.14 | -2.78 | -3.88 | 7.41 | 7.61 | 7.64 | 7.98 | 8.21 |
| ILMN_2180682 | MTPN | -0.96 | -0.38 | -0.81 | -3.14 | 11.88 | 12.03 | 11.94 | 12.00 | 12.35 |
| ILMN_2252309 | DPP7 | -0.96 | -0.96 | -0.71 | -3.37 | 8.51 | 8.74 | 8.74 | 8.68 | 9.30 |
| ILMN_2399208 | SCAMP3 | -0.96 | 0.24 | -0.64 | -3.92 | 6.96 | 7.04 | 6.94 | 7.01 | 7.28 |
| ILMN_2255579 | RAB37 | -0.96 | -2.35 | -2.49 | -5.04 | 6.72 | 6.78 | 6.88 | 6.89 | 7.07 |
| ILMN_1783771 | UBE2Z | -0.96 | -3.68 | -1.61 | -2.72 | 7.11 | 7.17 | 7.35 | 7.21 | 7.29 |
| ILMN_2393144 | ARL6IP4 | -0.96 | -2.39 | -2.42 | -3.82 | 9.19 | 9.37 | 9.65 | 9.65 | 9.92 |
| ILMN_1791478 | MTPN | -0.95 | -0.85 | -0.37 | -2.82 | 10.23 | 10.38 | 10.37 | 10.29 | 10.68 |
| ILMN_1761159 | ESYT1 | -0.95 | -1.77 | -2.93 | -5.81 | 9.75 | 9.89 | 10.01 | 10.17 | 10.59 |
| ILMN_2191428 | UBB | -0.95 | 1.60 | 3.41 | 4.12 | 13.16 | 13.26 | 12.98 | 12.79 | 12.71 |
| ILMN_1801215 | PDDC1 | -0.95 | -1.37 | -0.42 | -4.64 | 7.23 | 7.32 | 7.35 | 7.27 | 7.64 |
| ILMN_1798270 | C11orf75 | -0.95 | -2.70 | -1.93 | -3.50 | 7.66 | 7.77 | 7.96 | 7.87 | 8.04 |
| ILMN_1673026 | CHCHD3 | -0.95 | -1.18 | -0.97 | -5.07 | 8.54 | 8.66 | 8.69 | 8.66 | 9.18 |
| ILMN_1739259 | UBE4A | -0.94 | -1.37 | -0.80 | -2.74 | 9.21 | 9.28 | 9.31 | 9.27 | 9.40 |
| ILMN_1710873 | ZNF330 | -0.94 | 1.38 | 1.67 | 3.96 | 8.77 | 8.86 | 8.65 | 8.62 | 8.41 |
| ILMN_1700109 | PTOV1 | -0.94 | -0.61 | -0.55 | 4.30 | 9.87 | 9.98 | 9.94 | 9.94 | 9.35 |
| ILMN_1651285 | BCL6B | -0.94 | -2.06 | -1.77 | -7.34 | 6.84 | 6.95 | 7.08 | 7.05 | 7.70 |
| ILMN_2204754 | TMX4 | -0.94 | -4.73 | -3.76 | -1.95 | 8.81 | 8.89 | 9.23 | 9.14 | 8.98 |
| ILMN_1693259 | PDCD6IP | -0.94 | -1.31 | -0.86 | -2.80 | 8.01 | 8.21 | 8.28 | 8.19 | 8.59 |
| ILMN_1768582 | PPP2CB | -0.94 | -0.17 | -1.66 | 3.32 | 8.44 | 8.52 | 8.46 | 8.57 | 8.18 |
| ILMN_1715994 | HGS | -0.93 | -2.62 | -1.35 | -5.42 | 9.49 | 9.58 | 9.73 | 9.61 | 9.98 |
| ILMN_1684158 | GPT2 | -0.93 | -2.52 | -3.86 | 1.63 | 9.52 | 9.68 | 9.95 | 10.18 | 9.24 |
| ILMN_1656378 | NMT2 | -0.93 | -1.58 | -1.98 | 3.14 | 8.68 | 8.82 | 8.91 | 8.97 | 8.23 |
| ILMN_1767475 | CERK | -0.93 | -2.29 | -4.45 | -4.67 | 8.46 | 8.61 | 8.83 | 9.19 | 9.23 |
| ILMN_2376502 | RHOBTB1 | -0.93 | -1.38 | -2.52 | -5.19 | 6.51 | 6.63 | 6.69 | 6.83 | 7.17 |
| ILMN_1673329 | CTAGE6 | -0.93 | 1.85 | 1.47 | 4.91 | 7.61 | 7.69 | 7.45 | 7.48 | 7.18 |
| ILMN_1729915 | PILRA | -0.93 | -1.48 | -1.26 | -3.00 | 7.01 | 7.09 | 7.14 | 7.12 | 7.28 |
| ILMN_1784217 | SOX15 | -0.93 | -0.12 | -1.74 | -3.93 | 6.69 | 6.74 | 6.70 | 6.79 | 6.92 |
| ILMN_1803483 | KIAA2013 | -0.92 | 0.32 | -1.78 | -4.21 | 9.50 | 9.63 | 9.46 | 9.74 | 10.08 |
| ILMN_3243381 | MLEC | -0.92 | -2.83 | -1.46 | -5.27 | 8.45 | 8.55 | 8.75 | 8.61 | 9.01 |
| ILMN_1783798 | GAS8 | -0.92 | -2.35 | -1.38 | 3.49 | 7.13 | 7.20 | 7.30 | 7.23 | 6.88 |
| ILMN_1799614 | PNPLA6 | -0.92 | -1.28 | -1.58 | -3.17 | 8.95 | 9.07 | 9.12 | 9.16 | 9.37 |
| ILMN_1722491 | APRT | -0.91 | 0.53 | -0.28 | 4.19 | 9.90 | 10.04 | 9.82 | 9.94 | 9.24 |
| ILMN_2390318 | CIDEA | -0.91 | -2.95 | -0.86 | -2.73 | 6.67 | 6.73 | 6.85 | 6.72 | 6.84 |
| ILMN_2078599 | ACP5 | -0.91 | -1.16 | -0.60 | -2.73 | 6.72 | 6.82 | 6.84 | 6.78 | 7.01 |
| ILMN_1787256 | HCN3 | -0.91 | -1.87 | -1.20 | -2.96 | 7.36 | 7.48 | 7.60 | 7.51 | 7.74 |
| ILMN_1715745 | MCART6 | -0.91 | 1.63 | 2.34 | 2.77 | 6.81 | 6.87 | 6.70 | 6.65 | 6.62 |
| ILMN_1709237 | EPHX2 | -0.91 | 0.38 | -0.29 | -5.72 | 7.32 | 7.38 | 7.29 | 7.34 | 7.72 |
| ILMN_1691572 | TST | -0.90 | 0.85 | -1.57 | 4.52 | 10.18 | 10.35 | 10.02 | 10.47 | 9.35 |
| ILMN_2212909 | MELK | -0.90 | 3.70 | 0.59 | 4.61 | 10.45 | 10.56 | 10.01 | 10.38 | 9.91 |
| ILMN_1770977 | TMEM134 | -0.90 | -2.32 | -3.05 | -9.08 | 8.54 | 8.61 | 8.73 | 8.79 | 9.28 |
| ILMN_1742578 | MKLN1 | -0.89 | -0.98 | -0.09 | -2.95 | 8.81 | 8.91 | 8.92 | 8.82 | 9.14 |
| ILMN_1802843 | PRCC | -0.89 | -1.36 | -1.32 | -3.70 | 8.24 | 8.31 | 8.35 | 8.34 | 8.53 |
| ILMN_1803211 | FBXO2 | -0.89 | -0.58 | -0.99 | -4.45 | 6.96 | 7.05 | 7.02 | 7.06 | 7.40 |
| ILMN_3256325 | CYB561D1 | -0.89 | -2.22 | -1.54 | -5.30 | 8.37 | 8.47 | 8.62 | 8.55 | 8.97 |
| ILMN_1742382 | RIMS3 | -0.89 | -3.11 | -0.75 | -2.74 | 8.21 | 8.40 | 8.87 | 8.37 | 8.79 |
| ILMN_3235326 | LOC388796 | -0.89 | -0.89 | -0.26 | -3.92 | 9.17 | 9.32 | 9.32 | 9.21 | 9.83 |
| ILMN_2163819 | KIF21B | -0.89 | -2.09 | -3.54 | -3.03 | 7.49 | 7.59 | 7.73 | 7.90 | 7.84 |
| ILMN_1664153 | SLC30A5 | -0.89 | -0.06 | -1.13 | -3.05 | 8.19 | 8.29 | 8.20 | 8.31 | 8.52 |
| ILMN_3239775 | ODZ4 | -0.89 | 0.27 | -2.10 | 2.80 | 7.89 | 8.05 | 7.84 | 8.27 | 7.37 |
| ILMN_1712269 | ZNF605 | -0.89 | -1.50 | -0.42 | -3.47 | 7.07 | 7.14 | 7.20 | 7.10 | 7.37 |
| ILMN_1689704 | TMEM5 | -0.88 | 1.07 | 0.97 | -6.55 | 8.88 | 8.98 | 8.77 | 8.78 | 9.60 |
| ILMN_1689160 | DPEP2 | -0.88 | -0.96 | 0.20 | -2.81 | 7.06 | 7.13 | 7.14 | 7.04 | 7.29 |
| ILMN_2183409 | SCARB1 | -0.88 | -3.16 | -4.55 | -5.97 | 8.16 | 8.25 | 8.47 | 8.61 | 8.75 |
| ILMN_2319910 | DGKA | -0.88 | -1.30 | -0.04 | -4.41 | 7.27 | 7.35 | 7.38 | 7.27 | 7.65 |
| ILMN_3182069 | LOC100129697 | -0.88 | 1.55 | -0.11 | 3.27 | 7.08 | 7.16 | 6.93 | 7.09 | 6.77 |
| ILMN_1809139 | AHCTF1 | -0.88 | -0.44 | -0.03 | -4.02 | 8.66 | 8.75 | 8.70 | 8.66 | 9.06 |
| ILMN_2297710 | PLEKHB2 | -0.88 | -1.47 | -2.89 | -2.95 | 7.35 | 7.53 | 7.66 | 7.96 | 7.97 |
| ILMN_1659913 | ISG20 | -0.88 | 0.24 | -0.50 | 4.26 | 10.49 | 10.64 | 10.44 | 10.58 | 9.73 |
| ILMN_1667319 | LPPR2 | -0.87 | -1.49 | -0.77 | -3.39 | 6.85 | 6.91 | 6.95 | 6.90 | 7.07 |
| ILMN_2333367 | FKBP1A | -0.87 | -2.23 | -3.24 | -4.16 | 10.05 | 10.18 | 10.38 | 10.53 | 10.67 |
| ILMN_1674236 | HSPB1 | -0.87 | 0.89 | 0.65 | 3.23 | 11.22 | 11.47 | 10.97 | 11.04 | 10.30 |
| ILMN_2123730 | TBC1D22B | -0.87 | -2.61 | -0.06 | -3.94 | 7.55 | 7.60 | 7.70 | 7.55 | 7.77 |
| ILMN_1756999 | RBL2 | -0.87 | -1.13 | -0.19 | 4.02 | 8.63 | 8.72 | 8.74 | 8.65 | 8.21 |
| ILMN_3243244 | SNORD80 | -0.87 | -1.17 | 0.00 | -3.06 | 8.08 | 8.19 | 8.23 | 8.08 | 8.46 |
| ILMN_1730670 | FSTL3 | -0.87 | -1.24 | -0.81 | -5.25 | 6.84 | 6.89 | 6.92 | 6.89 | 7.16 |
| ILMN_2401878 | DUSP10 | -0.86 | 0.08 | -0.37 | -3.09 | 6.80 | 6.87 | 6.79 | 6.83 | 7.05 |
| ILMN_1660639 | TSHR | -0.86 | -2.03 | -1.08 | -6.82 | 6.85 | 6.92 | 7.01 | 6.93 | 7.38 |
| ILMN_1712430 | ATP5G1 | -0.86 | 1.52 | 1.62 | -3.96 | 7.81 | 7.92 | 7.61 | 7.60 | 8.33 |
| ILMN_1786718 | NDUFV1 | -0.86 | -0.48 | -0.72 | -3.52 | 10.53 | 10.61 | 10.57 | 10.60 | 10.87 |
| ILMN_1742577 | GTPBP4 | -0.86 | 1.05 | 3.05 | 3.33 | 11.88 | 11.94 | 11.81 | 11.67 | 11.65 |
| ILMN_1696420 | BRD7 | -0.86 | -1.35 | 0.53 | 6.84 | 9.35 | 9.43 | 9.47 | 9.30 | 8.71 |
| ILMN_1679520 | AGPAT1 | -0.85 | -0.11 | -1.32 | -3.56 | 8.00 | 8.08 | 8.01 | 8.13 | 8.34 |
| ILMN_1745112 | FAM102A | -0.85 | -1.63 | -2.17 | -3.96 | 6.84 | 6.92 | 6.98 | 7.03 | 7.18 |
| ILMN_1795937 | VIL2 | -0.85 | -1.26 | 0.63 | -2.89 | 11.25 | 11.33 | 11.36 | 11.19 | 11.51 |
| ILMN_1738237 | HS1BP3 | -0.85 | -1.62 | -2.02 | -3.74 | 7.55 | 7.68 | 7.80 | 7.87 | 8.14 |
| ILMN_1698185 | WDR90 | -0.85 | -2.19 | -1.40 | -4.58 | 7.14 | 7.24 | 7.38 | 7.30 | 7.65 |
| ILMN_1743021 | CAMKK2 | -0.85 | -0.27 | -0.96 | -4.80 | 7.42 | 7.52 | 7.45 | 7.54 | 8.00 |
| ILMN_2337928 | CXCR5 | -0.85 | -1.25 | -1.05 | -7.71 | 8.91 | 9.03 | 9.08 | 9.05 | 9.94 |
| ILMN_2101025 | ZNF75D | -0.84 | -0.66 | -0.66 | -3.05 | 7.08 | 7.14 | 7.13 | 7.13 | 7.31 |
| ILMN_1770865 | TRIM46 | -0.84 | -0.91 | -1.14 | -3.99 | 7.13 | 7.21 | 7.22 | 7.24 | 7.52 |
| ILMN_1653599 | ATP5D | -0.84 | 0.28 | -1.08 | -3.35 | 11.43 | 11.50 | 11.40 | 11.52 | 11.71 |
| ILMN_2099783 | ATP6V1F | -0.84 | -1.72 | -1.33 | -5.12 | 10.07 | 10.18 | 10.30 | 10.25 | 10.76 |
| ILMN_1912737 |  | -0.83 | -0.30 | 0.38 | -5.51 | 6.87 | 6.98 | 6.91 | 6.82 | 7.60 |
| ILMN_1803194 | GALK1 | -0.83 | -0.59 | -2.00 | -2.81 | 10.45 | 10.57 | 10.54 | 10.73 | 10.85 |
| ILMN_1756705 | CHTF18 | -0.83 | -3.16 | -3.06 | -3.69 | 7.77 | 7.85 | 8.09 | 8.08 | 8.14 |
| ILMN_1736077 | LIAS | -0.83 | 2.36 | 1.45 | 3.22 | 7.89 | 8.00 | 7.60 | 7.71 | 7.49 |
| ILMN_2310253 | TARBP2 | -0.83 | -2.79 | -3.54 | -7.34 | 7.30 | 7.36 | 7.51 | 7.57 | 7.86 |
| ILMN_1860084 |  | -0.83 | -0.95 | -2.16 | -4.17 | 7.55 | 7.64 | 7.65 | 7.79 | 8.02 |
| ILMN_1906437 |  | -0.83 | -0.75 | -1.63 | -3.29 | 7.23 | 7.33 | 7.32 | 7.43 | 7.63 |
| ILMN_1724708 | RAB33A | -0.83 | -1.24 | -2.43 | -3.77 | 6.65 | 6.70 | 6.73 | 6.80 | 6.89 |
| ILMN_2338323 | CDC25B | -0.83 | -0.63 | -1.02 | 4.92 | 11.64 | 11.72 | 11.70 | 11.74 | 11.14 |
| ILMN_1752988 | C11orf17 | -0.83 | -1.03 | 0.23 | -3.20 | 8.28 | 8.39 | 8.42 | 8.25 | 8.71 |
| ILMN_1742958 | UBE2C | -0.82 | -1.05 | -0.77 | -3.19 | 6.65 | 6.71 | 6.72 | 6.70 | 6.88 |
| ILMN_2069632 | GTSF1 | -0.82 | 1.23 | 0.58 | -2.84 | 10.90 | 11.03 | 10.71 | 10.81 | 11.34 |
| ILMN_2133675 | SGSH | -0.81 | -1.37 | 0.00 | -5.83 | 6.77 | 6.85 | 6.91 | 6.77 | 7.39 |
| ILMN_2332964 | LGMN | -0.80 | 0.85 | -0.44 | -2.85 | 7.65 | 7.77 | 7.53 | 7.71 | 8.06 |
| ILMN_1656792 | FLYWCH1 | -0.80 | -1.02 | -0.70 | -3.70 | 6.92 | 6.97 | 6.98 | 6.96 | 7.15 |
| ILMN_2314007 | TCF12 | -0.80 | 0.53 | -0.76 | 2.91 | 9.98 | 10.09 | 9.90 | 10.09 | 9.55 |
| ILMN_1661695 | IRAK3 | -0.80 | -1.59 | -2.08 | -4.99 | 7.18 | 7.33 | 7.47 | 7.56 | 8.10 |
| ILMN_2396571 | GPR108 | -0.80 | -0.80 | -1.38 | -3.30 | 7.82 | 7.90 | 7.90 | 7.96 | 8.16 |
| ILMN_1777397 | MSX1 | -0.80 | -2.68 | -4.51 | -1.49 | 8.56 | 8.69 | 8.98 | 9.26 | 8.79 |
| ILMN_1740351 | KIAA0174 | -0.80 | -0.76 | 0.84 | 7.68 | 10.82 | 10.89 | 10.88 | 10.75 | 10.18 |
| ILMN_1703430 | FLJ10374 | -0.79 | -1.99 | -2.36 | -3.05 | 8.70 | 8.81 | 8.98 | 9.03 | 9.13 |
| ILMN_2058251 | VIM | -0.79 | 0.69 | -1.55 | 5.54 | 10.85 | 10.93 | 10.78 | 11.01 | 10.29 |
| ILMN_2345837 | CLTA | -0.79 | 1.15 | 2.33 | 4.46 | 10.86 | 10.93 | 10.76 | 10.66 | 10.48 |
| ILMN_1669832 | TCF12 | -0.79 | 0.35 | 0.16 | 4.06 | 9.96 | 10.06 | 9.92 | 9.94 | 9.47 |
| ILMN_2141790 | HYOU1 | -0.79 | -0.51 | -1.18 | -4.61 | 7.45 | 7.51 | 7.49 | 7.55 | 7.84 |
| ILMN_3307799 | PSMD4 | -0.79 | 0.20 | 0.17 | -3.54 | 9.27 | 9.39 | 9.24 | 9.24 | 9.81 |
| ILMN_1731287 | ARFGAP3 | -0.79 | -0.07 | -2.22 | -5.23 | 7.57 | 7.65 | 7.58 | 7.79 | 8.08 |
| ILMN_2195957 | RHOBTB2 | -0.78 | -1.49 | -2.83 | -3.58 | 7.40 | 7.47 | 7.54 | 7.66 | 7.72 |
| ILMN_1790781 | DHRS13 | -0.78 | -1.78 | -0.53 | -3.67 | 7.40 | 7.48 | 7.57 | 7.45 | 7.75 |
| ILMN_1754894 | C1orf162 | -0.78 | -0.92 | 0.69 | -4.69 | 7.20 | 7.26 | 7.27 | 7.15 | 7.54 |
| ILMN_2365023 | C9orf24 | -0.78 | -1.09 | -1.46 | -2.96 | 6.90 | 6.99 | 7.02 | 7.06 | 7.22 |
| ILMN_2221046 | GM2A | -0.78 | -0.89 | 1.20 | -2.98 | 8.13 | 8.29 | 8.32 | 7.87 | 8.76 |
| ILMN_1706886 | BCL7A | -0.78 | -2.15 | -2.58 | -2.93 | 8.09 | 8.19 | 8.36 | 8.41 | 8.46 |
| ILMN_1771689 | EXD2 | -0.77 | -2.60 | -2.45 | -5.34 | 7.06 | 7.17 | 7.41 | 7.39 | 7.78 |
| ILMN_1739967 | TBK1 | -0.77 | 0.19 | 0.16 | -3.79 | 9.68 | 9.77 | 9.65 | 9.66 | 10.15 |
| ILMN_1711048 | ZBTB17 | -0.77 | -1.93 | 0.21 | -3.39 | 7.90 | 7.98 | 8.11 | 7.87 | 8.28 |
| ILMN_1768488 | TERF2 | -0.77 | 0.18 | 0.38 | 4.24 | 9.53 | 9.65 | 9.51 | 9.48 | 8.89 |
| ILMN_2131336 | TMEM194 | -0.77 | 0.00 | -0.45 | -3.09 | 9.60 | 9.69 | 9.60 | 9.65 | 9.95 |
| ILMN_1707051 | NFATC1 | -0.77 | -1.85 | -1.40 | -3.20 | 8.06 | 8.21 | 8.42 | 8.33 | 8.68 |
| ILMN_2045911 | FBXO28 | -0.77 | 1.98 | 2.27 | 3.00 | 9.15 | 9.21 | 8.98 | 8.96 | 8.90 |
| ILMN_1811373 | FAM20B | -0.77 | -1.35 | -0.90 | -4.23 | 8.66 | 8.74 | 8.80 | 8.76 | 9.10 |
| ILMN_1785660 | SRPR | -0.77 | -0.38 | -0.16 | -3.19 | 7.50 | 7.58 | 7.54 | 7.51 | 7.83 |
| ILMN_2243553 | ZNF275 | -0.76 | -1.78 | -1.82 | -3.51 | 8.17 | 8.25 | 8.35 | 8.36 | 8.53 |
| ILMN_2116714 | SLC39A1 | -0.76 | -0.24 | -0.71 | -3.63 | 9.34 | 9.43 | 9.37 | 9.43 | 9.80 |
| ILMN_1680091 | POP7 | -0.76 | -0.84 | -2.36 | -5.53 | 8.79 | 8.85 | 8.86 | 8.99 | 9.25 |
| ILMN_1687284 | USP12 | -0.76 | 0.38 | -0.48 | 2.81 | 6.81 | 6.86 | 6.78 | 6.84 | 6.61 |
| ILMN_1777342 | PREX1 | -0.76 | -2.95 | -1.67 | -6.76 | 7.72 | 7.81 | 8.06 | 7.91 | 8.49 |
| ILMN_1701731 | AKR1B1 | -0.76 | -1.31 | -1.07 | -4.34 | 10.17 | 10.30 | 10.40 | 10.36 | 10.95 |
| ILMN_1729749 | HERC5 | -0.75 | 0.82 | 0.48 | 3.76 | 9.65 | 9.89 | 9.40 | 9.50 | 8.47 |
| ILMN_1729767 | TARBP2 | -0.75 | -2.23 | -1.01 | -4.30 | 7.61 | 7.71 | 7.90 | 7.74 | 8.16 |
| ILMN_1662038 | LARGE | -0.75 | -0.36 | -2.46 | 3.80 | 7.79 | 7.93 | 7.86 | 8.25 | 7.08 |
| ILMN_2142815 | RPL35 | -0.75 | -0.24 | -1.94 | -2.93 | 13.30 | 13.38 | 13.32 | 13.51 | 13.62 |
| ILMN_1757343 | PABPC4 | -0.75 | -1.85 | -1.33 | -4.09 | 10.16 | 10.24 | 10.35 | 10.30 | 10.58 |
| ILMN_1801909 | IGHMBP2 | -0.75 | -1.45 | 0.04 | -3.20 | 6.92 | 6.98 | 7.03 | 6.92 | 7.16 |
| ILMN_1745860 | FLJ40852 | -0.74 | -1.11 | -1.11 | -3.25 | 6.88 | 6.93 | 6.96 | 6.96 | 7.11 |
| ILMN_1677953 | OGFOD1 | -0.74 | 0.41 | 0.24 | 4.17 | 9.55 | 9.65 | 9.49 | 9.51 | 8.97 |
| ILMN_1813846 | P2RX4 | -0.74 | -1.62 | -1.92 | -5.09 | 7.08 | 7.14 | 7.22 | 7.25 | 7.54 |
| ILMN_1726659 | THOP1 | -0.74 | -4.19 | -4.59 | -3.51 | 8.95 | 9.04 | 9.45 | 9.49 | 9.37 |
| ILMN_1765146 | IFNAR2 | -0.73 | -0.37 | 0.04 | -3.00 | 8.70 | 8.82 | 8.76 | 8.70 | 9.17 |
| ILMN_1777660 | RNF144 | -0.73 | -1.42 | -1.60 | -6.14 | 6.77 | 6.83 | 6.88 | 6.89 | 7.22 |
| ILMN_1691506 | NGRN | -0.73 | 1.26 | 0.90 | 4.15 | 11.29 | 11.35 | 11.18 | 11.21 | 10.95 |
| ILMN_1711988 | KCNK12 | -0.73 | -1.41 | -1.19 | -5.33 | 7.63 | 7.72 | 7.81 | 7.78 | 8.29 |
| ILMN_1765880 | C16orf57 | -0.73 | -0.87 | -0.79 | 3.99 | 8.94 | 9.03 | 9.05 | 9.04 | 8.47 |
| ILMN_1660812 | C14orf11 | -0.72 | 1.54 | 1.58 | 3.48 | 7.14 | 7.19 | 7.02 | 7.02 | 6.88 |
| ILMN_1749403 | TSPAN33 | -0.72 | -1.63 | -1.37 | -3.69 | 8.50 | 8.60 | 8.71 | 8.68 | 8.98 |
| ILMN_1728426 | INPPL1 | -0.72 | -3.36 | -3.55 | -3.39 | 8.33 | 8.41 | 8.69 | 8.71 | 8.69 |
| ILMN_1782635 | YARS2 | -0.72 | 0.54 | -0.11 | -4.58 | 8.72 | 8.78 | 8.67 | 8.73 | 9.14 |
| ILMN_1746025 | MINA | -0.72 | 1.34 | 1.41 | 3.05 | 7.70 | 7.77 | 7.57 | 7.57 | 7.41 |
| ILMN_2397846 | SNCB | -0.72 | -0.81 | -0.40 | -3.27 | 6.60 | 6.65 | 6.66 | 6.63 | 6.84 |
| ILMN_1745152 | UQCC | -0.72 | -0.07 | 0.24 | -3.38 | 9.99 | 10.06 | 10.00 | 9.97 | 10.32 |
| ILMN_2147114 | PSPH | -0.71 | 0.00 | -0.09 | 2.80 | 7.42 | 7.50 | 7.42 | 7.43 | 7.10 |
| ILMN_3234735 | ERI2 | -0.71 | 1.38 | 1.46 | 6.25 | 8.23 | 8.29 | 8.12 | 8.11 | 7.71 |
| ILMN_1727288 | EVPL | -0.71 | -0.67 | -0.32 | -3.28 | 6.75 | 6.81 | 6.80 | 6.77 | 7.02 |
| ILMN_1804332 | GPR137 | -0.71 | 0.25 | 0.71 | -3.04 | 9.38 | 9.49 | 9.35 | 9.28 | 9.83 |
| ILMN_1771482 | KIAA1324 | -0.71 | -2.75 | -2.27 | -3.74 | 7.62 | 7.72 | 7.99 | 7.92 | 8.12 |
| ILMN_3300358 | ZNF84 | -0.71 | -2.91 | -1.91 | -3.71 | 8.17 | 8.25 | 8.50 | 8.38 | 8.59 |
| ILMN_3295419 | LOC644363 | -0.71 | -0.89 | -0.37 | 3.27 | 8.13 | 8.19 | 8.21 | 8.16 | 7.84 |
| ILMN_2337058 | PORCN | -0.70 | 0.43 | -1.41 | -4.97 | 6.84 | 6.90 | 6.80 | 6.96 | 7.26 |
| ILMN_1666706 | LOC645676 | -0.70 | -0.07 | -0.07 | -3.04 | 7.13 | 7.19 | 7.13 | 7.13 | 7.40 |
| ILMN_1781816 | CHD6 | -0.70 | -1.84 | -0.70 | -2.80 | 7.41 | 7.47 | 7.57 | 7.47 | 7.66 |
| ILMN_1784822 | PPP1R3F | -0.70 | -0.12 | -1.78 | -3.08 | 7.80 | 7.90 | 7.82 | 8.05 | 8.23 |
| ILMN_1788017 | HSH2D | -0.70 | 0.23 | -1.68 | 2.73 | 7.76 | 7.88 | 7.72 | 8.05 | 7.27 |
| ILMN_3243471 | CNPY2 | -0.69 | 0.16 | 0.51 | -3.74 | 10.19 | 10.27 | 10.17 | 10.12 | 10.65 |
| ILMN_1675956 | LYST | -0.69 | 1.24 | 0.80 | 3.85 | 7.72 | 7.80 | 7.57 | 7.62 | 7.27 |
| ILMN_1795464 | LTA | -0.69 | 0.80 | 2.35 | 2.79 | 7.25 | 7.31 | 7.18 | 7.03 | 6.99 |
| ILMN_1726589 | CD248 | -0.69 | -0.82 | -1.17 | -7.47 | 7.38 | 7.45 | 7.46 | 7.50 | 8.11 |
| ILMN_1671906 | MND1 | -0.68 | 1.51 | -0.11 | 4.14 | 10.54 | 10.62 | 10.36 | 10.55 | 10.05 |
| ILMN_1685104 | C9orf6 | -0.68 | 0.95 | 1.06 | -2.88 | 8.04 | 8.12 | 7.92 | 7.91 | 8.39 |
| ILMN_1733781 | FSD1 | -0.68 | -1.32 | -0.34 | -3.39 | 6.93 | 6.99 | 7.05 | 6.96 | 7.23 |
| ILMN_1715543 | ACOT1 | -0.68 | -1.56 | -1.17 | -3.75 | 6.60 | 6.69 | 6.80 | 6.75 | 7.08 |
| ILMN_1670723 | MSL3 | -0.68 | 0.49 | -0.83 | 4.99 | 9.43 | 9.49 | 9.39 | 9.51 | 8.99 |
| ILMN_1790778 | PNMA2 | -0.67 | -0.38 | -0.65 | 6.52 | 10.15 | 10.25 | 10.21 | 10.25 | 9.17 |
| ILMN_1717334 | VAV1 | -0.66 | -2.49 | -3.72 | -4.15 | 7.85 | 7.92 | 8.10 | 8.23 | 8.27 |
| ILMN_3180557 | CYB561D1 | -0.66 | -1.29 | -1.82 | -5.27 | 7.21 | 7.28 | 7.34 | 7.39 | 7.74 |
| ILMN_1803590 | ZFC3H1 | -0.66 | -0.52 | -0.73 | -3.09 | 8.32 | 8.38 | 8.37 | 8.39 | 8.61 |
| ILMN_1692123 | ELOVL3 | -0.65 | -0.75 | -0.65 | -3.13 | 6.66 | 6.70 | 6.71 | 6.70 | 6.88 |
| ILMN_1736096 | DLL3 | -0.65 | -2.73 | -4.21 | -8.15 | 8.57 | 8.64 | 8.87 | 9.03 | 9.45 |
| ILMN_2413572 | MARK2 | -0.65 | -0.97 | -2.98 | -3.28 | 7.63 | 7.70 | 7.73 | 7.94 | 7.97 |
| ILMN_2402806 | TRPC4AP | -0.64 | -2.81 | -1.32 | -4.00 | 8.32 | 8.39 | 8.61 | 8.46 | 8.73 |
| ILMN_1670752 | KIAA0907 | -0.64 | -1.26 | -0.50 | -3.58 | 10.42 | 10.50 | 10.57 | 10.48 | 10.83 |
| ILMN_2084353 | M6PR | -0.64 | -0.88 | -0.72 | -4.43 | 8.34 | 8.45 | 8.49 | 8.46 | 9.10 |
| ILMN_1665219 | LTBP4 | -0.64 | -0.85 | -2.18 | -3.42 | 7.13 | 7.19 | 7.20 | 7.31 | 7.42 |
| ILMN_1720027 | ZNF23 | -0.64 | 1.29 | 1.23 | 2.97 | 8.01 | 8.09 | 7.86 | 7.87 | 7.66 |
| ILMN_3204842 | LOC644525 | -0.64 | -0.40 | 0.10 | -2.82 | 6.70 | 6.74 | 6.73 | 6.69 | 6.89 |
| ILMN_1696065 | SDF4 | -0.64 | -1.37 | -0.44 | -3.66 | 8.47 | 8.57 | 8.68 | 8.54 | 9.02 |
| ILMN_1712095 | FOXO4 | -0.64 | 0.41 | -1.81 | 2.83 | 8.27 | 8.36 | 8.22 | 8.51 | 7.90 |
| ILMN_3226769 | LOC730074 | -0.64 | 0.05 | -0.94 | -2.85 | 7.25 | 7.34 | 7.24 | 7.38 | 7.65 |
| ILMN_2131381 | PDE3B | -0.64 | -0.22 | -1.33 | -3.18 | 7.37 | 7.44 | 7.40 | 7.51 | 7.71 |
| ILMN_1873540 |  | -0.63 | -0.91 | -1.70 | -2.86 | 7.12 | 7.19 | 7.23 | 7.32 | 7.46 |
| ILMN_1684407 | LOC440509 | -0.63 | 1.85 | 0.19 | 3.55 | 6.91 | 6.95 | 6.78 | 6.90 | 6.67 |
| ILMN_1784269 | AASDH | -0.63 | 1.18 | 2.65 | 5.01 | 8.32 | 8.37 | 8.23 | 8.11 | 7.93 |
| ILMN_1718766 | MT1F | -0.63 | 0.56 | 3.06 | 4.16 | 10.20 | 10.38 | 10.04 | 9.32 | 9.01 |
| ILMN_1811121 | CXorf39 | -0.62 | 1.45 | 0.45 | 3.80 | 8.04 | 8.11 | 7.87 | 7.98 | 7.59 |
| ILMN_1772651 | CNOT2 | -0.62 | -0.22 | 0.24 | -3.36 | 9.12 | 9.22 | 9.16 | 9.09 | 9.63 |
| ILMN_1788268 | NAPG | -0.62 | 0.28 | -0.24 | -2.77 | 7.50 | 7.56 | 7.48 | 7.53 | 7.77 |
| ILMN_1655612 | ARRDC2 | -0.62 | -1.50 | -0.81 | -5.01 | 7.25 | 7.30 | 7.38 | 7.32 | 7.70 |
| ILMN_1745132 | GDF11 | -0.62 | -1.68 | -1.76 | -4.78 | 7.77 | 7.91 | 8.13 | 8.15 | 8.80 |
| ILMN_1655422 | RPL17 | -0.62 | -0.21 | -0.77 | -3.86 | 10.30 | 10.44 | 10.35 | 10.48 | 11.17 |
| ILMN_1724718 | NCK2 | -0.62 | 0.04 | 0.40 | 3.68 | 9.84 | 9.94 | 9.83 | 9.77 | 9.23 |
| ILMN_1681679 | TSPO | -0.62 | -0.66 | -2.57 | -3.31 | 9.02 | 9.16 | 9.17 | 9.59 | 9.75 |
| ILMN_2311826 | USP6NL | -0.61 | -0.08 | -0.37 | 5.16 | 8.52 | 8.57 | 8.53 | 8.55 | 8.10 |
| ILMN_1781672 | GAB1 | -0.61 | 0.20 | -0.92 | 4.12 | 7.88 | 7.98 | 7.85 | 8.02 | 7.26 |
| ILMN_1669394 | EI24 | -0.61 | -1.57 | -1.92 | -4.23 | 9.77 | 9.81 | 9.89 | 9.91 | 10.09 |
| ILMN_2148796 | MND1 | -0.61 | 1.47 | 0.28 | 3.14 | 10.65 | 10.72 | 10.47 | 10.61 | 10.27 |
| ILMN_1697959 | SLC35B4 | -0.61 | -0.91 | -0.98 | -3.01 | 7.57 | 7.63 | 7.66 | 7.67 | 7.87 |
| ILMN_1790819 | LOC728556 | -0.61 | 0.00 | -1.66 | -2.88 | 8.78 | 8.84 | 8.78 | 8.94 | 9.06 |
| ILMN_2212763 | ICAM3 | -0.61 | 0.10 | -0.51 | -4.03 | 9.27 | 9.39 | 9.25 | 9.37 | 10.08 |
| ILMN_2333107 | AES | -0.61 | -2.84 | -2.53 | -2.99 | 8.69 | 8.81 | 9.27 | 9.20 | 9.30 |
| ILMN_1770290 | CNN2 | -0.61 | 0.17 | -0.24 | -3.80 | 8.84 | 8.93 | 8.81 | 8.88 | 9.42 |
| ILMN_1669972 | DKFZp686O24166 | -0.61 | -1.53 | -1.15 | -3.51 | 6.73 | 6.80 | 6.89 | 6.85 | 7.10 |
| ILMN_1765523 | TOLLIP | -0.60 | -1.04 | -0.60 | -3.99 | 7.05 | 7.11 | 7.15 | 7.11 | 7.44 |
| ILMN_2384544 | ADAM15 | -0.60 | 0.03 | -0.80 | -4.52 | 7.42 | 7.48 | 7.41 | 7.50 | 7.87 |
| ILMN_1712389 | CKLF | -0.60 | -0.72 | 0.42 | 4.19 | 10.21 | 10.30 | 10.31 | 10.15 | 9.61 |
| ILMN_1675055 | C1orf166 | -0.60 | 0.36 | 1.48 | -2.77 | 8.00 | 8.05 | 7.97 | 7.88 | 8.23 |
| ILMN_1760778 | ENG | -0.60 | -1.26 | -1.58 | -3.33 | 6.62 | 6.66 | 6.70 | 6.72 | 6.82 |
| ILMN_1811392 | HMHA1 | -0.60 | -2.61 | -1.93 | -2.94 | 8.12 | 8.19 | 8.44 | 8.35 | 8.48 |
| ILMN_1759023 | WFS1 | -0.60 | -1.59 | -2.62 | 6.91 | 8.49 | 8.55 | 8.65 | 8.76 | 7.80 |
| ILMN_1704876 | USP38 | -0.60 | 1.40 | -0.84 | 6.43 | 9.92 | 9.98 | 9.79 | 10.00 | 9.31 |
| ILMN_1719627 | SLC27A3 | -0.60 | -1.36 | -2.02 | -3.15 | 8.01 | 8.08 | 8.17 | 8.25 | 8.38 |
| ILMN_1773059 | GPR124 | -0.59 | -1.10 | -1.78 | -2.73 | 6.97 | 7.04 | 7.09 | 7.17 | 7.28 |
| ILMN_1803624 | TMEM147 | -0.59 | -0.10 | 0.03 | -3.62 | 9.22 | 9.29 | 9.23 | 9.21 | 9.69 |
| ILMN_1651229 | IPO13 | -0.59 | -0.85 | -1.47 | -3.05 | 7.82 | 7.88 | 7.91 | 7.98 | 8.16 |
| ILMN_1697409 | TNFRSF14 | -0.59 | -2.24 | -1.07 | -2.81 | 8.77 | 8.87 | 9.14 | 8.95 | 9.23 |
| ILMN_1749115 | RTN2 | -0.58 | 0.43 | -1.21 | -3.00 | 6.89 | 6.94 | 6.86 | 7.00 | 7.15 |
| ILMN_2336585 | CTAG2 | -0.58 | -0.25 | -1.03 | -2.74 | 10.29 | 10.40 | 10.34 | 10.48 | 10.81 |
| ILMN_2099586 | CCDC28B | -0.58 | -0.38 | -1.06 | -3.11 | 7.66 | 7.74 | 7.71 | 7.80 | 8.07 |
| ILMN_1807925 | GNG2 | -0.58 | 0.65 | -1.77 | 2.93 | 7.41 | 7.49 | 7.32 | 7.65 | 7.00 |
| ILMN_1795026 | FAM189B | -0.58 | -2.32 | -3.72 | -7.34 | 9.60 | 9.64 | 9.76 | 9.85 | 10.10 |
| ILMN_1777745 | FAM133B | -0.58 | -0.73 | 1.26 | -4.01 | 8.64 | 8.71 | 8.73 | 8.48 | 9.15 |
| ILMN_1685441 | ASAP3 | -0.58 | -1.66 | -1.58 | -5.26 | 7.33 | 7.41 | 7.56 | 7.55 | 8.06 |
| ILMN_1695334 | PYGO2 | -0.57 | -2.64 | -2.68 | -5.51 | 7.65 | 7.70 | 7.88 | 7.88 | 8.13 |
| ILMN_1781360 | MPHOSPH6 | -0.57 | -0.03 | 0.47 | 2.86 | 8.52 | 8.60 | 8.53 | 8.46 | 8.16 |
| ILMN_1653856 | STS-1 | -0.57 | -0.97 | -1.63 | -3.41 | 7.32 | 7.41 | 7.47 | 7.57 | 7.84 |
| ILMN_1746706 | LOC653103 | -0.57 | -1.76 | -1.72 | 2.79 | 8.77 | 8.85 | 9.03 | 9.02 | 8.36 |
| ILMN_1699570 | TPD52L2 | -0.57 | -2.19 | -0.77 | -4.54 | 10.41 | 10.47 | 10.63 | 10.49 | 10.86 |
| ILMN_1718988 | DAZAP2 | -0.57 | -1.19 | -0.62 | -3.22 | 11.23 | 11.30 | 11.39 | 11.31 | 11.66 |
| ILMN_3250268 | LOC643911 | -0.57 | 0.59 | 1.17 | 6.17 | 8.34 | 8.46 | 8.23 | 8.11 | 7.12 |
| ILMN_1734353 | GPX4 | -0.57 | 0.82 | 0.13 | -4.79 | 9.85 | 9.92 | 9.74 | 9.83 | 10.47 |
| ILMN_1703955 | FBXO32 | -0.57 | 0.21 | 1.17 | -3.82 | 7.06 | 7.11 | 7.04 | 6.95 | 7.42 |
| ILMN_1887618 |  | -0.57 | -2.20 | -1.96 | -3.72 | 7.55 | 7.64 | 7.89 | 7.85 | 8.12 |
| ILMN_1718770 | FLJ36070 | -0.57 | -1.35 | -0.97 | -3.43 | 6.83 | 6.89 | 6.98 | 6.94 | 7.20 |
| ILMN_1669456 | NME3 | -0.57 | -0.83 | -0.24 | -4.80 | 7.45 | 7.51 | 7.54 | 7.47 | 7.98 |
| ILMN_1815759 | CTDP1 | -0.57 | -0.60 | -0.15 | -3.05 | 7.36 | 7.41 | 7.41 | 7.37 | 7.63 |
| ILMN_2336647 | NNT | -0.56 | 0.63 | -0.47 | 3.61 | 9.28 | 9.34 | 9.21 | 9.33 | 8.89 |
| ILMN_1815682 | C3orf37 | -0.56 | -1.75 | -0.99 | -7.47 | 7.48 | 7.61 | 7.87 | 7.70 | 9.16 |
| ILMN_3279652 | LOC392301 | -0.56 | 1.86 | 0.86 | 3.97 | 7.36 | 7.40 | 7.22 | 7.29 | 7.05 |
| ILMN_1776939 | MS4A1 | -0.56 | 0.78 | 0.50 | -3.02 | 8.56 | 8.70 | 8.38 | 8.44 | 9.29 |
| ILMN_1751395 | KRI1 | -0.56 | -0.19 | 0.33 | -3.30 | 7.32 | 7.36 | 7.33 | 7.30 | 7.56 |
| ILMN_1737689 | LOC649497 | -0.56 | -0.35 | -1.60 | -3.40 | 7.80 | 7.86 | 7.84 | 7.96 | 8.13 |
| ILMN_1662896 | BRWD2 | -0.55 | -3.05 | 0.00 | -3.23 | 7.91 | 7.95 | 8.13 | 7.91 | 8.14 |
| ILMN_3246604 | IPW | -0.55 | -2.16 | 0.22 | -4.71 | 6.80 | 6.84 | 6.93 | 6.79 | 7.09 |
| ILMN_2320888 | CXCR4 | -0.55 | -1.91 | -0.14 | -3.20 | 9.79 | 9.86 | 10.05 | 9.81 | 10.23 |
| ILMN_2407799 | IL24 | -0.55 | -0.70 | -1.30 | -2.95 | 6.79 | 6.83 | 6.84 | 6.88 | 6.99 |
| ILMN_1789839 | GTF3C1 | -0.55 | -1.95 | -0.40 | -3.11 | 7.64 | 7.70 | 7.85 | 7.68 | 7.98 |
| ILMN_2382657 | ARHGAP9 | -0.55 | -1.32 | -2.02 | -4.33 | 8.13 | 8.20 | 8.31 | 8.40 | 8.71 |
| ILMN_1775012 | BBS7 | -0.55 | 1.05 | 0.04 | 4.76 | 7.80 | 7.84 | 7.71 | 7.79 | 7.42 |
| ILMN_3232696 | LOC729816 | -0.55 | 1.24 | 2.64 | 2.79 | 10.53 | 10.59 | 10.39 | 10.24 | 10.22 |
| ILMN_1706342 | ZNF746 | -0.54 | -2.76 | -3.07 | -4.98 | 8.92 | 8.96 | 9.15 | 9.18 | 9.34 |
| ILMN_1741674 | PPP1R9A | -0.54 | -1.47 | -2.72 | -4.08 | 6.77 | 6.84 | 6.95 | 7.11 | 7.27 |
| ILMN_1731107 | CCDC92 | -0.54 | -2.57 | -4.39 | -1.95 | 7.88 | 7.97 | 8.31 | 8.61 | 8.21 |
| ILMN_1767837 | GOLT1B | -0.54 | -0.87 | -0.33 | -2.98 | 8.33 | 8.39 | 8.43 | 8.37 | 8.66 |
| ILMN_3215715 | LOC389386 | -0.54 | 0.36 | -0.90 | 3.43 | 8.24 | 8.30 | 8.20 | 8.34 | 7.86 |
| ILMN_1747227 | ADORA1 | -0.54 | -1.93 | -2.37 | -4.57 | 6.98 | 7.03 | 7.17 | 7.21 | 7.43 |
| ILMN_2408851 | ARHGAP30 | -0.54 | -1.63 | -1.48 | -3.27 | 8.54 | 8.64 | 8.86 | 8.83 | 9.18 |
| ILMN_2389970 | TSPAN32 | -0.54 | -0.58 | -0.70 | -2.94 | 6.74 | 6.78 | 6.78 | 6.79 | 6.97 |
| ILMN_2336595 | ACSS2 | -0.54 | -1.43 | -0.77 | -3.70 | 7.46 | 7.55 | 7.69 | 7.59 | 8.06 |
| ILMN_2227968 | NTHL1 | -0.54 | -1.72 | -0.14 | -3.40 | 8.97 | 9.02 | 9.13 | 8.98 | 9.28 |
| ILMN_1714197 | ACSS2 | -0.53 | -1.93 | -1.01 | -3.95 | 7.60 | 7.69 | 7.93 | 7.77 | 8.27 |
| ILMN_3246962 | CYTSA | -0.53 | -3.42 | -2.51 | -2.94 | 7.25 | 7.29 | 7.49 | 7.43 | 7.46 |
| ILMN_1703683 | COG4 | -0.53 | -0.26 | -1.10 | 4.61 | 8.04 | 8.08 | 8.06 | 8.13 | 7.63 |
| ILMN_1790962 | RINL | -0.53 | 0.77 | -3.09 | -3.53 | 7.82 | 7.86 | 7.76 | 8.07 | 8.11 |
| ILMN_1779353 | PUS7 | -0.53 | -0.71 | 0.00 | -4.40 | 9.93 | 9.98 | 9.99 | 9.93 | 10.32 |
| ILMN_1709091 | OXGR1 | -0.53 | 1.73 | -0.56 | 3.28 | 7.33 | 7.39 | 7.14 | 7.40 | 6.96 |
| ILMN_1789349 | UBQLN4 | -0.53 | -1.58 | -0.66 | -2.82 | 10.17 | 10.23 | 10.33 | 10.24 | 10.46 |
| ILMN_1697363 | C20orf27 | -0.53 | -0.77 | -1.54 | -2.87 | 9.70 | 9.76 | 9.78 | 9.87 | 10.01 |
| ILMN_1657317 | POLR2J | -0.52 | -0.55 | 0.09 | -2.86 | 7.46 | 7.52 | 7.52 | 7.45 | 7.77 |
| ILMN_3244029 | ANKRD52 | -0.52 | -1.83 | -1.98 | -3.13 | 6.70 | 6.73 | 6.82 | 6.83 | 6.90 |
| ILMN_2396639 | PDLIM7 | -0.52 | -0.72 | -1.40 | -3.85 | 6.88 | 6.92 | 6.94 | 7.00 | 7.20 |
| ILMN_1711627 | SIAH1 | -0.52 | 0.30 | 0.54 | 5.27 | 10.63 | 10.71 | 10.59 | 10.55 | 9.82 |
| ILMN_2145423 | DET1 | -0.52 | -1.01 | -1.32 | 2.91 | 7.88 | 7.93 | 7.98 | 8.01 | 7.60 |
| ILMN_3225358 | LOC729495 | -0.52 | -1.78 | -2.99 | -3.81 | 7.10 | 7.14 | 7.23 | 7.33 | 7.39 |
| ILMN_2403730 | ATP6V1H | -0.52 | 1.66 | 2.08 | 2.77 | 8.26 | 8.31 | 8.10 | 8.06 | 7.99 |
| ILMN_1687107 | RFWD3 | -0.52 | -2.21 | -0.19 | 2.94 | 9.46 | 9.54 | 9.80 | 9.49 | 9.01 |
| ILMN_2041577 | GPR172A | -0.52 | 0.60 | 0.64 | -4.34 | 8.21 | 8.26 | 8.16 | 8.16 | 8.58 |
| ILMN_2325837 | CD3D | -0.51 | 0.63 | 1.69 | 5.01 | 7.56 | 7.62 | 7.49 | 7.37 | 7.01 |
| ILMN_1749109 | PSAP | -0.51 | 0.39 | -0.03 | -3.11 | 11.50 | 11.56 | 11.46 | 11.50 | 11.84 |
| ILMN_1804351 | FZD7 | -0.51 | -0.95 | -2.96 | -2.96 | 7.37 | 7.41 | 7.45 | 7.62 | 7.62 |
| ILMN_1772208 | CCDC88B | -0.51 | -0.97 | -1.13 | -3.23 | 6.70 | 6.74 | 6.77 | 6.78 | 6.91 |
| ILMN_1747622 | CD33 | -0.51 | -1.10 | -0.35 | -3.79 | 6.61 | 6.66 | 6.73 | 6.65 | 7.01 |
| ILMN_3261197 | ATP5E | -0.51 | -0.06 | 1.94 | -2.83 | 10.38 | 10.44 | 10.39 | 10.15 | 10.72 |
| ILMN_1789001 | SLC35B2 | -0.50 | -1.54 | -1.65 | -4.32 | 9.66 | 9.72 | 9.84 | 9.85 | 10.17 |
| ILMN_1679405 | DDX56 | -0.50 | -2.05 | -1.04 | -3.09 | 9.81 | 9.87 | 10.03 | 9.92 | 10.14 |
| ILMN_1757408 | ZNF256 | -0.50 | 0.44 | 1.18 | 3.07 | 8.47 | 8.52 | 8.42 | 8.33 | 8.12 |
| ILMN_1723678 | PRPH | -0.50 | 0.09 | -0.68 | -3.50 | 6.59 | 6.62 | 6.58 | 6.64 | 6.84 |
| ILMN_1792710 | DAPK3 | -0.50 | -1.40 | -2.46 | -3.26 | 7.27 | 7.32 | 7.41 | 7.52 | 7.60 |
| ILMN_1776674 | SAC3D1 | -0.50 | -0.55 | -1.91 | -3.68 | 9.98 | 10.04 | 10.05 | 10.21 | 10.42 |
| ILMN_1782954 | HIP2 | -0.50 | 0.21 | -0.74 | 4.51 | 9.08 | 9.12 | 9.06 | 9.14 | 8.72 |
| ILMN_1663667 | FLAD1 | -0.50 | -1.45 | -0.60 | -5.27 | 9.57 | 9.61 | 9.70 | 9.62 | 10.06 |
| ILMN_1803559 | FLJ39632 | -0.49 | -2.57 | -4.08 | -6.62 | 7.65 | 7.69 | 7.86 | 7.98 | 8.19 |
| ILMN_1700541 | FBLN1 | -0.49 | 1.04 | 1.07 | 2.90 | 7.35 | 7.42 | 7.18 | 7.18 | 6.89 |
| ILMN_1722648 | SF3B4 | -0.49 | -2.08 | -0.73 | -3.18 | 10.83 | 10.87 | 11.00 | 10.89 | 11.09 |
| ILMN_3238633 | SDHAF2 | -0.49 | 0.54 | 0.49 | -3.28 | 9.84 | 9.91 | 9.76 | 9.77 | 10.31 |
| ILMN_1732985 | PHF20L1 | -0.49 | 2.67 | 0.81 | 3.22 | 8.68 | 8.73 | 8.41 | 8.60 | 8.35 |
| ILMN_1663132 | ADCK2 | -0.49 | -1.42 | -1.91 | -3.09 | 9.74 | 9.82 | 9.97 | 10.05 | 10.24 |
| ILMN_2303669 | SLC4A8 | -0.49 | -1.12 | -1.12 | -2.73 | 6.57 | 6.61 | 6.67 | 6.67 | 6.81 |
| ILMN_1725130 | FAM50A | -0.48 | -2.01 | -2.88 | -3.23 | 9.75 | 9.82 | 10.02 | 10.13 | 10.18 |
| ILMN_1744897 | KCNN3 | -0.48 | 0.31 | 0.39 | -6.37 | 6.81 | 6.87 | 6.78 | 6.77 | 7.57 |
| ILMN_1811592 | ARHGAP21 | -0.48 | -1.51 | -1.34 | 4.41 | 8.33 | 8.42 | 8.60 | 8.57 | 7.53 |
| ILMN_1657847 | SUNC1 | -0.47 | 0.24 | 0.39 | -2.99 | 6.93 | 6.97 | 6.91 | 6.90 | 7.18 |
| ILMN_1676280 | NSUN3 | -0.47 | 2.08 | 1.94 | 4.30 | 7.29 | 7.32 | 7.14 | 7.15 | 6.99 |
| ILMN_1785768 | PDE4A | -0.47 | -2.12 | -1.65 | -2.92 | 7.25 | 7.28 | 7.40 | 7.36 | 7.45 |
| ILMN_1738549 | VPREB1 | -0.47 | -1.60 | -2.24 | -5.07 | 7.90 | 8.10 | 8.59 | 8.87 | 10.09 |
| ILMN_1671800 | LOC643911 | -0.47 | 1.75 | 2.10 | 9.64 | 8.81 | 8.88 | 8.58 | 8.53 | 7.51 |
| ILMN_1777261 | FAM3C | -0.47 | -1.18 | -0.86 | -3.75 | 7.06 | 7.12 | 7.22 | 7.17 | 7.56 |
| ILMN_2213199 | KIAA1712 | -0.47 | 0.18 | -0.25 | 6.08 | 9.85 | 9.89 | 9.83 | 9.87 | 9.28 |
| ILMN_1695991 | COLQ | -0.46 | -1.31 | -2.02 | -2.91 | 7.03 | 7.07 | 7.14 | 7.19 | 7.26 |
| ILMN_2367063 | TPK1 | -0.46 | 0.17 | 0.84 | 2.74 | 6.77 | 6.81 | 6.76 | 6.70 | 6.55 |
| ILMN_1810000 | RDH16 | -0.46 | -1.04 | -0.19 | -2.74 | 6.66 | 6.70 | 6.75 | 6.68 | 6.90 |
| ILMN_1714158 | PON2 | -0.46 | -0.62 | -1.55 | -3.30 | 7.93 | 8.00 | 8.02 | 8.15 | 8.41 |
| ILMN_1775677 | TYSND1 | -0.46 | -1.41 | -1.19 | -3.92 | 10.00 | 10.06 | 10.17 | 10.15 | 10.48 |
| ILMN_2363065 | RTN3 | -0.46 | -0.70 | -1.13 | -2.74 | 9.15 | 9.22 | 9.26 | 9.32 | 9.57 |
| ILMN_1659766 | BAG3 | -0.46 | 2.20 | 1.70 | 3.47 | 8.29 | 8.35 | 7.98 | 8.05 | 7.81 |
| ILMN_1667857 | C12orf52 | -0.45 | -1.54 | -1.84 | -4.16 | 9.39 | 9.45 | 9.59 | 9.63 | 9.94 |
| ILMN_3248057 | LOC100132439 | -0.45 | -1.66 | -1.92 | -3.46 | 7.95 | 7.99 | 8.09 | 8.12 | 8.25 |
| ILMN_2309180 | SMARCD3 | -0.45 | -1.80 | -1.89 | -5.39 | 6.93 | 7.00 | 7.20 | 7.21 | 7.73 |
| ILMN_1711227 | GMDS | -0.45 | -0.81 | -0.77 | -3.33 | 9.09 | 9.16 | 9.21 | 9.20 | 9.58 |
| ILMN_1703891 | TBC1D9 | -0.45 | 0.37 | -0.11 | 3.45 | 9.32 | 9.41 | 9.24 | 9.34 | 8.60 |
| ILMN_1709396 | PDCD10 | -0.45 | 1.42 | 1.32 | 2.79 | 7.78 | 7.84 | 7.60 | 7.62 | 7.43 |
| ILMN_1671486 | HOMER2 | -0.45 | 0.70 | 1.93 | 4.53 | 8.38 | 8.48 | 8.24 | 7.98 | 7.44 |
| ILMN_3292551 | LOC286157 | -0.45 | -1.80 | -1.70 | -2.86 | 9.70 | 9.79 | 10.06 | 10.04 | 10.28 |
| ILMN_1682736 | LOC643452 | -0.44 | -0.49 | 1.59 | 8.90 | 8.50 | 8.54 | 8.54 | 8.38 | 7.83 |
| ILMN_1770127 | DNAJA2 | -0.44 | 0.97 | 1.57 | 3.95 | 11.06 | 11.10 | 10.98 | 10.93 | 10.74 |
| ILMN_1687626 | ZDHHC24 | -0.44 | 0.06 | 0.25 | -3.04 | 7.47 | 7.52 | 7.46 | 7.44 | 7.83 |
| ILMN_2292387 | TANK | -0.44 | 2.38 | 3.03 | 3.88 | 7.69 | 7.73 | 7.46 | 7.39 | 7.31 |
| ILMN_1881960 |  | -0.44 | -0.09 | -1.76 | 3.61 | 8.06 | 8.11 | 8.07 | 8.25 | 7.68 |
| ILMN_1764945 | AP3D1 | -0.44 | -2.42 | -2.90 | -3.42 | 8.18 | 8.22 | 8.39 | 8.43 | 8.47 |
| ILMN_2160209 | TACSTD1 | -0.44 | -0.75 | -1.56 | -2.84 | 6.81 | 6.85 | 6.89 | 6.97 | 7.11 |
| ILMN_2159044 | PDF | -0.44 | 0.44 | 0.08 | 3.92 | 7.10 | 7.14 | 7.07 | 7.10 | 6.77 |
| ILMN_3248928 | UBN2 | -0.43 | -0.57 | -0.07 | -2.77 | 7.01 | 7.05 | 7.07 | 7.02 | 7.29 |
| ILMN_3284447 | LOC647150 | -0.43 | 0.81 | 0.58 | 2.96 | 8.56 | 8.64 | 8.40 | 8.44 | 7.98 |
| ILMN_3240698 | LOC388279 | -0.43 | 0.21 | -0.34 | 2.88 | 6.87 | 6.91 | 6.86 | 6.90 | 6.65 |
| ILMN_1750805 | ARHGAP30 | -0.43 | -2.67 | -2.24 | -6.96 | 9.05 | 9.09 | 9.32 | 9.27 | 9.75 |
| ILMN_2089902 | NUS1 | -0.43 | -1.71 | -1.12 | -3.42 | 7.20 | 7.23 | 7.31 | 7.27 | 7.42 |
| ILMN_1802646 | EPHB6 | -0.43 | -0.68 | -1.15 | -4.06 | 6.84 | 6.87 | 6.89 | 6.93 | 7.15 |
| ILMN_1701998 | AFAP1 | -0.43 | -2.07 | -1.86 | 3.21 | 8.32 | 8.38 | 8.64 | 8.61 | 7.82 |
| ILMN_1677827 | TLR7 | -0.43 | 2.64 | 1.13 | 3.53 | 9.49 | 9.56 | 9.08 | 9.31 | 8.94 |
| ILMN_1757387 | UCHL1 | -0.42 | 0.38 | -0.56 | 2.83 | 7.90 | 7.97 | 7.85 | 7.99 | 7.48 |
| ILMN_2184708 | LIN7C | -0.42 | 0.11 | 0.00 | 2.87 | 7.59 | 7.63 | 7.58 | 7.59 | 7.34 |
| ILMN_1777061 | ZSWIM6 | -0.42 | 1.28 | 1.16 | 6.02 | 8.33 | 8.38 | 8.19 | 8.20 | 7.66 |
| ILMN_1737146 | TRAM1 | -0.42 | -0.96 | -1.33 | -3.62 | 11.18 | 11.24 | 11.31 | 11.35 | 11.65 |
| ILMN_1770378 | ANAPC10 | -0.42 | 2.59 | 0.96 | 6.68 | 9.49 | 9.53 | 9.22 | 9.39 | 8.79 |
| ILMN_2134555 | KCTD3 | -0.41 | -2.39 | -2.11 | -2.99 | 7.63 | 7.69 | 7.97 | 7.93 | 8.06 |
| ILMN_3251436 | DENND4C | -0.41 | -0.98 | -0.51 | 3.45 | 6.92 | 6.94 | 6.98 | 6.95 | 6.69 |
| ILMN_2209027 | RPS26 | -0.41 | -0.07 | -1.76 | -4.76 | 10.24 | 10.30 | 10.25 | 10.49 | 10.90 |
| ILMN_1692844 | TBC1D19 | -0.41 | 0.33 | 1.35 | 4.59 | 7.65 | 7.68 | 7.62 | 7.54 | 7.28 |
| ILMN_1785356 | DENND5A | -0.41 | 0.32 | -0.82 | 3.56 | 9.44 | 9.49 | 9.40 | 9.53 | 9.03 |
| ILMN_2288232 | PHKB | -0.41 | -0.17 | -0.07 | 2.75 | 7.90 | 7.97 | 7.93 | 7.91 | 7.40 |
| ILMN_1749629 | CUL1 | -0.41 | -1.00 | 0.16 | -3.50 | 9.66 | 9.71 | 9.78 | 9.64 | 10.09 |
| ILMN_2160210 | TACSTD1 | -0.40 | -0.76 | -2.14 | -4.98 | 7.38 | 7.43 | 7.48 | 7.68 | 8.08 |
| ILMN_1714159 | LUZP1 | -0.40 | 1.36 | 3.24 | 2.96 | 8.42 | 8.46 | 8.31 | 8.15 | 8.18 |
| ILMN_1738684 | NRXN2 | -0.40 | -1.46 | -0.18 | -2.80 | 6.81 | 6.85 | 6.95 | 6.83 | 7.07 |
| ILMN_1859524 |  | -0.40 | -2.45 | -2.27 | -5.22 | 7.02 | 7.06 | 7.25 | 7.23 | 7.50 |
| ILMN_1795856 | LOC644935 | -0.39 | -0.25 | 0.82 | 5.83 | 9.01 | 9.06 | 9.04 | 8.91 | 8.32 |
| ILMN_1733931 | PDCD6 | -0.39 | -2.17 | -0.99 | -2.91 | 10.78 | 10.81 | 10.93 | 10.85 | 10.98 |
| ILMN_1658499 | SYT13 | -0.39 | -0.35 | -0.35 | -5.49 | 6.58 | 6.61 | 6.61 | 6.61 | 7.05 |
| ILMN_1740169 | TYRO3 | -0.39 | -0.78 | -0.66 | -2.92 | 6.67 | 6.70 | 6.73 | 6.72 | 6.92 |
| ILMN_2098947 | LOC338799 | -0.39 | -1.87 | -0.89 | -4.27 | 6.95 | 6.99 | 7.16 | 7.05 | 7.43 |
| ILMN_1655755 | LOC400879 | -0.38 | -0.82 | -0.27 | -2.85 | 6.78 | 6.80 | 6.83 | 6.79 | 6.95 |
| ILMN_1764383 | MCOLN1 | -0.38 | -1.10 | -0.46 | -3.28 | 7.86 | 7.91 | 8.01 | 7.92 | 8.32 |
| ILMN_1670809 | NRM | -0.38 | -2.61 | -2.54 | -3.17 | 7.30 | 7.34 | 7.55 | 7.54 | 7.60 |
| ILMN_1671116 | C3orf21 | -0.38 | -0.52 | -0.49 | 3.12 | 9.47 | 9.51 | 9.52 | 9.52 | 9.17 |
| ILMN_1738491 | SNX30 | -0.38 | -1.42 | -0.88 | -5.46 | 7.57 | 7.61 | 7.72 | 7.66 | 8.15 |
| ILMN_1876924 | WNK1 | -0.38 | -2.45 | -2.83 | -3.43 | 9.07 | 9.12 | 9.39 | 9.44 | 9.52 |
| ILMN_2330552 | CDC2L2 | -0.38 | -1.19 | -1.79 | -3.14 | 6.99 | 7.03 | 7.12 | 7.18 | 7.32 |
| ILMN_1763264 | MRPL2 | -0.38 | -0.55 | -0.60 | -2.91 | 9.99 | 10.04 | 10.06 | 10.07 | 10.37 |
| ILMN_1711514 | COCH | -0.38 | -1.67 | -2.66 | -4.60 | 8.00 | 8.07 | 8.33 | 8.52 | 8.90 |
| ILMN_1680624 | CREG1 | -0.37 | 1.61 | 1.74 | -3.63 | 8.96 | 9.01 | 8.71 | 8.69 | 9.51 |
| ILMN_1666512 | SKIV2L | -0.37 | -2.20 | -1.25 | -4.20 | 8.79 | 8.82 | 8.97 | 8.89 | 9.13 |
| ILMN_2213558 | TMED10P | -0.37 | -0.37 | 0.48 | -2.75 | 10.57 | 10.61 | 10.61 | 10.53 | 10.84 |
| ILMN_1665696 | EFNA4 | -0.37 | -0.05 | -1.23 | -4.11 | 7.51 | 7.53 | 7.51 | 7.58 | 7.76 |
| ILMN_1653220 | PITPNM1 | -0.37 | -0.94 | -2.06 | -2.85 | 8.77 | 8.82 | 8.90 | 9.05 | 9.15 |
| ILMN_1659845 | KIAA0355 | -0.37 | -1.75 | -0.24 | 3.96 | 7.64 | 7.68 | 7.82 | 7.67 | 7.25 |
| ILMN_1775692 | EIF4G3 | -0.37 | -0.90 | -1.07 | -3.58 | 8.73 | 8.77 | 8.82 | 8.84 | 9.09 |
| ILMN_1690822 | VAPA | -0.37 | -0.41 | 0.29 | -3.52 | 7.07 | 7.10 | 7.11 | 7.05 | 7.36 |
| ILMN_2105573 | CCL3L3 | -0.37 | -1.61 | -1.51 | -5.63 | 6.89 | 6.95 | 7.15 | 7.13 | 7.81 |
| ILMN_2296369 | MATR3 | -0.37 | -0.14 | 1.16 | -2.85 | 7.46 | 7.51 | 7.48 | 7.33 | 7.80 |
| ILMN_2142935 | BLNK | -0.36 | 1.89 | 0.10 | 3.36 | 7.92 | 7.97 | 7.68 | 7.91 | 7.49 |
| ILMN_2362902 | RASSF5 | -0.36 | -1.25 | -0.95 | -3.70 | 9.74 | 9.78 | 9.89 | 9.85 | 10.18 |
| ILMN_2051381 | ALDH16A1 | -0.36 | -1.56 | -2.64 | -3.36 | 7.50 | 7.53 | 7.63 | 7.72 | 7.78 |
| ILMN_1742332 | KCTD12 | -0.36 | -2.78 | -5.33 | -5.41 | 7.18 | 7.21 | 7.41 | 7.62 | 7.63 |
| ILMN_1809352 | PRL | -0.36 | -2.02 | -1.51 | -4.89 | 6.97 | 7.05 | 7.46 | 7.33 | 8.15 |
| ILMN_1812031 | PALM | -0.36 | -0.95 | -0.24 | -2.85 | 6.64 | 6.67 | 6.72 | 6.66 | 6.88 |
| ILMN_1715214 | PTPN7 | -0.35 | -1.90 | -2.64 | -3.86 | 7.88 | 7.92 | 8.08 | 8.15 | 8.28 |
| ILMN_2038775 | TUBB2A | -0.35 | -0.66 | -1.66 | 3.68 | 8.01 | 8.06 | 8.10 | 8.25 | 7.49 |
| ILMN_3266471 | LOC100129566 | -0.35 | -0.60 | -0.46 | -3.39 | 8.39 | 8.44 | 8.47 | 8.45 | 8.87 |
| ILMN_1754068 | LOC643233 | -0.35 | -0.60 | -1.42 | -2.80 | 6.76 | 6.78 | 6.80 | 6.87 | 6.97 |
| ILMN_1801105 | PRKCD | -0.34 | -0.45 | -0.52 | -2.76 | 8.48 | 8.52 | 8.53 | 8.53 | 8.75 |
| ILMN_2376194 | CAMK2B | -0.34 | -3.02 | -3.70 | -5.40 | 7.49 | 7.54 | 7.96 | 8.07 | 8.33 |
| ILMN_1745737 | DIS3L2 | -0.34 | -1.55 | -0.44 | -3.19 | 7.25 | 7.27 | 7.35 | 7.28 | 7.47 |
| ILMN_1713561 | C20orf103 | -0.34 | 2.31 | 1.12 | -4.40 | 10.39 | 10.45 | 9.97 | 10.19 | 11.17 |
| ILMN_1807596 | UBAP1 | -0.34 | 2.25 | 1.28 | 3.02 | 8.35 | 8.38 | 8.12 | 8.22 | 8.05 |
| ILMN_1775111 | SND1 | -0.34 | -2.60 | -0.64 | -5.50 | 9.29 | 9.33 | 9.60 | 9.36 | 9.94 |
| ILMN_2367113 | CASP6 | -0.33 | 2.13 | -0.67 | 6.80 | 7.39 | 7.42 | 7.22 | 7.44 | 6.85 |
| ILMN_2375651 | SCNM1 | -0.33 | 0.24 | -0.14 | -3.36 | 8.50 | 8.56 | 8.46 | 8.53 | 9.07 |
| ILMN_1657680 | CCDC69 | -0.33 | -0.29 | -1.98 | -2.73 | 7.98 | 8.03 | 8.02 | 8.29 | 8.41 |
| ILMN_1729167 | EFTUD1 | -0.33 | 0.71 | 1.95 | 3.05 | 8.00 | 8.03 | 7.95 | 7.87 | 7.79 |
| ILMN_1769282 | FRMD6 | -0.33 | -1.24 | -2.19 | -3.42 | 6.70 | 6.72 | 6.78 | 6.85 | 6.94 |
| ILMN_1687958 | SLC25A22 | -0.33 | -2.31 | -0.78 | -4.28 | 7.66 | 7.69 | 7.85 | 7.72 | 8.01 |
| ILMN_1689655 | HLA-DRA | -0.33 | 0.37 | -0.12 | 2.84 | 13.22 | 13.24 | 13.19 | 13.23 | 12.99 |
| ILMN_1811171 | GPR132 | -0.33 | 0.46 | -0.87 | -4.06 | 7.09 | 7.14 | 7.02 | 7.22 | 7.71 |
| ILMN_1773901 | STX12 | -0.33 | 0.73 | 1.51 | 2.90 | 8.05 | 8.07 | 7.99 | 7.92 | 7.81 |
| ILMN_1724825 | PCBP2 | -0.32 | -3.13 | -1.06 | -5.79 | 11.06 | 11.09 | 11.41 | 11.18 | 11.71 |
| ILMN_1827211 |  | -0.32 | 0.36 | -2.38 | -3.17 | 7.38 | 7.41 | 7.35 | 7.60 | 7.68 |
| ILMN_1801710 | APBB1IP | -0.32 | 0.16 | -1.75 | 3.89 | 11.21 | 11.25 | 11.19 | 11.42 | 10.73 |
| ILMN_3307648 | CS | -0.32 | 0.23 | -0.26 | -3.02 | 11.29 | 11.32 | 11.26 | 11.32 | 11.63 |
| ILMN_3251477 | RG9MTD3 | -0.32 | 1.36 | 1.69 | 2.95 | 7.67 | 7.72 | 7.49 | 7.45 | 7.28 |
| ILMN_1707199 | WDR20 | -0.32 | 1.47 | 1.54 | 3.11 | 8.94 | 8.97 | 8.80 | 8.80 | 8.65 |
| ILMN_1688753 | PTDSS1 | -0.32 | -0.58 | -1.45 | -3.47 | 9.64 | 9.67 | 9.70 | 9.79 | 10.00 |
| ILMN_1689725 | RPLP1 | -0.32 | 0.27 | 0.43 | 3.44 | 12.66 | 12.70 | 12.62 | 12.59 | 12.15 |
| ILMN_1681304 | PAN3 | -0.32 | -0.90 | -1.71 | -2.75 | 9.69 | 9.73 | 9.80 | 9.89 | 10.01 |
| ILMN_1814526 | ADD3 | -0.32 | 1.10 | 3.47 | 5.27 | 9.34 | 9.38 | 9.23 | 8.98 | 8.79 |
| ILMN_1707123 | DDX19B | -0.31 | 1.32 | 0.22 | 4.09 | 6.94 | 6.96 | 6.84 | 6.92 | 6.63 |
| ILMN_1793732 | FARS2 | -0.31 | -0.15 | 0.27 | 4.56 | 8.73 | 8.75 | 8.74 | 8.70 | 8.33 |
| ILMN_2268156 | LFNG | -0.31 | -1.58 | -0.66 | -3.66 | 6.67 | 6.69 | 6.77 | 6.71 | 6.91 |
| ILMN_1775522 | MAGED1 | -0.30 | 0.27 | 0.91 | 2.81 | 11.43 | 11.46 | 11.40 | 11.33 | 11.12 |
| ILMN_2067980 | NKAP | -0.30 | 0.84 | 0.27 | 4.20 | 7.40 | 7.43 | 7.30 | 7.37 | 6.93 |
| ILMN_1789627 | 41522 | -0.30 | 0.18 | 0.04 | -3.39 | 6.71 | 6.74 | 6.69 | 6.71 | 7.02 |
| ILMN_2116299 | GRRP1 | -0.30 | -0.54 | -0.59 | -3.25 | 6.71 | 6.73 | 6.75 | 6.75 | 6.93 |
| ILMN_1657495 | MLEC | -0.30 | -1.98 | -0.80 | -3.62 | 9.58 | 9.61 | 9.82 | 9.68 | 10.03 |
| ILMN_1786379 | SYNGR3 | -0.29 | -0.79 | -0.05 | -5.94 | 6.61 | 6.63 | 6.66 | 6.61 | 7.01 |
| ILMN_1669433 | KIAA0913 | -0.29 | -2.75 | -2.29 | -3.22 | 8.27 | 8.30 | 8.58 | 8.53 | 8.63 |
| ILMN_1675677 | TMPRSS3 | -0.29 | -1.27 | -1.86 | -4.55 | 7.89 | 7.98 | 8.28 | 8.46 | 9.29 |
| ILMN_2375319 | RASGRP2 | -0.29 | -1.91 | -0.84 | -6.06 | 7.95 | 7.98 | 8.15 | 8.04 | 8.57 |
| ILMN_2366334 | FERMT3 | -0.29 | -0.48 | -0.90 | -2.77 | 7.66 | 7.71 | 7.74 | 7.80 | 8.10 |
| ILMN_1787324 | C16orf48 | -0.29 | -2.27 | -1.55 | 8.61 | 9.74 | 9.77 | 9.95 | 9.88 | 8.94 |
| ILMN_1801348 | GOT2 | -0.29 | -0.93 | 0.99 | 6.62 | 11.28 | 11.31 | 11.37 | 11.17 | 10.59 |
| ILMN_1722532 | JMJD1A | -0.29 | 1.62 | 0.78 | 2.98 | 8.97 | 9.00 | 8.76 | 8.87 | 8.59 |
| ILMN_1796235 | CIRH1A | -0.28 | 0.19 | 1.04 | 4.02 | 10.94 | 10.97 | 10.92 | 10.83 | 10.52 |
| ILMN_1761068 | MGC52000 | -0.28 | -2.77 | -1.67 | -2.77 | 7.85 | 7.88 | 8.15 | 8.03 | 8.15 |
| ILMN_2289844 | SERHL | -0.28 | 1.81 | 1.01 | 3.49 | 7.74 | 7.78 | 7.46 | 7.58 | 7.20 |
| ILMN_1683462 | GSS | -0.28 | -0.28 | -0.22 | -3.63 | 7.97 | 8.00 | 8.00 | 7.99 | 8.36 |
| ILMN_1703692 | LOC647000 | -0.28 | -1.16 | -1.05 | 3.06 | 10.76 | 10.79 | 10.90 | 10.88 | 10.39 |
| ILMN_3300198 | LOC729580 | -0.27 | -1.05 | 0.36 | -2.78 | 7.11 | 7.13 | 7.19 | 7.09 | 7.32 |
| ILMN_3251643 | C1orf194 | -0.27 | -1.78 | -2.32 | -3.22 | 7.04 | 7.08 | 7.26 | 7.33 | 7.44 |
| ILMN_3207933 | LOC647150 | -0.27 | 0.87 | 0.05 | 4.89 | 10.40 | 10.44 | 10.30 | 10.40 | 9.81 |
| ILMN_1793517 | RASAL1 | -0.27 | -0.50 | 0.37 | -2.78 | 8.54 | 8.58 | 8.61 | 8.49 | 8.92 |
| ILMN_1787718 | SLC27A1 | -0.27 | -0.75 | -0.05 | -3.13 | 6.74 | 6.76 | 6.79 | 6.74 | 6.93 |
| ILMN_2302118 | CCDC50 | -0.27 | 2.16 | 0.64 | 5.34 | 10.96 | 10.99 | 10.69 | 10.88 | 10.29 |
| ILMN_3228585 | LOC728661 | -0.26 | -1.86 | -1.21 | -2.78 | 9.69 | 9.73 | 9.95 | 9.86 | 10.08 |
| ILMN_2205963 | C10orf54 | -0.26 | 0.04 | -0.35 | -3.22 | 6.73 | 6.75 | 6.73 | 6.76 | 6.98 |
| ILMN_1668039 | GYPC | -0.26 | -1.74 | -0.30 | -4.84 | 8.73 | 8.78 | 9.02 | 8.78 | 9.54 |
| ILMN_1775542 | FAIM3 | -0.26 | -0.33 | -1.00 | -3.50 | 8.68 | 8.74 | 8.76 | 8.90 | 9.45 |
| ILMN_1800396 | KCNH4 | -0.26 | -0.98 | -0.98 | -2.83 | 6.62 | 6.64 | 6.70 | 6.70 | 6.84 |
| ILMN_2163975 | OXGR1 | -0.26 | 1.34 | -0.23 | 3.85 | 7.16 | 7.19 | 7.01 | 7.19 | 6.71 |
| ILMN_3235188 | LOC100131187 | -0.25 | -0.13 | -0.17 | -4.12 | 7.40 | 7.42 | 7.41 | 7.41 | 7.72 |
| ILMN_1720482 | CEND1 | -0.25 | -1.76 | -0.96 | -4.77 | 6.87 | 6.89 | 7.01 | 6.95 | 7.25 |
| ILMN_3239871 | LOC643896 | -0.25 | 1.70 | 1.00 | 4.01 | 7.01 | 7.03 | 6.90 | 6.94 | 6.74 |
| ILMN_1746716 | LOC643035 | -0.25 | -2.39 | -2.46 | -3.07 | 7.40 | 7.42 | 7.62 | 7.63 | 7.68 |
| ILMN_1665510 | ERRFI1 | -0.25 | -1.31 | -0.31 | -2.93 | 6.94 | 6.97 | 7.08 | 6.98 | 7.26 |
| ILMN_1676515 | IMPDH1 | -0.25 | -0.88 | -0.61 | -2.73 | 8.06 | 8.09 | 8.19 | 8.15 | 8.46 |
| ILMN_2311989 | CUTA | -0.25 | -1.45 | -1.84 | -2.89 | 10.25 | 10.28 | 10.41 | 10.45 | 10.56 |
| ILMN_2123402 | TMEM4 | -0.24 | 0.47 | 0.00 | -3.43 | 9.48 | 9.51 | 9.41 | 9.48 | 9.94 |
| ILMN_1730568 | ZDHHC7 | -0.24 | 0.11 | -0.90 | 2.96 | 9.43 | 9.46 | 9.41 | 9.54 | 9.06 |
| ILMN_1795359 | SPRR2A | -0.24 | 0.00 | -0.12 | -3.75 | 6.64 | 6.66 | 6.64 | 6.65 | 6.95 |
| ILMN_1746864 | PSCDBP | -0.24 | 2.35 | 1.79 | 3.55 | 10.15 | 10.18 | 9.86 | 9.93 | 9.71 |
| ILMN_1707077 | SORT1 | -0.24 | 0.14 | 1.34 | -4.27 | 6.87 | 6.90 | 6.85 | 6.68 | 7.46 |
| ILMN_3237319 | LOC678655 | -0.24 | -0.43 | -0.33 | -3.86 | 7.61 | 7.63 | 7.64 | 7.63 | 7.88 |
| ILMN_2151441 | FAM103A1 | -0.24 | 0.40 | -0.12 | 3.92 | 9.78 | 9.80 | 9.75 | 9.79 | 9.45 |
| ILMN_1741768 | TMPRSS3 | -0.23 | -1.34 | -1.77 | -4.26 | 9.18 | 9.26 | 9.64 | 9.78 | 10.63 |
| ILMN_3215138 | LOC285804 | -0.23 | 1.30 | 1.54 | 4.00 | 6.86 | 6.88 | 6.77 | 6.75 | 6.58 |
| ILMN_2415572 | GALC | -0.23 | -0.77 | -1.01 | -2.94 | 6.71 | 6.73 | 6.78 | 6.80 | 6.96 |
| ILMN_1733932 | SNUPN | -0.23 | 2.19 | 1.32 | 3.41 | 8.95 | 8.97 | 8.73 | 8.82 | 8.61 |
| ILMN_1662174 | ORMDL3 | -0.23 | 0.19 | -0.12 | -3.16 | 6.87 | 6.89 | 6.86 | 6.88 | 7.15 |
| ILMN_2052790 | NONO | -0.23 | -0.30 | 0.34 | 5.97 | 12.60 | 12.62 | 12.63 | 12.57 | 12.08 |
| ILMN_1690464 | TMEM20 | -0.23 | -0.59 | 0.46 | -3.14 | 6.87 | 6.88 | 6.91 | 6.83 | 7.10 |
| ILMN_1721977 | ARD1A | -0.23 | -1.03 | -1.30 | -5.28 | 9.86 | 9.90 | 10.03 | 10.07 | 10.72 |
| ILMN_1695058 | SLC38A5 | -0.22 | -1.04 | -1.77 | -4.51 | 8.24 | 8.27 | 8.36 | 8.45 | 8.78 |
| ILMN_2411559 | PUS1 | -0.22 | -3.04 | -2.45 | -5.01 | 7.52 | 7.54 | 7.80 | 7.74 | 7.97 |
| ILMN_1664802 | WSB1 | -0.22 | 1.33 | 0.74 | 3.17 | 9.39 | 9.42 | 9.21 | 9.29 | 8.96 |
| ILMN_2380566 | SIAH1 | -0.22 | 0.88 | -0.31 | 9.55 | 9.29 | 9.31 | 9.22 | 9.31 | 8.57 |
| ILMN_3273990 | LOC100128374 | -0.22 | -0.66 | 0.28 | -3.14 | 6.79 | 6.80 | 6.83 | 6.77 | 6.98 |
| ILMN_1658989 | MEX3B | -0.22 | -0.87 | -4.10 | 3.05 | 7.30 | 7.31 | 7.36 | 7.61 | 7.06 |
| ILMN_2323633 | TPD52L2 | -0.22 | -0.86 | -0.47 | -4.23 | 8.14 | 8.16 | 8.21 | 8.18 | 8.47 |
| ILMN_2097858 | KIAA1737 | -0.21 | -1.31 | 0.36 | -3.05 | 9.94 | 9.96 | 10.06 | 9.90 | 10.22 |
| ILMN_2403906 | ARFIP1 | -0.21 | 2.52 | 0.55 | 7.15 | 9.17 | 9.18 | 8.97 | 9.12 | 8.60 |
| ILMN_2176931 | PELI3 | -0.21 | -2.13 | -0.52 | -4.82 | 6.81 | 6.83 | 7.01 | 6.86 | 7.27 |
| ILMN_1770505 | BIK | -0.21 | -1.67 | 0.34 | -5.61 | 6.62 | 6.64 | 6.83 | 6.57 | 7.33 |
| ILMN_1713966 | MRPL52 | -0.21 | -0.15 | -0.36 | 4.23 | 8.85 | 8.88 | 8.87 | 8.89 | 8.38 |
| ILMN_2412549 | GAR1 | -0.21 | 0.36 | 0.21 | 4.58 | 11.50 | 11.52 | 11.45 | 11.47 | 10.91 |
| ILMN_1759595 | C22orf29 | -0.21 | -1.78 | -1.84 | -2.96 | 7.61 | 7.63 | 7.81 | 7.82 | 7.94 |
| ILMN_1663080 | LFNG | -0.20 | -1.95 | -3.44 | -3.03 | 9.89 | 9.91 | 10.11 | 10.28 | 10.24 |
| ILMN_2158548 | NACA2 | -0.20 | 1.58 | 0.40 | -2.77 | 8.21 | 8.23 | 8.02 | 8.16 | 8.54 |
| ILMN_1708709 | KIAA1211 | -0.20 | -0.11 | -0.99 | 2.88 | 7.16 | 7.18 | 7.17 | 7.27 | 6.82 |
| ILMN_1751072 | SRPRB | -0.20 | -0.69 | 1.18 | -2.75 | 8.90 | 8.92 | 8.97 | 8.78 | 9.18 |
| ILMN_2413141 | C6orf162 | -0.20 | -0.35 | 0.16 | -2.94 | 6.96 | 6.98 | 6.99 | 6.95 | 7.21 |
| ILMN_1680874 | TUBB2B | -0.20 | 0.68 | 1.56 | 5.18 | 9.17 | 9.22 | 9.00 | 8.77 | 7.85 |
| ILMN_1772312 | BARX1 | -0.19 | 0.82 | -0.86 | -3.98 | 6.67 | 6.68 | 6.61 | 6.73 | 6.94 |
| ILMN_2133936 | ZFP1 | -0.19 | -0.56 | -1.02 | 6.55 | 8.54 | 8.56 | 8.59 | 8.63 | 7.96 |
| ILMN_2147993 | ZNF23 | -0.19 | 0.73 | 0.50 | 3.67 | 8.20 | 8.23 | 8.08 | 8.12 | 7.61 |
| ILMN_1772131 | IL1R2 | -0.18 | -0.11 | -1.05 | -6.05 | 6.97 | 6.99 | 6.98 | 7.07 | 7.53 |
| ILMN_2222074 | PTPN12 | -0.18 | 1.52 | 2.64 | 8.95 | 8.16 | 8.18 | 8.02 | 7.92 | 7.33 |
| ILMN_1697200 | MON2 | -0.18 | -1.29 | -0.86 | -4.36 | 8.36 | 8.37 | 8.48 | 8.44 | 8.76 |
| ILMN_1726434 | UNC45A | -0.18 | -1.07 | -1.46 | -3.34 | 8.33 | 8.35 | 8.45 | 8.49 | 8.70 |
| ILMN_1739840 | LRRC8A | -0.18 | 0.97 | 0.04 | 2.87 | 7.55 | 7.57 | 7.48 | 7.55 | 7.34 |
| ILMN_1730347 | CCDC115 | -0.17 | 0.37 | -0.63 | -3.60 | 8.48 | 8.51 | 8.41 | 8.60 | 9.16 |
| ILMN_1735004 | C4orf43 | -0.17 | 2.30 | 0.78 | 8.19 | 8.01 | 8.02 | 7.83 | 7.95 | 7.38 |
| ILMN_1670218 | EXOSC6 | -0.17 | -0.56 | 0.85 | 4.59 | 10.20 | 10.23 | 10.28 | 10.09 | 9.58 |
| ILMN_3250850 | RFESD | -0.17 | 2.64 | 3.25 | 3.25 | 7.11 | 7.12 | 6.96 | 6.92 | 6.92 |
| ILMN_1723969 | PLCB1 | -0.17 | 0.59 | 0.04 | 6.40 | 8.87 | 8.88 | 8.82 | 8.87 | 8.36 |
| ILMN_1749213 | SDF2L1 | -0.17 | -0.65 | -1.02 | -3.39 | 9.03 | 9.06 | 9.13 | 9.19 | 9.57 |
| ILMN_1711208 | CELSR2 | -0.17 | -2.17 | -1.46 | -7.21 | 7.07 | 7.09 | 7.34 | 7.25 | 7.95 |
| ILMN_1769409 | C9orf123 | -0.16 | -2.58 | -1.59 | -3.35 | 7.93 | 7.95 | 8.24 | 8.12 | 8.33 |
| ILMN_3239955 | GNASAS | -0.16 | -0.84 | 0.52 | -3.57 | 6.87 | 6.88 | 6.94 | 6.82 | 7.16 |
| ILMN_1721113 | HLA-C | -0.16 | 1.14 | 0.37 | 2.79 | 8.17 | 8.19 | 8.00 | 8.12 | 7.76 |
| ILMN_3237534 | LOC100133517 | -0.16 | 0.08 | -0.40 | -3.40 | 7.36 | 7.37 | 7.35 | 7.39 | 7.64 |
| ILMN_1778177 | ZNF207 | -0.16 | 2.15 | 2.36 | 4.97 | 11.09 | 11.10 | 10.95 | 10.94 | 10.77 |
| ILMN_1744006 | GFOD2 | -0.16 | -1.10 | -2.04 | 3.19 | 7.87 | 7.88 | 7.94 | 8.00 | 7.67 |
| ILMN_2377862 | RAB6A | -0.16 | 1.09 | -0.44 | 3.35 | 8.12 | 8.14 | 7.98 | 8.18 | 7.69 |
| ILMN_1735199 | CIAPIN1 | -0.15 | 0.31 | -0.12 | 3.05 | 9.23 | 9.25 | 9.20 | 9.24 | 8.90 |
| ILMN_1756572 | COQ2 | -0.15 | 3.33 | 2.14 | 3.63 | 9.56 | 9.58 | 9.12 | 9.27 | 9.08 |
| ILMN_1842582 |  | -0.15 | 0.74 | 0.06 | -2.82 | 8.41 | 8.43 | 8.33 | 8.40 | 8.73 |
| ILMN_1756086 | INTS3 | -0.15 | -1.37 | -1.10 | -2.97 | 9.74 | 9.76 | 9.90 | 9.87 | 10.08 |
| ILMN_3266186 | HDAC7 | -0.15 | -3.00 | -1.61 | -3.95 | 7.07 | 7.08 | 7.34 | 7.21 | 7.43 |
| ILMN_1684255 | MYL4 | -0.14 | 0.50 | 0.04 | -3.45 | 6.77 | 6.78 | 6.72 | 6.76 | 7.09 |
| ILMN_1900998 |  | -0.14 | 1.13 | -0.19 | 4.13 | 7.31 | 7.32 | 7.23 | 7.33 | 7.02 |
| ILMN_3263329 | GAR1 | -0.14 | 0.91 | 0.80 | 6.48 | 10.79 | 10.81 | 10.68 | 10.70 | 10.01 |
| ILMN_1814173 | SMARCA4 | -0.14 | -0.03 | 0.41 | 3.87 | 11.31 | 11.32 | 11.31 | 11.26 | 10.84 |
| ILMN_1803945 | HCP5 | -0.14 | 3.04 | 2.27 | 5.13 | 10.85 | 10.89 | 10.11 | 10.30 | 9.60 |
| ILMN_1833858 |  | -0.13 | -1.57 | -3.59 | 3.47 | 9.62 | 9.65 | 9.93 | 10.33 | 8.94 |
| ILMN_2397627 | UPF3B | -0.13 | 2.68 | 2.68 | 3.35 | 9.13 | 9.15 | 8.79 | 8.79 | 8.71 |
| ILMN_1712400 | SERPINB6 | -0.13 | -0.55 | -0.55 | -4.01 | 6.97 | 6.98 | 7.03 | 7.03 | 7.38 |
| ILMN_2276820 | NSL1 | -0.13 | 1.17 | 0.85 | 2.84 | 7.11 | 7.13 | 6.99 | 7.02 | 6.81 |
| ILMN_1660436 | HSPA1B | -0.12 | 1.69 | -0.17 | 3.72 | 10.39 | 10.43 | 9.90 | 10.44 | 9.30 |
| ILMN_2261416 | CD3D | -0.12 | 0.43 | 1.86 | 4.46 | 7.41 | 7.42 | 7.36 | 7.21 | 6.93 |
| ILMN_2185675 | FAM159A | -0.12 | -1.76 | -1.64 | -3.47 | 6.68 | 6.69 | 6.87 | 6.86 | 7.06 |
| ILMN_1771799 | ZNF708 | -0.12 | -0.79 | 0.00 | 4.44 | 6.91 | 6.92 | 6.95 | 6.91 | 6.67 |
| ILMN_1724233 | LOC145783 | -0.12 | 0.51 | 0.04 | 3.03 | 7.01 | 7.02 | 6.97 | 7.01 | 6.76 |
| ILMN_3233179 | LOC728969 | -0.12 | -1.55 | -2.68 | -3.84 | 7.45 | 7.46 | 7.59 | 7.68 | 7.78 |
| ILMN_1719143 | APOBEC2 | -0.12 | -1.88 | -2.71 | 4.47 | 8.34 | 8.37 | 8.78 | 8.97 | 7.31 |
| ILMN_2315208 | CAMK2B | -0.11 | -2.46 | -2.31 | -3.44 | 6.82 | 6.83 | 7.04 | 7.02 | 7.12 |
| ILMN_1753500 | ARHGAP12 | -0.11 | 0.03 | -0.31 | 2.74 | 8.18 | 8.19 | 8.18 | 8.22 | 7.85 |
| ILMN_2414027 | CKLF | -0.11 | -0.09 | 1.20 | 4.06 | 9.76 | 9.78 | 9.77 | 9.58 | 9.14 |
| ILMN_1692948 | CCDC90B | -0.11 | 1.34 | 0.39 | 3.15 | 8.38 | 8.39 | 8.17 | 8.32 | 7.89 |
| ILMN_1763011 | C7orf10 | -0.11 | -0.83 | -2.06 | -2.94 | 7.15 | 7.16 | 7.25 | 7.41 | 7.52 |
| ILMN_2173611 | MT1E | -0.11 | 0.46 | 5.84 | 4.22 | 9.39 | 9.42 | 9.29 | 8.12 | 8.47 |
| ILMN_1660837 | CLCN3 | -0.11 | 1.74 | -0.39 | 6.56 | 8.92 | 8.93 | 8.76 | 8.96 | 8.30 |
| ILMN_1769031 | FRAP1 | -0.11 | 0.38 | 1.35 | -2.74 | 9.10 | 9.11 | 9.04 | 8.88 | 9.53 |
| ILMN_1882284 |  | -0.10 | -1.01 | 0.94 | -2.96 | 7.05 | 7.06 | 7.15 | 6.96 | 7.33 |
| ILMN_1810387 | PLAA | -0.09 | 2.04 | 1.67 | 3.93 | 8.52 | 8.53 | 8.30 | 8.34 | 8.10 |
| ILMN_1720344 | NIPA2 | -0.09 | 1.32 | 1.16 | 2.86 | 11.11 | 11.12 | 10.97 | 10.99 | 10.81 |
| ILMN_2350114 | TRIM13 | -0.09 | -0.97 | -1.03 | -2.74 | 7.67 | 7.68 | 7.77 | 7.78 | 7.96 |
| ILMN_1697286 | SF3A1 | -0.09 | -2.73 | -3.42 | -4.76 | 7.79 | 7.80 | 7.99 | 8.04 | 8.13 |
| ILMN_1671839 | TAF1C | -0.09 | -2.12 | -0.71 | 2.86 | 9.95 | 9.96 | 10.25 | 10.05 | 9.53 |
| ILMN_2396982 | BCL2L12 | -0.09 | -0.98 | -1.47 | -2.93 | 9.45 | 9.46 | 9.56 | 9.61 | 9.77 |
| ILMN_1784178 | IFT172 | -0.09 | -2.37 | -1.80 | -4.22 | 7.11 | 7.12 | 7.29 | 7.25 | 7.43 |
| ILMN_1751816 | MCTS1 | -0.09 | 2.00 | 0.69 | 6.76 | 10.06 | 10.07 | 9.83 | 9.98 | 9.27 |
| ILMN_1693310 | ITFG1 | -0.08 | 3.29 | 1.20 | 5.83 | 9.55 | 9.56 | 9.15 | 9.40 | 8.84 |
| ILMN_2406873 | ARFGAP1 | -0.08 | -1.52 | -0.57 | -2.99 | 7.09 | 7.10 | 7.21 | 7.14 | 7.33 |
| ILMN_1796458 | GABARAPL2 | -0.08 | 0.78 | 0.33 | 8.96 | 10.74 | 10.74 | 10.67 | 10.71 | 10.01 |
| ILMN_3250023 | CLEC2D | -0.08 | -0.53 | 0.93 | -4.65 | 6.82 | 6.83 | 6.86 | 6.74 | 7.20 |
| ILMN_2208495 | LASS5 | -0.08 | -1.08 | -0.50 | -2.87 | 7.93 | 7.94 | 8.07 | 8.00 | 8.31 |
| ILMN_1662658 | PUS1 | -0.07 | -4.06 | -2.25 | -4.56 | 9.99 | 10.00 | 10.37 | 10.20 | 10.41 |
| ILMN_1727466 | KCNMB4 | -0.07 | -1.17 | 0.58 | -4.29 | 7.46 | 7.47 | 7.68 | 7.35 | 8.25 |
| ILMN_1803398 | SRF | -0.07 | -1.89 | -2.41 | -4.42 | 9.48 | 9.49 | 9.75 | 9.82 | 10.11 |
| ILMN_3307827 | DNAJC4 | -0.07 | 0.10 | 0.59 | -4.10 | 8.30 | 8.31 | 8.29 | 8.25 | 8.70 |
| ILMN_1664233 | LOC644590 | -0.07 | 0.00 | -0.51 | -2.77 | 7.19 | 7.20 | 7.19 | 7.24 | 7.46 |
| ILMN_1667034 | PDPR | -0.07 | -0.27 | 0.00 | 4.54 | 9.05 | 9.06 | 9.09 | 9.05 | 8.38 |
| ILMN_1750722 | RPS7 | -0.07 | 0.37 | 0.70 | -4.49 | 8.99 | 9.00 | 8.95 | 8.92 | 9.44 |
| ILMN_1769575 | JAM3 | -0.07 | 0.07 | -0.73 | -3.08 | 9.56 | 9.57 | 9.55 | 9.67 | 10.02 |
| ILMN_3299955 | LOC728937 | -0.07 | 0.07 | -0.37 | -2.85 | 12.85 | 12.86 | 12.84 | 12.91 | 13.29 |
| ILMN_1757845 | SPIRE1 | -0.06 | -1.60 | -0.11 | -3.79 | 7.46 | 7.47 | 7.71 | 7.48 | 8.05 |
| ILMN_2069945 | SNRNP27 | -0.06 | 1.41 | 0.58 | 2.75 | 9.71 | 9.72 | 9.56 | 9.65 | 9.41 |
| ILMN_3236156 | OSTC | -0.06 | 0.36 | 0.06 | 3.02 | 11.60 | 11.61 | 11.56 | 11.60 | 11.26 |
| ILMN_1723287 | SLC6A16 | -0.06 | -0.82 | 0.73 | -6.38 | 7.65 | 7.66 | 7.74 | 7.57 | 8.37 |
| ILMN_1778977 | TYROBP | -0.06 | 0.19 | -0.75 | -3.10 | 8.75 | 8.77 | 8.70 | 8.96 | 9.63 |
| ILMN_1802753 | TSSC4 | -0.06 | -0.11 | -0.11 | -2.77 | 9.53 | 9.54 | 9.55 | 9.55 | 9.86 |
| ILMN_1672176 | SMPD2 | -0.06 | -1.69 | -1.47 | -3.72 | 6.83 | 6.83 | 6.93 | 6.92 | 7.05 |
| ILMN_1694027 | SESN3 | -0.05 | 1.24 | -0.88 | 3.26 | 7.51 | 7.52 | 7.43 | 7.57 | 7.30 |
| ILMN_2376205 | LTB | -0.05 | 0.76 | 4.72 | -1.34 | 8.70 | 8.71 | 8.55 | 7.78 | 8.96 |
| ILMN_1652806 | ATP5J | -0.05 | 0.00 | 0.22 | -3.73 | 9.61 | 9.62 | 9.61 | 9.57 | 10.33 |
| ILMN_2098643 | FADS3 | -0.05 | -1.42 | -0.97 | -4.45 | 7.50 | 7.50 | 7.68 | 7.62 | 8.08 |
| ILMN_1731358 | ZNF532 | -0.05 | -2.64 | -2.01 | -3.66 | 6.86 | 6.87 | 7.21 | 7.13 | 7.34 |
| ILMN_1757415 | C1orf163 | -0.05 | 1.32 | 1.86 | -3.32 | 6.98 | 6.98 | 6.89 | 6.85 | 7.20 |
| ILMN_2412807 | DCTN1 | -0.05 | -0.10 | -0.57 | 4.25 | 10.07 | 10.07 | 10.07 | 10.11 | 9.77 |
| ILMN_1778401 | HLA-B | -0.05 | 0.89 | 1.06 | 3.26 | 11.76 | 11.77 | 11.57 | 11.54 | 11.07 |
| ILMN_1708095 | PANK2 | -0.05 | -0.41 | 1.54 | 2.89 | 9.61 | 9.62 | 9.64 | 9.50 | 9.40 |
| ILMN_1687546 | HSP90AA1 | -0.04 | 2.68 | 2.24 | 3.38 | 7.20 | 7.20 | 7.00 | 7.03 | 6.94 |
| ILMN_1735156 | SLC4A11 | -0.04 | -1.44 | 0.04 | -5.67 | 6.71 | 6.72 | 6.82 | 6.71 | 7.15 |
| ILMN_1656977 | HIBCH | -0.04 | 2.51 | 3.04 | 3.44 | 9.42 | 9.42 | 9.02 | 8.94 | 8.88 |
| ILMN_2364072 | CLCNKA | -0.04 | -0.21 | -0.54 | -2.99 | 6.66 | 6.67 | 6.68 | 6.71 | 6.90 |
| ILMN_2137499 | LOC401010 | -0.04 | -1.09 | -0.70 | -3.67 | 6.97 | 6.98 | 7.07 | 7.03 | 7.29 |
| ILMN_1807981 | SIGIRR | -0.04 | -2.27 | -3.00 | -6.25 | 8.51 | 8.52 | 8.91 | 9.04 | 9.62 |
| ILMN_2120695 | TSPAN7 | -0.04 | -1.69 | -1.30 | -4.50 | 6.64 | 6.64 | 6.80 | 6.76 | 7.06 |
| ILMN_2100957 | CTAG1B | -0.04 | -1.34 | -0.92 | -3.93 | 7.35 | 7.36 | 7.48 | 7.44 | 7.72 |
| ILMN_1692517 | LOC653381 | -0.03 | 1.76 | 1.08 | 5.85 | 11.94 | 11.94 | 11.76 | 11.83 | 11.34 |
| ILMN_1712751 | HADHA | -0.03 | 0.67 | 0.42 | 2.98 | 8.99 | 8.99 | 8.92 | 8.94 | 8.68 |
| ILMN_1726597 | FAM65B | -0.03 | 1.65 | 2.69 | -3.46 | 9.70 | 9.71 | 9.52 | 9.41 | 10.08 |
| ILMN_2184525 | LOC220115 | -0.03 | -0.43 | -0.32 | -7.86 | 6.61 | 6.62 | 6.66 | 6.65 | 7.52 |
| ILMN_1735052 | ULK1 | -0.03 | -3.94 | -1.77 | -2.92 | 8.39 | 8.39 | 8.85 | 8.60 | 8.73 |
| ILMN_2320853 | UBE2D3 | -0.03 | 1.56 | 0.24 | 3.31 | 11.59 | 11.59 | 11.39 | 11.56 | 11.18 |
| ILMN_3233930 | LOC390557 | -0.03 | -1.22 | -0.47 | -5.06 | 10.47 | 10.48 | 10.63 | 10.53 | 11.12 |
| ILMN_2056479 | TXNL2 | -0.03 | 2.65 | 1.49 | 3.01 | 10.63 | 10.64 | 10.29 | 10.44 | 10.24 |
| ILMN_1675612 | BLCAP | -0.02 | -0.56 | 0.29 | -3.75 | 8.85 | 8.85 | 8.92 | 8.81 | 9.36 |
| ILMN_2343332 | TAF9 | -0.02 | 2.82 | 1.61 | 3.08 | 8.52 | 8.52 | 8.09 | 8.27 | 8.05 |
| ILMN_1779401 | CHP | -0.02 | 1.63 | 0.68 | 3.60 | 9.97 | 9.97 | 9.71 | 9.86 | 9.40 |
| ILMN_1720287 | E4F1 | 0.00 | 0.04 | -0.52 | -4.05 | 9.54 | 9.54 | 9.53 | 9.58 | 9.88 |
| ILMN_1707308 | IKBKG | 0.00 | 0.21 | -0.25 | -3.15 | 8.35 | 8.35 | 8.34 | 8.37 | 8.60 |
| ILMN_1809963 | RSAD1 | 0.00 | -1.66 | -2.31 | -4.60 | 7.75 | 7.75 | 7.91 | 7.97 | 8.19 |
| ILMN_1653134 | TMEM188 | 0.00 | 0.56 | 1.39 | 5.72 | 8.76 | 8.76 | 8.69 | 8.60 | 8.11 |
| ILMN_1715674 | ITPK1 | 0.00 | -1.70 | -0.71 | -3.18 | 8.52 | 8.52 | 8.72 | 8.61 | 8.90 |
| ILMN_1769259 | ANO9 | 0.00 | -1.55 | 0.24 | -3.77 | 6.78 | 6.78 | 6.89 | 6.77 | 7.04 |
| ILMN_1672331 | MAP3K7IP2 | 0.00 | 0.31 | -0.18 | 3.48 | 7.28 | 7.28 | 7.26 | 7.30 | 7.02 |
| ILMN_1721963 | MEN1 | 0.00 | -1.65 | -0.78 | -3.56 | 7.25 | 7.25 | 7.38 | 7.31 | 7.52 |
| ILMN_1734153 | GDI1 | 0.00 | -1.43 | -1.07 | 2.89 | 7.90 | 7.90 | 8.02 | 7.99 | 7.65 |
| ILMN_1730047 | C10orf141 | 0.00 | -0.92 | -1.97 | 3.15 | 6.97 | 6.97 | 7.07 | 7.17 | 6.65 |
| ILMN_1756469 | GAMT | 0.00 | 0.33 | -1.27 | -4.09 | 6.91 | 6.91 | 6.88 | 7.04 | 7.33 |
| ILMN_1664098 | FASTK | 0.00 | -0.63 | -1.95 | -3.99 | 7.80 | 7.80 | 7.86 | 8.00 | 8.22 |
| ILMN_1721712 | SYNGR1 | 0.00 | -0.30 | -1.40 | -4.19 | 7.18 | 7.18 | 7.21 | 7.33 | 7.65 |
| ILMN_1669310 | RHOT2 | 0.00 | -1.70 | -0.46 | -3.70 | 7.96 | 7.96 | 8.15 | 8.01 | 8.37 |
| ILMN_1673138 | ZBTB33 | 0.00 | 1.65 | 0.80 | 4.49 | 10.76 | 10.76 | 10.63 | 10.70 | 10.42 |
| ILMN_1689518 | PECAM1 | 0.00 | 2.40 | 1.22 | 4.44 | 7.97 | 7.97 | 7.73 | 7.85 | 7.53 |
| ILMN_1690040 | TM7SF2 | 0.00 | -1.01 | -0.20 | -3.40 | 8.52 | 8.52 | 8.69 | 8.55 | 9.08 |
| ILMN_1720158 | ETS2 | 0.00 | -0.59 | 0.07 | -4.62 | 7.23 | 7.23 | 7.29 | 7.23 | 7.71 |
| ILMN_1732809 | ALG9 | 0.00 | 0.47 | -0.72 | 3.17 | 9.30 | 9.30 | 9.26 | 9.35 | 9.05 |
| ILMN_1749253 | TUBD1 | 0.00 | 1.55 | 1.13 | 3.31 | 8.38 | 8.38 | 8.21 | 8.26 | 8.01 |
| ILMN_1753468 | CD63 | 0.00 | -0.20 | -0.04 | -3.95 | 9.06 | 9.06 | 9.09 | 9.06 | 9.72 |
| ILMN_1762606 | AQP11 | 0.00 | -1.84 | 0.30 | -3.28 | 6.81 | 6.81 | 6.93 | 6.79 | 7.03 |
| ILMN_1784766 | MCM3AP | 0.00 | -0.28 | 0.40 | -3.14 | 9.32 | 9.32 | 9.34 | 9.28 | 9.58 |
| ILMN_1794473 | PHF17 | 0.00 | 1.25 | 0.65 | 5.05 | 6.96 | 6.96 | 6.88 | 6.92 | 6.65 |
| ILMN_1795839 | SCCPDH | 0.00 | -2.52 | -0.93 | -3.86 | 7.50 | 7.50 | 7.72 | 7.58 | 7.84 |
| ILMN_1817255 |  | 0.00 | -0.52 | 0.70 | -3.34 | 9.73 | 9.73 | 9.80 | 9.64 | 10.16 |
| ILMN_3275615 | LOC645249 | 0.00 | -1.22 | -0.43 | -3.41 | 7.25 | 7.25 | 7.37 | 7.29 | 7.59 |
| ILMN_2249018 | LOC389816 | 0.01 | -0.10 | 1.77 | 2.80 | 8.28 | 8.27 | 8.30 | 7.80 | 7.52 |
| ILMN_1756204 | RPS6KA4 | 0.02 | -0.50 | 0.10 | -3.19 | 8.01 | 8.01 | 8.09 | 8.00 | 8.53 |
| ILMN_1792820 | NOC2L | 0.02 | -0.34 | -0.21 | -2.97 | 7.57 | 7.57 | 7.62 | 7.60 | 8.04 |
| ILMN_1790953 | TBCB | 0.02 | -0.60 | -0.49 | -2.88 | 9.68 | 9.68 | 9.77 | 9.76 | 10.12 |
| ILMN_3238740 | CENPBD1 | 0.03 | 1.83 | 0.51 | 4.91 | 7.59 | 7.59 | 7.38 | 7.53 | 7.02 |
| ILMN_2365711 | 41519 | 0.03 | -0.29 | 0.98 | -2.73 | 11.67 | 11.67 | 11.71 | 11.56 | 11.99 |
| ILMN_1737991 | LOC650298 | 0.03 | -0.17 | -0.17 | -4.26 | 7.87 | 7.86 | 7.89 | 7.89 | 8.35 |
| ILMN_2410371 | CD79A | 0.03 | -1.69 | -2.25 | -3.22 | 7.04 | 7.04 | 7.22 | 7.28 | 7.38 |
| ILMN_1651385 | MFN2 | 0.03 | -0.80 | 0.10 | -3.51 | 8.24 | 8.24 | 8.33 | 8.23 | 8.61 |
| ILMN_2344079 | ZGPAT | 0.03 | -0.60 | -1.27 | -3.41 | 6.88 | 6.88 | 6.94 | 7.01 | 7.22 |
| ILMN_1701466 | PEX16 | 0.03 | -0.44 | 0.13 | -3.10 | 9.42 | 9.42 | 9.47 | 9.41 | 9.73 |
| ILMN_1814573 | FTSJD1 | 0.03 | -1.05 | 0.74 | 4.06 | 8.38 | 8.38 | 8.49 | 8.31 | 7.98 |
| ILMN_2223922 | AGTPBP1 | 0.03 | 0.38 | 0.77 | 2.85 | 7.35 | 7.34 | 7.31 | 7.27 | 7.07 |
| ILMN_2355559 | PSAP | 0.04 | 0.14 | 0.36 | -4.02 | 11.45 | 11.44 | 11.43 | 11.41 | 11.82 |
| ILMN_1694877 | CASP6 | 0.04 | 1.86 | 1.31 | 8.13 | 7.82 | 7.81 | 7.65 | 7.70 | 7.07 |
| ILMN_1720088 | SFRS12 | 0.04 | 0.72 | 1.63 | 3.94 | 8.84 | 8.84 | 8.78 | 8.70 | 8.49 |
| ILMN_1789196 | TPM2 | 0.04 | 0.57 | 0.38 | -6.30 | 8.21 | 8.20 | 8.11 | 8.14 | 9.31 |
| ILMN_2278112 | PRKACB | 0.04 | 0.12 | 1.19 | 2.81 | 7.02 | 7.02 | 7.01 | 6.92 | 6.78 |
| ILMN_1807088 | TTC33 | 0.04 | -0.50 | 2.37 | 3.50 | 8.08 | 8.07 | 8.12 | 7.87 | 7.78 |
| ILMN_2357134 | SPHK1 | 0.04 | -0.04 | 0.41 | -3.34 | 6.66 | 6.65 | 6.66 | 6.62 | 6.93 |
| ILMN_1785330 | SH3BP4 | 0.04 | 1.23 | 1.01 | 4.21 | 8.66 | 8.65 | 8.27 | 8.34 | 7.31 |
| ILMN_1775048 | DIRAS1 | 0.04 | -1.17 | -3.81 | -2.18 | 6.73 | 6.73 | 6.83 | 7.04 | 6.91 |
| ILMN_1666076 | ARHGEF4 | 0.04 | 0.56 | 1.25 | -3.59 | 6.59 | 6.59 | 6.55 | 6.50 | 6.87 |
| ILMN_1754860 | MRPS12 | 0.05 | -0.69 | -0.69 | -2.97 | 6.86 | 6.85 | 6.91 | 6.91 | 7.07 |
| ILMN_2378100 | FBXL5 | 0.05 | 1.01 | 1.15 | 4.50 | 7.89 | 7.88 | 7.81 | 7.80 | 7.56 |
| ILMN_1676336 | AADACL1 | 0.05 | 1.44 | -0.91 | 3.40 | 7.34 | 7.33 | 7.24 | 7.40 | 7.10 |
| ILMN_2339705 | MED8 | 0.05 | 0.51 | -1.03 | -4.11 | 7.34 | 7.33 | 7.30 | 7.40 | 7.60 |
| ILMN_1880937 |  | 0.05 | 0.13 | -1.16 | -4.04 | 8.15 | 8.14 | 8.13 | 8.30 | 8.67 |
| ILMN_1692072 | LOC728006 | 0.05 | 1.15 | 1.22 | 4.74 | 9.06 | 9.05 | 8.84 | 8.83 | 8.15 |
| ILMN_1877039 |  | 0.05 | -0.43 | -0.53 | -2.78 | 6.81 | 6.81 | 6.84 | 6.85 | 6.99 |
| ILMN_1674665 | CPNE2 | 0.06 | -0.44 | 0.00 | 3.53 | 6.86 | 6.86 | 6.89 | 6.86 | 6.65 |
| ILMN_1753241 | SNTA1 | 0.06 | -0.88 | -0.80 | -4.30 | 8.47 | 8.46 | 8.62 | 8.61 | 9.21 |
| ILMN_1804789 | KIAA1967 | 0.06 | -2.28 | -0.27 | -3.16 | 9.20 | 9.19 | 9.45 | 9.23 | 9.54 |
| ILMN_1710150 | EED | 0.06 | 2.17 | 3.07 | 5.71 | 8.69 | 8.68 | 8.45 | 8.36 | 8.07 |
| ILMN_2198408 | MFF | 0.06 | -1.40 | -0.69 | -3.48 | 8.76 | 8.75 | 8.98 | 8.87 | 9.31 |
| ILMN_2402558 | ZHX1 | 0.06 | 2.99 | 2.74 | 3.09 | 8.01 | 8.00 | 7.69 | 7.72 | 7.68 |
| ILMN_1783226 | SSR2 | 0.06 | -0.29 | -0.36 | -2.78 | 9.66 | 9.65 | 9.69 | 9.70 | 9.95 |
| ILMN_2377240 | AKTIP | 0.07 | 0.46 | 0.62 | 7.35 | 8.83 | 8.82 | 8.78 | 8.77 | 8.08 |
| ILMN_1737715 | OSR2 | 0.07 | 0.91 | 1.21 | 4.69 | 7.39 | 7.38 | 7.30 | 7.27 | 6.92 |
| ILMN_3274904 | LOC284230 | 0.07 | 2.23 | 1.37 | 3.12 | 14.01 | 14.00 | 13.79 | 13.88 | 13.71 |
| ILMN_1702787 | SEMA4A | 0.07 | 1.39 | 0.92 | -3.63 | 6.94 | 6.94 | 6.81 | 6.86 | 7.28 |
| ILMN_1760855 | OCRL | 0.08 | -1.17 | 0.57 | 3.61 | 7.79 | 7.78 | 7.93 | 7.72 | 7.34 |
| ILMN_1760493 | LIMS2 | 0.08 | -0.08 | 0.41 | -3.62 | 6.63 | 6.63 | 6.64 | 6.60 | 6.93 |
| ILMN_1700975 | ENSA | 0.08 | 0.92 | 0.08 | 4.17 | 7.26 | 7.25 | 7.18 | 7.25 | 6.92 |
| ILMN_1657139 | ADAT1 | 0.09 | 1.06 | -0.12 | 3.93 | 8.26 | 8.25 | 8.06 | 8.29 | 7.51 |
| ILMN_2347917 | EED | 0.09 | 2.34 | 2.00 | 3.32 | 9.29 | 9.27 | 8.94 | 8.99 | 8.79 |
| ILMN_2276397 | RASGRP2 | 0.09 | 0.14 | 1.36 | -3.09 | 6.97 | 6.96 | 6.96 | 6.87 | 7.20 |
| ILMN_3267017 | LOC100129028 | 0.09 | -0.12 | 0.36 | 3.31 | 13.65 | 13.64 | 13.67 | 13.61 | 13.29 |
| ILMN_1718558 | PARP12 | 0.09 | -1.31 | -0.74 | -2.93 | 8.12 | 8.10 | 8.31 | 8.22 | 8.54 |
| ILMN_1665526 | TCEA2 | 0.09 | -1.15 | 0.15 | -4.83 | 7.96 | 7.95 | 8.08 | 7.94 | 8.48 |
| ILMN_1695585 | RPS26L | 0.09 | -0.38 | -0.12 | -3.37 | 11.98 | 11.96 | 12.03 | 11.99 | 12.45 |
| ILMN_2340259 | PDE4B | 0.09 | -1.41 | 1.19 | 3.01 | 9.04 | 9.03 | 9.19 | 8.92 | 8.72 |
| ILMN_1715392 | PRPF3 | 0.10 | -1.68 | -0.55 | -3.83 | 10.09 | 10.07 | 10.32 | 10.16 | 10.62 |
| ILMN_1789123 | PLK4 | 0.10 | 1.30 | 0.10 | 5.64 | 10.06 | 10.04 | 9.88 | 10.04 | 9.29 |
| ILMN_2074044 | PLS1 | 0.10 | 1.78 | 1.19 | 4.89 | 7.04 | 7.04 | 6.92 | 6.96 | 6.71 |
| ILMN_1683082 | RPUSD1 | 0.10 | 0.18 | 0.26 | -3.24 | 7.56 | 7.55 | 7.54 | 7.53 | 7.98 |
| ILMN_1663397 | CAMK2B | 0.10 | -1.60 | -1.39 | -2.85 | 6.77 | 6.76 | 6.92 | 6.90 | 7.04 |
| ILMN_1705447 | AFG3L1 | 0.10 | -0.52 | 0.77 | 3.34 | 8.14 | 8.13 | 8.23 | 8.02 | 7.61 |
| ILMN_1692168 | UBE2Z | 0.11 | -1.76 | -0.70 | -3.59 | 8.43 | 8.42 | 8.59 | 8.49 | 8.77 |
| ILMN_2344650 | N4BP2L1 | 0.11 | 0.46 | 1.06 | 2.90 | 7.09 | 7.08 | 7.04 | 6.99 | 6.81 |
| ILMN_1781565 | MESDC1 | 0.11 | 0.83 | 0.11 | 2.73 | 7.68 | 7.67 | 7.58 | 7.67 | 7.35 |
| ILMN_2070044 | PPM1K | 0.11 | 1.76 | 0.53 | 3.55 | 7.51 | 7.50 | 7.30 | 7.45 | 7.09 |
| ILMN_2339779 | ATP6V1E1 | 0.11 | 0.30 | 0.75 | -2.79 | 10.73 | 10.71 | 10.68 | 10.62 | 11.14 |
| ILMN_1756220 | DDX18 | 0.11 | -2.27 | -0.57 | -4.29 | 10.62 | 10.61 | 10.89 | 10.69 | 11.12 |
| ILMN_2246154 | SCAND3 | 0.12 | 0.62 | 1.27 | 2.84 | 7.45 | 7.44 | 7.36 | 7.27 | 7.04 |
| ILMN_2288070 | FTO | 0.12 | -0.93 | 0.44 | 4.21 | 7.39 | 7.38 | 7.50 | 7.34 | 6.91 |
| ILMN_3235312 | LOC92659 | 0.12 | -0.24 | -1.95 | -3.67 | 7.23 | 7.22 | 7.26 | 7.45 | 7.64 |
| ILMN_2181540 | YY1 | 0.12 | 2.26 | 1.45 | 3.98 | 10.83 | 10.82 | 10.58 | 10.67 | 10.39 |
| ILMN_2046730 | S100A10 | 0.12 | 0.95 | -0.61 | 3.62 | 10.62 | 10.58 | 10.29 | 10.84 | 9.35 |
| ILMN_2413251 | EWSR1 | 0.13 | -0.92 | -2.90 | -2.92 | 8.66 | 8.64 | 8.78 | 9.04 | 9.04 |
| ILMN_2130441 | HLA-H | 0.13 | 1.33 | 0.73 | 2.91 | 11.77 | 11.74 | 11.52 | 11.63 | 11.23 |
| ILMN_1713534 | LDB3 | 0.13 | 0.03 | -0.10 | 3.69 | 7.24 | 7.22 | 7.23 | 7.25 | 6.85 |
| ILMN_3244096 | ZFC3H1 | 0.13 | -1.00 | -1.06 | -4.05 | 9.62 | 9.61 | 9.73 | 9.73 | 10.04 |
| ILMN_1781148 | ASB10 | 0.13 | 0.24 | 0.61 | -5.88 | 6.73 | 6.72 | 6.70 | 6.66 | 7.47 |
| ILMN_2400940 | L3MBTL | 0.13 | -0.34 | -0.34 | -3.68 | 7.05 | 7.03 | 7.08 | 7.08 | 7.41 |
| ILMN_1807212 | USP44 | 0.14 | 0.86 | 1.27 | 4.50 | 7.36 | 7.35 | 7.26 | 7.21 | 6.82 |
| ILMN_2355486 | FAM3B | 0.14 | -0.62 | -0.80 | -3.17 | 7.28 | 7.26 | 7.38 | 7.41 | 7.81 |
| ILMN_1746819 | C5 | 0.14 | 0.70 | 0.35 | 3.74 | 8.67 | 8.65 | 8.57 | 8.62 | 8.13 |
| ILMN_1685397 | ITGA3 | 0.14 | -1.03 | -0.37 | -2.79 | 6.95 | 6.93 | 7.07 | 6.99 | 7.28 |
| ILMN_2112755 | HSDL1 | 0.15 | 1.42 | -0.25 | 4.07 | 7.43 | 7.41 | 7.30 | 7.45 | 7.05 |
| ILMN_1703524 | SRP68 | 0.15 | -0.19 | -0.44 | -2.86 | 9.59 | 9.58 | 9.60 | 9.62 | 9.79 |
| ILMN_1796712 | S100A10 | 0.15 | 0.93 | -0.26 | 3.85 | 10.22 | 10.17 | 9.92 | 10.30 | 8.98 |
| ILMN_2311041 | MRPL52 | 0.15 | -0.42 | -0.34 | 3.90 | 8.02 | 8.00 | 8.08 | 8.07 | 7.49 |
| ILMN_1710216 | AVEN | 0.15 | 0.55 | 0.18 | 6.22 | 8.70 | 8.69 | 8.65 | 8.69 | 8.14 |
| ILMN_1806825 | C14orf145 | 0.15 | 1.07 | 0.85 | 2.84 | 8.12 | 8.11 | 8.02 | 8.04 | 7.86 |
| ILMN_1743199 | EGR2 | 0.15 | -1.73 | -0.34 | -3.93 | 7.03 | 7.01 | 7.30 | 7.08 | 7.64 |
| ILMN_1671728 | CARD14 | 0.15 | 0.42 | -0.46 | -3.99 | 7.83 | 7.81 | 7.79 | 7.87 | 8.17 |
| ILMN_1720282 | NQO1 | 0.15 | 1.11 | 1.08 | 3.73 | 7.58 | 7.57 | 7.49 | 7.49 | 7.26 |
| ILMN_1683980 | PLEKHM2 | 0.15 | -0.49 | 0.18 | -3.94 | 9.37 | 9.35 | 9.42 | 9.35 | 9.79 |
| ILMN_1671152 | LOC649801 | 0.16 | 1.37 | 0.25 | 3.82 | 7.19 | 7.17 | 7.04 | 7.16 | 6.78 |
| ILMN_1892403 | SNORD13 | 0.16 | 0.22 | 0.77 | 3.95 | 10.66 | 10.60 | 10.57 | 10.35 | 9.07 |
| ILMN_3251155 | PCBP2 | 0.16 | -2.01 | -0.24 | -3.41 | 7.50 | 7.48 | 7.76 | 7.53 | 7.93 |
| ILMN_1691156 | MT1A | 0.16 | 0.65 | 4.56 | 4.73 | 10.28 | 10.23 | 10.09 | 8.95 | 8.90 |
| ILMN_1734543 | PTPRE | 0.16 | -1.94 | -0.25 | -2.93 | 7.80 | 7.78 | 8.08 | 7.84 | 8.23 |
| ILMN_2383611 | PTPRE | 0.17 | -2.19 | -0.09 | -3.11 | 8.18 | 8.15 | 8.58 | 8.20 | 8.74 |
| ILMN_1691111 | SPATA2L | 0.17 | 0.36 | -1.11 | 3.07 | 7.83 | 7.81 | 7.79 | 7.96 | 7.46 |
| ILMN_3254492 | LOC100129650 | 0.17 | -1.22 | -0.13 | -3.20 | 9.83 | 9.79 | 10.07 | 9.85 | 10.46 |
| ILMN_1691559 | ELF2 | 0.17 | -0.02 | 0.19 | 5.01 | 9.45 | 9.43 | 9.46 | 9.42 | 8.67 |
| ILMN_1770623 | FAM58A | 0.17 | -1.00 | -1.61 | -3.57 | 8.49 | 8.48 | 8.59 | 8.65 | 8.84 |
| ILMN_1792669 | HLCS | 0.17 | -1.37 | -0.60 | -2.96 | 7.10 | 7.09 | 7.21 | 7.15 | 7.33 |
| ILMN_1700628 | DDX24 | 0.17 | -0.46 | 1.35 | -2.75 | 9.94 | 9.92 | 9.99 | 9.78 | 10.26 |
| ILMN_1781752 | CLEC16A | 0.17 | 0.14 | 0.25 | 2.80 | 9.76 | 9.72 | 9.73 | 9.70 | 9.16 |
| ILMN_1729453 | TSPAN9 | 0.17 | 1.01 | 1.29 | 3.76 | 7.89 | 7.86 | 7.70 | 7.65 | 7.17 |
| ILMN_2382488 | CTU2 | 0.18 | -0.36 | 0.36 | 2.89 | 7.21 | 7.19 | 7.24 | 7.17 | 6.94 |
| ILMN_1665212 | EDC4 | 0.18 | -1.71 | 0.27 | 4.86 | 9.82 | 9.80 | 10.01 | 9.79 | 9.28 |
| ILMN_1727605 | FRK | 0.18 | 1.64 | 2.36 | 4.00 | 6.98 | 6.96 | 6.83 | 6.76 | 6.61 |
| ILMN_1744647 | CAND1 | 0.18 | -0.94 | -0.09 | -3.61 | 8.50 | 8.48 | 8.60 | 8.51 | 8.90 |
| ILMN_1772261 | GLG1 | 0.18 | -0.73 | -0.91 | 7.98 | 10.06 | 10.05 | 10.11 | 10.13 | 9.48 |
| ILMN_2123119 | MFSD8 | 0.19 | 1.80 | 2.10 | 3.64 | 8.02 | 8.00 | 7.80 | 7.76 | 7.57 |
| ILMN_2390821 | THOC2 | 0.19 | 0.67 | -0.35 | 4.39 | 9.41 | 9.38 | 9.32 | 9.45 | 8.86 |
| ILMN_2399497 | RFXANK | 0.19 | -0.35 | -0.19 | -2.80 | 7.66 | 7.64 | 7.70 | 7.68 | 7.96 |
| ILMN_1665982 | AKTIP | 0.19 | 0.04 | -0.04 | 5.52 | 7.64 | 7.62 | 7.63 | 7.64 | 7.16 |
| ILMN_2349658 | TSPO | 0.20 | -0.27 | -1.99 | -2.95 | 7.33 | 7.29 | 7.37 | 7.67 | 7.83 |
| ILMN_3249032 | EPCAM | 0.20 | -1.26 | -2.55 | -4.50 | 7.03 | 7.01 | 7.16 | 7.29 | 7.49 |
| ILMN_1690252 | ALKBH2 | 0.20 | -1.83 | -0.17 | -5.68 | 8.72 | 8.70 | 8.90 | 8.73 | 9.29 |
| ILMN_1706531 | ABCC5 | 0.20 | 0.12 | 0.08 | -3.01 | 7.07 | 7.05 | 7.06 | 7.06 | 7.32 |
| ILMN_1880103 |  | 0.20 | -0.20 | 0.00 | -2.76 | 7.11 | 7.09 | 7.13 | 7.11 | 7.34 |
| ILMN_1803256 | STOX2 | 0.20 | -3.52 | -0.81 | -5.08 | 6.95 | 6.92 | 7.47 | 7.07 | 7.70 |
| ILMN_1690352 | ADO | 0.20 | -1.43 | 0.20 | -2.90 | 10.24 | 10.22 | 10.38 | 10.22 | 10.52 |
| ILMN_1656185 | DEF8 | 0.21 | 1.56 | 0.19 | 3.66 | 8.85 | 8.81 | 8.55 | 8.81 | 8.14 |
| ILMN_1806907 | PAWR | 0.21 | 0.99 | 0.95 | 4.82 | 7.98 | 7.94 | 7.79 | 7.79 | 7.05 |
| ILMN_1686109 | CCL23 | 0.21 | -0.85 | -0.47 | -9.91 | 6.79 | 6.77 | 6.85 | 6.82 | 7.56 |
| ILMN_1807243 | PRPF18 | 0.22 | 0.76 | 1.31 | 3.20 | 8.04 | 8.02 | 7.97 | 7.92 | 7.75 |
| ILMN_1717886 | PKHD1L1 | 0.22 | 1.17 | 1.51 | 2.86 | 7.43 | 7.40 | 7.27 | 7.22 | 7.04 |
| ILMN_1764410 | C22orf13 | 0.22 | -1.30 | -2.20 | -3.15 | 9.54 | 9.50 | 9.73 | 9.87 | 10.01 |
| ILMN_1815924 | NUP107 | 0.22 | 0.16 | 0.79 | -4.40 | 9.57 | 9.55 | 9.56 | 9.49 | 10.04 |
| ILMN_1656933 | NKPD1 | 0.22 | 0.50 | -0.45 | -3.35 | 6.68 | 6.66 | 6.65 | 6.70 | 6.88 |
| ILMN_1715189 | LHX6 | 0.22 | -0.85 | 0.58 | -4.05 | 6.68 | 6.66 | 6.74 | 6.63 | 6.98 |
| ILMN_1720083 | EHD4 | 0.23 | 0.49 | 0.00 | 4.06 | 9.09 | 9.05 | 9.01 | 9.09 | 8.38 |
| ILMN_1892638 |  | 0.23 | -0.68 | -2.38 | -4.06 | 8.86 | 8.83 | 8.97 | 9.25 | 9.52 |
| ILMN_1799487 | N4BP2L1 | 0.23 | 0.92 | 2.48 | 2.82 | 7.06 | 7.04 | 6.98 | 6.84 | 6.81 |
| ILMN_1774258 | L3MBTL | 0.23 | -1.60 | -0.43 | -3.50 | 6.97 | 6.94 | 7.13 | 7.01 | 7.32 |
| ILMN_2321416 | DIAPH1 | 0.23 | -0.60 | -0.54 | -2.85 | 9.23 | 9.20 | 9.31 | 9.30 | 9.60 |
| ILMN_1692785 | KLHL21 | 0.23 | -0.90 | -0.19 | -4.76 | 7.44 | 7.42 | 7.52 | 7.46 | 7.85 |
| ILMN_1795918 | CENTG3 | 0.24 | -0.87 | -1.46 | -3.58 | 7.61 | 7.58 | 7.73 | 7.82 | 8.12 |
| ILMN_1673553 | PTH2 | 0.24 | -0.33 | -0.05 | -3.15 | 6.67 | 6.65 | 6.69 | 6.67 | 6.89 |
| ILMN_1805396 | LOC168474 | 0.24 | 2.15 | 0.24 | 3.42 | 7.33 | 7.31 | 7.15 | 7.31 | 7.04 |
| ILMN_3209832 | LOC100131801 | 0.24 | 1.73 | 1.22 | -3.29 | 11.31 | 11.28 | 11.11 | 11.17 | 11.67 |
| ILMN_1764788 | TNFRSF1B | 0.24 | 0.08 | -1.88 | -3.56 | 7.04 | 7.02 | 7.04 | 7.20 | 7.34 |
| ILMN_1720703 | SLC25A3 | 0.25 | 0.04 | -0.49 | -3.40 | 6.92 | 6.90 | 6.92 | 6.97 | 7.25 |
| ILMN_1700674 | LOC728481 | 0.25 | 3.12 | 2.68 | 3.07 | 13.16 | 13.13 | 12.74 | 12.80 | 12.74 |
| ILMN_2365248 | HVCN1 | 0.25 | -0.11 | 0.14 | -3.58 | 6.86 | 6.83 | 6.87 | 6.84 | 7.30 |
| ILMN_1745343 | ZMAT2 | 0.25 | 1.35 | 4.88 | 2.87 | 8.95 | 8.93 | 8.84 | 8.56 | 8.72 |
| ILMN_1767470 | SCPEP1 | 0.25 | -1.95 | -1.95 | -5.44 | 8.63 | 8.61 | 8.84 | 8.84 | 9.22 |
| ILMN_1682332 | GYPC | 0.25 | -1.04 | 0.47 | -4.37 | 8.38 | 8.34 | 8.53 | 8.31 | 9.02 |
| ILMN_1726410 | APRT | 0.25 | 1.19 | 0.71 | 5.61 | 9.75 | 9.71 | 9.57 | 9.64 | 8.93 |
| ILMN_2412380 | TSC22D1 | 0.25 | 0.09 | 0.57 | 3.88 | 7.81 | 7.79 | 7.80 | 7.75 | 7.40 |
| ILMN_2149053 | RIF1 | 0.25 | 0.54 | 3.85 | 1.39 | 8.75 | 8.73 | 8.70 | 8.35 | 8.61 |
| ILMN_1729319 | USP7 | 0.26 | -0.75 | 0.86 | 3.01 | 9.06 | 9.02 | 9.19 | 8.92 | 8.55 |
| ILMN_2078659 | DHFRL1 | 0.26 | 2.27 | 1.37 | 2.91 | 6.87 | 6.85 | 6.70 | 6.77 | 6.65 |
| ILMN_1665557 | USP15 | 0.26 | -0.68 | 0.96 | -3.12 | 8.35 | 8.32 | 8.44 | 8.23 | 8.75 |
| ILMN_1665964 | GAB2 | 0.26 | 0.24 | -0.52 | 5.52 | 8.15 | 8.10 | 8.11 | 8.23 | 7.23 |
| ILMN_1800573 | RPS21 | 0.26 | -0.12 | 0.02 | -2.77 | 10.83 | 10.78 | 10.86 | 10.83 | 11.36 |
| ILMN_1758034 | ETFDH | 0.26 | 1.49 | 1.41 | 3.95 | 8.34 | 8.31 | 8.15 | 8.16 | 7.84 |
| ILMN_2406169 | PKIG | 0.26 | -0.19 | -1.95 | -4.91 | 7.13 | 7.10 | 7.14 | 7.30 | 7.56 |
| ILMN_2362293 | FBXO38 | 0.26 | -0.76 | 1.48 | 2.99 | 8.33 | 8.30 | 8.41 | 8.18 | 8.03 |
| ILMN_1752294 | PCDH9 | 0.26 | -1.02 | 0.71 | 4.03 | 7.25 | 7.23 | 7.34 | 7.19 | 6.90 |
| ILMN_1805842 | FHL1 | 0.27 | 0.22 | -2.08 | 2.78 | 7.42 | 7.38 | 7.39 | 7.71 | 7.04 |
| ILMN_1707534 | NKRF | 0.27 | 0.83 | 1.48 | 3.00 | 8.98 | 8.96 | 8.91 | 8.85 | 8.72 |
| ILMN_3248906 | ZC4H2 | 0.27 | -0.59 | 0.19 | 3.00 | 7.15 | 7.12 | 7.22 | 7.13 | 6.78 |
| ILMN_1759772 | LRRC56 | 0.27 | -1.35 | -1.58 | -3.92 | 7.31 | 7.29 | 7.43 | 7.45 | 7.65 |
| ILMN_2076658 | MRPL1 | 0.27 | 0.97 | 1.49 | 3.28 | 9.82 | 9.78 | 9.67 | 9.58 | 9.30 |
| ILMN_1773847 | DYNC1I2 | 0.27 | 2.32 | 2.59 | 2.89 | 9.56 | 9.52 | 9.27 | 9.24 | 9.20 |
| ILMN_1696133 | SELI | 0.27 | -1.16 | -0.81 | -3.13 | 7.96 | 7.94 | 8.06 | 8.03 | 8.23 |
| ILMN_1720542 | POLR2I | 0.27 | -1.45 | -0.82 | -2.93 | 9.81 | 9.78 | 9.97 | 9.90 | 10.13 |
| ILMN_1812926 | ANTXR2 | 0.28 | 3.31 | -1.15 | 3.53 | 8.77 | 8.74 | 8.42 | 8.90 | 8.39 |
| ILMN_1794594 | RASGRP2 | 0.28 | -0.84 | -0.84 | -4.81 | 7.82 | 7.79 | 7.93 | 7.93 | 8.45 |
| ILMN_1658472 | APH1A | 0.28 | -1.40 | -0.59 | -3.23 | 10.19 | 10.16 | 10.34 | 10.26 | 10.54 |
| ILMN_1671693 | C18orf10 | 0.28 | -0.03 | -0.62 | 3.17 | 8.71 | 8.68 | 8.72 | 8.78 | 8.37 |
| ILMN_1692511 | TMEM106C | 0.28 | -1.38 | -0.04 | -3.96 | 9.38 | 9.34 | 9.59 | 9.39 | 9.99 |
| ILMN_1798619 | KCTD17 | 0.28 | -1.05 | -0.97 | -2.95 | 7.05 | 7.02 | 7.13 | 7.13 | 7.29 |
| ILMN_1673518 | BRWD1 | 0.29 | -2.95 | -0.73 | -4.09 | 8.36 | 8.33 | 8.67 | 8.43 | 8.79 |
| ILMN_2374633 | ZWILCH | 0.29 | 2.13 | 2.15 | 2.75 | 10.28 | 10.24 | 10.01 | 10.00 | 9.93 |
| ILMN_1678522 | LOC644934 | 0.29 | 0.29 | -0.57 | -3.71 | 10.49 | 10.44 | 10.44 | 10.58 | 11.09 |
| ILMN_1751034 | ITPRIPL2 | 0.29 | -2.25 | -3.01 | -3.85 | 8.37 | 8.33 | 8.68 | 8.79 | 8.91 |
| ILMN_1769601 | MGC16169 | 0.29 | 1.18 | 0.56 | 4.52 | 8.76 | 8.71 | 8.57 | 8.67 | 8.03 |
| ILMN_1770425 | CDIPT | 0.29 | -0.62 | -0.55 | -3.40 | 8.01 | 7.98 | 8.08 | 8.07 | 8.36 |
| ILMN_1732300 | POLR2C | 0.29 | 0.94 | 0.32 | 3.55 | 8.89 | 8.86 | 8.78 | 8.85 | 8.45 |
| ILMN_1803799 | LOC649555 | 0.30 | 0.79 | 0.73 | 2.88 | 9.45 | 9.38 | 9.27 | 9.28 | 8.77 |
| ILMN_1776005 | OSTC | 0.30 | 1.15 | 1.19 | 2.83 | 10.74 | 10.68 | 10.52 | 10.51 | 10.20 |
| ILMN_1654639 | HERC6 | 0.30 | 1.54 | 1.44 | 3.35 | 7.48 | 7.43 | 7.23 | 7.24 | 6.93 |
| ILMN_1713088 | MSI2 | 0.30 | 0.68 | -0.68 | 3.26 | 8.00 | 7.97 | 7.94 | 8.06 | 7.71 |
| ILMN_1808041 | RPL10A | 0.30 | 2.13 | 1.72 | 3.09 | 11.90 | 11.86 | 11.62 | 11.68 | 11.50 |
| ILMN_1716922 | DHX16 | 0.30 | -0.68 | -0.88 | -3.04 | 9.65 | 9.62 | 9.71 | 9.73 | 9.92 |
| ILMN_1767113 | AOX1 | 0.30 | 1.83 | 2.97 | 3.20 | 6.91 | 6.88 | 6.75 | 6.65 | 6.63 |
| ILMN_1673962 | NUP205 | 0.31 | -0.76 | -0.15 | -3.39 | 10.10 | 10.07 | 10.19 | 10.12 | 10.47 |
| ILMN_2413779 | SEZ6L2 | 0.31 | -1.01 | -1.13 | -5.97 | 6.81 | 6.77 | 6.96 | 6.97 | 7.66 |
| ILMN_1698100 | ANXA2P1 | 0.31 | 1.49 | -0.29 | 2.89 | 8.27 | 8.21 | 8.01 | 8.32 | 7.77 |
| ILMN_3255770 | LOC100129878 | 0.31 | 2.01 | 4.09 | 3.74 | 7.36 | 7.33 | 7.17 | 6.97 | 7.00 |
| ILMN_2101885 | TUBB | 0.31 | -0.51 | -0.55 | 4.04 | 12.31 | 12.28 | 12.35 | 12.36 | 11.97 |
| ILMN_1731353 | CHPF | 0.31 | -0.75 | 0.13 | -3.39 | 7.52 | 7.49 | 7.60 | 7.51 | 7.88 |
| ILMN_1718621 | TSPAN32 | 0.32 | 0.81 | 1.33 | -4.00 | 7.00 | 6.97 | 6.92 | 6.87 | 7.38 |
| ILMN_1702065 | MFSD5 | 0.32 | 0.25 | -0.85 | -4.49 | 7.82 | 7.79 | 7.80 | 7.91 | 8.30 |
| ILMN_1781764 | HNRNPH2 | 0.32 | 0.67 | 0.81 | 3.67 | 7.46 | 7.43 | 7.40 | 7.39 | 7.12 |
| ILMN_1657977 | MSRB2 | 0.32 | 1.07 | -0.84 | 3.53 | 8.57 | 8.53 | 8.44 | 8.66 | 8.16 |
| ILMN_1700518 | HMGN4 | 0.32 | 2.46 | 1.60 | 3.10 | 10.17 | 10.13 | 9.91 | 10.00 | 9.84 |
| ILMN_1656670 | HLA-G | 0.32 | 2.03 | 0.98 | 3.25 | 8.75 | 8.68 | 8.32 | 8.54 | 8.07 |
| ILMN_1696302 | FABP5 | 0.32 | 1.34 | 1.08 | 2.74 | 8.99 | 8.93 | 8.75 | 8.80 | 8.50 |
| ILMN_1697095 | SUZ12P | 0.32 | 0.04 | -1.29 | 2.94 | 7.36 | 7.33 | 7.36 | 7.48 | 7.09 |
| ILMN_1751020 | PACSIN1 | 0.32 | -0.04 | 0.69 | 2.84 | 7.14 | 7.11 | 7.14 | 7.08 | 6.91 |
| ILMN_2414848 | TBRG4 | 0.33 | -2.58 | -2.55 | -3.20 | 9.05 | 9.02 | 9.29 | 9.28 | 9.34 |
| ILMN_1727134 | KLHDC5 | 0.33 | -2.01 | -1.27 | -4.92 | 8.51 | 8.46 | 8.80 | 8.69 | 9.21 |
| ILMN_1741459 | CDK10 | 0.33 | -1.54 | 0.10 | 3.39 | 9.31 | 9.25 | 9.57 | 9.29 | 8.73 |
| ILMN_1689953 | CD81 | 0.33 | 0.30 | 0.80 | -3.49 | 12.67 | 12.63 | 12.63 | 12.57 | 13.09 |
| ILMN_1784218 | DDX23 | 0.33 | -2.69 | 0.09 | -4.92 | 9.09 | 9.05 | 9.38 | 9.08 | 9.63 |
| ILMN_1768050 | SCOC | 0.33 | 1.23 | 0.50 | 3.95 | 9.03 | 8.99 | 8.90 | 8.98 | 8.63 |
| ILMN_1746368 | SELT | 0.33 | 1.47 | 0.70 | -3.44 | 8.21 | 8.18 | 8.06 | 8.14 | 8.55 |
| ILMN_2149226 | CAV1 | 0.33 | 0.76 | 0.49 | -4.77 | 6.78 | 6.75 | 6.70 | 6.73 | 7.31 |
| ILMN_2180866 | RPS26P11 | 0.34 | 0.22 | 0.00 | -3.60 | 12.11 | 12.07 | 12.08 | 12.11 | 12.54 |
| ILMN_1653793 | PDPK1 | 0.34 | 1.99 | 1.07 | 2.83 | 7.53 | 7.49 | 7.30 | 7.41 | 7.20 |
| ILMN_1718830 | NARG2 | 0.34 | 1.31 | 1.21 | 6.65 | 8.00 | 7.97 | 7.91 | 7.91 | 7.54 |
| ILMN_2193706 | HRK | 0.34 | -1.53 | -1.89 | 2.76 | 9.30 | 9.23 | 9.60 | 9.67 | 8.76 |
| ILMN_1794677 | TMC6 | 0.34 | -1.42 | -1.40 | -3.53 | 8.10 | 8.05 | 8.29 | 8.29 | 8.58 |
| ILMN_1776076 | POFUT1 | 0.35 | -1.08 | -0.46 | -3.31 | 8.06 | 8.03 | 8.16 | 8.10 | 8.35 |
| ILMN_2223130 | SMARCA5 | 0.35 | 1.14 | -0.45 | 3.06 | 10.09 | 10.03 | 9.90 | 10.16 | 9.59 |
| ILMN_1683969 | FKBP1A | 0.35 | -0.70 | -1.42 | -3.48 | 10.18 | 10.14 | 10.26 | 10.34 | 10.58 |
| ILMN_3298829 | LOC729505 | 0.35 | -0.78 | -0.04 | 4.44 | 8.64 | 8.61 | 8.71 | 8.65 | 8.26 |
| ILMN_1763207 | BATF3 | 0.35 | -0.25 | -0.51 | -2.99 | 6.65 | 6.63 | 6.67 | 6.68 | 6.85 |
| ILMN_1743145 | ERAP2 | 0.36 | 3.64 | 1.36 | 4.06 | 8.56 | 8.49 | 7.85 | 8.30 | 7.77 |
| ILMN_1808765 | ZNF25 | 0.36 | -1.02 | -0.36 | -2.78 | 7.07 | 7.02 | 7.20 | 7.11 | 7.43 |
| ILMN_1765606 | YAF2 | 0.36 | -0.15 | 0.27 | -4.37 | 7.25 | 7.21 | 7.27 | 7.22 | 7.74 |
| ILMN_1803745 | SUOX | 0.36 | -1.18 | 1.03 | -3.60 | 6.72 | 6.70 | 6.80 | 6.65 | 6.95 |
| ILMN_1758173 | TMEM99 | 0.36 | 3.68 | 3.79 | 3.02 | 8.85 | 8.80 | 8.28 | 8.26 | 8.38 |
| ILMN_1742569 | PAPD1 | 0.36 | 2.58 | 1.36 | 4.58 | 8.83 | 8.81 | 8.64 | 8.73 | 8.50 |
| ILMN_2382354 | SENP7 | 0.36 | -0.20 | 2.06 | 4.96 | 9.00 | 8.97 | 9.01 | 8.83 | 8.59 |
| ILMN_1813975 | ADI1 | 0.36 | 2.59 | 1.82 | 3.31 | 8.17 | 8.11 | 7.79 | 7.90 | 7.68 |
| ILMN_1903568 |  | 0.36 | -0.82 | -0.48 | -4.12 | 9.53 | 9.47 | 9.65 | 9.60 | 10.13 |
| ILMN_1736555 | ZNF280D | 0.37 | -0.73 | 0.08 | 3.86 | 7.93 | 7.90 | 7.99 | 7.92 | 7.61 |
| ILMN_1751851 | CECR1 | 0.37 | 0.35 | -0.13 | 4.95 | 8.87 | 8.80 | 8.80 | 8.89 | 7.96 |
| ILMN_1659411 | POLR2C | 0.37 | 1.05 | 0.05 | 3.30 | 9.56 | 9.49 | 9.36 | 9.55 | 8.93 |
| ILMN_2128489 | UQCRB | 0.37 | 1.07 | 1.52 | 2.75 | 7.23 | 7.20 | 7.14 | 7.11 | 7.01 |
| ILMN_1810577 | RPS4X | 0.37 | 3.03 | 2.51 | 2.88 | 13.23 | 13.18 | 12.82 | 12.89 | 12.84 |
| ILMN_1717234 | CAST | 0.37 | 2.76 | 0.58 | 4.20 | 7.35 | 7.32 | 7.12 | 7.30 | 7.01 |
| ILMN_2390853 | CTSH | 0.37 | 0.54 | 0.47 | -2.81 | 10.04 | 9.97 | 9.94 | 9.96 | 10.54 |
| ILMN_1677200 | CYFIP2 | 0.37 | 0.20 | 0.10 | 3.06 | 11.69 | 11.64 | 11.67 | 11.68 | 11.28 |
| ILMN_1738657 | SATB2 | 0.37 | -1.76 | -0.13 | 3.50 | 9.76 | 9.70 | 10.02 | 9.78 | 9.23 |
| ILMN_1791647 | ASIP | 0.37 | 1.17 | 2.25 | 3.54 | 6.82 | 6.79 | 6.72 | 6.64 | 6.53 |
| ILMN_1764230 | GNPTG | 0.37 | -0.03 | 1.34 | -2.97 | 7.70 | 7.65 | 7.70 | 7.53 | 8.07 |
| ILMN_1771862 | TXNDC11 | 0.38 | 3.20 | 2.71 | 8.21 | 9.92 | 9.89 | 9.64 | 9.68 | 9.20 |
| ILMN_1726222 | FLOT2 | 0.38 | 0.59 | 0.52 | 3.75 | 10.65 | 10.60 | 10.57 | 10.58 | 10.12 |
| ILMN_2326737 | PPIE | 0.38 | -0.16 | -0.09 | -2.93 | 8.08 | 8.04 | 8.09 | 8.09 | 8.39 |
| ILMN_1812067 | RER1 | 0.38 | 0.15 | 0.93 | -3.71 | 7.77 | 7.72 | 7.75 | 7.66 | 8.19 |
| ILMN_1697694 | ATP6AP1 | 0.38 | -1.67 | -1.28 | -3.61 | 9.95 | 9.91 | 10.11 | 10.07 | 10.30 |
| ILMN_1762275 | CSE1L | 0.38 | -1.51 | -0.19 | -6.27 | 7.82 | 7.77 | 8.00 | 7.84 | 8.58 |
| ILMN_3244963 | WDR42A | 0.38 | -0.59 | 1.06 | -3.43 | 9.36 | 9.31 | 9.42 | 9.24 | 9.74 |
| ILMN_2359453 | ERGIC3 | 0.38 | -0.24 | 1.07 | -3.05 | 10.44 | 10.40 | 10.47 | 10.32 | 10.78 |
| ILMN_3241218 | ANKIB1 | 0.39 | -0.45 | 0.42 | -2.85 | 8.25 | 8.21 | 8.30 | 8.21 | 8.57 |
| ILMN_1757210 | CALML4 | 0.39 | 1.11 | 2.00 | 2.97 | 7.57 | 7.51 | 7.41 | 7.29 | 7.16 |
| ILMN_1678612 | ANXA6 | 0.39 | 0.47 | 0.09 | -3.02 | 6.96 | 6.93 | 6.92 | 6.95 | 7.19 |
| ILMN_1758250 | TRAFD1 | 0.39 | -0.21 | -0.30 | -3.58 | 8.09 | 8.05 | 8.12 | 8.13 | 8.49 |
| ILMN_1651347 | SERTAD2 | 0.39 | 3.81 | 1.82 | 4.46 | 10.56 | 10.51 | 10.08 | 10.33 | 9.99 |
| ILMN_1748427 | ZNF239 | 0.39 | -0.04 | 1.49 | 5.56 | 7.65 | 7.62 | 7.65 | 7.52 | 7.18 |
| ILMN_1768110 | ZAK | 0.39 | 1.01 | 0.54 | 3.90 | 8.72 | 8.62 | 8.45 | 8.58 | 7.69 |
| ILMN_1776656 | BBS7 | 0.39 | 1.34 | 0.20 | 3.11 | 7.32 | 7.29 | 7.21 | 7.31 | 7.06 |
| ILMN_1759818 | SORL1 | 0.39 | 0.00 | -0.11 | -4.16 | 6.84 | 6.80 | 6.84 | 6.85 | 7.23 |
| ILMN_2170949 | SNX10 | 0.40 | 0.58 | -1.43 | 5.03 | 9.49 | 9.45 | 9.43 | 9.65 | 8.94 |
| ILMN_1703720 | SF3B14 | 0.40 | 2.29 | 3.54 | 3.36 | 11.58 | 11.54 | 11.33 | 11.20 | 11.22 |
| ILMN_1661631 | LILRA3 | 0.40 | 0.28 | -2.46 | -2.82 | 6.70 | 6.67 | 6.68 | 6.91 | 6.94 |
| ILMN_1665192 | NUDT6 | 0.40 | 1.57 | 1.13 | 2.84 | 8.01 | 7.97 | 7.86 | 7.90 | 7.75 |
| ILMN_1758658 | FADD | 0.40 | 0.67 | -0.04 | 3.21 | 9.35 | 9.32 | 9.30 | 9.35 | 9.11 |
| ILMN_1704418 | FOXD1 | 0.40 | -0.54 | -2.37 | 3.54 | 7.91 | 7.88 | 7.95 | 8.09 | 7.65 |
| ILMN_1755741 | DACH1 | 0.40 | 0.36 | 0.04 | 3.36 | 6.86 | 6.83 | 6.83 | 6.85 | 6.61 |
| ILMN_2408987 | SMARCD3 | 0.40 | -1.01 | -1.58 | -4.56 | 6.65 | 6.62 | 6.74 | 6.78 | 7.03 |
| ILMN_2402674 | DPM3 | 0.41 | -0.99 | -0.10 | -3.24 | 8.54 | 8.47 | 8.70 | 8.56 | 9.07 |
| ILMN_1671731 | AVPI1 | 0.41 | 0.49 | 0.20 | -3.28 | 7.55 | 7.50 | 7.49 | 7.52 | 7.98 |
| ILMN_1697597 | KIAA0494 | 0.41 | -1.00 | -1.17 | -3.05 | 8.03 | 7.98 | 8.14 | 8.16 | 8.37 |
| ILMN_2156953 | ZFAND6 | 0.41 | 1.62 | 0.14 | 3.38 | 8.57 | 8.52 | 8.37 | 8.55 | 8.16 |
| ILMN_1815033 | HPCA | 0.41 | 0.60 | -1.42 | -2.98 | 6.69 | 6.66 | 6.65 | 6.79 | 6.91 |
| ILMN_1717765 | NUDT11 | 0.42 | -0.93 | -1.02 | 3.12 | 8.72 | 8.66 | 8.87 | 8.89 | 8.22 |
| ILMN_1696962 | PDZD8 | 0.42 | 1.18 | 1.12 | 2.97 | 7.66 | 7.62 | 7.54 | 7.55 | 7.35 |
| ILMN_1704261 | RANGRF | 0.42 | 1.88 | 1.84 | 3.13 | 7.74 | 7.70 | 7.56 | 7.57 | 7.44 |
| ILMN_2371964 | MRPS12 | 0.42 | 0.17 | -0.28 | -2.96 | 11.52 | 11.47 | 11.50 | 11.56 | 11.88 |
| ILMN_1674250 | NCKAP1L | 0.42 | -0.17 | -1.16 | -4.97 | 9.38 | 9.32 | 9.40 | 9.54 | 10.05 |
| ILMN_1799744 | GALC | 0.42 | -0.34 | 0.16 | -4.88 | 7.97 | 7.92 | 8.01 | 7.95 | 8.59 |
| ILMN_1742548 | GFI1B | 0.42 | 0.32 | -1.00 | -4.81 | 6.71 | 6.68 | 6.69 | 6.77 | 7.01 |
| ILMN_2361807 | OS9 | 0.42 | -0.75 | -0.16 | -3.48 | 7.57 | 7.53 | 7.65 | 7.59 | 7.93 |
| ILMN_1685378 | LOC728973 | 0.42 | 1.05 | 0.95 | 3.09 | 13.00 | 12.94 | 12.86 | 12.87 | 12.59 |
| ILMN_1700733 | KIAA1370 | 0.42 | 0.24 | 0.61 | 2.76 | 9.43 | 9.38 | 9.40 | 9.36 | 9.12 |
| ILMN_1705602 | KLHL17 | 0.43 | -1.58 | 0.13 | -2.86 | 7.56 | 7.51 | 7.77 | 7.55 | 7.94 |
| ILMN_1793386 | MED12 | 0.43 | -0.37 | -1.14 | 2.76 | 8.16 | 8.11 | 8.21 | 8.30 | 7.84 |
| ILMN_1664978 | TJP2 | 0.43 | -1.54 | -1.51 | -4.14 | 6.85 | 6.81 | 7.01 | 7.00 | 7.27 |
| ILMN_1766085 | REL | 0.43 | 2.48 | 1.69 | 4.62 | 7.78 | 7.71 | 7.39 | 7.51 | 7.06 |
| ILMN_3244125 | LOC100132810 | 0.43 | -1.21 | -0.13 | 3.11 | 8.15 | 8.08 | 8.37 | 8.18 | 7.60 |
| ILMN_1722838 | MRPL46 | 0.43 | 1.24 | 1.70 | 3.38 | 10.09 | 10.04 | 9.95 | 9.90 | 9.70 |
| ILMN_1665571 | LOC644869 | 0.43 | 1.94 | 1.33 | 3.32 | 8.01 | 7.95 | 7.75 | 7.83 | 7.57 |
| ILMN_1752340 | ARF5 | 0.44 | -1.56 | -0.75 | -2.85 | 9.31 | 9.24 | 9.57 | 9.44 | 9.79 |
| ILMN_1779410 | VAPA | 0.44 | -2.01 | -1.64 | -3.05 | 7.04 | 7.00 | 7.24 | 7.21 | 7.35 |
| ILMN_1694466 | ZBED1 | 0.44 | -0.16 | 0.11 | 3.77 | 9.67 | 9.62 | 9.69 | 9.66 | 9.21 |
| ILMN_1811029 | TLK1 | 0.44 | 2.03 | 2.11 | 3.62 | 9.44 | 9.38 | 9.19 | 9.18 | 9.00 |
| ILMN_1694847 | TERF1 | 0.44 | -1.36 | 0.22 | 3.90 | 8.13 | 8.10 | 8.24 | 8.12 | 7.84 |
| ILMN_1713744 | C14orf132 | 0.44 | 0.71 | 1.59 | 4.34 | 6.92 | 6.89 | 6.88 | 6.82 | 6.66 |
| ILMN_1698996 | SLC19A1 | 0.44 | -1.14 | 0.96 | -4.10 | 7.32 | 7.26 | 7.46 | 7.19 | 7.84 |
| ILMN_1697529 | RNF10 | 0.44 | -1.14 | -0.76 | -2.80 | 7.39 | 7.35 | 7.51 | 7.47 | 7.69 |
| ILMN_3203444 | LOC100132535 | 0.44 | 0.93 | 0.19 | -3.19 | 6.85 | 6.81 | 6.77 | 6.84 | 7.14 |
| ILMN_3258424 | LOC100129118 | 0.45 | 2.14 | 1.40 | 3.38 | 9.38 | 9.34 | 9.16 | 9.24 | 9.03 |
| ILMN_1815148 | MAN2A2 | 0.45 | -1.79 | -2.37 | -3.53 | 7.05 | 7.02 | 7.19 | 7.23 | 7.32 |
| ILMN_1687440 | HIPK2 | 0.45 | 0.25 | 1.59 | -5.40 | 7.41 | 7.31 | 7.35 | 7.04 | 8.65 |
| ILMN_1704404 | PSMD13 | 0.45 | -0.42 | -1.89 | -4.33 | 8.39 | 8.35 | 8.44 | 8.59 | 8.84 |
| ILMN_2100458 | RFESD | 0.45 | 4.84 | 5.02 | 3.75 | 7.61 | 7.58 | 7.26 | 7.24 | 7.34 |
| ILMN_1703500 | KCNQ2 | 0.46 | -0.05 | -1.46 | -2.74 | 6.74 | 6.71 | 6.75 | 6.85 | 6.94 |
| ILMN_1745374 | IFI35 | 0.46 | 1.49 | 1.08 | 4.34 | 8.93 | 8.84 | 8.63 | 8.71 | 8.05 |
| ILMN_1790555 | CCDC146 | 0.46 | 1.38 | 3.29 | 3.14 | 7.03 | 6.99 | 6.91 | 6.74 | 6.75 |
| ILMN_2124802 | MT1H | 0.46 | 0.72 | 5.02 | 4.71 | 9.48 | 9.30 | 9.19 | 7.48 | 7.60 |
| ILMN_1792138 | UQCRH | 0.46 | 1.10 | 2.49 | 3.39 | 10.48 | 10.43 | 10.35 | 10.19 | 10.09 |
| ILMN_1699545 | PCSK7 | 0.46 | -1.96 | -0.43 | -4.14 | 7.91 | 7.87 | 8.09 | 7.95 | 8.30 |
| ILMN_1789599 | NBL1 | 0.46 | 0.27 | 0.17 | 5.68 | 8.20 | 8.13 | 8.16 | 8.17 | 7.30 |
| ILMN_1687840 | ABCB7 | 0.46 | 2.04 | 0.37 | 3.65 | 8.84 | 8.79 | 8.62 | 8.80 | 8.45 |
| ILMN_1675359 | NKAP | 0.47 | 2.07 | 0.85 | 3.91 | 8.01 | 7.95 | 7.74 | 7.90 | 7.51 |
| ILMN_1812479 | ATE1 | 0.47 | 1.24 | 2.33 | 2.80 | 7.16 | 7.13 | 7.08 | 7.01 | 6.98 |
| ILMN_1909073 |  | 0.47 | -0.47 | -0.30 | -3.17 | 7.53 | 7.49 | 7.58 | 7.56 | 7.85 |
| ILMN_1657697 | SAR1A | 0.47 | 0.83 | -0.51 | 3.54 | 9.79 | 9.74 | 9.71 | 9.83 | 9.46 |
| ILMN_1809364 | NTF3 | 0.47 | -0.52 | -1.34 | -4.39 | 7.71 | 7.62 | 7.81 | 7.97 | 8.55 |
| ILMN_1713505 | NPC1 | 0.47 | 0.78 | -0.56 | -3.33 | 8.25 | 8.20 | 8.16 | 8.31 | 8.60 |
| ILMN_1671509 | CCL3 | 0.47 | -0.51 | -0.69 | -2.87 | 6.78 | 6.74 | 6.83 | 6.85 | 7.05 |
| ILMN_2381841 | DDX19B | 0.47 | 0.28 | 1.11 | 7.47 | 9.84 | 9.77 | 9.80 | 9.68 | 8.79 |
| ILMN_1727194 | CALU | 0.47 | 0.18 | 0.55 | -4.64 | 8.04 | 7.98 | 8.01 | 7.97 | 8.62 |
| ILMN_2057220 | HRSP12 | 0.48 | 2.46 | 3.20 | 3.78 | 7.71 | 7.65 | 7.40 | 7.31 | 7.23 |
| ILMN_1791534 | LOC731682 | 0.48 | 2.54 | 0.83 | 4.78 | 10.13 | 10.05 | 9.71 | 9.99 | 9.33 |
| ILMN_2342068 | ERC1 | 0.48 | 0.71 | 0.51 | 3.04 | 7.47 | 7.41 | 7.39 | 7.41 | 7.13 |
| ILMN_1812721 | LOC728014 | 0.48 | -2.69 | -3.60 | -9.38 | 9.86 | 9.83 | 10.07 | 10.14 | 10.58 |
| ILMN_1731137 | TXNDC9 | 0.48 | 2.07 | 1.71 | 4.74 | 9.79 | 9.75 | 9.62 | 9.65 | 9.39 |
| ILMN_1655225 | C19orf25 | 0.48 | -1.96 | -1.57 | -3.05 | 7.37 | 7.34 | 7.52 | 7.49 | 7.61 |
| ILMN_1679880 | THOC6 | 0.48 | -1.31 | -0.83 | -3.28 | 8.74 | 8.69 | 8.86 | 8.82 | 9.05 |
| ILMN_1672080 | NR2F6 | 0.48 | -1.37 | -2.11 | -3.59 | 7.52 | 7.47 | 7.68 | 7.77 | 7.94 |
| ILMN_2364852 | BTN2A1 | 0.48 | 2.90 | 3.31 | 4.24 | 8.73 | 8.69 | 8.47 | 8.43 | 8.35 |
| ILMN_2075927 | STK40 | 0.48 | 1.31 | -0.19 | 4.60 | 9.41 | 9.38 | 9.32 | 9.42 | 9.09 |
| ILMN_1777564 | MAD2L1 | 0.48 | 2.09 | 0.88 | 4.96 | 11.05 | 11.00 | 10.82 | 10.95 | 10.50 |
| ILMN_1784641 | NDUFA3 | 0.48 | -0.88 | -0.88 | -2.78 | 10.66 | 10.63 | 10.73 | 10.73 | 10.87 |
| ILMN_2090786 | GCSH | 0.49 | 0.29 | 1.12 | 4.00 | 7.02 | 6.99 | 7.00 | 6.95 | 6.75 |
| ILMN_1766974 | ZNF107 | 0.49 | 1.61 | 1.14 | 4.26 | 8.06 | 8.00 | 7.85 | 7.91 | 7.51 |
| ILMN_1723625 | MAP4K2 | 0.49 | -1.74 | 0.07 | -4.54 | 11.27 | 11.22 | 11.45 | 11.26 | 11.73 |
| ILMN_2082810 | BRD7 | 0.49 | -1.48 | 1.45 | 7.99 | 10.31 | 10.26 | 10.44 | 10.18 | 9.61 |
| ILMN_1657837 | ZC3H8 | 0.50 | 3.29 | 4.61 | 0.11 | 8.99 | 8.93 | 8.60 | 8.44 | 8.98 |
| ILMN_1726591 | CARD16 | 0.50 | 1.84 | 2.83 | 2.80 | 7.30 | 7.24 | 7.10 | 6.99 | 7.00 |
| ILMN_1656718 | DEF8 | 0.50 | 1.73 | 0.21 | 3.61 | 8.18 | 8.09 | 7.86 | 8.14 | 7.50 |
| ILMN_2142284 | SLC25A43 | 0.50 | 0.90 | -0.18 | 3.80 | 8.96 | 8.86 | 8.78 | 9.00 | 8.18 |
| ILMN_1820572 |  | 0.50 | 1.32 | 0.32 | 5.38 | 7.77 | 7.74 | 7.68 | 7.75 | 7.38 |
| ILMN_2343624 | METTL13 | 0.50 | 0.71 | 1.77 | -3.74 | 9.99 | 9.95 | 9.94 | 9.85 | 10.29 |
| ILMN_1785095 | ATP6V0E2 | 0.50 | -2.90 | -1.74 | -2.90 | 9.93 | 9.86 | 10.31 | 10.16 | 10.31 |
| ILMN_1798006 | ANKRD35 | 0.51 | -1.49 | -2.08 | -4.29 | 6.74 | 6.68 | 6.90 | 6.97 | 7.22 |
| ILMN_1678037 | HIRIP3 | 0.51 | 0.66 | 1.78 | 3.86 | 10.18 | 10.12 | 10.10 | 9.98 | 9.75 |
| ILMN_1733603 | NDUFC1 | 0.51 | 0.57 | -1.66 | 3.20 | 9.32 | 9.26 | 9.26 | 9.50 | 8.97 |
| ILMN_1654685 | MCTP1 | 0.51 | 1.67 | 0.71 | 3.83 | 7.63 | 7.53 | 7.31 | 7.49 | 6.91 |
| ILMN_1660199 | ACAA2 | 0.52 | 2.67 | 3.16 | 3.76 | 9.71 | 9.65 | 9.40 | 9.34 | 9.27 |
| ILMN_2363586 | SDCBP | 0.52 | 0.96 | 1.79 | 3.72 | 7.92 | 7.86 | 7.80 | 7.69 | 7.45 |
| ILMN_2205211 | LOC134997 | 0.52 | 1.97 | 0.95 | 3.02 | 13.42 | 13.34 | 13.13 | 13.28 | 12.98 |
| ILMN_1798163 | UPF3B | 0.52 | 3.47 | 3.21 | 4.87 | 9.87 | 9.82 | 9.52 | 9.55 | 9.38 |
| ILMN_1703288 | C19orf6 | 0.52 | -1.35 | -1.35 | -2.76 | 9.09 | 9.01 | 9.32 | 9.32 | 9.55 |
| ILMN_2179778 | PHLDB2 | 0.53 | 0.09 | 0.61 | 3.81 | 6.79 | 6.75 | 6.79 | 6.75 | 6.50 |
| ILMN_3235148 | FLJ43681 | 0.53 | 3.15 | 2.84 | 3.72 | 12.60 | 12.52 | 12.09 | 12.14 | 11.99 |
| ILMN_2334303 | SEC24B | 0.53 | 1.09 | 0.00 | 5.71 | 9.06 | 9.01 | 8.95 | 9.06 | 8.52 |
| ILMN_2259119 | PRMT2 | 0.53 | 1.86 | 1.80 | 3.02 | 6.86 | 6.82 | 6.74 | 6.74 | 6.67 |
| ILMN_1720849 | PYGM | 0.53 | -1.50 | 0.10 | -4.44 | 6.79 | 6.75 | 6.89 | 6.78 | 7.10 |
| ILMN_1779399 | SNRPA | 0.54 | -1.33 | -0.48 | -2.82 | 9.27 | 9.21 | 9.41 | 9.32 | 9.56 |
| ILMN_2182120 | SF3B14 | 0.54 | 2.56 | 2.19 | 3.01 | 10.41 | 10.34 | 10.11 | 10.15 | 10.05 |
| ILMN_1694068 | EFCAB2 | 0.54 | 1.32 | 1.82 | 3.14 | 6.86 | 6.81 | 6.74 | 6.70 | 6.59 |
| ILMN_1742798 | SFRS10 | 0.55 | 0.95 | 0.66 | 3.71 | 11.96 | 11.91 | 11.87 | 11.90 | 11.62 |
| ILMN_1664610 | LOC402057 | 0.56 | 2.02 | 2.17 | 2.98 | 14.19 | 14.16 | 14.06 | 14.05 | 14.00 |
| ILMN_3236637 | PIP4K2A | 0.56 | 1.09 | 1.15 | 2.80 | 9.25 | 9.15 | 9.05 | 9.04 | 8.74 |
| ILMN_1702835 | SH3BGRL | 0.56 | 1.60 | -2.49 | 3.08 | 10.93 | 10.88 | 10.78 | 11.15 | 10.65 |
| ILMN_1807136 | LOC729559 | 0.56 | -1.35 | 1.29 | -2.89 | 9.33 | 9.26 | 9.49 | 9.17 | 9.67 |
| ILMN_2212999 | KIF5C | 0.56 | -0.39 | 0.18 | -3.48 | 6.76 | 6.71 | 6.80 | 6.75 | 7.09 |
| ILMN_2154455 | ZNF330 | 0.56 | 1.39 | 0.83 | 5.51 | 9.35 | 9.30 | 9.21 | 9.27 | 8.80 |
| ILMN_1756402 | TMEM177 | 0.57 | -0.54 | 0.25 | -3.26 | 8.66 | 8.57 | 8.74 | 8.62 | 9.14 |
| ILMN_3251383 | CCDC74B | 0.57 | -0.86 | -0.52 | -3.05 | 7.70 | 7.62 | 7.83 | 7.78 | 8.15 |
| ILMN_1774272 | ESRRA | 0.57 | 0.15 | 0.80 | -3.84 | 8.96 | 8.91 | 8.94 | 8.89 | 9.29 |
| ILMN_1704730 | CD93 | 0.57 | -1.02 | -0.87 | -6.29 | 6.92 | 6.83 | 7.08 | 7.06 | 7.91 |
| ILMN_1771376 | PEA15 | 0.57 | 1.66 | 0.42 | -2.93 | 9.24 | 9.17 | 9.05 | 9.19 | 9.56 |
| ILMN_1720526 | CENPN | 0.58 | 1.88 | 1.98 | 6.21 | 9.99 | 9.91 | 9.74 | 9.73 | 9.16 |
| ILMN_2098013 | CETP | 0.58 | -0.62 | 0.92 | -2.89 | 7.16 | 7.11 | 7.21 | 7.08 | 7.41 |
| ILMN_2053281 | C14orf149 | 0.58 | 2.62 | 3.02 | 3.05 | 7.23 | 7.18 | 6.99 | 6.95 | 6.95 |
| ILMN_1667626 | EGLN3 | 0.58 | 1.63 | 0.93 | 5.55 | 7.51 | 7.46 | 7.37 | 7.43 | 7.03 |
| ILMN_2405009 | NBL1 | 0.59 | 0.08 | -0.31 | 5.54 | 7.85 | 7.78 | 7.84 | 7.89 | 7.19 |
| ILMN_1717485 | LOC285556 | 0.59 | 0.20 | 1.44 | 2.87 | 7.30 | 7.20 | 7.27 | 7.06 | 6.83 |
| ILMN_2334296 | IL18BP | 0.59 | 1.85 | 1.98 | 3.34 | 7.44 | 7.37 | 7.24 | 7.22 | 7.08 |
| ILMN_1789558 | FAM164A | 0.59 | 1.18 | 0.31 | 3.13 | 7.04 | 6.99 | 6.94 | 7.02 | 6.78 |
| ILMN_3242586 | RHOU | 0.59 | -0.03 | 0.68 | 5.76 | 7.63 | 7.57 | 7.63 | 7.56 | 7.01 |
| ILMN_1782419 | GNG11 | 0.59 | 1.30 | 1.20 | 5.55 | 10.11 | 9.89 | 9.62 | 9.66 | 8.02 |
| ILMN_1748488 | TXNL4B | 0.59 | -0.21 | 0.83 | 3.13 | 7.38 | 7.32 | 7.40 | 7.30 | 7.08 |
| ILMN_3246388 | MED14 | 0.59 | -1.54 | -1.86 | -3.34 | 8.79 | 8.72 | 8.96 | 9.00 | 9.16 |
| ILMN_2162234 | NEK1 | 0.59 | 0.24 | 0.00 | 3.23 | 8.57 | 8.50 | 8.54 | 8.57 | 8.20 |
| ILMN_1758049 | NFIA | 0.59 | 1.02 | 0.39 | 2.79 | 7.99 | 7.88 | 7.80 | 7.92 | 7.49 |
| ILMN_1677532 | TARDBP | 0.60 | -1.09 | 0.43 | -2.81 | 8.52 | 8.43 | 8.69 | 8.46 | 8.96 |
| ILMN_2346769 | FAM189B | 0.60 | -0.27 | -1.86 | -4.21 | 7.04 | 7.00 | 7.06 | 7.15 | 7.30 |
| ILMN_3248301 | ZNF583 | 0.60 | 1.17 | 1.84 | 3.08 | 7.37 | 7.31 | 7.26 | 7.20 | 7.08 |
| ILMN_1722945 | C6orf52 | 0.60 | 3.18 | 3.16 | 4.15 | 7.97 | 7.89 | 7.53 | 7.54 | 7.40 |
| ILMN_2207363 | RABAC1 | 0.61 | -1.82 | -1.70 | -3.48 | 9.37 | 9.30 | 9.57 | 9.56 | 9.75 |
| ILMN_1876729 |  | 0.61 | 1.08 | 1.95 | 3.50 | 7.47 | 7.41 | 7.37 | 7.28 | 7.13 |
| ILMN_1811650 | DUS2L | 0.61 | 0.28 | 0.26 | 3.08 | 8.46 | 8.37 | 8.42 | 8.42 | 8.02 |
| ILMN_3245413 | DENND5A | 0.61 | 1.37 | 0.99 | 3.96 | 8.63 | 8.58 | 8.51 | 8.54 | 8.28 |
| ILMN_1724009 | SETD6 | 0.61 | -1.61 | 2.19 | 2.83 | 8.15 | 8.09 | 8.30 | 7.95 | 7.89 |
| ILMN_2126957 | NOMO1 | 0.61 | -1.17 | 0.86 | -6.40 | 8.15 | 8.11 | 8.23 | 8.10 | 8.57 |
| ILMN_1744963 | ERO1L | 0.61 | 1.59 | 0.99 | 2.81 | 7.55 | 7.51 | 7.44 | 7.48 | 7.35 |
| ILMN_1732080 | SUMO1P3 | 0.61 | 2.10 | 2.39 | 3.26 | 9.93 | 9.86 | 9.69 | 9.66 | 9.56 |
| ILMN_2041101 | ANXA2P1 | 0.61 | 1.92 | -0.79 | 3.85 | 10.94 | 10.82 | 10.57 | 11.09 | 10.21 |
| ILMN_1807211 | NICN1 | 0.61 | -1.84 | 0.52 | 3.59 | 8.55 | 8.50 | 8.68 | 8.51 | 8.29 |
| ILMN_2112599 | C16orf80 | 0.61 | -0.38 | 1.23 | 6.94 | 9.73 | 9.69 | 9.76 | 9.64 | 9.24 |
| ILMN_1742813 | TMEM167A | 0.62 | 2.32 | 1.47 | 3.79 | 8.83 | 8.78 | 8.63 | 8.70 | 8.50 |
| ILMN_1686477 | CNOT7 | 0.62 | 0.00 | 0.73 | 3.22 | 7.01 | 6.97 | 7.01 | 6.96 | 6.80 |
| ILMN_1729980 | RNF216 | 0.62 | -2.40 | -4.15 | -2.34 | 8.21 | 8.14 | 8.46 | 8.65 | 8.46 |
| ILMN_2073604 | EBP | 0.62 | 0.29 | -1.01 | -3.87 | 8.73 | 8.65 | 8.69 | 8.86 | 9.23 |
| ILMN_1672022 | EPHA4 | 0.63 | -1.16 | -0.43 | -4.87 | 6.67 | 6.63 | 6.75 | 6.70 | 7.01 |
| ILMN_2388484 | MAP2 | 0.63 | 2.24 | 2.87 | 3.37 | 6.87 | 6.82 | 6.70 | 6.65 | 6.62 |
| ILMN_1802316 | SMPD3 | 0.63 | 2.32 | 0.73 | 2.89 | 7.17 | 7.10 | 6.93 | 7.09 | 6.88 |
| ILMN_1803357 | FLJ40125 | 0.63 | 0.00 | 0.42 | -2.84 | 6.82 | 6.78 | 6.82 | 6.80 | 7.00 |
| ILMN_2079655 | KLRB1 | 0.63 | 1.81 | 2.60 | 4.95 | 7.78 | 7.66 | 7.44 | 7.30 | 6.86 |
| ILMN_2125747 | LOC606724 | 0.63 | -0.90 | -1.27 | -3.36 | 10.43 | 10.35 | 10.56 | 10.61 | 10.89 |
| ILMN_1798472 | LOC550112 | 0.64 | 0.98 | 1.83 | 2.76 | 7.00 | 6.95 | 6.92 | 6.86 | 6.78 |
| ILMN_2226304 | ANKRD50 | 0.64 | 1.35 | -1.19 | 3.56 | 7.41 | 7.34 | 7.27 | 7.53 | 7.04 |
| ILMN_1732725 | SAPS3 | 0.65 | -1.47 | 0.68 | -3.01 | 9.18 | 9.11 | 9.34 | 9.11 | 9.51 |
| ILMN_1812701 | C4orf33 | 0.65 | 2.53 | 1.01 | 7.73 | 8.48 | 8.40 | 8.18 | 8.36 | 7.56 |
| ILMN_1739985 | TAGAP | 0.65 | 0.16 | 0.08 | 4.20 | 7.53 | 7.48 | 7.52 | 7.52 | 7.18 |
| ILMN_2229922 | C12orf35 | 0.65 | 2.24 | 1.41 | 4.03 | 10.33 | 10.25 | 10.07 | 10.17 | 9.87 |
| ILMN_1680037 | FAM65A | 0.65 | -0.45 | 0.28 | 6.90 | 10.17 | 10.09 | 10.22 | 10.14 | 9.35 |
| ILMN_1669273 | PPT1 | 0.65 | 0.56 | 0.43 | -2.81 | 10.77 | 10.70 | 10.71 | 10.72 | 11.07 |
| ILMN_2162328 | PTS | 0.65 | 1.20 | 1.78 | 4.48 | 10.96 | 10.89 | 10.84 | 10.78 | 10.50 |
| ILMN_1704621 | WDR44 | 0.65 | 2.18 | 0.80 | 3.68 | 7.36 | 7.30 | 7.17 | 7.29 | 7.04 |
| ILMN_1671132 | LOC646512 | 0.65 | 1.26 | 2.35 | 3.63 | 7.43 | 7.34 | 7.25 | 7.10 | 6.91 |
| ILMN_1798804 | SRPK1 | 0.65 | 0.55 | 1.41 | -2.82 | 10.30 | 10.23 | 10.24 | 10.16 | 10.57 |
| ILMN_1738718 | CYB561D2 | 0.65 | 0.05 | 0.98 | -2.92 | 7.89 | 7.80 | 7.88 | 7.75 | 8.31 |
| ILMN_1761131 | PECI | 0.66 | 3.23 | 3.06 | 5.43 | 9.67 | 9.58 | 9.24 | 9.27 | 8.95 |
| ILMN_1808405 | HLA-DQA1 | 0.66 | 2.00 | 2.53 | 3.53 | 11.66 | 11.59 | 11.45 | 11.39 | 11.29 |
| ILMN_3231881 | LOC728026 | 0.66 | 1.13 | 0.47 | 3.35 | 11.79 | 11.72 | 11.67 | 11.74 | 11.43 |
| ILMN_2096191 | AASDHPPT | 0.66 | 3.33 | 2.38 | 3.85 | 10.91 | 10.85 | 10.60 | 10.69 | 10.56 |
| ILMN_1774779 | LOC647121 | 0.66 | 2.69 | 1.45 | 5.13 | 7.26 | 7.21 | 7.04 | 7.14 | 6.85 |
| ILMN_1724295 | LOC643007 | 0.66 | 0.69 | 1.27 | 4.41 | 10.63 | 10.56 | 10.55 | 10.49 | 10.14 |
| ILMN_1790211 | C7orf57 | 0.67 | 0.29 | -1.00 | -4.89 | 6.68 | 6.63 | 6.66 | 6.75 | 7.02 |
| ILMN_1657509 | TSEN54 | 0.67 | -1.06 | 0.63 | -3.72 | 7.47 | 7.41 | 7.56 | 7.41 | 7.78 |
| ILMN_1717165 | IGBP1 | 0.67 | 2.67 | 1.91 | 3.43 | 9.46 | 9.37 | 9.09 | 9.20 | 8.98 |
| ILMN_1772658 | KIAA0947 | 0.67 | 1.88 | 1.38 | 3.45 | 8.49 | 8.38 | 8.19 | 8.27 | 7.94 |
| ILMN_2243687 | LAMP2 | 0.67 | 1.81 | 2.10 | 4.79 | 7.77 | 7.72 | 7.63 | 7.60 | 7.39 |
| ILMN_2160764 | HBP1 | 0.67 | 0.67 | 2.81 | 3.49 | 7.93 | 7.88 | 7.88 | 7.71 | 7.66 |
| ILMN_1755462 | UGCGL1 | 0.67 | -0.43 | -2.21 | -3.12 | 7.16 | 7.11 | 7.20 | 7.35 | 7.43 |
| ILMN_2135232 | SFRS3 | 0.67 | -0.13 | 0.07 | 2.86 | 7.77 | 7.71 | 7.79 | 7.77 | 7.49 |
| ILMN_2094061 | IMPA2 | 0.68 | 0.41 | 1.13 | 3.50 | 9.05 | 8.95 | 8.99 | 8.88 | 8.53 |
| ILMN_1683441 | NCAPD3 | 0.68 | -2.15 | -2.27 | -3.47 | 7.58 | 7.52 | 7.76 | 7.77 | 7.87 |
| ILMN_2133146 | NUDT21 | 0.68 | -0.15 | 0.34 | 3.80 | 7.56 | 7.48 | 7.57 | 7.52 | 7.15 |
| ILMN_1728779 | NGLY1 | 0.68 | 0.83 | 2.00 | -3.29 | 9.23 | 9.17 | 9.16 | 9.06 | 9.52 |
| ILMN_2122374 | FAM49B | 0.68 | 1.73 | 1.91 | 3.30 | 9.45 | 9.35 | 9.20 | 9.17 | 8.97 |
| ILMN_2217809 | TMEM126A | 0.68 | 2.53 | 0.96 | 3.01 | 10.61 | 10.55 | 10.37 | 10.52 | 10.32 |
| ILMN_1751378 | RARS | 0.69 | 3.39 | 2.48 | 3.13 | 11.16 | 11.10 | 10.87 | 10.95 | 10.89 |
| ILMN_1773760 | PAICS | 0.69 | 0.20 | 0.59 | 3.36 | 11.99 | 11.90 | 11.96 | 11.91 | 11.55 |
| ILMN_2112493 | DAP | 0.69 | 0.89 | -1.30 | 3.34 | 8.51 | 8.42 | 8.39 | 8.68 | 8.07 |
| ILMN_1724194 | NPEPL1 | 0.69 | -1.11 | -0.39 | -4.23 | 7.59 | 7.50 | 7.74 | 7.64 | 8.16 |
| ILMN_2051232 | SDHA | 0.69 | -0.65 | -0.80 | 2.84 | 9.54 | 9.48 | 9.59 | 9.61 | 9.29 |
| ILMN_1665823 | LOC389787 | 0.69 | 1.35 | 1.09 | 2.81 | 12.80 | 12.66 | 12.54 | 12.59 | 12.25 |
| ILMN_1683026 | PSMB10 | 0.69 | 0.24 | 0.53 | 3.59 | 12.23 | 12.14 | 12.20 | 12.16 | 11.78 |
| ILMN_1716265 | PGM2L1 | 0.69 | 0.65 | 1.71 | 4.28 | 7.08 | 7.02 | 7.02 | 6.94 | 6.73 |
| ILMN_1728676 | KIAA0196 | 0.69 | 2.61 | 3.50 | 2.94 | 9.66 | 9.59 | 9.40 | 9.31 | 9.36 |
| ILMN_2056167 | OSTC | 0.69 | 1.08 | 0.66 | 3.54 | 10.63 | 10.50 | 10.43 | 10.51 | 9.98 |
| ILMN_1874735 |  | 0.70 | 0.37 | 1.76 | 2.92 | 6.84 | 6.79 | 6.82 | 6.72 | 6.63 |
| ILMN_1686194 | SDCCAG10 | 0.70 | 0.97 | 2.24 | 3.94 | 9.79 | 9.72 | 9.69 | 9.55 | 9.36 |
| ILMN_1714083 | KLHL8 | 0.70 | 1.76 | 1.07 | 3.04 | 7.79 | 7.69 | 7.54 | 7.64 | 7.35 |
| ILMN_2373763 | CASP7 | 0.70 | -1.05 | 0.70 | 3.75 | 7.17 | 7.13 | 7.24 | 7.13 | 6.92 |
| ILMN_1874689 |  | 0.70 | -3.12 | 0.92 | 3.33 | 8.04 | 7.94 | 8.48 | 7.91 | 7.57 |
| ILMN_1781680 | DAP3 | 0.70 | 0.92 | 1.11 | -2.79 | 10.50 | 10.42 | 10.39 | 10.37 | 10.85 |
| ILMN_1756311 | UFSP2 | 0.71 | 1.03 | 0.99 | 3.20 | 9.64 | 9.54 | 9.50 | 9.50 | 9.19 |
| ILMN_1689002 | DTX1 | 0.71 | -1.65 | -0.88 | -6.03 | 9.93 | 9.85 | 10.12 | 10.03 | 10.61 |
| ILMN_2226314 | DBR1 | 0.71 | -0.17 | 1.25 | 2.97 | 7.60 | 7.53 | 7.61 | 7.47 | 7.30 |
| ILMN_1882315 |  | 0.71 | -1.34 | 0.98 | 5.97 | 7.39 | 7.34 | 7.49 | 7.32 | 6.95 |
| ILMN_2367681 | SPAG1 | 0.71 | 2.27 | 1.73 | 2.94 | 8.45 | 8.34 | 8.10 | 8.18 | 8.00 |
| ILMN_1689456 | ZBTB20 | 0.71 | 1.03 | 1.69 | 4.23 | 7.79 | 7.67 | 7.61 | 7.49 | 7.04 |
| ILMN_2386790 | KLRC3 | 0.72 | 1.48 | 2.46 | 4.05 | 8.24 | 8.14 | 8.03 | 7.89 | 7.67 |
| ILMN_1768176 | CXorf26 | 0.72 | 2.64 | 1.92 | 6.30 | 10.17 | 10.08 | 9.86 | 9.94 | 9.43 |
| ILMN_1659703 | WWP2 | 0.72 | 0.84 | 2.71 | 4.18 | 8.81 | 8.75 | 8.74 | 8.59 | 8.46 |
| ILMN_1713846 | PPM1H | 0.72 | 0.23 | 1.24 | 2.97 | 7.06 | 6.98 | 7.03 | 6.93 | 6.75 |
| ILMN_1665049 | SPG11 | 0.72 | 1.24 | -0.03 | 3.26 | 9.74 | 9.65 | 9.59 | 9.74 | 9.36 |
| ILMN_2405305 | ARNTL | 0.73 | 0.14 | 0.97 | 2.90 | 7.16 | 7.09 | 7.15 | 7.07 | 6.88 |
| ILMN_2055330 | KIF26B | 0.73 | 0.61 | -0.25 | 3.16 | 7.27 | 7.16 | 7.18 | 7.31 | 6.81 |
| ILMN_1678454 | CASP4 | 0.73 | 3.33 | 3.06 | 4.41 | 10.04 | 9.93 | 9.51 | 9.55 | 9.34 |
| ILMN_1771949 | TAF4B | 0.73 | 0.70 | 0.30 | 2.97 | 8.09 | 8.01 | 8.01 | 8.06 | 7.76 |
| ILMN_1652024 | CSNK1G3 | 0.73 | 1.42 | 1.92 | 3.98 | 8.41 | 8.30 | 8.20 | 8.12 | 7.81 |
| ILMN_1768097 | RPGR | 0.73 | 2.13 | 1.94 | 2.79 | 8.04 | 7.96 | 7.81 | 7.83 | 7.74 |
| ILMN_1757736 | IRX5 | 0.74 | 1.49 | 1.81 | 4.61 | 7.54 | 7.41 | 7.29 | 7.23 | 6.76 |
| ILMN_3245707 | RIMKLB | 0.74 | -0.17 | 0.85 | -4.68 | 7.50 | 7.37 | 7.53 | 7.35 | 8.32 |
| ILMN_2400922 | OPRL1 | 0.74 | -0.29 | -2.36 | -5.89 | 8.06 | 7.98 | 8.09 | 8.33 | 8.73 |
| ILMN_2186806 | HLA-F | 0.74 | 1.40 | 1.83 | 3.70 | 11.11 | 10.94 | 10.79 | 10.70 | 10.27 |
| ILMN_2320850 | UBE2D3 | 0.74 | 1.98 | 2.41 | 4.39 | 11.25 | 11.16 | 11.01 | 10.96 | 10.73 |
| ILMN_1680692 | NUCKS1 | 0.74 | 1.30 | 0.99 | 3.14 | 9.58 | 9.39 | 9.24 | 9.32 | 8.76 |
| ILMN_2289849 | FCGR2A | 0.74 | 1.40 | 0.54 | 3.38 | 7.26 | 7.20 | 7.15 | 7.22 | 6.99 |
| ILMN_3208715 | LOC440063 | 0.74 | 1.80 | 0.99 | 3.88 | 13.17 | 13.02 | 12.80 | 12.97 | 12.37 |
| ILMN_2393060 | PPIL3 | 0.75 | 3.50 | 3.09 | 4.44 | 7.71 | 7.64 | 7.40 | 7.43 | 7.31 |
| ILMN_2326282 | C19orf6 | 0.75 | -2.22 | -1.05 | -4.20 | 8.98 | 8.90 | 9.23 | 9.10 | 9.45 |
| ILMN_1815745 | SOX4 | 0.75 | -1.16 | -1.22 | -3.91 | 9.48 | 9.39 | 9.61 | 9.62 | 9.93 |
| ILMN_1803939 | YIPF6 | 0.75 | -0.39 | 0.39 | 4.27 | 8.60 | 8.51 | 8.64 | 8.55 | 8.09 |
| ILMN_1726667 | LOC648638 | 0.75 | 1.53 | 1.03 | 3.35 | 8.85 | 8.73 | 8.60 | 8.68 | 8.31 |
| ILMN_1784256 | HDGFRP3 | 0.75 | 0.40 | 1.66 | 8.61 | 8.55 | 8.44 | 8.49 | 8.30 | 7.26 |
| ILMN_1679920 | LOC651894 | 0.76 | 1.31 | 2.78 | 2.75 | 11.79 | 11.72 | 11.66 | 11.52 | 11.52 |
| ILMN_1691430 | GSTCD | 0.76 | -1.23 | -1.42 | 3.26 | 7.00 | 6.95 | 7.09 | 7.10 | 6.77 |
| ILMN_2083833 | CNOT6L | 0.76 | 2.52 | 1.01 | 2.78 | 7.23 | 7.17 | 7.03 | 7.15 | 7.01 |
| ILMN_1786388 | RNF113A | 0.76 | -0.07 | -0.43 | 3.89 | 8.97 | 8.85 | 8.98 | 9.04 | 8.37 |
| ILMN_3277715 | LOC389873 | 0.76 | 1.76 | 1.26 | 3.26 | 8.63 | 8.56 | 8.46 | 8.51 | 8.31 |
| ILMN_2140559 | IRX5 | 0.76 | 2.44 | 2.77 | 7.03 | 7.63 | 7.54 | 7.35 | 7.31 | 6.83 |
| ILMN_1681101 | 41334 | 0.77 | 2.19 | 1.69 | 4.03 | 7.01 | 6.94 | 6.82 | 6.86 | 6.66 |
| ILMN_1695969 | PRSS7 | 0.77 | 1.42 | -0.39 | 5.67 | 7.28 | 7.22 | 7.17 | 7.31 | 6.84 |
| ILMN_1786976 | RAB22A | 0.77 | -0.84 | -1.39 | -4.06 | 9.05 | 8.97 | 9.14 | 9.19 | 9.47 |
| ILMN_1804822 | SRXN1 | 0.78 | -0.24 | -0.66 | -3.33 | 7.67 | 7.56 | 7.71 | 7.77 | 8.14 |
| ILMN_1783681 | MRPL34 | 0.78 | 0.30 | -0.05 | -4.44 | 8.90 | 8.80 | 8.86 | 8.91 | 9.45 |
| ILMN_1812312 | NDUFS4 | 0.78 | 2.53 | 2.62 | 6.49 | 10.54 | 10.44 | 10.23 | 10.22 | 9.76 |
| ILMN_3289171 | LOC100131572 | 0.78 | 1.30 | 1.54 | 2.83 | 10.17 | 10.08 | 10.03 | 10.00 | 9.86 |
| ILMN_1780699 | THAP11 | 0.79 | -0.21 | -0.21 | 5.07 | 10.38 | 10.31 | 10.40 | 10.40 | 9.91 |
| ILMN_1674588 | HAO1 | 0.79 | 2.52 | 2.12 | 3.07 | 6.94 | 6.87 | 6.73 | 6.76 | 6.68 |
| ILMN_1802162 | RFESD | 0.79 | 2.03 | 3.78 | 2.95 | 7.26 | 7.20 | 7.10 | 6.96 | 7.03 |
| ILMN_1756455 | IL5RA | 0.79 | 1.95 | 1.79 | 5.23 | 7.88 | 7.77 | 7.60 | 7.62 | 7.13 |
| ILMN_1803743 | LOC196752 | 0.79 | -1.06 | -2.30 | -3.66 | 7.09 | 7.02 | 7.18 | 7.29 | 7.41 |
| ILMN_1685170 | ANXA11 | 0.79 | 0.09 | 0.61 | 2.95 | 7.83 | 7.71 | 7.82 | 7.74 | 7.40 |
| ILMN_1752953 | BCL2L12 | 0.79 | -0.25 | -0.29 | -3.32 | 9.31 | 9.23 | 9.33 | 9.33 | 9.61 |
| ILMN_2332267 | CSNK1G3 | 0.80 | 2.31 | 2.41 | 4.22 | 8.37 | 8.21 | 7.92 | 7.90 | 7.56 |
| ILMN_1792283 | RPP30 | 0.80 | 0.22 | 1.24 | 2.75 | 6.91 | 6.85 | 6.89 | 6.81 | 6.70 |
| ILMN_1768867 | AP3B1 | 0.80 | 2.06 | 2.34 | 3.90 | 12.13 | 11.96 | 11.70 | 11.64 | 11.32 |
| ILMN_1673369 | SEPHS1 | 0.80 | 0.91 | 0.42 | 2.98 | 9.41 | 9.29 | 9.27 | 9.35 | 8.96 |
| ILMN_1747217 | C15orf41 | 0.80 | 0.77 | 1.57 | 3.38 | 7.62 | 7.54 | 7.55 | 7.47 | 7.30 |
| ILMN_1777584 | KARS | 0.80 | 1.20 | 0.26 | 4.74 | 9.82 | 9.75 | 9.71 | 9.80 | 9.39 |
| ILMN_1710522 | RUNX1T1 | 0.80 | 1.56 | -0.99 | 2.79 | 6.84 | 6.78 | 6.73 | 6.91 | 6.64 |
| ILMN_2196569 | NUP93 | 0.80 | 0.48 | 0.90 | 5.79 | 10.59 | 10.51 | 10.54 | 10.50 | 9.99 |
| ILMN_1670875 | PPM1D | 0.81 | 0.99 | 2.45 | 4.06 | 8.97 | 8.89 | 8.87 | 8.71 | 8.54 |
| ILMN_3244323 | LOC148413 | 0.82 | -1.14 | 0.41 | -3.02 | 9.80 | 9.70 | 9.94 | 9.75 | 10.17 |
| ILMN_1674386 | PITX1 | 0.82 | 1.31 | 0.96 | 2.79 | 9.56 | 9.28 | 9.12 | 9.24 | 8.62 |
| ILMN_1675239 | NDUFA7 | 0.83 | -0.22 | 0.45 | -2.72 | 10.00 | 9.92 | 10.03 | 9.96 | 10.29 |
| ILMN_1799024 | VAC14 | 0.83 | 0.44 | 1.23 | 3.16 | 8.27 | 8.15 | 8.21 | 8.09 | 7.79 |
| ILMN_1745415 | BBX | 0.83 | 0.57 | 0.97 | 4.57 | 8.74 | 8.61 | 8.65 | 8.59 | 8.04 |
| ILMN_1658290 | C16orf68 | 0.83 | 1.84 | 2.09 | 3.48 | 8.69 | 8.59 | 8.47 | 8.44 | 8.27 |
| ILMN_1764867 | LOC649431 | 0.84 | 2.55 | 2.29 | 3.65 | 6.96 | 6.89 | 6.76 | 6.78 | 6.68 |
| ILMN_2183856 | TSPAN6 | 0.84 | 0.36 | 0.04 | 2.91 | 7.08 | 7.01 | 7.05 | 7.08 | 6.84 |
| ILMN_1745852 | WDR33 | 0.84 | -2.59 | -0.61 | -4.94 | 7.92 | 7.82 | 8.23 | 8.00 | 8.51 |
| ILMN_1684563 | SPIN4 | 0.84 | -0.81 | -0.57 | 5.40 | 9.98 | 9.89 | 10.07 | 10.04 | 9.38 |
| ILMN_1701434 | RAP1B | 0.84 | -0.49 | 0.68 | -3.31 | 7.92 | 7.81 | 7.98 | 7.83 | 8.32 |
| ILMN_2269256 | DNAJC12 | 0.84 | 0.84 | 2.94 | 4.81 | 8.18 | 8.01 | 8.01 | 7.59 | 7.21 |
| ILMN_2303955 | FKBP1B | 0.85 | 0.13 | 0.49 | 2.81 | 7.12 | 7.06 | 7.11 | 7.09 | 6.91 |
| ILMN_2188204 | ATG12 | 0.85 | 1.80 | 3.34 | 2.92 | 9.57 | 9.46 | 9.34 | 9.15 | 9.20 |
| ILMN_1797534 | RIOK1 | 0.85 | 2.49 | 4.00 | 8.37 | 9.37 | 9.32 | 9.23 | 9.15 | 8.91 |
| ILMN_1831834 |  | 0.85 | 1.02 | 1.36 | 3.36 | 6.98 | 6.91 | 6.90 | 6.87 | 6.72 |
| ILMN_1738095 | PER2 | 0.85 | 0.23 | -0.65 | -2.87 | 9.61 | 9.51 | 9.59 | 9.69 | 9.95 |
| ILMN_1776325 | UBE2Q1 | 0.85 | -1.05 | -0.92 | -3.08 | 8.27 | 8.18 | 8.37 | 8.36 | 8.58 |
| ILMN_1736567 | CD74 | 0.85 | 1.35 | 1.48 | 3.00 | 13.24 | 13.14 | 13.08 | 13.06 | 12.88 |
| ILMN_1730291 | ATP1B1 | 0.85 | -0.44 | 0.52 | 3.22 | 7.26 | 7.15 | 7.31 | 7.19 | 6.87 |
| ILMN_1715401 | MT1G | 0.85 | 1.11 | 5.56 | 5.34 | 11.02 | 10.64 | 10.52 | 8.53 | 8.62 |
| ILMN_2094938 | OMA1 | 0.85 | 2.06 | 1.67 | 2.92 | 7.36 | 7.28 | 7.17 | 7.21 | 7.09 |
| ILMN_1653718 | ZFAND2B | 0.85 | 2.11 | 2.86 | 2.78 | 9.79 | 9.69 | 9.53 | 9.44 | 9.45 |
| ILMN_2333865 | DNAJB12 | 0.85 | 0.60 | 0.56 | 3.76 | 7.92 | 7.85 | 7.87 | 7.88 | 7.63 |
| ILMN_1798256 | UPP1 | 0.86 | 0.19 | 1.08 | -5.34 | 7.73 | 7.63 | 7.71 | 7.60 | 8.37 |
| ILMN_1746138 | RLTPR | 0.86 | -1.34 | 0.52 | 3.68 | 7.31 | 7.23 | 7.44 | 7.26 | 6.96 |
| ILMN_1775423 | C10orf88 | 0.86 | 2.68 | 3.44 | 5.68 | 8.85 | 8.79 | 8.66 | 8.61 | 8.45 |
| ILMN_1732831 | CHST7 | 0.86 | -0.08 | -0.41 | -4.02 | 8.43 | 8.35 | 8.44 | 8.47 | 8.79 |
| ILMN_1810127 | ZNF789 | 0.86 | -0.24 | 0.71 | -3.20 | 8.40 | 8.26 | 8.43 | 8.29 | 8.89 |
| ILMN_1713918 | CYTH3 | 0.87 | -0.14 | -1.28 | -2.91 | 7.28 | 7.19 | 7.29 | 7.40 | 7.56 |
| ILMN_1691772 | ZSCAN29 | 0.87 | 0.74 | 2.69 | 3.05 | 7.54 | 7.45 | 7.46 | 7.26 | 7.22 |
| ILMN_1789624 | TPM2 | 0.87 | 1.03 | 0.93 | -2.93 | 6.75 | 6.66 | 6.64 | 6.65 | 7.05 |
| ILMN_1769783 | ZDHHC2 | 0.87 | 2.20 | -1.85 | 5.09 | 7.68 | 7.63 | 7.55 | 7.79 | 7.39 |
| ILMN_1676528 | BTN3A2 | 0.87 | 1.56 | 1.14 | 4.32 | 8.13 | 8.00 | 7.90 | 7.96 | 7.50 |
| ILMN_1749081 | AUTS2 | 0.87 | 0.64 | 2.17 | 4.99 | 9.52 | 9.34 | 9.39 | 9.08 | 8.51 |
| ILMN_1670748 | GALNT7 | 0.87 | 0.58 | 0.96 | 2.83 | 6.97 | 6.90 | 6.92 | 6.89 | 6.74 |
| ILMN_2100815 | TMEM9B | 0.87 | 0.41 | 2.85 | -3.63 | 8.27 | 8.20 | 8.24 | 8.06 | 8.53 |
| ILMN_3236675 | LOC100133823 | 0.87 | -0.22 | 0.06 | -3.18 | 7.80 | 7.66 | 7.84 | 7.79 | 8.34 |
| ILMN_1712985 | C17orf58 | 0.88 | 2.92 | 3.05 | 3.60 | 8.14 | 8.05 | 7.84 | 7.82 | 7.77 |
| ILMN_1659285 | PSMG1 | 0.89 | 2.69 | 4.02 | 1.80 | 10.54 | 10.45 | 10.26 | 10.12 | 10.35 |
| ILMN_2094942 | 41334 | 0.89 | 1.90 | 1.11 | 4.92 | 7.68 | 7.55 | 7.39 | 7.51 | 6.94 |
| ILMN_2360291 | UGCGL1 | 0.89 | -0.18 | -2.81 | -3.29 | 8.05 | 7.97 | 8.07 | 8.30 | 8.35 |
| ILMN_1667030 | HSBP1 | 0.89 | 0.44 | 1.26 | 4.32 | 11.24 | 11.11 | 11.17 | 11.06 | 10.62 |
| ILMN_1688127 | LOC341457 | 0.89 | 2.18 | 2.18 | 3.31 | 14.17 | 14.07 | 13.93 | 13.93 | 13.81 |
| ILMN_1660359 | LOC654126 | 0.89 | 0.98 | 1.87 | 2.97 | 7.40 | 7.33 | 7.32 | 7.25 | 7.17 |
| ILMN_1687785 | PPA2 | 0.89 | 0.77 | 0.71 | 4.90 | 10.21 | 10.11 | 10.12 | 10.13 | 9.68 |
| ILMN_1717834 | EIF1AD | 0.89 | 0.43 | 0.83 | 3.14 | 7.50 | 7.41 | 7.46 | 7.41 | 7.16 |
| ILMN_1666545 | GCNT1 | 0.89 | 1.69 | 1.21 | -4.82 | 8.42 | 8.32 | 8.24 | 8.29 | 8.92 |
| ILMN_1717261 | HLA-DRB3 | 0.89 | 0.36 | -0.50 | 3.26 | 9.94 | 9.85 | 9.90 | 9.98 | 9.63 |
| ILMN_1777325 | STAT1 | 0.90 | 1.72 | 2.39 | 2.91 | 11.22 | 10.97 | 10.74 | 10.55 | 10.41 |
| ILMN_1804834 | C6orf130 | 0.90 | 4.03 | 4.93 | 5.21 | 10.42 | 10.33 | 9.99 | 9.89 | 9.86 |
| ILMN_2310968 | RUFY1 | 0.90 | 1.57 | 1.24 | 2.89 | 7.82 | 7.74 | 7.68 | 7.71 | 7.56 |
| ILMN_3205924 | LOC651198 | 0.90 | 2.50 | 3.91 | 6.41 | 8.19 | 8.13 | 8.02 | 7.93 | 7.76 |
| ILMN_1714956 | PLA2G12A | 0.90 | 1.13 | 2.62 | 3.34 | 7.00 | 6.93 | 6.91 | 6.80 | 6.75 |
| ILMN_1667050 | PRPS1 | 0.90 | 0.37 | 0.84 | 5.76 | 10.37 | 10.28 | 10.33 | 10.28 | 9.76 |
| ILMN_1816582 |  | 0.90 | 0.94 | 1.81 | 2.83 | 6.99 | 6.92 | 6.91 | 6.84 | 6.76 |
| ILMN_1689774 | MRFAP1L1 | 0.90 | 1.39 | 1.86 | 6.26 | 11.14 | 11.02 | 10.95 | 10.89 | 10.29 |
| ILMN_1662852 | IQCK | 0.90 | 2.23 | 2.68 | 3.97 | 7.62 | 7.53 | 7.41 | 7.36 | 7.24 |
| ILMN_2047856 | EFHA2 | 0.91 | 0.00 | 1.77 | 4.45 | 7.05 | 6.98 | 7.05 | 6.91 | 6.70 |
| ILMN_1749821 | MED28 | 0.91 | 1.25 | 2.41 | 4.17 | 10.40 | 10.30 | 10.26 | 10.14 | 9.95 |
| ILMN_1652160 | LRBA | 0.91 | 1.73 | 0.79 | 2.76 | 7.46 | 7.36 | 7.26 | 7.37 | 7.15 |
| ILMN_2394975 | OTUD4 | 0.91 | 2.22 | 0.57 | 2.73 | 6.92 | 6.87 | 6.79 | 6.89 | 6.76 |
| ILMN_1809477 | CARHSP1 | 0.92 | 0.42 | -0.11 | 3.74 | 11.06 | 10.94 | 11.01 | 11.07 | 10.59 |
| ILMN_3271122 | LOC100129742 | 0.92 | 0.92 | 0.82 | 3.09 | 13.15 | 12.96 | 12.96 | 12.98 | 12.53 |
| ILMN_1693494 | CMTM1 | 0.93 | -0.87 | 0.60 | 2.89 | 6.81 | 6.76 | 6.87 | 6.78 | 6.64 |
| ILMN_3264073 | LOC100130070 | 0.93 | 2.07 | 1.85 | 3.60 | 11.81 | 11.72 | 11.61 | 11.63 | 11.47 |
| ILMN_1783709 | RRAGA | 0.93 | 0.55 | 0.16 | 3.63 | 10.42 | 10.31 | 10.35 | 10.40 | 9.98 |
| ILMN_2257833 | BBS7 | 0.93 | 2.46 | 1.29 | 4.95 | 8.78 | 8.68 | 8.51 | 8.64 | 8.23 |
| ILMN_1805225 | LPCAT3 | 0.93 | 0.34 | 2.41 | -3.66 | 8.82 | 8.73 | 8.79 | 8.59 | 9.18 |
| ILMN_2415144 | SP110 | 0.93 | 2.36 | 3.03 | 3.85 | 9.65 | 9.19 | 8.48 | 8.15 | 7.74 |
| ILMN_1721729 | PPARBP | 0.94 | 0.91 | 1.54 | 3.25 | 9.38 | 9.25 | 9.25 | 9.16 | 8.92 |
| ILMN_1670970 | PPP3CA | 0.94 | 0.23 | 0.74 | 2.81 | 7.55 | 7.45 | 7.52 | 7.47 | 7.27 |
| ILMN_1792712 | LOC201725 | 0.94 | -0.71 | -0.50 | 4.44 | 9.39 | 9.28 | 9.47 | 9.44 | 8.88 |
| ILMN_1772286 | OCIAD2 | 0.94 | 0.97 | -0.32 | 4.54 | 9.36 | 9.26 | 9.26 | 9.39 | 8.89 |
| ILMN_1690386 | CSRP2BP | 0.94 | 0.94 | 4.40 | 0.64 | 9.31 | 9.22 | 9.22 | 8.92 | 9.25 |
| ILMN_3251691 | POLR3G | 0.94 | 1.79 | 2.71 | 3.37 | 8.07 | 7.97 | 7.88 | 7.79 | 7.72 |
| ILMN_1700147 | VPREB3 | 0.94 | -1.12 | -1.17 | -3.01 | 10.27 | 10.04 | 10.54 | 10.55 | 10.99 |
| ILMN_1677534 | SCAP | 0.95 | -2.72 | -0.95 | -2.84 | 9.82 | 9.72 | 10.12 | 9.92 | 10.13 |
| ILMN_1687508 | ALDH7A1 | 0.95 | 2.44 | 3.02 | 4.08 | 8.37 | 8.08 | 7.64 | 7.46 | 7.15 |
| ILMN_1729417 | GNE | 0.95 | 1.16 | 1.73 | 2.85 | 8.87 | 8.70 | 8.67 | 8.57 | 8.38 |
| ILMN_3309349 | SNHG8 | 0.95 | 2.31 | 3.63 | 4.72 | 11.80 | 11.64 | 11.40 | 11.18 | 10.99 |
| ILMN_1681301 | AIM2 | 0.95 | 3.74 | 3.62 | 4.64 | 8.07 | 7.93 | 7.52 | 7.53 | 7.38 |
| ILMN_1711566 | TIMP1 | 0.95 | -0.46 | -0.08 | -7.08 | 6.96 | 6.89 | 7.00 | 6.97 | 7.53 |
| ILMN_2210713 | COMMD8 | 0.95 | 1.21 | 0.56 | 3.38 | 7.69 | 7.60 | 7.57 | 7.64 | 7.35 |
| ILMN_2102960 | KIAA1370 | 0.95 | 1.74 | 0.73 | 3.06 | 9.87 | 9.76 | 9.67 | 9.79 | 9.51 |
| ILMN_1721138 | GRPEL2 | 0.95 | 2.83 | 3.70 | 4.17 | 9.51 | 9.40 | 9.19 | 9.09 | 9.04 |
| ILMN_2207539 | RPS17 | 0.95 | 2.07 | 2.10 | 4.65 | 13.20 | 13.10 | 12.98 | 12.98 | 12.71 |
| ILMN_2101810 | ARHGAP12 | 0.96 | 1.59 | 0.14 | 5.13 | 7.51 | 7.42 | 7.36 | 7.50 | 7.03 |
| ILMN_1752199 | LHPP | 0.96 | -1.70 | -1.31 | 3.65 | 8.07 | 7.91 | 8.36 | 8.29 | 7.45 |
| ILMN_3236346 | LOC100132901 | 0.96 | 0.14 | 2.39 | 3.54 | 7.04 | 6.97 | 7.03 | 6.87 | 6.79 |
| ILMN_1709439 | CHMP1A | 0.96 | -0.17 | 0.32 | 3.41 | 9.06 | 8.93 | 9.08 | 9.01 | 8.59 |
| ILMN_1756898 | COQ9 | 0.96 | 2.00 | 1.45 | 5.96 | 9.88 | 9.76 | 9.64 | 9.70 | 9.15 |
| ILMN_2122669 | TOPORS | 0.96 | 2.68 | 2.99 | 5.17 | 7.61 | 7.52 | 7.37 | 7.35 | 7.16 |
| ILMN_1782551 | E2F5 | 0.96 | 2.08 | 1.92 | 4.29 | 9.46 | 9.36 | 9.25 | 9.26 | 9.02 |
| ILMN_1667306 | RANBP10 | 0.96 | -0.19 | 0.19 | 3.65 | 8.08 | 7.96 | 8.10 | 8.06 | 7.62 |
| ILMN_1741253 | UBR5 | 0.96 | 2.72 | 3.25 | 3.22 | 8.68 | 8.58 | 8.39 | 8.33 | 8.34 |
| ILMN_2294274 | S100PBP | 0.96 | 0.99 | 2.26 | 3.09 | 9.07 | 8.98 | 8.98 | 8.85 | 8.77 |
| ILMN_2315694 | STRADA | 0.96 | 2.21 | 2.37 | 4.49 | 9.42 | 9.34 | 9.24 | 9.23 | 9.05 |
| ILMN_2189859 | FLJ38482 | 0.96 | 0.96 | 0.69 | 4.62 | 8.40 | 8.32 | 8.32 | 8.34 | 8.00 |
| ILMN_2181883 | C14orf129 | 0.96 | 3.01 | 2.39 | 3.16 | 7.88 | 7.80 | 7.62 | 7.68 | 7.61 |
| ILMN_2358069 | MAD1L1 | 0.96 | 0.16 | 1.76 | 3.43 | 7.95 | 7.81 | 7.92 | 7.70 | 7.46 |
| ILMN_1662640 | C20orf127 | 0.97 | 1.54 | 4.31 | 3.99 | 7.63 | 7.50 | 7.42 | 7.05 | 7.09 |
| ILMN_1776213 | RGMB | 0.97 | 0.87 | 2.05 | 3.35 | 7.95 | 7.81 | 7.83 | 7.66 | 7.48 |
| ILMN_3251365 | PGGT1B | 0.97 | 0.92 | 1.89 | 3.05 | 7.12 | 7.05 | 7.05 | 6.99 | 6.91 |
| ILMN_1740035 | POU4F2 | 0.97 | 0.28 | 1.73 | 4.14 | 7.23 | 7.09 | 7.19 | 6.99 | 6.65 |
| ILMN_1804629 | TPK1 | 0.97 | 2.50 | 2.55 | 2.90 | 7.52 | 7.39 | 7.20 | 7.19 | 7.15 |
| ILMN_1761049 | EIF2C3 | 0.97 | 1.44 | 2.36 | 3.02 | 7.77 | 7.70 | 7.67 | 7.61 | 7.57 |
| ILMN_1680129 | NSUN2 | 0.97 | 2.62 | 3.13 | 3.27 | 10.55 | 10.41 | 10.17 | 10.10 | 10.08 |
| ILMN_1770515 | UBE2V2 | 0.97 | 2.12 | 1.37 | 3.22 | 9.93 | 9.80 | 9.65 | 9.75 | 9.50 |
| ILMN_1683923 | MT1H | 0.97 | 0.93 | 3.72 | 4.08 | 7.27 | 7.12 | 7.13 | 6.72 | 6.67 |
| ILMN_2310703 | RPS26L | 0.98 | 0.12 | 0.15 | -3.15 | 10.95 | 10.82 | 10.94 | 10.93 | 11.38 |
| ILMN_2090782 | GCSH | 0.98 | 1.32 | 2.09 | 4.04 | 9.10 | 8.93 | 8.87 | 8.73 | 8.39 |
| ILMN_3207738 | LOC646527 | 0.98 | 0.86 | 1.50 | 3.85 | 11.53 | 11.32 | 11.35 | 11.21 | 10.70 |
| ILMN_1655433 | BCKDHB | 0.98 | -0.41 | 1.51 | 2.82 | 7.42 | 7.34 | 7.45 | 7.30 | 7.19 |
| ILMN_3245869 | LOC440957 | 0.98 | 0.10 | 1.76 | -2.87 | 8.67 | 8.54 | 8.65 | 8.44 | 9.04 |
| ILMN_1737015 | RPL39 | 0.98 | 1.61 | 0.54 | 3.35 | 13.78 | 13.70 | 13.66 | 13.74 | 13.53 |
| ILMN_1654151 | COX6C | 0.98 | 3.20 | 3.83 | 0.53 | 12.14 | 12.05 | 11.84 | 11.78 | 12.09 |
| ILMN_1777220 | VCP | 0.98 | 2.17 | 1.63 | 4.11 | 10.17 | 10.07 | 9.95 | 10.01 | 9.76 |
| ILMN_1654851 | FAM134A | 0.98 | 2.38 | 2.50 | -4.39 | 8.04 | 7.96 | 7.85 | 7.84 | 8.40 |
| ILMN_3248833 | LOC644928 | 0.99 | -0.62 | -0.23 | -3.29 | 10.27 | 10.15 | 10.35 | 10.30 | 10.70 |
| ILMN_2077758 | CTDSPL2 | 0.99 | -0.53 | 0.96 | 2.77 | 8.65 | 8.55 | 8.70 | 8.55 | 8.37 |
| ILMN_1753490 | C10orf39 | 0.99 | 0.72 | 2.19 | -2.74 | 7.16 | 7.06 | 7.09 | 6.95 | 7.43 |
| ILMN_1721558 | FLJ44313 | 0.99 | 1.28 | 1.32 | 3.35 | 7.14 | 7.07 | 7.05 | 7.04 | 6.90 |
| ILMN_1691341 | IL7R | 0.99 | -0.58 | 0.63 | 4.22 | 7.69 | 7.48 | 7.81 | 7.55 | 6.79 |
| ILMN_2136089 | MTE | 0.99 | 1.20 | 4.78 | 4.52 | 7.75 | 7.60 | 7.56 | 7.00 | 7.04 |
| ILMN_1685016 | LOC645099 | 1.00 | 3.30 | 4.08 | 4.89 | 9.35 | 9.17 | 8.75 | 8.60 | 8.45 |
| ILMN_1693334 | P4HA1 | 1.00 | 1.25 | -0.50 | 3.24 | 8.82 | 8.73 | 8.70 | 8.87 | 8.52 |
| ILMN_1747160 | SYT15 | 1.00 | 0.40 | 1.23 | 2.73 | 8.14 | 8.04 | 8.10 | 8.01 | 7.86 |
| ILMN_2106725 | NCF1B | 1.00 | 0.63 | 1.37 | 3.63 | 6.84 | 6.77 | 6.80 | 6.75 | 6.61 |
| ILMN_1726809 | BHLHB3 | 1.00 | 2.66 | 2.00 | 3.41 | 7.81 | 7.70 | 7.52 | 7.59 | 7.44 |
| ILMN_1776102 | PSMD10 | 1.01 | 1.98 | 0.81 | 3.51 | 9.60 | 9.49 | 9.39 | 9.51 | 9.24 |
| ILMN_1792997 | NPTN | 1.01 | 0.97 | -0.23 | 3.18 | 9.47 | 9.38 | 9.38 | 9.49 | 9.19 |
| ILMN_1864422 |  | 1.01 | 0.86 | 0.90 | 2.80 | 9.00 | 8.91 | 8.92 | 8.92 | 8.75 |
| ILMN_1704195 | FUK | 1.01 | 0.00 | -0.22 | 3.67 | 7.37 | 7.28 | 7.37 | 7.39 | 7.03 |
| ILMN_1798187 | MYST2 | 1.01 | 1.88 | -0.38 | 3.08 | 7.28 | 7.21 | 7.15 | 7.31 | 7.07 |
| ILMN_1777483 | APOOL | 1.02 | 2.20 | 0.65 | 4.32 | 7.42 | 7.33 | 7.24 | 7.36 | 7.06 |
| ILMN_1755120 | MAN1A2 | 1.02 | 2.76 | 1.74 | 3.94 | 7.40 | 7.30 | 7.12 | 7.23 | 7.00 |
| ILMN_1682781 | TEAD2 | 1.02 | 0.27 | -0.29 | 3.89 | 10.33 | 10.19 | 10.29 | 10.37 | 9.79 |
| ILMN_1815168 | HVCN1 | 1.02 | -0.67 | -0.07 | -5.09 | 7.50 | 7.36 | 7.60 | 7.51 | 8.22 |
| ILMN_2178618 | WDR44 | 1.02 | 1.68 | 0.28 | 6.66 | 7.68 | 7.61 | 7.56 | 7.66 | 7.20 |
| ILMN_1709307 | GPSM1 | 1.03 | -0.58 | 0.58 | 2.85 | 8.95 | 8.70 | 9.09 | 8.81 | 8.26 |
| ILMN_3245476 | PHRF1 | 1.03 | -1.71 | -0.24 | -3.05 | 10.31 | 10.21 | 10.48 | 10.33 | 10.61 |
| ILMN_3293676 | LOC100131387 | 1.03 | 1.25 | 1.06 | 4.08 | 13.69 | 13.55 | 13.52 | 13.54 | 13.13 |
| ILMN_1903021 |  | 1.03 | 0.89 | -0.17 | 2.93 | 7.25 | 7.13 | 7.15 | 7.27 | 6.90 |
| ILMN_1846922 |  | 1.03 | -2.37 | 0.49 | -2.79 | 9.08 | 8.94 | 9.38 | 9.01 | 9.44 |
| ILMN_2285375 | SORD | 1.03 | 2.01 | 0.85 | 5.85 | 9.48 | 9.35 | 9.22 | 9.37 | 8.74 |
| ILMN_1724350 | TRIM36 | 1.03 | 0.33 | -0.03 | 3.30 | 7.03 | 6.93 | 7.00 | 7.03 | 6.70 |
| ILMN_2164081 | KLHL12 | 1.04 | 2.26 | 4.18 | 2.19 | 9.92 | 9.83 | 9.72 | 9.55 | 9.73 |
| ILMN_1746561 | BCL2L2 | 1.04 | 0.33 | 1.01 | -2.78 | 7.40 | 7.26 | 7.35 | 7.26 | 7.76 |
| ILMN_1708907 | MEIG1 | 1.04 | 2.41 | 1.44 | 4.96 | 7.73 | 7.63 | 7.49 | 7.59 | 7.24 |
| ILMN_1811181 | FLJ20444 | 1.04 | 1.17 | 2.34 | 5.80 | 9.28 | 9.14 | 9.13 | 8.98 | 8.53 |
| ILMN_1675460 | LOC283412 | 1.04 | 1.04 | 1.63 | 2.73 | 12.62 | 12.50 | 12.50 | 12.43 | 12.30 |
| ILMN_1729533 | APOA1BP | 1.05 | 0.45 | 1.28 | -2.75 | 10.69 | 10.56 | 10.63 | 10.54 | 11.01 |
| ILMN_1806603 | MESP1 | 1.05 | -1.05 | -1.02 | -3.97 | 7.13 | 7.02 | 7.25 | 7.25 | 7.57 |
| ILMN_1724062 | LIN54 | 1.05 | 0.67 | 0.56 | 3.68 | 8.44 | 8.34 | 8.38 | 8.39 | 8.09 |
| ILMN_3294222 | LOC100132673 | 1.06 | 1.73 | 2.33 | 4.68 | 12.93 | 12.74 | 12.61 | 12.50 | 12.07 |
| ILMN_2159694 | HLA-DRB4 | 1.06 | 0.99 | 0.41 | 4.48 | 10.19 | 10.03 | 10.04 | 10.13 | 9.54 |
| ILMN_3278754 | LOC649445 | 1.06 | -0.29 | 1.54 | 3.32 | 7.64 | 7.57 | 7.66 | 7.54 | 7.41 |
| ILMN_1716368 | CCDC111 | 1.06 | 1.36 | 1.29 | 2.94 | 7.22 | 7.11 | 7.08 | 7.09 | 6.92 |
| ILMN_1749634 | PLRG1 | 1.06 | 1.11 | 1.17 | 2.88 | 10.52 | 10.35 | 10.35 | 10.34 | 10.06 |
| ILMN_2342579 | IL7R | 1.06 | -0.34 | 1.06 | 3.98 | 6.94 | 6.82 | 6.97 | 6.82 | 6.51 |
| ILMN_1659777 | RUFY1 | 1.07 | 1.79 | 2.40 | 3.62 | 7.72 | 7.63 | 7.57 | 7.51 | 7.41 |
| ILMN_2413780 | SEZ6L2 | 1.07 | -0.26 | 0.33 | -3.12 | 6.70 | 6.60 | 6.72 | 6.67 | 6.98 |
| ILMN_1715384 | B3GNT6 | 1.07 | -1.62 | 0.48 | -2.91 | 7.50 | 7.41 | 7.65 | 7.46 | 7.77 |
| ILMN_1781010 | ARHGEF3 | 1.07 | 2.64 | 2.23 | 7.83 | 10.13 | 10.02 | 9.85 | 9.90 | 9.30 |
| ILMN_1770020 | PPIL3 | 1.07 | 2.54 | 3.10 | 4.04 | 11.97 | 11.83 | 11.65 | 11.58 | 11.46 |
| ILMN_1660412 | MGC27345 | 1.07 | -0.31 | 0.71 | -3.89 | 7.21 | 7.13 | 7.24 | 7.16 | 7.50 |
| ILMN_1698225 | MYO5A | 1.07 | 2.41 | 1.94 | 4.26 | 8.68 | 8.56 | 8.41 | 8.46 | 8.20 |
| ILMN_3200539 | LOC100131672 | 1.07 | 1.20 | 1.80 | 3.47 | 11.24 | 11.13 | 11.11 | 11.05 | 10.87 |
| ILMN_1660292 | MRPS21 | 1.07 | 0.67 | 1.48 | -2.88 | 9.62 | 9.43 | 9.50 | 9.36 | 10.12 |
| ILMN_1676026 | MRPS26 | 1.08 | -1.78 | -0.62 | -3.63 | 9.79 | 9.67 | 9.98 | 9.86 | 10.18 |
| ILMN_1714515 | MRPS12 | 1.08 | 1.28 | -0.23 | -2.96 | 8.89 | 8.74 | 8.72 | 8.92 | 9.28 |
| ILMN_3187357 | LOC100130746 | 1.08 | 0.17 | 1.00 | 6.39 | 7.37 | 7.28 | 7.35 | 7.29 | 6.85 |
| ILMN_1740216 | ERCC3 | 1.08 | -0.55 | -0.02 | -2.81 | 8.44 | 8.27 | 8.52 | 8.44 | 8.88 |
| ILMN_2157709 | C14orf19 | 1.08 | 2.85 | 1.88 | 3.98 | 7.73 | 7.59 | 7.36 | 7.48 | 7.21 |
| ILMN_1720850 | BAZ2B | 1.08 | 1.26 | 3.48 | 4.00 | 8.37 | 8.11 | 8.07 | 7.54 | 7.42 |
| ILMN_1684628 | ZFP90 | 1.08 | -0.15 | 2.41 | 4.91 | 8.85 | 8.74 | 8.87 | 8.59 | 8.32 |
| ILMN_2143314 | SPIB | 1.08 | 0.06 | 0.47 | 2.75 | 9.94 | 9.81 | 9.93 | 9.88 | 9.61 |
| ILMN_1891851 |  | 1.08 | 1.77 | 1.48 | 2.85 | 6.95 | 6.88 | 6.84 | 6.86 | 6.78 |
| ILMN_1675583 | SPG7 | 1.08 | 0.12 | 0.32 | 5.82 | 7.55 | 7.46 | 7.54 | 7.53 | 7.07 |
| ILMN_1704713 | CSNK1G1 | 1.09 | 1.17 | 1.53 | 2.82 | 8.59 | 8.50 | 8.50 | 8.47 | 8.36 |
| ILMN_1654915 | LOC646786 | 1.09 | 0.60 | 0.17 | 4.20 | 8.30 | 8.15 | 8.22 | 8.28 | 7.72 |
| ILMN_1720322 | PTS | 1.09 | 2.68 | 2.55 | 5.27 | 8.65 | 8.57 | 8.44 | 8.45 | 8.23 |
| ILMN_1795158 | FGR | 1.09 | 0.49 | 2.33 | -3.61 | 7.67 | 7.44 | 7.56 | 7.17 | 8.43 |
| ILMN_1755235 | XPO6 | 1.09 | -1.99 | -0.08 | -4.14 | 9.00 | 8.90 | 9.17 | 9.00 | 9.36 |
| ILMN_1715169 | HLA-DRB1 | 1.09 | -0.18 | 1.21 | 3.36 | 7.65 | 7.52 | 7.67 | 7.51 | 7.27 |
| ILMN_2129161 | LRRC32 | 1.09 | 1.23 | -0.34 | 3.75 | 7.64 | 7.48 | 7.46 | 7.69 | 7.09 |
| ILMN_3224926 | RBM47 | 1.10 | 3.12 | 3.82 | -0.06 | 7.59 | 7.46 | 7.22 | 7.14 | 7.60 |
| ILMN_3249240 | C4orf46 | 1.10 | -0.96 | -0.82 | 4.46 | 9.17 | 9.07 | 9.27 | 9.25 | 8.74 |
| ILMN_1712197 | KCNMB3 | 1.10 | -0.06 | 2.26 | 2.94 | 8.52 | 8.39 | 8.52 | 8.26 | 8.19 |
| ILMN_1733176 | LIMS1 | 1.10 | 1.91 | 1.08 | 4.41 | 9.41 | 9.25 | 9.13 | 9.25 | 8.76 |
| ILMN_1670561 | TCEAL8 | 1.10 | 1.84 | 1.53 | 3.34 | 7.28 | 7.16 | 7.08 | 7.11 | 6.92 |
| ILMN_3217276 | LOC644517 | 1.10 | 1.81 | 2.73 | 2.73 | 7.36 | 7.28 | 7.23 | 7.16 | 7.16 |
| ILMN_2110167 | POLR1E | 1.10 | 1.49 | 2.31 | 3.12 | 10.14 | 9.97 | 9.91 | 9.78 | 9.65 |
| ILMN_1815719 | PLCG2 | 1.10 | 0.33 | 0.23 | 5.34 | 11.83 | 11.69 | 11.79 | 11.80 | 11.12 |
| ILMN_1778599 | SP140 | 1.11 | 1.96 | 2.77 | 3.70 | 9.12 | 8.66 | 8.30 | 7.95 | 7.56 |
| ILMN_1665655 | CTDSPL2 | 1.11 | 0.36 | 1.50 | 3.62 | 8.73 | 8.61 | 8.69 | 8.57 | 8.36 |
| ILMN_1688279 | PVRIG | 1.11 | 0.88 | 2.89 | 4.65 | 8.94 | 8.79 | 8.82 | 8.56 | 8.34 |
| ILMN_1737651 | CDC40 | 1.11 | 3.60 | 4.03 | 3.07 | 9.04 | 8.95 | 8.73 | 8.69 | 8.78 |
| ILMN_1781039 | VPS26 | 1.11 | 0.76 | 0.50 | 3.10 | 8.65 | 8.49 | 8.54 | 8.58 | 8.21 |
| ILMN_2328378 | OSBPL3 | 1.11 | -1.16 | -1.60 | -2.88 | 8.61 | 8.45 | 8.78 | 8.84 | 9.03 |
| ILMN_1771139 | FBXO31 | 1.11 | 0.04 | 0.59 | 4.20 | 8.83 | 8.73 | 8.83 | 8.78 | 8.46 |
| ILMN_1811702 | GRN | 1.12 | 2.59 | 3.44 | 3.46 | 9.04 | 8.84 | 8.58 | 8.43 | 8.42 |
| ILMN_1776582 | PDK3 | 1.12 | 1.08 | 2.46 | 2.73 | 7.64 | 7.54 | 7.54 | 7.42 | 7.40 |
| ILMN_3200921 | LOC642590 | 1.12 | 2.40 | 2.56 | 3.87 | 9.63 | 9.47 | 9.29 | 9.27 | 9.08 |
| ILMN_3228269 | LOC730236 | 1.12 | -0.82 | 0.79 | 4.83 | 7.64 | 7.51 | 7.73 | 7.55 | 7.09 |
| ILMN_3247998 | STAP1 | 1.12 | 1.63 | 1.54 | 7.25 | 9.24 | 9.08 | 9.00 | 9.02 | 8.20 |
| ILMN_1789508 | GTF3C3 | 1.12 | 2.41 | 4.77 | 1.95 | 9.71 | 9.58 | 9.42 | 9.13 | 9.48 |
| ILMN_1742074 | C8orf76 | 1.12 | 3.17 | 4.02 | 1.38 | 10.26 | 10.13 | 9.88 | 9.78 | 10.10 |
| ILMN_2109536 | FLJ32679 | 1.12 | 2.12 | 1.30 | 2.90 | 6.79 | 6.71 | 6.63 | 6.69 | 6.57 |
| ILMN_1656463 | C11orf73 | 1.13 | 1.63 | 1.08 | 4.20 | 8.86 | 8.72 | 8.66 | 8.73 | 8.33 |
| ILMN_2207328 | C18orf10 | 1.13 | 0.49 | -0.21 | 3.42 | 9.20 | 9.08 | 9.15 | 9.23 | 8.83 |
| ILMN_3236877 | LOC729362 | 1.13 | 2.19 | 0.98 | 2.76 | 7.85 | 7.75 | 7.65 | 7.76 | 7.60 |
| ILMN_1700515 | C17orf58 | 1.13 | 2.93 | 2.45 | 3.51 | 7.87 | 7.77 | 7.60 | 7.65 | 7.55 |
| ILMN_2119421 | LOC143543 | 1.13 | 2.29 | 1.33 | 2.95 | 9.35 | 9.16 | 8.96 | 9.12 | 8.85 |
| ILMN_2357193 | DDX59 | 1.14 | 1.59 | 0.82 | 2.82 | 7.16 | 7.08 | 7.05 | 7.10 | 6.96 |
| ILMN_1681757 | FAM80B | 1.14 | 0.03 | 1.24 | -3.67 | 6.99 | 6.85 | 6.99 | 6.83 | 7.47 |
| ILMN_1712027 | RSBN1L | 1.14 | 1.42 | 1.57 | 4.07 | 9.50 | 9.38 | 9.35 | 9.33 | 9.06 |
| ILMN_1782729 | CLECL1 | 1.14 | 4.69 | 3.42 | 4.08 | 11.27 | 11.12 | 10.66 | 10.82 | 10.74 |
| ILMN_2044293 | KBTBD7 | 1.14 | 0.00 | -0.54 | 3.71 | 8.84 | 8.72 | 8.84 | 8.89 | 8.45 |
| ILMN_3305942 | LOC729970 | 1.14 | 0.62 | 0.86 | 3.10 | 6.86 | 6.78 | 6.81 | 6.80 | 6.64 |
| ILMN_1661051 | WBP11 | 1.15 | 0.72 | 0.81 | 2.97 | 6.99 | 6.90 | 6.93 | 6.93 | 6.76 |
| ILMN_1730516 | TMEM133 | 1.15 | -1.01 | -1.67 | 3.53 | 7.35 | 7.26 | 7.43 | 7.48 | 7.08 |
| ILMN_1854833 |  | 1.15 | -0.44 | -1.01 | -2.91 | 8.32 | 8.20 | 8.36 | 8.42 | 8.60 |
| ILMN_1679587 | LOC651202 | 1.15 | 1.11 | 1.27 | 3.90 | 12.78 | 12.59 | 12.60 | 12.57 | 12.14 |
| ILMN_1730416 | CYCS | 1.15 | -0.47 | -0.73 | -3.19 | 8.42 | 8.28 | 8.48 | 8.52 | 8.83 |
| ILMN_2081883 | IQCK | 1.15 | 1.88 | 2.63 | 4.60 | 8.53 | 8.40 | 8.32 | 8.24 | 8.02 |
| ILMN_2326509 | CASP1 | 1.15 | 2.24 | 3.83 | 6.33 | 10.16 | 10.03 | 9.90 | 9.72 | 9.43 |
| ILMN_1775448 | PFN2 | 1.15 | -0.89 | 0.35 | 2.77 | 7.71 | 7.53 | 7.84 | 7.65 | 7.29 |
| ILMN_1678957 | WDR55 | 1.15 | 1.95 | 2.72 | 3.07 | 8.38 | 8.24 | 8.15 | 8.05 | 8.01 |
| ILMN_2181241 | LOC649946 | 1.15 | 3.06 | 4.13 | 5.39 | 11.90 | 11.76 | 11.52 | 11.39 | 11.23 |
| ILMN_3302456 | LOC730029 | 1.15 | 1.31 | 1.19 | 3.45 | 10.42 | 10.18 | 10.14 | 10.17 | 9.69 |
| ILMN_1713178 | FAM116A | 1.15 | -1.94 | 0.04 | 3.28 | 9.67 | 9.56 | 9.84 | 9.66 | 9.37 |
| ILMN_1751431 | WIBG | 1.15 | 0.85 | 0.58 | -3.01 | 9.26 | 9.13 | 9.17 | 9.20 | 9.59 |
| ILMN_2383693 | UPF2 | 1.15 | 2.31 | 2.52 | 3.83 | 11.28 | 11.16 | 11.03 | 11.01 | 10.86 |
| ILMN_1856480 |  | 1.16 | -1.64 | -2.08 | 4.93 | 8.19 | 8.04 | 8.40 | 8.46 | 7.55 |
| ILMN_1747192 | RNF125 | 1.16 | 0.53 | 1.47 | 3.12 | 7.34 | 7.23 | 7.29 | 7.20 | 7.05 |
| ILMN_3232573 | MSL3 | 1.16 | 0.52 | 0.52 | 3.92 | 7.74 | 7.64 | 7.70 | 7.70 | 7.39 |
| ILMN_1778691 | TIA1 | 1.16 | 1.27 | -0.27 | 3.03 | 9.19 | 9.02 | 9.00 | 9.23 | 8.75 |
| ILMN_1789074 | HSPA1A | 1.16 | 3.58 | 1.35 | 5.72 | 8.75 | 8.40 | 7.65 | 8.34 | 7.00 |
| ILMN_1693830 | LACTB | 1.16 | 1.86 | 1.35 | 2.88 | 6.91 | 6.83 | 6.78 | 6.81 | 6.70 |
| ILMN_1725169 | INTS12 | 1.16 | 2.43 | 0.15 | 3.59 | 9.72 | 9.51 | 9.28 | 9.70 | 9.06 |
| ILMN_3234142 | LOC728855 | 1.16 | -0.58 | -0.12 | 2.78 | 8.23 | 8.13 | 8.27 | 8.24 | 8.00 |
| ILMN_2393450 | C14orf173 | 1.16 | 0.88 | 0.00 | 2.83 | 10.25 | 10.09 | 10.13 | 10.25 | 9.85 |
| ILMN_1716869 | GPM6A | 1.16 | 1.13 | 1.78 | 3.73 | 9.88 | 9.57 | 9.58 | 9.40 | 8.87 |
| ILMN_1684887 | SAMSN1 | 1.17 | 2.27 | 1.69 | 4.51 | 7.34 | 7.22 | 7.11 | 7.17 | 6.88 |
| ILMN_2057826 | PHF3 | 1.17 | 0.78 | 1.52 | 2.80 | 7.63 | 7.53 | 7.56 | 7.50 | 7.39 |
| ILMN_1713764 | LOC440928 | 1.17 | -0.77 | 1.66 | 4.88 | 9.86 | 9.68 | 9.98 | 9.60 | 9.10 |
| ILMN_1732923 | SIPA1L2 | 1.17 | 1.82 | 3.07 | 4.00 | 9.23 | 8.90 | 8.72 | 8.36 | 8.10 |
| ILMN_1711462 | MNS1 | 1.17 | 0.75 | 1.82 | 4.31 | 7.81 | 7.59 | 7.67 | 7.47 | 7.01 |
| ILMN_2147471 | PI4K2B | 1.18 | -0.67 | -0.17 | 5.75 | 7.73 | 7.64 | 7.78 | 7.74 | 7.27 |
| ILMN_1714349 | GLCE | 1.18 | -0.09 | 0.28 | 5.79 | 7.65 | 7.56 | 7.65 | 7.63 | 7.24 |
| ILMN_2400297 | MAPK9 | 1.18 | 1.72 | 1.61 | 3.79 | 7.81 | 7.68 | 7.61 | 7.63 | 7.37 |
| ILMN_1717935 | SPTA1 | 1.18 | -0.64 | 0.00 | 3.22 | 7.15 | 7.03 | 7.22 | 7.15 | 6.82 |
| ILMN_1801833 | ARHGAP24 | 1.18 | 1.71 | 3.10 | 7.01 | 7.96 | 7.86 | 7.82 | 7.71 | 7.39 |
| ILMN_2131523 | SACS | 1.18 | 1.72 | -0.03 | 3.51 | 9.42 | 9.30 | 9.25 | 9.42 | 9.07 |
| ILMN_1757794 | BRWD3 | 1.18 | 2.94 | 1.96 | 4.93 | 7.29 | 7.19 | 7.05 | 7.13 | 6.88 |
| ILMN_1699071 | C21orf7 | 1.18 | -0.04 | -0.90 | 4.31 | 7.61 | 7.50 | 7.62 | 7.70 | 7.21 |
| ILMN_1762972 | CHD9 | 1.19 | 0.39 | 1.78 | 4.73 | 10.48 | 10.25 | 10.41 | 10.13 | 9.55 |
| ILMN_1736068 | CNOT8 | 1.19 | 2.04 | 0.51 | 5.52 | 8.63 | 8.54 | 8.47 | 8.59 | 8.20 |
| ILMN_1729450 | C16orf62 | 1.19 | 1.53 | 2.30 | 3.15 | 7.04 | 6.95 | 6.92 | 6.86 | 6.80 |
| ILMN_1753507 | FRMPD3 | 1.19 | 1.40 | 0.60 | 3.41 | 7.43 | 7.31 | 7.29 | 7.37 | 7.10 |
| ILMN_1655827 | COPS2 | 1.19 | 2.76 | 2.53 | 4.83 | 8.71 | 8.57 | 8.39 | 8.41 | 8.14 |
| ILMN_3246560 | EMB | 1.20 | 1.54 | 1.74 | 3.56 | 7.26 | 7.14 | 7.11 | 7.09 | 6.91 |
| ILMN_1745282 | RAGE | 1.20 | 0.57 | 4.85 | 4.58 | 8.00 | 7.87 | 7.94 | 7.46 | 7.49 |
| ILMN_1672759 | CCDC109A | 1.20 | 0.09 | 0.51 | 3.55 | 7.90 | 7.81 | 7.89 | 7.86 | 7.64 |
| ILMN_1744611 | WDSOF1 | 1.20 | 3.28 | 2.82 | 3.52 | 9.03 | 8.88 | 8.62 | 8.68 | 8.59 |
| ILMN_1727738 | RAB33B | 1.20 | 1.27 | 1.09 | 6.07 | 9.07 | 8.89 | 8.88 | 8.91 | 8.18 |
| ILMN_2192683 | DHX37 | 1.20 | -1.31 | -0.64 | -2.82 | 9.83 | 9.72 | 9.94 | 9.88 | 10.08 |
| ILMN_2373073 | LIG4 | 1.20 | 0.96 | 0.33 | 3.09 | 8.00 | 7.86 | 7.89 | 7.96 | 7.63 |
| ILMN_1698953 | BTN1A1 | 1.20 | 1.62 | 1.16 | 2.78 | 6.93 | 6.83 | 6.80 | 6.84 | 6.71 |
| ILMN_1791217 | FAM40A | 1.20 | 0.26 | 1.28 | -5.90 | 7.62 | 7.51 | 7.60 | 7.50 | 8.16 |
| ILMN_3306482 | LOC730107 | 1.20 | 1.32 | 2.17 | 5.50 | 10.67 | 10.50 | 10.49 | 10.37 | 9.90 |
| ILMN_1690392 | COMMD3 | 1.20 | -0.29 | 0.95 | 4.79 | 11.64 | 11.51 | 11.67 | 11.54 | 11.13 |
| ILMN_1692116 | LOC650321 | 1.21 | 2.75 | 4.26 | 3.20 | 7.91 | 7.77 | 7.60 | 7.44 | 7.55 |
| ILMN_1767068 | SMAD6 | 1.21 | -0.32 | 0.00 | -3.46 | 6.83 | 6.73 | 6.86 | 6.83 | 7.12 |
| ILMN_3246544 | OSTCL | 1.21 | 1.00 | 1.52 | 3.99 | 7.88 | 7.72 | 7.75 | 7.68 | 7.37 |
| ILMN_1700204 | ZMYND8 | 1.21 | -0.47 | 1.51 | 3.19 | 6.89 | 6.80 | 6.93 | 6.78 | 6.65 |
| ILMN_1697000 | KIAA0258 | 1.21 | 3.14 | 3.36 | 3.69 | 6.97 | 6.90 | 6.78 | 6.77 | 6.75 |
| ILMN_1713688 | DHX32 | 1.21 | -0.16 | 1.87 | 5.17 | 8.11 | 7.99 | 8.13 | 7.92 | 7.58 |
| ILMN_1724658 | BNIP3 | 1.21 | 2.67 | 2.85 | 3.24 | 10.82 | 10.63 | 10.39 | 10.36 | 10.30 |
| ILMN_1672389 | CRYZ | 1.21 | 2.46 | 3.05 | 6.73 | 9.38 | 9.28 | 9.17 | 9.12 | 8.81 |
| ILMN_1794968 | LRRC28 | 1.21 | 2.09 | 1.21 | 5.06 | 8.14 | 8.04 | 7.97 | 8.04 | 7.73 |
| ILMN_1715351 | SCYE1 | 1.21 | 0.31 | 0.85 | 3.60 | 6.97 | 6.88 | 6.95 | 6.91 | 6.70 |
| ILMN_1815479 | NOP10 | 1.22 | 0.78 | 1.02 | 5.35 | 11.83 | 11.75 | 11.78 | 11.76 | 11.46 |
| ILMN_1795243 | LOC220433 | 1.22 | 2.27 | 1.92 | 3.89 | 12.89 | 12.74 | 12.61 | 12.66 | 12.41 |
| ILMN_1800975 | PSME3 | 1.22 | 1.95 | 3.01 | 2.97 | 9.42 | 9.24 | 9.13 | 8.97 | 8.98 |
| ILMN_1722239 | TIMM8A | 1.22 | 2.58 | 3.01 | 4.56 | 9.13 | 8.98 | 8.82 | 8.77 | 8.59 |
| ILMN_3282174 | LOC646688 | 1.22 | 1.47 | 1.93 | 4.11 | 12.75 | 12.60 | 12.57 | 12.52 | 12.26 |
| ILMN_1803073 | DNAJC12 | 1.22 | 0.84 | 2.83 | 4.89 | 8.14 | 7.93 | 7.99 | 7.65 | 7.29 |
| ILMN_1738333 | CCDC50 | 1.22 | 2.24 | 1.89 | 3.76 | 7.35 | 7.21 | 7.09 | 7.13 | 6.92 |
| ILMN_1780132 | PELI2 | 1.22 | 0.31 | 2.67 | 5.84 | 9.30 | 9.16 | 9.27 | 8.98 | 8.60 |
| ILMN_1794560 | TMEM93 | 1.23 | 1.37 | 2.11 | 2.74 | 7.20 | 7.12 | 7.11 | 7.06 | 7.02 |
| ILMN_1701216 | BANP | 1.23 | 0.70 | -1.84 | 5.47 | 8.13 | 8.04 | 8.08 | 8.27 | 7.71 |
| ILMN_1653266 | DNAJB14 | 1.23 | -0.69 | 0.09 | 2.92 | 8.16 | 8.03 | 8.23 | 8.15 | 7.85 |
| ILMN_1738027 | BRCA1 | 1.23 | 1.33 | 3.37 | 3.17 | 7.98 | 7.86 | 7.85 | 7.65 | 7.67 |
| ILMN_1853824 | MGAT3 | 1.23 | 0.53 | 0.28 | -3.01 | 7.16 | 7.00 | 7.09 | 7.12 | 7.56 |
| ILMN_1814120 | PECR | 1.23 | 4.29 | 3.09 | 3.16 | 7.89 | 7.77 | 7.48 | 7.59 | 7.59 |
| ILMN_2367384 | EPHB2 | 1.23 | 1.82 | 1.64 | 3.55 | 7.12 | 7.03 | 6.99 | 7.00 | 6.86 |
| ILMN_1764166 | BCKDHB | 1.23 | 0.00 | -0.87 | 2.83 | 7.41 | 7.33 | 7.41 | 7.47 | 7.23 |
| ILMN_3247783 | MGC21881 | 1.24 | 0.66 | 2.61 | 6.98 | 7.51 | 7.41 | 7.46 | 7.31 | 6.98 |
| ILMN_1776842 | DKFZp451A211 | 1.24 | -1.11 | -2.15 | 6.39 | 9.36 | 9.18 | 9.53 | 9.69 | 8.40 |
| ILMN_2291644 | SIRT5 | 1.24 | -0.45 | 0.50 | -3.08 | 6.99 | 6.90 | 7.02 | 6.95 | 7.19 |
| ILMN_1755115 | RPL23 | 1.24 | 1.86 | 2.36 | 3.20 | 13.10 | 12.92 | 12.84 | 12.77 | 12.65 |
| ILMN_1737110 | LOC651957 | 1.24 | 2.26 | 2.72 | 3.01 | 7.96 | 7.65 | 7.40 | 7.28 | 7.21 |
| ILMN_1674297 | HCFC2 | 1.25 | 2.66 | 2.34 | 2.93 | 7.43 | 7.33 | 7.21 | 7.23 | 7.18 |
| ILMN_2413264 | SOCS4 | 1.25 | 1.09 | -0.13 | 2.99 | 7.45 | 7.32 | 7.34 | 7.46 | 7.15 |
| ILMN_1667577 | LCMT2 | 1.25 | 3.49 | 4.02 | 7.65 | 9.28 | 9.17 | 8.98 | 8.93 | 8.61 |
| ILMN_1739161 | PPAP2A | 1.25 | 1.75 | 2.21 | 4.34 | 6.93 | 6.83 | 6.79 | 6.75 | 6.58 |
| ILMN_1799516 | DNAJC9 | 1.25 | 1.18 | 2.75 | 4.73 | 10.99 | 10.87 | 10.88 | 10.73 | 10.54 |
| ILMN_2392546 | PAICS | 1.25 | 0.80 | 1.80 | 4.96 | 12.15 | 12.01 | 12.06 | 11.94 | 11.57 |
| ILMN_1671902 | THUMPD3 | 1.26 | 1.17 | 1.13 | 2.76 | 7.26 | 7.16 | 7.17 | 7.17 | 7.04 |
| ILMN_1754531 | AP4E1 | 1.26 | 0.08 | 0.49 | 3.49 | 8.52 | 8.41 | 8.51 | 8.48 | 8.23 |
| ILMN_1731418 | SP110 | 1.26 | 2.41 | 3.22 | 4.19 | 10.06 | 9.42 | 8.83 | 8.42 | 7.92 |
| ILMN_2098616 | C5orf39 | 1.26 | 1.08 | -0.38 | 3.10 | 8.68 | 8.51 | 8.53 | 8.73 | 8.27 |
| ILMN_1704785 | C10orf125 | 1.26 | 0.39 | 1.23 | 3.96 | 8.01 | 7.89 | 7.98 | 7.90 | 7.64 |
| ILMN_1684321 | CYB5B | 1.26 | 1.40 | 0.36 | 7.56 | 11.74 | 11.63 | 11.61 | 11.71 | 11.04 |
| ILMN_3193623 | LOC100129657 | 1.26 | 1.05 | 0.83 | 2.94 | 11.80 | 11.60 | 11.63 | 11.67 | 11.33 |
| ILMN_1797668 | INSM1 | 1.26 | 0.51 | 1.37 | 5.04 | 9.74 | 9.54 | 9.66 | 9.52 | 8.94 |
| ILMN_1807945 | ANP32A | 1.27 | 1.51 | 1.75 | 4.85 | 8.47 | 8.32 | 8.30 | 8.27 | 7.92 |
| ILMN_2167011 | ECHDC1 | 1.27 | 1.71 | 1.71 | 3.33 | 8.32 | 8.16 | 8.11 | 8.11 | 7.91 |
| ILMN_2057399 | ZBTB8OS | 1.27 | 2.61 | 4.19 | 1.27 | 8.40 | 8.25 | 8.09 | 7.90 | 8.25 |
| ILMN_1680239 | NUDT9 | 1.28 | 1.43 | 1.59 | 2.80 | 8.74 | 8.56 | 8.53 | 8.51 | 8.33 |
| ILMN_1730879 | CBY1 | 1.28 | 0.25 | 0.75 | 3.98 | 7.67 | 7.53 | 7.64 | 7.59 | 7.24 |
| ILMN_1674458 | KLHL7 | 1.28 | 0.26 | -1.02 | 3.37 | 8.19 | 8.02 | 8.15 | 8.32 | 7.75 |
| ILMN_1795298 | GPER | 1.28 | 0.72 | -0.40 | 2.75 | 11.16 | 10.92 | 11.03 | 11.24 | 10.64 |
| ILMN_1704637 | C4orf23 | 1.28 | 0.73 | 0.38 | 3.20 | 6.93 | 6.83 | 6.87 | 6.90 | 6.68 |
| ILMN_1714384 | PCCA | 1.28 | 0.87 | -0.68 | 3.69 | 7.65 | 7.54 | 7.58 | 7.71 | 7.33 |
| ILMN_2207562 | C4orf16 | 1.28 | 1.00 | 0.81 | 5.13 | 7.35 | 7.26 | 7.28 | 7.30 | 6.99 |
| ILMN_2354478 | CYFIP2 | 1.28 | 1.31 | 0.58 | 3.20 | 9.81 | 9.63 | 9.63 | 9.73 | 9.37 |
| ILMN_3272436 | LOC100129386 | 1.28 | 1.42 | 1.67 | 4.06 | 7.29 | 7.16 | 7.15 | 7.13 | 6.90 |
| ILMN_1658995 | ACOT9 | 1.29 | 3.42 | 2.64 | 4.32 | 8.65 | 8.47 | 8.18 | 8.28 | 8.05 |
| ILMN_1695902 | ZFP1 | 1.29 | 1.58 | -0.66 | 7.84 | 7.74 | 7.63 | 7.61 | 7.79 | 7.11 |
| ILMN_1677122 | FAM168B | 1.29 | -2.02 | -1.68 | -4.10 | 8.46 | 8.32 | 8.68 | 8.65 | 8.91 |
| ILMN_1782609 | STAG2 | 1.29 | 0.92 | 0.33 | 3.83 | 10.16 | 10.04 | 10.08 | 10.13 | 9.81 |
| ILMN_1723706 | GRAMD4 | 1.29 | -1.44 | -4.06 | -2.23 | 7.58 | 7.41 | 7.77 | 8.13 | 7.88 |
| ILMN_1655644 | ZNF804A | 1.29 | 1.96 | 2.34 | 3.25 | 8.63 | 8.40 | 8.28 | 8.22 | 8.05 |
| ILMN_2402272 | TCEAL8 | 1.29 | 1.77 | 2.58 | 3.16 | 7.96 | 7.77 | 7.70 | 7.58 | 7.50 |
| ILMN_3291352 | LOC643778 | 1.29 | 0.14 | 0.97 | 5.17 | 7.30 | 7.20 | 7.29 | 7.23 | 6.92 |
| ILMN_3235013 | LOC730288 | 1.29 | 0.33 | 1.14 | 3.21 | 10.80 | 10.66 | 10.76 | 10.67 | 10.44 |
| ILMN_2385239 | PBRM1 | 1.29 | 0.67 | -0.04 | 2.86 | 7.23 | 7.12 | 7.18 | 7.24 | 6.99 |
| ILMN_1745811 | TDRD3 | 1.29 | 1.14 | 0.77 | 3.30 | 7.84 | 7.70 | 7.72 | 7.76 | 7.48 |
| ILMN_2184049 | COX7B | 1.30 | 2.95 | 2.14 | 3.40 | 11.76 | 11.53 | 11.24 | 11.38 | 11.16 |
| ILMN_3249419 | LOC728457 | 1.30 | 0.30 | 1.00 | 2.89 | 8.82 | 8.61 | 8.77 | 8.66 | 8.34 |
| ILMN_1695576 | MRPL24 | 1.30 | -0.04 | -0.04 | -3.71 | 10.60 | 10.49 | 10.61 | 10.61 | 10.92 |
| ILMN_3201221 | LOC341315 | 1.30 | 2.09 | 3.05 | 4.54 | 10.45 | 10.25 | 10.14 | 9.99 | 9.77 |
| ILMN_1855797 |  | 1.30 | 2.52 | 2.95 | 2.74 | 7.07 | 6.97 | 6.88 | 6.84 | 6.86 |
| ILMN_1670764 | USP25 | 1.31 | 1.72 | 2.46 | 3.24 | 7.14 | 7.06 | 7.03 | 6.99 | 6.94 |
| ILMN_2202481 | UBLCP1 | 1.31 | 1.22 | 4.22 | 5.68 | 10.25 | 10.10 | 10.11 | 9.78 | 9.61 |
| ILMN_1707062 | REV1 | 1.31 | -0.10 | 0.67 | 2.75 | 9.01 | 8.88 | 9.02 | 8.94 | 8.73 |
| ILMN_1652594 | ACTR5 | 1.31 | 0.95 | 0.88 | -3.47 | 8.04 | 7.91 | 7.95 | 7.95 | 8.40 |
| ILMN_1676799 | MFAP1 | 1.31 | 0.98 | 1.60 | 3.16 | 9.76 | 9.65 | 9.68 | 9.63 | 9.50 |
| ILMN_1792075 | CD22 | 1.31 | 0.00 | -0.14 | -3.76 | 7.26 | 7.14 | 7.26 | 7.28 | 7.63 |
| ILMN_2217630 | CDKL3 | 1.31 | 3.02 | 4.37 | 4.19 | 7.58 | 7.46 | 7.30 | 7.17 | 7.19 |
| ILMN_2379644 | CD74 | 1.31 | 1.08 | 1.31 | 2.93 | 12.73 | 12.52 | 12.56 | 12.52 | 12.27 |
| ILMN_1746664 | WSB2 | 1.32 | -1.69 | -1.11 | 2.99 | 8.01 | 7.83 | 8.25 | 8.17 | 7.59 |
| ILMN_3201975 | LOC392285 | 1.32 | 1.63 | 1.22 | 4.86 | 12.44 | 12.22 | 12.16 | 12.23 | 11.61 |
| ILMN_1729179 | SPATA5L1 | 1.32 | 1.90 | 0.79 | 3.24 | 9.58 | 9.40 | 9.33 | 9.47 | 9.15 |
| ILMN_1814022 | NR1H3 | 1.32 | 1.92 | 0.94 | 6.07 | 7.47 | 7.35 | 7.30 | 7.39 | 6.93 |
| ILMN_1702501 | RPS6KA2 | 1.32 | 0.11 | 1.11 | 3.61 | 7.12 | 7.00 | 7.11 | 7.02 | 6.79 |
| ILMN_1808374 | SNTB2 | 1.32 | 1.25 | -0.23 | 7.20 | 8.57 | 8.45 | 8.46 | 8.59 | 7.95 |
| ILMN_1804611 | C12orf23 | 1.32 | 0.84 | 2.25 | 3.13 | 7.54 | 7.43 | 7.47 | 7.35 | 7.28 |
| ILMN_1652379 | SUCLG2 | 1.33 | 0.42 | 0.94 | 3.85 | 9.69 | 9.56 | 9.65 | 9.60 | 9.32 |
| ILMN_1732782 | SCN2A | 1.33 | 2.12 | 1.56 | 3.59 | 9.03 | 8.66 | 8.44 | 8.60 | 8.03 |
| ILMN_3296002 | LOC440461 | 1.33 | 2.06 | 3.32 | 3.09 | 7.05 | 6.93 | 6.87 | 6.76 | 6.78 |
| ILMN_2124064 | REL | 1.34 | 2.89 | 1.52 | 3.93 | 7.25 | 7.10 | 6.93 | 7.08 | 6.82 |
| ILMN_1839750 | LOC729776 | 1.34 | 2.55 | 2.52 | 3.70 | 7.69 | 7.55 | 7.42 | 7.42 | 7.30 |
| ILMN_1669468 | KDM4D | 1.34 | 2.88 | 3.45 | 3.74 | 7.30 | 7.21 | 7.10 | 7.06 | 7.04 |
| ILMN_1710571 | PAPD5 | 1.34 | 1.41 | 1.38 | 4.07 | 9.11 | 8.99 | 8.98 | 8.98 | 8.73 |
| ILMN_3224907 | LOC728672 | 1.35 | 2.21 | 2.03 | 2.97 | 13.32 | 13.13 | 13.00 | 13.03 | 12.89 |
| ILMN_3243042 | LOC100134209 | 1.35 | 1.56 | 1.26 | -3.39 | 7.45 | 7.35 | 7.33 | 7.36 | 7.71 |
| ILMN_1754600 | FNBP1L | 1.35 | 0.11 | 1.29 | 4.97 | 7.62 | 7.45 | 7.61 | 7.46 | 7.00 |
| ILMN_1711919 | SCYL2 | 1.35 | 3.53 | 3.33 | 4.36 | 9.15 | 9.03 | 8.85 | 8.87 | 8.78 |
| ILMN_2112460 | MAD2L1 | 1.35 | 2.51 | 1.04 | 9.02 | 9.29 | 9.17 | 9.07 | 9.20 | 8.51 |
| ILMN_1660806 | CSRP2 | 1.35 | 2.11 | 0.13 | 2.79 | 11.93 | 11.72 | 11.61 | 11.91 | 11.50 |
| ILMN_1795706 | WNT6 | 1.35 | 1.39 | 2.96 | 2.79 | 6.92 | 6.81 | 6.81 | 6.68 | 6.70 |
| ILMN_1689989 | CDC42BPB | 1.35 | 0.97 | 1.95 | 3.30 | 8.90 | 8.65 | 8.72 | 8.54 | 8.29 |
| ILMN_1698470 | SYAP1 | 1.35 | 2.38 | 1.78 | 3.15 | 8.86 | 8.68 | 8.54 | 8.62 | 8.44 |
| ILMN_3242176 | UBR3 | 1.35 | 3.57 | 2.67 | 3.67 | 8.05 | 7.92 | 7.70 | 7.79 | 7.69 |
| ILMN_1682495 | FOXP1 | 1.35 | -1.18 | -0.17 | -3.80 | 7.82 | 7.72 | 7.91 | 7.84 | 8.11 |
| ILMN_2152828 | KIF16B | 1.36 | 1.31 | 1.36 | 3.13 | 7.62 | 7.51 | 7.52 | 7.51 | 7.37 |
| ILMN_2407235 | LOC402057 | 1.36 | 1.53 | 1.76 | 3.11 | 10.01 | 9.81 | 9.78 | 9.75 | 9.54 |
| ILMN_1714765 | LOC389599 | 1.36 | 0.81 | 1.39 | 3.93 | 8.28 | 8.15 | 8.20 | 8.15 | 7.90 |
| ILMN_2242937 | ARSB | 1.36 | 0.62 | 0.35 | 3.33 | 7.46 | 7.31 | 7.39 | 7.42 | 7.09 |
| ILMN_2404112 | TRDMT1 | 1.36 | 0.31 | 0.80 | 2.75 | 7.52 | 7.39 | 7.49 | 7.44 | 7.25 |
| ILMN_2373566 | PJA1 | 1.36 | -0.46 | 0.93 | 4.11 | 7.62 | 7.47 | 7.67 | 7.52 | 7.17 |
| ILMN_2197577 | SPRR2C | 1.36 | 1.39 | 2.44 | -4.54 | 6.76 | 6.61 | 6.61 | 6.50 | 7.25 |
| ILMN_2086238 | SMYD4 | 1.36 | 1.70 | 2.57 | 2.78 | 8.32 | 8.15 | 8.11 | 8.00 | 7.97 |
| ILMN_1696503 | JRKL | 1.37 | 2.19 | 2.27 | 3.17 | 7.05 | 6.93 | 6.86 | 6.86 | 6.78 |
| ILMN_3231621 | LOC730235 | 1.37 | 0.52 | 1.34 | 3.53 | 7.75 | 7.60 | 7.69 | 7.60 | 7.36 |
| ILMN_1814726 | SCARB2 | 1.37 | 1.93 | 2.15 | 5.44 | 10.49 | 10.26 | 10.16 | 10.13 | 9.57 |
| ILMN_2214603 | PPP2R3C | 1.37 | 1.17 | 1.58 | 2.82 | 10.30 | 10.12 | 10.15 | 10.10 | 9.94 |
| ILMN_2052891 | PKD2 | 1.38 | 0.18 | 1.33 | 3.55 | 8.32 | 8.12 | 8.30 | 8.13 | 7.81 |
| ILMN_2321292 | WIPI2 | 1.38 | -2.38 | -1.98 | -2.99 | 8.12 | 7.98 | 8.35 | 8.31 | 8.41 |
| ILMN_2384056 | GPER | 1.38 | 0.54 | -0.22 | 3.52 | 11.63 | 11.36 | 11.52 | 11.67 | 10.94 |
| ILMN_1676846 | ABCE1 | 1.38 | 1.56 | 0.94 | 5.85 | 10.45 | 10.29 | 10.27 | 10.34 | 9.79 |
| ILMN_1690638 | LOC402677 | 1.39 | 0.97 | 1.59 | 3.76 | 9.66 | 9.43 | 9.50 | 9.39 | 9.03 |
| ILMN_1679185 | LEF1 | 1.39 | 0.95 | 2.93 | 2.84 | 8.22 | 7.72 | 7.88 | 7.16 | 7.19 |
| ILMN_2347807 | EXOC1 | 1.39 | 2.24 | 2.64 | 4.16 | 8.29 | 8.19 | 8.13 | 8.10 | 7.98 |
| ILMN_2404407 | ABAT | 1.39 | 1.09 | 1.93 | 5.12 | 7.11 | 6.98 | 7.01 | 6.92 | 6.61 |
| ILMN_1710075 | FAM89A | 1.39 | 1.07 | 0.77 | 5.00 | 9.45 | 9.21 | 9.26 | 9.32 | 8.57 |
| ILMN_3286813 | LOC391019 | 1.39 | 1.10 | 1.66 | 3.54 | 10.84 | 10.60 | 10.65 | 10.55 | 10.23 |
| ILMN_1692121 | USO1 | 1.39 | 2.40 | 1.99 | 3.10 | 9.21 | 9.07 | 8.96 | 9.00 | 8.89 |
| ILMN_1769633 | CTSO | 1.39 | 0.48 | 1.61 | 3.16 | 6.85 | 6.76 | 6.82 | 6.75 | 6.65 |
| ILMN_1766718 | LYSMD3 | 1.39 | 1.75 | 0.76 | 3.62 | 7.71 | 7.60 | 7.57 | 7.65 | 7.41 |
| ILMN_1660844 | INTS4 | 1.40 | 1.16 | 0.89 | 4.22 | 9.64 | 9.49 | 9.51 | 9.54 | 9.17 |
| ILMN_1737462 | OXR1 | 1.40 | 2.01 | 3.09 | 3.54 | 9.72 | 9.47 | 9.35 | 9.16 | 9.07 |
| ILMN_1724497 | ABI2 | 1.40 | 0.94 | 2.10 | 2.95 | 7.83 | 7.67 | 7.72 | 7.60 | 7.50 |
| ILMN_2371147 | BCL7C | 1.40 | 0.22 | 0.19 | -3.15 | 8.44 | 8.26 | 8.41 | 8.41 | 8.83 |
| ILMN_1857897 |  | 1.40 | 0.93 | 2.30 | 3.30 | 6.99 | 6.85 | 6.90 | 6.76 | 6.66 |
| ILMN_1892652 |  | 1.40 | 2.47 | 2.89 | 2.80 | 6.99 | 6.87 | 6.79 | 6.75 | 6.76 |
| ILMN_1670895 | ZNF207 | 1.41 | 3.79 | 3.01 | 3.67 | 11.02 | 10.90 | 10.70 | 10.76 | 10.71 |
| ILMN_1807719 | CTNS | 1.41 | 2.01 | 2.68 | 3.80 | 8.82 | 8.64 | 8.56 | 8.47 | 8.33 |
| ILMN_2124221 | CLLU1OS | 1.41 | 1.76 | 2.08 | 2.98 | 6.86 | 6.74 | 6.71 | 6.68 | 6.60 |
| ILMN_3250243 | FAM119A | 1.41 | 1.31 | 2.11 | 2.77 | 8.48 | 8.29 | 8.30 | 8.19 | 8.10 |
| ILMN_1703005 | IFP38 | 1.41 | 1.54 | 1.36 | 2.85 | 10.62 | 10.44 | 10.42 | 10.45 | 10.26 |
| ILMN_2096604 | NIP30 | 1.41 | -0.81 | -0.09 | 6.43 | 8.10 | 7.99 | 8.16 | 8.10 | 7.60 |
| ILMN_2154101 | UPRT | 1.42 | 1.13 | 1.50 | 4.59 | 8.08 | 7.91 | 7.95 | 7.90 | 7.55 |
| ILMN_1659463 | APAF1 | 1.42 | 0.54 | -0.79 | 3.12 | 8.45 | 8.30 | 8.39 | 8.53 | 8.12 |
| ILMN_1712019 | ANKRD17 | 1.42 | 0.35 | 1.27 | 3.19 | 8.71 | 8.52 | 8.66 | 8.54 | 8.29 |
| ILMN_1752046 | SH2B3 | 1.42 | 2.49 | 1.66 | 5.06 | 10.43 | 10.25 | 10.12 | 10.22 | 9.80 |
| ILMN_2078430 | MAGEB2 | 1.42 | 1.08 | 1.46 | 3.01 | 7.15 | 7.04 | 7.07 | 7.04 | 6.92 |
| ILMN_3239272 | SNORD71 | 1.42 | 0.60 | 1.39 | 2.88 | 6.92 | 6.79 | 6.87 | 6.80 | 6.66 |
| ILMN_1677840 | CNOT1 | 1.43 | 1.71 | 2.20 | 3.54 | 7.07 | 6.95 | 6.93 | 6.89 | 6.78 |
| ILMN_1883997 |  | 1.43 | 1.22 | 0.55 | 3.44 | 6.85 | 6.74 | 6.75 | 6.81 | 6.58 |
| ILMN_1660698 | GTPBP8 | 1.43 | 1.96 | 1.55 | 3.58 | 8.16 | 8.01 | 7.95 | 8.00 | 7.78 |
| ILMN_1660868 | ZBTB8OS | 1.43 | 1.96 | 3.01 | 3.28 | 6.98 | 6.89 | 6.85 | 6.79 | 6.77 |
| ILMN_1744949 | RHOBTB3 | 1.43 | 1.92 | 1.77 | 5.07 | 6.89 | 6.80 | 6.76 | 6.77 | 6.55 |
| ILMN_1708328 | RAB11FIP3 | 1.43 | -0.54 | 0.46 | 2.89 | 8.98 | 8.85 | 9.03 | 8.94 | 8.71 |
| ILMN_2410864 | RAB28 | 1.43 | 1.28 | 1.52 | 3.30 | 7.52 | 7.35 | 7.37 | 7.34 | 7.14 |
| ILMN_1701077 | LOC642897 | 1.43 | 3.95 | 4.12 | 2.44 | 7.63 | 7.52 | 7.32 | 7.30 | 7.44 |
| ILMN_1779264 | PSMG1 | 1.43 | 3.18 | 3.44 | 2.93 | 10.19 | 9.96 | 9.69 | 9.65 | 9.73 |
| ILMN_1786036 | GPATCH2 | 1.43 | 2.91 | 3.53 | 4.52 | 7.14 | 7.04 | 6.93 | 6.88 | 6.81 |
| ILMN_1808059 | BCAS4 | 1.43 | 1.50 | -0.19 | -3.47 | 9.17 | 8.94 | 8.93 | 9.20 | 9.73 |
| ILMN_3209676 | LOC644315 | 1.43 | 1.60 | 2.47 | 3.06 | 12.83 | 12.60 | 12.58 | 12.44 | 12.34 |
| ILMN_1754501 | LOC339804 | 1.44 | 3.97 | 3.18 | 4.49 | 8.43 | 8.20 | 7.79 | 7.92 | 7.71 |
| ILMN_1763461 | ALDH7A1 | 1.44 | 2.70 | 3.51 | 5.01 | 8.17 | 7.84 | 7.55 | 7.36 | 7.02 |
| ILMN_1757781 | SAP30L | 1.44 | 3.56 | 3.17 | 4.28 | 9.88 | 9.75 | 9.55 | 9.59 | 9.48 |
| ILMN_1664691 | DAPP1 | 1.44 | 2.40 | 2.16 | 4.21 | 7.83 | 7.61 | 7.47 | 7.50 | 7.19 |
| ILMN_1757084 | LOC642443 | 1.44 | 1.91 | 1.88 | 3.58 | 7.11 | 6.97 | 6.93 | 6.93 | 6.78 |
| ILMN_1763228 | MEF2D | 1.45 | -0.80 | 0.51 | -3.89 | 8.70 | 8.48 | 8.82 | 8.62 | 9.28 |
| ILMN_2128967 | C11orf1 | 1.45 | 3.13 | 3.26 | 4.63 | 10.86 | 10.51 | 10.10 | 10.07 | 9.74 |
| ILMN_1667825 | MLKL | 1.45 | 1.05 | 0.72 | 3.05 | 8.23 | 7.97 | 8.04 | 8.10 | 7.67 |
| ILMN_1810628 | KIAA0367 | 1.45 | 1.94 | 2.85 | 3.76 | 7.13 | 7.01 | 6.97 | 6.90 | 6.83 |
| ILMN_2106167 | RAP1GDS1 | 1.45 | 1.12 | 1.75 | 2.92 | 8.78 | 8.56 | 8.61 | 8.51 | 8.34 |
| ILMN_1793017 | DGKQ | 1.45 | 1.01 | 1.91 | 4.14 | 8.64 | 8.38 | 8.46 | 8.29 | 7.89 |
| ILMN_1679280 | LOC643997 | 1.45 | 1.92 | 1.82 | 3.89 | 10.97 | 10.72 | 10.64 | 10.66 | 10.30 |
| ILMN_1678290 | HMG20A | 1.45 | -0.22 | 0.36 | 3.06 | 8.09 | 7.95 | 8.11 | 8.05 | 7.81 |
| ILMN_2048607 | ANKRD9 | 1.46 | -1.17 | 0.44 | -3.89 | 7.59 | 7.44 | 7.71 | 7.54 | 8.00 |
| ILMN_2389429 | DCUN1D4 | 1.46 | 3.22 | 2.92 | 4.17 | 7.13 | 7.04 | 6.92 | 6.94 | 6.86 |
| ILMN_1730998 | TSPAN6 | 1.46 | -1.46 | 0.73 | 4.69 | 7.69 | 7.55 | 7.83 | 7.62 | 7.24 |
| ILMN_1735679 | DHX38 | 1.46 | -2.22 | 0.16 | 3.95 | 7.83 | 7.74 | 7.96 | 7.82 | 7.58 |
| ILMN_1673450 | DDN | 1.46 | -0.88 | -0.99 | -6.58 | 7.61 | 7.47 | 7.69 | 7.70 | 8.21 |
| ILMN_1699695 | TNFRSF21 | 1.46 | 1.31 | 3.33 | 5.11 | 9.42 | 9.19 | 9.22 | 8.90 | 8.62 |
| ILMN_2095273 | KIAA1407 | 1.46 | 1.17 | 0.70 | 6.96 | 9.08 | 8.92 | 8.95 | 9.00 | 8.29 |
| ILMN_1717099 | DSCR3 | 1.46 | 0.81 | 0.27 | 3.08 | 8.80 | 8.67 | 8.73 | 8.77 | 8.53 |
| ILMN_1689652 | RNMTL1 | 1.46 | 2.84 | 4.86 | 3.02 | 9.83 | 9.67 | 9.52 | 9.31 | 9.50 |
| ILMN_1859863 |  | 1.46 | 2.63 | 2.86 | 6.42 | 9.07 | 8.92 | 8.80 | 8.78 | 8.42 |
| ILMN_2172755 | DEPDC6 | 1.46 | 0.73 | 1.54 | 2.81 | 6.95 | 6.82 | 6.89 | 6.82 | 6.71 |
| ILMN_1773380 | UNC13D | 1.47 | -0.73 | -0.15 | -3.88 | 7.26 | 7.13 | 7.33 | 7.28 | 7.62 |
| ILMN_3199737 | LOC727865 | 1.47 | 0.89 | 1.62 | 3.45 | 11.72 | 11.56 | 11.62 | 11.54 | 11.34 |
| ILMN_2135991 | C16orf69 | 1.47 | 1.30 | 1.80 | 5.12 | 7.07 | 6.96 | 6.97 | 6.93 | 6.67 |
| ILMN_1689371 | GIMAP2 | 1.47 | 2.18 | 3.65 | 3.70 | 7.10 | 7.00 | 6.96 | 6.86 | 6.86 |
| ILMN_1692058 | NDN | 1.47 | 1.67 | 2.53 | 2.74 | 6.88 | 6.76 | 6.75 | 6.68 | 6.66 |
| ILMN_2228873 | STARD3NL | 1.47 | 0.81 | 0.21 | 2.77 | 7.87 | 7.73 | 7.79 | 7.85 | 7.61 |
| ILMN_1813455 | SP110 | 1.47 | 2.90 | 4.05 | 4.58 | 7.46 | 7.24 | 7.04 | 6.87 | 6.79 |
| ILMN_2268026 | C15orf44 | 1.47 | 1.51 | 2.34 | 3.81 | 7.96 | 7.83 | 7.82 | 7.75 | 7.61 |
| ILMN_1795463 | MORC4 | 1.48 | 1.30 | 1.69 | 3.65 | 6.99 | 6.88 | 6.89 | 6.86 | 6.71 |
| ILMN_1765409 | STAM | 1.48 | 0.00 | 0.80 | 4.75 | 8.26 | 8.13 | 8.26 | 8.19 | 7.84 |
| ILMN_1787345 | FKBP11 | 1.48 | 1.58 | 2.16 | -2.89 | 9.45 | 9.25 | 9.24 | 9.16 | 9.85 |
| ILMN_1759396 | NNT | 1.48 | 1.37 | 2.18 | 5.62 | 7.66 | 7.53 | 7.54 | 7.47 | 7.18 |
| ILMN_1690680 | SSX1 | 1.48 | 2.92 | 3.31 | 4.31 | 7.78 | 7.43 | 7.10 | 7.01 | 6.78 |
| ILMN_2400292 | MAPK9 | 1.49 | 1.49 | 3.40 | 3.02 | 8.35 | 8.25 | 8.25 | 8.12 | 8.14 |
| ILMN_2088124 | TMEM154 | 1.49 | 1.97 | 1.52 | 5.00 | 9.68 | 9.36 | 9.26 | 9.35 | 8.60 |
| ILMN_2386205 | C21orf33 | 1.49 | -0.06 | -0.22 | -3.11 | 8.29 | 8.11 | 8.29 | 8.31 | 8.66 |
| ILMN_2064898 | CCDC56 | 1.49 | 1.42 | 2.78 | 3.47 | 10.52 | 10.37 | 10.38 | 10.24 | 10.17 |
| ILMN_1727041 | EWSR1 | 1.49 | 1.13 | 1.49 | 3.14 | 11.39 | 11.24 | 11.28 | 11.24 | 11.08 |
| ILMN_2290118 | MEGF9 | 1.49 | 0.37 | 0.17 | 3.23 | 7.33 | 7.21 | 7.30 | 7.31 | 7.07 |
| ILMN_1802257 | PCTP | 1.49 | 2.71 | 0.95 | 4.44 | 8.06 | 7.92 | 7.80 | 7.97 | 7.63 |
| ILMN_1778956 | STS | 1.49 | 0.38 | 0.21 | 3.80 | 7.18 | 7.06 | 7.15 | 7.16 | 6.88 |
| ILMN_1738075 | CMIP | 1.49 | 0.04 | 0.19 | 4.46 | 10.67 | 10.44 | 10.66 | 10.64 | 9.98 |
| ILMN_1668498 | CWF19L2 | 1.49 | 1.05 | 0.71 | 4.01 | 9.44 | 9.30 | 9.34 | 9.37 | 9.05 |
| ILMN_1798827 | SRBD1 | 1.49 | 1.81 | 4.40 | 5.86 | 8.97 | 8.84 | 8.82 | 8.60 | 8.47 |
| ILMN_1716014 | LOC653232 | 1.49 | 1.11 | 1.56 | 2.79 | 12.22 | 11.97 | 12.04 | 11.96 | 11.76 |
| ILMN_1796813 | EED | 1.50 | -0.44 | 2.06 | 3.25 | 7.51 | 7.32 | 7.57 | 7.25 | 7.09 |
| ILMN_1761939 | TIPIN | 1.50 | 1.97 | 2.47 | 3.94 | 8.46 | 8.25 | 8.19 | 8.12 | 7.91 |
| ILMN_1740927 | LYRM4 | 1.50 | 0.72 | 0.31 | 2.86 | 7.99 | 7.80 | 7.90 | 7.95 | 7.62 |
| ILMN_1749070 | HLA-DPB1 | 1.50 | 3.28 | 1.57 | 7.24 | 8.83 | 8.68 | 8.51 | 8.68 | 8.12 |
| ILMN_1743770 | SLC25A14 | 1.50 | 1.48 | 1.96 | 3.73 | 8.28 | 8.04 | 8.04 | 7.97 | 7.68 |
| ILMN_2386008 | MPZL1 | 1.51 | 0.27 | -1.20 | -3.24 | 8.99 | 8.86 | 8.96 | 9.09 | 9.27 |
| ILMN_1720422 | G3BP2 | 1.51 | 0.63 | 1.34 | 2.79 | 8.60 | 8.35 | 8.49 | 8.38 | 8.14 |
| ILMN_1763104 | TRAF4 | 1.51 | 2.94 | 2.03 | 3.43 | 7.87 | 7.74 | 7.61 | 7.69 | 7.57 |
| ILMN_1724054 | ZNF519 | 1.51 | 0.81 | 1.43 | 3.05 | 7.07 | 6.95 | 7.00 | 6.95 | 6.82 |
| ILMN_1795139 | S100Z | 1.51 | 1.01 | 1.66 | 3.52 | 6.78 | 6.65 | 6.69 | 6.63 | 6.47 |
| ILMN_1733937 | MMD | 1.51 | 0.41 | -0.78 | 5.59 | 10.88 | 10.76 | 10.85 | 10.95 | 10.43 |
| ILMN_1749244 | LYRM1 | 1.51 | 1.66 | 2.44 | 4.38 | 11.45 | 11.28 | 11.27 | 11.18 | 10.97 |
| ILMN_1726873 | TPCN2 | 1.51 | -0.85 | 0.92 | -4.61 | 7.26 | 7.13 | 7.34 | 7.18 | 7.68 |
| ILMN_1683614 | ZPBP | 1.51 | 0.99 | 0.83 | 3.39 | 6.83 | 6.74 | 6.77 | 6.78 | 6.62 |
| ILMN_1772486 | ELF2 | 1.51 | 2.20 | 1.37 | 4.00 | 7.00 | 6.89 | 6.85 | 6.90 | 6.72 |
| ILMN_1681670 | SLC25A4 | 1.51 | 1.66 | 0.83 | 4.93 | 9.98 | 9.84 | 9.82 | 9.90 | 9.52 |
| ILMN_1806601 | GRSF1 | 1.51 | 2.80 | 3.08 | 2.94 | 9.84 | 9.66 | 9.51 | 9.47 | 9.49 |
| ILMN_1777106 | LRRC57 | 1.52 | 0.39 | 0.99 | 4.08 | 8.19 | 8.00 | 8.14 | 8.07 | 7.69 |
| ILMN_1747058 | TRAPPC2L | 1.52 | 1.08 | 2.63 | 6.83 | 11.26 | 10.95 | 11.04 | 10.72 | 9.86 |
| ILMN_1686562 | KIF13B | 1.52 | 0.38 | 1.27 | 2.95 | 7.50 | 7.38 | 7.47 | 7.40 | 7.27 |
| ILMN_1748546 | 41519 | 1.52 | 1.00 | 2.36 | -2.75 | 11.63 | 11.48 | 11.53 | 11.39 | 11.92 |
| ILMN_1678086 | CCDC74A | 1.52 | -0.24 | -0.21 | -4.80 | 7.37 | 7.16 | 7.41 | 7.40 | 8.05 |
| ILMN_1798874 | TMEM85 | 1.52 | 1.31 | 0.08 | 3.08 | 10.35 | 10.23 | 10.25 | 10.34 | 10.11 |
| ILMN_1707901 | DHRS7B | 1.52 | 3.21 | 2.12 | 3.88 | 7.41 | 7.26 | 7.09 | 7.20 | 7.02 |
| ILMN_1750051 | FLJ39827 | 1.52 | -0.42 | -0.65 | 4.73 | 8.78 | 8.65 | 8.82 | 8.84 | 8.37 |
| ILMN_1689234 | LOC643836 | 1.53 | 2.21 | 3.31 | 3.47 | 6.98 | 6.88 | 6.84 | 6.77 | 6.76 |
| ILMN_2213136 | LEF1 | 1.53 | 1.15 | 3.44 | 3.19 | 8.46 | 7.90 | 8.04 | 7.19 | 7.29 |
| ILMN_1747771 | LOC730744 | 1.53 | 0.53 | 1.00 | 3.35 | 11.02 | 10.84 | 10.96 | 10.90 | 10.64 |
| ILMN_1780560 | MYO18A | 1.53 | 0.90 | 0.33 | 3.05 | 7.50 | 7.33 | 7.40 | 7.46 | 7.16 |
| ILMN_1784292 | ANKMY2 | 1.53 | 1.91 | 1.69 | 2.87 | 8.24 | 8.08 | 8.04 | 8.06 | 7.94 |
| ILMN_1739810 | RAI1 | 1.53 | 1.37 | 2.11 | 2.90 | 7.14 | 7.02 | 7.03 | 6.97 | 6.91 |
| ILMN_1792144 | TCL1B | 1.54 | -0.36 | 1.41 | 2.98 | 7.24 | 7.07 | 7.28 | 7.09 | 6.91 |
| ILMN_3251445 | MED27 | 1.54 | 2.05 | 1.33 | 3.05 | 8.79 | 8.59 | 8.52 | 8.62 | 8.39 |
| ILMN_1683792 | LAP3 | 1.54 | 2.31 | 0.56 | 4.26 | 10.99 | 10.79 | 10.69 | 10.92 | 10.44 |
| ILMN_1667417 | RAB23 | 1.54 | 1.33 | 0.99 | 2.84 | 7.19 | 7.04 | 7.06 | 7.09 | 6.91 |
| ILMN_2367530 | ZNF280D | 1.54 | 2.33 | 1.63 | 3.13 | 7.06 | 6.95 | 6.89 | 6.94 | 6.83 |
| ILMN_2395474 | REV1 | 1.54 | -0.14 | 0.43 | 3.05 | 9.17 | 9.03 | 9.19 | 9.13 | 8.89 |
| ILMN_1682857 | NDUFAF2 | 1.54 | 2.81 | 3.19 | 3.19 | 10.42 | 10.18 | 9.97 | 9.91 | 9.91 |
| ILMN_3214256 | LOC128192 | 1.54 | 2.53 | 1.69 | 4.30 | 12.11 | 11.84 | 11.66 | 11.81 | 11.35 |
| ILMN_2221066 | VDAC2 | 1.55 | 0.64 | 1.96 | 3.24 | 7.45 | 7.34 | 7.40 | 7.31 | 7.21 |
| ILMN_2134855 | LOC728758 | 1.55 | 0.11 | -0.16 | 3.58 | 7.14 | 7.05 | 7.14 | 7.15 | 6.92 |
| ILMN_1737949 | GALNS | 1.55 | 2.76 | 3.17 | 5.42 | 7.70 | 7.52 | 7.38 | 7.34 | 7.08 |
| ILMN_3241510 | FBXO45 | 1.55 | 1.67 | -0.43 | 3.19 | 8.21 | 8.08 | 8.07 | 8.25 | 7.94 |
| ILMN_1752086 | C4orf41 | 1.55 | 1.67 | 1.75 | 2.83 | 9.03 | 8.84 | 8.83 | 8.82 | 8.69 |
| ILMN_1719756 | ZAP70 | 1.56 | 1.03 | 3.26 | 3.83 | 7.35 | 7.13 | 7.21 | 6.90 | 6.82 |
| ILMN_1671969 | UGP2 | 1.56 | 1.40 | 2.39 | 4.26 | 7.01 | 6.91 | 6.92 | 6.86 | 6.74 |
| ILMN_1770433 | PIK3CG | 1.56 | 2.22 | 1.13 | 3.99 | 7.68 | 7.53 | 7.47 | 7.57 | 7.31 |
| ILMN_1682812 | C21orf33 | 1.56 | 1.11 | 0.51 | -3.06 | 9.18 | 9.00 | 9.05 | 9.12 | 9.52 |
| ILMN_1771149 | MRPL19 | 1.56 | 2.00 | 1.72 | 3.33 | 10.18 | 9.95 | 9.89 | 9.93 | 9.69 |
| ILMN_1710209 | MFSD6 | 1.57 | 1.33 | 2.52 | 3.13 | 7.15 | 7.00 | 7.02 | 6.90 | 6.84 |
| ILMN_2330243 | NUDT1 | 1.57 | 0.43 | 0.70 | 2.78 | 11.69 | 11.49 | 11.63 | 11.60 | 11.34 |
| ILMN_1682197 | NFXL1 | 1.57 | 1.87 | 2.46 | 3.28 | 8.56 | 8.42 | 8.40 | 8.34 | 8.27 |
| ILMN_3234813 | LOC100130648 | 1.57 | 2.02 | 2.02 | 2.83 | 7.43 | 7.25 | 7.19 | 7.19 | 7.10 |
| ILMN_1664738 | C11orf54 | 1.57 | 1.83 | 1.52 | 3.62 | 7.41 | 7.29 | 7.27 | 7.30 | 7.14 |
| ILMN_1664488 | C10orf64 | 1.57 | 0.48 | 0.77 | 3.46 | 8.13 | 7.93 | 8.07 | 8.03 | 7.70 |
| ILMN_1765159 | ELMOD2 | 1.57 | 1.68 | 1.68 | 4.46 | 7.55 | 7.40 | 7.39 | 7.39 | 7.14 |
| ILMN_1788213 | FRAT2 | 1.58 | 0.92 | 1.51 | 5.38 | 9.55 | 9.40 | 9.46 | 9.41 | 9.03 |
| ILMN_3244893 | BAG2 | 1.58 | 2.24 | 2.64 | 5.74 | 8.33 | 8.13 | 8.04 | 7.99 | 7.58 |
| ILMN_1730631 | C2orf44 | 1.58 | 1.50 | 3.05 | 2.84 | 9.63 | 9.43 | 9.44 | 9.24 | 9.27 |
| ILMN_1793846 | BZW1 | 1.58 | 1.73 | 2.31 | 3.12 | 7.05 | 6.91 | 6.90 | 6.85 | 6.78 |
| ILMN_1779886 | TBC1D14 | 1.58 | 0.00 | -0.41 | 2.75 | 9.64 | 9.42 | 9.64 | 9.70 | 9.26 |
| ILMN_1761281 | LOC441019 | 1.58 | 1.17 | 2.50 | 2.91 | 6.96 | 6.86 | 6.89 | 6.80 | 6.77 |
| ILMN_1659095 | LOC644029 | 1.58 | 0.83 | 2.30 | 3.38 | 13.94 | 13.79 | 13.86 | 13.72 | 13.62 |
| ILMN_1695745 | DISP1 | 1.58 | 1.48 | 0.66 | 3.65 | 8.27 | 8.06 | 8.08 | 8.18 | 7.79 |
| ILMN_1671565 | RNASET2 | 1.58 | 0.87 | 0.47 | -3.23 | 10.69 | 10.52 | 10.60 | 10.64 | 11.04 |
| ILMN_2359306 | CAPSL | 1.58 | 1.88 | 2.21 | 3.02 | 6.87 | 6.72 | 6.70 | 6.67 | 6.59 |
| ILMN_1801403 | DCUN1D4 | 1.59 | 1.73 | 0.94 | 5.46 | 9.14 | 8.92 | 8.90 | 9.01 | 8.38 |
| ILMN_1651336 | MLYCD | 1.59 | 0.87 | 1.35 | 5.14 | 7.17 | 7.06 | 7.11 | 7.08 | 6.81 |
| ILMN_1657627 | CBFA2T3 | 1.59 | 1.67 | 0.53 | 3.06 | 7.36 | 7.17 | 7.16 | 7.29 | 6.99 |
| ILMN_1779171 | SGSM2 | 1.59 | 0.99 | 2.58 | 2.87 | 8.98 | 8.71 | 8.81 | 8.54 | 8.49 |
| ILMN_2352633 | ARHGAP24 | 1.59 | 1.90 | 3.00 | 4.24 | 7.16 | 7.04 | 7.02 | 6.93 | 6.84 |
| ILMN_1795762 | PLEK | 1.59 | 4.88 | 5.34 | 2.79 | 8.75 | 8.52 | 8.04 | 7.97 | 8.34 |
| ILMN_1756701 | DHRS11 | 1.59 | 1.44 | 3.41 | 2.94 | 7.91 | 7.74 | 7.76 | 7.55 | 7.60 |
| ILMN_1657746 | BPHL | 1.60 | 1.43 | 0.58 | 4.89 | 8.23 | 8.07 | 8.09 | 8.17 | 7.75 |
| ILMN_1732489 | SLC10A7 | 1.60 | 2.28 | 1.97 | 5.37 | 7.59 | 7.43 | 7.37 | 7.40 | 7.06 |
| ILMN_1813475 | HERC2 | 1.60 | -0.59 | 0.91 | 5.81 | 9.19 | 9.02 | 9.25 | 9.09 | 8.59 |
| ILMN_2393765 | IGLL1 | 1.60 | 0.74 | 0.99 | 4.14 | 13.31 | 13.06 | 13.20 | 13.16 | 12.66 |
| ILMN_1791792 | C12orf5 | 1.60 | 3.34 | 3.41 | 2.88 | 8.75 | 8.52 | 8.26 | 8.25 | 8.33 |
| ILMN_1799069 | LOC440280 | 1.60 | 0.42 | 0.87 | 2.99 | 8.24 | 8.01 | 8.18 | 8.12 | 7.82 |
| ILMN_3267760 | LOC100128936 | 1.60 | 1.38 | 1.85 | 3.61 | 12.28 | 12.11 | 12.13 | 12.08 | 11.89 |
| ILMN_1676215 | DLG2 | 1.61 | -1.28 | 1.25 | 6.60 | 7.32 | 7.17 | 7.44 | 7.20 | 6.70 |
| ILMN_1729563 | UGDH | 1.61 | 1.50 | 1.61 | 6.96 | 8.85 | 8.70 | 8.71 | 8.70 | 8.20 |
| ILMN_1679268 | PELI1 | 1.61 | 2.32 | 2.84 | 5.18 | 8.31 | 8.08 | 7.98 | 7.90 | 7.56 |
| ILMN_3240446 | ZNF286C | 1.61 | 1.05 | 2.46 | 3.28 | 7.06 | 6.89 | 6.95 | 6.81 | 6.72 |
| ILMN_2392356 | CTPS2 | 1.61 | 0.93 | 2.42 | 4.16 | 7.54 | 7.41 | 7.46 | 7.35 | 7.21 |
| ILMN_3184724 | C7orf58 | 1.61 | 1.50 | 2.39 | 3.84 | 6.99 | 6.89 | 6.90 | 6.85 | 6.76 |
| ILMN_2220845 | PRSS7 | 1.62 | 1.08 | -1.52 | 3.14 | 7.07 | 6.96 | 7.00 | 7.17 | 6.86 |
| ILMN_2403852 | CYTH1 | 1.62 | 1.41 | 1.18 | 4.29 | 9.44 | 9.23 | 9.26 | 9.29 | 8.89 |
| ILMN_1774336 | POLE2 | 1.62 | 1.23 | 2.37 | 2.97 | 9.10 | 8.92 | 8.97 | 8.84 | 8.77 |
| ILMN_2112988 | NCF1C | 1.62 | 0.12 | -1.02 | 4.24 | 7.88 | 7.65 | 7.87 | 8.03 | 7.27 |
| ILMN_2252160 | UBC | 1.62 | 2.48 | 2.07 | 3.33 | 6.83 | 6.71 | 6.65 | 6.68 | 6.58 |
| ILMN_3249667 | LOC100133678 | 1.62 | 3.94 | 3.18 | 5.02 | 12.44 | 12.26 | 12.00 | 12.09 | 11.88 |
| ILMN_3249235 | GIN1 | 1.62 | 1.96 | 2.54 | 3.20 | 7.38 | 7.25 | 7.22 | 7.18 | 7.12 |
| ILMN_1771651 | MON1B | 1.62 | -0.08 | 1.13 | 3.48 | 7.61 | 7.46 | 7.61 | 7.51 | 7.30 |
| ILMN_1748123 | KLHL14 | 1.63 | 1.06 | -0.80 | 2.90 | 9.83 | 9.65 | 9.71 | 9.92 | 9.50 |
| ILMN_1748018 | GORASP2 | 1.63 | 2.21 | 2.52 | 2.84 | 9.84 | 9.61 | 9.53 | 9.48 | 9.44 |
| ILMN_1728975 | SCO1 | 1.63 | 4.55 | 5.61 | 2.45 | 9.28 | 9.08 | 8.71 | 8.57 | 8.97 |
| ILMN_2401730 | C1GALT1C1 | 1.63 | 2.47 | 1.87 | 2.94 | 7.34 | 7.22 | 7.16 | 7.20 | 7.13 |
| ILMN_2404327 | SSX2 | 1.63 | 1.95 | 2.17 | 3.49 | 8.55 | 8.28 | 8.22 | 8.19 | 7.97 |
| ILMN_2330371 | TATDN3 | 1.64 | 1.84 | 0.95 | 3.92 | 9.71 | 9.52 | 9.50 | 9.60 | 9.27 |
| ILMN_1655429 | TNFAIP1 | 1.64 | 1.99 | 2.02 | 3.50 | 8.02 | 7.84 | 7.81 | 7.80 | 7.65 |
| ILMN_1721657 | RSU1 | 1.64 | 0.74 | -0.15 | 4.18 | 8.97 | 8.72 | 8.86 | 9.00 | 8.34 |
| ILMN_1683494 | TMEM154 | 1.65 | 1.78 | 1.11 | 4.20 | 8.69 | 8.33 | 8.30 | 8.45 | 7.78 |
| ILMN_1731412 | UBTD2 | 1.65 | 2.69 | 2.69 | 4.14 | 7.53 | 7.39 | 7.31 | 7.31 | 7.19 |
| ILMN_1659761 | SNX29 | 1.65 | -1.39 | -0.03 | 2.85 | 8.00 | 7.83 | 8.15 | 8.00 | 7.70 |
| ILMN_1807633 | HRSP12 | 1.65 | 3.91 | 4.41 | 4.33 | 8.61 | 8.40 | 8.12 | 8.06 | 8.07 |
| ILMN_2317348 | APTX | 1.65 | 3.16 | 4.20 | 5.68 | 9.68 | 9.52 | 9.37 | 9.26 | 9.12 |
| ILMN_2373556 | SPAST | 1.65 | 1.43 | 1.37 | 3.61 | 9.06 | 8.87 | 8.89 | 8.90 | 8.63 |
| ILMN_1812256 | GANC | 1.65 | 1.51 | 1.44 | 3.35 | 7.34 | 7.18 | 7.20 | 7.20 | 7.02 |
| ILMN_1745318 | CREM | 1.66 | 0.12 | 1.62 | 4.31 | 7.29 | 7.16 | 7.28 | 7.16 | 6.95 |
| ILMN_1652787 | PIK3AP1 | 1.66 | 0.60 | 0.35 | 3.38 | 10.49 | 10.25 | 10.40 | 10.44 | 10.00 |
| ILMN_1696031 | C15orf21 | 1.66 | 2.21 | 0.97 | 3.22 | 7.92 | 7.80 | 7.76 | 7.85 | 7.69 |
| ILMN_3283449 | LOC440991 | 1.66 | 1.00 | 2.37 | 4.78 | 11.60 | 11.41 | 11.48 | 11.32 | 11.04 |
| ILMN_1796339 | PLEKHA2 | 1.66 | 0.53 | 3.21 | 3.16 | 10.98 | 10.74 | 10.91 | 10.52 | 10.53 |
| ILMN_1686846 | AKAP12 | 1.66 | 1.03 | 1.03 | 2.88 | 6.94 | 6.79 | 6.85 | 6.85 | 6.68 |
| ILMN_1729075 | PTH2R | 1.66 | 2.83 | 3.06 | 4.74 | 7.58 | 7.29 | 7.09 | 7.05 | 6.75 |
| ILMN_2171640 | ZNF650 | 1.66 | 3.87 | 3.66 | 3.72 | 7.98 | 7.80 | 7.55 | 7.58 | 7.57 |
| ILMN_1672661 | SP110 | 1.66 | 2.49 | 3.04 | 4.13 | 8.96 | 8.30 | 7.97 | 7.75 | 7.31 |
| ILMN_1747744 | LHFPL2 | 1.66 | 2.30 | 3.76 | 3.86 | 8.55 | 8.09 | 7.91 | 7.51 | 7.48 |
| ILMN_1800530 | CENTG2 | 1.66 | 2.09 | 3.01 | 3.77 | 8.29 | 7.73 | 7.58 | 7.27 | 7.02 |
| ILMN_1703111 | BBS7 | 1.67 | 2.55 | 2.20 | 7.73 | 7.49 | 7.36 | 7.30 | 7.32 | 6.90 |
| ILMN_1807095 | MRPS36 | 1.67 | 2.31 | 4.25 | 3.01 | 7.92 | 7.74 | 7.67 | 7.45 | 7.59 |
| ILMN_1746135 | PHF23 | 1.67 | 2.37 | 2.15 | 2.97 | 8.73 | 8.58 | 8.52 | 8.54 | 8.46 |
| ILMN_3245179 | LOC100134071 | 1.67 | 0.99 | 1.02 | 2.77 | 6.95 | 6.81 | 6.87 | 6.86 | 6.71 |
| ILMN_1695945 | MEIS2 | 1.68 | 2.14 | 2.76 | 6.98 | 7.65 | 7.47 | 7.42 | 7.35 | 6.90 |
| ILMN_1717565 | LOC497190 | 1.68 | 0.71 | 1.75 | 3.28 | 6.92 | 6.77 | 6.86 | 6.77 | 6.63 |
| ILMN_2296950 | APOBEC3F | 1.68 | 2.24 | 3.65 | 3.16 | 7.46 | 7.29 | 7.23 | 7.09 | 7.14 |
| ILMN_2113074 | UFM1 | 1.68 | 1.06 | 1.00 | 3.71 | 9.18 | 9.01 | 9.07 | 9.08 | 8.80 |
| ILMN_1779374 | AMMECR1 | 1.68 | 0.55 | -2.35 | 4.76 | 9.18 | 9.00 | 9.12 | 9.44 | 8.66 |
| ILMN_1756439 | SCRN1 | 1.68 | -0.08 | -0.75 | 4.30 | 9.13 | 8.73 | 9.15 | 9.31 | 8.11 |
| ILMN_2368318 | FGR | 1.68 | 0.83 | 2.77 | -3.60 | 8.02 | 7.66 | 7.85 | 7.43 | 8.79 |
| ILMN_1690993 | NEUROG2 | 1.68 | 0.93 | 0.98 | -3.77 | 6.97 | 6.85 | 6.90 | 6.90 | 7.26 |
| ILMN_2202637 | CRY1 | 1.69 | 1.99 | 1.82 | 3.44 | 9.81 | 9.64 | 9.61 | 9.63 | 9.47 |
| ILMN_2110908 | MYC | 1.69 | -0.23 | 0.07 | 2.81 | 11.72 | 11.48 | 11.76 | 11.71 | 11.31 |
| ILMN_2111229 | BZRAP1 | 1.69 | -0.04 | 1.86 | -3.38 | 6.91 | 6.78 | 6.91 | 6.76 | 7.18 |
| ILMN_1695509 | PTPN12 | 1.69 | 2.06 | 1.26 | 4.93 | 7.82 | 7.62 | 7.58 | 7.67 | 7.25 |
| ILMN_1719158 | CTBP1 | 1.69 | -1.09 | 0.72 | 3.84 | 10.18 | 10.03 | 10.28 | 10.12 | 9.84 |
| ILMN_1724699 | ACAD8 | 1.70 | 1.04 | 1.75 | 2.79 | 7.00 | 6.90 | 6.94 | 6.89 | 6.83 |
| ILMN_1679460 | PPFIBP1 | 1.70 | 1.27 | 1.06 | 2.93 | 6.83 | 6.69 | 6.73 | 6.74 | 6.60 |
| ILMN_1782621 | RPS12 | 1.70 | 2.40 | 2.72 | 2.97 | 14.24 | 14.08 | 14.01 | 13.98 | 13.96 |
| ILMN_1774823 | RPL34 | 1.70 | 3.45 | 4.46 | 6.82 | 10.90 | 10.70 | 10.49 | 10.37 | 10.09 |
| ILMN_1696709 | TADA2A | 1.70 | 2.85 | 3.46 | 2.79 | 6.97 | 6.88 | 6.82 | 6.78 | 6.82 |
| ILMN_1652003 | GNG10 | 1.70 | 1.45 | 1.12 | 3.65 | 7.18 | 7.04 | 7.06 | 7.09 | 6.89 |
| ILMN_1666967 | BRP44L | 1.70 | 2.20 | 0.69 | 3.21 | 8.84 | 8.64 | 8.58 | 8.76 | 8.45 |
| ILMN_1785107 | NXT2 | 1.70 | 2.11 | 1.54 | 7.14 | 11.17 | 10.89 | 10.83 | 10.92 | 10.00 |
| ILMN_2157240 | MNS1 | 1.70 | 0.53 | 2.71 | 7.06 | 9.41 | 9.11 | 9.32 | 8.94 | 8.17 |
| ILMN_1759252 | ADD1 | 1.71 | 1.92 | 0.18 | 4.81 | 11.48 | 11.29 | 11.27 | 11.46 | 10.95 |
| ILMN_3290353 | LOC644790 | 1.71 | 1.10 | 1.27 | 3.46 | 12.12 | 11.89 | 11.97 | 11.95 | 11.65 |
| ILMN_1658071 | ATP1B1 | 1.71 | -0.12 | -0.47 | 4.35 | 6.95 | 6.81 | 6.96 | 6.99 | 6.58 |
| ILMN_1651254 | LPP | 1.71 | 1.33 | 0.54 | 2.80 | 8.97 | 8.67 | 8.73 | 8.87 | 8.47 |
| ILMN_1653026 | PLAC8 | 1.71 | 2.36 | 2.53 | 6.33 | 10.69 | 10.25 | 10.08 | 10.03 | 9.04 |
| ILMN_2232478 | APOBEC3G | 1.71 | 3.52 | 2.58 | 3.26 | 8.02 | 7.84 | 7.65 | 7.75 | 7.68 |
| ILMN_2365111 | MAP4K1 | 1.71 | -2.68 | -2.27 | -8.05 | 8.51 | 8.38 | 8.70 | 8.67 | 9.09 |
| ILMN_1708891 | SCFD2 | 1.71 | 0.65 | 0.33 | 3.06 | 7.18 | 7.04 | 7.12 | 7.15 | 6.93 |
| ILMN_1764186 | LOC146517 | 1.71 | 0.61 | 0.49 | 6.16 | 8.56 | 8.42 | 8.51 | 8.52 | 8.06 |
| ILMN_1804329 | TUSC2 | 1.71 | -0.35 | 0.91 | -3.03 | 7.56 | 7.45 | 7.59 | 7.50 | 7.76 |
| ILMN_3244583 | NCRNA00219 | 1.72 | 2.81 | 6.17 | 1.48 | 10.41 | 10.24 | 10.14 | 9.81 | 10.27 |
| ILMN_1653115 | ECH1 | 1.72 | 1.76 | 4.19 | -1.31 | 9.28 | 8.92 | 8.91 | 8.40 | 9.56 |
| ILMN_1746517 | KYNU | 1.72 | 3.79 | 1.41 | 6.14 | 9.70 | 9.39 | 9.02 | 9.45 | 8.59 |
| ILMN_1778985 | PRMT10 | 1.72 | 2.43 | 1.91 | 4.34 | 8.33 | 8.08 | 7.98 | 8.06 | 7.71 |
| ILMN_1721713 | EXOSC9 | 1.72 | 2.30 | 2.59 | 4.89 | 9.92 | 9.65 | 9.55 | 9.51 | 9.14 |
| ILMN_2116639 | TFDP2 | 1.72 | 1.72 | 3.35 | 3.15 | 7.35 | 7.11 | 7.11 | 6.89 | 6.92 |
| ILMN_1727526 | KIAA1407 | 1.72 | 1.76 | 0.64 | 5.65 | 8.19 | 8.01 | 8.00 | 8.12 | 7.60 |
| ILMN_1735499 | DCBLD2 | 1.73 | -0.37 | 1.12 | 4.47 | 7.46 | 7.29 | 7.50 | 7.35 | 7.02 |
| ILMN_1676406 | CTU2 | 1.73 | 0.92 | 0.96 | 3.27 | 7.14 | 6.98 | 7.05 | 7.05 | 6.84 |
| ILMN_1711888 | COBL | 1.73 | 1.46 | 3.61 | 4.12 | 7.21 | 7.02 | 7.05 | 6.81 | 6.75 |
| ILMN_2191720 | ZNF471 | 1.73 | -0.33 | 0.98 | 2.95 | 6.98 | 6.85 | 7.00 | 6.91 | 6.77 |
| ILMN_1757298 | BTBD7 | 1.73 | 2.49 | 2.27 | 3.12 | 7.61 | 7.42 | 7.34 | 7.37 | 7.28 |
| ILMN_1791226 | NXN | 1.74 | 0.36 | 2.51 | 3.05 | 7.55 | 7.35 | 7.51 | 7.27 | 7.21 |
| ILMN_1742224 | SLTM | 1.74 | 0.58 | 1.60 | 3.00 | 10.12 | 9.95 | 10.07 | 9.97 | 9.83 |
| ILMN_3239795 | SIK2 | 1.74 | 0.57 | 1.85 | 3.03 | 7.18 | 7.02 | 7.13 | 7.01 | 6.91 |
| ILMN_1656186 | SLC41A1 | 1.74 | 1.49 | 1.49 | -3.74 | 7.44 | 7.28 | 7.30 | 7.30 | 7.78 |
| ILMN_1680996 | ALOX5 | 1.75 | 1.77 | 1.82 | 2.93 | 11.70 | 11.47 | 11.47 | 11.46 | 11.32 |
| ILMN_1778876 | FAM179B | 1.75 | -0.30 | 1.27 | 3.17 | 8.45 | 8.25 | 8.48 | 8.30 | 8.09 |
| ILMN_3211463 | LOC644037 | 1.75 | 1.13 | 3.39 | 4.03 | 9.22 | 8.98 | 9.06 | 8.76 | 8.67 |
| ILMN_1795118 | SIDT1 | 1.75 | 1.47 | 0.21 | 2.90 | 7.52 | 7.36 | 7.38 | 7.50 | 7.25 |
| ILMN_1715569 | CCDC53 | 1.75 | 3.66 | 3.69 | 3.39 | 9.79 | 9.58 | 9.35 | 9.34 | 9.38 |
| ILMN_1714965 | NFKB1 | 1.75 | 2.58 | 4.82 | 7.16 | 10.70 | 10.51 | 10.42 | 10.18 | 9.92 |
| ILMN_1686811 | LOC402644 | 1.75 | 2.33 | 2.27 | 6.13 | 12.64 | 12.34 | 12.24 | 12.25 | 11.60 |
| ILMN_2395204 | SLTM | 1.75 | 0.94 | 1.61 | 2.98 | 10.15 | 9.90 | 10.02 | 9.92 | 9.73 |
| ILMN_2369286 | NME7 | 1.75 | 2.42 | 2.42 | 2.91 | 7.80 | 7.62 | 7.56 | 7.56 | 7.51 |
| ILMN_1705630 | LOC641700 | 1.76 | 1.45 | 1.11 | 2.94 | 7.21 | 7.06 | 7.09 | 7.12 | 6.96 |
| ILMN_2195236 | PGRMC2 | 1.76 | 1.67 | 1.08 | 5.77 | 10.12 | 9.86 | 9.87 | 9.96 | 9.27 |
| ILMN_2322806 | CAST | 1.76 | 2.37 | 0.93 | 5.78 | 8.65 | 8.43 | 8.35 | 8.53 | 7.92 |
| ILMN_1785170 | ARMCX2 | 1.76 | 1.34 | 2.78 | 5.23 | 6.93 | 6.81 | 6.84 | 6.73 | 6.56 |
| ILMN_2380494 | ANXA11 | 1.76 | 1.29 | 2.09 | 5.71 | 7.88 | 7.70 | 7.75 | 7.67 | 7.30 |
| ILMN_3199929 | LOC390183 | 1.76 | 2.46 | 3.19 | 4.64 | 10.55 | 10.28 | 10.18 | 10.07 | 9.85 |
| ILMN_1709623 | MAPK7 | 1.76 | 0.94 | 1.92 | 2.83 | 7.09 | 6.95 | 7.01 | 6.93 | 6.86 |
| ILMN_1672009 | LOC130773 | 1.76 | 3.56 | 3.00 | 2.82 | 8.52 | 8.33 | 8.14 | 8.20 | 8.22 |
| ILMN_2044453 | LPAR5 | 1.77 | 1.58 | 1.09 | -2.82 | 8.54 | 8.38 | 8.40 | 8.44 | 8.79 |
| ILMN_1707631 | MED10 | 1.77 | 5.85 | 5.95 | 3.30 | 8.86 | 8.74 | 8.46 | 8.45 | 8.63 |
| ILMN_1762292 | LOC644873 | 1.77 | 2.49 | 1.82 | 3.06 | 6.83 | 6.71 | 6.66 | 6.71 | 6.62 |
| ILMN_1691942 | CCNI | 1.78 | 0.14 | 1.19 | 4.52 | 12.52 | 12.31 | 12.50 | 12.38 | 11.98 |
| ILMN_1815734 | FCHSD2 | 1.78 | 1.03 | 2.23 | 4.32 | 9.28 | 8.88 | 9.05 | 8.78 | 8.32 |
| ILMN_3245752 | LOC100134011 | 1.78 | 0.17 | 1.64 | -4.02 | 7.44 | 7.27 | 7.42 | 7.28 | 7.82 |
| ILMN_2183784 | TTC12 | 1.78 | 2.47 | 2.94 | 3.41 | 8.00 | 7.75 | 7.65 | 7.59 | 7.52 |
| ILMN_1859946 |  | 1.79 | -0.20 | 0.48 | -4.72 | 10.62 | 10.47 | 10.64 | 10.58 | 11.02 |
| ILMN_1741281 | RNF175 | 1.79 | 4.22 | 2.85 | 4.54 | 7.82 | 7.58 | 7.25 | 7.43 | 7.21 |
| ILMN_1706957 | BMPR1A | 1.79 | -1.47 | 0.74 | 7.83 | 7.74 | 7.62 | 7.83 | 7.69 | 7.24 |
| ILMN_2092118 | FPR1 | 1.79 | 0.85 | 1.84 | 3.38 | 6.93 | 6.81 | 6.88 | 6.81 | 6.71 |
| ILMN_3216336 | LOC285741 | 1.79 | 1.52 | 2.18 | 3.88 | 11.60 | 11.36 | 11.40 | 11.31 | 11.08 |
| ILMN_2405628 | TOP1MT | 1.79 | 1.73 | 2.16 | 2.93 | 10.34 | 9.95 | 9.97 | 9.87 | 9.71 |
| ILMN_1737943 | THPO | 1.79 | 1.79 | 2.82 | 4.15 | 6.95 | 6.83 | 6.83 | 6.76 | 6.68 |
| ILMN_2140207 | ATPBD4 | 1.79 | 0.43 | 1.43 | 4.02 | 7.74 | 7.56 | 7.69 | 7.59 | 7.33 |
| ILMN_3237452 | C17orf100 | 1.79 | 1.64 | 2.62 | 4.14 | 7.85 | 7.66 | 7.67 | 7.57 | 7.40 |
| ILMN_2311089 | BRCA1 | 1.80 | 1.33 | 4.32 | 4.36 | 7.66 | 7.52 | 7.56 | 7.32 | 7.32 |
| ILMN_1688158 | CYB5R4 | 1.80 | 1.66 | 1.63 | 5.02 | 8.82 | 8.65 | 8.66 | 8.66 | 8.33 |
| ILMN_1659415 | MAP2K1IP1 | 1.80 | 1.96 | 2.88 | 3.66 | 9.96 | 9.70 | 9.68 | 9.55 | 9.43 |
| ILMN_1754279 | FBXW7 | 1.80 | 1.04 | 1.01 | 6.01 | 9.16 | 8.97 | 9.05 | 9.06 | 8.53 |
| ILMN_1689274 | NIPA1 | 1.80 | 1.48 | 0.92 | 5.50 | 10.29 | 10.01 | 10.06 | 10.14 | 9.43 |
| ILMN_1806040 | TYMS | 1.80 | 1.17 | 3.50 | 3.50 | 11.18 | 11.01 | 11.07 | 10.85 | 10.85 |
| ILMN_2058347 | NT5DC1 | 1.81 | 1.62 | 1.47 | 3.71 | 7.11 | 6.98 | 7.00 | 7.01 | 6.85 |
| ILMN_1717197 | CD3G | 1.81 | 2.93 | 1.57 | 6.52 | 7.62 | 7.42 | 7.29 | 7.44 | 6.89 |
| ILMN_1705774 | TIGD5 | 1.81 | 2.14 | 2.63 | 2.72 | 8.88 | 8.63 | 8.58 | 8.51 | 8.50 |
| ILMN_1771835 | NUP54 | 1.81 | 2.16 | 3.49 | 5.46 | 8.70 | 8.51 | 8.48 | 8.34 | 8.13 |
| ILMN_2226324 | BRP44L | 1.82 | 3.68 | 1.60 | 6.05 | 11.22 | 11.08 | 10.94 | 11.10 | 10.76 |
| ILMN_1720440 | HELQ | 1.82 | 2.69 | 2.44 | 2.96 | 8.14 | 7.89 | 7.78 | 7.81 | 7.74 |
| ILMN_2330341 | TCEAL4 | 1.82 | 0.71 | 2.53 | 3.48 | 7.09 | 6.93 | 7.03 | 6.87 | 6.79 |
| ILMN_2115336 | GNB3 | 1.82 | -2.75 | 0.32 | 3.13 | 8.45 | 8.26 | 8.73 | 8.41 | 8.12 |
| ILMN_2384807 | LRRCC1 | 1.82 | 2.63 | 2.70 | 3.34 | 7.65 | 7.47 | 7.39 | 7.38 | 7.32 |
| ILMN_2059357 | KLRC2 | 1.82 | 1.11 | 3.72 | 4.05 | 8.11 | 7.75 | 7.89 | 7.38 | 7.31 |
| ILMN_1809590 | GINS2 | 1.82 | 0.19 | 0.76 | 4.00 | 11.09 | 10.71 | 11.05 | 10.93 | 10.26 |
| ILMN_2168449 | DHX15 | 1.82 | 2.43 | 1.41 | 3.07 | 12.14 | 11.92 | 11.85 | 11.97 | 11.77 |
| ILMN_1727831 | RAD51L1 | 1.82 | 3.22 | 3.61 | 3.54 | 7.61 | 7.44 | 7.31 | 7.28 | 7.28 |
| ILMN_1737025 | PLCL2 | 1.82 | -0.09 | 1.18 | 3.38 | 10.48 | 10.27 | 10.49 | 10.34 | 10.09 |
| ILMN_2262901 | RUFY3 | 1.83 | 2.20 | 3.16 | 5.24 | 8.52 | 8.26 | 8.21 | 8.07 | 7.77 |
| ILMN_1688959 | CD27 | 1.83 | 1.85 | 1.00 | 2.84 | 10.25 | 9.80 | 9.80 | 10.00 | 9.56 |
| ILMN_1814011 | LOC731985 | 1.83 | 4.33 | 4.26 | 5.23 | 12.34 | 12.17 | 11.94 | 11.95 | 11.86 |
| ILMN_2113016 | C17orf39 | 1.83 | 1.78 | 2.23 | 3.96 | 7.12 | 6.98 | 6.99 | 6.95 | 6.82 |
| ILMN_2385161 | CUL4B | 1.83 | 1.49 | 0.85 | 4.03 | 8.49 | 8.31 | 8.35 | 8.41 | 8.10 |
| ILMN_2300186 | DYNLL1 | 1.83 | 2.68 | -0.93 | 3.39 | 11.50 | 11.33 | 11.26 | 11.58 | 11.19 |
| ILMN_2207988 | SERPINI1 | 1.83 | 2.51 | 2.11 | 5.02 | 7.18 | 7.01 | 6.94 | 6.98 | 6.71 |
| ILMN_3200421 | LOC641746 | 1.83 | 1.17 | 1.59 | 5.54 | 8.22 | 7.99 | 8.08 | 8.02 | 7.53 |
| ILMN_1734909 | CUZD1 | 1.83 | 2.14 | 2.81 | 3.30 | 7.18 | 7.04 | 7.02 | 6.97 | 6.93 |
| ILMN_1761594 | FAM35A | 1.83 | 1.35 | 3.03 | 3.18 | 9.29 | 9.09 | 9.14 | 8.95 | 8.94 |
| ILMN_3274143 | LOC649299 | 1.83 | 2.42 | 2.38 | 2.85 | 7.29 | 7.14 | 7.09 | 7.09 | 7.05 |
| ILMN_1763884 | Magmas | 1.84 | 2.16 | 3.79 | 2.25 | 9.44 | 9.18 | 9.13 | 8.90 | 9.12 |
| ILMN_1674983 | LOC387841 | 1.84 | 2.85 | 0.96 | 3.16 | 9.66 | 9.42 | 9.29 | 9.54 | 9.25 |
| ILMN_1776352 | MUTED | 1.84 | 2.37 | 3.07 | 3.10 | 9.21 | 9.00 | 8.94 | 8.86 | 8.86 |
| ILMN_1789505 | ITPR1 | 1.84 | 0.97 | 0.91 | 3.77 | 7.76 | 7.55 | 7.65 | 7.66 | 7.33 |
| ILMN_2255133 | BCL11A | 1.85 | 2.10 | 2.16 | 5.01 | 11.67 | 11.45 | 11.42 | 11.41 | 11.08 |
| ILMN_2135272 | GIMAP2 | 1.85 | 1.48 | 2.85 | 2.96 | 7.68 | 7.45 | 7.50 | 7.32 | 7.31 |
| ILMN_2344850 | VPS26A | 1.85 | 1.29 | 2.54 | 3.14 | 10.82 | 10.63 | 10.69 | 10.56 | 10.50 |
| ILMN_1730504 | AGPAT4 | 1.85 | -0.03 | 0.75 | 4.62 | 7.89 | 7.69 | 7.89 | 7.81 | 7.40 |
| ILMN_1684771 | PGRMC1 | 1.86 | 1.20 | 2.48 | 5.77 | 9.99 | 9.78 | 9.86 | 9.71 | 9.35 |
| ILMN_3248910 | MIR155HG | 1.86 | 2.95 | 4.84 | 3.59 | 9.44 | 9.10 | 8.90 | 8.55 | 8.78 |
| ILMN_2415467 | AP1GBP1 | 1.86 | 1.17 | 1.86 | 4.30 | 6.94 | 6.82 | 6.86 | 6.82 | 6.67 |
| ILMN_1669206 | CNOT1 | 1.86 | 1.77 | 2.59 | 8.47 | 11.33 | 11.19 | 11.19 | 11.13 | 10.68 |
| ILMN_1800958 | ALS2CR4 | 1.86 | 3.72 | 4.52 | 3.97 | 9.13 | 8.82 | 8.52 | 8.39 | 8.48 |
| ILMN_1664369 | DHTKD1 | 1.86 | 1.11 | 0.77 | 4.76 | 8.36 | 8.10 | 8.21 | 8.25 | 7.70 |
| ILMN_1762888 | FAM119A | 1.86 | 3.63 | 2.75 | 4.12 | 8.32 | 8.13 | 7.95 | 8.04 | 7.90 |
| ILMN_3203189 | LOC100133591 | 1.87 | 2.06 | 1.31 | 3.37 | 7.00 | 6.84 | 6.83 | 6.89 | 6.72 |
| ILMN_1791896 | EBAG9 | 1.87 | 4.15 | 4.78 | 4.62 | 9.88 | 9.68 | 9.44 | 9.38 | 9.39 |
| ILMN_2382309 | TCL1B | 1.87 | 0.07 | 1.44 | 3.23 | 13.23 | 13.06 | 13.23 | 13.10 | 12.93 |
| ILMN_1751143 | C7orf23 | 1.87 | 2.26 | 2.02 | 3.37 | 10.39 | 10.15 | 10.10 | 10.13 | 9.95 |
| ILMN_1672947 | CAST | 1.87 | 0.83 | 0.25 | 4.27 | 8.19 | 7.94 | 8.08 | 8.15 | 7.62 |
| ILMN_1656486 | DNAJC10 | 1.88 | 2.91 | 1.37 | 3.15 | 7.73 | 7.54 | 7.44 | 7.59 | 7.41 |
| ILMN_3178302 | FNDC3B | 1.88 | 0.05 | 2.32 | 2.82 | 7.05 | 6.93 | 7.05 | 6.90 | 6.86 |
| ILMN_2120210 | RCAN2 | 1.88 | 2.24 | 1.98 | 4.06 | 8.45 | 7.89 | 7.79 | 7.86 | 7.24 |
| ILMN_1659075 | HLA-DOA | 1.88 | 1.21 | 1.74 | 4.65 | 9.43 | 9.13 | 9.24 | 9.16 | 8.70 |
| ILMN_2087528 | CPSF3 | 1.88 | 2.66 | 3.86 | 4.87 | 11.56 | 11.42 | 11.37 | 11.28 | 11.21 |
| ILMN_1700428 | HLA-DOB | 1.89 | 2.63 | 3.63 | 6.70 | 9.50 | 9.20 | 9.08 | 8.93 | 8.44 |
| ILMN_1656427 | C17orf39 | 1.89 | 2.05 | 1.35 | 2.97 | 7.10 | 6.98 | 6.97 | 7.01 | 6.91 |
| ILMN_2295518 | TRO | 1.89 | 0.29 | 2.05 | 5.58 | 7.66 | 7.46 | 7.63 | 7.45 | 7.08 |
| ILMN_1682402 | SNORD46 | 1.89 | 0.69 | 1.89 | 3.63 | 7.71 | 7.35 | 7.58 | 7.35 | 7.01 |
| ILMN_1812616 | MYO1C | 1.89 | 2.60 | 2.77 | 3.51 | 7.29 | 7.07 | 6.98 | 6.96 | 6.88 |
| ILMN_2159152 | TP53TG3 | 1.89 | 2.59 | 3.88 | 2.84 | 7.37 | 7.17 | 7.10 | 6.96 | 7.07 |
| ILMN_1806165 | HSPA6 | 1.89 | 1.71 | 1.69 | 3.57 | 8.60 | 8.19 | 8.23 | 8.24 | 7.83 |
| ILMN_3201115 | LOC440043 | 1.90 | 0.79 | 2.24 | 3.44 | 12.30 | 12.10 | 12.22 | 12.06 | 11.94 |
| ILMN_1721901 | CTNNAL1 | 1.90 | 1.15 | 3.74 | 3.02 | 8.31 | 8.08 | 8.17 | 7.85 | 7.94 |
| ILMN_1694084 | PSCD1 | 1.90 | 1.87 | 2.77 | 6.10 | 11.08 | 10.88 | 10.89 | 10.79 | 10.45 |
| ILMN_1749799 | NSBP1 | 1.90 | 2.25 | 1.96 | 3.90 | 7.55 | 7.22 | 7.16 | 7.21 | 6.87 |
| ILMN_1878029 |  | 1.90 | 0.64 | 1.68 | 4.15 | 7.87 | 7.45 | 7.73 | 7.50 | 6.95 |
| ILMN_1704656 | PPP2R1B | 1.90 | 1.49 | 2.23 | 4.22 | 7.99 | 7.77 | 7.82 | 7.74 | 7.51 |
| ILMN_2309549 | AMZ2 | 1.91 | 1.50 | 2.32 | 2.73 | 7.01 | 6.89 | 6.91 | 6.86 | 6.83 |
| ILMN_1735822 | TTC30A | 1.91 | 2.22 | 3.39 | 3.65 | 7.17 | 7.03 | 7.00 | 6.91 | 6.89 |
| ILMN_1801254 | LOC648984 | 1.91 | 2.87 | 2.78 | 3.61 | 7.60 | 7.46 | 7.38 | 7.39 | 7.33 |
| ILMN_1745256 | CXXC5 | 1.92 | 0.73 | 2.05 | 3.54 | 12.22 | 11.98 | 12.13 | 11.96 | 11.77 |
| ILMN_1798233 | PSMB9 | 1.92 | 2.83 | 1.60 | 3.55 | 7.72 | 7.56 | 7.48 | 7.58 | 7.42 |
| ILMN_1808115 | ATP7A | 1.92 | -0.39 | 0.85 | 3.27 | 7.11 | 7.00 | 7.14 | 7.06 | 6.92 |
| ILMN_2380754 | CCNG1 | 1.92 | 2.15 | 3.27 | 2.96 | 7.31 | 7.12 | 7.10 | 6.99 | 7.02 |
| ILMN_1776327 | LOC727820 | 1.92 | -0.25 | 0.95 | 3.34 | 8.07 | 7.87 | 8.10 | 7.97 | 7.72 |
| ILMN_1797372 | C3orf58 | 1.92 | 0.81 | 0.05 | 4.71 | 8.96 | 8.72 | 8.86 | 8.95 | 8.38 |
| ILMN_1896406 |  | 1.92 | 0.10 | 0.25 | 4.31 | 7.00 | 6.87 | 6.99 | 6.98 | 6.72 |
| ILMN_1738909 | TROVE2 | 1.93 | 2.58 | 2.26 | 3.76 | 7.69 | 7.55 | 7.50 | 7.53 | 7.42 |
| ILMN_1739428 | IFIT2 | 1.93 | 1.91 | 1.97 | 3.14 | 7.49 | 7.17 | 7.17 | 7.16 | 6.96 |
| ILMN_1685095 | CNO | 1.93 | 1.64 | 0.38 | 4.56 | 9.69 | 9.45 | 9.49 | 9.64 | 9.12 |
| ILMN_1757702 | LOC647673 | 1.93 | 0.70 | 2.04 | 3.71 | 10.58 | 10.33 | 10.49 | 10.32 | 10.10 |
| ILMN_3251881 | LOC100130503 | 1.93 | 0.18 | 3.31 | 2.84 | 7.36 | 7.14 | 7.34 | 6.98 | 7.04 |
| ILMN_2211780 | SLC25A4 | 1.93 | 1.77 | 1.00 | 4.97 | 11.85 | 11.66 | 11.68 | 11.75 | 11.36 |
| ILMN_1693630 | C16orf7 | 1.93 | -0.49 | 0.18 | 4.27 | 7.01 | 6.86 | 7.04 | 6.99 | 6.69 |
| ILMN_3225121 | LOC728484 | 1.93 | 3.29 | 2.73 | 5.32 | 10.40 | 10.13 | 9.93 | 10.01 | 9.64 |
| ILMN_1781173 | HDAC9 | 1.94 | -0.11 | 0.89 | 3.17 | 8.27 | 7.93 | 8.29 | 8.11 | 7.71 |
| ILMN_2194627 | GMCL1 | 1.94 | 1.04 | 0.74 | 2.78 | 8.29 | 8.05 | 8.16 | 8.20 | 7.95 |
| ILMN_1803045 | TUBGCP5 | 1.94 | 0.97 | 2.44 | 4.96 | 8.72 | 8.44 | 8.58 | 8.37 | 8.00 |
| ILMN_1769734 | NT5C3 | 1.94 | 1.85 | 0.17 | 2.95 | 9.28 | 9.05 | 9.06 | 9.26 | 8.93 |
| ILMN_1766505 | COMMD10 | 1.94 | 1.28 | 0.88 | 5.60 | 9.38 | 9.23 | 9.28 | 9.31 | 8.96 |
| ILMN_1772218 | HLA-DPA1 | 1.94 | 2.31 | 2.38 | 4.47 | 12.06 | 11.86 | 11.82 | 11.81 | 11.59 |
| ILMN_1709486 | SRPX | 1.94 | 2.01 | 1.51 | 3.49 | 7.00 | 6.80 | 6.79 | 6.84 | 6.64 |
| ILMN_2093343 | PLAC8 | 1.94 | 2.78 | 2.80 | 7.01 | 11.64 | 11.17 | 10.97 | 10.96 | 9.93 |
| ILMN_2389347 | NR3C1 | 1.94 | 0.95 | 1.87 | 3.23 | 7.32 | 7.15 | 7.24 | 7.15 | 7.03 |
| ILMN_1807300 | PKD2 | 1.95 | -0.40 | 1.59 | 3.18 | 7.21 | 7.04 | 7.24 | 7.07 | 6.94 |
| ILMN_1653129 | CSTF2 | 1.95 | 1.02 | 0.82 | 4.32 | 9.64 | 9.38 | 9.51 | 9.53 | 9.07 |
| ILMN_1730622 | EVL | 1.95 | 0.98 | 2.11 | 2.91 | 11.34 | 11.01 | 11.17 | 10.98 | 10.85 |
| ILMN_1733390 | LARP1B | 1.95 | 2.48 | 1.89 | 9.44 | 9.47 | 9.27 | 9.22 | 9.28 | 8.52 |
| ILMN_1667711 | HRASLS3 | 1.96 | 2.17 | 1.47 | 4.19 | 7.20 | 6.99 | 6.97 | 7.04 | 6.75 |
| ILMN_2398995 | MRPL24 | 1.96 | 1.35 | 0.46 | -2.76 | 9.50 | 9.28 | 9.35 | 9.45 | 9.82 |
| ILMN_1724753 | NIN | 1.96 | 0.99 | -0.42 | -2.80 | 9.02 | 8.80 | 8.91 | 9.07 | 9.33 |
| ILMN_2168564 | KLHL14 | 1.96 | 1.77 | -0.58 | 5.71 | 8.56 | 8.36 | 8.38 | 8.62 | 7.97 |
| ILMN_1829664 |  | 1.96 | 2.03 | 1.58 | 2.92 | 7.62 | 7.32 | 7.31 | 7.38 | 7.18 |
| ILMN_1654609 | TIGA1 | 1.96 | 3.03 | 6.63 | 1.30 | 10.57 | 10.38 | 10.27 | 9.91 | 10.44 |
| ILMN_1670870 | ALCAM | 1.97 | 0.21 | -0.50 | 2.83 | 8.39 | 8.05 | 8.35 | 8.48 | 7.90 |
| ILMN_1678494 | ZNF438 | 1.97 | 2.72 | 2.86 | 4.63 | 7.17 | 7.03 | 6.97 | 6.96 | 6.84 |
| ILMN_1809141 | ING4 | 1.97 | 1.09 | 3.90 | 2.71 | 7.82 | 7.63 | 7.71 | 7.44 | 7.55 |
| ILMN_2414762 | TLR10 | 1.97 | 1.87 | 0.48 | 4.93 | 8.31 | 7.86 | 7.88 | 8.20 | 7.18 |
| ILMN_2373831 | BTN3A3 | 1.97 | 2.74 | 2.57 | 3.52 | 7.18 | 6.99 | 6.92 | 6.93 | 6.84 |
| ILMN_1767362 | ADAMTS6 | 1.97 | 1.64 | 4.07 | 5.28 | 7.08 | 6.93 | 6.95 | 6.76 | 6.66 |
| ILMN_1733374 | LOC642197 | 1.97 | 2.03 | 2.25 | 2.80 | 8.85 | 8.64 | 8.63 | 8.61 | 8.55 |
| ILMN_3305397 | LOC728843 | 1.97 | 2.00 | 2.60 | 3.60 | 9.80 | 9.53 | 9.53 | 9.45 | 9.32 |
| ILMN_1748481 | TMEM199 | 1.97 | 3.25 | 3.42 | 2.81 | 8.66 | 8.42 | 8.27 | 8.25 | 8.32 |
| ILMN_1743783 | CCDC43 | 1.98 | 1.37 | 3.53 | 3.56 | 8.37 | 8.16 | 8.22 | 7.99 | 7.98 |
| ILMN_1682336 | MASTL | 1.98 | 1.51 | 2.75 | 4.96 | 8.57 | 8.40 | 8.44 | 8.34 | 8.15 |
| ILMN_2104486 | GAGE8 | 1.98 | 0.48 | 0.85 | 2.83 | 7.13 | 6.76 | 7.04 | 6.97 | 6.60 |
| ILMN_2312719 | EXOSC9 | 1.98 | 2.77 | 2.55 | 6.02 | 10.68 | 10.35 | 10.22 | 10.26 | 9.68 |
| ILMN_3244506 | LOC441089 | 1.98 | 0.28 | 0.90 | 3.53 | 10.39 | 10.18 | 10.36 | 10.30 | 10.01 |
| ILMN_2389590 | PRKAR1A | 1.98 | 0.89 | 2.32 | 3.45 | 11.42 | 11.22 | 11.33 | 11.19 | 11.08 |
| ILMN_2340935 | WBP5 | 1.99 | 1.19 | 2.03 | 3.44 | 7.89 | 7.58 | 7.70 | 7.57 | 7.36 |
| ILMN_1863284 |  | 1.99 | 2.81 | 3.79 | 4.41 | 7.62 | 7.45 | 7.38 | 7.30 | 7.24 |
| ILMN_1796455 | RYR3 | 1.99 | 1.68 | 2.80 | 3.49 | 7.04 | 6.83 | 6.86 | 6.74 | 6.67 |
| ILMN_1752335 | CEACAM19 | 1.99 | 2.52 | -0.77 | 2.93 | 7.01 | 6.84 | 6.80 | 7.07 | 6.77 |
| ILMN_1737416 | PGBD3 | 2.00 | 1.35 | 2.39 | 3.34 | 7.13 | 6.97 | 7.02 | 6.94 | 6.87 |
| ILMN_1656410 | TRPC6 | 2.00 | 1.64 | 0.16 | 3.22 | 7.73 | 7.31 | 7.38 | 7.70 | 7.05 |
| ILMN_1664994 | MINPP1 | 2.00 | 1.55 | 1.55 | 4.37 | 7.96 | 7.79 | 7.83 | 7.83 | 7.60 |
| ILMN_2102693 | NUFIP2 | 2.00 | 2.25 | 2.78 | 3.34 | 9.28 | 9.07 | 9.04 | 8.98 | 8.92 |
| ILMN_2126832 | SEC24A | 2.00 | 3.45 | 1.10 | 4.70 | 8.25 | 8.12 | 8.02 | 8.18 | 7.94 |
| ILMN_2128795 | LRIG1 | 2.00 | 0.86 | 2.79 | 4.56 | 9.57 | 9.08 | 9.36 | 8.89 | 8.45 |
| ILMN_1799467 | SAMD9L | 2.00 | 1.32 | 2.39 | 3.35 | 7.17 | 6.93 | 7.01 | 6.89 | 6.77 |
| ILMN_1779706 | TP53BP2 | 2.00 | 1.85 | 2.60 | 4.49 | 8.31 | 8.13 | 8.15 | 8.08 | 7.91 |
| ILMN_2327812 | IL5RA | 2.00 | 3.31 | 3.53 | 7.20 | 7.65 | 7.41 | 7.25 | 7.22 | 6.78 |
| ILMN_1790637 | C11orf80 | 2.00 | 1.60 | 2.41 | 6.15 | 8.19 | 8.00 | 8.04 | 7.96 | 7.59 |
| ILMN_1736015 | PHF17 | 2.01 | 1.07 | 1.95 | 8.05 | 9.36 | 9.11 | 9.23 | 9.12 | 8.36 |
| ILMN_1751234 | C1GALT1C1 | 2.01 | 3.19 | 1.08 | 3.73 | 8.53 | 8.27 | 8.11 | 8.39 | 8.04 |
| ILMN_1654671 | SLMO1 | 2.01 | -0.46 | 4.10 | 6.31 | 7.89 | 7.73 | 7.93 | 7.57 | 7.39 |
| ILMN_1885728 | KIAA1147 | 2.01 | 0.33 | 0.96 | 3.79 | 7.58 | 7.38 | 7.55 | 7.49 | 7.20 |
| ILMN_1668634 | FBXW7 | 2.01 | 2.75 | -0.83 | 5.59 | 8.25 | 8.04 | 7.97 | 8.34 | 7.67 |
| ILMN_1761031 | PTPDC1 | 2.02 | 1.61 | 2.78 | 3.70 | 7.84 | 7.59 | 7.64 | 7.50 | 7.38 |
| ILMN_1694106 | GPD1L | 2.02 | 0.81 | 1.12 | 3.74 | 10.22 | 9.99 | 10.13 | 10.09 | 9.80 |
| ILMN_1739045 | LOC647009 | 2.02 | 2.11 | 1.45 | 4.34 | 8.37 | 8.15 | 8.14 | 8.21 | 7.89 |
| ILMN_1805271 | ZNF721 | 2.02 | 0.98 | 1.88 | 2.92 | 9.87 | 9.55 | 9.72 | 9.57 | 9.41 |
| ILMN_1797001 | DDX58 | 2.02 | 2.08 | 1.47 | 3.96 | 7.29 | 7.07 | 7.07 | 7.13 | 6.86 |
| ILMN_1659103 | PSMA8 | 2.02 | 2.62 | 2.20 | 3.00 | 7.49 | 7.30 | 7.24 | 7.28 | 7.21 |
| ILMN_3243705 | PDXDC1 | 2.03 | 2.85 | 2.96 | 3.26 | 10.41 | 10.18 | 10.09 | 10.07 | 10.04 |
| ILMN_1762639 | MED11 | 2.03 | 2.00 | 2.19 | 2.81 | 7.69 | 7.48 | 7.48 | 7.46 | 7.40 |
| ILMN_2151114 | VSNL1 | 2.03 | 1.98 | 1.52 | 3.46 | 6.91 | 6.77 | 6.78 | 6.81 | 6.68 |
| ILMN_1674302 | PPAT | 2.03 | 1.49 | 2.50 | 3.08 | 9.91 | 9.65 | 9.72 | 9.59 | 9.52 |
| ILMN_3207605 | LOC399804 | 2.03 | 2.03 | 2.89 | 5.39 | 8.01 | 7.82 | 7.82 | 7.74 | 7.50 |
| ILMN_3276019 | LOC728060 | 2.03 | 4.49 | 3.47 | 4.90 | 9.55 | 9.29 | 8.98 | 9.11 | 8.92 |
| ILMN_1756550 | NUP62CL | 2.04 | 1.03 | 1.05 | 4.05 | 7.65 | 7.34 | 7.49 | 7.49 | 7.04 |
| ILMN_1735908 | UTP15 | 2.04 | 1.64 | 3.32 | 2.84 | 8.04 | 7.87 | 7.90 | 7.76 | 7.80 |
| ILMN_1798659 | CCDC28A | 2.04 | 2.18 | 4.36 | 0.85 | 8.62 | 8.48 | 8.47 | 8.31 | 8.56 |
| ILMN_1718070 | CASP9 | 2.04 | 0.80 | 1.20 | -3.10 | 7.90 | 7.75 | 7.84 | 7.81 | 8.13 |
| ILMN_1668714 | SIK2 | 2.04 | -0.94 | 0.50 | 2.98 | 6.75 | 6.62 | 6.82 | 6.72 | 6.55 |
| ILMN_1695026 | LOC642775 | 2.04 | 2.00 | 1.11 | 3.45 | 7.23 | 7.05 | 7.05 | 7.13 | 6.92 |
| ILMN_2154566 | RPL10A | 2.06 | 2.44 | 2.63 | 3.08 | 13.81 | 13.63 | 13.59 | 13.58 | 13.54 |
| ILMN_3202396 | LOC390735 | 2.06 | 2.42 | 1.91 | 4.88 | 10.16 | 9.87 | 9.82 | 9.89 | 9.48 |
| ILMN_1731714 | CREB5 | 2.06 | 1.83 | 2.43 | 3.84 | 8.57 | 7.93 | 8.00 | 7.81 | 7.37 |
| ILMN_1671372 | LOC137107 | 2.06 | 1.74 | 2.70 | 2.99 | 8.30 | 8.10 | 8.13 | 8.04 | 8.02 |
| ILMN_3274711 | LOC392501 | 2.07 | 3.15 | 4.39 | 3.19 | 8.11 | 7.82 | 7.66 | 7.49 | 7.66 |
| ILMN_1778347 | NUDT2 | 2.07 | 2.82 | 3.75 | 2.59 | 9.58 | 9.28 | 9.17 | 9.03 | 9.20 |
| ILMN_1700834 | SLK | 2.07 | 0.63 | 2.03 | 3.47 | 7.56 | 7.38 | 7.51 | 7.38 | 7.25 |
| ILMN_1738656 | GLOD4 | 2.07 | 1.00 | 3.01 | 2.89 | 9.88 | 9.65 | 9.77 | 9.55 | 9.56 |
| ILMN_1717852 | USH1G | 2.07 | 2.07 | 2.71 | 3.47 | 8.04 | 7.82 | 7.82 | 7.76 | 7.68 |
| ILMN_1702301 | DOCK10 | 2.07 | 2.85 | 2.73 | 3.27 | 10.70 | 10.47 | 10.39 | 10.40 | 10.34 |
| ILMN_1674460 | ATP2A1 | 2.07 | 2.69 | 0.06 | 2.79 | 8.28 | 7.02 | 6.64 | 8.24 | 6.58 |
| ILMN_3303162 | LOC728979 | 2.07 | 2.24 | 2.24 | 3.36 | 7.19 | 7.02 | 7.01 | 7.01 | 6.92 |
| ILMN_1764617 | GPATCH8 | 2.07 | 2.34 | 3.11 | 3.65 | 7.14 | 6.99 | 6.97 | 6.91 | 6.87 |
| ILMN_1781085 | BRDG1 | 2.08 | 1.99 | 1.90 | 10.18 | 9.15 | 8.90 | 8.91 | 8.92 | 7.95 |
| ILMN_1732696 | SSX6 | 2.08 | 3.85 | 2.98 | 4.42 | 8.16 | 7.91 | 7.69 | 7.80 | 7.62 |
| ILMN_1651405 | BRD9 | 2.08 | -0.51 | 1.02 | 3.25 | 10.91 | 10.68 | 10.97 | 10.80 | 10.55 |
| ILMN_1703435 | ZNF658 | 2.08 | 3.16 | 3.20 | 3.81 | 7.13 | 6.97 | 6.88 | 6.88 | 6.83 |
| ILMN_1719905 | TLR10 | 2.08 | 2.29 | 0.52 | 6.92 | 8.56 | 8.13 | 8.09 | 8.45 | 7.14 |
| ILMN_1775034 | LOC649987 | 2.08 | 2.22 | 1.77 | 5.49 | 7.65 | 7.49 | 7.48 | 7.51 | 7.23 |
| ILMN_2185339 | LRTOMT | 2.08 | 0.40 | 1.80 | 5.03 | 7.59 | 7.40 | 7.55 | 7.42 | 7.12 |
| ILMN_1753607 | PNO1 | 2.09 | 1.72 | 2.72 | 2.99 | 9.29 | 9.01 | 9.06 | 8.92 | 8.89 |
| ILMN_1706217 | TLR4 | 2.09 | 1.64 | 1.86 | 2.93 | 6.89 | 6.71 | 6.75 | 6.73 | 6.63 |
| ILMN_1711878 | ENOPH1 | 2.09 | 1.47 | 2.09 | 4.45 | 10.08 | 9.89 | 9.94 | 9.89 | 9.67 |
| ILMN_1710495 | PAPLN | 2.10 | 2.24 | 2.50 | 7.76 | 8.83 | 8.39 | 8.36 | 8.30 | 7.18 |
| ILMN_1733523 | NARG1L | 2.10 | 1.29 | 1.06 | 3.88 | 7.92 | 7.67 | 7.77 | 7.80 | 7.46 |
| ILMN_1700546 | ELOVL6 | 2.10 | 2.17 | 2.65 | 4.16 | 8.38 | 7.90 | 7.88 | 7.77 | 7.42 |
| ILMN_2216852 | PGK1 | 2.10 | 1.05 | -0.77 | 3.78 | 10.44 | 10.19 | 10.31 | 10.53 | 9.98 |
| ILMN_3273229 | LOC100129781 | 2.10 | 2.25 | 2.54 | 2.73 | 6.94 | 6.79 | 6.78 | 6.76 | 6.75 |
| ILMN_3233388 | RELL1 | 2.10 | 2.60 | -1.60 | 7.48 | 9.11 | 8.86 | 8.80 | 9.30 | 8.22 |
| ILMN_2060770 | RAI1 | 2.10 | 1.32 | 2.03 | 2.91 | 7.69 | 7.42 | 7.52 | 7.43 | 7.31 |
| ILMN_1709728 | SLC30A5 | 2.10 | 1.75 | 3.24 | 4.87 | 9.72 | 9.43 | 9.48 | 9.27 | 9.04 |
| ILMN_2090607 | IRF2 | 2.10 | 0.67 | 1.23 | 3.58 | 7.26 | 7.06 | 7.19 | 7.14 | 6.92 |
| ILMN_2061405 | NUP54 | 2.11 | 1.72 | 3.13 | 4.38 | 9.99 | 9.74 | 9.78 | 9.61 | 9.46 |
| ILMN_1741556 | MTMR1 | 2.11 | 1.30 | 4.44 | 1.00 | 8.38 | 8.07 | 8.19 | 7.74 | 8.23 |
| ILMN_1721921 | BLMH | 2.11 | 1.74 | 2.05 | 2.78 | 8.89 | 8.57 | 8.63 | 8.58 | 8.47 |
| ILMN_3235853 | S1PR1 | 2.12 | 0.27 | 1.41 | 3.53 | 7.92 | 7.74 | 7.90 | 7.80 | 7.62 |
| ILMN_2044226 | PPP3CA | 2.12 | 0.79 | -1.45 | 5.64 | 8.12 | 7.95 | 8.06 | 8.24 | 7.67 |
| ILMN_1656386 | SEC24D | 2.12 | 1.66 | 2.60 | 5.97 | 7.80 | 7.52 | 7.58 | 7.45 | 7.01 |
| ILMN_1672503 | DPYSL2 | 2.12 | 1.64 | 0.65 | 11.10 | 11.31 | 11.11 | 11.15 | 11.25 | 10.23 |
| ILMN_1699623 | FAM81A | 2.12 | 2.66 | 2.55 | 6.75 | 8.60 | 8.22 | 8.12 | 8.14 | 7.39 |
| ILMN_1670925 | CYB5D1 | 2.12 | 3.08 | 4.58 | 2.12 | 8.08 | 7.77 | 7.62 | 7.40 | 7.77 |
| ILMN_3292082 | LOC642367 | 2.13 | 1.22 | 3.50 | 4.29 | 7.71 | 7.53 | 7.61 | 7.41 | 7.35 |
| ILMN_1687884 | ZNF2 | 2.13 | 1.10 | 1.66 | 3.62 | 7.71 | 7.49 | 7.60 | 7.54 | 7.34 |
| ILMN_1792301 | LAMB4 | 2.13 | 2.45 | 3.15 | 3.03 | 7.06 | 6.82 | 6.78 | 6.70 | 6.71 |
| ILMN_1788778 | 41528 | 2.13 | 2.38 | 4.20 | 5.71 | 8.61 | 7.98 | 7.91 | 7.38 | 6.94 |
| ILMN_3230337 | LOC100132707 | 2.13 | 1.07 | 1.89 | 3.08 | 7.10 | 6.92 | 7.01 | 6.94 | 6.84 |
| ILMN_3242271 | GAPT | 2.13 | 3.06 | 2.27 | 4.93 | 8.10 | 7.79 | 7.66 | 7.77 | 7.39 |
| ILMN_3244168 | GAGE2A | 2.13 | 1.03 | 0.98 | 3.02 | 7.35 | 6.89 | 7.13 | 7.14 | 6.69 |
| ILMN_2371825 | AGL | 2.13 | 1.42 | 2.45 | 2.95 | 9.47 | 9.27 | 9.34 | 9.24 | 9.20 |
| ILMN_2415926 | THOC3 | 2.14 | 1.79 | 4.49 | -1.23 | 9.22 | 9.02 | 9.05 | 8.80 | 9.34 |
| ILMN_2412172 | APOBEC3F | 2.15 | 2.04 | 1.58 | 2.94 | 7.23 | 7.04 | 7.05 | 7.09 | 6.97 |
| ILMN_1751589 | NUDCD2 | 2.15 | 5.15 | 1.77 | 3.23 | 10.13 | 9.95 | 9.69 | 9.98 | 9.85 |
| ILMN_1685661 | RRP15 | 2.15 | 1.82 | 2.59 | 4.14 | 9.76 | 9.55 | 9.58 | 9.50 | 9.35 |
| ILMN_1683112 | FANCC | 2.15 | 0.57 | 0.53 | 3.25 | 7.26 | 7.07 | 7.21 | 7.21 | 6.97 |
| ILMN_1713486 | RNF25 | 2.16 | 2.78 | 4.41 | 1.92 | 7.54 | 7.39 | 7.35 | 7.24 | 7.41 |
| ILMN_2377669 | CD247 | 2.16 | 1.42 | 1.37 | 3.37 | 11.04 | 10.76 | 10.86 | 10.86 | 10.61 |
| ILMN_1802106 | APOBEC3G | 2.16 | 4.76 | 3.16 | 4.35 | 8.05 | 7.82 | 7.55 | 7.72 | 7.59 |
| ILMN_1763000 | ADAP2 | 2.16 | 2.23 | 2.16 | 2.97 | 7.00 | 6.80 | 6.79 | 6.80 | 6.72 |
| ILMN_3237256 | BEND4 | 2.16 | 1.71 | 1.36 | 3.16 | 8.98 | 8.66 | 8.73 | 8.78 | 8.51 |
| ILMN_1769277 | LOC651436 | 2.16 | 2.58 | 2.61 | 2.91 | 13.55 | 13.31 | 13.27 | 13.26 | 13.23 |
| ILMN_2125010 | SKAP2 | 2.16 | 1.95 | 1.39 | 5.66 | 8.82 | 8.57 | 8.60 | 8.66 | 8.18 |
| ILMN_1811551 | DERA | 2.17 | 2.82 | 2.60 | 7.50 | 9.88 | 9.71 | 9.66 | 9.68 | 9.30 |
| ILMN_3238106 | FAM161A | 2.17 | 1.31 | 2.17 | 3.44 | 7.73 | 7.49 | 7.59 | 7.49 | 7.35 |
| ILMN_2099528 | BTLA | 2.17 | -1.32 | 2.26 | 3.30 | 6.80 | 6.64 | 6.89 | 6.64 | 6.56 |
| ILMN_1674243 | TFRC | 2.17 | 0.77 | 4.01 | 0.07 | 11.74 | 11.52 | 11.66 | 11.34 | 11.73 |
| ILMN_1660027 | FCGR2B | 2.17 | 3.30 | 3.01 | 3.42 | 10.36 | 10.12 | 9.99 | 10.02 | 9.98 |
| ILMN_1802631 | AGA | 2.17 | 0.69 | 1.30 | 5.87 | 8.35 | 8.16 | 8.29 | 8.23 | 7.83 |
| ILMN_1695386 | RAD51C | 2.18 | 3.87 | 4.58 | 3.44 | 10.18 | 9.93 | 9.73 | 9.65 | 9.78 |
| ILMN_1738438 | MAST4 | 2.18 | 3.16 | 4.00 | 5.77 | 8.95 | 8.50 | 8.29 | 8.12 | 7.75 |
| ILMN_1762879 | DCAF4L1 | 2.18 | 2.52 | 2.28 | 3.22 | 7.20 | 6.90 | 6.85 | 6.89 | 6.76 |
| ILMN_1801766 | CCDC109B | 2.19 | 1.51 | 0.68 | 8.53 | 11.64 | 11.42 | 11.49 | 11.57 | 10.76 |
| ILMN_2311020 | DNAJC12 | 2.19 | 2.00 | 4.31 | 6.22 | 8.00 | 7.66 | 7.69 | 7.33 | 7.03 |
| ILMN_1760335 | ADPRHL1 | 2.19 | -0.88 | 0.23 | 5.80 | 9.91 | 9.50 | 10.07 | 9.87 | 8.83 |
| ILMN_1734198 | RNGTT | 2.19 | -0.25 | 1.06 | 7.52 | 11.82 | 11.61 | 11.84 | 11.72 | 11.11 |
| ILMN_1725773 | DNAJC12 | 2.19 | 2.22 | 4.61 | 5.14 | 7.46 | 7.17 | 7.17 | 6.86 | 6.79 |
| ILMN_1666594 | IRF8 | 2.19 | 1.00 | 0.83 | 4.24 | 12.04 | 11.78 | 11.92 | 11.94 | 11.54 |
| ILMN_3246247 | LOC399491 | 2.19 | 0.53 | 2.88 | 3.54 | 8.24 | 8.00 | 8.18 | 7.93 | 7.86 |
| ILMN_1792660 | CAMSAP1L1 | 2.19 | 0.11 | 1.60 | 4.63 | 7.58 | 7.31 | 7.57 | 7.39 | 7.01 |
| ILMN_1669497 | OSBPL10 | 2.20 | 2.77 | 1.81 | 5.35 | 10.54 | 10.18 | 10.09 | 10.24 | 9.66 |
| ILMN_2056551 | RBMX2 | 2.20 | 2.51 | 2.62 | 6.36 | 9.21 | 8.95 | 8.92 | 8.90 | 8.47 |
| ILMN_1851492 |  | 2.20 | 1.01 | 1.35 | 4.06 | 7.73 | 7.60 | 7.67 | 7.65 | 7.49 |
| ILMN_1691747 | KHDRBS3 | 2.20 | 2.16 | 3.87 | 3.22 | 7.35 | 7.17 | 7.17 | 7.03 | 7.09 |
| ILMN_3236963 | GAGE2E | 2.20 | 1.03 | 0.85 | 3.29 | 7.35 | 6.86 | 7.12 | 7.16 | 6.62 |
| ILMN_1725338 | CLDN23 | 2.20 | 2.41 | 1.70 | 2.87 | 11.56 | 11.09 | 11.05 | 11.20 | 10.95 |
| ILMN_2413231 | MAGEB1 | 2.20 | 2.46 | 2.91 | 2.94 | 6.94 | 6.68 | 6.65 | 6.59 | 6.59 |
| ILMN_1768510 | MAN2B2 | 2.20 | 1.01 | 0.10 | 2.81 | 9.60 | 9.24 | 9.43 | 9.58 | 9.14 |
| ILMN_2134974 | RAB38 | 2.20 | 0.86 | -0.90 | 3.55 | 7.10 | 6.90 | 7.02 | 7.18 | 6.78 |
| ILMN_3240247 | NOP10 | 2.20 | 1.35 | 1.84 | 3.96 | 12.37 | 12.21 | 12.27 | 12.23 | 12.08 |
| ILMN_2390453 | BCKDHB | 2.21 | 2.12 | 2.09 | 4.71 | 7.84 | 7.60 | 7.61 | 7.61 | 7.33 |
| ILMN_2326512 | CASP1 | 2.22 | 2.86 | 3.90 | 6.49 | 10.08 | 9.78 | 9.69 | 9.55 | 9.20 |
| ILMN_1667199 | SQRDL | 2.22 | 2.28 | 2.94 | 8.20 | 10.01 | 9.36 | 9.34 | 9.15 | 7.61 |
| ILMN_1663631 | BANP | 2.22 | 2.79 | 1.22 | 6.09 | 11.16 | 10.95 | 10.90 | 11.04 | 10.59 |
| ILMN_1741264 | MRPS33 | 2.22 | 2.86 | 4.29 | 1.43 | 10.84 | 10.56 | 10.48 | 10.30 | 10.66 |
| ILMN_1799589 | NOXA1 | 2.23 | 0.15 | 1.27 | 2.78 | 7.07 | 6.83 | 7.05 | 6.93 | 6.77 |
| ILMN_1768751 | MTA3 | 2.23 | 2.09 | 2.89 | 3.14 | 8.79 | 8.52 | 8.54 | 8.44 | 8.41 |
| ILMN_1661307 | JRK | 2.23 | 2.33 | 2.72 | 3.37 | 6.94 | 6.79 | 6.79 | 6.76 | 6.72 |
| ILMN_1675462 | LSM6 | 2.23 | 2.39 | 1.13 | 6.02 | 8.78 | 8.46 | 8.44 | 8.62 | 7.91 |
| ILMN_3301740 | LOC729887 | 2.23 | 0.23 | 1.62 | 5.30 | 8.19 | 7.96 | 8.16 | 8.02 | 7.64 |
| ILMN_2354269 | FAM164C | 2.23 | 2.82 | 4.16 | 2.13 | 7.82 | 7.67 | 7.63 | 7.54 | 7.67 |
| ILMN_1744046 | DIAPH2 | 2.23 | 3.07 | 1.67 | 8.61 | 8.96 | 8.74 | 8.66 | 8.80 | 8.13 |
| ILMN_1788607 | RPL23A | 2.23 | 4.22 | 4.62 | 4.87 | 10.19 | 9.92 | 9.67 | 9.62 | 9.59 |
| ILMN_2338921 | C4orf41 | 2.23 | 0.49 | 2.15 | 5.15 | 9.74 | 9.46 | 9.68 | 9.47 | 9.11 |
| ILMN_1812043 | LOC642570 | 2.24 | 2.94 | 4.11 | 5.05 | 8.31 | 7.67 | 7.46 | 7.12 | 6.85 |
| ILMN_2221076 | C17orf85 | 2.24 | 2.28 | 4.39 | 3.99 | 8.14 | 7.95 | 7.95 | 7.77 | 7.80 |
| ILMN_3287583 | LOC648390 | 2.24 | 2.24 | 3.64 | 3.64 | 10.76 | 10.46 | 10.46 | 10.28 | 10.28 |
| ILMN_3248890 | SNORA24 | 2.24 | 2.05 | 3.04 | 3.81 | 9.39 | 8.84 | 8.88 | 8.64 | 8.45 |
| ILMN_1659583 | LOC644617 | 2.24 | 2.17 | 1.43 | 2.90 | 7.21 | 7.01 | 7.02 | 7.08 | 6.96 |
| ILMN_1696330 | GUF1 | 2.24 | 3.75 | 6.34 | 6.27 | 8.72 | 8.51 | 8.36 | 8.12 | 8.12 |
| ILMN_2110532 | RPL26L1 | 2.24 | 3.50 | 3.75 | 1.94 | 10.39 | 10.12 | 9.96 | 9.93 | 10.15 |
| ILMN_1815500 | ITPR3 | 2.25 | 0.22 | 2.45 | 3.36 | 10.02 | 9.58 | 9.98 | 9.54 | 9.37 |
| ILMN_1745116 | ABHD12 | 2.25 | 1.73 | 1.18 | 4.02 | 8.33 | 8.13 | 8.17 | 8.22 | 7.97 |
| ILMN_2355462 | CYFIP1 | 2.25 | 2.35 | 3.39 | 5.70 | 7.32 | 7.09 | 7.08 | 6.97 | 6.74 |
| ILMN_1716563 | PRKCB1 | 2.25 | 2.04 | 1.62 | 2.88 | 9.14 | 8.82 | 8.85 | 8.91 | 8.73 |
| ILMN_1690282 | CRADD | 2.25 | 1.79 | 2.32 | 5.93 | 8.80 | 8.59 | 8.63 | 8.58 | 8.24 |
| ILMN_3280496 | LOC100131526 | 2.26 | 3.77 | 4.41 | 4.24 | 9.52 | 9.11 | 8.83 | 8.72 | 8.75 |
| ILMN_1737819 | C7orf16 | 2.26 | 1.78 | 2.93 | 4.47 | 6.87 | 6.72 | 6.75 | 6.67 | 6.56 |
| ILMN_1785795 | METAP1 | 2.26 | 2.09 | 2.87 | 4.43 | 10.11 | 9.85 | 9.87 | 9.78 | 9.60 |
| ILMN_1737514 | KYNU | 2.26 | 3.95 | 2.18 | 5.98 | 8.14 | 7.85 | 7.63 | 7.86 | 7.37 |
| ILMN_1780036 | WDR1 | 2.27 | 2.97 | 2.80 | 6.14 | 11.68 | 11.37 | 11.28 | 11.30 | 10.84 |
| ILMN_2150284 | RNPC3 | 2.27 | 0.51 | 2.04 | 2.82 | 8.49 | 8.09 | 8.40 | 8.13 | 8.00 |
| ILMN_1738607 | LOC646350 | 2.27 | 0.80 | 1.09 | 3.39 | 7.84 | 7.56 | 7.74 | 7.70 | 7.42 |
| ILMN_1763663 | FLJ20718 | 2.27 | -0.57 | 1.06 | 4.52 | 9.55 | 9.25 | 9.63 | 9.41 | 8.94 |
| ILMN_2102069 | SLC16A4 | 2.27 | 3.19 | 3.96 | 3.92 | 7.97 | 7.58 | 7.43 | 7.29 | 7.30 |
| ILMN_1798880 | RPA4 | 2.28 | -1.00 | -0.47 | 3.04 | 6.95 | 6.79 | 7.02 | 6.98 | 6.74 |
| ILMN_3272424 | LOC100128836 | 2.28 | 1.78 | 3.92 | 3.79 | 9.08 | 8.85 | 8.90 | 8.69 | 8.70 |
| ILMN_1745599 | LOC643977 | 2.28 | 0.68 | 0.59 | 3.46 | 7.12 | 6.95 | 7.07 | 7.08 | 6.87 |
| ILMN_1797342 | FNBP1 | 2.28 | 0.21 | 2.30 | 4.65 | 7.96 | 7.64 | 7.93 | 7.64 | 7.31 |
| ILMN_1805526 | MYL1 | 2.28 | 2.83 | 0.42 | 2.97 | 8.89 | 7.21 | 6.80 | 8.58 | 6.70 |
| ILMN_2136446 | CTNNAL1 | 2.28 | 1.55 | 3.63 | 4.39 | 8.50 | 8.24 | 8.32 | 8.09 | 8.00 |
| ILMN_1695079 | ZNF101 | 2.28 | 1.67 | 4.71 | 0.95 | 7.56 | 7.36 | 7.42 | 7.15 | 7.48 |
| ILMN_1808789 | MYO5C | 2.28 | 1.69 | 2.10 | 3.97 | 7.17 | 7.00 | 7.05 | 7.02 | 6.88 |
| ILMN_1801703 | CPLX1 | 2.28 | 2.03 | 3.59 | 3.60 | 8.95 | 8.17 | 8.26 | 7.73 | 7.72 |
| ILMN_1656254 | NOTCH2NL | 2.28 | 3.17 | 3.94 | -0.17 | 8.14 | 7.88 | 7.77 | 7.68 | 8.16 |
| ILMN_3285356 | LOC645944 | 2.29 | 1.60 | 2.29 | 3.56 | 7.38 | 7.20 | 7.25 | 7.20 | 7.09 |
| ILMN_1802456 | DCTD | 2.29 | 0.35 | 2.47 | 4.97 | 9.66 | 9.45 | 9.63 | 9.43 | 9.19 |
| ILMN_1754114 | FLJ20021 | 2.30 | 1.63 | 2.83 | 5.02 | 8.42 | 8.21 | 8.27 | 8.16 | 7.95 |
| ILMN_2120072 | FLJ13305 | 2.30 | 1.72 | 1.80 | 4.41 | 7.21 | 7.01 | 7.06 | 7.05 | 6.83 |
| ILMN_3238058 | LOC151162 | 2.30 | -1.55 | 1.23 | -3.65 | 9.63 | 9.43 | 9.76 | 9.52 | 9.93 |
| ILMN_1812297 | CYP26B1 | 2.30 | 1.49 | 3.67 | 4.85 | 7.72 | 7.29 | 7.44 | 7.03 | 6.80 |
| ILMN_1845086 |  | 2.31 | 1.71 | 1.92 | 4.00 | 7.38 | 7.05 | 7.13 | 7.10 | 6.80 |
| ILMN_3201216 | LOC441550 | 2.31 | 2.34 | 3.48 | 4.35 | 9.83 | 9.53 | 9.52 | 9.37 | 9.26 |
| ILMN_1707475 | UBE2E2 | 2.31 | 2.59 | 3.14 | 6.53 | 9.85 | 9.57 | 9.54 | 9.47 | 9.06 |
| ILMN_3222631 | LOC729433 | 2.31 | 1.61 | -0.25 | 2.87 | 6.88 | 6.73 | 6.78 | 6.90 | 6.69 |
| ILMN_1859908 |  | 2.32 | 1.90 | 3.15 | 7.13 | 9.29 | 8.90 | 8.97 | 8.76 | 8.10 |
| ILMN_1782034 | ZXDA | 2.32 | 0.51 | 1.58 | 4.30 | 6.88 | 6.74 | 6.85 | 6.79 | 6.63 |
| ILMN_2406335 | ANKRD17 | 2.32 | 0.89 | 0.80 | 6.07 | 8.47 | 8.30 | 8.41 | 8.41 | 8.02 |
| ILMN_1733364 | QRSL1 | 2.32 | 0.74 | 1.16 | 3.48 | 7.58 | 7.42 | 7.53 | 7.50 | 7.33 |
| ILMN_1718424 | MRPS28 | 2.33 | 2.91 | 3.89 | 2.91 | 9.23 | 8.98 | 8.92 | 8.81 | 8.92 |
| ILMN_1900344 |  | 2.33 | 1.81 | 2.33 | 3.02 | 6.92 | 6.74 | 6.78 | 6.74 | 6.69 |
| ILMN_3225761 | MGC87895 | 2.33 | 2.25 | 2.61 | 3.22 | 12.81 | 12.52 | 12.53 | 12.48 | 12.40 |
| ILMN_1670901 | COX10 | 2.33 | 2.96 | 3.71 | 2.82 | 8.63 | 8.36 | 8.29 | 8.20 | 8.31 |
| ILMN_3200661 | LOC389404 | 2.34 | 3.20 | 2.16 | 3.14 | 13.44 | 13.19 | 13.09 | 13.21 | 13.10 |
| ILMN_1657283 | ALKBH5 | 2.35 | 1.97 | 2.20 | 3.12 | 11.38 | 11.11 | 11.15 | 11.12 | 11.02 |
| ILMN_2123431 | RPS6KC1 | 2.35 | 2.49 | 3.07 | 4.73 | 8.01 | 7.80 | 7.78 | 7.73 | 7.58 |
| ILMN_2213297 | C11orf54 | 2.35 | 3.09 | 1.96 | 7.26 | 9.39 | 9.13 | 9.04 | 9.17 | 8.58 |
| ILMN_1736178 | AEBP1 | 2.35 | -1.13 | 0.73 | 3.13 | 10.67 | 10.40 | 10.80 | 10.58 | 10.31 |
| ILMN_1687571 | LOC728734 | 2.35 | 0.73 | 1.71 | 2.86 | 7.15 | 6.97 | 7.09 | 7.02 | 6.93 |
| ILMN_2190051 | CCDC91 | 2.36 | 2.08 | 2.83 | 3.96 | 7.38 | 7.15 | 7.18 | 7.11 | 7.00 |
| ILMN_1707342 | LRIG1 | 2.36 | 0.55 | 2.91 | 4.16 | 8.27 | 7.88 | 8.18 | 7.78 | 7.57 |
| ILMN_2115862 | ESPNL | 2.36 | 1.10 | 2.43 | -4.12 | 8.75 | 8.32 | 8.55 | 8.31 | 9.49 |
| ILMN_1748650 | MRPL45 | 2.36 | 2.91 | 4.18 | 5.67 | 10.32 | 10.04 | 9.98 | 9.83 | 9.66 |
| ILMN_2162860 | SLFN11 | 2.37 | 0.69 | 2.87 | 4.90 | 9.10 | 8.81 | 9.02 | 8.74 | 8.49 |
| ILMN_1707448 | CRKRS | 2.37 | 2.83 | 2.12 | 4.45 | 8.32 | 8.09 | 8.05 | 8.12 | 7.90 |
| ILMN_1695588 | HNRPC | 2.37 | 0.77 | 2.18 | 3.56 | 10.73 | 10.48 | 10.65 | 10.50 | 10.36 |
| ILMN_2128741 | RDH11 | 2.38 | 2.30 | 2.38 | 3.64 | 8.70 | 8.48 | 8.49 | 8.48 | 8.37 |
| ILMN_1797082 | SNX13 | 2.38 | 2.81 | 2.38 | 4.69 | 8.03 | 7.78 | 7.73 | 7.78 | 7.53 |
| ILMN_1681543 | RHBDD1 | 2.38 | 2.93 | 3.03 | 3.13 | 9.40 | 9.00 | 8.90 | 8.89 | 8.87 |
| ILMN_3259712 | LOC100129952 | 2.38 | 1.31 | 1.29 | 2.93 | 9.56 | 9.16 | 9.34 | 9.34 | 9.06 |
| ILMN_2204142 | PPP1R2P9 | 2.38 | 2.08 | 3.24 | 3.24 | 6.91 | 6.70 | 6.73 | 6.62 | 6.62 |
| ILMN_1748625 | TCEAL4 | 2.38 | 1.64 | 3.96 | 4.02 | 8.68 | 8.26 | 8.39 | 7.99 | 7.98 |
| ILMN_2331735 | AP2B1 | 2.38 | 2.38 | 1.56 | 2.83 | 7.90 | 7.69 | 7.69 | 7.76 | 7.65 |
| ILMN_2413236 | MAGEB1 | 2.39 | 2.37 | 2.87 | 2.96 | 7.16 | 6.73 | 6.74 | 6.65 | 6.63 |
| ILMN_3270866 | LOC100130255 | 2.39 | 1.15 | 1.77 | 2.93 | 7.49 | 7.30 | 7.40 | 7.35 | 7.26 |
| ILMN_1690342 | LTA4H | 2.39 | 3.30 | 4.36 | 2.82 | 11.68 | 11.40 | 11.30 | 11.17 | 11.35 |
| ILMN_1745423 | UTP3 | 2.39 | 2.47 | 2.96 | 6.62 | 9.82 | 9.50 | 9.49 | 9.43 | 8.95 |
| ILMN_3239168 | LOC645332 | 2.40 | 2.24 | 2.83 | 3.23 | 7.59 | 7.39 | 7.40 | 7.35 | 7.32 |
| ILMN_2365544 | NHP2 | 2.40 | 1.50 | 2.72 | 4.63 | 11.35 | 11.15 | 11.23 | 11.13 | 10.97 |
| ILMN_1741881 | C9orf72 | 2.40 | 1.70 | 1.07 | 4.72 | 8.14 | 7.93 | 7.99 | 8.05 | 7.72 |
| ILMN_2343048 | ABCB9 | 2.40 | 1.14 | 2.59 | 3.18 | 9.11 | 8.83 | 8.98 | 8.80 | 8.73 |
| ILMN_1660727 | ENPP5 | 2.40 | 2.44 | 3.36 | 3.56 | 7.03 | 6.82 | 6.82 | 6.74 | 6.72 |
| ILMN_1795428 | WDR59 | 2.40 | -0.07 | 1.38 | 7.46 | 9.10 | 8.86 | 9.11 | 8.96 | 8.35 |
| ILMN_1792207 | MLX | 2.40 | 2.63 | 2.63 | 4.11 | 7.03 | 6.86 | 6.84 | 6.84 | 6.73 |
| ILMN_1795119 | MYH3 | 2.40 | 1.43 | 4.43 | 2.49 | 7.10 | 6.92 | 6.99 | 6.78 | 6.92 |
| ILMN_2318685 | ABHD12 | 2.40 | 3.24 | 2.51 | 3.71 | 7.63 | 7.48 | 7.43 | 7.47 | 7.40 |
| ILMN_1760849 | NETO2 | 2.40 | 0.70 | 2.04 | 5.70 | 8.36 | 7.82 | 8.20 | 7.90 | 7.08 |
| ILMN_1799128 | SLC30A9 | 2.41 | 1.89 | 1.63 | 5.81 | 10.43 | 10.21 | 10.26 | 10.28 | 9.90 |
| ILMN_2379788 | HIF1A | 2.41 | -0.51 | -0.04 | 4.43 | 8.26 | 8.05 | 8.30 | 8.26 | 7.88 |
| ILMN_1862217 |  | 2.41 | 1.42 | 3.40 | 3.23 | 9.12 | 8.94 | 9.01 | 8.86 | 8.87 |
| ILMN_1815134 | PI4K2B | 2.41 | 2.49 | 1.97 | 4.12 | 10.06 | 9.84 | 9.84 | 9.88 | 9.69 |
| ILMN_2404454 | NLE1 | 2.41 | 1.39 | 4.72 | 3.29 | 7.83 | 7.61 | 7.70 | 7.40 | 7.53 |
| ILMN_1774602 | FBLN2 | 2.41 | 2.28 | 4.37 | 6.90 | 8.99 | 8.32 | 8.36 | 7.78 | 7.08 |
| ILMN_2153825 | MEIG1 | 2.42 | 2.17 | 2.07 | 5.08 | 7.25 | 7.09 | 7.10 | 7.11 | 6.91 |
| ILMN_3244574 | KIAA1430 | 2.42 | 0.76 | 0.45 | 3.25 | 8.07 | 7.86 | 8.00 | 8.03 | 7.78 |
| ILMN_3244755 | LOC342994 | 2.42 | 2.54 | 3.20 | 4.80 | 7.94 | 7.73 | 7.72 | 7.66 | 7.53 |
| ILMN_3251298 | THNSL1 | 2.42 | 2.42 | 2.42 | 4.92 | 7.21 | 7.00 | 7.00 | 7.00 | 6.79 |
| ILMN_1679438 | MLF1IP | 2.42 | -0.17 | 2.47 | 3.40 | 8.85 | 8.51 | 8.88 | 8.51 | 8.38 |
| ILMN_1691567 | GNPDA2 | 2.43 | 1.49 | 3.40 | 5.40 | 7.93 | 7.74 | 7.82 | 7.67 | 7.51 |
| ILMN_1720266 | LOC91431 | 2.43 | 0.24 | 1.21 | 3.11 | 8.29 | 7.93 | 8.26 | 8.11 | 7.82 |
| ILMN_1782178 | LOC642946 | 2.43 | 1.84 | 1.61 | 4.37 | 7.61 | 7.37 | 7.43 | 7.45 | 7.17 |
| ILMN_1747305 | GIMAP8 | 2.43 | 2.49 | 3.64 | 3.85 | 10.46 | 10.05 | 10.04 | 9.84 | 9.80 |
| ILMN_1695590 | ADRB2 | 2.43 | 1.97 | 1.85 | 7.47 | 8.47 | 8.19 | 8.25 | 8.26 | 7.61 |
| ILMN_1705346 | NBEA | 2.43 | 2.79 | 3.07 | 4.39 | 7.14 | 6.94 | 6.91 | 6.89 | 6.78 |
| ILMN_3250592 | FAM86A | 2.43 | 1.93 | 1.64 | 3.23 | 7.76 | 7.53 | 7.58 | 7.61 | 7.46 |
| ILMN_3244196 | LOC729088 | 2.43 | 0.20 | 1.08 | 3.19 | 7.07 | 6.87 | 7.06 | 6.98 | 6.81 |
| ILMN_1706117 | LOC391656 | 2.44 | 2.59 | 3.01 | 3.94 | 13.79 | 13.52 | 13.50 | 13.46 | 13.35 |
| ILMN_1745365 | PKNOX1 | 2.44 | 0.65 | 1.86 | 3.31 | 9.43 | 8.94 | 9.30 | 9.06 | 8.77 |
| ILMN_3250870 | USP46 | 2.44 | 1.13 | 2.23 | 3.42 | 7.23 | 6.97 | 7.11 | 6.99 | 6.86 |
| ILMN_1705247 | ACSL5 | 2.44 | 1.19 | 1.05 | 3.35 | 9.35 | 9.12 | 9.24 | 9.25 | 9.03 |
| ILMN_1660125 | SFMBT2 | 2.45 | 1.58 | 1.20 | 2.80 | 8.06 | 7.73 | 7.85 | 7.90 | 7.69 |
| ILMN_3198499 | LOC654350 | 2.46 | 2.09 | 3.90 | 7.94 | 10.48 | 10.24 | 10.28 | 10.10 | 9.70 |
| ILMN_2346573 | PSME3 | 2.46 | 3.24 | 4.44 | 4.59 | 9.47 | 9.19 | 9.11 | 8.97 | 8.96 |
| ILMN_1810852 | LAMC1 | 2.46 | 0.96 | 1.40 | 4.42 | 8.97 | 8.72 | 8.87 | 8.83 | 8.52 |
| ILMN_3275447 | LOC391126 | 2.46 | 3.18 | 5.35 | 5.33 | 12.18 | 11.90 | 11.81 | 11.56 | 11.56 |
| ILMN_1779674 | PTPRG | 2.46 | 1.78 | 4.05 | 4.43 | 7.09 | 6.87 | 6.93 | 6.73 | 6.70 |
| ILMN_1765876 | ARHGAP24 | 2.46 | 0.96 | 2.96 | 2.99 | 7.90 | 7.50 | 7.74 | 7.42 | 7.42 |
| ILMN_1732688 | DUT | 2.47 | -0.29 | 3.24 | 3.24 | 10.15 | 9.90 | 10.18 | 9.82 | 9.82 |
| ILMN_1725043 | ADAL | 2.47 | 2.44 | 3.67 | 5.52 | 8.10 | 7.79 | 7.80 | 7.64 | 7.41 |
| ILMN_1683888 | SRP72 | 2.47 | 1.20 | 2.32 | 4.02 | 9.81 | 9.60 | 9.71 | 9.61 | 9.46 |
| ILMN_1713491 | VAMP2 | 2.47 | 1.69 | 3.47 | 2.78 | 8.11 | 7.77 | 7.88 | 7.63 | 7.72 |
| ILMN_1810431 | LOC642299 | 2.47 | 3.19 | 3.50 | 3.00 | 9.18 | 8.66 | 8.51 | 8.44 | 8.55 |
| ILMN_1762327 | DHRS12 | 2.48 | 1.31 | 0.83 | 3.06 | 6.99 | 6.82 | 6.90 | 6.94 | 6.78 |
| ILMN_1655796 | 03-Mar | 2.48 | 1.24 | 2.93 | 2.78 | 7.53 | 7.20 | 7.37 | 7.14 | 7.16 |
| ILMN_2130180 | RPL13L | 2.48 | 0.85 | 1.48 | 8.45 | 11.21 | 10.81 | 11.07 | 10.97 | 9.86 |
| ILMN_1741176 | CHMP2B | 2.48 | 1.28 | 4.13 | 5.10 | 8.35 | 8.13 | 8.23 | 7.98 | 7.89 |
| ILMN_2399896 | SEC31A | 2.48 | 1.16 | 1.10 | 3.32 | 10.13 | 9.87 | 10.01 | 10.02 | 9.78 |
| ILMN_1763326 | C5orf25 | 2.48 | 2.26 | 4.42 | 6.72 | 7.98 | 7.75 | 7.77 | 7.57 | 7.36 |
| ILMN_3264543 | LOC100128060 | 2.48 | 1.63 | 2.26 | 3.26 | 10.79 | 10.24 | 10.43 | 10.29 | 10.06 |
| ILMN_1740633 | PRF1 | 2.48 | 2.43 | 4.02 | 3.87 | 7.25 | 6.91 | 6.91 | 6.70 | 6.72 |
| ILMN_2112811 | RPL36A | 2.48 | 2.51 | 3.98 | 1.66 | 8.03 | 7.72 | 7.72 | 7.54 | 7.83 |
| ILMN_1813938 | CHCHD4 | 2.49 | 4.15 | 5.23 | 1.41 | 10.05 | 9.82 | 9.67 | 9.58 | 9.92 |
| ILMN_1726879 | PRR15 | 2.49 | 1.41 | 1.74 | 3.00 | 6.98 | 6.80 | 6.88 | 6.86 | 6.77 |
| ILMN_2390919 | FBLN2 | 2.49 | 1.79 | 4.08 | 6.69 | 8.63 | 7.99 | 8.17 | 7.58 | 6.91 |
| ILMN_2393243 | GTPBP8 | 2.49 | 3.15 | 3.11 | 3.35 | 8.12 | 7.92 | 7.86 | 7.87 | 7.85 |
| ILMN_1672878 | ABR | 2.49 | 1.50 | 2.90 | 2.75 | 10.38 | 9.93 | 10.11 | 9.86 | 9.88 |
| ILMN_1784880 | TAF9L | 2.49 | 3.13 | 2.41 | 6.00 | 7.65 | 7.46 | 7.41 | 7.46 | 7.18 |
| ILMN_1731546 | RPL26 | 2.49 | 3.77 | 5.75 | 4.28 | 12.14 | 11.80 | 11.63 | 11.36 | 11.56 |
| ILMN_2398926 | C17orf58 | 2.50 | 2.86 | 3.26 | 4.84 | 8.96 | 8.68 | 8.64 | 8.60 | 8.43 |
| ILMN_1764396 | HDAC4 | 2.50 | 0.50 | 0.91 | 3.10 | 7.66 | 7.31 | 7.59 | 7.53 | 7.23 |
| ILMN_1729515 | PIN4 | 2.50 | 2.00 | 2.20 | 3.89 | 7.37 | 7.16 | 7.20 | 7.18 | 7.04 |
| ILMN_1803593 | WNT3 | 2.50 | 0.83 | 1.63 | 3.34 | 7.05 | 6.81 | 6.97 | 6.89 | 6.73 |
| ILMN_1780298 | FAM86A | 2.51 | 2.62 | 2.98 | 4.38 | 7.71 | 7.41 | 7.39 | 7.35 | 7.18 |
| ILMN_1802973 | ANAPC4 | 2.51 | 2.22 | 3.50 | 3.40 | 9.28 | 8.94 | 8.98 | 8.81 | 8.82 |
| ILMN_2154603 | CCDC16 | 2.51 | 1.82 | 2.83 | 2.98 | 9.66 | 9.29 | 9.39 | 9.24 | 9.22 |
| ILMN_1742026 | PYHIN1 | 2.51 | 2.36 | 4.48 | 1.25 | 7.66 | 7.28 | 7.30 | 6.98 | 7.47 |
| ILMN_1690807 | MKL2 | 2.52 | 2.04 | 2.83 | 6.92 | 8.20 | 7.93 | 7.98 | 7.90 | 7.47 |
| ILMN_1726846 | LOC649856 | 2.52 | 3.15 | 3.99 | 4.75 | 8.14 | 7.38 | 7.19 | 6.94 | 6.72 |
| ILMN_1724612 | SLC25A35 | 2.52 | 1.77 | 3.43 | 3.27 | 6.90 | 6.74 | 6.79 | 6.68 | 6.69 |
| ILMN_1739576 | CYB5R2 | 2.52 | 2.86 | 5.10 | 0.72 | 8.29 | 7.87 | 7.81 | 7.44 | 8.17 |
| ILMN_1772702 | SFRS2B | 2.53 | 1.87 | 2.17 | 4.48 | 9.77 | 9.49 | 9.56 | 9.53 | 9.27 |
| ILMN_2320250 | NOL6 | 2.54 | 1.34 | 3.99 | 2.45 | 10.31 | 9.93 | 10.11 | 9.71 | 9.94 |
| ILMN_3232156 | C7orf58 | 2.54 | 2.64 | 3.43 | 4.82 | 6.93 | 6.76 | 6.76 | 6.70 | 6.61 |
| ILMN_2202423 | HELLS | 2.54 | 1.18 | 3.75 | 5.13 | 9.52 | 9.22 | 9.38 | 9.07 | 8.91 |
| ILMN_3290199 | LOC441073 | 2.54 | 3.67 | 4.85 | 4.94 | 12.81 | 12.45 | 12.28 | 12.11 | 12.10 |
| ILMN_1795429 | VCL | 2.54 | 2.26 | 1.17 | 2.85 | 10.75 | 10.28 | 10.33 | 10.53 | 10.22 |
| ILMN_2169736 | PGBD4 | 2.55 | 3.08 | 4.92 | 4.09 | 6.90 | 6.76 | 6.73 | 6.63 | 6.67 |
| ILMN_1793671 | TFDP2 | 2.55 | 2.24 | 4.13 | 4.48 | 7.05 | 6.86 | 6.88 | 6.74 | 6.71 |
| ILMN_1664016 | ARHGEF18 | 2.55 | 0.00 | 1.49 | 4.44 | 10.68 | 10.45 | 10.68 | 10.54 | 10.27 |
| ILMN_1661470 | LOC648931 | 2.55 | 3.40 | 3.35 | 4.73 | 7.02 | 6.86 | 6.80 | 6.81 | 6.72 |
| ILMN_2392261 | FABP6 | 2.55 | 2.20 | 3.34 | 3.71 | 8.21 | 7.85 | 7.90 | 7.73 | 7.68 |
| ILMN_2366790 | DDX52 | 2.55 | 2.06 | 4.01 | 3.60 | 9.65 | 9.34 | 9.40 | 9.16 | 9.21 |
| ILMN_1679881 | WRN | 2.55 | 2.25 | 3.79 | -0.44 | 8.15 | 7.95 | 7.98 | 7.86 | 8.18 |
| ILMN_2067269 | RECK | 2.56 | 0.71 | 2.42 | 3.60 | 7.50 | 7.24 | 7.43 | 7.26 | 7.14 |
| ILMN_2166524 | CCNYL1 | 2.56 | 2.33 | 4.22 | 4.72 | 7.20 | 7.04 | 7.06 | 6.94 | 6.91 |
| ILMN_1673417 | NHEDC1 | 2.56 | 2.09 | 1.77 | 3.31 | 6.95 | 6.74 | 6.78 | 6.80 | 6.67 |
| ILMN_1707312 | NFIL3 | 2.56 | 2.79 | 4.04 | 3.81 | 7.54 | 7.39 | 7.37 | 7.30 | 7.31 |
| ILMN_2208455 | DDHD1 | 2.56 | 2.56 | 2.20 | 3.09 | 7.13 | 6.92 | 6.92 | 6.95 | 6.88 |
| ILMN_1755075 | IDI1 | 2.56 | 2.69 | 1.11 | 2.91 | 9.47 | 9.05 | 9.03 | 9.29 | 8.99 |
| ILMN_1810531 | DRG2 | 2.57 | 2.19 | 5.20 | 5.02 | 7.46 | 7.23 | 7.26 | 6.99 | 7.01 |
| ILMN_2414533 | ARMCX6 | 2.57 | 3.09 | 2.23 | 2.94 | 7.89 | 7.66 | 7.62 | 7.69 | 7.63 |
| ILMN_1797191 | KIAA0040 | 2.57 | 3.20 | 3.10 | 3.50 | 8.25 | 8.00 | 7.93 | 7.94 | 7.90 |
| ILMN_2149566 | VPS25 | 2.57 | 1.43 | 3.11 | 2.84 | 8.56 | 8.27 | 8.40 | 8.21 | 8.24 |
| ILMN_1787843 | HSDL2 | 2.57 | 2.95 | 3.21 | 6.25 | 8.47 | 8.20 | 8.16 | 8.13 | 7.81 |
| ILMN_1659437 | TXNDC17 | 2.57 | 4.01 | 3.91 | 1.89 | 11.35 | 11.09 | 10.94 | 10.95 | 11.16 |
| ILMN_3277297 | LOC391825 | 2.58 | 4.57 | 3.74 | 4.14 | 8.80 | 8.52 | 8.30 | 8.39 | 8.35 |
| ILMN_1663646 | DMXL1 | 2.58 | 3.85 | 2.23 | 3.47 | 8.02 | 7.77 | 7.65 | 7.81 | 7.69 |
| ILMN_1772700 | TMEM18 | 2.58 | 1.21 | 2.98 | 4.95 | 8.06 | 7.89 | 7.98 | 7.86 | 7.73 |
| ILMN_2374778 | DUT | 2.58 | 0.34 | 3.07 | 3.13 | 9.33 | 9.05 | 9.29 | 8.99 | 8.99 |
| ILMN_1737398 | PTPLAD1 | 2.58 | 0.51 | 2.62 | 3.09 | 11.10 | 10.85 | 11.05 | 10.84 | 10.80 |
| ILMN_2330267 | ABCE1 | 2.58 | 2.26 | 1.57 | 6.18 | 10.61 | 10.32 | 10.36 | 10.44 | 9.92 |
| ILMN_2343278 | PPAP2A | 2.58 | 2.48 | 3.09 | 6.86 | 7.48 | 7.24 | 7.25 | 7.20 | 6.85 |
| ILMN_1852384 |  | 2.59 | -0.63 | 1.05 | 3.06 | 8.44 | 8.13 | 8.52 | 8.32 | 8.07 |
| ILMN_1753472 | SUMF1 | 2.59 | 2.50 | 4.01 | 4.34 | 7.48 | 7.27 | 7.28 | 7.16 | 7.13 |
| ILMN_1806149 | C16orf74 | 2.59 | 2.01 | 1.62 | 3.69 | 7.85 | 7.59 | 7.65 | 7.69 | 7.47 |
| ILMN_2135984 | MASTL | 2.59 | 0.81 | 1.33 | 3.01 | 8.15 | 7.82 | 8.05 | 7.98 | 7.77 |
| ILMN_2313926 | CDC42SE2 | 2.60 | 2.57 | 1.77 | 3.19 | 9.79 | 9.51 | 9.51 | 9.60 | 9.45 |
| ILMN_3236130 | LOC100132547 | 2.60 | 3.43 | 4.19 | 3.72 | 10.02 | 9.46 | 9.29 | 9.13 | 9.23 |
| ILMN_1761969 | DERL2 | 2.61 | 2.31 | 4.10 | -0.45 | 8.18 | 7.94 | 7.97 | 7.81 | 8.22 |
| ILMN_1664910 | RPSA | 2.61 | 0.89 | 2.42 | 2.77 | 8.03 | 7.65 | 7.90 | 7.67 | 7.62 |
| ILMN_3246793 | OOEP | 2.61 | 1.73 | 1.89 | 3.73 | 7.18 | 6.96 | 7.04 | 7.02 | 6.87 |
| ILMN_2407824 | ATP1B1 | 2.61 | -0.12 | -0.37 | 6.02 | 7.56 | 7.27 | 7.57 | 7.60 | 6.90 |
| ILMN_2145396 | AKR7A3 | 2.61 | 1.93 | 2.19 | 3.00 | 9.08 | 8.81 | 8.88 | 8.86 | 8.77 |
| ILMN_2299843 | ATP5S | 2.61 | 2.00 | 2.47 | 2.90 | 8.12 | 7.94 | 7.98 | 7.95 | 7.92 |
| ILMN_1775235 | AFF3 | 2.62 | 1.31 | 2.02 | 5.39 | 10.77 | 10.49 | 10.63 | 10.55 | 10.19 |
| ILMN_1796397 | CISD2 | 2.62 | 1.82 | 2.81 | 4.05 | 8.62 | 8.31 | 8.40 | 8.28 | 8.13 |
| ILMN_3273885 | LOC100127983 | 2.62 | 3.63 | 2.53 | 5.42 | 7.08 | 6.89 | 6.82 | 6.90 | 6.69 |
| ILMN_1752899 | BCL11A | 2.62 | 1.82 | 0.95 | 4.52 | 11.77 | 11.43 | 11.53 | 11.65 | 11.18 |
| ILMN_1711414 | MRPS27 | 2.62 | 1.27 | 2.78 | 4.05 | 10.31 | 9.97 | 10.15 | 9.95 | 9.78 |
| ILMN_1820787 |  | 2.63 | 2.35 | 3.16 | 3.55 | 8.37 | 7.99 | 8.03 | 7.92 | 7.86 |
| ILMN_1666635 | RPS14 | 2.63 | 2.57 | 3.56 | 2.89 | 13.89 | 13.62 | 13.62 | 13.52 | 13.59 |
| ILMN_1717982 | BZW1 | 2.63 | 2.72 | 4.12 | 2.59 | 7.44 | 7.24 | 7.23 | 7.13 | 7.24 |
| ILMN_2278518 | NFATC2IP | 2.63 | 0.00 | 2.07 | 5.55 | 7.62 | 7.40 | 7.62 | 7.45 | 7.16 |
| ILMN_1764090 | AK3L1 | 2.64 | 1.62 | 2.30 | 3.83 | 6.99 | 6.78 | 6.86 | 6.81 | 6.69 |
| ILMN_3230435 | LOC729086 | 2.64 | 0.45 | 0.73 | 7.32 | 11.05 | 10.83 | 11.01 | 10.99 | 10.45 |
| ILMN_1793349 | MOBKL1A | 2.64 | 3.52 | 2.67 | 5.29 | 7.73 | 7.44 | 7.34 | 7.44 | 7.15 |
| ILMN_1722583 | COX4NB | 2.65 | 0.85 | 0.88 | 5.76 | 8.31 | 8.06 | 8.23 | 8.23 | 7.77 |
| ILMN_1675191 | GAPT | 2.65 | 3.73 | 2.26 | 5.99 | 8.60 | 8.27 | 8.13 | 8.32 | 7.84 |
| ILMN_1800390 | ZNF511 | 2.66 | 2.68 | 3.40 | 5.79 | 11.35 | 11.02 | 11.02 | 10.93 | 10.63 |
| ILMN_2268618 | PHF20L1 | 2.66 | 0.43 | 2.85 | 3.04 | 7.08 | 6.89 | 7.05 | 6.88 | 6.86 |
| ILMN_2085236 | SNX24 | 2.66 | 0.80 | 2.36 | 9.07 | 7.85 | 7.64 | 7.78 | 7.66 | 7.13 |
| ILMN_1791106 | HEATR6 | 2.66 | 2.39 | 4.03 | 0.90 | 7.67 | 7.44 | 7.46 | 7.32 | 7.59 |
| ILMN_2298366 | TLR10 | 2.66 | 1.04 | 2.29 | 3.24 | 6.90 | 6.69 | 6.82 | 6.72 | 6.64 |
| ILMN_1780996 | ETAA1 | 2.66 | 2.45 | 2.72 | 4.02 | 7.36 | 7.08 | 7.10 | 7.07 | 6.93 |
| ILMN_1657153 | ACTR3 | 2.66 | 3.03 | 3.00 | 3.08 | 11.47 | 11.16 | 11.11 | 11.12 | 11.11 |
| ILMN_2214355 | RAB30 | 2.66 | 5.44 | 4.53 | 7.41 | 8.33 | 8.09 | 7.84 | 7.92 | 7.66 |
| ILMN_2049642 | RPA1 | 2.67 | 2.10 | 3.88 | 2.55 | 10.72 | 10.50 | 10.55 | 10.40 | 10.51 |
| ILMN_2366972 | NUDT6 | 2.67 | 4.45 | 2.76 | 9.27 | 8.35 | 8.16 | 8.03 | 8.15 | 7.69 |
| ILMN_1718336 | C7orf50 | 2.67 | 0.46 | 1.28 | 2.87 | 11.38 | 11.03 | 11.32 | 11.21 | 11.01 |
| ILMN_1658266 | LOC146053 | 2.67 | 2.31 | 2.47 | 4.11 | 9.00 | 8.55 | 8.61 | 8.58 | 8.30 |
| ILMN_3226807 | NHP2 | 2.67 | 1.49 | 2.06 | 3.48 | 10.62 | 10.39 | 10.49 | 10.44 | 10.32 |
| ILMN_1695491 | WDYHV1 | 2.68 | 2.19 | 4.17 | 3.20 | 8.90 | 8.49 | 8.57 | 8.27 | 8.41 |
| ILMN_1776073 | CCT4 | 2.68 | 2.68 | 3.92 | 4.47 | 8.81 | 8.52 | 8.52 | 8.38 | 8.32 |
| ILMN_3236680 | LOC100134393 | 2.68 | 1.12 | 1.81 | 3.48 | 10.84 | 10.44 | 10.67 | 10.57 | 10.32 |
| ILMN_1721580 | TBX15 | 2.68 | 2.03 | 2.65 | 5.09 | 7.76 | 7.46 | 7.53 | 7.46 | 7.19 |
| ILMN_1664776 | EFR3A | 2.68 | 2.04 | 3.57 | 3.05 | 9.28 | 8.91 | 9.00 | 8.79 | 8.86 |
| ILMN_1688335 | MAGEB2 | 2.68 | 2.26 | 2.64 | 7.25 | 10.57 | 10.36 | 10.39 | 10.36 | 10.00 |
| ILMN_1677292 | C5orf30 | 2.68 | 1.89 | 3.09 | 5.08 | 7.96 | 7.68 | 7.76 | 7.64 | 7.43 |
| ILMN_3304042 | LOC729051 | 2.69 | 2.85 | 3.83 | 3.83 | 7.46 | 7.03 | 7.00 | 6.84 | 6.84 |
| ILMN_1788841 | TCL1A | 2.69 | 1.68 | 2.75 | 5.35 | 12.60 | 12.29 | 12.41 | 12.29 | 11.99 |
| ILMN_1743299 | PXMP3 | 2.69 | 1.43 | 3.74 | 3.74 | 8.16 | 7.91 | 8.03 | 7.81 | 7.81 |
| ILMN_1757995 | PARP2 | 2.69 | 1.75 | 3.70 | 3.40 | 9.23 | 8.95 | 9.05 | 8.85 | 8.88 |
| ILMN_1698007 | LOC651872 | 2.69 | 1.15 | 2.39 | 3.19 | 6.93 | 6.75 | 6.86 | 6.77 | 6.72 |
| ILMN_1662158 | LOC653147 | 2.70 | 4.11 | 3.89 | 3.11 | 9.25 | 8.96 | 8.81 | 8.84 | 8.92 |
| ILMN_1772814 | NLK | 2.70 | 2.94 | 3.58 | 5.50 | 7.90 | 7.63 | 7.61 | 7.55 | 7.36 |
| ILMN_3239946 | LOC729375 | 2.70 | 2.78 | 2.16 | 5.03 | 8.14 | 7.92 | 7.92 | 7.97 | 7.74 |
| ILMN_1694177 | PCNA | 2.70 | 0.05 | 1.99 | 3.87 | 11.90 | 11.72 | 11.90 | 11.77 | 11.65 |
| ILMN_2388155 | CASP3 | 2.70 | 1.33 | 3.95 | 4.11 | 9.91 | 9.68 | 9.80 | 9.58 | 9.57 |
| ILMN_1743396 | ACOX3 | 2.70 | -0.22 | 1.94 | 4.58 | 7.91 | 7.63 | 7.93 | 7.71 | 7.43 |
| ILMN_2124155 | ATP11B | 2.71 | 3.08 | 0.99 | 5.79 | 8.86 | 8.64 | 8.61 | 8.78 | 8.39 |
| ILMN_1770811 | PELO | 2.71 | 3.73 | 3.86 | 6.16 | 9.41 | 9.13 | 9.02 | 9.01 | 8.77 |
| ILMN_1721605 | SMYD2 | 2.71 | 1.70 | 2.88 | 3.36 | 8.32 | 8.05 | 8.15 | 8.03 | 7.99 |
| ILMN_2374449 | SPP1 | 2.72 | 3.86 | 2.48 | 6.46 | 8.90 | 8.44 | 8.25 | 8.48 | 7.81 |
| ILMN_1789095 | BMPR2 | 2.72 | 3.12 | 3.81 | 4.33 | 7.25 | 6.94 | 6.89 | 6.81 | 6.75 |
| ILMN_1777286 | LOC388344 | 2.72 | 1.21 | 1.13 | 8.37 | 10.14 | 9.81 | 9.99 | 10.00 | 9.12 |
| ILMN_3290136 | LOC643733 | 2.73 | 3.40 | 3.53 | 4.29 | 7.00 | 6.79 | 6.74 | 6.73 | 6.68 |
| ILMN_3261278 | C18orf18 | 2.73 | 0.34 | 1.83 | 2.86 | 7.59 | 7.30 | 7.56 | 7.40 | 7.29 |
| ILMN_3233272 | MGC23284 | 2.73 | 1.27 | 1.78 | 2.86 | 6.87 | 6.73 | 6.81 | 6.78 | 6.72 |
| ILMN_1783131 | SAMD13 | 2.74 | 2.31 | 2.52 | 4.83 | 7.20 | 6.99 | 7.02 | 7.00 | 6.82 |
| ILMN_2246882 | SP140 | 2.74 | 3.40 | 4.87 | 5.44 | 8.57 | 7.81 | 7.62 | 7.21 | 7.06 |
| ILMN_1782439 | CNN3 | 2.74 | 1.12 | 2.62 | 6.36 | 8.95 | 8.25 | 8.66 | 8.28 | 7.32 |
| ILMN_2178088 | ZNF131 | 2.74 | 2.91 | 3.07 | 3.20 | 7.99 | 7.77 | 7.75 | 7.74 | 7.73 |
| ILMN_1680673 | NT5DC1 | 2.75 | 2.48 | 2.85 | 3.81 | 8.43 | 8.15 | 8.18 | 8.14 | 8.04 |
| ILMN_2247572 | SSX1 | 2.75 | 3.50 | 3.86 | 5.12 | 9.77 | 8.93 | 8.70 | 8.59 | 8.20 |
| ILMN_1758674 | TMEM93 | 2.75 | 3.56 | 5.41 | 2.52 | 10.89 | 10.69 | 10.63 | 10.49 | 10.70 |
| ILMN_2355463 | CYFIP1 | 2.75 | 2.10 | 2.18 | 4.19 | 6.98 | 6.77 | 6.82 | 6.81 | 6.66 |
| ILMN_2413278 | RPL13 | 2.75 | 2.79 | 1.41 | 9.84 | 10.72 | 10.45 | 10.45 | 10.58 | 9.76 |
| ILMN_1775269 | EID1 | 2.76 | 2.06 | 3.97 | 4.37 | 7.63 | 7.38 | 7.45 | 7.27 | 7.24 |
| ILMN_2158336 | SH3GLB2 | 2.76 | 0.36 | 1.67 | 4.73 | 10.14 | 9.86 | 10.11 | 9.97 | 9.66 |
| ILMN_2390299 | PSMB8 | 2.76 | 1.99 | 2.47 | 3.91 | 11.01 | 10.49 | 10.64 | 10.55 | 10.28 |
| ILMN_2315979 | LBH | 2.76 | 1.29 | 1.06 | 5.17 | 10.06 | 9.61 | 9.85 | 9.89 | 9.22 |
| ILMN_1659273 | LOC441408 | 2.76 | 2.85 | 4.10 | 4.51 | 9.58 | 9.18 | 9.16 | 8.98 | 8.92 |
| ILMN_1763730 | APPL1 | 2.77 | 1.74 | 2.29 | 3.21 | 8.56 | 8.29 | 8.39 | 8.34 | 8.25 |
| ILMN_1679025 | LOC641848 | 2.77 | 1.73 | 2.79 | 6.66 | 11.83 | 11.40 | 11.56 | 11.40 | 10.80 |
| ILMN_3307729 | CXXC5 | 2.77 | 1.88 | 2.06 | 4.43 | 11.98 | 11.65 | 11.76 | 11.74 | 11.46 |
| ILMN_1778371 | CCBL2 | 2.77 | 1.41 | 2.94 | 2.77 | 8.80 | 8.58 | 8.69 | 8.56 | 8.58 |
| ILMN_3247723 | NOP16 | 2.77 | 4.75 | 4.78 | 0.61 | 9.40 | 9.10 | 8.88 | 8.88 | 9.33 |
| ILMN_1676924 | CD247 | 2.77 | 1.90 | 2.58 | 3.43 | 11.00 | 10.66 | 10.76 | 10.68 | 10.58 |
| ILMN_1679797 | ADARB1 | 2.77 | -0.18 | 2.85 | 4.27 | 8.81 | 8.36 | 8.84 | 8.34 | 8.11 |
| ILMN_1747223 | FRYL | 2.78 | 2.35 | 1.62 | 4.24 | 8.63 | 8.39 | 8.42 | 8.49 | 8.26 |
| ILMN_1771264 | ELL3 | 2.78 | 3.37 | 1.28 | 3.30 | 9.80 | 9.36 | 9.27 | 9.60 | 9.28 |
| ILMN_1771238 | CHM | 2.78 | 2.21 | 3.85 | 5.79 | 8.52 | 8.28 | 8.33 | 8.18 | 8.01 |
| ILMN_2346997 | RAB23 | 2.78 | 1.80 | 2.00 | 4.81 | 8.81 | 8.49 | 8.60 | 8.58 | 8.25 |
| ILMN_2312386 | PAIP1 | 2.78 | 1.19 | 2.78 | 3.75 | 8.38 | 8.13 | 8.27 | 8.13 | 8.04 |
| ILMN_1763147 | NDUFB6 | 2.79 | 3.50 | 4.34 | 0.68 | 10.65 | 10.42 | 10.36 | 10.29 | 10.60 |
| ILMN_1812096 | CADM4 | 2.79 | 1.86 | 1.56 | 3.81 | 7.16 | 6.97 | 7.04 | 7.06 | 6.90 |
| ILMN_1711166 | WDR8 | 2.79 | 0.15 | 1.03 | -3.71 | 8.94 | 8.69 | 8.93 | 8.85 | 9.28 |
| ILMN_1673360 | BDH2 | 2.80 | 2.14 | 3.12 | 4.60 | 6.83 | 6.66 | 6.70 | 6.64 | 6.55 |
| ILMN_1745447 | HIVEP2 | 2.80 | 0.34 | 0.97 | 3.48 | 7.69 | 7.47 | 7.66 | 7.61 | 7.41 |
| ILMN_3288268 | LOC100132499 | 2.80 | 1.74 | 1.71 | 3.30 | 11.83 | 11.53 | 11.64 | 11.64 | 11.47 |
| ILMN_1772722 | MRPS33 | 2.80 | 3.13 | 3.90 | -0.18 | 8.27 | 7.96 | 7.93 | 7.84 | 8.29 |
| ILMN_3227440 | LOC729051 | 2.80 | 2.33 | 3.92 | 4.36 | 7.51 | 7.15 | 7.21 | 7.01 | 6.95 |
| ILMN_1673543 | PGM2 | 2.80 | 2.80 | 3.71 | 4.33 | 8.57 | 8.34 | 8.34 | 8.27 | 8.22 |
| ILMN_1704431 | LOC554203 | 2.80 | 2.22 | 1.99 | 3.03 | 8.04 | 7.83 | 7.87 | 7.89 | 7.81 |
| ILMN_1677906 | LOC643287 | 2.81 | 2.43 | 2.56 | 4.53 | 11.08 | 10.74 | 10.78 | 10.77 | 10.52 |
| ILMN_2062524 | RBBP4 | 2.81 | 1.51 | 2.97 | 5.12 | 9.45 | 9.11 | 9.26 | 9.09 | 8.83 |
| ILMN_3294126 | LOC100131160 | 2.81 | 1.94 | 1.08 | 4.02 | 8.69 | 8.34 | 8.45 | 8.55 | 8.19 |
| ILMN_1696670 | LOC92497 | 2.81 | 1.20 | 3.47 | 5.04 | 7.51 | 7.28 | 7.41 | 7.23 | 7.10 |
| ILMN_3307858 | STIM2 | 2.81 | 1.28 | 2.49 | 4.31 | 7.92 | 7.67 | 7.81 | 7.70 | 7.53 |
| ILMN_1798061 | ZFYVE26 | 2.82 | 2.18 | 4.20 | 3.88 | 8.83 | 8.50 | 8.57 | 8.34 | 8.38 |
| ILMN_1710326 | CLDND1 | 2.82 | 2.57 | 1.75 | 3.78 | 9.38 | 9.12 | 9.14 | 9.22 | 9.03 |
| ILMN_2233539 | SLC39A8 | 2.82 | 1.89 | 1.39 | 4.74 | 9.14 | 8.84 | 8.94 | 8.99 | 8.63 |
| ILMN_1705685 | MEIS1 | 2.82 | 0.97 | 1.82 | 3.99 | 7.60 | 7.27 | 7.48 | 7.38 | 7.13 |
| ILMN_1730685 | MRPL16 | 2.82 | 2.90 | 4.56 | 3.90 | 10.55 | 10.32 | 10.32 | 10.18 | 10.24 |
| ILMN_1671265 | ING2 | 2.82 | 1.27 | 0.32 | 4.54 | 8.44 | 8.11 | 8.29 | 8.40 | 7.91 |
| ILMN_1789094 | NINL | 2.82 | 1.22 | 2.01 | 4.67 | 7.48 | 7.11 | 7.32 | 7.21 | 6.86 |
| ILMN_3225432 | MAPKSP1 | 2.82 | 1.61 | 3.23 | 3.11 | 7.16 | 7.00 | 7.07 | 6.98 | 6.98 |
| ILMN_1808661 | TOMM5 | 2.83 | 3.90 | 4.33 | 4.22 | 10.96 | 10.69 | 10.59 | 10.55 | 10.56 |
| ILMN_1678170 | MME | 2.83 | 0.90 | 2.86 | 2.83 | 9.31 | 8.71 | 9.12 | 8.70 | 8.71 |
| ILMN_1721559 | FABP6 | 2.83 | 2.69 | 3.80 | 3.66 | 8.36 | 7.96 | 7.98 | 7.83 | 7.85 |
| ILMN_1815154 | MYH10 | 2.84 | 2.48 | 4.37 | 5.16 | 9.68 | 9.26 | 9.31 | 9.03 | 8.91 |
| ILMN_2116661 | PRPSAP2 | 2.84 | 2.07 | 3.56 | 3.98 | 9.34 | 8.98 | 9.08 | 8.89 | 8.84 |
| ILMN_1756826 | MORN2 | 2.84 | 1.56 | 4.36 | 2.75 | 7.25 | 7.05 | 7.14 | 6.94 | 7.06 |
| ILMN_1695316 | SLC39A8 | 2.85 | 1.07 | 0.96 | 3.50 | 8.45 | 8.11 | 8.32 | 8.34 | 8.03 |
| ILMN_1750507 | RPL9 | 2.85 | 3.14 | 2.79 | 3.06 | 14.09 | 13.77 | 13.74 | 13.78 | 13.75 |
| ILMN_2222991 | ETF1 | 2.86 | 1.88 | 2.69 | 3.49 | 8.91 | 8.63 | 8.73 | 8.65 | 8.57 |
| ILMN_1777449 | IFT74 | 2.86 | 2.86 | 3.38 | 4.27 | 7.24 | 7.00 | 7.00 | 6.96 | 6.88 |
| ILMN_3203098 | LOC400836 | 2.86 | 2.41 | 2.08 | 3.29 | 7.36 | 7.06 | 7.11 | 7.14 | 7.02 |
| ILMN_2299045 | BMP2K | 2.87 | 2.54 | 3.61 | 6.31 | 8.06 | 7.77 | 7.80 | 7.69 | 7.41 |
| ILMN_1760441 | MRPS5 | 2.87 | 0.84 | 2.15 | 4.13 | 10.00 | 9.78 | 9.94 | 9.83 | 9.68 |
| ILMN_1658494 | C13orf15 | 2.87 | 3.85 | 4.76 | 5.89 | 9.81 | 8.78 | 8.43 | 8.10 | 7.70 |
| ILMN_3213640 | LOC100132444 | 2.87 | 0.00 | 0.71 | 3.67 | 7.87 | 7.67 | 7.87 | 7.82 | 7.61 |
| ILMN_1794597 | ZNF167 | 2.87 | 0.66 | 2.91 | 3.84 | 7.13 | 6.91 | 7.08 | 6.91 | 6.84 |
| ILMN_3188076 | LOC100128060 | 2.87 | 1.77 | 2.99 | 4.37 | 11.88 | 11.32 | 11.53 | 11.29 | 11.02 |
| ILMN_2376553 | SLC23A1 | 2.87 | 1.06 | 3.05 | 3.31 | 7.00 | 6.78 | 6.92 | 6.77 | 6.75 |
| ILMN_2080751 | ADNP2 | 2.87 | 1.17 | 1.96 | 5.24 | 9.04 | 8.64 | 8.88 | 8.77 | 8.31 |
| ILMN_1671045 | LOC439992 | 2.87 | 1.75 | 1.57 | 3.81 | 9.34 | 8.81 | 9.01 | 9.05 | 8.63 |
| ILMN_1685446 | NARG1L | 2.87 | 0.76 | 1.75 | 5.05 | 7.58 | 7.29 | 7.50 | 7.40 | 7.07 |
| ILMN_2215043 | RNF32 | 2.88 | 2.66 | 3.53 | 4.18 | 7.50 | 7.28 | 7.30 | 7.23 | 7.18 |
| ILMN_2174127 | DCBLD2 | 2.88 | -1.08 | 1.33 | 5.73 | 8.59 | 8.24 | 8.72 | 8.43 | 7.90 |
| ILMN_1846306 |  | 2.88 | 3.38 | 4.47 | 7.02 | 7.72 | 7.43 | 7.38 | 7.27 | 7.01 |
| ILMN_1806908 | PRKCB1 | 2.88 | 2.02 | 2.69 | 5.94 | 11.67 | 11.32 | 11.43 | 11.35 | 10.96 |
| ILMN_2352563 | CLDND1 | 2.89 | 1.68 | 1.08 | 3.58 | 9.54 | 9.31 | 9.41 | 9.45 | 9.26 |
| ILMN_1768480 | VGLL4 | 2.89 | -0.68 | 0.78 | 5.35 | 8.58 | 8.31 | 8.65 | 8.51 | 8.08 |
| ILMN_1743205 | ABCA7 | 2.89 | -0.66 | 2.23 | -3.45 | 8.03 | 7.63 | 8.12 | 7.72 | 8.50 |
| ILMN_1784436 | KIAA1688 | 2.89 | 1.45 | 3.60 | 4.53 | 7.77 | 7.42 | 7.60 | 7.33 | 7.22 |
| ILMN_1792986 | RFC1 | 2.89 | -0.12 | 2.31 | 3.55 | 7.81 | 7.58 | 7.82 | 7.62 | 7.52 |
| ILMN_2070896 | BMPR2 | 2.90 | 2.69 | 3.55 | 4.33 | 7.26 | 6.89 | 6.91 | 6.80 | 6.70 |
| ILMN_1708143 | FAM127A | 2.90 | 2.19 | 2.12 | 4.48 | 7.88 | 7.60 | 7.67 | 7.67 | 7.44 |
| ILMN_1810423 | RPP40 | 2.90 | 3.01 | 4.42 | 2.44 | 10.75 | 10.41 | 10.40 | 10.24 | 10.47 |
| ILMN_1686664 | MT2A | 2.90 | 0.38 | 5.73 | 5.17 | 8.30 | 7.74 | 8.23 | 7.20 | 7.30 |
| ILMN_1780799 | ENPP2 | 2.90 | 3.28 | 3.08 | 7.90 | 9.05 | 8.47 | 8.39 | 8.43 | 7.46 |
| ILMN_2080760 | SNX22 | 2.90 | 0.59 | 0.82 | 3.08 | 7.93 | 7.72 | 7.89 | 7.87 | 7.71 |
| ILMN_2399431 | PRPS2 | 2.90 | 3.09 | 5.29 | 3.26 | 9.79 | 9.49 | 9.47 | 9.24 | 9.45 |
| ILMN_1677043 | AKR7A2 | 2.90 | 0.62 | 1.79 | 3.20 | 11.09 | 10.76 | 11.02 | 10.89 | 10.73 |
| ILMN_3279927 | LOC100131675 | 2.90 | 1.83 | 1.67 | 2.84 | 8.19 | 7.88 | 7.99 | 8.01 | 7.89 |
| ILMN_3252395 | LOC100129934 | 2.91 | 2.36 | 2.86 | 4.82 | 8.73 | 8.32 | 8.40 | 8.33 | 8.05 |
| ILMN_1738604 | ADH5 | 2.91 | 1.86 | 1.72 | 2.96 | 7.19 | 6.99 | 7.06 | 7.07 | 6.98 |
| ILMN_1747857 | SMARCE1 | 2.91 | 2.88 | 4.55 | 4.00 | 9.48 | 9.16 | 9.17 | 8.98 | 9.04 |
| ILMN_1739397 | GLMN | 2.91 | 1.81 | 3.95 | -0.27 | 8.49 | 8.17 | 8.29 | 8.05 | 8.52 |
| ILMN_1815519 | EPN2 | 2.92 | 0.94 | 1.86 | 2.80 | 7.41 | 7.08 | 7.30 | 7.20 | 7.09 |
| ILMN_1720311 | SLC25A46 | 2.92 | 3.12 | 3.37 | 3.44 | 9.78 | 9.50 | 9.48 | 9.45 | 9.45 |
| ILMN_2194467 | SGCB | 2.92 | 0.80 | 0.55 | 3.81 | 7.16 | 6.93 | 7.10 | 7.12 | 6.86 |
| ILMN_1706051 | PLD5 | 2.92 | 3.72 | 5.02 | 6.38 | 7.73 | 7.30 | 7.18 | 6.98 | 6.78 |
| ILMN_1680770 | UBQLN2 | 2.92 | 0.37 | 1.63 | 3.54 | 9.76 | 9.44 | 9.72 | 9.58 | 9.37 |
| ILMN_2174369 | ELOVL5 | 2.93 | 2.57 | -0.18 | 4.42 | 9.53 | 9.20 | 9.24 | 9.55 | 9.04 |
| ILMN_1691395 | ZBTB11 | 2.93 | 3.01 | 3.94 | 2.79 | 8.17 | 7.90 | 7.89 | 7.81 | 7.91 |
| ILMN_1737981 | FSTL5 | 2.94 | 0.68 | 1.22 | 6.85 | 10.06 | 9.22 | 9.86 | 9.71 | 8.11 |
| ILMN_3300640 | MGC26356 | 2.94 | 2.66 | 2.28 | 3.59 | 7.36 | 7.08 | 7.11 | 7.14 | 7.02 |
| ILMN_2370414 | CHD3 | 2.94 | 1.77 | 2.25 | 3.16 | 7.00 | 6.78 | 6.87 | 6.83 | 6.76 |
| ILMN_2409642 | TRO | 2.95 | 1.26 | 3.79 | 6.73 | 8.14 | 7.79 | 7.99 | 7.69 | 7.34 |
| ILMN_1782543 | EEF1D | 2.95 | 2.10 | 2.89 | 3.92 | 10.07 | 9.76 | 9.85 | 9.77 | 9.66 |
| ILMN_1714167 | CYB5A | 2.95 | 0.31 | 2.29 | 3.81 | 9.66 | 9.41 | 9.64 | 9.47 | 9.34 |
| ILMN_1764096 | CCBL1 | 2.96 | 1.75 | 3.83 | 4.47 | 8.59 | 8.30 | 8.42 | 8.21 | 8.15 |
| ILMN_1807515 | CSTF2T | 2.96 | 0.58 | 2.74 | 6.06 | 8.27 | 8.04 | 8.22 | 8.06 | 7.81 |
| ILMN_1674367 | SPRR2F | 2.96 | 1.70 | 3.19 | -5.34 | 6.83 | 6.57 | 6.68 | 6.55 | 7.31 |
| ILMN_2322346 | SSX4 | 2.96 | 3.23 | 2.14 | 6.54 | 10.32 | 9.95 | 9.91 | 10.05 | 9.50 |
| ILMN_1710303 | TTC25 | 2.97 | 2.77 | 3.64 | 3.18 | 6.90 | 6.71 | 6.72 | 6.66 | 6.69 |
| ILMN_1735347 | MCEE | 2.98 | 2.91 | 2.48 | 4.19 | 9.55 | 9.26 | 9.26 | 9.31 | 9.14 |
| ILMN_1778488 | WDR41 | 2.98 | 3.42 | 5.05 | 11.93 | 9.26 | 9.05 | 9.02 | 8.91 | 8.43 |
| ILMN_1762262 | PKIA | 2.99 | 1.76 | 2.46 | 9.10 | 7.81 | 7.53 | 7.65 | 7.58 | 6.95 |
| ILMN_1775441 | ARHGAP24 | 2.99 | 1.91 | 2.32 | 5.26 | 7.07 | 6.87 | 6.94 | 6.92 | 6.73 |
| ILMN_1911042 |  | 2.99 | 2.77 | 2.74 | 4.98 | 10.77 | 10.37 | 10.40 | 10.40 | 10.11 |
| ILMN_1797822 | SEL1L3 | 3.00 | 3.67 | 5.43 | 5.96 | 10.04 | 9.40 | 9.26 | 8.89 | 8.78 |
| ILMN_1719906 | HADH | 3.00 | 2.39 | 4.11 | 11.82 | 11.41 | 11.11 | 11.17 | 11.00 | 10.24 |
| ILMN_3308158 | MIR330 | 3.00 | 3.43 | 4.70 | 6.36 | 7.71 | 7.43 | 7.39 | 7.28 | 7.12 |
| ILMN_1701837 | KLHL2 | 3.00 | 0.71 | 2.25 | 4.17 | 8.03 | 7.79 | 7.97 | 7.85 | 7.70 |
| ILMN_1730931 | RUFY3 | 3.02 | 1.69 | 1.76 | 5.27 | 7.70 | 7.37 | 7.52 | 7.51 | 7.13 |
| ILMN_1737517 | RPL29 | 3.02 | 3.74 | 4.16 | 2.34 | 9.29 | 8.82 | 8.70 | 8.64 | 8.92 |
| ILMN_2274199 | SUPT3H | 3.03 | 1.97 | 1.56 | 6.76 | 7.41 | 7.21 | 7.28 | 7.31 | 6.97 |
| ILMN_2064917 | AGGF1 | 3.03 | 2.26 | 4.07 | 2.77 | 9.61 | 9.25 | 9.34 | 9.13 | 9.28 |
| ILMN_1801124 | KIAA1826 | 3.03 | 3.29 | 5.41 | 4.41 | 9.36 | 9.05 | 9.02 | 8.80 | 8.90 |
| ILMN_1805812 | TOR1A | 3.03 | -0.60 | 0.64 | -3.58 | 8.69 | 8.45 | 8.74 | 8.64 | 8.97 |
| ILMN_2285996 | KIAA0101 | 3.03 | 1.45 | 5.96 | 4.82 | 10.76 | 10.47 | 10.62 | 10.19 | 10.30 |
| ILMN_1777366 | TTC35 | 3.03 | 3.67 | 3.96 | 3.92 | 7.41 | 7.17 | 7.12 | 7.09 | 7.10 |
| ILMN_2300396 | COMMD5 | 3.03 | 1.23 | 3.46 | 3.08 | 8.69 | 8.48 | 8.60 | 8.45 | 8.47 |
| ILMN_1718672 | NHP2 | 3.04 | 1.68 | 2.36 | 3.69 | 11.26 | 10.97 | 11.10 | 11.04 | 10.91 |
| ILMN_2390338 | UBE2E3 | 3.04 | 0.74 | 1.17 | 5.32 | 10.50 | 10.20 | 10.43 | 10.38 | 9.97 |
| ILMN_1661039 | MRPL30 | 3.05 | 1.50 | 3.10 | 4.26 | 7.63 | 7.42 | 7.53 | 7.42 | 7.34 |
| ILMN_1813091 | ARL1 | 3.05 | 1.65 | 4.86 | 0.82 | 8.64 | 8.40 | 8.51 | 8.25 | 8.58 |
| ILMN_1811387 | TFF3 | 3.05 | 1.65 | 2.62 | 4.42 | 11.84 | 11.18 | 11.48 | 11.27 | 10.88 |
| ILMN_1696270 | PLAG1 | 3.05 | 0.72 | 3.05 | 2.83 | 7.73 | 7.50 | 7.68 | 7.50 | 7.52 |
| ILMN_1783276 | NEXN | 3.05 | 1.48 | 1.58 | 3.84 | 7.06 | 6.86 | 6.96 | 6.96 | 6.80 |
| ILMN_3227992 | LOC729332 | 3.07 | 2.73 | 3.68 | 3.19 | 7.89 | 7.56 | 7.60 | 7.49 | 7.55 |
| ILMN_1703074 | CPD | 3.07 | 1.63 | 3.48 | 4.70 | 7.69 | 7.42 | 7.55 | 7.38 | 7.28 |
| ILMN_1737283 | MGC39900 | 3.07 | 3.62 | 3.13 | 7.83 | 9.26 | 8.91 | 8.85 | 8.90 | 8.37 |
| ILMN_2293992 | RAB28 | 3.07 | 1.64 | 1.52 | 4.43 | 7.50 | 7.25 | 7.37 | 7.38 | 7.13 |
| ILMN_1782688 | THNSL1 | 3.07 | 2.24 | 2.16 | 4.23 | 7.61 | 7.35 | 7.42 | 7.43 | 7.25 |
| ILMN_3266944 | LOC100129599 | 3.07 | 1.51 | 2.11 | 3.14 | 10.98 | 10.66 | 10.83 | 10.76 | 10.66 |
| ILMN_1777998 | ARHGAP25 | 3.08 | 3.27 | 4.74 | 2.88 | 9.27 | 8.95 | 8.93 | 8.77 | 8.97 |
| ILMN_1703379 | VPS41 | 3.08 | 3.20 | 3.12 | 6.43 | 10.08 | 9.81 | 9.80 | 9.81 | 9.53 |
| ILMN_1653666 | KLHL7 | 3.08 | 2.26 | 2.74 | 3.08 | 7.09 | 6.81 | 6.89 | 6.84 | 6.81 |
| ILMN_1681269 | DYRK4 | 3.08 | 2.43 | 2.95 | 3.41 | 10.20 | 9.88 | 9.95 | 9.89 | 9.85 |
| ILMN_3280020 | LOC441506 | 3.09 | 1.31 | 2.95 | 4.50 | 10.85 | 10.24 | 10.59 | 10.27 | 9.97 |
| ILMN_3236135 | FAM86D | 3.10 | 0.80 | 2.76 | 4.53 | 7.38 | 7.20 | 7.34 | 7.22 | 7.12 |
| ILMN_2224103 | PAPSS1 | 3.10 | 3.39 | 2.04 | 11.83 | 9.92 | 9.56 | 9.53 | 9.69 | 8.55 |
| ILMN_1678808 | KIAA0831 | 3.12 | 0.03 | 4.08 | 1.19 | 8.71 | 8.39 | 8.70 | 8.30 | 8.59 |
| ILMN_1651237 | CDT1 | 3.12 | -0.26 | 2.14 | 8.28 | 9.19 | 8.76 | 9.23 | 8.89 | 8.03 |
| ILMN_3246097 | LOC649839 | 3.12 | 2.80 | 2.77 | 6.05 | 9.64 | 9.25 | 9.29 | 9.30 | 8.89 |
| ILMN_2098325 | C8orf33 | 3.13 | 0.89 | 3.16 | 4.44 | 9.91 | 9.59 | 9.82 | 9.59 | 9.46 |
| ILMN_2402303 | SSX4B | 3.13 | 3.49 | 3.52 | 4.69 | 9.70 | 9.33 | 9.28 | 9.28 | 9.14 |
| ILMN_1708991 | CCNY | 3.13 | 0.30 | 2.53 | 5.13 | 8.17 | 7.90 | 8.15 | 7.95 | 7.72 |
| ILMN_1811957 | CAMSAP1 | 3.14 | 1.35 | 1.87 | 4.00 | 8.51 | 8.23 | 8.39 | 8.34 | 8.15 |
| ILMN_1691425 | LDOC1L | 3.15 | -0.20 | 0.33 | 3.34 | 8.08 | 7.76 | 8.10 | 8.05 | 7.74 |
| ILMN_2159322 | SLK | 3.15 | 1.54 | 3.32 | 4.05 | 7.88 | 7.46 | 7.68 | 7.44 | 7.34 |
| ILMN_1741105 | LOC654085 | 3.15 | 1.47 | 2.64 | 4.53 | 7.38 | 7.13 | 7.26 | 7.17 | 7.02 |
| ILMN_3247059 | HYALP1 | 3.15 | 1.36 | 1.87 | 3.48 | 7.35 | 7.06 | 7.23 | 7.18 | 7.03 |
| ILMN_2105441 | IGJ | 3.15 | 3.02 | 5.07 | 8.50 | 10.94 | 9.85 | 9.89 | 9.18 | 8.00 |
| ILMN_2059505 | ARPP19 | 3.15 | 3.47 | 1.68 | 8.19 | 10.93 | 10.73 | 10.71 | 10.82 | 10.41 |
| ILMN_2392352 | CTPS2 | 3.15 | 1.37 | 2.63 | 7.48 | 9.35 | 9.09 | 9.24 | 9.14 | 8.74 |
| ILMN_2096405 | WDR37 | 3.15 | 0.20 | 0.07 | 3.42 | 8.79 | 8.47 | 8.77 | 8.78 | 8.44 |
| ILMN_1691290 | CELSR3 | 3.15 | 1.39 | 4.02 | 0.22 | 8.16 | 7.64 | 7.93 | 7.50 | 8.12 |
| ILMN_1693221 | CENPH | 3.16 | 2.29 | 4.27 | 4.51 | 8.32 | 8.01 | 8.10 | 7.91 | 7.88 |
| ILMN_1696615 | CA8 | 3.16 | 3.39 | 4.74 | 7.10 | 7.37 | 7.00 | 6.98 | 6.82 | 6.55 |
| ILMN_1758104 | PRPS2 | 3.16 | 2.13 | 3.47 | 2.85 | 9.31 | 9.01 | 9.11 | 8.98 | 9.04 |
| ILMN_1842286 |  | 3.17 | 3.07 | 3.35 | 6.72 | 8.14 | 7.62 | 7.64 | 7.59 | 7.03 |
| ILMN_1652434 | MTHFD2L | 3.18 | 2.67 | 3.03 | 5.63 | 8.59 | 8.15 | 8.22 | 8.17 | 7.81 |
| ILMN_2210601 | RNASEL | 3.18 | 4.06 | 4.48 | 3.22 | 8.03 | 7.76 | 7.68 | 7.64 | 7.75 |
| ILMN_2325506 | BCAS4 | 3.18 | 2.75 | 0.56 | -3.22 | 9.97 | 9.63 | 9.68 | 9.91 | 10.32 |
| ILMN_2153373 | LRBA | 3.19 | 2.53 | 3.76 | 7.55 | 8.39 | 8.02 | 8.10 | 7.95 | 7.51 |
| ILMN_1804568 | HOMER1 | 3.19 | 2.59 | 3.53 | 7.49 | 7.49 | 7.24 | 7.28 | 7.21 | 6.90 |
| ILMN_2173004 | RAB8B | 3.19 | 2.24 | 4.08 | 3.33 | 8.76 | 8.45 | 8.54 | 8.36 | 8.44 |
| ILMN_1762622 | FAM111B | 3.20 | 2.00 | 4.53 | 4.00 | 7.28 | 6.98 | 7.09 | 6.85 | 6.90 |
| ILMN_1695899 | LOC648659 | 3.20 | 2.20 | 2.89 | 4.40 | 10.37 | 9.86 | 10.02 | 9.91 | 9.66 |
| ILMN_1745900 | LOC641849 | 3.20 | 2.47 | 2.53 | 5.31 | 10.61 | 10.04 | 10.17 | 10.16 | 9.67 |
| ILMN_1771051 | RPL29 | 3.20 | 4.79 | 4.99 | 2.83 | 9.32 | 8.83 | 8.59 | 8.56 | 8.89 |
| ILMN_1733164 | FBXO11 | 3.20 | 0.69 | 2.48 | 3.13 | 8.46 | 8.15 | 8.39 | 8.22 | 8.15 |
| ILMN_2218780 | PPM2C | 3.20 | -0.17 | 1.32 | 6.29 | 7.14 | 6.96 | 7.15 | 7.07 | 6.78 |
| ILMN_1767006 | PSMB8 | 3.20 | 4.63 | 2.99 | 6.44 | 9.41 | 9.11 | 8.98 | 9.13 | 8.81 |
| ILMN_1725594 | FAM188A | 3.20 | 1.11 | 2.51 | 5.59 | 9.53 | 9.27 | 9.44 | 9.33 | 9.08 |
| ILMN_3235221 | LOC644936 | 3.22 | 1.39 | -0.52 | 3.61 | 11.43 | 10.73 | 11.13 | 11.54 | 10.64 |
| ILMN_1693685 | LOC205251 | 3.22 | 3.26 | 3.88 | 1.37 | 7.92 | 7.68 | 7.68 | 7.63 | 7.82 |
| ILMN_2093980 | TMEM38B | 3.22 | 1.27 | 2.49 | 2.79 | 7.00 | 6.82 | 6.93 | 6.86 | 6.85 |
| ILMN_1664136 | LOC644074 | 3.22 | -0.69 | 2.53 | 2.88 | 8.68 | 8.14 | 8.80 | 8.26 | 8.20 |
| ILMN_1694491 | CCNG1 | 3.22 | 3.36 | 3.19 | 3.93 | 10.87 | 10.55 | 10.54 | 10.55 | 10.48 |
| ILMN_2327994 | AZIN1 | 3.22 | 2.46 | 4.13 | 5.38 | 9.04 | 8.76 | 8.83 | 8.68 | 8.57 |
| ILMN_2083066 | IGLL3 | 3.23 | 0.44 | 0.81 | 4.52 | 9.27 | 8.80 | 9.21 | 9.15 | 8.62 |
| ILMN_2261784 | CCNY | 3.23 | -0.70 | 0.43 | 3.56 | 8.96 | 8.56 | 9.05 | 8.91 | 8.52 |
| ILMN_3235472 | WDYHV1 | 3.24 | 2.54 | 4.33 | 4.58 | 8.66 | 8.23 | 8.32 | 8.08 | 8.05 |
| ILMN_1796925 | CXADR | 3.24 | 0.87 | 1.55 | 2.74 | 7.04 | 6.74 | 6.96 | 6.90 | 6.79 |
| ILMN_1809400 | FAM49B | 3.25 | 2.38 | 3.62 | 3.78 | 9.20 | 8.88 | 8.97 | 8.84 | 8.83 |
| ILMN_1750880 | AMN1 | 3.28 | 1.13 | 2.22 | 3.64 | 7.60 | 7.30 | 7.50 | 7.40 | 7.27 |
| ILMN_1706498 | DSE | 3.28 | 3.54 | 4.84 | 6.40 | 7.29 | 7.08 | 7.06 | 6.98 | 6.88 |
| ILMN_3237779 | TMEM184C | 3.29 | 2.98 | 3.04 | 7.57 | 8.66 | 8.30 | 8.34 | 8.33 | 7.84 |
| ILMN_3248882 | KIAA0114 | 3.29 | 2.14 | 3.60 | 4.53 | 11.77 | 11.32 | 11.48 | 11.28 | 11.15 |
| ILMN_1666976 | PLD3 | 3.29 | 2.69 | 0.38 | 3.12 | 7.79 | 7.59 | 7.62 | 7.76 | 7.60 |
| ILMN_1651354 | SPP1 | 3.30 | 4.07 | 2.23 | 10.34 | 9.62 | 9.25 | 9.16 | 9.37 | 8.46 |
| ILMN_1755222 | C9orf82 | 3.30 | 3.34 | 4.25 | 5.16 | 8.56 | 8.25 | 8.24 | 8.16 | 8.07 |
| ILMN_1716596 | NSMAF | 3.30 | -1.04 | 4.29 | 1.70 | 8.81 | 8.58 | 8.89 | 8.51 | 8.69 |
| ILMN_2138435 | MRPS27 | 3.31 | 3.04 | 3.47 | 6.29 | 10.73 | 10.32 | 10.35 | 10.30 | 9.94 |
| ILMN_1767523 | IL17RB | 3.31 | 1.05 | 4.01 | -5.26 | 7.15 | 6.87 | 7.06 | 6.81 | 7.60 |
| ILMN_1697971 | TRIM38 | 3.31 | -0.68 | 1.62 | 3.96 | 7.80 | 7.49 | 7.86 | 7.65 | 7.43 |
| ILMN_2194229 | TMEM128 | 3.31 | 2.20 | 2.12 | 6.51 | 8.64 | 8.36 | 8.46 | 8.46 | 8.10 |
| ILMN_1726448 | MMP1 | 3.32 | 2.37 | 4.14 | 4.31 | 7.04 | 6.78 | 6.85 | 6.72 | 6.70 |
| ILMN_1747195 | PSMB8 | 3.33 | 2.22 | 3.11 | 5.55 | 10.78 | 10.26 | 10.43 | 10.29 | 9.91 |
| ILMN_3213692 | LOC441073 | 3.33 | 4.52 | 5.57 | 4.86 | 9.46 | 8.98 | 8.80 | 8.65 | 8.75 |
| ILMN_2154836 | BTG3 | 3.33 | 2.46 | 4.31 | 4.48 | 8.62 | 8.22 | 8.33 | 8.11 | 8.09 |
| ILMN_3289730 | LOC100132199 | 3.34 | 2.46 | 2.29 | 5.73 | 9.90 | 9.57 | 9.65 | 9.67 | 9.33 |
| ILMN_1808354 | SLC4A7 | 3.34 | 2.43 | 2.82 | 3.90 | 7.03 | 6.77 | 6.84 | 6.81 | 6.73 |
| ILMN_2302654 | LRP8 | 3.34 | 0.77 | 1.92 | 3.94 | 7.06 | 6.86 | 7.02 | 6.95 | 6.82 |
| ILMN_2261076 | NEDD9 | 3.34 | 3.87 | 4.75 | 5.81 | 7.69 | 7.40 | 7.35 | 7.28 | 7.18 |
| ILMN_3237645 | LRTOMT | 3.34 | 1.19 | 1.76 | 6.47 | 7.56 | 7.31 | 7.47 | 7.43 | 7.07 |
| ILMN_1806306 | SV2B | 3.35 | 1.49 | 1.49 | 3.20 | 7.58 | 7.28 | 7.45 | 7.45 | 7.30 |
| ILMN_1675085 | UBA6 | 3.35 | 1.67 | 2.04 | 7.22 | 10.00 | 9.64 | 9.82 | 9.78 | 9.22 |
| ILMN_1741954 | SMYD3 | 3.36 | 2.59 | 1.07 | 4.88 | 9.58 | 9.29 | 9.36 | 9.49 | 9.16 |
| ILMN_1803801 | LRRC38 | 3.36 | 2.79 | 2.46 | 4.97 | 7.77 | 7.32 | 7.40 | 7.44 | 7.10 |
| ILMN_1740512 | MGC39900 | 3.36 | 2.62 | 2.38 | 7.81 | 10.48 | 10.05 | 10.15 | 10.18 | 9.49 |
| ILMN_2081988 | LANCL1 | 3.37 | 2.37 | 5.73 | 3.88 | 10.22 | 9.85 | 9.96 | 9.59 | 9.80 |
| ILMN_1704210 | CNTNAP4 | 3.37 | 2.05 | 3.08 | 4.64 | 6.96 | 6.73 | 6.82 | 6.75 | 6.64 |
| ILMN_1657679 | VAV3 | 3.37 | 1.00 | 3.85 | -1.90 | 7.50 | 7.28 | 7.43 | 7.25 | 7.62 |
| ILMN_1666757 | SDHALP1 | 3.38 | 2.90 | 5.19 | 5.52 | 8.09 | 7.86 | 7.89 | 7.73 | 7.71 |
| ILMN_1741003 | ANXA5 | 3.38 | 2.58 | 3.50 | 9.73 | 10.28 | 9.99 | 10.06 | 9.98 | 9.44 |
| ILMN_2045419 | BNIP3L | 3.40 | 1.35 | 2.11 | 5.38 | 8.37 | 8.03 | 8.24 | 8.16 | 7.83 |
| ILMN_2352131 | ERBB2 | 3.40 | 3.11 | 5.08 | 2.61 | 7.42 | 7.19 | 7.21 | 7.08 | 7.25 |
| ILMN_1758827 | RTN4IP1 | 3.42 | 2.85 | 4.15 | 5.36 | 9.71 | 9.45 | 9.49 | 9.39 | 9.30 |
| ILMN_1731640 | MGC39900 | 3.42 | 3.58 | 2.75 | 8.11 | 9.26 | 8.83 | 8.81 | 8.92 | 8.24 |
| ILMN_3290385 | LOC401640 | 3.42 | 1.46 | 2.80 | 5.21 | 9.40 | 9.01 | 9.23 | 9.08 | 8.81 |
| ILMN_3307158 | ATG4A | 3.43 | 3.30 | 3.51 | 5.51 | 7.90 | 7.62 | 7.63 | 7.61 | 7.45 |
| ILMN_3246214 | B3GNT1 | 3.43 | 0.59 | 2.54 | -3.47 | 8.33 | 8.06 | 8.28 | 8.13 | 8.60 |
| ILMN_2211018 | SPRR2E | 3.43 | 1.81 | 3.79 | -5.87 | 7.79 | 6.91 | 7.33 | 6.82 | 9.28 |
| ILMN_3247163 | TET1 | 3.43 | 0.19 | 3.98 | 4.34 | 7.73 | 7.38 | 7.71 | 7.32 | 7.28 |
| ILMN_3299520 | PRKCB | 3.43 | 2.58 | 2.41 | 3.61 | 10.47 | 9.94 | 10.07 | 10.10 | 9.92 |
| ILMN_1692026 | SUV420H1 | 3.44 | 3.26 | 4.16 | 6.11 | 8.51 | 8.18 | 8.19 | 8.11 | 7.92 |
| ILMN_1689329 | SCD | 3.44 | 1.44 | 0.00 | 3.40 | 11.34 | 10.74 | 11.09 | 11.34 | 10.75 |
| ILMN_1761411 | C10orf119 | 3.45 | 0.21 | 2.78 | 2.91 | 7.64 | 7.36 | 7.62 | 7.42 | 7.41 |
| ILMN_1703263 | SP140 | 3.45 | 4.51 | 6.55 | 6.81 | 7.99 | 7.38 | 7.20 | 6.84 | 6.79 |
| ILMN_1705928 | SNRNP200 | 3.45 | 1.18 | 3.28 | 4.82 | 7.95 | 7.54 | 7.81 | 7.56 | 7.37 |
| ILMN_1711312 | GPM6A | 3.45 | 1.38 | 3.09 | 3.66 | 8.60 | 7.89 | 8.31 | 7.96 | 7.85 |
| ILMN_2170595 | RRM2B | 3.45 | 1.65 | 4.42 | 5.93 | 8.21 | 7.98 | 8.10 | 7.91 | 7.81 |
| ILMN_2390586 | SP100 | 3.45 | 4.51 | 5.26 | 5.74 | 7.39 | 7.00 | 6.89 | 6.80 | 6.75 |
| ILMN_1798875 | LOC727855 | 3.46 | -0.51 | 1.77 | 2.99 | 8.61 | 8.09 | 8.69 | 8.35 | 8.16 |
| ILMN_2360705 | ACSL3 | 3.46 | 3.95 | 2.68 | 4.40 | 10.16 | 9.67 | 9.60 | 9.78 | 9.53 |
| ILMN_3288529 | LOC645630 | 3.46 | 2.10 | 2.80 | 3.42 | 9.98 | 9.43 | 9.65 | 9.54 | 9.44 |
| ILMN_1727574 | ZNF827 | 3.46 | 1.54 | 3.49 | 4.10 | 8.29 | 7.78 | 8.06 | 7.78 | 7.69 |
| ILMN_1656399 | TCEAL8 | 3.47 | 2.87 | 4.00 | 5.66 | 8.68 | 8.31 | 8.37 | 8.25 | 8.08 |
| ILMN_1653039 | LOC642934 | 3.47 | 1.86 | 5.02 | 5.05 | 10.35 | 10.01 | 10.17 | 9.86 | 9.86 |
| ILMN_1719224 | C17orf45 | 3.47 | 3.43 | 5.21 | 4.41 | 12.69 | 12.11 | 12.12 | 11.82 | 11.96 |
| ILMN_3239435 | HTT | 3.47 | -0.28 | 0.42 | 3.14 | 7.23 | 6.98 | 7.25 | 7.20 | 7.00 |
| ILMN_3200484 | LOC126235 | 3.48 | 3.19 | 3.41 | 3.23 | 8.52 | 8.20 | 8.22 | 8.20 | 8.22 |
| ILMN_2232157 | SLMO1 | 3.48 | -0.43 | 3.84 | 6.08 | 8.88 | 8.56 | 8.92 | 8.52 | 8.31 |
| ILMN_1674231 | CHAF1B | 3.49 | 0.31 | 4.75 | 2.25 | 8.49 | 8.12 | 8.46 | 7.98 | 8.25 |
| ILMN_1683598 | ACSL4 | 3.49 | 1.53 | 0.72 | 5.45 | 9.44 | 9.17 | 9.32 | 9.39 | 9.02 |
| ILMN_1791328 | STK39 | 3.49 | 4.07 | 5.09 | 3.89 | 9.28 | 8.76 | 8.67 | 8.52 | 8.70 |
| ILMN_3237396 | AAGAB | 3.49 | 1.42 | 2.78 | 3.49 | 8.73 | 8.35 | 8.58 | 8.43 | 8.35 |
| ILMN_1690971 | SSX3 | 3.50 | 3.38 | 3.53 | 3.79 | 8.69 | 8.29 | 8.31 | 8.29 | 8.26 |
| ILMN_3235176 | C2orf89 | 3.51 | 3.59 | 2.70 | 3.59 | 6.94 | 6.67 | 6.66 | 6.73 | 6.66 |
| ILMN_2106818 | MBIP | 3.52 | 1.66 | 3.44 | 2.77 | 8.55 | 8.25 | 8.41 | 8.26 | 8.31 |
| ILMN_2111187 | ELOVL6 | 3.53 | 1.79 | 3.83 | 6.75 | 10.42 | 9.70 | 10.05 | 9.64 | 9.05 |
| ILMN_1721978 | CARD11 | 3.55 | 0.20 | 1.03 | 5.39 | 10.58 | 10.09 | 10.55 | 10.44 | 9.84 |
| ILMN_1807662 | IGF2R | 3.55 | 2.13 | 3.68 | 4.53 | 9.32 | 8.93 | 9.09 | 8.92 | 8.82 |
| ILMN_1718832 | SPHAR | 3.56 | 1.80 | 3.60 | 4.72 | 7.97 | 7.73 | 7.85 | 7.72 | 7.65 |
| ILMN_1703279 | CXorf57 | 3.56 | 0.81 | 3.05 | 8.55 | 8.78 | 8.22 | 8.65 | 8.30 | 7.44 |
| ILMN_3282395 | LOC646966 | 3.56 | 4.73 | 5.98 | 5.58 | 9.36 | 8.82 | 8.64 | 8.45 | 8.51 |
| ILMN_3298266 | LOC728774 | 3.56 | 3.36 | 3.60 | 3.00 | 8.95 | 8.60 | 8.62 | 8.59 | 8.65 |
| ILMN_3225843 | LOC729298 | 3.60 | 1.65 | 2.41 | 3.73 | 7.91 | 7.62 | 7.78 | 7.72 | 7.61 |
| ILMN_2405297 | NOTCH2 | 3.61 | 2.59 | 4.03 | -0.43 | 7.84 | 7.47 | 7.57 | 7.43 | 7.88 |
| ILMN_2337974 | PKIA | 3.61 | 2.72 | 3.38 | 12.58 | 8.35 | 8.03 | 8.11 | 8.05 | 7.25 |
| ILMN_3272441 | LOC100129165 | 3.61 | 2.79 | 1.85 | 7.17 | 7.18 | 6.90 | 6.96 | 7.04 | 6.62 |
| ILMN_2085722 | ING2 | 3.61 | 2.09 | 0.76 | 6.21 | 9.21 | 8.87 | 9.01 | 9.14 | 8.63 |
| ILMN_1739236 | ZNF668 | 3.61 | 3.36 | 5.12 | 1.35 | 8.47 | 8.23 | 8.24 | 8.13 | 8.38 |
| ILMN_1808837 | LOC644029 | 3.62 | 2.34 | 2.28 | 5.44 | 10.32 | 9.74 | 9.95 | 9.96 | 9.45 |
| ILMN_3241118 | LOC641727 | 3.62 | 1.35 | 2.63 | 4.83 | 8.80 | 8.46 | 8.67 | 8.55 | 8.35 |
| ILMN_3288218 | LOC644315 | 3.64 | 3.86 | 3.59 | 3.64 | 8.86 | 8.58 | 8.57 | 8.59 | 8.58 |
| ILMN_1812759 | GCH1 | 3.64 | 0.31 | 0.93 | 2.93 | 7.38 | 7.11 | 7.36 | 7.31 | 7.16 |
| ILMN_1701239 | SPRR2E | 3.65 | 2.19 | 3.91 | -5.29 | 7.53 | 6.79 | 7.08 | 6.73 | 8.61 |
| ILMN_2331636 | ACACA | 3.66 | 0.93 | 2.26 | 3.41 | 9.07 | 8.58 | 8.95 | 8.77 | 8.61 |
| ILMN_1665291 | NUB1 | 3.67 | 0.69 | 3.01 | -4.07 | 8.60 | 8.23 | 8.53 | 8.30 | 9.01 |
| ILMN_1677038 | FLJ21986 | 3.68 | 1.70 | 2.82 | 6.63 | 8.76 | 8.03 | 8.43 | 8.20 | 7.45 |
| ILMN_1776577 | DSCC1 | 3.69 | 1.70 | 4.43 | 2.67 | 9.54 | 9.12 | 9.34 | 9.03 | 9.23 |
| ILMN_1754947 | TBC1D1 | 3.70 | 1.06 | 1.83 | 4.87 | 7.11 | 6.87 | 7.04 | 6.99 | 6.79 |
| ILMN_3235964 | HSPA7 | 3.70 | 3.15 | 2.62 | 4.62 | 7.67 | 7.29 | 7.35 | 7.40 | 7.20 |
| ILMN_2073592 | CAND2 | 3.71 | 2.29 | 4.13 | 6.07 | 8.03 | 7.54 | 7.72 | 7.48 | 7.22 |
| ILMN_1786050 | RBBP9 | 3.71 | 0.79 | 3.80 | 3.18 | 8.57 | 8.29 | 8.51 | 8.28 | 8.33 |
| ILMN_2264011 | GRAP | 3.71 | 2.98 | 4.60 | 5.40 | 9.51 | 8.96 | 9.07 | 8.82 | 8.70 |
| ILMN_1752520 | SLFN11 | 3.72 | 1.55 | 2.84 | 4.30 | 9.99 | 9.55 | 9.81 | 9.66 | 9.49 |
| ILMN_1669189 | RASSF5 | 3.73 | 2.67 | 5.04 | 4.31 | 7.94 | 7.44 | 7.58 | 7.27 | 7.37 |
| ILMN_1669015 | XPNPEP1 | 3.73 | 1.14 | 3.80 | 6.08 | 10.87 | 10.52 | 10.77 | 10.52 | 10.30 |
| ILMN_1701869 | FBXO22 | 3.74 | 1.67 | 4.64 | 3.78 | 7.70 | 7.42 | 7.58 | 7.36 | 7.42 |
| ILMN_1811933 | SHMT1 | 3.74 | 3.09 | 5.14 | 2.88 | 8.15 | 7.80 | 7.86 | 7.67 | 7.88 |
| ILMN_2373791 | ENPP2 | 3.74 | 4.72 | 4.17 | 9.57 | 9.40 | 8.70 | 8.52 | 8.62 | 7.60 |
| ILMN_1813704 | KIAA1199 | 3.75 | 3.64 | 5.53 | 4.93 | 7.50 | 7.17 | 7.18 | 7.01 | 7.06 |
| ILMN_1665152 | FCRL2 | 3.75 | 2.41 | 4.06 | 0.22 | 6.98 | 6.70 | 6.80 | 6.67 | 6.96 |
| ILMN_1741976 | SMARCAD1 | 3.76 | 1.36 | 3.80 | 4.92 | 8.89 | 8.58 | 8.78 | 8.58 | 8.49 |
| ILMN_1767337 | SFXN5 | 3.79 | 1.87 | 1.28 | 5.48 | 7.27 | 6.99 | 7.13 | 7.17 | 6.87 |
| ILMN_1718069 | MIS12 | 3.79 | 3.16 | 4.61 | 5.05 | 10.10 | 9.70 | 9.77 | 9.61 | 9.57 |
| ILMN_1742461 | UAP1 | 3.80 | 2.49 | 4.83 | 3.68 | 8.81 | 8.48 | 8.59 | 8.39 | 8.49 |
| ILMN_3200597 | LOC441154 | 3.80 | 1.78 | 2.13 | 5.40 | 10.04 | 9.67 | 9.87 | 9.83 | 9.52 |
| ILMN_1795715 | DPYD | 3.81 | 5.49 | 4.26 | 8.12 | 7.11 | 6.89 | 6.79 | 6.86 | 6.63 |
| ILMN_1697962 | NSMCE1 | 3.82 | 2.47 | 5.37 | 6.48 | 10.33 | 10.07 | 10.16 | 9.96 | 9.88 |
| ILMN_1763129 | DCTPP1 | 3.82 | 2.17 | 3.36 | 3.22 | 11.17 | 10.62 | 10.86 | 10.69 | 10.71 |
| ILMN_2246328 | PTPN22 | 3.82 | 3.74 | 4.75 | -0.95 | 8.43 | 7.96 | 7.97 | 7.84 | 8.54 |
| ILMN_1674551 | SMAD5 | 3.83 | 0.82 | 1.00 | 1.54 | 9.96 | 9.61 | 9.89 | 9.87 | 9.82 |
| ILMN_2059452 | SLC12A2 | 3.83 | 1.12 | 3.64 | 5.20 | 8.81 | 8.47 | 8.71 | 8.48 | 8.34 |
| ILMN_3216979 | LOC646949 | 3.84 | 3.15 | 5.45 | 4.91 | 9.52 | 9.17 | 9.23 | 9.02 | 9.07 |
| ILMN_2102515 | PGAM4 | 3.84 | 1.59 | 2.18 | 2.95 | 8.31 | 7.97 | 8.17 | 8.12 | 8.05 |
| ILMN_2310909 | ATP2A3 | 3.85 | 0.65 | 2.41 | 0.79 | 9.14 | 8.67 | 9.06 | 8.84 | 9.04 |
| ILMN_1687501 | MOXD1 | 3.85 | 2.53 | 4.46 | 8.18 | 8.29 | 7.59 | 7.83 | 7.48 | 6.80 |
| ILMN_1666453 | STK3 | 3.85 | 3.67 | 4.37 | 4.71 | 8.98 | 8.48 | 8.50 | 8.41 | 8.37 |
| ILMN_2145997 | SP4 | 3.86 | 2.01 | 0.57 | 6.74 | 10.11 | 9.77 | 9.93 | 10.06 | 9.51 |
| ILMN_1692698 | VASH2 | 3.86 | 1.73 | 2.49 | 6.71 | 8.62 | 8.15 | 8.41 | 8.32 | 7.80 |
| ILMN_1838313 |  | 3.87 | 2.06 | 1.91 | 1.63 | 8.78 | 8.36 | 8.56 | 8.57 | 8.60 |
| ILMN_1804448 | MSI2 | 3.88 | 3.47 | 3.70 | 4.37 | 9.23 | 8.78 | 8.83 | 8.80 | 8.73 |
| ILMN_2183938 | LEMD3 | 3.88 | 0.49 | 2.73 | -1.41 | 11.44 | 11.04 | 11.39 | 11.16 | 11.58 |
| ILMN_1755364 | RALA | 3.89 | 2.42 | 1.18 | 5.52 | 10.80 | 10.44 | 10.57 | 10.69 | 10.28 |
| ILMN_1727390 | ATPAF2 | 3.89 | 3.89 | 3.93 | 4.17 | 7.25 | 6.97 | 6.97 | 6.97 | 6.95 |
| ILMN_1750044 | ZNHIT3 | 3.89 | 3.23 | 4.71 | 2.88 | 9.06 | 8.51 | 8.60 | 8.39 | 8.65 |
| ILMN_1720438 | LOC653147 | 3.89 | 4.54 | 5.42 | 3.65 | 10.75 | 10.31 | 10.24 | 10.14 | 10.34 |
| ILMN_1781819 | PAPSS1 | 3.90 | 2.71 | 3.27 | 10.80 | 10.70 | 10.23 | 10.38 | 10.31 | 9.40 |
| ILMN_2356031 | RNF121 | 3.91 | 0.83 | 3.24 | 4.53 | 9.07 | 8.75 | 9.00 | 8.81 | 8.70 |
| ILMN_1695370 | LOC645968 | 3.91 | 2.70 | 3.29 | 5.71 | 10.83 | 10.18 | 10.38 | 10.28 | 9.88 |
| ILMN_1772064 | LOC54103 | 3.94 | 2.75 | 3.03 | 6.26 | 7.42 | 7.14 | 7.23 | 7.21 | 6.98 |
| ILMN_2148469 | RASL11B | 3.94 | 1.60 | 2.18 | 2.88 | 7.85 | 7.44 | 7.68 | 7.62 | 7.55 |
| ILMN_3277137 | LOC389404 | 3.94 | 2.43 | 2.45 | 2.83 | 8.98 | 8.50 | 8.68 | 8.68 | 8.63 |
| ILMN_2285817 | FAM89A | 3.94 | 2.15 | 2.53 | 6.34 | 9.14 | 8.62 | 8.86 | 8.81 | 8.31 |
| ILMN_1738523 | MYD88 | 3.94 | 1.57 | 3.30 | 5.51 | 8.57 | 8.24 | 8.44 | 8.29 | 8.11 |
| ILMN_2171289 | SAMSN1 | 3.94 | 4.32 | 4.70 | 8.65 | 7.36 | 7.11 | 7.09 | 7.07 | 6.82 |
| ILMN_3301042 | LQK1 | 3.96 | 4.36 | 4.03 | 4.93 | 8.29 | 7.92 | 7.88 | 7.91 | 7.83 |
| ILMN_1772123 | ACACA | 3.98 | 1.17 | 2.22 | 3.77 | 8.67 | 8.11 | 8.51 | 8.36 | 8.14 |
| ILMN_2051731 | DUSP5P | 3.99 | 0.34 | 2.94 | 3.40 | 7.08 | 6.76 | 7.05 | 6.84 | 6.81 |
| ILMN_1691717 | RHBDF2 | 3.99 | 0.81 | 2.62 | 0.93 | 10.15 | 9.82 | 10.09 | 9.94 | 10.08 |
| ILMN_1701386 | STRADB | 3.99 | 3.10 | 3.61 | 4.67 | 8.84 | 8.52 | 8.59 | 8.55 | 8.47 |
| ILMN_2381753 | G3BP2 | 4.01 | 2.34 | 3.53 | 7.10 | 10.74 | 10.40 | 10.54 | 10.44 | 10.14 |
| ILMN_1758412 | COPS7A | 4.02 | 3.38 | 4.80 | 1.87 | 10.40 | 10.11 | 10.15 | 10.05 | 10.26 |
| ILMN_1748150 | SAMD7 | 4.04 | 3.24 | 3.35 | 4.78 | 7.47 | 7.08 | 7.16 | 7.15 | 7.01 |
| ILMN_1674399 | ZNF143 | 4.04 | 4.36 | 4.95 | 4.23 | 8.74 | 8.31 | 8.28 | 8.21 | 8.29 |
| ILMN_1805449 | TAPBPL | 4.05 | 3.43 | 2.86 | 2.55 | 7.68 | 7.33 | 7.38 | 7.43 | 7.46 |
| ILMN_1656129 | SLC39A10 | 4.06 | 2.62 | 3.54 | 3.86 | 10.07 | 9.20 | 9.51 | 9.31 | 9.25 |
| ILMN_1745497 | C12orf26 | 4.06 | 2.34 | 6.16 | 2.29 | 7.65 | 7.36 | 7.48 | 7.22 | 7.49 |
| ILMN_2395981 | PYHIN1 | 4.08 | 2.52 | 6.46 | 1.25 | 8.59 | 7.97 | 8.20 | 7.60 | 8.40 |
| ILMN_2144088 | FDFT1 | 4.08 | 1.42 | -0.47 | 4.10 | 11.53 | 10.84 | 11.29 | 11.61 | 10.84 |
| ILMN_3219739 | LOC727970 | 4.09 | 3.02 | 3.60 | 7.38 | 9.68 | 9.15 | 9.29 | 9.21 | 8.72 |
| ILMN_1770732 | COPS3 | 4.10 | 3.41 | 5.67 | 3.58 | 10.45 | 10.05 | 10.11 | 9.89 | 10.10 |
| ILMN_3245773 | PION | 4.13 | 3.03 | 3.75 | 7.20 | 7.34 | 7.01 | 7.10 | 7.04 | 6.77 |
| ILMN_1703697 | LANCL1 | 4.13 | 2.62 | 5.14 | 3.31 | 9.85 | 9.29 | 9.50 | 9.16 | 9.40 |
| ILMN_1685781 | C14orf142 | 4.13 | 2.09 | 3.28 | 1.05 | 10.30 | 9.81 | 10.05 | 9.91 | 10.17 |
| ILMN_2355033 | KIAA1147 | 4.13 | 1.14 | 2.15 | 4.85 | 10.03 | 9.51 | 9.89 | 9.76 | 9.42 |
| ILMN_2176768 | SEPHS1 | 4.14 | 2.52 | 2.35 | 7.72 | 10.83 | 10.59 | 10.68 | 10.69 | 10.37 |
| ILMN_1753515 | SRR | 4.14 | 2.69 | 3.39 | 6.19 | 7.19 | 6.94 | 7.03 | 6.98 | 6.81 |
| ILMN_1741005 | RG9MTD2 | 4.15 | 3.02 | 4.50 | 4.11 | 7.96 | 7.57 | 7.68 | 7.54 | 7.57 |
| ILMN_3221865 | RCADH5 | 4.16 | 2.74 | 4.95 | 8.18 | 7.85 | 7.53 | 7.64 | 7.47 | 7.23 |
| ILMN_1654542 | C5orf21 | 4.16 | 1.66 | 3.15 | 4.04 | 8.73 | 8.17 | 8.50 | 8.30 | 8.18 |
| ILMN_2376529 | UMODL1 | 4.16 | 2.73 | 2.96 | 1.29 | 9.92 | 9.27 | 9.49 | 9.46 | 9.72 |
| ILMN_1697820 | HINT2 | 4.17 | 2.70 | 2.80 | 3.74 | 10.22 | 9.79 | 9.94 | 9.93 | 9.83 |
| ILMN_3236825 | RAPGEF5 | 4.18 | 3.64 | 5.16 | 6.68 | 8.25 | 7.47 | 7.57 | 7.29 | 7.01 |
| ILMN_3260345 | AGFG1 | 4.18 | 1.18 | 3.46 | 2.37 | 8.24 | 7.91 | 8.14 | 7.96 | 8.05 |
| ILMN_3281594 | LOC646093 | 4.18 | 1.17 | 2.27 | 1.45 | 7.98 | 7.59 | 7.87 | 7.77 | 7.84 |
| ILMN_1664912 | IL11RA | 4.18 | 2.34 | 3.39 | 2.09 | 7.78 | 7.27 | 7.50 | 7.37 | 7.53 |
| ILMN_1812445 | PRPSAP2 | 4.19 | 2.41 | 3.18 | 3.45 | 8.40 | 8.04 | 8.19 | 8.13 | 8.10 |
| ILMN_1802708 | BTN3A1 | 4.21 | 1.77 | 3.98 | 6.03 | 7.27 | 6.91 | 7.12 | 6.93 | 6.76 |
| ILMN_1754272 | GINS3 | 4.22 | 0.81 | 4.72 | 9.08 | 9.38 | 8.88 | 9.28 | 8.82 | 8.30 |
| ILMN_1728048 | LOC158160 | 4.23 | 2.48 | 2.35 | 6.65 | 8.40 | 7.98 | 8.15 | 8.16 | 7.74 |
| ILMN_1703487 | LMO4 | 4.24 | 2.90 | 3.02 | 4.47 | 10.28 | 9.79 | 9.94 | 9.93 | 9.76 |
| ILMN_1678353 | FARP1 | 4.25 | 2.62 | 2.58 | 3.87 | 7.25 | 6.84 | 7.00 | 7.00 | 6.88 |
| ILMN_1781155 | LYN | 4.26 | 1.53 | 1.29 | 5.97 | 11.30 | 10.89 | 11.15 | 11.17 | 10.73 |
| ILMN_1711810 | PNKD | 4.26 | 4.31 | 4.92 | 1.35 | 8.57 | 8.03 | 8.02 | 7.95 | 8.40 |
| ILMN_1775473 | LOC400652 | 4.26 | 3.24 | 3.36 | 5.16 | 10.49 | 9.92 | 10.05 | 10.04 | 9.80 |
| ILMN_1788416 | FAM108C1 | 4.27 | 1.83 | 4.50 | 7.90 | 9.77 | 9.21 | 9.53 | 9.18 | 8.74 |
| ILMN_1880086 |  | 4.28 | 0.89 | 3.25 | 3.13 | 7.54 | 7.05 | 7.44 | 7.17 | 7.18 |
| ILMN_1692707 | C2orf79 | 4.32 | 3.36 | 4.96 | 7.03 | 9.18 | 8.80 | 8.88 | 8.74 | 8.56 |
| ILMN_1769895 | CCR2 | 4.32 | -0.73 | 0.83 | 2.14 | 9.00 | 8.59 | 9.07 | 8.92 | 8.80 |
| ILMN_1669842 | CHAF1A | 4.33 | -1.28 | 1.14 | 1.09 | 7.96 | 7.66 | 8.05 | 7.88 | 7.89 |
| ILMN_1660871 | NEK6 | 4.33 | 4.56 | 3.35 | 6.86 | 8.69 | 8.25 | 8.22 | 8.35 | 7.99 |
| ILMN_2049184 | DNASE1L3 | 4.34 | 3.38 | 4.95 | 8.39 | 7.79 | 7.30 | 7.41 | 7.23 | 6.83 |
| ILMN_1791329 | FCRL2 | 4.35 | 2.86 | 5.45 | -1.94 | 9.06 | 8.24 | 8.52 | 8.03 | 9.43 |
| ILMN_1756595 | SH3TC1 | 4.36 | 2.29 | 3.69 | 7.12 | 10.40 | 9.45 | 9.90 | 9.60 | 8.85 |
| ILMN_1864166 |  | 4.36 | -1.32 | 0.12 | 3.19 | 8.09 | 7.61 | 8.23 | 8.07 | 7.74 |
| ILMN_1741096 | FDFT1 | 4.38 | 2.45 | 0.06 | 4.80 | 10.50 | 9.77 | 10.09 | 10.49 | 9.70 |
| ILMN_2074258 | BARD1 | 4.41 | 2.60 | 4.20 | 2.78 | 9.78 | 9.29 | 9.49 | 9.32 | 9.47 |
| ILMN_1751062 | SCARA5 | 4.42 | 4.17 | 4.59 | 2.44 | 8.98 | 8.38 | 8.42 | 8.36 | 8.65 |
| ILMN_2348975 | NASP | 4.43 | 0.52 | 3.39 | 3.63 | 8.92 | 8.49 | 8.87 | 8.59 | 8.57 |
| ILMN_1710962 | TMEM97 | 4.43 | 2.19 | 3.52 | 3.69 | 10.59 | 10.07 | 10.33 | 10.17 | 10.15 |
| ILMN_1721081 | SP4 | 4.48 | 1.75 | 0.00 | 5.86 | 9.47 | 9.10 | 9.33 | 9.47 | 8.99 |
| ILMN_1664718 | CYP51A1 | 4.48 | 0.61 | -0.96 | 1.88 | 8.18 | 7.79 | 8.12 | 8.26 | 8.01 |
| ILMN_3276209 | LOC727865 | 4.49 | 2.64 | 2.83 | 4.51 | 12.37 | 11.81 | 12.04 | 12.01 | 11.80 |
| ILMN_1737312 | SLC25A17 | 4.49 | 2.71 | 4.49 | 4.02 | 8.26 | 7.91 | 8.05 | 7.91 | 7.95 |
| ILMN_2398865 | VPS13C | 4.52 | 2.26 | 1.01 | 4.38 | 7.70 | 7.37 | 7.54 | 7.63 | 7.38 |
| ILMN_3251085 | RBBP4 | 4.54 | 0.47 | 1.43 | -0.22 | 9.52 | 9.04 | 9.47 | 9.37 | 9.55 |
| ILMN_2323366 | SDHC | 4.54 | 3.66 | 4.20 | 7.82 | 8.83 | 8.52 | 8.58 | 8.54 | 8.30 |
| ILMN_1653292 | PFKFB4 | 4.54 | 1.88 | 2.43 | 4.60 | 7.80 | 7.28 | 7.58 | 7.52 | 7.27 |
| ILMN_2189027 | LIPG | 4.56 | 2.57 | -0.75 | 3.78 | 7.85 | 6.99 | 7.36 | 7.99 | 7.14 |
| ILMN_1788108 | TXNDC5 | 4.59 | 1.62 | 4.06 | 3.10 | 9.59 | 9.13 | 9.43 | 9.18 | 9.28 |
| ILMN_1677756 | UBE2D2 | 4.61 | 2.14 | 3.69 | 0.00 | 7.63 | 7.26 | 7.46 | 7.34 | 7.63 |
| ILMN_2165867 | DHCR7 | 4.64 | 1.65 | 1.46 | 2.57 | 8.05 | 7.40 | 7.82 | 7.85 | 7.69 |
| ILMN_3244521 | LOC283267 | 4.69 | 1.21 | 2.06 | 4.53 | 8.36 | 7.97 | 8.26 | 8.19 | 7.98 |
| ILMN_1700202 | TMEM135 | 4.70 | 2.03 | 4.06 | 6.13 | 8.99 | 8.63 | 8.83 | 8.68 | 8.52 |
| ILMN_3300051 | LOC731542 | 4.71 | 2.09 | 3.19 | 4.50 | 8.25 | 7.80 | 8.05 | 7.95 | 7.82 |
| ILMN_1716895 | RPA3 | 4.73 | 2.66 | 2.83 | 4.12 | 11.68 | 11.21 | 11.42 | 11.40 | 11.27 |
| ILMN_2100689 | MAP2K4 | 4.73 | 2.59 | 5.87 | 4.69 | 7.96 | 7.60 | 7.77 | 7.52 | 7.61 |
| ILMN_1669447 | PYHIN1 | 4.76 | 2.19 | 6.62 | 2.68 | 9.24 | 8.39 | 8.85 | 8.06 | 8.76 |
| ILMN_1660793 | PAQR4 | 4.80 | 0.49 | 3.12 | 4.44 | 9.86 | 9.23 | 9.80 | 9.45 | 9.28 |
| ILMN_2217935 | RFC1 | 4.84 | 1.50 | 4.43 | 5.15 | 9.66 | 9.18 | 9.51 | 9.22 | 9.15 |
| ILMN_1651346 | TICAM2 | 4.86 | 3.24 | 3.37 | 4.81 | 7.90 | 7.52 | 7.65 | 7.64 | 7.52 |
| ILMN_1730572 | HNRPDL | 4.89 | 0.37 | 3.87 | 4.51 | 9.74 | 9.26 | 9.71 | 9.36 | 9.30 |
| ILMN_1788053 | SLC25A12 | 4.89 | 3.73 | 4.20 | 3.57 | 8.53 | 8.01 | 8.14 | 8.09 | 8.15 |
| ILMN_2211546 | HAO1 | 4.91 | 5.26 | 5.70 | 8.44 | 7.43 | 7.06 | 7.03 | 7.00 | 6.79 |
| ILMN_1664682 | DNA2 | 4.94 | -0.17 | 1.63 | 3.28 | 8.71 | 8.12 | 8.73 | 8.51 | 8.32 |
| ILMN_1672662 | SLC20A1 | 4.96 | 0.97 | 4.57 | 0.50 | 10.45 | 10.02 | 10.37 | 10.06 | 10.41 |
| ILMN_2385278 | WDR21A | 4.96 | 0.42 | 2.92 | 1.36 | 8.35 | 8.03 | 8.32 | 8.16 | 8.26 |
| ILMN_1753010 | PET112L | 4.99 | 4.65 | 6.41 | 12.82 | 9.26 | 8.87 | 8.89 | 8.75 | 8.24 |
| ILMN_2043728 | ZNF341 | 4.99 | 3.27 | 5.25 | 3.01 | 7.64 | 7.18 | 7.34 | 7.16 | 7.36 |
| ILMN_1771800 | PRKCA | 5.00 | 4.38 | 5.36 | 3.05 | 7.84 | 7.46 | 7.51 | 7.43 | 7.61 |
| ILMN_1666902 | GPR114 | 5.02 | 3.31 | 2.00 | 6.44 | 8.99 | 8.29 | 8.53 | 8.71 | 8.10 |
| ILMN_1715024 | LSS | 5.06 | 1.50 | 2.41 | 2.01 | 8.77 | 8.14 | 8.58 | 8.47 | 8.52 |
| ILMN_1699022 | ENDOD1 | 5.09 | 1.21 | 4.18 | 3.71 | 8.34 | 7.84 | 8.22 | 7.93 | 7.97 |
| ILMN_1790534 | MAP2K3 | 5.14 | 2.57 | 3.37 | 5.19 | 9.15 | 8.51 | 8.83 | 8.73 | 8.50 |
| ILMN_3264466 | FAM54B | 5.23 | 2.94 | 4.64 | 3.52 | 7.77 | 7.48 | 7.61 | 7.51 | 7.57 |
| ILMN_3299356 | LOC729004 | 5.25 | 2.95 | 2.08 | 2.55 | 8.08 | 7.60 | 7.81 | 7.89 | 7.84 |
| ILMN_1783156 | LOC650832 | 5.27 | 2.02 | 3.02 | 6.08 | 8.77 | 8.25 | 8.57 | 8.47 | 8.17 |
| ILMN_1704554 | LOC648470 | 5.28 | 5.35 | 6.91 | 6.97 | 7.42 | 6.91 | 6.90 | 6.75 | 6.74 |
| ILMN_1753353 | SLBP | 5.32 | 1.86 | 4.16 | 3.43 | 9.48 | 8.75 | 9.22 | 8.91 | 9.01 |
| ILMN_1821176 |  | 5.34 | 1.94 | 1.84 | 5.30 | 8.95 | 8.45 | 8.77 | 8.78 | 8.45 |
| ILMN_1727495 | L3MBTL3 | 5.35 | 2.25 | 4.50 | 5.27 | 7.55 | 7.07 | 7.35 | 7.14 | 7.07 |
| ILMN_3245578 | LOC729510 | 5.35 | 3.93 | 6.81 | 7.13 | 8.42 | 7.98 | 8.09 | 7.86 | 7.83 |
| ILMN_2413898 | MCM10 | 5.35 | -0.57 | 2.95 | 5.45 | 10.24 | 9.68 | 10.30 | 9.93 | 9.67 |
| ILMN_1774761 | CCR2 | 5.39 | 0.51 | 1.83 | 1.86 | 9.13 | 8.60 | 9.08 | 8.95 | 8.94 |
| ILMN_1737635 | RAD1 | 5.48 | 2.23 | 3.69 | 4.08 | 8.15 | 7.77 | 8.00 | 7.90 | 7.87 |
| ILMN_1749502 | ZNF215 | 5.50 | 4.18 | 6.76 | 7.69 | 7.48 | 6.91 | 7.04 | 6.78 | 6.68 |
| ILMN_1657395 | HMGCR | 5.57 | 4.23 | 2.51 | 4.81 | 9.69 | 9.11 | 9.25 | 9.43 | 9.19 |
| ILMN_2326273 | CHI3L2 | 5.57 | 1.97 | 1.49 | 2.19 | 7.55 | 7.05 | 7.38 | 7.42 | 7.36 |
| ILMN_3201937 | LOC645381 | 5.58 | 2.24 | 2.00 | 3.92 | 8.54 | 8.00 | 8.32 | 8.34 | 8.16 |
| ILMN_1686319 | USP37 | 5.60 | 2.07 | 5.58 | 3.24 | 8.80 | 8.08 | 8.53 | 8.08 | 8.38 |
| ILMN_1756417 | ANKRD37 | 5.61 | 3.94 | 4.10 | 9.36 | 8.31 | 7.61 | 7.82 | 7.80 | 7.15 |
| ILMN_3235185 | SNRNP200 | 5.63 | 1.77 | 3.80 | 6.13 | 8.25 | 7.65 | 8.06 | 7.84 | 7.59 |
| ILMN_1738554 | LOC647346 | 5.66 | 4.13 | 6.25 | 9.82 | 8.87 | 8.39 | 8.52 | 8.34 | 8.04 |
| ILMN_3235567 | LOC150568 | 5.72 | 3.83 | 5.59 | 7.31 | 7.86 | 7.27 | 7.46 | 7.28 | 7.11 |
| ILMN_1720889 | SC4MOL | 5.74 | 2.10 | 0.45 | 5.49 | 9.37 | 8.48 | 9.04 | 9.30 | 8.52 |
| ILMN_2055700 | SLBP | 5.77 | 2.25 | 4.03 | 6.01 | 10.70 | 9.98 | 10.42 | 10.20 | 9.95 |
| ILMN_1808757 | RPL37A | 5.89 | 2.52 | 6.82 | 6.03 | 8.15 | 7.34 | 7.80 | 7.21 | 7.32 |
| ILMN_1811148 | BMP2K | 5.99 | 3.95 | 5.17 | 12.32 | 8.68 | 8.10 | 8.30 | 8.18 | 7.48 |
| ILMN_1815626 | DHCR7 | 6.18 | 2.86 | 2.13 | 3.26 | 8.06 | 7.39 | 7.75 | 7.83 | 7.71 |
| ILMN_1727098 | PPP1R16B | 6.23 | 1.29 | 1.86 | 1.62 | 10.39 | 9.87 | 10.28 | 10.23 | 10.25 |
| ILMN_2205032 | MAGEE1 | 6.33 | 3.01 | 5.10 | 7.86 | 7.84 | 7.24 | 7.56 | 7.36 | 7.10 |
| ILMN_2053415 | LDLR | 6.52 | 2.18 | -0.78 | 1.67 | 8.81 | 7.47 | 8.36 | 8.97 | 8.47 |
| ILMN_1683250 | LOC440731 | 6.61 | 5.90 | 6.93 | 5.47 | 8.29 | 7.32 | 7.43 | 7.28 | 7.49 |
| ILMN_1779711 | DTL | 6.82 | -0.11 | 2.22 | 3.00 | 9.41 | 8.80 | 9.42 | 9.21 | 9.14 |
| ILMN_1797728 | HMGCS1 | 7.03 | 3.39 | 2.05 | 5.30 | 10.13 | 8.80 | 9.49 | 9.74 | 9.13 |
| ILMN_1790891 | CKAP4 | 7.03 | 1.90 | 5.22 | -0.68 | 9.51 | 9.03 | 9.38 | 9.16 | 9.56 |
| ILMN_1793474 | INSIG1 | 7.63 | 3.48 | 3.00 | 4.92 | 10.13 | 8.97 | 9.60 | 9.68 | 9.38 |
| ILMN_1707975 | SERPIND1 | 7.85 | 2.98 | 4.33 | 9.16 | 7.36 | 6.78 | 7.14 | 7.04 | 6.68 |
| ILMN_2041293 | SQLE | 8.91 | 5.13 | 2.37 | 4.71 | 10.72 | 9.79 | 10.19 | 10.47 | 10.23 |
| ILMN_3236428 | TMEM170B | 9.72 | 1.71 | 3.59 | 8.77 | 8.58 | 7.76 | 8.43 | 8.28 | 7.84 |
