# Supplementary material for: Combined inhibition of Chk1 and Wee1 as a new therapeutic strategy for mantle cell lymphoma
Source: Oncotarget. 2014 Oct 25;6(5):3394–408. doi: 10.18632/oncotarget.2583 (PMC4413661; doi:10.18632/oncotarget.2583)
Supplement: Supplementary file 3 [file oncotarget-06-3394-s003.doc]

| **Comparison** | **NAME** | **SIZE** | **ES** | **NES** | **NOM p-val** | **FDR q-val** | **Genes in the Enrichment Core** |
| --- | --- | --- | --- | --- | --- | --- | --- |
| up in combo1 vs ctr | NAKAYAMA_SOFT_TISSUE_TUMORS_PCA2_UP | 63 | -0.825 | -3.207 | 0.000 | 0.000 | PBK, MLLT11, NUSAP1, GTSE1, BIRC5, SCG5, CDKN3, NEK2, HMMR, CKS2, KIF11, CCNB1, CCNB2, MKI67, CENPE, TOP2A, CCNA2, DLGAP5, GPSM2, SLC7A5, CENPF, TPX2, ESPL1, CEP55, BUB1, CENPA, CDC20, HJURP, KIF2C, CDCA8, KIF20A |
| up in combo1 vs ctr | SOTIRIOU_BREAST_CANCER_GRADE_1_VS_3_UP | 139 | -0.710 | -3.138 | 0.000 | 0.000 | OIP5, TIMM10, MARS, SPAG5, KIF4A, KPNA2, NCAPG, FOXM1, NUSAP1, HN1, GTSE1, BIRC5, CDKN3, NEK2, HMMR, CKS2, PTTG1, ECT2, KIF11, PTTG3P, NDC80, CCNB1, TROAP, CCNB2, FAM64A, MKI67, CENPE, TOP2A, CCNA2, AURKB, CDCA3, DLGAP5, TTK, PLK1, STIL, SLC7A5, CENPF, TPX2, TACC3, ESPL1, RACGAP1, PRC1, CEP55, BUB1, BUB1B, KIF14, KIFC1, CENPA, CDC20, AURKA, HJURP, KIF2C, CDCA8, KIF20A |
| up in combo1 vs ctr | AMUNDSON_GAMMA_RADIATION_RESPONSE | 39 | -0.864 | -2.991 | 0.000 | 0.000 | CDC25C, KIF23, NEK2, CKS2, PTTG1, CCNB1, H2AFX, CCNB2, CENPE, WEE1, CCNA2, AURKB, TTK, PLK1, CENPF, TACC3, ESPL1, PRC1, BUB1, BUB1B, CCNF, KIFC1, CENPA, AURKA, KIF2C |
| up in combo1 vs ctr | WHITFIELD_CELL_CYCLE_G2_M | 182 | -0.649 | -2.991 | 0.000 | 0.000 | CTNND1, THRAP3, TLE3, CCDC88A, ATF7IP, TGIF1, ZC3HC1, HPS4, CADM1, CNTROB, KIF20B, GOT1, CDKN2D, CCDC99, DEPDC1B, NUP98, GRK6, DNAJA1, HP1BP3, ZFX, ZMYM1, CKAP5, CKS1B, SGOL2, LMNA, SETD8, ASXL1, PRR11, HMG20B, ODF2, NCOA5, RCBTB2, KLF9, SPAG5, PBK, MDC1, ARL6IP1, PRPSAP1, KIF5B, NCAPD2, FOXM1, TNFAIP8L1, NUSAP1, HN1, GTSE1, BIRC5, NEK2, NDE1, HMMR, CKS2, YWHAH, ECT2, ARHGAP19, CCNB2, FAM64A, MKI67, CTCF, CENPE, CCNA2, TMEM138, CDCA3, DLGAP5, TTK, PLK1, CENPF, TPX2, TACC3, AKIRIN2, ANLN, CEP55, CKAP2, BUB1, RANGAP1, KIF14, CENPA, CDC20, AURKA, KIF2C |
| up in combo1 vs ctr | WHITFIELD_CELL_CYCLE_G2 | 152 | -0.663 | -2.948 | 0.000 | 0.000 | KIF20B, TNPO2, GABPB1, CEP350, EBI3, DCAF7, CHEK2, HP1BP3, IFNAR1, TRIM59, KBTBD2, HMGB2, CDKN1B, NMB, WDR62, KIAA1524, ASXL1, MEPCE, NCOA5, CDC25C, KIF23, KATNA1, KCTD9, KIF22, BRD8, KPNA2, KIF5B, NUSAP1, HN1, FAM72B, ATL2, CENPL, KIF11, ARHGAP19, KLF6, NDC80, H2AFX, LIX1L, PIF1, TOP2A, CCNA2, AURKB, CDCA3, CDR2, ARHGAP11B, STIL, ESPL1, FAM83D, ANLN, FAM110A, CKAP2, RANGAP1, CCNF, KIFC1, CDCA2, CKAP2L, HJURP, CDCA8, PSRC1 |
| up in combo1 vs ctr | ROSTY_CERVICAL_CANCER_PROLIFERATION_CLUSTER | 130 | -0.664 | -2.923 | 0.000 | 0.000 |  |
| up in combo1 vs ctr | REICHERT_MITOSIS_LIN9_TARGETS | 24 | -0.908 | -2.873 | 0.000 | 0.000 |  |
| up in combo1 vs ctr | ODONNELL_TFRC_TARGETS_DN | 102 | -0.677 | -2.864 | 0.000 | 0.000 |  |
| up in combo1 vs ctr | HU_GENOTOXIC_DAMAGE_4HR | 33 | -0.825 | -2.854 | 0.000 | 0.000 |  |
| up in combo1 vs ctr | ZHOU_CELL_CYCLE_GENES_IN_IR_RESPONSE_24HR | 109 | -0.668 | -2.843 | 0.000 | 0.000 |  |
| up in combo1 vs ctr | FARMER_BREAST_CANCER_CLUSTER_2 | 32 | -0.850 | -2.834 | 0.000 | 0.000 |  |
| up in combo1 vs ctr | CHANG_CYCLING_GENES | 131 | -0.644 | -2.832 | 0.000 | 0.000 |  |
| up in combo1 vs ctr | TANG_SENESCENCE_TP53_TARGETS_DN | 48 | -0.759 | -2.810 | 0.000 | 0.000 |  |
| up in combo1 vs ctr | CROONQUIST_IL6_DEPRIVATION_DN | 92 | -0.662 | -2.793 | 0.000 | 0.000 |  |
| up in combo1 vs ctr | SHEPARD_BMYB_TARGETS | 51 | -0.744 | -2.792 | 0.000 | 0.000 |  |
| up in ctr vs combo1 | HORTON_SREBF_TARGETS | 22 | 0.871 | 2.531 | 0.000 | 0.000 | SQLE, INSIG1, HMGCS1, LDLR, DHCR7, LSS, CYP51A1, FDFT1, ACACA, ELOVL6, ALDOC, FDPS, IDI1, RDH11, SC5DL |
| up in ctr vs combo1 | SCHMIDT_POR_TARGETS_IN_LIMB_BUD_UP | 22 | 0.860 | 2.525 | 0.000 | 0.000 | SQLE, INSIG1, HMGCS1, LDLR, DHCR7, LSS, CYP51A1, FDFT1, ELOVL6, FDPS, ACAT2, IDI1, MVK, SC5DL, NSDHL |
| up in ctr vs combo1 | ZHAN_MULTIPLE_MYELOMA_CD1_VS_CD2_DN | 45 | 0.675 | 2.293 | 0.000 | 0.000 | ZNF215, PRKCA, UBE2D2, NEK6, RAPGEF5, SLFN11, TBC1D1, PRKCB, CD1D, BCL11A, RYK, OSBPL10, RNGTT, SC5DL, PNOC, KLHL14, RASSF6, CD27, PIK3AP1, GPR160, SPINT2, TNS3 |
| up in ctr vs combo1 | WHITFIELD_CELL_CYCLE_G1_S | 110 | 0.544 | 2.180 | 0.000 | 0.000 | DTL, SLBP, NASP, BARD1, CHAF1A, GINS3, KIAA1147, DSCC1, UNG, CHAF1B, CREBZF, FAM111B, CCNE1, MCM6, HSF2, ABCA7, RAB23, PLCXD1, PCNA, CCNE2, ZMYND19, HELLS, MCM2, UBR7, MED31, RNPC3, MRI1, SKP2, E2F2, AP4B1, NUP43, APEX2, DHFRL1, NPAT, GINS2, CDC25A, DONSON, E2F1, RECQL4, SPIN3, TIPIN, PDXP, ATAD2, PASK, CASP2, INSR, MBOAT1, TTC14 |
| up in ctr vs combo1 | BREDEMEYER_RAG_SIGNALING_NOT_VIA_ATM_UP | 37 | 0.595 | 1.973 | 0.000 | 0.015 | SLC20A1, NOTCH2, ELOVL6, GH1, KLHL7, SGCB, PAIP1, ISOC1, CDC23, RCAN1, PHF17, KLHL14, ACPP, PIK3AP1, GAK, SMG1, PJA1, CCDC25, MAF |
| up in ctr vs combo1 | WANG_RESPONSE_TO_FORSKOLIN_UP | 17 | 0.709 | 1.935 | 0.000 | 0.024 |  |
| up in ctr vs combo1 | LEE_LIVER_CANCER_MYC_TGFA_DN | 34 | 0.597 | 1.933 | 0.000 | 0.022 |  |
| up in ctr vs combo1 | GUO_TARGETS_OF_IRS1_AND_IRS2 | 67 | 0.526 | 1.925 | 0.002 | 0.022 |  |
| up in ctr vs combo1 | BURTON_ADIPOGENESIS_10 | 23 | 0.647 | 1.919 | 0.000 | 0.021 |  |
| up in ctr vs combo1 | STEIN_ESRRA_TARGETS_RESPONSIVE_TO_ESTROGEN_DN | 32 | 0.604 | 1.912 | 0.002 | 0.021 |  |
| up in ctr vs combo1 | AIYAR_COBRA1_TARGETS_DN | 22 | 0.662 | 1.911 | 0.000 | 0.020 |  |
| up in ctr vs combo1 | LE_EGR2_TARGETS_DN | 70 | 0.514 | 1.907 | 0.000 | 0.018 |  |
| up in ctr vs combo1 | PLASARI_TGFB1_SIGNALING_VIA_NFIC_1HR_UP | 22 | 0.640 | 1.873 | 0.000 | 0.029 |  |
| up in ctr vs combo1 | GUILLAUMOND_KLF10_TARGETS_UP | 34 | 0.576 | 1.835 | 0.000 | 0.046 |  |
| up in ctr vs combo1 | SLEBOS_HEAD_AND_NECK_CANCER_WITH_HPV_UP | 60 | 0.506 | 1.820 | 0.002 | 0.052 |  |
| up in MK1775 vs ctr | NAKAYAMA_SOFT_TISSUE_TUMORS_PCA2_UP | 63 | -0.735 | -3.019 | 0.000 | 0.000 | CENPF, CENPA, KIF20A, CDC20, KIF2C, SLC7A5 |
| up in MK1775 vs ctr | AMUNDSON_GAMMA_RADIATION_RESPONSE | 39 | -0.770 | -2.924 | 0.000 | 0.000 | AIMP2, LBR, CCNB1, CKS2, CCNA2, WEE1, NEK2, TTK, CCNB2, CENPE, H2AFX, AURKA, PRC1, MSX1, PLK1, ESPL1, CCNF, BUB1, CENPF, CENPA, TACC3, BUB1B, KIFC1, KIF2C |
| up in MK1775 vs ctr | SOTIRIOU_BREAST_CANCER_GRADE_1_VS_3_UP | 139 | -0.602 | -2.883 | 0.000 | 0.000 | CENPI, NCAPG, ASPM, TUBA1B, FOXM1, CCNB1, OIP5, CDKN3, CKS2, KIF11, STIL, EZH2, BIRC5, NUTF2, TOP2A, CCNA2, NUSAP1, QPRT, NDC80, HMGB3, ECT2, NEK2, HN1, TROAP, TTK, CCNB2, CENPE, MKI67, DLGAP5, MARS, GTSE1, AURKA, CDCA3, KIF4A, PRC1, RACGAP1, PLK1, ESPL1, CEP55, FAM64A, BUB1, TPX2, CDCA8, CENPF, CENPA, TACC3, KIF20A, KIF14, BUB1B, KIFC1, CDC20, KIF2C, SLC7A5 |
| up in MK1775 vs ctr | WHITFIELD_CELL_CYCLE_G2_M | 182 | -0.567 | -2.845 | 0.000 | 0.000 | NCAPD2, CDKN2D, SPTBN1, FOXM1, DR1, CCDC88A, CKS2, NDE1, RNF126, SRF, PRR11, BIRC5, PRPSAP1, KLF9, MDC1, ARL6IP1, CCNA2, NUSAP1, RCBTB2, HMGB3, ECT2, NEK2, HN1, GRK6, DZIP3, TTK, SETD8, NCOA5, CCNB2, CENPE, CTCF, CKAP2, MKI67, DLGAP5, AKIRIN2, TNFAIP8L1, LMNA, GTSE1, AURKA, CDCA3, HMG20B, PLK1, YWHAH, CEP55, FAM64A, BUB1, TPX2, CENPF, ANLN, CENPA, TACC3, KIF14, ARHGAP19, RANGAP1, CDC20, KIF2C |
| up in MK1775 vs ctr | REICHERT_MITOSIS_LIN9_TARGETS | 24 | -0.834 | -2.785 | 0.000 | 0.000 | ASPM, NCAPD2, CCNB1, TOP2A, NUSAP1, CDCA2, CENPE, MKI67, AURKA, PLK1, CCNF, CEP55, CENPF, CENPA, KIF20A, KIF2C |
| up in MK1775 vs ctr | TANG_SENESCENCE_TP53_TARGETS_DN | 48 | -0.687 | -2.673 | 0.000 | 0.000 |  |
| up in MK1775 vs ctr | ROSTY_CERVICAL_CANCER_PROLIFERATION_CLUSTER | 130 | -0.560 | -2.666 | 0.000 | 0.000 |  |
| up in MK1775 vs ctr | ODONNELL_TFRC_TARGETS_DN | 102 | -0.571 | -2.663 | 0.000 | 0.000 |  |
| up in MK1775 vs ctr | KRIGE_AMINO_ACID_DEPRIVATION | 25 | -0.796 | -2.661 | 0.000 | 0.000 |  |
| up in MK1775 vs ctr | WHITEFORD_PEDIATRIC_CANCER_MARKERS | 104 | -0.571 | -2.650 | 0.000 | 0.000 |  |
| up in MK1775 vs ctr | BURTON_ADIPOGENESIS_PEAK_AT_24HR | 33 | -0.718 | -2.629 | 0.000 | 0.000 |  |
| up in MK1775 vs ctr | FARMER_BREAST_CANCER_CLUSTER_2 | 32 | -0.721 | -2.618 | 0.000 | 0.000 |  |
| up in MK1775 vs ctr | GREENBAUM_E2A_TARGETS_UP | 29 | -0.740 | -2.580 | 0.000 | 0.000 |  |
| up in MK1775 vs ctr | HU_GENOTOXIC_DAMAGE_4HR | 33 | -0.706 | -2.578 | 0.000 | 0.000 |  |
| up in MK1775 vs ctr | LY_AGING_MIDDLE_DN | 15 | -0.878 | -2.556 | 0.000 | 0.000 |  |
| up in ctr vs MK1775 | GHANDHI_DIRECT_IRRADIATION_UP | 56 | 0.569 | 2.034 | 0.000 | 0.038 | MT1E, MT2A, MT1G, MT1H, MT1A, RRM2B, BTG3, MMP1, MT1X, CYP26B1, NFKBIZ, BIRC3, RELB, ASCC3, EYA3, KYNU, ZC3H12C |
| up in ctr vs MK1775 | CHICAS_RB1_TARGETS_LOW_SERUM | 64 | 0.556 | 1.997 | 0.000 | 0.031 | KIAA0101, MT1E, MT2A, MT1H, RPL36A, RIF1, MT1X, HELLS, TYMS, ZNF827, TCEB2, MT1F, MTHFD2L, RPA3, MCM6, ATOX1, ATP5L, DTL, CDT1, CCNE2, SNRPE, RNF145, CDCA7 |
| up in ctr vs MK1775 | GARY_CD5_TARGETS_DN | 392 | 0.447 | 1.997 | 0.000 | 0.021 | SP140, KIAA1826, BMP2K, LTB, RG9MTD2, RPP40, APTX, AZIN1, BZW1, LCMT2, TFRC, GLMN, CASP1, MT1X, PGM2, SH3TC1, GEMIN6, AIM2, ETS1, COMMD5, DNAJC10, NASP, SLC25A19, DCTPP1, UTP15, WDR36, SLC30A5, KRT10, MRPL30, PAPOLA, GRSF1, CRYZ, GTPBP4, POLA2, TANK, FCGR2B, ZNF207, MAGOHB, TFAM, UTP3, PHAX, FEN1, PSMF1, SLC44A1, IPO11, RFC2, DNAJC9, CSTF2T, PFDN1, PNO1, YRDC, POLR3G, ETF1, HSPA4, OTUD6B, BAG2, PPIG, RRP15, BTN3A3, EIF4E2, GFM1, SLC11A2, MRPS23, MANEA, RARS, TIPIN, ASCC3, MRPL35, GEMIN4, TCEB3, TMEM33, HLA-DPA1, POLE2, BAG5, SEPHS1, ATP6V1C1, WDR3, RBM22, SAMD9, TLR10, FBXO28, DTL, INTS2, PPWD1, RFC3, BCL11A, ASNSD1, EIF1AX, CCNE2, UFD1L, AFF3, TDG, EMG1, USP16, CCT6A, BCCIP, KMO, STAG1, POLR1C, HSPA14, NAV2, PIGW, PSMC5, SERTAD2, ADI1, NOL11, ZNF407, IKBIP, METTL13, TUBA4A, ANP32A, PSMA7, UBE2F, TXNDC9, TNPO1, PLAA, FASTKD2, WWP1, DNAJA1, CREM, FUBP1, UGDH, STX7, NUP160, ATP2B1, GPR183 |
| up in ctr vs MK1775 | MILI_PSEUDOPODIA_HAPTOTAXIS_UP | 422 | 0.431 | 1.941 | 0.000 | 0.036 | KIAA1826, ZMAT2, UBLCP1, CHMP2B, BZW1, RAB8B, AGGF1, TTC35, CCT4, NUDT2, UBE2D2, EFR3A, PDK1, ANAPC4, NUP54, TTC1, AGFG1, MBIP, CYFIP1, IFT74, SLC25A46, CCNG1, CRYZL1, VPS41, MRRF, OXR1, ZNF131, GTPBP4, HIBCH, MTHFD2L, TANK, TIMM8A, RABEP1, TOPORS, TRMT11, PSMC3IP, CETN3, MFF, CISD2, PAIP1, NUFIP2, PNO1, KCTD10, WDR75, UBR3, DCAF6, SNX5, NDUFS4, EXOSC9, COPS2, SF3B1, MLF1IP, RPS20, CENPQ, CSNK1G3, UBE2D3, SAMD9L, UGP2, AASDHPPT, TTC33, ATE1, ZNF277, TROVE2, DMXL1, USP46, SSB, YTHDF2, MAPK8, PPWD1, ASNSD1, DYNC1LI2, EIF1AX, CCNE2, ABI2, PPP1R7, HMGCS1, PTPN2, MRPL13, USP16, IFIT2, STAG1, MYO5A, HSPA9, PAPD4, PJA2, RPAP2, NR3C1, PDSS1, BTBD1, CUL3, MRPL47, MAK16, NUDCD2, CENPK, TIGD2, TXNDC9, KIAA1143, ZRANB2, RPL17, ARPP19, PRPF40A, HDGFRP3, MYCBP, SKP1, RPF2, CHPT1, TMEM167A, GPATCH4, MTAP, RBM25, NAP1L1, YES1, ANKRD13C, MRPS14, RASA1, VPS4B, UBE2V2, ERGIC2, OGFRL1, NAMPT, TOMM70A, RBM34, CCNH, PDCD10, OPA1, TBC1D15, FNBP1L, KIF2A, SNHG6, FAM179B, PSMD6, FAM133B, RWDD1, MATR3, XPO1, GTF3C6, PTAR1, SUMO1, MRPS18C, DEK, NAE1, FKBP3, ZNRF2, RALA, ARL5A, PIP4K2A, COMMD8, CAB39L, GMPS, METAP2, SCRN3, THUMPD3, ATP6V1D |
| up in ctr vs MK1775 | KIM_TIAL1_TARGETS | 30 | 0.602 | 1.914 | 0.002 | 0.044 | NSMCE1, MT1H, SMARCE1, DPYD, MT1X, ELOVL6, COX10 |
| up in ctr vs MK1775 | WONG_MITOCHONDRIA_GENE_MODULE | 195 | 0.456 | 1.909 | 0.000 | 0.040 |  |
| up in ctr vs MK1775 | NIKOLSKY_BREAST_CANCER_8Q23_Q24_AMPLICON | 95 | 0.485 | 1.889 | 0.000 | 0.048 |  |
| up in ctr vs MK1775 | RICKMAN_METASTASIS_UP | 256 | 0.429 | 1.887 | 0.000 | 0.043 |  |
| up in ctr vs MK1775 | LASTOWSKA_NEUROBLASTOMA_COPY_NUMBER_UP | 143 | 0.458 | 1.874 | 0.000 | 0.048 |  |
| up in ctr vs MK1775 | MOOTHA_HUMAN_MITODB_6_2002 | 371 | 0.413 | 1.872 | 0.000 | 0.044 |  |
| up in ctr vs MK1775 | SLEBOS_HEAD_AND_NECK_CANCER_WITH_HPV_UP | 60 | 0.510 | 1.853 | 0.001 | 0.054 |  |
| up in ctr vs MK1775 | HEIDENBLAD_AMPLICON_12P11_12_UP | 24 | 0.629 | 1.852 | 0.000 | 0.050 |  |
| up in ctr vs MK1775 | KIM_MYC_AMPLIFICATION_TARGETS_UP | 150 | 0.445 | 1.851 | 0.000 | 0.047 |  |
| up in ctr vs MK1775 | MOOTHA_MITOCHONDRIA | 380 | 0.411 | 1.845 | 0.000 | 0.046 |  |
| up in ctr vs MK1775 | GUILLAUMOND_KLF10_TARGETS_UP | 34 | 0.575 | 1.830 | 0.001 | 0.052 |  |
| up in PF4777369 vs ctr | KRIGE_AMINO_ACID_DEPRIVATION | 25 | -0.662 | -2.359 | 0.000 | 0.001 | WARS, PSAT1, SESN2, IL8, CHAC1, CARS, ATF3, CEBPB, ATF5, MARS, TRIB3 |
| up in PF4777369 vs ctr | GINESTIER_BREAST_CANCER_ZNF217_AMPLIFIED_DN | 261 | -0.408 | -2.257 | 0.000 | 0.005 | ANKRD13D, APBA3, NSUN4, PTBP1, NEU4, LMNA, PPP1R12C, ANKRD54, EIF3B, TTC17, ZC3H7B, SETD5, GPC2, U2AF2, MESP1, TARDBP, TEAD3, ARHGAP30, MOGS, RANGAP1, CAMK2B, ZNF473, ABCC10, SEC31B, TCF3, CSNK1G2, SOLH, MDN1, TNPO3, KIAA0907, SEMA6B, LENG8, ARMC6, PQBP1, EHMT2, RBM14, QTRT1, PPARD, MYO9B, ZNF524, GIGYF1, GRAMD4, SLC4A11, TRMU, LTB4R, SNRNP70, CTNND1, ARPC5L, RBM15B, DAGLB, MBD3, KIAA0415, SETDB1, ZMYND19, SUV39H1, SLC12A9, CNOT3, TSPAN14, DOM3Z, HCFC1, SMG5, EPN1, TNPO2, UBAP2L, PBX2, FNBP4, ACIN1, TMCC1, INTS1, FURIN, CSNK1E, REXO1, ATF5, RBM10, MAPK8IP3, TRABD, CIC, TBC1D22B, MAP2K2, GTF2H4, PHF21A, PUS1, ZNF444, SRL, DENND4B, PPM1G, ZNF358 |
| up in PF4777369 vs ctr | PENG_GLUCOSE_DEPRIVATION_UP | 37 | -0.577 | -2.223 | 0.000 | 0.007 | AGFG2, CORO1A, CLCN6, PCK2, GM2A, CTTN, UCKL1, ASNS, TCF20, TLX2, MFNG, WARS, PYCR1, POU2F2, MYB, CARS, CTSD, CEBPB, ACD, ATF5, ADA, AARS, SBF1 |
| up in PF4777369 vs ctr | ZHAN_MULTIPLE_MYELOMA_CD1_VS_CD2_UP | 39 | -0.543 | -2.111 | 0.000 | 0.021 | TBC1D16, GALNT3, ASNS, MAP1B, RAB33A, KCNK12, CTAGE5, WARS, PHGDH, SESN2, CEBPG, KIF21B, ITPRIPL2, CEBPB, ATF5, GPT2, INHBE, TM6SF1, SLC7A5, TRIB3 |
| up in PF4777369 vs ctr | QI_HYPOXIA_TARGETS_OF_HIF1A_AND_FOXA2 | 27 | -0.581 | -2.072 | 0.000 | 0.028 | SRM, JUND, MED15, CTNND1, BCL2L1, CNOT3, TRPC4AP, AES, PPM1G |
| up in PF4777369 vs ctr | NIKOLSKY_BREAST_CANCER_16P13_AMPLICON | 68 | -0.458 | -2.067 | 0.000 | 0.024 |  |
| up in PF4777369 vs ctr | SHEDDEN_LUNG_CANCER_GOOD_SURVIVAL_A5 | 52 | -0.480 | -2.002 | 0.000 | 0.044 |  |
| up in PF4777369 vs ctr | CHARAFE_BREAST_CANCER_LUMINAL_VS_BASAL_UP | 246 | -0.367 | -1.994 | 0.000 | 0.040 |  |
| up in PF4777369 vs ctr | WELCSH_BRCA1_TARGETS_DN | 124 | -0.403 | -1.989 | 0.000 | 0.037 |  |
| up in PF4777369 vs ctr | URS_ADIPOCYTE_DIFFERENTIATION_DN | 16 | -0.643 | -1.984 | 0.000 | 0.036 |  |
| up in PF4777369 vs ctr | TAKEDA_TARGETS_OF_NUP98_HOXA9_FUSION_6HR_DN | 24 | -0.567 | -1.953 | 0.000 | 0.043 |  |
| up in PF4777369 vs ctr | FRASOR_RESPONSE_TO_SERM_OR_FULVESTRANT_DN | 46 | -0.467 | -1.932 | 0.000 | 0.049 |  |
| up in PF4777369 vs ctr | RASHI_RESPONSE_TO_IONIZING_RADIATION_3 | 38 | -0.486 | -1.931 | 0.000 | 0.046 |  |
| up in PF4777369 vs ctr | KASLER_HDAC7_TARGETS_2_DN | 23 | -0.554 | -1.896 | 0.003 | 0.060 |  |
| up in PF4777369 vs ctr | KANNAN_TP53_TARGETS_UP | 45 | -0.455 | -1.867 | 0.000 | 0.072 |  |
| up in ctr vs PF4777369 | TIEN_INTESTINE_PROBIOTICS_6HR_UP | 52 | 0.595 | 2.151 | 0.000 | 0.006 | RPL23A, SEL1L3, TXN, RPL9, RPL13, RPS14, RPL37A, UBC, RPS6, B2M, RPS17, RPS11, RPL23, RPS23, RPL27A, TPT1, CDC5L, ACTB, RPL39, RPS24, GAPDH, ALDH3B1, RPS2, EEF1A1, RPLP0 |
| up in ctr vs PF4777369 | ZHAN_MULTIPLE_MYELOMA_CD1_VS_CD2_DN | 45 | 0.605 | 2.106 | 0.000 | 0.006 | NEK6, PRKCA, ZNF215, RAPGEF5, OSBPL10, PRKCB, SC5DL, CSNK1G3, UBE2D2, BCL11A, STXBP6, TLK1, CORO1C, PPAP2B, BLNK, CD27, KLHL14, STAP1, SLFN11 |
| up in ctr vs PF4777369 | WONG_MITOCHONDRIA_GENE_MODULE | 195 | 0.481 | 2.095 | 0.000 | 0.005 | SCO1, SLC25A19, NDUFB6, MRPS30, COX6C, MRPS33, DECR1, NDUFB3, COX7B, MCEE, MRPL45, MRPS28, GRPEL2, UQCRB, SLC25A17, HINT2, AIFM1, NDUFS4, MRPS18B, CRYZ, PDHX, MRPL49, MRPS36, NDUFA8, NDUFA9, PDHA1, MRPS15, MTX2, ACAT1, PDHB, PTRH2, MRPL36, NDUFB9, GSTZ1, NDUFA10, MRPS18C, AK2, ATP6V1C1, MRPL18, SFXN5, TIMM8B, DLD, ATP5O, COX5B, NDUFAF1, TOMM7, NDUFA1, ATP5F1, ATP5J, CYC1, CYB5A, ATP5H, TIMM10, NDUFB5, DBT, ATP5L, ATP5G1, ATP5C1, ATP5B, PRDX5, HAX1, COX7C, NDUFA6, MRPL13, SOD2, MIPEP, UQCRQ, FIBP, TUFM, MRPL11, TOMM70A, NDUFAB1, MRPL3, GLUD1, SDHB, KARS, MRPL40, GPD2, COX7A2, NDUFA5, DUT, MRPL42, NDUFS2, NQO1, PDK3, SUCLA2, ATP5G3, PCCB, ATOX1, FH, NDUFS5, ATP5J2 |
| up in ctr vs PF4777369 | SCHMIDT_POR_TARGETS_IN_LIMB_BUD_UP | 22 | 0.694 | 2.079 | 0.000 | 0.006 | SQLE, INSIG1, HMGCS1, DHCR7, ACAT2, IDI1, SC5DL, FDFT1, LDLR, ELOVL6, LSS, MVK, FDPS |
| up in ctr vs PF4777369 | MILI_PSEUDOPODIA_HAPTOTAXIS_UP | 422 | 0.448 | 2.066 | 0.000 | 0.005 | NUDCD2, DMXL1, TTC35, UBR3, TTC1, ARPP19, HMGCS1, I153CCNG1, AASDHPPT, CAB39L, KIAA1826, VPS41, SLC25A46, RBM34, ZNF131, DCAF6, IFT74, NUDT2, TAF9, EXOSC9, COPS2, BZW1, MFF, IDI1, TOPORS, ZDHHC2, MRRF, NUP54, UBE2D2, UBE2V2, NXT2, TXNDC9, RPF2, ASNSD1, ABCB7, EFR3A, DDX10, OXR1, WDR75, UBE2D3, UCHL5, MRPS18C, LARP7, MAK16, ATF2, COMMD8, LIMS1, IFIT2, SF3B1, RPL31, RABEP1, CISD2, DNAJB4, KIF2A, PNO1, PIK3C2A, GBAS, KCTD10, CYB5R4, MBIP, CISD1, SUMO1, WWP1, CDC5L, TSR1, TRIP4, SH3BGRL, INTS8, USP16, UGP2, CENPP, ANKRD13C, ACSL4, GSPT1, CUL4B, MRPL47, PJA2, SERF1A, SELT, ABI2, UTP14A, SLTM, NAMPT, PSMC3IP, RFWD2, ARMCX3, PDCD10, TMLHE, BCLAF1, FASTKD2, YWHAB, MRE11A, MRPL13, RPL17, VBP1, C1D, ZMAT2, INTS6, PRPF40A, NCOR1, CCDC90B, SAMD9L, TRMT11, PPWD1, SH3GLB1, AGTPBP1, TOMM70A, TXNL1, CHMP2B, EIF3E, NAP1L1, TIAL1, ARL5A, SCRN3, ATE1, RPAP2, TCF12, UBLCP1, VAMP4, RWDD1, PAIP1, AGFG1, THUMPD3, PPP1R7, YTHDF2, SLU7, TLK2, USP46, PTPN2, PIP4K2A, BTBD1, NR3C1, PSMD6, SUCLA2, CAMK2D, PDK1, R3HDM1, RPS6KA3, GTPBP4, PAPD4, STAG2, UFSP2, HSPA9, CSTF2, NFIA, PKP4, YME1L1, XPO1, VPS13A, GPBP1, CRYZL1, PRIM1, MTAP, CNOT7 |
| up in ctr vs PF4777369 | HORTON_SREBF_TARGETS | 22 | 0.689 | 2.051 | 0.000 | 0.005 |  |
| up in ctr vs PF4777369 | MOOTHA_VOXPHOS | 76 | 0.518 | 1.994 | 0.000 | 0.013 |  |
| up in ctr vs PF4777369 | STEIN_ESRRA_TARGETS_UP | 316 | 0.441 | 1.979 | 0.000 | 0.015 |  |
| up in ctr vs PF4777369 | SHEN_SMARCA2_TARGETS_UP | 375 | 0.428 | 1.976 | 0.000 | 0.014 |  |
| up in ctr vs PF4777369 | HAMAI_APOPTOSIS_VIA_TRAIL_UP | 438 | 0.419 | 1.946 | 0.000 | 0.023 |  |
| up in ctr vs PF4777369 | GARY_CD5_TARGETS_DN | 392 | 0.421 | 1.930 | 0.000 | 0.027 |  |
| up in ctr vs PF4777369 | LASTOWSKA_NEUROBLASTOMA_COPY_NUMBER_UP | 143 | 0.459 | 1.918 | 0.000 | 0.030 |  |
| up in ctr vs PF4777369 | PUIFFE_INVASION_INHIBITED_BY_ASCITES_DN | 117 | 0.463 | 1.911 | 0.000 | 0.030 |  |
| up in ctr vs PF4777369 | CHIANG_LIVER_CANCER_SUBCLASS_UNANNOTATED_DN | 174 | 0.438 | 1.878 | 0.000 | 0.046 |  |
| up in ctr vs PF4777369 | MOOTHA_HUMAN_MITODB_6_2002 | 371 | 0.405 | 1.854 | 0.000 | 0.058 |  |
| up i n combo2 vs ctr | NIKOLSKY_BREAST_CANCER_20Q12_Q13_AMPLICON | 87 | -0.624 | -2.667 | 0.000 | 0.000 | STX16, CEBPB, ADNP, ARFGAP1, TCEA2, ARFGEF2, PTK6, PFDN4, RAE1, PRPF6, ZBTB46, NCOA3, RGS19, OGFR, YTHDF1, SOX18, SPATA2, OPRL1, ARFRP1, PPDPF, UCKL1, MYT1, RBM38, SAMD10, TOMM34, RPS21, ATP5E, BCAS4, ZGPAT, DNAJC5, UBE2V1, ZNF217, STMN3, TH1L, CTCFL, RAB22A, TPD52L2, NPEPL1, SYS1, EEF1A2, PCMTD2, ADRM1, SLC17A9, AURKA, SLCO4A1, PREX1, TCFL5 |
| up i n combo2 vs ctr | NIKOLSKY_BREAST_CANCER_1Q21_AMPLICON | 27 | -0.744 | -2.471 | 0.000 | 0.000 | PBXIP1, EFNA1, GBA, THEM4, CKS1B, ZBTB7B, RUSC1, CLK2, DPM3, EFNA4, HCN3, MUC1, SCAMP3, TRIM46, FAM189B, SHC1, FLAD1, PYGO2, KRTCAP2 |
| up i n combo2 vs ctr | NIKOLSKY_BREAST_CANCER_16P13_AMPLICON | 68 | -0.591 | -2.442 | 0.000 | 0.000 | SNRNP25, NARFL, TBL3, TSC2, ITFG3, FLYWCH2, DECR2, JMJD8, NPW, FBXL16, GNPTG, HAGH, SPSB3, RPUSD1, THOC6, MRPS34, NTHL1, CHTF18, FLYWCH1, RHOT2, IL32, CLCN7, WDR90, NME3, FAM173A, FAM195A, SYNGR3, HAGHL, PRSS21 |
| up i n combo2 vs ctr | REICHERT_MITOSIS_LIN9_TARGETS | 24 | -0.696 | -2.252 | 0.000 | 0.002 | CCNB1, TOP2A, VCPIP1, PLK1, MKI67, CCNF, CENPA, KIF20A, AURKA, CDCA2, KIF2C, CENPF |
| up i n combo2 vs ctr | BURTON_ADIPOGENESIS_PEAK_AT_24HR | 33 | -0.629 | -2.224 | 0.000 | 0.002 | CENPL, CCNB1, TOP2A, SNRNP70, PRC1, RANGAP1, EXOSC5, GALK1, ANLN, UBE2C, TRAPPC5, H2AFX, TK1, CDC20, RACGAP1, KIF20A, BUB1, CAD |
| up i n combo2 vs ctr | CAIRO_PML_TARGETS_BOUND_BY_MYC_UP | 19 | -0.709 | -2.153 | 0.000 | 0.004 |  |
| up i n combo2 vs ctr | NIKOLSKY_BREAST_CANCER_12Q13_Q21_AMPLICON | 34 | -0.595 | -2.093 | 0.000 | 0.007 |  |
| up i n combo2 vs ctr | NIKOLSKY_BREAST_CANCER_20Q11_AMPLICON | 23 | -0.656 | -2.075 | 0.000 | 0.008 |  |
| up i n combo2 vs ctr | HAMAI_APOPTOSIS_VIA_TRAIL_DN | 124 | -0.455 | -2.051 | 0.000 | 0.011 |  |
| up i n combo2 vs ctr | NIKOLSKY_BREAST_CANCER_11Q12_Q14_AMPLICON | 103 | -0.460 | -2.009 | 0.000 | 0.017 |  |
| up i n combo2 vs ctr | HOFMANN_MYELODYSPLASTIC_SYNDROM_RISK_UP | 15 | -0.697 | -1.992 | 0.000 | 0.020 |  |
| up i n combo2 vs ctr | GINESTIER_BREAST_CANCER_ZNF217_AMPLIFIED_DN | 261 | -0.400 | -1.989 | 0.000 | 0.019 |  |
| up i n combo2 vs ctr | PARK_APL_PATHOGENESIS_DN | 38 | -0.565 | -1.986 | 0.000 | 0.018 |  |
| up i n combo2 vs ctr | PARK_TRETINOIN_RESPONSE_AND_PML_RARA_FUSION | 25 | -0.607 | -1.980 | 0.006 | 0.018 |  |
| up i n combo2 vs ctr | AMUNDSON_GAMMA_RADIATION_RESPONSE | 39 | -0.551 | -1.980 | 0.000 | 0.017 |  |
| up in ctr vs combo2 | NIKOLSKY_BREAST_CANCER_16Q24_AMPLICON | 35 | 0.792 | 2.491 | 0.000 | 0.000 | RPL13, CDT1, TRAPPC2L, KLHDC4, BANP, SPG7, COX4NB, APRT, GALNS, CENPBD1, MAP1LC3B, ANKRD11, GINS2, CTU2, TCF25, DEF8, GAS8, CHMP1A, CDK10, TUBB3, SPATA2L, CBFA2T3, CYBA |
| up in ctr vs combo2 | ROYLANCE_BREAST_CANCER_16Q_COPY_NUMBER_UP | 40 | 0.728 | 2.318 | 0.000 | 0.000 | GINS3, CNOT1, AKTIP, GOT2, GPR114, COQ9, PLCG2, CENPBD1, CHD9, CMIP, FTO, PSMD7, RBL2, TCF25, DEF8, RSPRY1, POLR2C, CPNE2, GAS8 |
| up in ctr vs combo2 | LINDGREN_BLADDER_CANCER_CLUSTER_2A_DN | 108 | 0.557 | 2.058 | 0.000 | 0.005 | BMP2K, DPYSL2, ANXA5, PHF17, NEK6, UBE2E2, CNN3, DCBLD2, ACSL4, CCDC50, MT2A, SLFN11, MT1H, CTNNAL1, MYO5A, MT1E, NGRN, RCAN2, ZAK, NFIL3, SGCB, SDCBP, MT1X, PTPDC1, GNG10, DNAJC10, LIG4, PMP22, ALCAM, AP1S2, RCAN1, ST6GAL1, DUSP22, PPP6C, BEX2, RNF38, UGCG, KIF2A, EVI2A, MS4A4A |
| up in ctr vs combo2 | YANG_BREAST_CANCER_ESR1_UP | 21 | 0.745 | 2.036 | 0.000 | 0.005 | LRBA, WFS1, ABAT, TFF3, XBP1, GREB1, TBC1D9, WWP1, FBP1, BBS4 |
| up in ctr vs combo2 | ZHAN_MULTIPLE_MYELOMA_CD1_VS_CD2_DN | 45 | 0.634 | 2.011 | 0.000 | 0.007 | ZNF215, RNGTT, STAP1, NEK6, RAPGEF5, KLHL14, OSBPL10, BCL11A, SLFN11, TBC1D1, CSNK1G3, TLK1, PRKCB, PIK3AP1, BLNK, DUS2L, PRKCA, CD27, PPAP2B, RASSF6, ST6GAL1, TNS3, LAPTM4B, RYK, NAV2 |
| up in ctr vs combo2 | MILI_PSEUDOPODIA_HAPTOTAXIS_UP | 422 | 0.467 | 1.985 | 0.000 | 0.010 |  |
| up in ctr vs combo2 | FLECHNER_BIOPSY_KIDNEY_TRANSPLANT_OK_VS_DONOR_UP | 447 | 0.462 | 1.970 | 0.000 | 0.012 |  |
| up in ctr vs combo2 | PROVENZANI_METASTASIS_UP | 157 | 0.507 | 1.969 | 0.000 | 0.010 |  |
| up in ctr vs combo2 | JOHNSTONE_PARVB_TARGETS_1_DN | 43 | 0.605 | 1.943 | 0.000 | 0.014 |  |
| up in ctr vs combo2 | GENTILE_UV_LOW_DOSE_DN | 48 | 0.573 | 1.894 | 0.000 | 0.028 |  |
| up in ctr vs combo2 | DITTMER_PTHLH_TARGETS_DN | 62 | 0.545 | 1.874 | 0.000 | 0.034 |  |
| up in ctr vs combo2 | SHEN_SMARCA2_TARGETS_UP | 375 | 0.442 | 1.871 | 0.000 | 0.034 |  |
| up in ctr vs combo2 | IZADPANAH_STEM_CELL_ADIPOSE_VS_BONE_UP | 99 | 0.503 | 1.871 | 0.000 | 0.031 |  |
| up in ctr vs combo2 | WENG_POR_TARGETS_LIVER_UP | 25 | 0.644 | 1.866 | 0.001 | 0.031 |  |
| up in ctr vs combo2 | KENNY_CTNNB1_TARGETS_DN | 37 | 0.593 | 1.863 | 0.000 | 0.030 |  |
